# Synthesis of Indomorphan Pseudo‐Natural Product Inhibitors of Glucose Transporters GLUT‐1 and ‐3

**DOI:** 10.1002/anie.201909518

**Published:** 2019-10-07

**Authors:** Javier Ceballos, Melanie Schwalfenberg, George Karageorgis, Elena S. Reckzeh, Sonja Sievers, Claude Ostermann, Axel Pahl, Magnus Sellstedt, Jessica Nowacki, Marjorie A. Carnero Corrales, Julian Wilke, Luca Laraia, Kirsten Tschapalda, Malte Metz, Dominik A. Sehr, Silke Brand, Konstanze Winklhofer, Petra Janning, Slava Ziegler, Herbert Waldmann

**Affiliations:** ^1^ Department of Chemical Biology Max-Planck-Institute of Molecular Physiology Otto-Hahn-Strasse 11 44227 Dortmund Germany; ^2^ Faculty of Chemistry and Chemical Biology Technical University Dortmund Otto-Hahn-Strasse 6 44227 Dortmund Germany; ^3^ Compound Management and Screening Center, Dortmund Otto-Hahn-Strasse 11 44227 Dortmund Germany; ^4^ Department of Chemistry Umeå University 901 87 Umeå Sweden; ^5^ Department of Molecular Cell Biology Institute of Biochemistry and Pathobiochemistry Ruhr University Bochum 44801 Bochum Germany; ^6^ Current address: Laboratory of Catalysis and Organic Synthesis EPFL SB ISIC LCSO, BCH 4221 1015 Lausanne Switzerland; ^7^ Current address: School of Chemistry University of Leeds Leeds LS2 9JT UK; ^8^ Current address: Department of Chemistry Technical University of Denmark Kemitorvet, Bygning 207 2800 Kgs Lyngby Denmark; ^9^ Current address: Clinical Chemistry, Laboratory Medicine University Hospital of Umeå 901 85 Umeå Sweden

**Keywords:** antitumor agents, glucose transporters, inhibitors, natural products, pseudo-natural products

## Abstract

Bioactive compound design based on natural product (NP) structure may be limited because of partial coverage of NP‐like chemical space and biological target space. These limitations can be overcome by combining NP‐centered strategies with fragment‐based compound design through combination of NP‐derived fragments to afford structurally unprecedented “pseudo‐natural products” (pseudo‐NPs). The design, synthesis, and biological evaluation of a collection of indomorphan pseudo‐NPs that combine biosynthetically unrelated indole‐ and morphan‐alkaloid fragments are described. Indomorphane derivative Glupin was identified as a potent inhibitor of glucose uptake by selectively targeting and upregulating glucose transporters GLUT‐1 and GLUT‐3. Glupin suppresses glycolysis, reduces the levels of glucose‐derived metabolites, and attenuates the growth of various cancer cell lines. Our findings underscore the importance of dual GLUT‐1 and GLUT‐3 inhibition to efficiently suppress tumor cell growth and the cellular rescue mechanism, which counteracts glucose scarcity.

## Graphical abstract

**Combination therapy**: Indomorphan pseudo‐natural products (pseudo‐NPs) have been synthesized that combine biosynthetically unrelated indole and morphan alkaloid fragments. This new glucose uptake inhibitor chemotype selectively targets and upregulates the glucose transporters GLUT‐1 and GLUT‐3 and inhibits the growth of cancer cells.

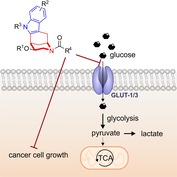

## Introduction

Strategies for the design and discovery of novel chemical matter endowed with bioactivity can draw from previous insight about the biological relevance of compound classes. This reasoning underlies, for example, biology‐oriented synthesis (BIOS)^1^ and ring‐distortion and/or ‐modification approaches (“complexity to diversity”; CtD).^2^ Limitations of BIOS derive from restricted coverage of natural product like chemical space and biological target space. These limitations may be overcome by the combination of BIOS with fragment‐based design^3^ to arrive at “pseudo‐natural products”.^4^ Pseudo‐natural products are obtained by the de novo combination of natural product fragments^5^ that generate unprecedented compound classes not accessible by known biosynthesis pathways, and, therefore, go beyond the chemical space explored by nature. They may have new biological targets and modes of action, such that their bioactivity will best be evaluated in unbiased target‐agnostic cell‐based assays covering a wide range of biological processes.^6^

**Figure 1:**
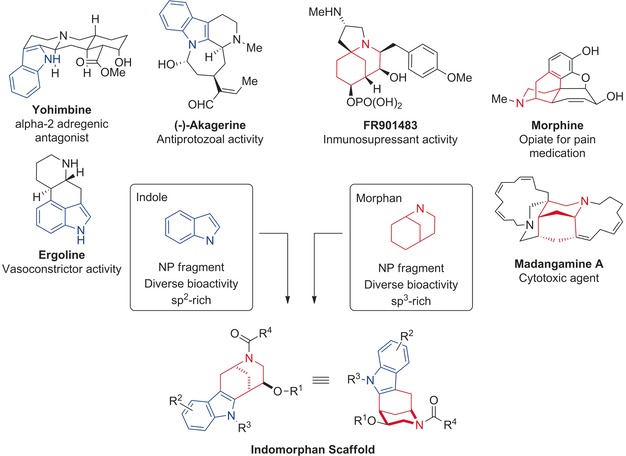
Design of the indomorphan pseudo‐NP class based on the natural product fragments derived from indole (in blue) and morphan (in red) alkaloids.

Here we describe the design, synthesis, and biological investigation of indomorphan pseudo‐natural products that contain characteristic structural fragments of the biosynthetically unrelated indole^7^ and morphan^8^ alkaloid classes, each of which is endowed with diverse and different bioactivities (Figure 1). A piperidine fragment is attached in an edge‐on connection to an indole moiety in numerous polycyclic indole alkaloids,^9^ whereas a benzene ring is linked to a piperidine‐containing fragment through a bridge in the scaffold of the morphinan alkaloids.^9^ In the indomorphans, these fragments are merged to a bridged, bicyclic indolylethylamine‐containing novel pseudo‐natural product class (Figure 1).

Biological investigation of the compound collection revealed that the indomorphans define a truly novel, structurally unprecedented glucose uptake inhibitor chemotype. The most potent inhibitor, termed Glupin, selectively blocks glucose uptake mediated by GLUT‐1 and GLUT‐3 with low nanomolar potency, inhibits glycolysis, and efficiently suppresses the growth of various cancer cell lines.

## Results

**Scheme 1:**
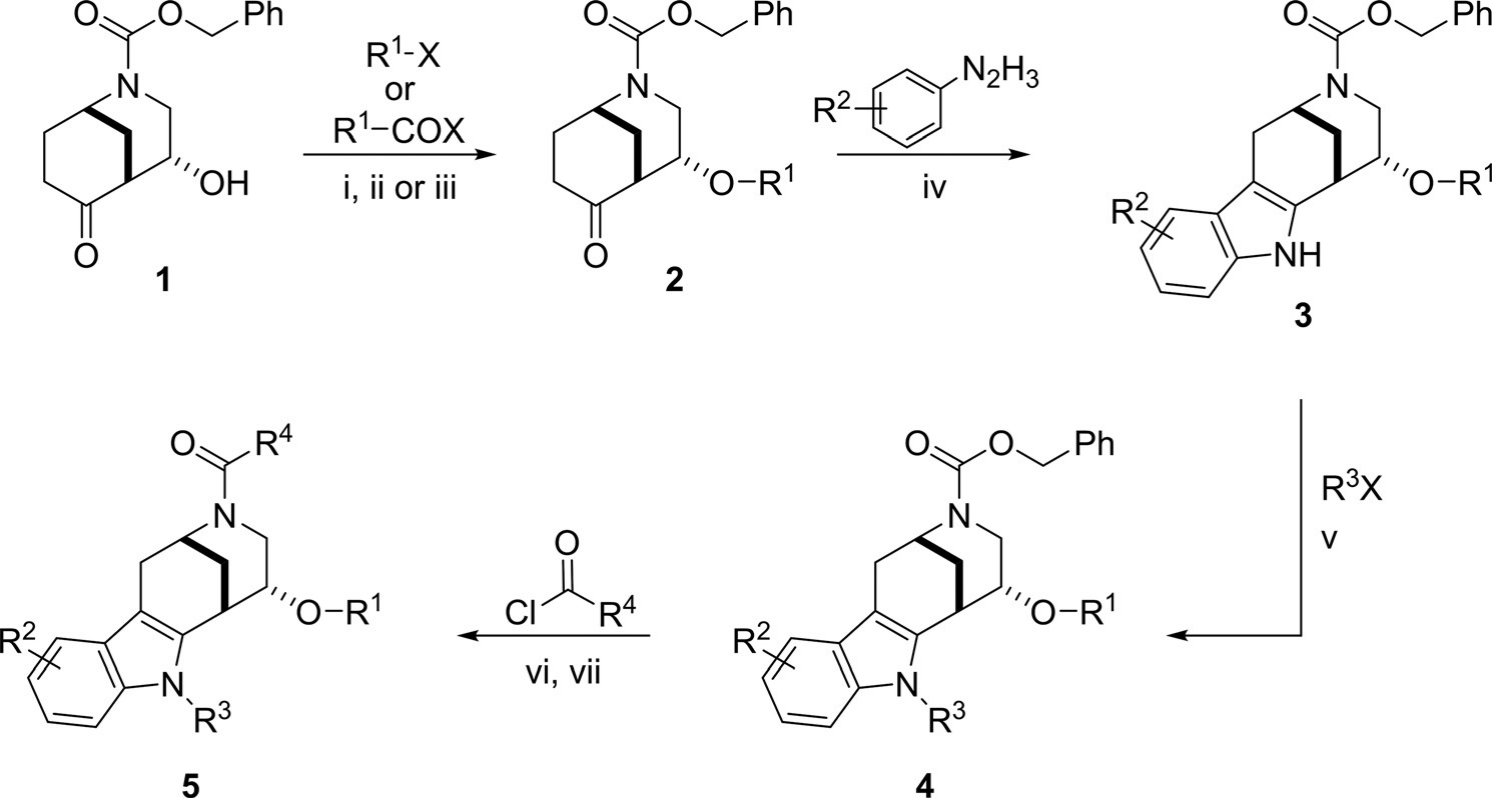
Synthesis of the indomorphan pseudo‐NP class. i) MeI (5 equiv), Ag_2_O (5 equiv), CH_2_Cl_2_, 72 h, rt; ii) AcCl, CH_2_Cl_2_/pyridine, 3 h, −10 °C; iii) allyl ethylcarbonate (2 equiv) Pd(PPh_3_)_4_ (5 mol %), THF, 80 °C, 2.5 h; iv) ArN_2_H_3_⋅HCl (1 equiv), AcOH, reflux, 1.5 h; v) R^3^Br (3 equiv), Cs_2_CO_3_ (3 equiv), DMF, 6 h, rt; vi) H_2_, Pd/C, EtOH, 5 h, rt; vii) ArCOCl (1.0 equiv), NEt_3_ (1.2 equiv), CH_2_Cl_2_, 12 h, rt.

For the synthesis of the indomorphan library, bicyclic ketone **1** 10 (Scheme 1) was derivatized to yield ethers **2**, which were then transformed into different indole derivatives **3** by means of the Fisher indole synthesis. Alkylation of the indole nitrogen atom (→**4**), deprotection, and acylation of the morphan nitrogen atom yielded indomorphans **5**. All the transformations are preparatively simple and have wide scope. The synthesis yielded 43 indomorphans in five linear steps. In addition, 19 selected indomorphans were obtained commercially^11^ (see Table S1 in the Supporting Information).

Calculation of the natural product score (NP‐score) distribution^12^ of this pseudo‐NP class, and comparison with the scores calculated for the NP set in ChEMBL,^13^ as well as for the set of marketed and experimental drugs listed in DrugBank,^14^ revealed that indomorphans exhibit a narrow distribution in a part of the NP‐score graph that is not heavily populated by NPs but rather by synthetically accessible biologically relevant molecules (Figure S2 a). Analysis of the principal moments of inertia (PMI),^15^ used as a crude measure of molecular shape, revealed that these pseudo‐NPs possess a higher degree of three‐dimensional character than common synthetically accessible compound collections, which reside typically on the rod‐like to disc‐like vertex of a PMI plot (Figure S2 b).^16^ In addition, indomorphans display similar shape characteristics as indole‐ or morphan‐containing NPs and non‐naturally occurring biologically relevant compounds (see Figure S3 and Schemes S3 and S4). Further analysis using “Lipinski‐Ro5” criteria, showed that 61 % of this collection is included within the limits of drug‐like space (see Figure S2c and Table S2), thus indicating that de novo combinations of NP‐derived fragments may result in compound collections with desirable properties.

The indomorphans were investigated in several cell‐based assays that monitor biological signaling, for example, the Wnt and Hedgehog pathway, or metabolic processes such as autophagy and glucose uptake. The indomorphans proved to be inhibitors of glucose uptake in HCT116 cells, with the most potent compounds showing IC_50_ values in the very low nanomolar range (see below). Cancer cells meet their increased demand for metabolic energy by increasing glucose uptake, accelerated glycolysis, and conversion of pyruvate into lactate, even in the presence of oxygen (this phenomenon is termed the Warburg effect).^17^ To this end, tumors frequently upregulate facilitative, high‐affinity glucose transporters (GLUTs). Several small molecules have been reported to inhibit glucose transporters (for a review, see Granchi et al.^18^). However, many of them displayed low potency or selectivity. Very recently BAY‐876, a potent and GLUT‐1‐selective inhibitor was developed by Bayer.^19^ However, both GLUT‐1 and GLUT‐3 are upregulated in various cancers^20^ and GLUT‐3 expression can increase upon radio‐chemotherapy or primary surgery. Therefore, cells overexpressing GLUT‐3 may possess a survival advantage and may be clonally selected.^21^ Moreover, induction of *GLUT3* expression has been reported to be a protective mechanism against hypoglycemia in neurons,^22^ and cancer cells respond similarly to decreased glucose levels.^23^ Thus, efficient inhibition of glucose uptake in tumors and cancer cells may require simultaneous targeting of both GLUT‐1 and GLUT‐3, and we recently developed the first GLUT‐1/GLUT‐3‐selective compounds.4b, 24

The glucose uptake assay monitored the inhibition of 2‐deoxyglucose (2DG) uptake as described by Yamamoto et al.^25^ Briefly, 2DG uptake is determined by quantification of the 2DG‐6‐phosphate (2DG6P) that accumulates inside the cells by using the diaphorase‐resazurin/‐resorufin system (see Figure S7 a). Several indomorphans inhibited 2DG uptake with IC_50_ values ranging from submicromolar to low micromolar levels (Table 1 and Table S1), with compound **5 b** (Table 1, entry 2) being the most active with an IC_50_ value of 53±23 nm.

**Table 1:** Structure‐activity relationship analysis for the indomorphan class (see Table S1 for further details). 
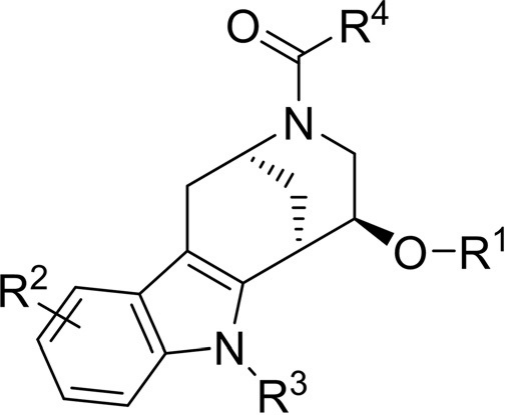

Entry	Compound	R^1^	R^2^	R^3^	R^4^	IC_50_ [μm] ^[a]^
1	(±)‐Glupin (**5 a**)	Me	H	CH_2_CO_2_Et	2‐(methylthio)pyridin‐3‐yl	0.055±0.017
2	**5 b**	Ac	H	CH_2_CO_2_Et	2‐(methylthio)pyridin‐3‐yl	0.053±0.023
3	**5 c**	H	H	CH_2_CO_2_Et	2‐(methylthio)pyridin‐3‐yl	0.13±0.05
4	**5 d**	^*n*^Pr	H	CH_2_CO_2_Et	2‐(methylthio)pyridin‐3‐yl	0.087±0.015
5	**5 e**	PEG*^[b]^	H	CH_2_CO_2_Et	2‐(methylthio)pyridin‐3‐yl	0.083±0.026
6	**5 f**	Me	5‐OH	CH_2_CO_2_Et	2‐(methylthio)pyridin‐3‐yl	0.10±0.06
7	**5 g**	Me	5‐Cl	CH_2_CO_2_Et	2‐(methylthio)pyridin‐3‐yl	1.4±0.2
8	**5 h**	Me	7‐Cl	CH_2_CO_2_Et	2‐(methylthio)pyridin‐3‐yl	0.21±0.09
9	**5 i**	Me	5‐CO_2_Et	CH_2_CO_2_Et	2‐(methylthio)pyridin‐3‐yl	>30
10	**5 j**	Me	H	H	2‐(methylthio)pyridin‐3‐yl	>30
11	**5 k**	Me	H	CH_2_CO_2_^*t*^Bu	2‐(methylthio)pyridin‐3‐yl	0.19±0.06
12	**5 l**	Me	H	CH_2_CO_2_^*n*^Pr	2‐(methylthio)pyridin‐3‐yl	0.093±0.035
13	**5 m**	Me	H	CH_2_CONHMe	2‐(methylthio)pyridin‐3‐yl	1.45±0.6
14	**5 n**	Me	H	CH_2_CO_2_H	2‐(methylthio)pyridin‐3‐yl	10.0±4.0
15	**5 o**	Me	H	furan‐2‐ylmethyl	2‐(methylthio)pyridin‐3‐yl	6.7±2.4
16	**5 p**	Me	H	CO_2_Et	2‐(methylthio)pyridin‐3‐yl	28±2
17	**5 q**	Me	H	(CH_2_)_2_CO_2_Et	2‐(methylthio)pyridin‐3‐yl	8.4±3.6
18	**5 r**	Me	H	CH_2_CO_2_Et	*N*‐morpholino	3.16+ ±0.6
19	**5 s**	Me	H	CH_2_CO_2_Et	2‐furyl	3.4±0.3
20	**5 t**	Me	H	CH_2_CO_2_Et	pyridine‐3‐yl	2.3±0.8
21	**5 u**	Me	H	CH_2_CO_2_Et	2‐fluoropyridin‐3‐yl	0.54±0.16
22	**5 v**	Me	H	CH_2_CO_2_Et	2‐methoxypyridin‐3‐yl	0.23±0.05
23	**5 w**	Me	H	CH_2_CO_2_Et	2‐trifluoromethylpyridin‐3‐yl	0.12±0.03
24	**5 x**	Me	H	CH_2_CO_2_Et	2‐(methylthio)‐4‐(trifluoromethyl)pyridin‐3‐yl	6.7±1.6
25	**5 y**	Me	H	CH_2_CO_2_Et	2‐(methylthio)‐5‐(trifluoromethyl)pyridin‐3‐yl	14±4

[a] IC_50_ values determined for the inhibition of 2DG uptake in HCT116 cells. Data are mean values (*N*≥3 independent experiments, *n*≥3 independent replicates). Error represents mean ±S.D. [b] PEG*: (2,2‐dimethyl‐4‐oxo‐3,9,12,15‐tetraoxa‐5‐azaoctadecan‐18‐yl)carbamic acid.

Investigation of the structure‐activity relationship (SAR) profile of the indomorphan scaffold revealed that potent analogues were obtained when small groups, such as hydrogen, methyl, or acetyl, were used at the R^1^ position (Table 1, entries 1–4). Even a PEG‐based linker introduced at R^1^ afforded a highly potent compound (entry 5). Substitution at the benzene ring (R^2^) strongly reduced the activity, particularly with substitution at position 5, but potency was maintained when a hydroxy group was introduced (entries 6–9). Alkylation of the indole nitrogen atom (R^3^) was essential for activity (entries 10–17). Derivatives with an ester functional group embedded in the substituent yielded the most potent compounds, whilst amides or a carboxylic acid resulted in a loss of potency. A one‐carbon linker to the ester group was found to be optimal (entries 16 and 17). These findings show that an ester group is best, although not absolutely necessary, for the highest activity. For the ethyl ester and the carboxylic acid we calculated the log *P* values to be 3.4 and 2.3, respectively. Moreover, calculation of the pharmacokinetic properties of the acid and the ethyl ester revealed similar predicted permeability in Caco2 cells, namely, 1.12 and 1.08 (log *P*_app_ values in 10^−6^ cm s^−1^) using the pkCSM tool^41^ and 38.9 and 23.0 using the preADMET tool.^42^ These data indicate that cell penetration by both the ester and the carboxylic acid is similar, such that the activity of the acid, if at all, represents only to a minor extent the possible hydrolysis of the ethyl ester in cellulo. This finding is further supported by the activity recorded for the *tert*‐butyl ester and the amide, both of which would be hydrolyzed only slowly or not at all. Nevertheless, we cannot fully rule out that the ethyl ester may represent a prodrug. To explore whether other substituents may be tolerated, furanylmethl derivative **5 o** (Table 1, entry 15) was synthesized. This analogue inhibited glucose import with an IC_50_ value of 6.6±2.4 μm. This heterocycle is not a full replacement of the ester, but the result indicates that other groups are tolerated at this site. Acylation of the morphan nitrogen atom (R^4^) was essential for the inhibition of 2DG uptake (entries 18–25). Activity in the low micromolar range was observed for several heterocycles and more potent analogues carried a functionalized nicotinate group. However, over‐functionalization of the pyridine ring was disadvantageous for the activity, as exemplified by the introduction of a CF_3_ group at positions 4 and 5 (entries 24 and 25).

**Figure 2:**
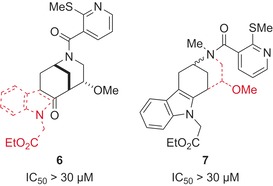
Structure and activity of the Glupin‐based fragment analogues **6** and **7**. Red dashed lines show the fragments missing from the indomorphan core, with R^1^ to R^4^ overall shown as in Glupin.

To investigate the contribution of the indole and morphan fragments on the bioactivity and to determine whether the novel fragment combination is required, we prepared indomorphan fragments **6** and **7** (Figure 2) which were decorated with the same functional groups as the active compound **5 a** (Table 1, entry 1). Notably, the inhibiting activity of 2‐DG uptake was not shared by the indolylethyl fragment, which lacks the morphan scaffold, or by the bicyclic ketomorphan fragment, which lacks the indole ring. These results suggest that the observed biological activity of the indomorphan scaffold is not shared by the individual NP fragments and is, therefore, a property of the new pseudo‐NP scaffold.

**Figure 3:**
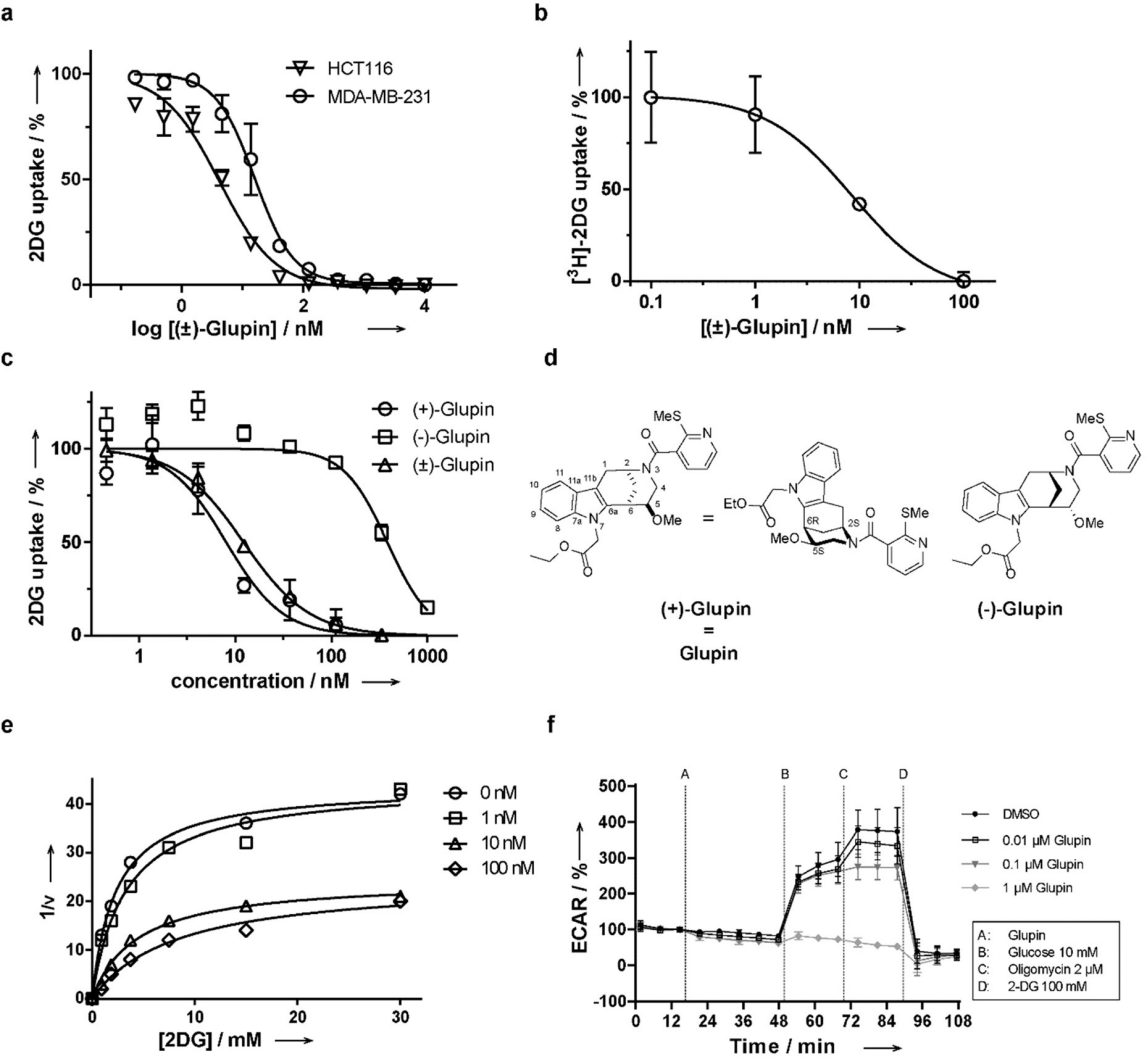
Inhibition of 2DG uptake by Glupin. a) (±)‐Glupin inhibition of 2DG uptake in MDA‐MB‐231 and in HCT116. Data are mean values ±SD (*N*=3, *n*=3). b) (±)‐Glupin inhibition of ^3^H‐2DG uptake in MDA‐MB‐231 cells. Cells were treated with compound or DMSO and 1 μCi ^3^H‐2DG for more than 30 min. Data are mean values ±SD (*N*=3, *n*=3). c) Glupin is the more potent enantiomer. 2DG uptake was measured in MDA‐MB‐231 cells. Data are mean values ±SEM (*n*=3). d) Structures of (+)‐Glupin (=Glupin) and the (−)‐enantiomer. e) The 2DG uptake in the presence of different amounts of Glupin was measured in a time‐ and concentration‐dependent manner in MDA‐MB‐231 cells and then analyzed by nonlinear regression using the Michaelis–Menten equation to determine the *V*_max_ and *K*_M,app_ values. f) Influence of Glupin on glycolysis. Glucose‐starved MDA‐MB‐231 cells were treated with Glupin and the extracellular acidification rate (ECAR) was measured over time in a Seahorse XFp Extracellular flux analyzer. Data are mean values ±SD (*n*=3).

Based on the potency and structure, compound **5 a**, termed (±)‐Glupin (from glucose uptake inhibitor; Table 1, entry 1), was selected for further analysis. (±)‐Glupin showed an IC_50_ value of 5±1 nm in separate, non‐automated determinations on HCT116 cells and also reduced the uptake of 2DG in the highly glycolytic human breast cancer cell line MDA‐MB‐231 (IC_50_=17±5 nm; Figure 3 a).^26^ This finding was further confirmed by the inhibition of tritium‐radiolabeled 2DG uptake by (±)‐Glupin with an IC_50_ value of 8±3 nm (Figure 3 b). (±)‐Glupin also potently suppressed 2DG uptake in HeLa (IC_50_=22±2 nm) and CHO cells (IC_50_=4±3 nm). (±)‐Glupin enantiomers were separated by preparative chiral HPLC, and the absolute configuration was assigned by means of chiral derivatization and independent synthesis of reference compounds (see the Supporting Information for details). The more potent (+)‐(2S, 5S, 6R)‐Glupin enantiomer inhibits 2DG uptake in MDA‐MB‐231 cells with IC_50_=4±2 nm, while (−)‐(2*R*,5*R*,6*S*)‐Glupin displays an IC_50_ value of 335±47 nm (Figure 3 c,d). (+)‐Glupin (hereafter referred to as “Glupin”) did not inhibit the downstream hexokinase, thus demonstrating direct interference with glucose uptake (see Figure S7 b).

Kinetic measurements in MDA‐MB‐231 cells showed that the maximum rate (*V*_max_) decreased, whereas the *K*_M_ value increased as the concentration of Glupin increased, thus indicating a mixed type of inhibition (Figure 3 e). Investigation of the influence of Glupin on the extracellular acidification rate (as a measure of glycolysis) and the oxygen consumption rate (as a measure of oxidative phosphorylation) of MDA‐MB‐231 cells revealed that 1 μm Glupin inhibited glycolysis to the level of glucose‐starved cells (Figure 3 f) and increased the oxygen consumption rate (Figure S8 a). Moreover, treatment of MOLT16 cells with 50 nm (±)‐Glupin reduced the levels of metabolites linked to glycolysis (glycerol‐3‐phosphate, lactic acid), the tricarboxylic acid (TCA) cycle (citric/isocitric acid), the pentose phosphate pathway (ribitol/arabitol), and the hexosamine biosynthetic pathway (UDP‐*N*‐acetylglucosamine), which all utilize glucose‐6‐phosphate (Table S3). The level of aspartate, which can fuel the TCA cycle, was elevated upon treatment with Glupin, most likely as a compensatory mechanism.^27^ These results are in agreement with inhibition of glucose uptake by Glupin and, consequently, the synthesis of glucose‐6‐phosphate.

In the absence of glucose, cells may utilize alternative energy sources and building blocks to sustain cell growth, in particular fatty acids, and altered lipid storage and metabolism is strongly connected with cancer malignancy.^28^ Treatment of MDA‐MB‐231 cells with Glupin in the presence of 400 μm oleic acid markedly reduced the number of lipid droplets with an IC_50_ value of 84.9 nm (Figure S8 b). Preformed lipid droplets were not affected, thus suggesting that Glupin has no stimulating effect on lipolysis (Figure S8 c).

**Figure 4:**
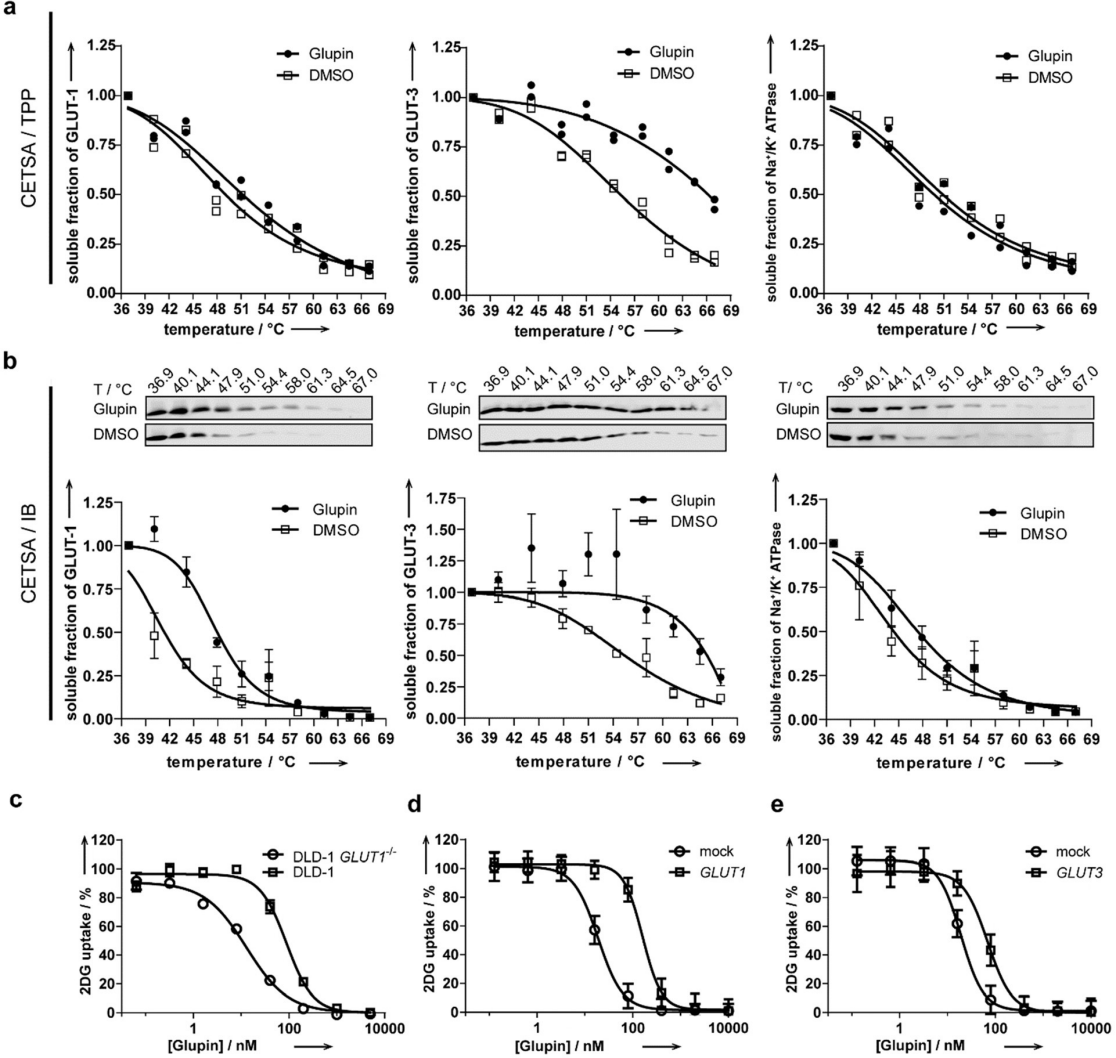
Target engagement of GLUT‐1 and GLUT‐3 by Glupin. The cellular thermal shift assay was performed with lysates from SW480 cells in the presence of 1 μm Glupin or DMSO using mass spectrometry (a) or immunoblotting (b) to detect GLUT‐1 or GLUT‐3 and Na^+^/K^+^‐ATPase as a control. Data of two biological replicates (a) or mean values ±SD and representative of *n*=3 (b) are shown. c) Uptake of 2DG in DLD‐1 versus DLD‐1 *GLUT1* (−/−) cells after incubation with the compound for 30 min. d) Uptake of 2DG in CHO cells that ectopically express GLUT‐1 compared to cells transfected with an empty vector (mock). e) Uptake of 2DG in CHO cells that ectopically express GLUT‐3 compared to cells transfected with an empty vector (mock). All data are mean values ±SD (*n*=3).

Target engagement was demonstrated by means of a proteome‐wide cellular thermal shift assay (CETSA, thermal proteome profiling, TPP)^29^ using SW480 cells, which revealed thermal stabilization of GLUT‐3 (Δ*T*_m_=7.1±0.4 °C) and GLUT‐1 (Δ*T*_m_=1.9±0.3 °C) in the presence of 1 μm Glupin (Figure 4 a). No further members of the GLUT family could be detected. A separate CETSA experiment using immunoblotting (Figure 4 b) confirmed this finding and showed thermal stabilization of GLUT‐1 (Δ*T*_m_=6.5±0.6 °C) and GLUT‐3 (Δ*T*_m_ >12 °C).

DLD‐1 *GLUT1* (−/−) cells do not express GLUT‐1 but exhibit substantial expression of GLUT‐3 (Figure S9).4b In contrast, the isogenic parental cell line DLD‐1 expresses mainly GLUT‐1 (Supporting Figure S9).4b, 30 Glupin inhibited the uptake of 2DG in both cell lines with IC_50_ values of 59.6±8.4 nm for DLD‐1 and 11.4±1.6 nm for DLD‐1 *GLUT1* (−/−; Figure 4 c). This finding confirms that Glupin targets both GLUT‐1 and GLUT‐3 and indicates that Glupin inhibits GLUT‐3 more strongly than GLUT‐1. Ectopic expression and, thereby, increased abundance of GLUT‐1 or GLUT‐3 in CHO cells4b (Figure S10 a,c), led to a partial rescue of the Glupin‐mediated inhibition of glucose uptake as detected by the increase in the IC_50_ values from 19.2±2.1 nm to 162±14 nm for GLUT‐1 overexpression (Figure 4 d) and from 19.1±2.9 nm to 68±7.2 nm for GLUT‐3 (Figure 4 e). In contrast, overexpression of GLUT‐2 or GLUT‐4 did not have any influence on the activity of the compound (Figures S10 b,d and S11 as well as Table S4). These results suggest that Glupin interacts with GLUT‐1 and GLUT‐3 but not with GLUT‐2 and GLUT‐4.

**Figure 5:**
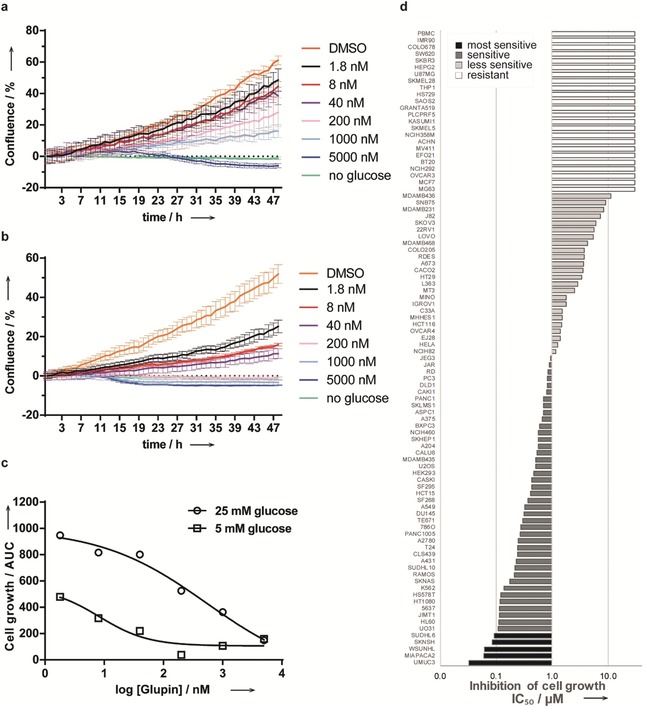
Inhibition of cancer cell growth by Glupin. a–c) The growth of MDA‐MB‐231 cells in 25 mm (a) or 5 mm glucose (b) was monitored in the presence of Glupin or DMSO by means of live‐cell kinetic analysis and confluence as a measure. Data are mean values (*N*=3) ±SD and representative of *n*=3. c) Comparison of cell growth at 5 and 25 mm glucose. The area under the curve (AUC) was calculated for the curves in (a) and (b). d) Glupin inhibits the growth of various cancer cell lines. 94 cell lines were treated with different concentrations of Glupin for 72 h followed by the sulforhodamine B assay and IC_50_ determination.

Real‐time analysis of the growth of the MDA‐MB‐231 cell line in the presence of Glupin showed a dose‐dependent suppression of cell growth in plain medium containing 25 mm glucose (Figure 5 a). This effect was much stronger at a physiological glucose concentration (5 mm; Figure 5 b,c). Exposure of the compound to more than 90 cancer cell lines and IMR‐90 and PBMC cells as noncancerous controls (see Figure 5 d and Table S5) revealed that the growth of the bladder cancer cell line UM‐UC‐3 (IC_50_=32 nm), the pancreas cancer cell line MIA PaCa‐2 (IC_50_=61 nm), the lymphoma cell lines WSU‐NHL and SU‐DHL‐6 (IC_50_=62 nm and 92 nm, respectively), and the neuroblastoma cell line SK‐N‐SH (IC_50_=84 nm), which depend on glucose for cell growth,^31^ was potently inhibited. IC_50_ values lower than 300 nm were obtained for more than 20 cell lines, whereas 24 cell lines were not affected by the compound, among them the peripheral blood mononuclear control cells (PBMCs). Sensitivity to Glupin was detected across various tissue types (Table S6). These results indicate that targeting glucose import by GLUT‐1 and GLUT‐3 may be a viable approach to inhibit the growth of cancer cells.

Since glucose depletion may lead to increased expression of GLUT‐1 and GLUT‐3,^22^, ^23^, ^32^ we analyzed the influence of Glupin on the expression of *GLUT1* (*SLC2A1*) and *GLUT3* (*SLC2A3*). The application of Glupin for 48 h induced only a slight increase in the expression of both genes at the mRNA level (Figure S12). However, treatment with 0.5 μm Glupin significantly increased GLUT‐1 levels after 24 h of treatment (Figure S13 a,b) and of GLUT‐3 levels after 48 h of treatment (Figure S13 c,d). These results indicate that Glupin‐mediated inhibition of glucose uptake increases the levels of both GLUT‐1 and GLUT‐3 in DLD‐1 cells.

## Discussion

Our results demonstrate that the combination of natural product (NP) fragments in unprecedented arrangements may yield biologically relevant pseudo‐NP collections with both novel NP‐inspired structure and novel bioactivity. The indomorphan pseudo‐NPs retain the characteristic structural elements of the guiding NPs but reside in an area of chemical space which is inaccessible to nature, reflected by the fact that they are not obtainable by known biosynthetic pathways. This novel, and structurally complex scaffold may be endowed by design with advantageous physiochemical properties, as the pseudo‐NP collection displays a NP‐score distribution closer to that of approved drugs.

The term “pseudo‐natural product” and reasoning related to the concept have inspired synthesis efforts before. Thus, Suga and co‐workers used the term to describe in vitro synthesized cyclic peptides embodying non‐natural amino acids,^33^ and Oshima and co‐workers qualified alkaloid‐like compounds obtained by the interception of biosynthetic pathways as pseudo‐NPs.^34^ Tietze et al.^35^ reviewed the syntheses of natural product hybrids, that is, NP‐like compounds which are composed of different NP substructures and may be considered pseudo‐NPs if they represent pseudo‐NP combinations that match our concept.4b, 5 In attempts to generate potential alkaloid types which might not have been identified in nature at the time, Morrison et al.^36^ replaced 3,4‐dihydroxyphenylalanine by tryptophan in a synthesis following the biogenetic route leading to the morphine scaffold. Bosch et al. (see Ref. 37 and references therein) developed heteromorphans, that is, compounds in which the benzene ring in 6,7‐benzomorphans is replaced by a heterocyclic ring, including an indomorphan analogue. “Unnatural” polyketide natural products have been obtained by means of synthetic biology approaches, for example, through precursor‐directed biosynthesis, mutasynthesis, or combinatorial biosynthesis (for reviews see Ref. 38), and natural and non‐natural structural elements have been combined in polyketide syntheses to yield natural product analogues not accessible by biosynthetic pathways (see for example, Ref. 39).

The indomorphan pseudo‐NPs define a novel glucose uptake inhibitor class with unprecedented chemotype. The most potent compound Glupin selectively targets glucose transporters GLUT‐1 and ‐3 and potently inhibits the growth of cancer cells. Our results demonstrate that inhibition of GLUT‐1 and GLUT‐3 may be necessary to efficiently inhibit the growth of cancer cells since both glucose transporters GLUT‐1 and GLUT‐3 may be upregulated upon withdrawal of glucose.^22^, ^23^, ^32^ Dual GLUT‐1‐ and GLUT‐3‐selective compounds such as Glupin would inhibit both transporters. Indeed, treatment with Glupin increased the expression of both GLUT‐1 and GLUT‐3 in DLD‐1 cells, which primarily express GLUT‐1. Upregulation of GLUT‐3 upon glucose deprivation also ensures the uptake of glucose at lower glucose concentrations in the cell environment compared to GLUT‐1 because of its higher affinity for glucose.^40^ Thus, in particular, the overexpression of GLUT‐3 may pose a hurdle for the efficacy of GLUT‐1‐selective agents and emphasizes the need to simultaneously target both proteins. These findings may inspire new drug discovery programs aimed at the modulation of tumor metabolism.

## Conflict of interest

The development of glucose transport inhibitors is an actively pursued drug discovery project at the Lead Discovery Center GmbH (LDC) of the Max Planck Gesellschaft, sponsored by H.W.

## Supporting information

As a service to our authors and readers, this journal provides supporting information supplied by the authors. Such materials are peer reviewed and may be re‐organized for online delivery, but are not copy‐edited or typeset. Technical support issues arising from supporting information (other than missing files) should be addressed to the authors.

Supplementary

## References

[1a] S. Wetzel , R. S. Bon , K. Kumar , H. Waldmann , Angew. Chem. Int. Ed. 2011, 50, 10800; 10.1002/anie.201007004 22038946

[1b] H. van Hattum , H. Waldmann , J. Am. Chem. Soc. 2014, 136, 11853. 25074019 10.1021/ja505861d

[2a] R. W. Huigens III , K. C. Morrison , R. W. Hicklin , T. A. Flood, Jr. , M. F. Richter , P. J. Hergenrother , Nat. Chem. 2013, 5, 195; 23422561 10.1038/nchem.1549 PMC3965367

[2b] R. J. Rafferty , R. W. Hicklin , K. A. Maloof , P. J. Hergenrother , Angew. Chem. Int. Ed. 2014, 53, 220; 10.1002/anie.201308743 24273016

[3] C. W. Murray , D. C. Rees , Nat. Chem. 2009, 1, 187. 21378847 10.1038/nchem.217

[4a] T. Schneidewind , S. Kapoor , G. Garivet , G. Karageorgis , R. Narayan , G. Vendrell-Navarro , A. P. Antonchick , S. Ziegler , H. Waldmann , Cell Chem. Biol. 2019, 26, 512; 30686759 10.1016/j.chembiol.2018.11.014

[4b] G. Karageorgis , E. S. Reckzeh , J. Ceballos , M. Schwalfenberg , S. Sievers , C. Ostermann , A. Pahl , S. Ziegler , H. Waldmann , Nat. Chem. 2018, 10, 1103. 30202104 10.1038/s41557-018-0132-6

[5] B. Over , S. Wetzel , C. Grutter , Y. Nakai , S. Renner , D. Rauh , H. Waldmann , Nat. Chem. 2013, 5, 21. 23247173 10.1038/nchem.1506

[6a] B. K. Wagner , S. L. Schreiber , Cell Chem. Biol. 2016, 23, 3; 26933731 10.1016/j.chembiol.2015.11.008 PMC4779180

[6b] A. Ursu , H. Waldmann , Bioorg. Med. Chem. Lett. 2015, 25, 3079. 26115575 10.1016/j.bmcl.2015.06.023

[7a] N. K. Kaushik , N. Kaushik , P. Attri , N. Kumar , C. H. Kim , A. K. Verma , E. H. Choi , Molecules 2013, 18, 6620; 23743888 10.3390/molecules18066620 PMC6270133

[7b] G. R. Humphrey , J. T. Kuethe , Chem. Rev. 2006, 106, 2875. 16836303 10.1021/cr0505270

[8a] G. Hofner , B. Streicher , B. Wunsch , Arch. Pharm. 2001, 334, 284; 10.1002/1521-4184(200109)334:8/9<284::aid-ardp284>3.0.co;2-b 11688139

[8b] J. B. Thomas , X. Zheng , S. W. Mascarella , R. B. Rothman , C. M. Dersch , J. S. Partilla , J. L. Flippen-Anderson , C. F. George , B. E. Cantrell , D. M. Zimmerman , F. I. Carroll , J. Med. Chem. 1998, 41, 4143; 9767649 10.1021/jm980290i

[8c] B. Bradshaw , C. Parra , J. Bonjoch , Org. Lett. 2013, 15, 2458. 23627688 10.1021/ol400926p

[9] P. M. Dewick , Medicinal Natural Products: A Biosynthetic Approach, Wiley, New York, 2002.

[10] F. Diaba , J. Bonjoch , Org. Biomol. Chem. 2009, 7, 2517. 19503923 10.1039/b906835j

[11] Selected indomorphans were obtained from Edelris, 115 Avenue Lacassagne, 69003 Lyon France; http://www.edelris.com/. Compounds commercialized by Edelris are available for purchase, but the company catalogue needs to be obtained individually from the company.

[12] P. Ertl , S. Roggo , A. Schuffenhauer , J. Chem. Inf. Model. 2008, 48, 68. 18034468 10.1021/ci700286x

[13] A. P. Bento , A. Gaulton , A. Hersey , L. J. Bellis , J. Chambers , M. Davies , F. A. Kruger , Y. Light , L. Mak , S. McGlinchey , M. Nowotka , G. Papadatos , R. Santos , J. P. Overington , Nucleic Acids Res. 2014, 42, D1083. 10.1093/nar/gkt1031 PMC3965067 24214965

[14] V. Law , C. Knox , Y. Djoumbou , T. Jewison , A. C. Guo , Y. Liu , A. Maciejewski , D. Arndt , M. Wilson , V. Neveu , A. Tang , G. Gabriel , C. Ly , S. Adamjee , Z. T. Dame , B. Han , Y. Zhou , D. S. Wishart , Nucleic Acids Res. 2014, 42, D1091. 10.1093/nar/gkt1068 PMC3965102 24203711

[15] W. H. Sauer , M. K. Schwarz , J. Chem. Inf. Comput. Sci. 2003, 43, 987. 12767158 10.1021/ci025599w

[16] I. Colomer , C. J. Empson , P. Craven , Z. Owen , R. G. Doveston , I. Churcher , S. P. Marsden , A. Nelson , Chem. Commun. 2016, 52, 7209. 10.1039/c6cc03244c 27145833

[17a] W. H. Koppenol , P. L. Bounds , C. V. Dang , Nat. Rev. Cancer 2011, 11, 325; 21508971 10.1038/nrc3038

[17b] M. G. Vander Heiden , L. C. Cantley , C. B. Thompson , Science 2009, 324, 1029. 19460998 10.1126/science.1160809 PMC2849637

[18] C. Granchi , S. Fortunato , F. Minutolo , MedChemComm 2016, 7, 1716. 28042452 10.1039/C6MD00287K PMC5198910

[19] H. Siebeneicher , A. Cleve , H. Rehwinkel , R. Neuhaus , I. Heisler , T. Muller , M. Bauser , B. Buchmann , ChemMedChem 2016, 11, 2261. 27552707 10.1002/cmdc.201600276 PMC5095872

[20a] R. E. Airley , A. Mobasheri , Chemotherapy 2007, 53, 233; 17595539 10.1159/000104457

[20b] P. Jóźwiak , A. Krześlak , L. Pomorski , A. Lipińska , Mol. Med. Rep. 2012, 6, 601; 22752218 10.3892/mmr.2012.969

[20c] A. Krzeslak , K. Wojcik-Krowiranda , E. Forma , P. Jozwiak , H. Romanowicz , A. Bienkiewicz , M. Brys , Pathol. Oncol. Res. 2012, 18, 721; 22270867 10.1007/s12253-012-9500-5 PMC3342495

[20d] P. Mellanen , H. Minn , R. Grenman , P. Harkonen , Int. J. Cancer 1994, 56, 622; 8314336 10.1002/ijc.2910560503

[20e] T. Yamamoto , Y. Seino , H. Fukumoto , G. Koh , H. Yano , N. Inagaki , Y. Yamada , K. Inoue , T. Manabe , H. Imura , Biochem. Biophys. Res. Commun. 1990, 170, 223; 2372287 10.1016/0006-291x(90)91263-r

[20f] M. Younes , R. W. Brown , M. Stephenson , M. Gondo , P. T. Cagle , Cancer 1997, 80, 1046. 9305704 10.1002/(sici)1097-0142(19970915)80:6<1046::aid-cncr6>3.0.co;2-7

[21] P. Fonteyne , V. Casneuf , P. Pauwels , N. Van Damme , M. Peeters , R. Dierckx , C. Van de Wiele , Histol. Histopathol. 2009, 24, 971. 19554504 10.14670/HH-24.971

[22] S. Nagamatsu , H. Sawa , N. Inoue , Y. Nakamichi , H. Takeshima , T. Hoshino , Biochem. J. 1994, 300, 125. 8198523 10.1042/bj3000125 PMC1138133

[23] A. Marín-Hernández , S. Y. López-Ramírez , I. Del Mazo-Monsalvo , J. C. Gallardo-Pérez , S. Rodríguez-Enriquez , R. Moreno-Sánchez , E. Saavedra , FEBS J. 2014, 281, 3325. 24912776 10.1111/febs.12864

[24] E. S. Reckzeh , G. Karageorgis , M. Schwalfenberg , J. Ceballos , J. Nowacki , M. C. M. Stroet , A. Binici , L. Knauer , S. Brand , A. Choidas , C. Strohmann , S. Ziegler , H. Waldmann , Cell. Chem. Biol. 2019, 10.1016/j.chembiol.2019.06.005. 31303578

[25a] N. Yamamoto , K. Kawasaki , K. Kawabata , H. Ashida , Anal. Biochem. 2010, 404, 238; 20494642 10.1016/j.ab.2010.05.012

[25b] N. Yamamoto , T. Sato , K. Kawasaki , S. Murosaki , Y. Yamamoto , Anal. Biochem. 2006, 351, 139. 16442489 10.1016/j.ab.2005.12.011

[26] I. F. Robey , A. D. Lien , S. J. Welsh , B. K. Baggett , R. J. Gillies , Neoplasia 2005, 7, 324. 15967109 10.1593/neo.04430 PMC1501147

[27] O. E. Owen , S. C. Kalhan , R. W. Hanson , J. Biol. Chem. 2002, 277, 30409. 12087111 10.1074/jbc.R200006200

[28] S. Beloribi-Djefaflia , S. Vasseur , F. Guillaumond , Oncogenesis 2016, 5, e189. 26807644 10.1038/oncsis.2015.49 PMC4728678

[29] H. Franken , T. Mathieson , D. Childs , G. M. Sweetman , T. Werner , I. Togel , C. Doce , S. Gade , M. Bantscheff , G. Drewes , F. B. Reinhard , W. Huber , M. M. Savitski , Nat. Protoc. 2015, 10, 1567. 26379230 10.1038/nprot.2015.101

[30] H. Siebeneicher , M. Bauser , B. Buchmann , I. Heisler , T. Muller , R. Neuhaus , H. Rehwinkel , J. Telser , L. Zorn , Bioorg. Med. Chem. Lett. 2016, 26, 1732. 26949183 10.1016/j.bmcl.2016.02.050

[31a] A. Daemen , D. Peterson , N. Sahu , R. McCord , X. N. Du , B. N. Liu , K. Kowanetz , R. Hong , J. Moffat , M. Gao , A. Boudreau , R. Mroue , L. Corson , T. O'Brien , J. Qing , D. Sampath , M. Merchant , R. Yauch , G. Manning , J. Settleman , G. Hatzivassiliou , M. Evangelista , Proc. Natl. Acad. Sci. USA 2015, 112, E4410; 10.1073/pnas.1501605112 PMC4538616 26216984

[31b] M. A. Lea , M. Altayyar , C. desBordes , Anticancer Res. 2015, 35, 5889; 26504012

[31c] X. X. Liu , L. Wang , W. Y. Jiang , W. H. Lu , J. Yang , W. B. Yang , J. Cancer 2018, 9, 1582. 29760796 10.7150/jca.24331 PMC5950587

[32] I. Guilletdeniau , A. Leturque , J. Girard , J. Cell Sci. 1994, 107, 487. 8006068

[33a] T. Ozaki , K. Yamashita , Y. Goto , M. Shimomura , S. Hayashi , S. Asamizu , Y. Sugai , H. Ikeda , H. Suga , H. Onaka , Nat. Commun. 2017, 8, 14207; 28165449 10.1038/ncomms14207 PMC5303826

[33b] Y. Goto , Y. Ito , Y. Kato , S. Tsunoda , H. Suga , Chem. Biol. 2014, 21, 766. 24856821 10.1016/j.chembiol.2014.04.008

[34a] H. Kikuchi , K. Ichinohe , S. Kida , S. Murase , O. Yamada , Y. Oshima , Org. Lett. 2016, 18, 5948; 27934494 10.1021/acs.orglett.6b03057

[34b] T. Asai , K. Tsukada , S. Ise , N. Shirata , M. Hashimoto , I. Fujii , K. Gomi , K. Nakagawara , E. N. Kodama , Y. Oshima , Nat. Chem. 2015, 7, 737. 26291946 10.1038/nchem.2308

[35] L. F. Tietze , H. P. Bell , S. Chandrasekhar , Angew. Chem. Int. Ed. 2003, 42, 3996; 10.1002/anie.200200553 12973759

[36a] G. C. Morrison , R. O. Waite , J. Shavel , J. Org. Chem. 1967, 32, 2555; 10.1021/jo01286a096 5622462

[36b] G. C. Morrison , R. O. Waite , F. Serafin , J. Shavel , J. Org. Chem. 1967, 32, 2551.

[37] J. Bosch , J. Bonjoch , I. Serret , Heterocycles 1980, 14, 1983.

[38a] A. Kirschning , F. Hahn , Angew. Chem. Int. Ed. 2012, 51, 4012; 10.1002/anie.201107386 22441812

[38b] R. J. Goss , S. Shankar , A. A. Fayad , Nat. Prod. Rep. 2012, 29, 870. 22744619 10.1039/c2np00001f

[39] F. Feyen , F. Cachoux , J. Gertsch , M. Wartmann , K. H. Altmann , Acc. Chem. Res. 2008, 41, 21. 18159935 10.1021/ar700157x

[40] I. A. Simpson , D. Dwyer , D. Malide , K. H. Moley , A. Travis , S. J. Vannucci , Am. J. Physiol. Endocrinol. Metab. 2008, 295, E242. 10.1152/ajpendo.90388.2008 PMC2519757 18577699

[41] http://biosig.unimelb.edu.au/pkcsm/.

[42] https://preadmet.bmdrc.kr/adme-prediction/.

