# Supplementary material for: Synthesis of Indomorphan Pseudo‐Natural Product Inhibitors of Glucose Transporters GLUT‐1 and ‐3
Source: Angew Chem Int Ed Engl. 2019 Oct 7;58(47):17016–25. doi: 10.1002/anie.201909518 (PMC6900016; doi:10.1002/anie.201909518)
Supplement: Supplementary file 1 — Supplementary [file ANIE-58-17016-s001.pdf]

Angewandte Chemie
Eine Zeitschrift der Gesellschaft Deutscher Chemiker

Supporting Information

Synthesis of Indomorphan Pseudo-Natural Product Inhibitors of Glucose Transporters GLUT-1 and -3

*Javier Ceballos+, Melanie Schwalfenberg+, George Karageorgis, Elena S. Reckzeh, Sonja Sievers, Claude Ostermann, Axel Pahl, Magnus Sellstedt, Jessica Nowacki, Marjorie A. Carnero Corrales, Julian Wilke, Luca Laraia, Kirsten Tschapalda, Malte Metz, Dominik A. Sehr, Silke Brand, Konstanze Winklhofer, Petra Janning, Slava Ziegler, and Herbert Waldmann**

anie_201909518_sm_miscellaneous_information.pdf

Author Contributions

S.Z., H.W. designed research. J.C. and G.K. performed the chemical syntheses. M.S., E.S.R., G.K., M.S., J.N., M.R., J.W., L.L., K.T., D.A.S, and S.B. performed the biological research. G.K., C.O., and A.P. performed the chemoinformatic analyses. S.S. performed the high-throughput screening. J.C., M.S., E.S.R., G.K., M.S., J.N., M.R., J.W., L.L., K.T., M.M., D.A.S., K.W., P.J., S.Z., and H. W. analyzed the data. J.C., G.K., S.Z., and H.W. wrote the manuscript. All authors discussed the results and commented on the manuscript.

Table of contents:

Chemistry Part 6

General Information: 7

SAR analysis of the indomorphan class 9

General Procedures: 12

General Procedure A: Fischer Indole Synthesis. 12

General Procedure B: Indole *N*-alkylation (1). 12

General Procedure C: Indole *N*-alkylation (2). 12

General Procedure D: Cbz deprotection. 13

General Procedure E: Amide formation. 13

General Procedure F: Cbz deprotection and amide formation one-pot 13

General Procedure G: Cbz deprotection with HBr 14

General Procedure H: Carboxylic acid chlorination and amide formation *in situ* 14

General Procedure I: TBTU-mediated amide bond formation 14

General Procedure J: Synthesis of 2-Substituted nicotinic acid and amide bond formation 15

Synthesis of (±)-Glupin: 16

Synthesis of the Indomorphan class of compounds 23

R^1 23

R^2 35

R^3 56

R^4 65

Cheminformatic Analysis 86

Synthesis of the indomorphan (±)-Glupin fragments: 92

Separation of (±)-Glupin racemic mixture: 97

Determination of the absolute configuration of the enantiomers of the indomorphan class. 98

Determination of the activity of the separated enantiomers 26 and 26’ 107

NMR Spectra: 110

Chiral Chromatograms: 183

Indomorphan MS and UV absoprtion chromatograms 185

Biology Part 233

Supporting Figures S7-S13 234

Supporting Figure S7: Screening for modulators of glucose uptake 234

Supporting Figure S8: Influence of Glupin on mitochondrial respiration and lipid droplet formation. 236

Supporting Figure S9: Detection of GLUT-1 and GLUT-3 in DLD-1 and DLD-1 GLUT1(-/-) cells 237

Supporting Figure S10: Detection of GLUT-1-4 upon overexpression in CHO cells. 239

Supporting Figure S11. Influence of Glupin in GLUT-2 or GLUT-4 overexpression CHO cells. 240

Supporting Figure S12: Influence of Glupin on the expression of GLUT-1 and GLUT-3. 241

Supporting Figure S13: Influence of Glupin on the expression of GLUT-1 and GLUT-3. 242

Supporting Tables 244

Supporting Table S3: Influence of (±)-Glupin on metabolites in Molt16 cells. 244

Supporting Table S4: IC50 values for inhibition of glucose uptake in CHO and GLUT-1-4 overexpressing cell lines. 245

Supporting Table S5: IC50 values for inhibition of cell growth by Glupin in a panel of 94 cell lines. 246

Supporting Table S6: IC50 values for inhibition of cell growth by Glupin in a panel of 94 cell lines. 252

Experimental 257

Cell culture 257

Resazurin-based 2DG uptake assay (semi-automated high throughput) 257

Resazurin-based 2DG uptake assay (low throughput) 258

Hexokinase assay 259

^{3}H-2DG uptake assay 260

Seahorse measurements 260

Metabolite profiling 261

Cellular thermal shift assay (CETSA) 262

Transient overexpression of GLUT-1-4 in CHO cells and 2DG assay[1] 263

Real-time live-cell analysis 264

Detection of lipid droplets 264

RT-qPCR 265

Immunoblotting 265

Cell panel profiling 267

Statistics 268

Chemistry Part

General Information:

All reactions involving air- or moisture-sensitive reagents or intermediates were carried out in flame-dried glassware under an argon atmosphere. Dry solvents (THF, toluene, MeOH, DMF) were used as commercially available. Analytical thin-layer chromatography (TLC) was performed on Merck silica gel aluminum plates with F-254 indicator. Compounds were visualized by irradiation with UV light or potassium permanganate staining. Column chromatography was performed using silica gel Merck 60 (particle size 0.040-0.063 mm). ^{1}H-NMR and ^{13}C-NMR were recorded on a Bruker DRX400 (400 MHz), Bruker DRX500 (500 MHz) and INOVA500 (500 MHz) at 300 K using $CDCl_3$, MeOD or $(CD_3)_2SO$ as solvents. All resonances are reported relative to TMS. Spectra were calibrated relative to solvent's residual proton and carbon chemical shift: CDCl3 (δ=7.26 ppm for ^{1}H NMR and δ=77.16 ppm for ^{13}C NMR); (CD3)2SO: δ=2.50 ppm for ^{1}H NMR and δ=39.52 ppm for ^{13}C NMR); MeOD (δ=3.31 ppm for ^{1}H NMR and δ = 49.00ppm for^{13}C NMR). Multiplicities are indicated as: bs (broadened singlet), s (singlet), d (doublet), t (triplet), q (quartet), quin (quintet), m (multiplet); and coupling constants (J) are given in Hertz (Hz). In the NMR spectra where rotameric mixtures are present, the proton signals that split are given as fractions (e.g. 0.5H) so as to easily differentiate which signals split due to the mixture and which don´t. High resolution mass spectra were recorded on a LTQ Orbitrap mass spectrometer coupled to an Acceka HPLC-System (HPLC column: Hypersyl GOLD, 50 mm × 1 mm, particle size 1.9 µm, ionization method: electron spray ionization). Preparative HPLC separations were carried out using a reversed-phase C18 column (RP C18, flow 20.0 mL/min, solvent A: 0.1% TFA in water, solvent B: 0.1% TFA in Acetonitrile). All other chemicals and solvents were purchased from Sigma-Aldrich, Fluka, TCI, Acros Organics, ABCR and AlfaAesar. Unless otherwise noted, all commercially available compounds were used as received without further purifications. Compounds with 2-substituted nicotinic acid can give rise to very complicated spectra. The lack of impurities/isomers was checked by the NMR spectra of the starting material of the final reaction, the free amine (compound **12**). At that point, we can

see that only one isomer is present and with very high purity. However, the formation of the amide bond and 2-substitution of the nicotinic acid can give rise to rotamers and atropoisomers, respectively. This can be seen in the NMR spectra of the final products. In some cases, due to very complicated spectra, only 1H are given. This is nicely noticeable when we look at the 78 ppm area of the ^{13}C spectra, where 4 signals can be seen for the same carbon (instead of the normal 1 signal per carbon). Purity was checked by uHPLC and in all cases over 95% before submitting the compounds for biological activity measurement. Also, when describing the 1H Spectra, data is given in the most clear way possible. Some signals are given as fractions of protons and some apparent coupling constants are given in cases were clearly the signal splitting is due to rotamers/atropoisomers and not because of actual coupling between 1H nuclei (i.e. values over 30 Hz). Furthermore, when easily determined the ratio of the rotamers mixture was given, but for simplification the integrals of the NMR spectra was given 0.5H/0.5H (instead of e.g. 0.46H/0.54H).

SAR analysis of the indomorphan class

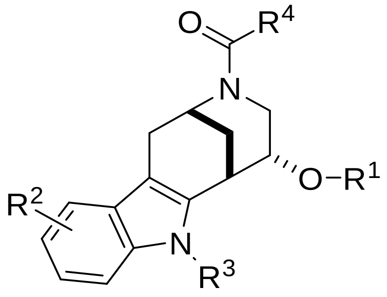

Supporting Table S1: Complete SAR of the Indomorphan compound class.

Entry	Compound	R1[a]	R2[a]	R3[a]	R4[a]	IC50 (μM)[b]
1	(±)-Glupin	Me	H	CH_2CO_2Et	2-(methylthio)pyridin-3-yl	0.055 ± 0.017
2	5b	Ac	H	CH_2CO_2Et	2-(methylthio)pyridin-3-yl	0.053 ± 0.023
3	5c	H	H	CH_2CO_2Et	2-(methylthio)pyridin-3-yl	0.13 ±0.05
4	5d	^{n}Pr	H	CH_2CO_2Et	2-(methylthio)pyridin-3-yl	0.087 ± 0.015
5	5e	PEG*[c]	H	CH_2CO_2Et	2-(methylthio)pyridin-3-yl	0.083 ± 0.026
6	5z	Allyl	H	CH_2CO_2Et	2-(methylthio)pyridin-3-yl	0.12 ± 0.02
7	5f	Me	5-OH	CH_2CO_2Et	2-(methylthio)pyridin-3-yl	0.10 ± 0.06
8	5g	Me	5-Cl	CH_2CO_2Et	2-(methylthio)pyridin-3-yl	1.4 ± 0.2
9	5h	Me	7-Cl	CH_2CO_2Et	2-(methylthio)pyridin-3-yl	0.21 ± 0.09
10	5i	Me	5-CO_2Et	CH_2CO_2Et	2-(methylthio)pyridin-3-yl	>30
11	5aa	Me	5-OMe	CH_2CO_2Et	2-(methylthio)pyridin-3-yl	4.22 ± 3.46
12	5ab	Me	5-COmorpholine	CH_2CO_2Et	2-(methylthio)pyridin-3-yl	>30
13	(-)-5ac	Me	5-Br	CH_2CO_2Et	2-(methylthio)pyridin-3-yl	4.7 ± 0.8
14	(+)-5ac	Me	5-Br	CH_2CO_2Et	2-(methylthio)pyridin-3-yl	>30
15	5j	Me	H	H	2-(methylthio)pyridin-3-yl	>30
16	5k	Me	H	$CH_2CO_2{}^tBu$	2-(methylthio)pyridin-3-yl	0.19 ± 0.06
17	5l	Me	H	$CH_2CO_2{}^nPr$	2-(methylthio)pyridin-3-yl	0.093 ± 0.035
18	5m[d]	Me	H	CH_2CONHMe	2-(methylthio)pyridin-3-yl	1.45 ± 0.6
19	5n	Me	H	CH_2CO_2H	2-(methylthio)pyridin-3-yl	10.0 ± 4.0
20	5o	Me	H	furan-2-ymethyl	2-(methylthio)pyridin-3-yl	6.7 ± 2.4
21	5p	Me	H	CO_2Et	2-(methylthio)pyridin-3-yl	28 ± 2
22	5q	Me	H	$(CH_2)_2CO_2Et$	2-(methylthio)pyridin-3-yl	8.4 ± 3.6
23	5ad	Me	H	CH_2CO_2Me	2-(methylthio)pyridin-3-yl	0.089 ± 0.032
24	5ae	Me	H	CH_2CO_2Ph	2-(methylthio)pyridin-3-yl	0.22± 0.08

Entry	Compound	R1[a]	R2[a]	R3[a]	R4[a]	IC50 (μM)[b]
25	5r[d]	Me	H	CH_2CO_2Et	*N*-morpholino	3.16+ ± 0.6
26	5s[d]	Me	H	CH_2CO_2Et	2-furyl	3.4 ± 0.3
27	5t	Me	H	CH_2CO_2Et	pyridine-3-yl	2.3 ± 0.8
28	5u	Me	H	CH_2CO_2Et	2-fluoropyridin-3-yl	0.54 ± 0.16
29	5v	Me	H	CH_2CO_2Et	2-methoxypyridin-3-yl	0.23 ± 0.05
30	5w	Me	H	CH_2CO_2Et	2-trifluoromethylpyridin-3-yl	0.12 ± 0.03
31	5x	Me	H	CH_2CO_2Et	2-(methylthio)-4-(trifluoromethyl)pyridin-3-yl	6.7 ± 1.6
32	5y	Me	H	CH_2CO_2Et	2-(methylthio)-5-(trifluoromethyl)pyridin-3-yl	14 ± 4
33	5af	Me	H	CH_2CO_2Et	2-chloropyridin-3-yl	0.093 ± 0.041
34	5ag	Me	H	CH_2CO_2Et	2-(methylsulfonyl)pyridin-3-yl	1.7 ± 0.7
35	5ah	Me	H	CH_2CO_2Et	2-(phenylthio)pyridin-3-yl	0.14 ± 0.07
36	5ai	Me	H	CH_2CO_2Et	2-(^{n}propylthio)pyridin-3-yl	0.19 ± 0.12
37	5aj	Me	H	CH_2CO_2Et	2-bromopyridin-3-yl	0.18 ± 0.06
38	5ak	Me	H	CH_2CO_2Et	2-ethoxypyridin-3-yl	0.22 ± 0.07
39	5al	Me	H	CH_2CO_2Et	2-morpholinopyridin-3-yl	1.4 ± 0.2
40	5am	Me	H	CH_2CO_2Et	2-pivalamidopyridin-3-yl	>30
41	5an	Me	H	CH_2CO_2Et	pyridine-2-yl	24 ± 3
42	5ao	Me	H	CH_2CO_2Et	pyridine-4-yl	13 ± 3
43	5ap	Me	H	CH_2CO_2Et	pyrimidin-5-yl	14 ± 5
44	5aq	Me	H	CH_2CO_2Et	thiophen-2-yl	3.0 ± 0.4
45	5ar	Me	H	CH_2CO_2Et	6-methyl-2-(methylthio)pyridin-3-yl	6.7 ± 1.6
46	5as	Me	H	CH_2CO_2Et	5-bromo-2-(methylthio)pyridin-3-yl	14 ± 4
47	5at	Me	H	CH_2CO_2Et	2-(5-methylfuyl)	8.6 ± 4.0
48	5au[d]	Me	H	$CH_2CONHMe$	1-(thiophen-2-yl)methan-1-yl	16 ± 1
49	5av[d]	Me	H	$CH_2CONHMe$	pyrazin-2-yl	>30
50	5aw[d]	Me	H	CH_2CO_2Et	pyrazin-2-yl	2.0 ± 0.4
51	5ax[d]	Me	H	CH_2CONH_2	pyrazin-2-yl	>30
52	5ay[d]	Me	H	$CH_2CONHMe$	*N*-morpholino	>30
53	5az[d]	Me	H	$CH_2CONHMe$	3-(trifluoromethyl)anilinyl	>30
54	5ba[d]	Me	H	H	(No carbonyl) 1-benzoylpiperidin-4-yl	>30
55	5bb[d]	Me	H	H	3-(trifluoromethoxy)phenyl	>30
56	5bc[d]	Me	H	H	(No carbonyl) pyridine-2-yl	>30

Entry	Compound	R1[a]	R2[a]	R3[a]	R4[a]	IC50 (μM)[b]
57	5bd[d]	pyridine -2-yl	H	H	3-cyanophenyl	>30
58	5be[d]	pyridine -2-yl	H	H	cyclopentanamine	>30
59	5bf[d]	pyridine -2-yl	H	H	aniline	>30
60	5bg[d]	Me	H	$CH_2CONHMe$	(No carbonyl) tetrahydro-2H-pyran-4-yl	>30
61	5bh[d]	Me	H	$CH_2CONHMe$	ethylamine	>30
62	5bi[d]	pyridine -2-yl	H	H	2-phenylethan-1-yl	>30
63	5bj[d]	pyridine -2-yl	H	H	phenylmethanamine	>30

[a] The cells highlighted indicate where the modification was introduced compared to (±)-Glupin (entry 1). [b] IC_{50} values determined for the inhibition of 2-DG uptake in HCT116 cells using the semi-automated hight-throughput assay. Data are mean values (more than three independent experiments). Data are mean values ± s.d. [c] PEG*: (2,2-dimethyl-4-oxo-3,9,12,15-tetraoxa-5-azaoctadecan-18-yl)carbamic acid (see section: Synthesis of the Indomorphan class of compounds, compd.: **5e**). [d] Compound obtained commercially from EDELRIS.

General Procedures:

General Procedure A: Fischer Indole Synthesis.

The corresponding ketone (1.0 equiv.) was dissolved in acetic acid (0.15M), the corresponding hydrazine hydrochloride (1.0 equiv.) was added and the solution was stirred and refluxed for 1.5h. The reaction was quenched with sodium bicarbonate (saturated aqueous solution), the mixture was extracted with CH_2Cl_2, dried over $MgSO_4$, filtered and the solvents were concentrated *in vacuo*. The crude product was purified by flash chromatography (5 to 50% E.A./CH_2Cl_2) to afford the corresponding indole as the pure *anti* diasteroisomer as an orange solid.

General Procedure B: Indole *N*-alkylation (1).

The corresponding indole (1.0 equiv.), cesium carbonate (3.0 equiv.) and a 2-bromoacetate compound (3.0 equiv) were dissolved in DMF (0.17M) and allowed to stir at room temperature for 2h. The reaction was then partitioned between CH_2Cl_2 and BRINE/H_2O (1:1). The aqueous phase was extracted with CH_2Cl_2 (5X), dried over $MgSO_4$, filtered and the solvents were concentrated *in vacuo*. The crude product was purified by column chromatography (1 to 5% E.A./CH_2Cl_2) to afford the corresponding alkylated indole product as an orange oil.

General Procedure C: Indole *N*-alkylation (2).

The corresponding alkyl bromoacetate (3.2 equiv.) was added to a stirred suspension of the indole (1 equiv.) and Cs_2CO_3 (3.2 equiv.) in DMF (0.5 M) and the reaction mixture was stirred at rt. After 24 h, the reaction mixture was diluted with EtOAc (20 mL) and washed sequentially with H_2O (20 mL), sat. brine (2 × 20 mL) and sat. LiCl (2 × 10 mL). The organic layer was dried over $MgSO_4$, filtered and conc. *in vacuo* to give a crude mixture.

General Procedure D: Cbz deprotection.

The corresponding Cbz *N*-protected compound (1.0 equiv.) was dissolved in ethanol (0.15M), Pd/C (50mg/mmol) was suspended in the reaction and the atmosphere was changed to hydrogen gas (1atm). After 5h the mixture was filtered through Celite, solvents were concentrated *in vacuo* and the crude product was purified by column chromatography (5% MeOH/CH_2Cl_2) to afford the *N*-deprotected product typically as a white solid.

General Procedure E: Amide formation.

The corresponding amine (1.0 equiv.), triethylamine (1.2 equiv.) and the corresponding aroyl chloride (1.0 equiv.) were dissolved in CH_2Cl_2 (0.2M) and the reaction was allowed to stir overnight at room temperature. The mixture was washed with water and BRINE, solvents were concentrated *in vacuo* and the crude product was purified by chromatography by preparative HPLC (10% to 50% ACN/H_2O with 0.1% TFA), unless otherwise stated, to afford the indomorphan class analogue.

General Procedure F: Cbz deprotection and amide formation one-pot

Pd on activated carbon (10% w/w), was added to a stirred solution of the carbamate (1 equiv.) and ammonium formate (5 equiv.) in EtOH (0.1 M) and the reaction mixture was heated to reflux. After 2 h, the reaction mixture was allowed to cool to rt, and filtered in vacuo through a celite pad, eluting with CH_2Cl_2 (4 × reaction volume) and the filtrate concentrated *in vacuo*. The crude was rediluted in CH_2Cl_2 (0.15 M) and Et_3N (1.6 equiv.) and the corresponding acyl chloride (1.5 equiv.) were added and the reaction mixture was stirred at rt. After 24 h the reaction mixture was conc. *in vacuo* to give a crude. The

crude product was purified by preparative HPLC (10% to 50% ACN/H_2O with 0.1% TFA), unless otherwise stated**.**

General Procedure G: Cbz deprotection with HBr

To a solution of the corresponding Cbz-protected compound (1 equiv)l) in acetic acid (0.3 M), HBr in acetic acid 33% solution (0.98 equiv.) was added and the reaction was allowed to stir for 30 min. Then water was added and the aqueous phase was extracted with CH_2Cl_2 (5X). Evaporation of the solvents under reduced pressure afforded the corresponding HBr-amine salt as an orange sticky foamy solid.

General Procedure H: Carboxylic acid chlorination and amide formation *in situ*

To a solution of nicotinic acid (1.2 equiv.) (13.5 mg, 0.110 mmol) in toluene (0.067 M) thionyl chloride (1.2 equiv) freshly distilled and a drop of DMF were added. The solution was stirred at reflux for 1h, then solvents were evaporated and the crude was redissolved in CH_2Cl_2 (0.55M). The solution was canulated to another solution of compound **12a** (1.0 equiv), triethylamine (1.2 equiv.) in CH_2Cl_2 (0.37 M). The mixture was allowed to stir at room temperature overnight. Then the organic phase was washed with water and BRINE, concentrated *in vacuo* to afford the crude product.

General Procedure I: TBTU-mediated amide bond formation

Compound **12a** (1.0 equiv.), TBTU (CAS: 125700-67-6) (1.6 equiv.) and the corresponding carboxylic acid (1.5 equiv.) and DIPEA (2.5 equiv.) were dissolved in DMF (0.06 M), and allowed to stir for 3 hours. The solvents were concentrated *in vacuo* to afford the crude product.

General Procedure J: Synthesis of 2-Substituted nicotinic acid and amide bond formation

2-chloronicotinic acid (1.0 equiv.) and sodium thiometoxide (2.5 equiv.) were suspended in dioxane (0.4 M) and water (1.0 M) in a sealed tube. The reaction was stir overnight at 120°. The reaction was then acidify with citric acid (20% aqueous solution) to pH 3-4, extracted with ethyl acetate and concentrated *in vacuo* to afford the crude product.

Synthesis of (±)-Glupin:

Compound **9** (Scheme S1), was synthesised following the reported procedure from Bonjoch et al.[1]

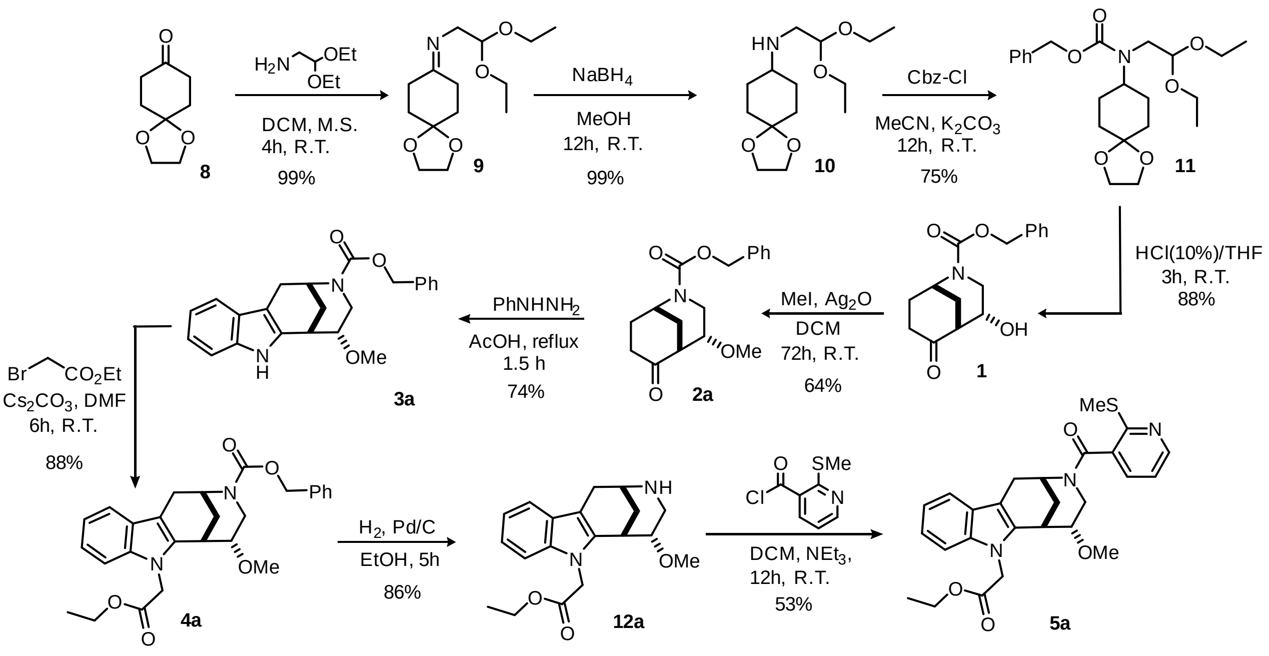

Supporting Scheme S1: Synthetic pathway towards (+/-)-Glupin.

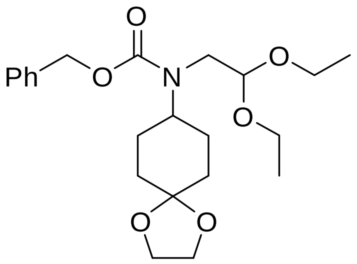

Benzyl (2,2-diethoxyethyl)(1,4-dioxaspiro[4.5]decan-8-yl)carbamate (11):

To a room temperature solution of the crude amine **10** (10g, 36.58mmol) in MeCN (0,155M), K2CO3 (10.11g, 73.16mmol) and benzyl chloroformate (10.44mL, 73.16mmol) were added. The mixture was allowed to stir at room temperature for 12h. Then the solvent was evaporated and the oil was partitioned between BRINE and CH_2Cl_2. The aqueous phase was extracted 2 times with CH2Cl2 and the organic

phases were combined. Solvents were removed to afford the crude product that was further purified by chromatography (2 to 30% E.A./CH_2Cl_2) to yield compound 4 as a colourless oil (11.18g, 75%).

^{1}H NMR (CDCl3, 400MHz) (rotameric mixture 1:1): δ 1.12 (m, 6H), 1.59-1.78 (m, 8H), 1.88 (m, 2H), 3.25-3.73 (m, 7H), 3.92 (s, 4H), 4.53 (bs, 0.5H), 4.70 (bs, 0.5H), 5.14 (s, 2H), 7.28-7.38 (m, 5H).

HRMS (ESI)**:** calc. for $[M+H]^+$ $C_{22}H_{34}NO_6$: 408.23806, found. 408.23844.

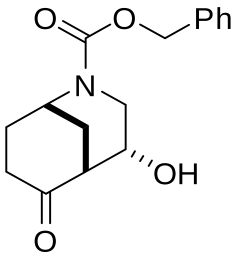

(±)-Benzyl-4-hydroxy-6-oxo-2-azabicyclo[3.3.1]nonane-2-carboxylate (1):

Carbamate **11** (11.18g, 27.43mmol) was dissolved in a mixture of THF (180mL, 0.15M) and 10% aqueous HCl (365mL, 0.075M) and stirred at room temperature for 3h. The mixture was extracted with CH_2Cl_2 and the solvents removed. Crude product **5** (6.98g, 88%) contains around 15% of the *syn* diasteroisomer but is pure enough to continue the synthesis.*Syn* and *anti* diasteroisomers coelute and cannot be separated by chromatography.

^{1}H NMR (CDCl3, 400MHz) (NMR Spectra shows a 1:1.5 mixture of rotamers plus a some *syn* diasteroisomer, data given for the mayor diasteroisomer)**:** 1.91-2.24 (m, 4H), 2.40-2.61 (m, 2H), 2.79 (bs, 1H), 2.88 (t, *J*= 12.3, 1H), 3.01-3.04 (m, 1H), 3.92-4.02 (m, 1H), 4.29 (dd, *J* =13.3, 6.1 Hz, 0.6H), 4.37 (dd, *J*=13.5, 6.1 Hz, 0.4H), 4.52 (bs, 0.4H), 4.63(bs, 0.6H), 5.15 (s, 2H), 7.35 (m, 5H).

^{13}C NMR (CDCl3, 100MHz): δ 27.7, 28.7, 29.5, 30.0, 36.9, 38.4, 38.5, 43.7, 44.0, 46.1, 46.3, 49.4, 49.6, 67.6, 67.9, 128.0, 128.1, 128.2, 128.3, 128.7, 136.5, 136.6, 155.5, 212.5, 213.4

HRMS (ESI)**:** calc. for $[M+H]^+$ $C_{16}H_{20}NO_4$:290.13868, found:290.13906

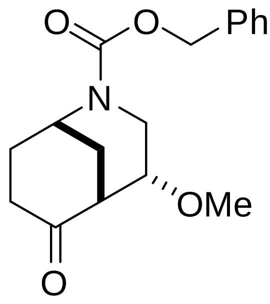

(±)-Benzyl 4-methoxy-6-oxo-2-azabicyclo[3.3.1]nonane-2-carboxylate (2a):

Ketone **5** (3.0g, 10.37 mmol) was dissolved in CH_2Cl_2 (0.145M) and protected from light. Silver oxide (12.01g, 51.84 mmol) and methyl iodide (3.23mL, 51.85 mmol) were added and the reaction was allowed to stir at room temperature for 48h. The reaction was filtered through Celite and solvents were evaporated. The crude was purified by chromatography (8 to 50% E.A./CH_2Cl_2) to obtain **6** (isolated with 10% of the *syn* diasteroisomer) (2.01g, 64%) as a colorless oil and starting material **5** (0.690g, 23% recovery).*Syn* and *anti* diasteroisomers coelute and cannot be separated by chromatography.

^{1}H NMR (CDCl3, 700MHz) (NMR Spectra shows a 1:1mixture of rotamers plus the *syn* diasteroisomer impurity, data given for the mayor diasteroisomer): δ 1.92-2.05 (m, 3H), 2.13-2.23 (m, 1H), 2.35-2.58 (m, 2H), 3.07-3.20 (m, 2H), 3.30-3.39 (m, 3H), 3.60 (m,1H), 4.30 (dd, *J*=13.6, 7.1 Hz, 0.5H), 4.39-4.42 (m,1H), 4.51 (bs, 0.5H), 5.13-5.18 (m, 2H), 7.32-7.36 (m, 5H).

^{13}C NMR (CDCl3, 100MHz): δ29.8, 30.4, 30.5, 30.8, 39.0, 44.3, 44.4, 44.9, 46.9, 47.3, 56.6, 56.7, 67.5, 67.6, 75.4, 75.6, 128.0, 128.1, 128.2 128,6 136.4, 136.5, 155.4, 155.5, 209.2, 209.3.

HRMS (ESI)**:** calc. for $[M+H]^+$ $C_{17}H_{22}NO_4$: 304.15433, found. 304.15450.

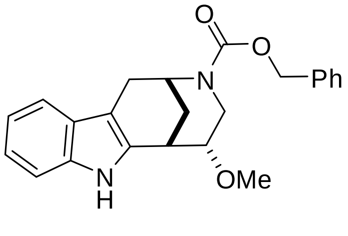

(±)-Benzyl 5-methoxy-1,2,4,5,6,7-hexahydro-3H-2,6-methanoazocino[5,4-b]indole-3-carboxylate (3a):

Ketone **6** (2.0g, 6.59mmol) was dissolved in acetic acid (44.0mL, 0.15M). Phenyl hydrazine hydrochloride (0.953g, 6.59mmol) was added and the solution was stirred and refluxed for 1.5h. The reaction was quenched with sodium bicarbonate (saturated aqueous solution), the mixture was extracted with CH_2Cl_2, solvents were evaporated and the crude was purified by chromatography (3 to 15% E.A./CH_2Cl_2) to yield compound**7**(as the pure *anti* diasteroisomer) (1.74g, 74%) as an orange solid.

^{1}H NMR (CDCl3, 400MHz): δ 2.02-2.11 (m, 2H), 2.54 (dt, *J*=23.5, 11.7 Hz, 1H), 2.81 (t, *J*=18.0 Hz, 1H), 3.11 (ddd, *J*=16.8, 7.4, 5.8 Hz, 1H), 3.34 (m, 1H), 3.40 (s, 3H), 3.55 (td, *J*=10.3, 5.7 Hz, 1H), 4.13 (dd, *J*=12.5, 5.1 Hz, 0.5H), 4.29 (dd, *J*=12.7, 5.3 Hz, 0.5H), 4.79 (d, *J*=28.4 Hz, 1H), 5.18 (d, *J*=19.3 Hz, 2H), 7.09 (t, *J*=7.4 Hz, 1H), 7.15 (td, *J*=6.8, 1.2 Hz, 1H), 7.31-7.58 (m, 7H), 7.95 (d, *J*=12.6 Hz, 1H).

^{13}C NMR (CDCl3, 100MHz): δ 27.8, 28.2, 30.1, 30.5, 32.5, 32.9, 42.3, 42.4, 45.4, 56.9, 67.3, 67.5, 77.7, 78.0, 109.1, 109.4, 111.0, 118.0, 118.1, 119.4, 121.6, 126.95, 127.00, 127.97, 128.03, 128.1, 128.2, 128.6, 128.7, 132.7, 132.8, 136.1, 136.9, 137.0, 155.7.

HRMS (ESI)**:** calc. for $[M+H]^+$ $C_{23}H_{25}N_2O_3$: 377.18597, found. 377.18728.

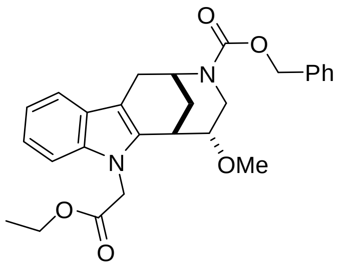

(±)-Benzyl 7-(2-ethoxy-2-oxoethyl)-5-methoxy-1,2,4,5,6,7-hexahydro-3H-2,6-methanoazocino[5,4-b]indole-3-carboxylate (4a):

Compound **7** (1.5g, 3.98mmol), cesium carbonate (3.89g, 11.94mmol) and ethyl 2-bromoacetate (1.32mL, 11.94mmol)were dissolved in DMF (23.4mL, 0.17M) and allowed to stir at room temperature

for 2h. The reaction was then partitioned between CH_2Cl_2 and BRINE/H_2O (1:1). The aqueous phase was extracted with CH_2Cl_2 (5X), solvents were removed and the crude was purified by chromatography (1to 5% E.A./CH_2Cl_2) to yield an orange oil as product **8** (1.62g, 88%).

^{1}H NMR (CDCl3, 400MHz) (with small impurity of the ethyl 2-bromoacetate):δ 1.24 (t, *J*=7.12 Hz, 3H), 1.97-2.07 (m, 2H), 2.57 (t, *J*=11.8 Hz, 0.5H),2.64 (t, *J*=11.6 Hz, 0.5H), 2.80 (d, *J*=16.8, 0.5H), 2.84 (d, *J*=16.9 Hz, 0.5H), 3.16 (td, *J*=5.5, 16.1 Hz, 1H), 3.34 (d, *J*=9.2 Hz, 3H), 3.40 (s, 1H), 3.54-3.59 (m, 1H), 4.13-4.33 (m, 3H), 4.77-4.84 (m, 2H), 5.08-5.16(m, 1H), 5.17 (s, 1H), 5.21 (s, 1H), 7.10 (s, 1H), 7.18-7.21 (m, 2H), 7.31-7.48 (m, 6H).

^{13}C NMR (CDCl3, 100MHz): δ14.3, 27.9, 28.2, 30.6, 30.9, 31.7, 31.8, 41.6, 41.7, 45.0, 45.2, 56.8, 56.9, 61.2, 61.4, 67.3, 67.5, 68.3, 78.3, 78.7, 108.9, 109.5, 109.8, 118.1, 118.2, 119.4, 121.6, 126.6, 126.7, 127.9, 128.0, 128.1, 128.2, 128.6, 128.7, 134.0, 134.1, 136.9, 137.1, 137.2, 155.6, 169.5, 169.6, 169.8

HRMS (ESI)**:** calc. for $[M+H]^+$ $C_{27}H_{31}N_2O_5$: 463.22275, found. 463.22269.

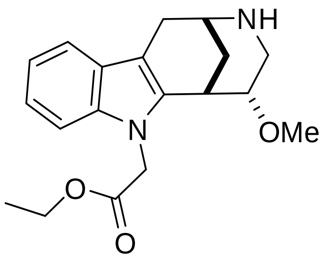

(±)-Ethyl 2-(5-methoxy-1,2,3,4,5,6-hexahydro-7H-2,6-methanoazocino[5,4-b]indol-7-yl)acetate (12a):

Compound **8** (1.0g, 2.16mmol) was dissolved in Ethanol (14.4mL, 0.15M), Pd/C (0.108mg, 50mg/mmol) was suspended in the reaction and the atmosphere was changed to hydrogen gas (1atm). After 5h the reaction was filtered through Celite, solvents were evaporated and submitted to chromatography (5% MeOH/CH_2Cl_2) to yield compound **9** as a white solid (0.610g, 86%).

^{1}H NMR (CDCl3, 600MHz): δ 1.25 (t, *J*=7.13 Hz, 3H), 2.00 (ddd, *J*=2.3, 3.8, 12.6 Hz, 1H), 2.16 (dt, *J*=3.0, 12.6 Hz, 1H), 2.55 (t, *J*=11.1 Hz, 1H), 2.81 (d, *J*=17.0 Hz, 1H), 3.06 (dd, *J*=4.5, 11.6 Hz, 1H),

3.19 (dd, *J*=6.0, 16.9 Hz, 1H), 3.30 (s, 3H), 3.39 (m, 1H), 3.53-3.56 (m, 2H), 3.60 (dt, *J*=4.4, 10.4 Hz, 1H), 4.12-4.24 (m, 2H), 4.79 (d, *J*=18.0 Hz, 1H), 5.11 (d, *J*=18.0 Hz, 1H), 7.1 (ddd, *J*=1.2, 6.6, 7.8 Hz, 1H), 7.18 (td, *J*=1.2, 6.6 Hz, 1H), 7.20 (d, *J*=8.0 Hz, 1H), 7.49 (d, *J*=7.8 Hz, 1H).

^{13}C NMR (CDCl3, 100MHz): δ 14.2, 29.1, 32.1, 32.2, 42.2, 44.8, 45.6, 56.5, 61.2, 76.9, 77.2, 77.4, 78.9, 108.8, 110.4, 118.1, 119.2, 121.3, 126.5, 135.1, 137.0, 169.5.

NOESY: To confirm the *anti* configuration between the methylene bridge and the methoxy group a NOESY experiment was done. NOE interactions are shown below (for simplicity only one enantiomer has been drawn).

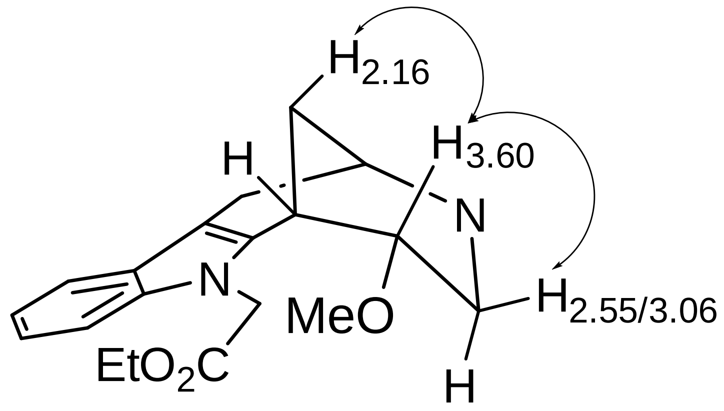

Supporting Figure S1: Proton coupling detected by NOESY experiment.

HRMS (ESI)**:** calc. for $[M+H]^+$ $C_{19}H_{25}N_2O_3$: 329.18597, found. 329.18658.

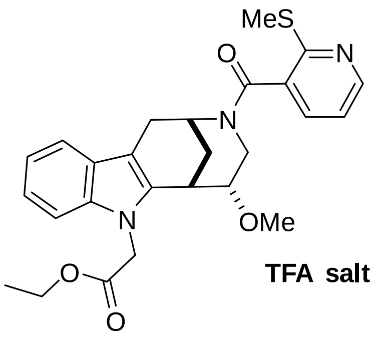

(±)-Glupin-1: Ethyl 2-(5-methoxy-3-(2-(methylthio)nicotinoyl)-1,2,3,4,5,6-hexahydro-7H-2,6-methanoazocino[5,4-b]indol-7-yl)acetate (5a):

Compound **9** (50.0 mg, 0.152 mmol), Triethylamine (0.0254mL, 0.182mmol) and 2-(methylthio)nicotinoyl chloride (28.5mg, 0.152mmol) were dissolved in CH_2Cl_2 (0.8mL, 0.2M) and the reaction was allowed to stir overnight at room temperature. The reaction was washed with water and BRINE, solvents were evaporated and the crude product was purified by chromatography (1.5% MeOH/CH_2Cl_2) and by preparative HPLC (10% to 50% ACN/H_2O with 0.1% TFA) to yield compound **10** (38.6mg, 53%) as the TFA salt.

^{1}H NMR (CDCl3, 500MHz): δ 1.22-1.27 (m, 3H), 1.94-2.33 (m, 2H), 2.60-2.66 (m, 4H),2.83-3.10 (m, 1.5H),3.18 (s, 1.5H), 3.30-3.36 (m, 1H), 3.42 (s, 1.5H), 3.48 (s, 1H), 3.69-3.84 (m, 1H), 4.01 (bs,0.5H), 4.10-4.26 (m, 2H), 4.79 (dd, *J*=18.0, 14.4 Hz, 1H), 4.89 (dd, *J*=4.9, 12.9 Hz, 0.5H), 5.03 (d, *J*=18.0 Hz, 0.5H), 5.15 (d, *J*=18.0 Hz, 0.5H), 5.44 (bs, 0.5H), 7.06-7.23 (m, 4H), 7.44-7.52 (m, 2H), 7.73 (bs, 1H), 8.51 (d, *J*=3.8 Hz, 0.5H), 8.57 (d, *J*=3.8 Hz, 0.5H).

^{13}C NMR (CDCl3, 100MHz): δ 13.3, 13.5, 14.3, 31.5, 31.9, 32.3, 43.3, 45.0, 45.1, 49.4, 57.1, 61.5, 78.1, 79.2, 109.0, 109.1, 118.2, 118.3, 119.4, 119.6, 121.9, 126.6, 133.8, 133.9, 134.1, 137.2, 137.3, 149.5, 149.6, 167.4, 169.4, 169.5.

HRMS (ESI)**:** calc. for $[M+H]^+$ $C_{26}H_{30}N_3O_4S$: 480.19515, found. 480.19559. Af

Optical rotation: (not as the TFA salt): (+)-Glupin-1 $[\alpha]_D$= + 10.7° ± 3.4; (-)-Glupin-1 $[\alpha]_D$= - 10.0° ± 1.0

Synthesis of the Indomorphan class of compounds

R[1]

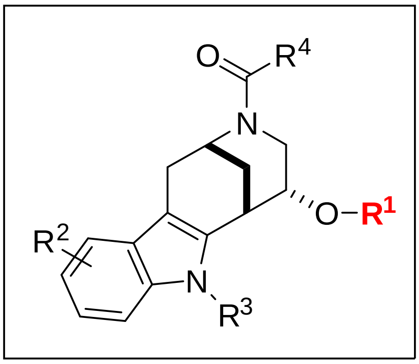

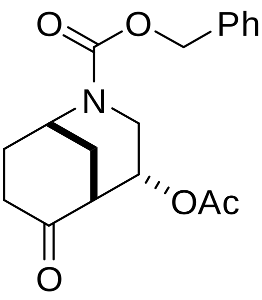

(±)-Benzyl 4-acetoxy-6-oxo-2-azabicyclo[3.3.1]nonane-2-carboxylate (2b):

Compound **1** (2.0 g, 6.91 mmol) was dissolved in pyridine (40 mL, 0.175 M). To this solution a solution of acetyl chloride (1.5 mL, 20.73 mmol) in CH_2Cl_2 (40 mL, 0.175 M). was added dropwise at -10°, the reaction was stirred at this temperature for 3 hours. The reaction was then washed with a saturated solution of copper sulfate (8X) to remove the pyridine. The solvents were coevaporated with toluene and the crude was then purified by chromatography (5 to 20% E.A./CH_2Cl_2) to yield product **2b** (with ~15% of the *syn* diasteroisomer) (1.6g, 69%) as a light brown oil.

^{1}H NMR (CDCl3, 400MHz) (NMR Spectra shows a 1:1 mixture of rotamers, data given for the mayor diasteroisomer): δ 1.97-2.06 (m, 6H), 2.16-2.28 (m, 1H), 2.36-2.54 (m, 1H), 2.59-2.65 (m, 1H), 3.00 (s,

1H), 3.23 (t, *J*=12.0 Hz, 1H), 4.27-4.36 (m, 1H), 4.45 (s, 0.44H), 4.55 (s, 0.56H), 5.06-5.21 (m, 3H), 7.36 (m, 5H).

^{13}C NMR (CDCl3, 100MHz): δ 21.0, 29.6, 30.4, 30.5, 30.7, 37.0, 38.9, 43.8, 43.9, 44.2, 44.4, 47.1, 47.2, 67.7, 68.0, 128.1, 128.2, 128.4, 128.6, 128.7, 136.3, 155.4, 169.9, 208.4, 208.6.

HRMS (ESI)**:** calc. for $[M+H]^+$ $C_{18}H_{22}NO_5$: 332.14925, found. 332.14977.

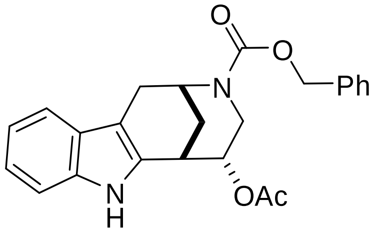

(±)-Benzyl 5-acetoxy-1,2,4,5,6,7-hexahydro-3H-2,6-methanoazocino[5,4-b]indole-3-carboxylate (3b):

Compound **3b** (76% yield, light orange foamy solid) was synthesized from **2b** following General Procedure **A**, the crude product can be further used to continue the synthesis. For analytical purposes some product was purified by chromatography (3 to 15% E.A./CH_2Cl_2) to yield an orange foamy solid.

^{1}H NMR (CDCl3, 400MHz) (rotameric mixture 1:1.5)**:** δ 2.07-2.16 (m, 5H), 2.67-2.87 (m, 2H), 3.09-3.17 (m, 1H), 3.38 (m, 1H), 4.08-4.21 (m, 1H), 4.78 (s, 0.4H), 4.86 (m, 0.6H), 5.05-5.12 (m, 1H), 5.14-5.21(m, 2H), 7.12 (t, *J*=7.4 Hz, 1H), 7.17-7.21 (m, 1H), 7.29-7.41 (m, 6H), 7.49 (t, *J*=8.4 Hz, 1H), 7.86 (s, 1H).

HRMS (ESI)**:** calc. for $[M+H]^+$ $C_{24}H_{25}N_2O_4$: 405.18088, found. 405.18110.

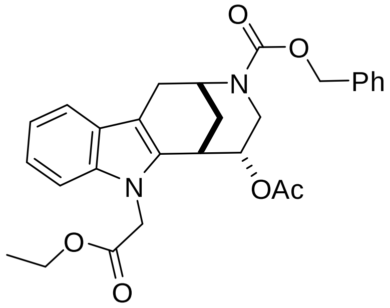

(±)-Benzyl 5-acetoxy-7-(2-ethoxy-2-oxoethyl)-1,2,4,5,6,7-hexahydro-3H-2,6-methanoazocino[5,4-b]indole-3-carboxylate (4b):

Compound **4b** (85% yield, light orange oil) was synthesised from **3b** following General Procedure **B**.

^{1}H NMR (CDCl3, 500MHz): δ 1.25 (t, *J*=7.1 Hz, 3H), 2.03-2.15 (m, 5H), 2.68-2.89 (m, 2H), 3.09-3.25 (m, 1H), 3.34-3.42 (m, 1H), 4.10-4.25 (m, 3H), 4.79-4.89 (m, 3H), 5.08-5.22 (m, 3H), 7.11-7.15 (m, 1H), 7.20-7.22 (m, 2H), 7.30-7.51 (m, 6H).

^{13}C NMR (CDCl3, 100MHz): δ 14.3, 21.2, 27.8, 28.2, 29.4, 29.8, 30.6, 30.6. 30.9, 31.1, 41.1, 44.8, 44.9, 61.8, 67.5, 67.7, 71.4, 71.6, 109.0, 110.5, 110.7, 118.6, 119.9, 122.1, 126.8, 127.8, 128.0, 128.1, 128.3, 132.9, 136.7, 137.3, 155.5, 168.8, 170.0

HRMS (ESI)**:** calc. for [M+H]+ $C_{28}H_{31}N_2O_6$: 491.21766, found. 491.21961.

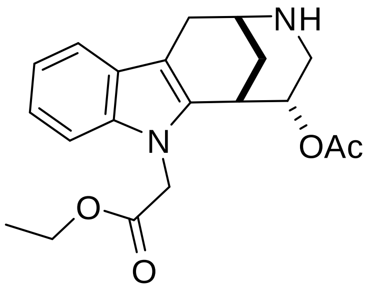

(±)-Ethyl 2-(5-acetoxy-1,2,3,4,5,6-hexahydro-7H-2,6-methanoazocino[5,4-b]indol-7-yl)acetate (12b):

Compound **12b** (72%, white solid) was synthesised from **4b** following General Procedure **D**.

^{1}H NMR (CDCl3, 400MHz): δ 1.26 (t, *J*=7.1 Hz, 3H), 2.01 (s, 3H), 2.06 (d, *J*=14.0 Hz, 1H), 2.21 (d, *J*=12.6 Hz, 1H), 2.67 (t, *J*=11.3 Hz, 1H), 2.80 (bs, 1H), 2.85 (d, *J*=17.1 Hz, 1H), 2.99 (dd, *J*=4.7, 12.0 Hz, 1H), 3.22 (dd, *J*=6.2, 17.2 Hz, 1H), 3.41 (s, 1H), 3.54 (s, 1H), 4.12-4.26 (m, 2H), 4.87 (s, 2H), 5.08 (dt, *J*=4.5, 9.2 Hz, 1H), 7.13-7.17 (m, 1H), 7.20-7.26 (m, 2H), 7.54 (d, *J*=7.7 Hz, 1H).).

^{13}C NMR (CDCl3, 100MHz): δ 14.2, 21.1, 28.8, 31.1, 33.0, 42.1, 44.7, 45.2, 61.5, 72.7, 108.8, 111.4, 118.4, 119.5, 121.7, 126.7, 134.1, 137.0, 168.8, 170.0.

HRMS (ESI)**:** calc. for $[M+H]^+$ $C_{20}H_{25}N_2O_4$: 357.18088, found. 357.18259.

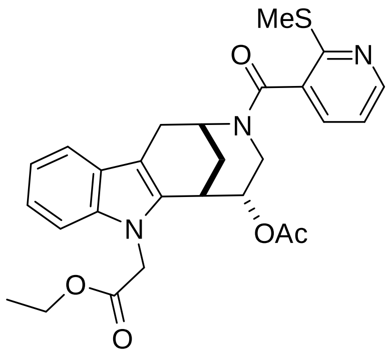

(±)-Ethyl 2-(5-acetoxy-3-(2-(methylthio)nicotinoyl)-1,2,3,4,5,6-hexahydro-7H-2,6-methanoazocino[5,4-b]indol-7-yl)acetate (5b):

Compound **5b** (71%, white solid) was synthesised from **12b** following General Procedure **E**.

^{1}H NMR (CDCl3, 400MHz) (rotameric mixture)**:** δ 1.25 (t, *J*=7.12 Hz, 3H), 1.94-2.00 (m, 2H), 2.08 (s, 1H), 2.11-2.19 (m, 1H), 2.27 (bs, 1H), 2.59-2.66 (m, 3H), 2.75-3.09 (m, 3H), 3.33-3.38 (m, 1H), 3.50 (dd, *J*=2.8, 15.0 Hz, 1H), 4.12-4.24 (m, 2H), 4.71-4.90 (m, 2H), 5.01-5.24 (m, 1H), 5.47 (s, 1H), 7.07-7.24 (m, 4H), 7.47-7.51 (m, 1H), 7.56 (d, *J*=7.71 Hz, 1H), 8.38-8.54 (m, 1H).Fe

^{13}C NMR (CDCl3, 100MHz) (rotameric mixture)**:** δ 13.3, 13.4, 14.2, 21.0, 21.1, 27.1, 28.4, 30.3, 30.8, 30.9, 31.4, 42.8, 44.8, 61.8, 71.0, 71.8, 109.0, 109.1, 110.4, 118.4, 118.6, 119.4, 120.0, 120.0, 122.3, 122.3, 126.5, 126.7, 130.7, 131.2, 132.8, 132.9, 134.0, 134.3, 137.2, 149.6, 156.0, 167.3, 167.4, 168.7, 169.8, 169.8

HRMS (ESI)**:** calc. for $[M+H]^+$ $C_{27}H_{30}N_3O_5S$: 508.19007, found. 508.19232.

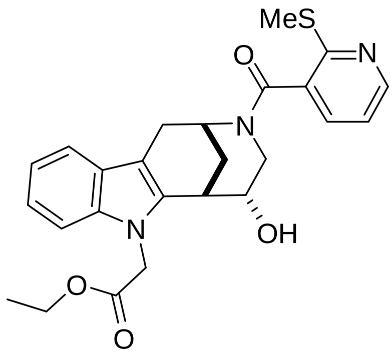

(±)-Ethyl 2-(5-hydroxy-3-(2-(methylthio)nicotinoyl)-1,2,3,4,5,6-hexahydro-7H-2,6-methanoazocino[5,4-b]indol-7-yl)acetate (5c):

Compound **5b** (0.39 g, 0.786 mmol) and LiOH (0.110 g, 4.61 mmol) were dissolved in a mixture of THF (0.07M) / H_2O (0.16M). The reaction was allowed to stir for one hour then neutralized with HCl 1M to pH~3 and extracted with CH_2Cl_2 (3x). Solvents were evaporated and the crude was dissolved in EtOH (0.1 M) and sulphuric acid was added (8 M). Reaction was allowed to stir overnight and neutralized with Na_2CO_3. The mixture was then partitioned between water and CH_2Cl_2 and the aqueous layer was extracted by CH_2Cl_2 (3x) and dried over $MgSO_4$. After filtration and evaporation of the solvents under reduced pressure, the crude product was purified by preparative HPLC (10% to 50% ACN/H_2O with 0.1% TFA) to yield compound **5c** (53%) as a light yellow solid.

^{1}H NMR (CDCl3, 400MHz) (rotameric mixture): δ 1.28 (td, *J*=2.6, 7.1 Hz, 3H), 1.94-2.12 (m, 1H), 2.19-2.35 (m, 1H), 2.58-2.65 (m, 3H), 3.02 (bs, 2H), 3.26-3.36 (m, 1H), 3.44-3.47 (m, 1H), 4.00-4.11 (m, 1H), 4.21 (qd, *J*=3.5, 7.2 Hz, 2H), 4.68 (dd, *J*=5.3, 13.4 Hz, 0.4H), 4.84-5.07 (m, 4H), 5.44 (bs, 0.6H), 7.06-7.24 (m, 4H), 7.46 (bs, 1H), 7.54 (dd, *J*=7.8, 15.1 Hz, 1H), 8.50 (dd, *J*=1.5, 5.1 Hz, 0.6H), 8.57 (dd, *J*=1.4, 4.9 Hz, 0.4H).

^{13}C NMR (CDCl3, 100MHz) (rotameric mixture)**:**13.4, 13.5, 14.3, 28.4, 30.3, 31.5, 33.5, 33.6, 42.4, 42.9, 45.1, 49.1, 62.1, 62.3, 69.4, 70.1, 109.0, 109.1, 118.3, 118.6, 119.5, 120.1, 120.2, 122.3, 122.4, 126.5, 126.8, 132.8, 133.3, 134.1, 134.5, 137.3, 137.5, 149.4, 149.5, 156.1, 167.2, 167.3, 170.2, 170.6.

HRMS (ESI)**:** calc. for $[M+H]^+$ $C_{25}H_{28}N_3O_4S$: 466.17950, found. 466.17937.

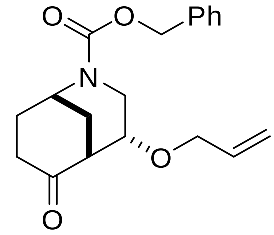

(±)-Benzyl 4-(allyloxy)-6-oxo-2-azabicyclo[3.3.1]nonane-2-carboxylate (2d):

To a solution of compound **1** (0.103 g, 0.356 mmol) and allyl ethylcarbonate (1469-70-1) (0.120 mL, 0.712 mmol) in THF (1.8 mL, 0.2M), a solution of $Pd(PPh_3)_4$ (0.021 mg, 0.018 mmol) in THF (1.8 mL; 0.01M) was added. The reaction was allowed to stir at 80° for 2.5 hours before filtering it through Celite and evaporating the solvents under reduced pressure. The crude product was purified by flash chromatography (1 to 5% E.A./CH_2Cl_2) to obtain compound **2d** (50.7 mg, 43%) as colorless oil.

^{1}H NMR (CDCl3, 400MHz) (rotameric mixture 1:1): δ 1.88-2.06 (m, 3H), 2.15-2.27´(m, 1H), 2.42-2.61 (m, 2H), 3.06 (bs, 1H), 3.15-3.23 (m, 1H), 3.76 (td, *J*=11.5, 5.9 Hz, 1H), 3.94-4.03 (m, 1H), 4.14-4.21 (m, 1H), 4.34 (ddd, *J*=20.9, 14.4, 7.5 Hz, 1H), 4.42(bs, 0.5H), 4.51 (bs, 0.5H), 5.14-5.20 (m, 3H), 5.25 (d, *J*=17.3 Hz, 1H), 5.85 (ddd, *J*=22.7, 10.9, 5.8 Hz, 1H), 7.31-7.38 (m, 5H).

HRMS (ESI)**:** calc. for $[M+H]^+$ $C_{19}H_{24}NO_4$: 330.16998, found: 330.17035

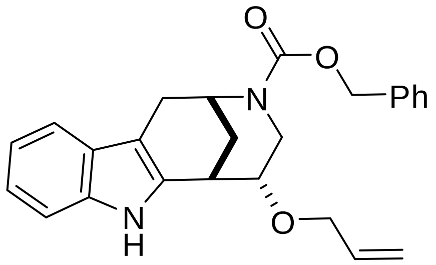

(±)-Benzyl 5-(allyloxy)-1,2,4,5,6,7-hexahydro-3H-2,6-methanoazocino[5,4-b]indole-3-carboxylate (3d):

Compound **3d** (35 mg, 73%) was synthesized from **2d** following General Procedure **A** and isolated with a 10% impurity.

^{1}H NMR (CDCl3, 400MHz) (rotameric mixture 1:1): δ 1.98-2.09 (m, 2H), 1.98-2.09 (m, 2H), 2.54 (dd, *J*=12.0, 11.2, 0.5H), 2.60 (t, *J*=8.0 Hz, 0.5H), 2.78 (d, *J*=16.8 Hz, 0.5H), 2.83 (d, *J*=17.0 Hz, 0.5H), 3.07-3.13 (m, 1H), 3.32-3.33 (m, 1H), 3.69 (ddd, *J*=15.5, 10.7, 4.8 Hz, 1H), 4.03-4.10 (m, 2H), 4.23 (dd, *J*=12.6, 5.3 Hz, 0.5H), 4.74 (bs, 0.5H), 4.81 (bs, 0.5H), 5.12-5.20 (m, 3.5H), 5.27 (dd, *J*=17.3, 6.5 Hz, 1H), 5.90 (ddd, *J*=22.6, 10.7, 5.5 Hz, 1H), 7.07-7.10 (m, 1H), 7.14-7.18 (m, 2H), 7.30-7.36 (m, 3H), 7.41 (d, *J*=4.2 Hz, 2H), 7.45 (dd, *J*=15.8, 7.8 Hz, 1H), 7.92 (bs, 0.5H), 7.96 (bs, 0,5H).

^{13}C NMR (CDCl3, 100MHz): δ 21.2, 21.6, 27.8, 28.2, 30.2, 30.6, 33.0, 33.2, 42.6, 42.7, 67.3, 67.5, 67.0, 70.0, 75.5, 75.8, 109.2, 109.5, 111.0, 111.1, 117.2, 118.0, 118.1, 119.4, 121.6, 125.4, 127.0, 127.0, 128.0, 128.1, 128.1, 128.2, 128.4, 128.6, 128.7, 129.2, 132.8, 132.9, 135.0, 136.2, 136.9, 137.0, 138.0, 155.7, 155.7

HRMS (ESI)**:** calc. for $[M+H]^+$ $C_{25}H_{27}N_2O_3$: 403.20162, found: 403.20132

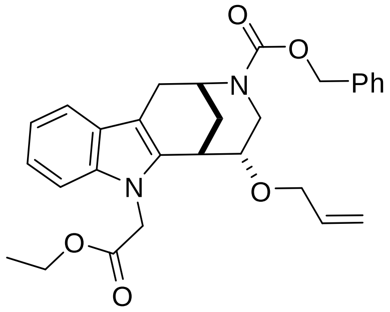

(±)-Benzyl 5-(allyloxy)-7-(2-ethoxy-2-oxoethyl)-1,2,4,5,6,7-hexahydro-3H-2,6-methanoazocino[5,4-b]indole-3-carboxylate (4d):

Compound **4d** (0.150 mg, 85%) was synthesized from **3d** following General Procedure **B.**

^{1}**H NMR** (CDCl3, 400MHz) (rotameric mixture 1:1): δ 1.24 (t, *J*=6.9 Hz, 3H), 1.97-2.07 (m, 2H), 2.59-2.70 (m, 1H), 2.80 (t, *J*=17.9 Hz, 1H), 3.12-3.20 (m, 1H), 3.40 (d, *J*=3.0 Hz, 1H), 3.67-3.73 (m, 1H), 3.97-4.27 (m, 5H), 4.75-4.77 (m, 1H), 4.81-4.82 (m, 1H), 5.13-5.30 (m, 5H), 5.81-5.90 (m, 1H), 7.08-7.11 (m, 1H), 7.13-7.21 (m, 2H), 7.31-7.37 (m, 3H), 7.41 (d, *J*=4.3 Hz, 2H), 7.46 (dd, *J*=11.0, 8.0 Hz, 1 H).

^{13}**C NMR** (CDCl3, 100MHz): δ 14.3, 30.7, 31.0, 32.0, 32.0, 42.0, 42.1, 45.1, 45.1, 45.2, 61.4, 67.3, 67.5, 70.2, 70.4, 76.3, 76.6, 108.9, 109.6, 109.9, 117.4, 117.5, 118.2, 118.3, 119.5, 121.7, 126.7, 126.7, 128.0, 128.1,128.1, 128.2, 128.6, 128.7, 134.1, 134.1, 134.6, 134.6, 136.9, 137.2, 155.6, 169.5, 169.6

HRMS (ESI)**:** calc. for $[M+H]^+$ $C_{29}H_{33}N_2O_5$: 489.23840, found: 489.23778

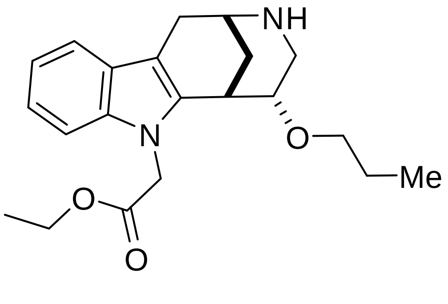

(±)-Ethyl 2-(5-propoxy-1,2,3,4,5,6-hexahydro-7H-2,6-methanoazocino[5,4-b]indol-7-yl)acetate (12d):

Compound **12d** (0.041 g, 93%) was synthesized from **4d** following General Procedure **D**.

^{1}H NMR (CDCl3, 400MHz): δ 0.87 (t, *J*=7.4 Hz, 3H), 1.26 (t, *J*=7.1 Hz, 3H), 1.54 (dq, *J*=14.1, 7.0 Hz, 3H), 1.97-2.18 (m, 2H), 2.53 (d, *J*=13.6 Hz, 1H), 2.74 (t, *J*=11.5 Hz, 1H), 3.22-3.48 (m, 6H), 4.04-4.07 (m, 1H), 4.13-4.25 (m, 2H), 4.78 (d, *J*=18.0 Hz, 1H), 5.14 (d, *J*=18.0 Hz, 1H), 7.08-7.24 (m, 3H), 7.46-7.51 (m, 1H).

^{13}C NMR (CDCl3, 100MHz): δ 10.7, 14.3, 23.2, 25.4, 28.9, 31.3, 40.4, 44.9, 46.5, 61.6, 71.7, 74.8, 108.4, 109.0, 118.5, 119.9, 122.2, 126.2, 133.7, 137.2, 169.3

HRMS (ESI)**:** calc. for $[M+H]^+$ $C_{21}H_{28}N_2O_3$: 357.21727, found: 357.21660

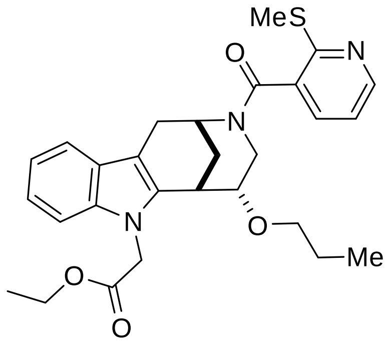

(±)-Ethyl 2-(3-(2-(methylthio)nicotinoyl)-5-propoxy-1,2,3,4,5,6-hexahydro-7H-2,6-methanoazocino[5,4-b]indol-7-yl)acetate (5d):

Compound **5d** (0.022 g, 39%) was synthesized from **12d** following General Procedure **E**.

^{1}H NMR (CDCl3, 700MHz) (rotameric mixture 1:1): δ0.8 (t,*J*=7.4 Hz, 1H), 0.91 (t, *J*=7.4 Hz, 2H), 1.25 (q, *J*=7.1 Hz, 4H), 1.41-1.46 (m, 1H), 1.51-1.60 (m, 2H), 1.94-2.33 (m, 2H), 2.56-2.66 (m, 3H), 3.05-3.34 (m, 2H), 3.46 (s, 1.5H), 3.48 (s, 1H), 3.57 (dd, *J*=15.5, 7.0 Hz, 0.5H), 3.76 (bs, 0.5H), 3.99 (s, 0.5H), 4.13-4.24 (m, 2H), 4.78 (dd, *J*=23.4, 18.0 Hz, 1H), 4.86 (dd, *J*=12.9, 4.9 Hz, 0.5H), 5.16 (d, *J*=18.0 Hz,

0.5H), 5.27 (d, *J*=18.1 Hz, 0.5H), 5.42 (s, 0.5H), 7.05-7.23 (m, 4H), 7.41-7.52 (m, 2H), 8.48 (d, *J*=4.8 Hz, 0.5H), 8.54 (d, *J*=4.8 Hz, 0.5H).

^{13}C NMR (CDCl3, 100MHz): δ 10.7, 10.8, 13.3, 13.4, 14.3, 23.1, 23.4, 28.5, 304, 31.7, 32.4, 32.6, 42.9, 45.0, 45.0, 51.0, 61.5, 71.8, 71.3, 108.9, 109.0, 109.7, 118.1, 118.2, 118.4, 119.3, 119.5, 119.6, 121.8, 121.8, 126.5, 126.7, 133.7, 134.2, 134.5, 137.2, 137.2, 149.2, 149.3, 161.3, 161.4, 166.8, 166.9, 169.5, 169.6

HRMS (ESI)**:** calc. for $[M+H]^+$ $C_{28}H_{33}N_3O_4S$:508.21918, found: 508.22645

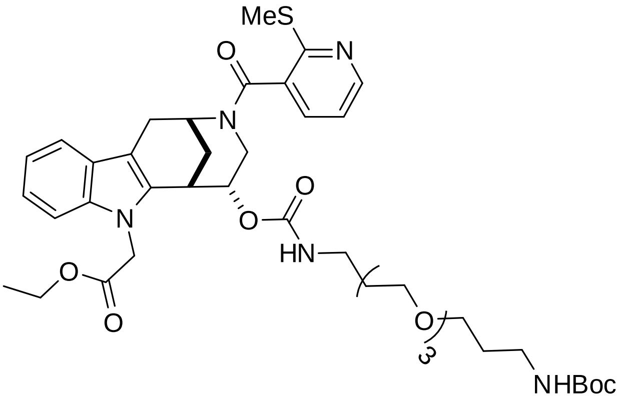

(±)-Ethyl 2-(5-(((2,2-dimethyl-4-oxo-3,9,12,15-tetraoxa-5-azaoctadecan-18-yl)carbamoyl)oxy)-3-(2-(methylthio)nicotinoyl)-1,2,3,4,5,6-hexahydro-7H-2,6-methanoazocino[5,4-b]indol-7-yl)acetate (racemic mixture) (5e):

Compound **5c** (30mg, 0.064mmol), 4-nitrophenyl carbonochloridate (52mg, 0.258mmol) (CAS: 7693-46-1), DMAP (24mg, 0.192mmol) and triethylamine (18mL, 0.128 mmol) were suspended in DCE (4mL, 0.016M). The mixture was heated to 60° overnight (after 15/20min everything solubilizes). Then tert-butyl (3-(2-(2-(3-aminopropoxy)ethoxy)ethoxy)propyl)carbamate (410mg, 1.28mmol) and the reaction was allowed to stir for 5 more hours. Solvents were evaporated, the crude product was dissolved in MeCN (1mL) and subjected for preparative HPLC purification. (10% to 50% ACN/H2O with 0.1% TFA). After evaporation of the solvents compound **5e** (31.2mg, 60%) is obtained as a white solid.

^{1}H NMR (CDCl3, 400MHz) (rotameric mixture): δ 1.23 (td, *J*=2.4, 7.1 Hz, 3H), 1.40-1.43 (m, 9H), 1.61-1.83 (m, 4H), 1.95-2.26 (m, 2H), 2.54 (bs, 1.8H), 2.62 (s, 1.2H), 2.70-2.76 (m, 1H), 2.96-3.59 (m, 20H), 4.02 (bs, 0.4H), 4.10-4.22 (m, 2H), 4.66-5.14 (m, 4H), 5.31 (bs, 0.6H), 5.46 (bs, 1H), 7.01-7.23 (m, 4H), 7.42-7.48 (m, 1.4H), 7.54 (d, *J*=7.5 Hz, 0.6H), 8.44 (dd, *J*=1.6, 4.9 Hz, 0.6H), 8.51 (dd, *J*=1.5, 4.9 Hz, 0.4H).).

^{13}C NMR (CDCl3, 100MHz) (rotameric mixture): 13.1, 13.2, 14.3, 27.1, 28.7, 29.5, 29.7, 30.2, 31.1, 31.3, 31.4, 38.6, 39.2, 39.4, 39.5, 42.4, 44.9, 48.7, 61.7, 69.5, 69.6, 70.23, 70.31, 70.28, 70.49, 70.52, 70.56, 70.61, 71.0, 71.4, 109.0, 110.3, 118.3, 118.6, 119.1, 119.2, 119.9, 122.1, 126.6, 126.8, 130.8, 133.4, 133.5, 133.8, 137.3, 149.7, 155.2, 155.4, 156.1, 156.2, 167.1, 167.3, 169.1, 169.2.

HRMS (ESI): calc. for [M+H]+ C41H58N5O10S: 812.38989, found. 812.39275

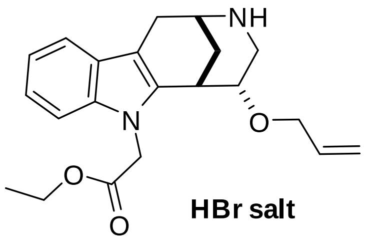

(±)-Ethyl 2-(5-(allyloxy)-1,2,3,4,5,6-hexahydro-7H-2,6-methanoazocino[5,4-b]indol-7-yl)acetate (12z):

Compound **12z** (0.05 g, 79%) was synthesized from **4d** following General Procedure **G**. The crude product was purified by chromatography (5 to 10% MeOH/CH_2C_{l2}).

^{1}H NMR (CDCl3, 400MHz) (rotameric mixture 1:1): δ 1.25 (t, *J*=7.1 Hz, 3H), 2.14 (d, *J*=12.5 Hz, 1H), 2.61 (d, *J*=13.8 Hz, 1H), 2.76-2.84 (m, 1H), 3.34 (d, *J*=2.7 Hz, 2H), 3.43 (d, *J*=10.6 Hz, 1H), 3.50 (d, *J*=2.2 Hz, 1H), 4.06 (d, *J*=5.5 Hz, 2H), 4.12-4.24 (m, 4H), 4.79 (d, *J*=18.0 Hz, 1H), 5.07 (d, *J*=18.0 Hz, 1H), 5.21 (d, *J*=10.4 Hz, 1H), 5.25 (dd, *J*=17.2, 1.2 Hz, 1H), 5.83 (ddd, *J*=22.7, 10.9, 5.7 Hz, 1H), 7.13-7.16 (m, 1H), 7.21-7.25 (m, 2H), 7.51 (d, *J*=7.8 Hz, 1H), 9.47 (bs, 2H).

^{13}C NMR (CDCl3, 100MHz): δ 14.3, 25.0, 28.5, 31.1, 40.2, 45.0, 46.8, 61.7, 71.1, 73.8, 108.1, 109.1, 118.4, 118.6, 120.0, 122.4, 126.0, 133.2, 133.7, 137.3, 169.2

HRMS (ESI)**:** calc. for $[M+H]^+$ $C_{21}H_{27}N_2O_3$: 355.20162, found: 355.20058

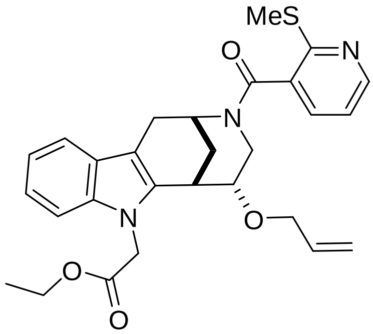

(±)-Ethyl 2-(5-(allyloxy)-3-(2-(methylthio)nicotinoyl)-1,2,3,4,5,6-hexahydro-7H-2,6-methanoazocino[5,4-b]indol-7-yl)acetate (5z):

Compound **5z** (0.039 g, 61%) was synthesized from **12z** following General Procedure **E**.

^{1}H NMR (CDCl3, 500MHz) (rotameric mixture 1:1): δ 1.24 (q, *J*=6.9 Hz, 3H), 1.94-2.33 (m, 2.5H), 2.59-2.65 (m, 4H), 2.79-3.07 (m, 1.5H), 3.30-3.35 (m, 1H), 3.45-3.47 (m, 1.5H), 3.82 (bs, 1.5H), 4.00 (bs, 0.5H), 4.06 (dd, *J*=12.6, 5.6 Hz, 0.5H), 5.02-5.11 (m, 1.5H), 5.19-5.29 (m, 1.5H), 5.42 (s, 0.5H), 5.71 (qd, *J*=11.1, 5.7 Hz, 0.5H), 5.89 (ddd, *J*=22.5, 10.7, 5.5 Hz, 0.5H), 7.04 (dd, *J*=7.1, 5.2 Hz, 0.5H), 7.09-7.14 (m, 1.5H), 7.18-7.23 (m, 2H), 8.47 (dd, *J*=4.8, 1.3 Hz, 0.5H), 8.53 (dd, *J*=4.8 Hz, 0.5H).

^{13}C NMR (CDCl3, 100MHz): δ 13.1, 13.3, 14.3, 28.4, 31.7, 32.3, 32.5, 42.8, 45.0, 45.0, 49.0, 51.0, 61.5, 61.5, 70.4, 70.5, 76.2, 109.0, 109.0, 117.4, 117.8, 118.2, 118.4, 119.2, 119.6, 119.6, 121.8, 121.8, 126.4, 126.6, 133.6, 134.0, 134.2, 134.4, 134.5, 137.1, 137.2, 149.4, 149.4, 166.9, 167.0, 169.4, 169.5

HRMS (ESI)**:** calc. for $[M+H]^+$ $C_{28}H_{32}N_3O_4S$: 506.21080, found: 506.21021

R^2

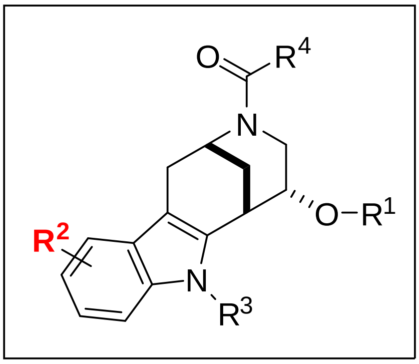

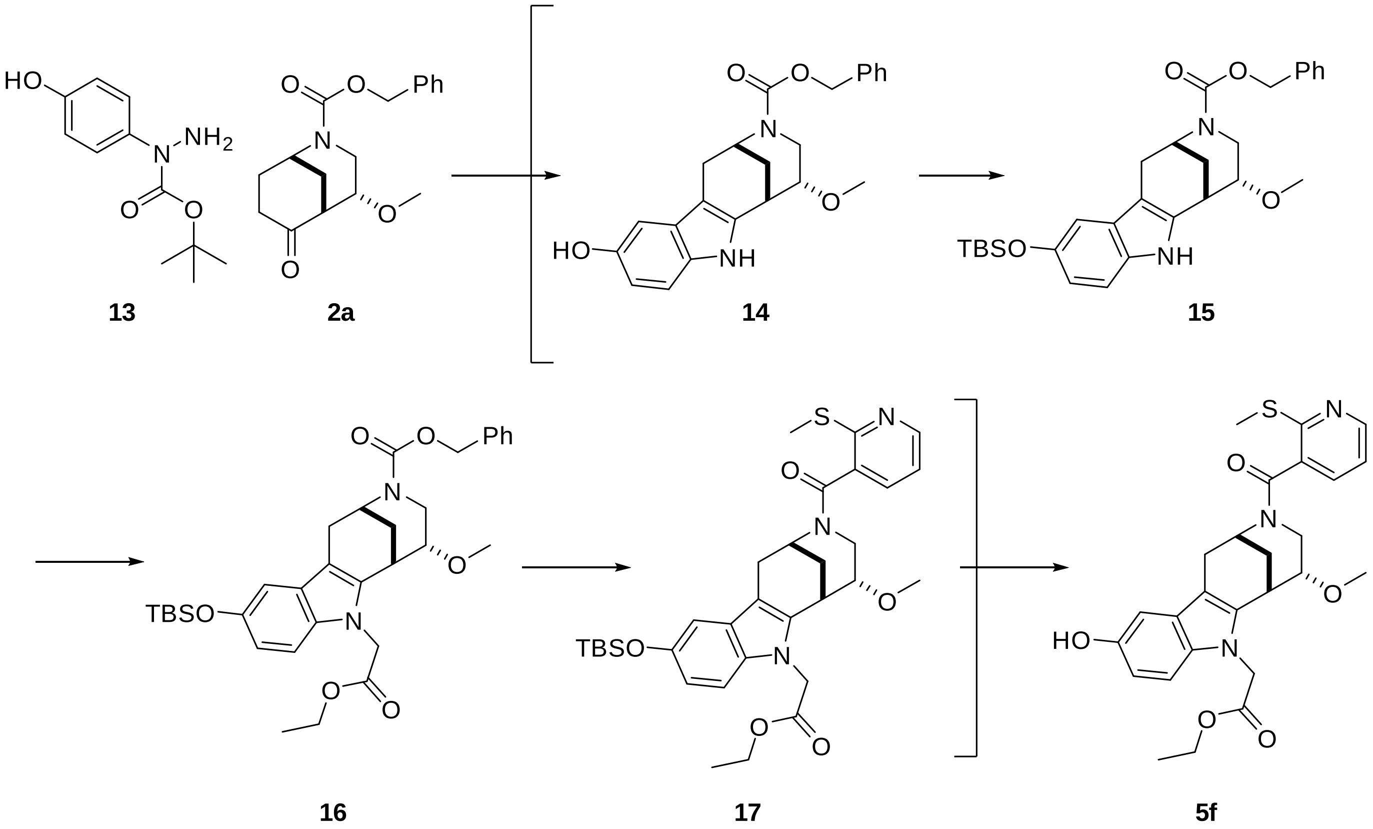

Supporting Scheme S2: Synthesis of the 5-*O* indole-substituted indomorphan analogues. The non-alkylated, hydroxyl indole was found to be unstable, thus most of the steps were telescoped and the crude mixtures were used directly for subsequent synthetic steps, involving minimal purification.

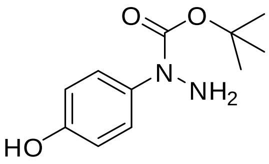

tert-butyl 1-(4-hydroxyphenyl)hydrazine-1-carboxylate (13):

By the method of Johnson M.G., et al.[2] CuI (38 mg, 0.1 mol%) was added to a stirred solution of 4-iodophenol (440 mg, 2 mmol, 1 equiv.), tert-butyl carbazate (318 mg, 2.4 mmo, 1.2 equiv.), 1,10-phenantroline (36 mg, 0.2 mol%), and cesium carbonate (977 mg, 3 mmol, 1.5 equiv.) in DMF (4 mL, 0.5 M) and the reaction mixture was heated at 80 ˚C. After 22 h the reaction mixture was allowed to cool to rt and quenched by the addition of H2O (20 mL). The crude was dilute with EtOAc (40 mL) and layers separated. The organic layer was washed sequentially with H2O (40 mL), sat. brine (2 × 20 mL) and sat. LiCl (2 × 10 mL), dried over MgSO4, filtered and conc. in vacuo to give a crude mixture. Purification by flash chromatography eluting with 20-50% EtOAc in Petrol afforded the product **13** as a yellow amorphous solid (350 mg, 80% yield) with spectroscopic data matching those reported in literature.

^{1}H NMR (CDCl3, 500MHz): δ 1.46 (s, 9H), 4.91 (s, br, 2H), 6.70 (d, *J*=9.2 Hz, 2H), 7.16 (d, *J*=9.2 Hz, 2H).

LCMS (m/z): 247.1 $(M + Na)^+$.

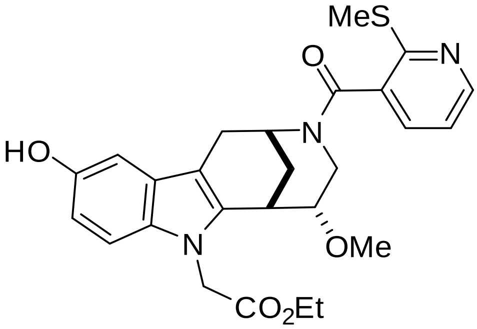

(±)-Ethyl 2-(-10-hydroxy-5-methoxy-3-(2-(methylthio)nicotinoyl)-1,2,3,4,5,6-hexahydro-7H-2,6-methanoazocino[5,4-b]indol-7-yl)acetate (5f):

By the method of Johnson et al.[3] pTSA (6 equiv.) was added to a stirred solution of the ketone (1 equiv.) and 4-hydroxyphenly (tert-butyl)hydrazite (1.5 equiv) in EtOH (0.08 M) and the reaction mixture was heated to reflux. After 2.5 h, the reaction mixture was allowed to cool to rt, neutralized with sat. aq. NaHCO3 solution and extracted with CH_2Cl_2 (4 × 10 mL). The combined organic layers were dried over MgSO4, filtered and conc. in vacuo. The crude was filtered through a silica pad eluting with 1:1 Petrol—EtOAc (50 mL) and conc. in vacuo to give a crude. The crude was rediluted in Toluene (1 M) and Et3N (3 equiv.) and TBDMS chloride as a 1.5 M solution in toluene (3 equiv.) was added and the resulting mixture was stirred at rt. After 24 h, the reaction mixture was conc. in vacuo and filtered through a silica pad eluting with 9:1 CH_2Cl_2—EtOAc (50 mL) and conc. in vacuo to give a crude which was directly submitted to General procedure **C** using ethyl bromoacetate. Filtration through a silica pad eluting with 7:3 Petrol—EtOAc (50 mL) and conc. in vacuo afforded a crude product which required no further purification as judged by spectroscopic data 1H NMR (CDCl3, 500MHz): δ 0.20 (s, 6H), 0.80 (s, 9H), 1.04 (app. t, *J*=7.3 Hz, 3H), 1.41 (s, br, 1H), 1.77-1.86 (m, 2H), 2.34-2.60 (m, 2H), 2.84-2.97 (m, 2H), 3.13-3.19 (m, 2H), 3.30-3.40 (m, 1H), 3.89-4.18 (m, 3H), 4.45-4.66 (m, 2H), 4.79-4.93 (m, 1H), 4.95-5.05 (m, 2H), 6.53 (d, *J*=8.5 Hz, 1H), 6.64-6.69 (m, 1H), 6.82 (d, *J*= 9 Hz, 1H), 7.10-7.25 (m, 5H), and used directly as in General Procedure **F**. Filtration through a silica pad eluting with 9:1 CH2Cl2—EtOAc (50 mL) and conc. in vacuo afforded a crude product which required no further purification as judged by spectroscopic data 1H NMR (CDCl3, 500MHz): δ 0.20 (s, 12H), 0.84 (s, 18H), 1.14-1.19 (m, 6H), 1.65-2.02 (m, 4H), 2.25-2.47 (m, 8H), 2.60-2.82 (m, 3H), 2.97 (s, 3H), 3.01-3.09 (m, 1H), 3.14 (s, 2H), 3.22 (s, 6H), 3.46 (s, br, 1H), 3.78 (s, br, 1H), 3.92-4.03 (m, 4H), 4.47-4.57 (m, 2H), 4.65-4.96 (m, 3H), 6.50-6.58 (m, 2H), 6.63-6.74 (m, 2H), 6.78-6.94 (m, 4H), 7.16-7.34 (m, 2H), 8.25 (dd, *J*=4.9 and 2.2 Hz, 1H), 8.32 (dd, *J*=4.9 and 2.1 Hz, 1H), and used directly for the next synthetic step. The crude was diluted in

THF (0.07M) and TBAF as a 1 M solution in THF (1.2 equiv.) was added and the reaction mixture was stirred at rt. After 2 h, sat. aq. NH4Cl solution (2 × reaction volume) was added and the aqueous layer was extracted with EtOAc (4 × reaction volume). The combined organic layers were dried over MgSO4, filtered and conc. in vacuo to give a crude. Purification by flash chromatography eluting with 20% EtOAc in CH_2Cl_2 afforded the desired compound **5f** in 18% yield over 6 steps.

^{1}H NMR (CDCl3, 500MHz): δ 1.23-1.28 (m, 6H), 1.95 (d, *J*=10.2 Hz, 1H), 2.11 (d, *J*=10.2 Hz, 1H), 2.55-2.70 (m, 4H), 2.77 (s, 6H), 3.03 (m, 1H), 3.17-3.26 (m, 4H), 3.32 (dd, *J*= 4.8 and 12.7 Hz, 1H), 3.42 (s, 6H), 3.43-3.46 (m, 2H), 3.65-3.72 (m, 1H), 3.93-3.98 (m, 1H), 3.93-3.98 (m, 1H), 4.12-4.26 (m, 4H), 4.75 (app. t, *J*= 17.7 Hz, 2H), 4.90 (dd, *J*=4.8 and 12.9 Hz, 1H), 4.98 (d, *J*=17.7 Hz, 1H), 5.10 (d, *J*=18 Hz, 1H), 5.41 (m, 1H), 6.74-6.80 (m, 2H), 6.87 (d, *J*=16 Hz, 2H), 7.03-7.08 (m, 2H), 7.10-7.16 (m, 1H), 7.20 (dd, *J*=7.0 Hz, 1H), 7.58 (app. d, *J*=7.0 Hz, 1H), 8.54 (d, *J*=5.2 Hz, 1H), 8.59 (d, *J*=5.2 Hz, 1H).

^{13}C NMR (CDCl3, 100MHz): δ 14.2, 22.7, 31.4, 43.0, 44.8, 49.1, 56.8, 61.3, 109.5, 111.2, 119.5, 126.8, 127.0, 132.4, 134.5, 134.8, 148.1, 148.4 149.8, 149.9, 166.1, 166.4 169.4, 169.5.

HRMS (ESI): calc. for $[M+Na]^+$ $C_{26}H_{29}N_3O_5SNa$: 518.1720; Found: 518.1724.

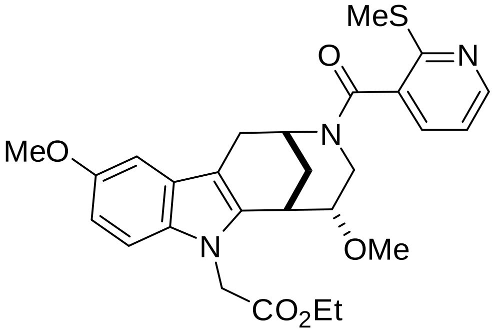

(±)-Ethyl 2-((2R,5R,6S)-5,10-dimethoxy-3-(2-(methylthio)nicotinoyl)-1,2,3,4,5,6-hexahydro-7H-2,6-methanoazocino[5,4-b]indol-7-yl)acetate (5aa):

Potassium hydroxide (45 mg, 0.8 mmol, 4 equiv.) was added to a stirred solution of the phenol (100 mg, 0.2 mmol, 1 equiv.) in DMSO (1.5 mL, 1.33 M) and the reaction mixture was stirred at rt. After 30 min, iodomethane (0.8 mmol, 4 equiv.) was added and the resulting mixture was stirred at rt. After 4 h the

reaction mixture was diluted with EtOAc (15 mL), and washed with sat. aq. NH4Cl solution (2 × 5 mL). The organic layer was dried over MgSO4, filtered and conc. in vacuo to give a crude. Purification by preparative HPLC eluting with 10-100% MeCN in H2O, afforded the desired compound in 65% yield.

^{1}H NMR (CDCl3, 500MHz): δ 1.22-1.33 (m, 6H), 1.90-2.02 (m, 4H), 2.12 (d, *J*=11.2 Hz, 1H), 2.16-2.24 (m, 4H), 2.57-2.69 (m, 4H), 2.71 (s, 5H), 2.98-3.10 (m, 2H), 3.15-3.25 (m, 2H), 3.30 (dd, *J*= 5.9 and 17.2 Hz, 1H), 3.34 (dd, *J*= 4.4 and 12.8 Hz, 1H), 3.43 (s, 2H), 3.44-3.48 (m, 2H), 3.66-3.73 (m, 1H), 3.87 (s, 2H), 3.89 (s, 2H), 4.01 (s, 1H), 4.11-4.26 (m, 4H), 4.75 (d, *J*= 18.4 Hz, 1H), 4.78 (d, *J*= 18.4, 1H), 4.92 (dd, *J*=4.7 and 13.1 Hz, 1H), 5.00 (d, *J*=17.9 Hz, 1H), 5.12 (d, *J*=17.9 Hz, 1H), 5.41-5.47 (m, 1H), 6.87 (d, *J*=8.7 Hz, 2H), 6.89-6.94 (m, 1H), 6.96-7.02 (m, 1H), 7.07-7.15 (m, 2H), 7.15-7.20 (m, 1H), 7.41-7.50 (m, 1H), 7.51-7.58 (m, 2H), 8.52 (d, *J*=4.5 Hz, 1H), 8.57 (d, *J*=4.5 Hz, 1H).

^{13}C NMR (CDCl3, 100MHz): δ 14.2, 22.7, 31.4, 42.7, 44.9, 49.0, 56.0, 56.8, 61.3, 109.6, 111.4, 119.2, 126.4, 126.8, 132.2, 133.8, 134.3, 134.7, 148.9, 154.2, , 166.5, 169.2, 169.4.

HRMS (ESI): calc. for $[M+H]^+$ $C_{27}H_{32}N_3O_5S$: 510.2057; Found: 510.2062.

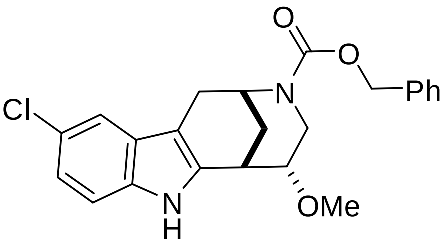

(±)-Benzyl 10-chloro-5-methoxy-1,2,4,5,6,7-hexahydro-3H-2,6-methanoazocino[5,4-b]indole-3-carboxylate (3g):

Compound **3g** (0.104 g, 73%) was synthesized from **2a** following General Procedure **A**, using 4-chlorophenylhydrazine hydrochloride (CAS: 1073-70-7).

^{1}H NMR (CDCl3, 400MHz) (rotameric mixture 1:1): δ 2.00-2.08 (m, 2H), 2.50 (dt, *J*=23.6, 11.8 Hz, 1H), 2.76 (t, *J*=17.6 Hz, 1H), 3.05 (dt, *J*=16.6, 5.1 Hz, 1H), 3.31-3.33 (m, 1H), 3.40 (d, *J*=8.2 Hz, 3H), 3.54 (dd, *J*=9.2, 4.3 Hz, 1H), 4.14 (dd, *J*=12.8, 5.2 Hz, 0.5H), 4.30 (dd, *J*=12.7, 5.2 Hz, 0.5H), 4.75 (bs,

0.5H), 4.83 (bs, 0.5H), 5.19 (d, *J*=18.9 Hz, 2H), 7.08 (d, *J*=8.6, 1.8 Hz, 1H), 7.18 (d, *J*=8.6 Hz, 1H), 7.35-7.42 (m, 6H), 8.09 (d, *J*=10.9 Hz, 1H).

^{13}C NMR (CDCl3, 100MHz): δ 27.6, 28.0, 30.0, 30.3, 42.3, 42.3, 45.2, 56.9, 67.4, 67.6, 77.4, 77.5, 77.9, 108.9, 109.2, 111.9, 112.0, 117.5, 117.6, 121.6, 125.0, 128.0, 128.0, 128.1, 128.2, 128.6, 128.7, 134.3, 134.4, 134.4, 136.8, 136.8, 155.6

HRMS (ESI)**:** calc. for $[M+H]^+$ $C_{23}H_{24}N_2O_3Cl$: 411.14700, found: 411.14757. Calc. for $[M+H]^+$ $C_{23}H_{24}N_2O_3{}^{37}Cl$: 413.14405, found: 413.14456

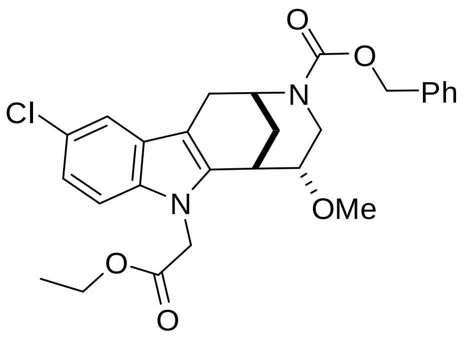

(±)-Benzyl 10-chloro-7-(2-ethoxy-2-oxoethyl)-5-methoxy-1,2,4,5,6,7-hexahydro-3H-2,6-methanoazocino[5,4-b]indole-3-carboxylate (4g):

Compound **4g** (0.253 g, 88%) was synthesized from **3g** following General Procedure **B**. Compound **4g** was isolated with a small impurity of the ethyl 2-bromoacetate, as an orange oil.

^{1}H NMR (CDCl3, 400MHz) (rotameric mixture 1:1): δ1.24 (t, *J*=7.1 Hz, 3H), 1.95-2.07 (m, 2H), 2.56 (dt, *J*=25.3, 11.8 Hz, 1H), 2.76 (t, *J*=16.6 Hz, 1H), 3.10 (ddd, *J*=15.5, 8.6, 5.9 Hz, 1H), 3.35 (d, *J*=4.7 Hz, 3H), 3.38 (sept, *J*=4.5 Hz, 1H), 4.11-4.22 (m, 2,5H), 4.33 (dd, *J*=12.6, 5.0 Hz, 0.5H), 4.74-4.79 (m, 1.5H), 4.84 (bs, 0.5H), 5.09 (dd, *J*=21.1, 18.3 Hz, 1H), 5.19 (d, *J*=14.6 Hz, 2H), 7.09-7.14 (m, 2H), 7.31-7.42 (m, 6H).

^{13}C NMR (CDCl3, 100MHz): δ 14.3, 27.78, 28.0, 30.4, 30.7, 31.8, 31.9, 41.6, 41.7, 45.0, 45.1, 56.8, 57.0, 61.4, 67.3, 67.5, 68.3, 77.4, 78.2, 78.6, 109.3, 109.6, 110.0, 117.7, 117.8, 121.7, 125.2, 127.6, 127.7, 127.9, 128.0, 128.1, 128.2, 128.6, 128.7, 135.5, 135.6, 136.8, 155.5, 169.0, 169.1

HRMS (ESI)**:** calc. for $[M+H]^+$ $C_{27}H_{30}N_2O_5Cl$: 497.18378, found: 497.18455 Calc. for $[M+H]^+$ $C_{27}H_{30}N_2O_5{}^{37}Cl$: 499.18083, found: 499.18182

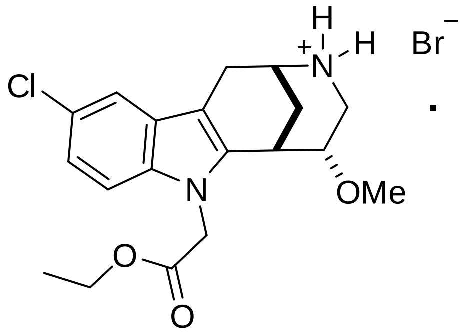

(±)-7-(2-ethoxy-2-oxoethyl)-10-chloro-5-methoxy-2,3,4,5,6,7-hexahydro-1H-2,6-methanoazocino[5,4-b]indol-3-ium bromide (racemic mixture) (12g):

Compound **12g** (39.0 mg, 50%) was synthesized from **4g** following General Procedure **G**. The crude product was purified by chromatography (5 to 10% MeOH/CH_2Cl_2).

^{1}H NMR (CDCl3, 400MHz): δ1.25 (t, *J*=7.1 Hz, 3H), 2.10 (d, *J*=12.3 Hz, 1H), 2.54 (d, *J*=13.8 Hz, 1H), 2.70 (dd, *J*=20.6, 11.1 Hz, 1H), 3.23 (s, 2H), 3.36 (s, 3H), 3.43-3.47 (m, 2H), 3.99-4.02 (m, 2H), 4.12-4.24 (m, 3H), 4.75 (d, *J*=18.0 Hz, 1H), 7.11 (d, *J*=8.7 Hz, 1H), 7.16 (dd, *J*=8.7, 1.8 Hz, 1H), 7.45 (d, *J*=1.5 Hz, 1H), 9.46 (bs, 2H).

^{13}C NMR (CDCl3, 100MHz): δ 14.3, 24.8, 28.3, 30.9, 40.0, 45.1, 46.7, 57.5, 61.8, 75.7, 61.8, 75.7, 107.8, 110.3, 118.0, 122.6, 125.8, 127.1, 134.7, 135.8, 168.8

HRMS (ESI)**:** calc. for $[M+H]^+$ $C_{19}H_{24}N_2O_3Cl$: 363.14700, found: 363.14740; calc. for $[M+H]^+$ $C_{19}H_{24}N_2O_3{}^{37}Cl$: 365.14405, found: 365.14441

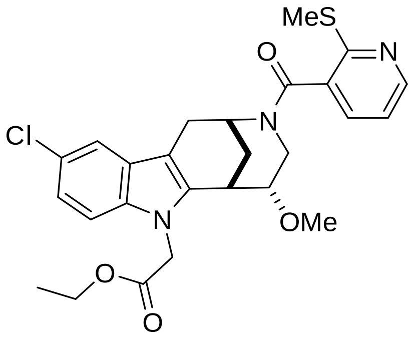

(±)-Ethyl 2-(10-chloro-5-methoxy-3-(2-(methylthio)nicotinoyl)-1,2,3,4,5,6-hexahydro-7H-2,6-methanoazocino[5,4-b]indol-7-yl)acetate (5g):

Compound **12g** (39.0 mg, 0.107 mmol), 2-(methylthio)nicotinoyl chloride (20.1 mg, 0.107 mmol) and triethylamine (0.0328 mL, 0.236 mmol) were dissolved in CH_2Cl_2 (1.2 mL, 0.09M). The reaction was allowed to stir overnight at room temperature, the solvents were then evaporated and the crude was purified by preparative HPLC (no TFA was added) to give compound **5g** (16.1 mg, 29%).

^{1}H NMR (CDCl3, 400MHz) (rotameric mixture 1:1.3): δ 1.24 (q, *J*=7.1 Hz, 3H), 1.93 (d, *J*=11.6 Hz, 0.5H), 2.07-2.32 (m, 1.5H), 2.58 (bs, 2H), 2.64 (s, 2H), 2.88-3.02 (m, 1.5H), 3.12-3.19 (m, 1.5H), 3.31 (ddd, *J*=23.4, 15.1, 5.3 Hz, 1H), 3.41 (s, 1.5H), 3.44 (d, *J*=3.4 Hz, 1H), 3.68-3.80 (m, 1H), 4.00 (bs, 0.5H), 4.10-4.25 (m, 2H), 4.75 (dd, *J*=18.0, 13.6 Hz, 1H), 4.91 (dd,*J*=12.8, 4.9 Hz, 0.5H), 5.00 (d, *J*=18.0 Hz, 0.5H), 5.11 (d, *J*=18.1 Hz, 0.5H), 5.43 (bs, 0.5H), 7.04 (dd, *J*=7.5, 5.0 Hz, 0.5H), 7.09-7.16 (m, 2.5H), 7.41 (bs, 1H), 7.47 (bs, 1H), 8.47 (dd*J*=4.9, 1.7 Hz, 0.5H), 8.54 (dd, *J*=4.9, 1.7 Hz, 0.5H).

^{13}C NMR (CDCl3, 100MHz): δ 13.2, 13.3, 14.3, 28.3, 30.2, 31.5, 32.1, 32.4, 42.8, 45.2, 45.2, 46.2, 49.0, 57.1, 57.1, 61.6, 61.6, 78.0, 79.1, 109.6, 110.1, 110.1, 117.8, 118.0, 119.3, 122.0, 125.4, 127.5, 127.7, 133.5, 135.7, 135.7, 135.8, 149.7, 167.3, 167.4, 169.0, 169.2

HRMS (ESI)**:** calc. for $[M+H]^+$ $C_{26}H_{29}N_3O_4ClS$: 514.15618, found: 514.15692; calc. for $[M+H]^+$ $C_{26}H_{29}N_3O_4{}^{37}ClS$: 516.15323, found: 516.15420

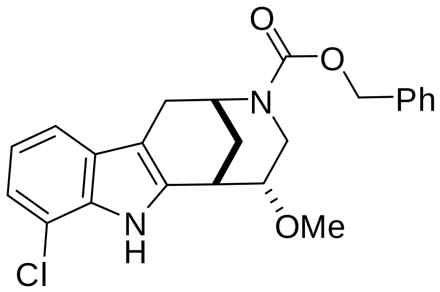

(±)-Benzyl 8-chloro-5-methoxy-1,2,4,5,6,7-hexahydro-3H-2,6-methanoazocino[5,4-b]indole-3-carboxylate (3h):

Compound **3h** (0.220 g, 48%) was synthesized from **2a** following General Procedure **A**, using 4-n-2-Chlorophenylhydrazine hydrochloride (CAS: 41052-75-9).

^{1}H NMR (CDCl3, 400MHz) (rotameric mixture 1:1): δ 2.04 (d, *J*=12.1 Hz, 2H), 2.60 (quin, *J*=11.4 Hz, 1H), 2.86 (t, *J*=17.9 Hz, 1H), 3.12 (dt, *J*=16.4, 4.8 Hz, 1H), 3.38-3.44 (m, 4H), 3.57 (bs, 1H), 4.22 (dd, *J*=12.0, 4.7 Hz, 0.5H), 4.39 (dd, *J*=12.5, 4,9 Hz, 0.5H), 4.81 (bs, 0.5H), 4.90 (bs, 0.5H), 5.26 (d, *J*=18.1 Hz, 2H), 7.06 (t, *J*=7.7 Hz, 1H), 7.19 (d, *J*=7.5 Hz, 1H), 7.30-7.47 (m, 6H), 8.38 (bs, 0.5H), 8.42 (bs, 0.5H).

^{13}C NMR (CDCl3, 100MHz): δ 27.7, 28.1, 29.9, 30.3, 32.4, 32.7, 42.0, 42.1, 45.0, 56.7, 56.8, 67.2, 67.4, 77.3, 77.7, 110.0, 110.3, 116.2, 116.3, 116.5, 116.6, 120.0, 120.8, 127.8, 127.9, 128.0, 128.1,128.3, 128.4, 128.5, 128.6, 133.2, 133.5, 133.6, 136.7, 136.8, 155.5

HRMS (ESI)**:** calc. for $[M+H]^+$ $C_{23}H_{24}N_2O_3Cl$: 411.14700, found: 411.14752. Calc. for $[M+H]^+$ $C_{23}H_{24}N_2O_3{}^{37}Cl$: 413.14405, found: 413.14436

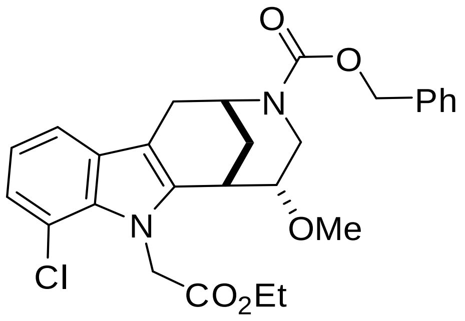

(±)-Benzyl 8-chloro-7-(2-ethoxy-2-oxoethyl)-5-methoxy-1,2,4,5,6,7-hexahydro-3H-2,6-methanoazocino[5,4-b]indole-3-carboxylate (racemic mixure) (4h):

Compound **4h** (0.155 g, 72%) was synthesized from **3h** following General Procedure **B**. Compound **4h** was isolated with a small impurity of the ethyl 2-bromoacetate, as an orange oil.

^{1}H NMR (CDCl3, 400MHz) (rotameric mixture 1:1): δ 1.25 (t, *J*=7.1 Hz, 3H), 1.93-2.06 (m, 2H), 2.56-2.68 (m, 1H), 2.79 (t, *J*=17.2 Hz, 1H), 3.08-3.16 (m, 1H), 3.36 (d, *J*=7.7 Hz, 4H), 3.57 (m, 1H), 4.13-4.22 (m, 2.5H), 4.35 (dd, *J*=12.6, 5.0 Hz, 0.5H), 4.79 (bs, 0.5H), 4.86 (bs, 0.5H), 5.21 (d, *J*=14.5 Hz, 2H), 5.33 (dd, *J*=18.4, 9.3 Hz, 1H), 5.48 (bs, 1H), 6.99 (t, *J*=7.7 Hz, 1H), 7.12 (dd, *J*=7.6, 0.6 Hz, 1H), 7.32-7.43 (m, 6H).

^{13}C NMR (CDCl3, 100MHz): δ 14.2, 27.6, 27.9, 30.5, 30.8, 31.4, 31.5, 41.4, 41.5, 44.9, 46.6, 56.7, 56.8, 60.9, 6.1, 67.2, 67.4, 68.1, 78.1, 78.5, 110.0, 110.3, 116.3, 116.7, 116.8, 120.0, 123.3, 127.8, 127.9, 128.0, 128.1, 128.5, 128.5, 129.6, 129.6, 132.1, 135.6, 135.7, 136.7, 155.4, 169.6, 169.8, 169.9

HRMS (ESI)**:** calc. for $[M+H]^+$ $C_{27}H_{30}N_2O_5Cl$: 497.18378, found: 497.18457; calc. for $[M+H]^+$ $C_{27}H_{30}N_2O_5{}^{37}Cl$: 499.18083, found: 499.18175

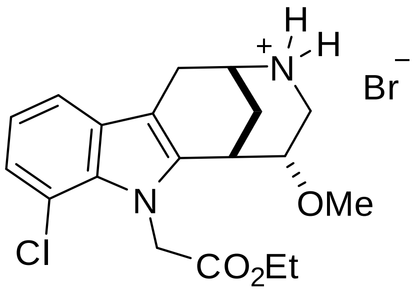

(±)-8-chloro-7-(2-ethoxy-2-oxoethyl)-5-methoxy-2,3,4,5,6,7-hexahydro-1H-2,6-methanoazocino[5,4-b]indol-3-ium bromide (12h):

Compound **12h** (0.150 g, 65%) was synthesized from **4h** following General Procedure **G**. The crude product was purified by chromatography (5 to 10% MeOH/CH_2Cl_2).

^{1}H NMR (MeOD, 400MHz): δ 1.23 (t, *J*=7.1 Hz, 3H), 2.11 (ddd, *J*=14.1, 3.9, 2.1 Hz, 1H), 2.36 (dt, *J*=14.2, 2.8 Hz, 1H), 2.61 (t, *J*=11.7 Hz, 1H), 3.02 (d, *J*=18.0 Hz, 1H), 3.26 (d, *J*=6.2 Hz, 1H), 3.37-3.42 (m, 4H), 3.62-3.63 (m, 1H), 3.90 (dt, *J*=11.2, 4.3 Hz, 1H), 4.09-4.10 (m, 1H), 4.14-4.21 (m, 2H), 5.31 (bs, 2H), 7.02 (t, *J*=7.7 Hz, 1H), 7.12 (dd, *J*=7.7, 0.8 Hz, 1H), 7.41 (dd, *J*=7.8, 0.8 Hz, 1H).

^{13}C NMR (MeOD, 100MHz): δ 14.5, 25.4, 29.4, 31.3, 41.0, 47.8, 48.0, 57.9, 62.5, 77.1, 109.8, 117.5, 118.0, 121.5, 124.8, 130.6, 133.9, 136.7, 171.5

HRMS (ESI)**:** calc. for $[M+H]^+$ $C_{19}H_{24}N_2O_3Cl$: 363.14700, found: 363.14784; calc. for $[M+H]^+$ $C_{19}H_{24}N_2O_3{}^{37}Cl$: 365.14405, found: 365.14485

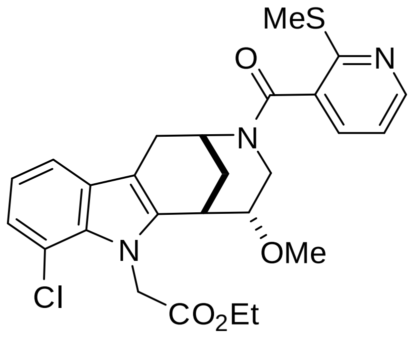

(±)-Ethyl 2-(8-chloro-5-methoxy-3-(2-(methylthio)nicotinoyl)-1,2,3,4,5,6-hexahydro-7H-2,6-methanoazocino[5,4-b]indol-7-yl)acetate (5h):

Compound **5h** (0.110 mg, 43%) was synthesized from **2a** following General Procedure **E**, and was purified y flash chromatography (5 to 50% E.A./CHCl3).

^{1}H NMR (CDCl3, 600MHz) (rotameric mixture 1:1.5): δ 1.14-1.20 (m, 3H), 1.23 (bs, 1H), 1.80 (bs, 0.5H), 1.92 (d, *J*=12.9 Hz, 0.5H), 2.06 (d, *J*=12.6 Hz, 0.5H), 2.12 (bs, 0.5H), 2.31-2.42 (m, 1H), 2.56 (s, 2H), 2.66 (bs, 0.5H), 2.77-2.80 (m, 1H), 2.89-2.99 (m, 0.5H), 3.12 (s, 1H), 3.16-3.19 (m, 1H), 3.36 (m, 2H), 3.62 (d, *J*=15.6 Hz, 2H), 3.86 (s, 0.5H), 4.08-4.18 (m, 2H), 4.64 (dd, *J*=12.7, 4.9 Hz, 0.5H), 5.19-5.33 (m, 2H), 7.01 (dt, *J*=11.3, 7.7 Hz, 1H), 7.11 (dd, *J*=7.5, 2.5 Hz, 1H), 7.20 (bs, 0.4H), 7.26 (dd, *J*=7.4, 4.9 Hz, 0.6H), 7.37 (d, *J*=7.7 Hz, 0.6H), 7.45 (d, *J*=7.7 Hz, 0.4H), 7.64 (bs, 0.4H), 7.76 (d, *J*=6.7 Hz, 0.6H), 8.50 (dd, *J*=4.9, 1.7 Hz, 0.4H), 8.55 (dd, *J*=4.9, 1.7 Hz, 0.6H).

^{13}C NMR (CDCl3, 100MHz): δ 12.4, 12.6, 14.1, 14.1, 27.3, 29.6, 30.3, 30.6, 40.1, 41.7, 46.3, 48.2, 56.6, 60.8, 77.3, 109.4, 109.7, 115.4, 117.1, 117.2, 119.5, 119.6, 120.1, 120.1, 122.8, 129.2, 129.4, 130.9, 131.3, 131.6, 133.9, 136.5, 149.4, 149.5, 154.5, 154.6, 165.9, 169.2, 169.3

HRMS (ESI)**:** calc. for $[M+H]^+$ $C_{26}H_{29}N_3O_4ClS$: 514.15618, found: 514.15706; calc. for $[M+H]^+$ $C_{26}H_{29}N_3O_4{}^{37}ClS$: 516.15323, found: 516.15425

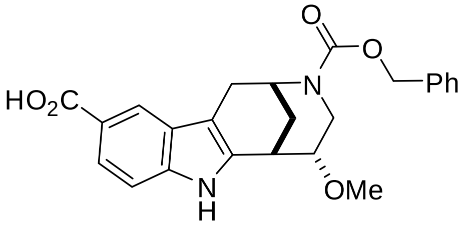

(±)-3-((benzyloxy)carbonyl)-5-methoxy-2,3,4,5,6,7-hexahydro-1H-2,6-methanoazocino[5,4-b]indole-10-carboxylic acid (3i):

To a solution of compound **2a** (0.250 g, 0.824 mmol) in acetic acid (5.5 mL, 0.15 M) 4-hydrazinobenzoic acid (0.125 g, 0.824 mmol) (619-67-0) was added. The reaction was allowed to stir under reflux for 2 h, water and CH_2Cl_2 were then added and the aqueous phase was repeatedly extracted with CH2Cl2 for several times. Solvents were evaporated and the crude product was purified by chromatography (1 to 100% E.A./CH_2Cl_2) to yield compound **3i** (0.240 g, 69%) as a very pasty solid. The purified product showed high purity when checked by HPLC but the NMR Spectra obtained was very complicated. The product was used further as obtained after chromatography.

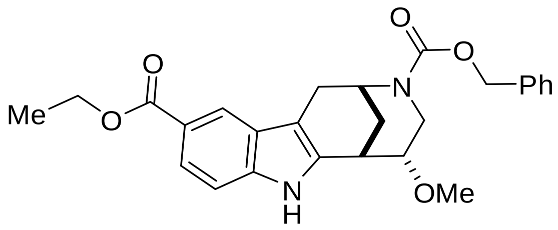

(±)-3-benzyl 10-ethyl-7-(2-ethoxy-2-oxoethyl)-5-methoxy-1,2,4,5,6,7-hexahydro-3H-2,6-methanoazocino[5,4-b]indole-3,10-dicarboxylate (3i´):

To a solution of compound **3i** (0.093 g, 0.221 mmol) in ethanol (2.2 mL, 0.1 M), sulfuric acid (16 µL, 0.014 M) was added. The reaction was allowed to stir at room temperature for 3 h before quenching it with sodium bicarbonate (saturated aqueous solution). The aqueous phase was then extracted with CH_2Cl_2 and the solvents were evaporated. Crude product was purified by chromatography (1 to 100% E.A./CH_2Cl_2) to yield compound **3i´** (65 mg, 65%). Compound was further used in the synthesis without characterization.

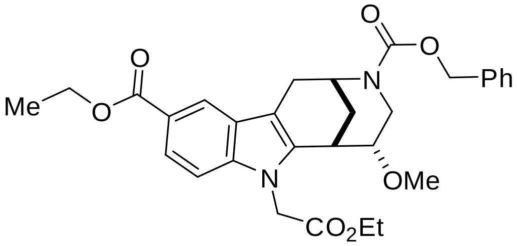

(±)-3-benzyl 10-ethyl 7-(2-ethoxy-2-oxoethyl)-5-methoxy-1,2,4,5,6,7-hexahydro-3H-2,6-methanoazocino[5,4-b]indole-3,10-dicarboxylate (4i):

Compound **4i** (26 mg, 41%) was synthesized from **3i´** following General Procedure **B**.

^{1}H NMR (CDCl3, 500MHz) (rotameric mixture 1:1): δ 1.29 (t, *J*=7.2 Hz, 3H), 1.41 (t, *J*=7.1 Hz, 3H), 1.96-2.07 (m, 2H), 2.54 (td, *J*=30.3, 11.8 Hz, 1H), 2.85 (t, *J*=17.4 Hz, 1H), 3.13-3.20 (m, 1H), 3.34 (d, *J*=6.9 Hz, 3H), 3.38 (s, 1H), 3.53-3.59 (m, 1H), 4.11-4.19 (m, 2H), 4.26-4.34 (m, 1H), 4.35-4.43 (m, 2H), 4.77 (bs, 0.5H), 4.79 (s, 0.5H), 4.83 (s, 0.5H), 4.84 (bs, 0.5H), 5.07-5.19 (m, 3H), 7.19 (d, *J*=8.7 Hz, 1H), 7.30-7.42 (m, 5H), 7.90 (d, *J*=8.6 Hz, 1H), 8.22 (d, *J*=6.2 Hz, 1H).

^{13}C NMR (CDCl3, 100MHz): δ 14.3, 14.3, 14.6, 27.8, 28.1, 30.4, 30.8, 31.8, 31.9, 41.6, 41.7, 45.0, 45.1, 56.9, 57.0, 60.6, 61.2, 61.6, 67.4, 67.6, 68.3., 78.1, 78.5, 108.6, 110.1, 111.4, 121.0, 121.1, 121.8, 123.2, 126.3, 126.3, 128.0, 128.1, 128.1, 128.3, 128.6, 128.7, 135.5, 135.6, 136.8, 139.8, 155.5, 155.6, 167.9, 169.0, 169.1, 169.8

HRMS (ESI)**:** calc. for $[M+H]^+$ $C_{30}H_{35}N_2O_7$: 535.24388, found: 535.24388

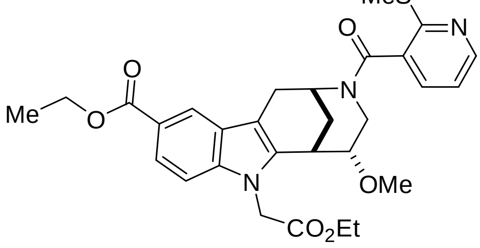

Ethyl 7-(2-ethoxy-2-oxoethyl)-5-methoxy-3-(2-(methylthio)nicotinoyl)-2,3,4,5,6,7-hexahydro-1H-2,6-methanoazocino[5,4-b]indole-10-carboxylate (5i):

Compound **5i** (0.011 g, 42%) was obtained from **4i** following General Procedures **D** and **E**, without the need to purify the crude product after the Cbz deprotection.

^{1}H NMR (CDCl3, 500 MHz) (rotameric mixture 1:1): δ 1.24 (dt, *J*=8.9, 7.1 Hz, 3H), 1.42 (dd, *J*=13.2, 7.0 Hz, 3H), 1.95 (bs, 0.5H), 2.07-2.34 (m, 1H), 2.56-2.79 (m, 4H), 2.87-3.11 (m, 1H), 3.18 (s, 1H), 3.31-3.37 (m, 1H), 3.42 (s, 1.5 H), 3.46 (d, *J*=3.2 Hz, 1H), 3.70 (bs, 0.5H), 3.92-4.02 (m, 2.5H), 4.11-4.24 (m, 2H), 4.40 (quin, *J*=6.9 Hz, 2H), 4.82 (t, *J*=17.9 Hz, 1H), 4.91 (dd, *J*=12.9, 4.9 Hz, 0.5H), 5.04 (d, *J*=18.0 Hz, 0.5H), 5.15 (d, *J*=18.1 Hz, 0.5H), 5.45 (bs, 0.5H), 7.07 (dd, *J*=7.3, 5.1 Hz, 0.5H), 7.15 (bs, 0.5H), 7.20 (dd, *J*=8.6, 6.0 Hz, 1H), 7.43-7.50 (m, 1H), 7.91 (dd, *J*=8.6, 1.5 Hz, 1H), 8.21 (bs, 0.5H), 8.27 (bs, 0.5H), 8.49 (dd, *J*=4.9, 1.7 Hz, 0.5H), 8.56 (dd, *J*=5.0, 1.7 Hz, 0.5H).

^{13}C NMR (CDCl3, 100MHz): δ 13.2, 13.4, 14.3, 14.6, 28.4, 30.1, 31.4, 32.0, 32.3, 39.3, 42.9, 45.1, 45.2, 49.0, 57.1, 60.8, 60.8, 61.7, 61.7, 77.9, 78.9, 108.8, 119.4, 121.0, 121.2, 121.9, 121.9, 123.4, 126.1, 126.2, 133.8, 135.4, 135.7, 139.8, 149.7, 167.4, 167.9, 168.0, 168.1, 169.1

HRMS (ESI)**:** calc. for $[M+H]^+$ $C_{29}H_{34}N_3O_6S$: 552.21628, found: 552.21580; Calc. for $[M+Na]^+$ $C_{29}H_{33}N_3O_6NaS$: 574.19823, found: 574.19767

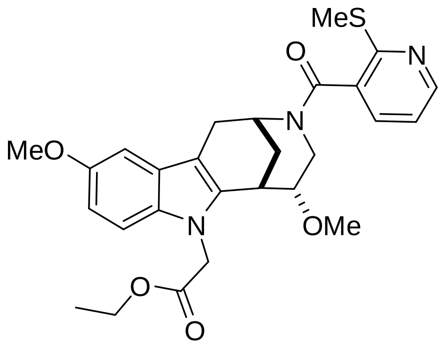

(±)-Ethyl 2-(5,10-dimethoxy-3-(2-(methylthio)nicotinoyl)-1,2,3,4,5,6-hexahydro-7H-2,6-methanoazocino[5,4-b]indol-7-yl)acetate (5aa):

^{1}H NMR (CDCl3, 500MHz): δ 1.22-1.33 (m, 5H), 1.96 (m, 2H), 2.12 (d, *J*=11.2 Hz, 1H), 2.16-2.24 (m, 1H), 2.57-2.69 (m, 3H), 2.71 (s, 3H), 2.98-3.10 (m, 2H), 3.15-3.25 (m, 2H), 3.30 (dd, *J*=17.2, 5.9 Hz, 1H), 3.34 (dd, *J*=12.8, 4.4 Hz, 1H), 3.43 (s, 2H), 3.44-3.48 (m, 2H), 3.66-3.73 (m, 1H), 3.87 (s, 2H), 3.89 (s, 2H), 4.01 (s, 1H), 4.11-4.26 (m, 4H), 4.75 (d, *J*=18.4 Hz, 1H), 4.78 (d, *J*=18.4, 1H), 4.92 (dd, *J*=4.7 and 13.1 Hz, 1H), 5.00 (d, *J*=17.9 Hz, 1H), 5.12 (d, *J*=17.9 Hz, 1H), 5.41-5.47 (m, 1H), 6.87 (d, *J*=8.7 Hz, 2H), 6.89-6.94 (m, 1H), 6.96-7.02 (m, 1H), 7.07-7.15 (m, 2H), 7.15-7.20 (m, 1H), 7.41-7.50 (m, 1H), 7.51-7.58 (m, 1H), 8.52 (d, *J*=4.5 Hz, 1H), 8.57 (d, *J*=4.5 Hz, 1H).

^{13}C NMR (CDCl3, 100MHz): δ 14.2, 22.7, 31.4, 42.7, 44.9, 49.0, 56.0, 56.8, 61.3, 109.6, 111.4, 119.2, 126.4, 126.8, 132.2, 133.8, 134.3, 134.7, 148.9, 154.2, , 166.5, 169.2, 169.4.

HRMS (ESI) (ESI): calc. for $[M+H]^+$ $C_{27}H_{32}N_3O_5S$: 510.2057; Found: 510.2062.

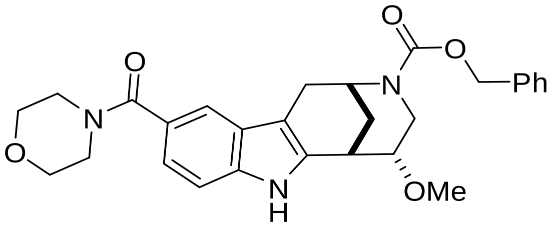

(±)-Benzyl 5-methoxy-10-(morpholine-4-carbonyl)-1,2,4,5,6,7-hexahydro-3H-2,6-methanoazocino[5,4-b]indole-3-carboxylate (3ab´):

Compound **3i** (0.091 g, 0.216 mmol), TBTU (0.111 g, 0.346 mmol) (CAS number: 125700-67-6) and DIPEA (60.3 µL, 0.346 mmol) were dissolved in DMF (3.6 mL, 0.06 M) and allowed to stir at room temperature for 5 min before adding morpholine (18.7 µL, 0.216 mmol). The reaction was allowed to stir further for 1 h and the reaction was partitioned between CH_2Cl_2 and BRINE/water (1:1), the organic phase was washed several time with this mixture and the organic solvents were removed. The crude product was purified by chromatography (1 to 3% MeOH/CH_2Cl_2) to obtain compound **3ab** (87 mg, 82%) with a

minor impurity (most probably the *syn* diasteroisomer). For analytical purposes some of the product was further purified by preparative HPLC.

^{1}H NMR (CDCl3, 600MHz) (rotameric mixture 1:1): δ 1.99-2.09 (m, 2H), 2.44 (dd, *J*=12.2, 11.0 Hz, 0.5H), 2.49-2.52 (m, 0.5H), 2.79 (dd, *J*=29.3, 16.8 Hz, 1H), 3.08 (dd, *J*=17.0, 5.2 Hz, 1H), 3.34 (bs, 0.5H), 3.36 (bs, 0.5H), 3.40 (s, 1H), 3.41 (s, 1H), 3.53-3.58 (m, 1H), 3.72-3.91 (m, 9H), 4.13 (dd, *J*=12.6, 5.3 Hz, 0.5H), 4.28 (dd, *J*=12.6, 5.3 Hz, 0.5H), 4.75 (bs, 0.5H), 4.82 (bs, 0.5H), 5.15 (s, 1H), 5.19 (d, *J*=3.1 Hz, 1H), 7.18-7.19 (m, 1H), 7.29-7.41 (m, 6H), 7.55 (d, *J*=6.2 Hz, 1H), 8.16 (bs, 0.5H), 8.12 (bs, 0.5H).

^{13}C NMR (CDCl3, 100MHz): δ 27.6, 28.0, 30.0, 30.3, 32.6, 32.9, 42.3, 45.2, 45.3, 53.6, 57.0, 57.0, 67.1, 67.5, 67.7, 77.5, 77.9, 109.9, 110.2, 111.0, 111.0, 118.0, 118.1, 120.8, 120.9, 125.1, 125.8, 126.6, 126.6, 128.1, 128.1, 128.2 ,128.3, 128.7, 128.8, 134.3, 134.4, 136.7, 136.7, 137.0, 155.8, 155.8, 173.0, 173.1

ESI: calc. [M+H$^+$]: $C_{28}H_{32}N_3O_5$: 490.2, found 490.2.

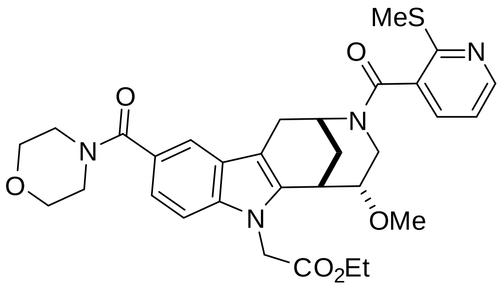

(±)-Ethyl 2-(5-methoxy-3-(2-(methylthio)nicotinoyl)-10-(morpholine-4-carbonyl)-1,2,3,4,5,6-hexahydro-7H-2,6-methanoazocino[5,4-b]indol-7-yl)acetate (5ab):

Compound **5ab** (8 mg, 47 %) was synthesized from **3ab´** following General Procedures **B**, **D** and **E** without the need for purification of the intermediate crude products.

^{1}H NMR (CDCl3, 500 MHz) (rotameric mixture 1:1): δ 1.25 (dd, *J*= 13.3, 7.1 Hz, 3H), 1.93-2.20 (m, 1.5H), 2.48-2.58 (m, 5H), 2.63 (s, 2H), 2.89-3.17 (m, 2.5H), 3.33 (ddd, *J*=23.4, 15.1, 5.4 Hz, 1H), 3.41 (s, 1.5H), 3.45 (d, *J*=2.7 Hz, 1H), 3.71 (bs, 7H), 4.01 (bs, 0.5H), 4.12-4.25 (m, 2H), 4.80 (t, *J*=18.0 Hz,

1H), 4.91 (dd, *J*=12.9, 5.0 Hz, 0.5H), 5.04 (d,*J*=18.0 Hz, 0.5H), 5.15 (d, *J*=18.1 Hz, 0.5H), 5.44 (bs, 0.5H), 7.05 (dd, *J*=7.5, 5.0 Hz, 0.5H), 7.12 (m, 0.5H), 7.19-7.25 (m, 2H), 7.41-7.47 (m, 1H), 7.58 (bs, 0.5H), 7.63 (bs, 0.5H); 8.47 (dd, *J*=4.9, 1.7 Hz, 0.5H), 8.53 (dd, *J*=4.9, 1.7 Hz, 0.5H).

HRMS (ESI)**:** calc. for $[M+H]^+$ $C_{31}H_{37}N_4O_6S$: 593.24283, found: 593.24252; calc. for $[M+Na]^+$ $C_{31}H_{36}N_4O_6NaS$: 615.22478, found: 615.22452

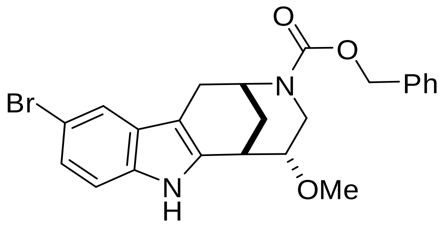

(±)-Benzyl 10-bromo-5-methoxy-1,2,4,5,6,7-hexahydro-3H-2,6-methanoazocino[5,4-b]indole-3-carboxylate (3ac):

Compound **3ac** (0.378 g, 84%) was synthesised from compound **2a** following general procedure **B**, using 4-bromophenylhydrazine hydrochloride (CAS: 622-88-8).

^{1}H NMR (CDCl3, 400MHz) (rotameric mixture 1:1): δ 1.96-2.09 (m, 2H), 2.47 (dt, *J*=23.4, 11.8 Hz, 1H), 2.75 (t, *J*=17.8 Hz, 1H), 3.04 (dt, *J*=16.6, 6.6 Hz, 1H), 3.31-3.34 (m, 1H), 3.39 (d, *J*=3.5 Hz, 3H), 3.54 (td, *J*=10.1, 5.6 Hz, 1H), 4.13 (dd, *J*=12.8, 5.6 Hz, 0.5H), 4.13 (dd, *J*=12.6, 5.3 Hz, 0.5H), 4.74 (bs, 0.5H), 4.82 (bs, 0.5H), 5.18 (d, *J*=18.5 Hz, 2H), 7.16 (d, *J*=8.6 Hz, 1H), 7.22 (dd, *J*=8.6, 1.8 Hz, 1H), 7.31-7.41 (m, 5H), 7.57 (d, *J*=5.8 Hz, 1H), 8.00 (d, *J*=12.9 Hz, 1H).

^{13}C NMR (CDCl3, 100MHz): δ 27.6, 27.0, 30.0, 30.4, 32.6, 33.0, 42.3, 42.3, 45.2, 57.0, 57.0, 67.4, 67.6, 77.4, 77.5, 77.9, 108.9, 109.2, 112.4, 112.7, 120.7, 120.8, 124.3, 128.0, 128.1, 128.2, 128.3, 128.6, 128.7, 134.2, 134.3, 134.7, 136.8, 136.8, 155.6

ESI: calc. $[M+H^+]$: $C_{23}H_{24}N_2O_3Br$: 455.0, found 455.0; calc. $[M+H^+]$: $C_{23}H_{24}N_2O_3{}^{81}Br$: 457.0, found 457.0

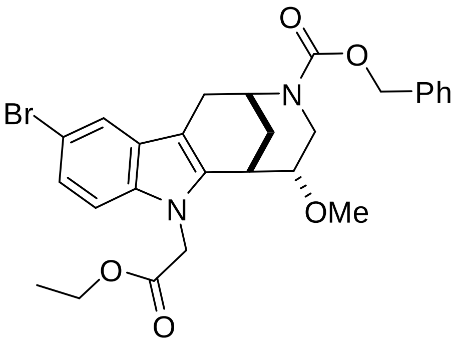

(±)-Benzyl 10-bromo-7-(2-ethoxy-2-oxoethyl)-5-methoxy-1,2,4,5,6,7-hexahydro-3H-2,6-methanoazocino[5,4-b]indole-3-carboxylate (4ac):

Compound **4ac** (0.240 g, 84%) was synthesized from **3ac** following General Procedure **B**. Compound **4ac** was isolated with a small impurity of the ethyl 2-bromoacetate, as an orange oil.

^{1}H NMR (CDCl3, 400MHz) (rotameric mixture 1:1): δ 1.25 (t, *J*=7.1 Hz, 3H), 1.94-2.05 (m, 2H), 2.51-2.63 (m, 1H), 2.77 (t, *J*=17.0 Hz, 1H), 3.05-3.13 (m, 1H), 3.35 (d, *J*=4.8 Hz, 3H), 3.39 (d, *J*=2.8 Hz, 1H), 3.54-3.59 (m, 1H), 4.12-4.27 (m, 2.5H), 4.34 (dd, *J*=12.5, 4.9 Hz, 0.5H), 4.76-4.80 (m, 1.5H), 4.85 (bs, 0.5H), 5.09 (t, *J*=19.5 Hz, 1H), 5.21 (d,*J*=14.8 Hz, 2H), 7.07 (d, *J*=8.7 Hz, 1H), 7.26 (dd, *J*=8.6, 1.9 Hz, 1H), 7.33-7.43 (m, 5H), 7.59 (d, *J*=3.7 Hz, 1H).

^{13}C NMR (CDCl3, 100MHz): δ 14.0, 14.1, 27.5, 27.8, 30.2, 30.5, 31.6, 31.7, 41.5, 41.6, 44.8, 44.9, 56.7, 56.8, 60.9, 61.3, 67.1, 67., 368.1, 77.4, 78.0, 78.3, 109.0, 109.3, 110.4, 112.5, 120.6, 124.0, 127.8, 127.8, 127.9, 128.0, 128.1, 128.2, 128.4, 128.5, 135.3, 135.4, 135.8, 136.7, 155.3, 168.7, 168.8

HRMS (ESI)**:** calc. for $[M+H]^+$ $C_{27}H_{30}N_2O_5Br$: 541.13326, found: 541.13476. Calc. for $[M+H]^+$ $C_{27}H_{30}N_2O_5{}^{81}Br$: 543.13121, found: 543.13278

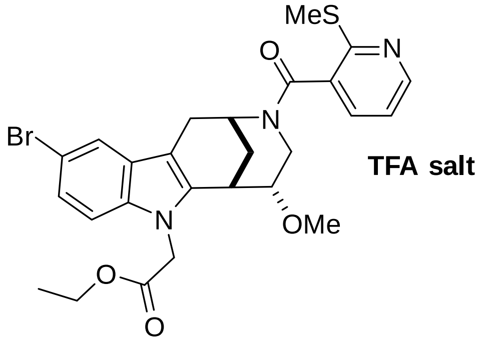

(±)-Ethyl 2-(10-bromo-5-methoxy-3-(2-(methylthio)nicotinoyl)-1,2,3,4,5,6-hexahydro-7H-2,6-methanoazocino[5,4-b]indol-7-yl)acetate (5ac):

Compound **5ac** (30.9 mg, 60%) was synthesized from **4ac** following General Procedures **D** and **E**, without the need to purified the intermediate crude product. The pure enantiomers were obtained following the same procedure as for compond 5a (Glupin) (see section: Separation of (±)-Glupin racemic mixture).

^{1}H NMR (CDCl3, 400MHz) (rotameric mixture 1:1.3): δ 1.24 (q, *J*=7.1 Hz, 3H), 1.94 (d, *J*=11.0 Hz, 0.5H), 2.08-2.32 (m, 1.5H), 2.60 (bs, 2H), 2.66 (s, 2H), 2.83-3.03 (m, 1.5H), 3.17 (s, 1.5H), 3.30 (ddd, *J*=23.4, 15.1, 5.4 Hz, 1H), 3.41 (s, 1.5H), 3.45 (d, *J*=3.5 Hz, 1H), 3.68-3.82 (m, 1H), 4.00 (s, 0.5H), 4.09-4.25 (m, 2H), 4.75 (dd, *J*=18.0, 13.7 Hz, 1H), 4.90 (dd, *J*=12.9, 4.9 Hz, 0.5H), 4.99 (d, *J*=180 Hz, 0.5H), 5.11 (d, *J*=18.0 Hz, 0.5H), 5.42 (bs, 0.5H), 7.05-7.10 (m, 1.5H), 7.15 (dd, *J*=7.2, 5.1 Hz, 0.5H), 7.26-7.28 (m, 1H), 7.46-7.51 (m, 1H), 7.57 (s, 0.5H), 7.63 (s, 0.5H), 7.83 (bs, 1H), 8.50 (dd, *J*=5.0, 1.7 Hz, 0.5H), 8.57 (dd, *J*=5.0, 1.7 Hz, 0.5H).

^{13}C NMR (CDCl3, 100MHz): δ 13.3, 13.5, 14.3, 28.3, 30.1, 31.4, 32.0, 32.3, 39.4, 43.1, 45.1, 45.1, 49.2, 57.1, 61.4, 61.7, 77.9, 79.0, 110.6, 110.6, 113.0, 119.5, 120.8, 121.0, 124.6, 128.1, 128.3, 133.9, 135.2, 135.5, 136.0, 136.0, 149.6, 167.4, 167.5, 169.0, 169.1

HRMS (ESI)**:** calc. for $[M+H]^+$ $C_{26}H_{29}N_3O_4BrS$:558.10567, found: 558.10757; calc. for $[M+H]^+$ $C_{26}H_{29}N_3O_4{}^{81}BrS$:560.10362, found: 560.10521

Specific optical rotations (as the TFA salt): (+)-**5ac**$[\alpha]_D$= - 14.8°; (-)-**5ac**$[\alpha]_D$= +20.3°

R3

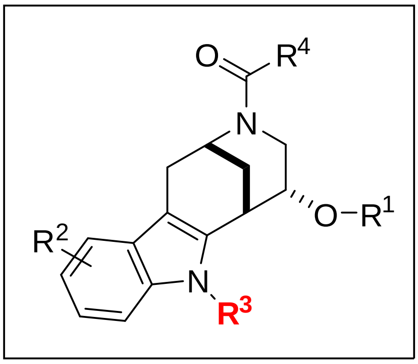

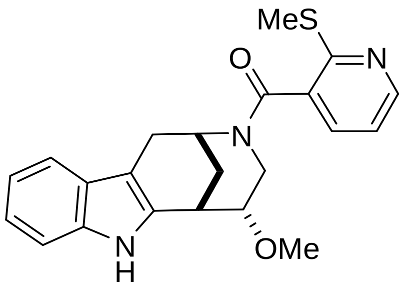

(±)- (5-methoxy-1,2,4,5,6,7-hexahydro-3H-2,6-methanoazocino[5,4-b]indol-3-yl)(2-(methylthio)pyridin-3-yl)methanone (5j):

Compound **5j** (92 mg, 81%) was synthesized from **3a** following General Procedure **F**. Compound 5j was isolated as a white foamy solid.

HRMS (ESI)**:** calc. for $[M+H]^+$ $C_{22}H_{24}N_3O_2S$: 394.15837, found: 394.15833; calc. for $[M+Na]^+$ $C_{22}H_{23}N_3O_2NaS$: 416.14032, found: 416.14028

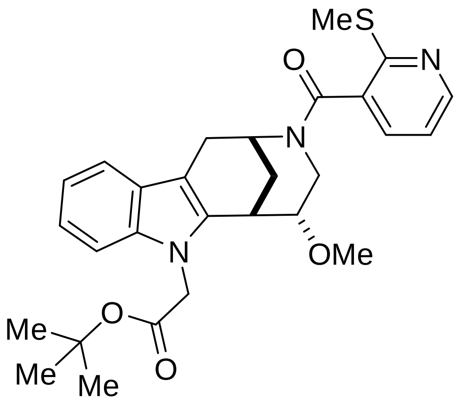

(±)-tert-butyl 2-(5-methoxy-3-(2-(methylthio)nicotinoyl)-3,4,5,6-tetrahydro-1H-2,6-methanoazocino[5,4-b]indol-7(2H)-yl)acetate (5k):

Compound **5k** was synthesized from **2a** following General Procedures **C** and **F**. The compound was purified by preparative HPLC eluting with 10-100% MeCN in H_2O.

^{1}H NMR (CDCl3, 500MHz): δ 1.34 (s, 18H), 1.88 (s, 2H), 2.04-2.37 (m, 4H), 2.40-2.54 (m, 4H), 2.56 (s, 3H), 2.62-3.03 (m, 5H), 3.12 (s, 2H), 3.20-3.30 (m, 2H), 3.33-3.42 (m, 4H), 3.62 (s, 1H), 3.93 (s, 1H), 4.62 (app t, *J*= 17.6 Hz, 2H), 4.80-4.87 (m, 2H), 4.95 (d, *J*=17.6, 1H), 5.36 (m, 1H), 6.91-6.98 (m, 2H), 6.99-7.06 (m, 3H), 7.08-7.15 (m, 3H), 7.31-7.49 (m, 4H), 8.38 (d, *J*=4.7 Hz, 1H), 8.45 (d, *J*=4.7 Hz, 1H).

^{13}C NMR (CDCl3, 100MHz): δ 13.1, 27.9, 31.5, 32.2, 42.8, 45.7, 56.9, 78.9, 79.3, 82.0, 108.9, 119.3, 121.6, 126.3, 126.5, 149.5, 166.9, 168.4, 168.6.

HRMS (ESI): calc. for $[M+H]^+$ $C_{28}H_{34}N_3O_4S$: 508.2265; Found: 508.2260.

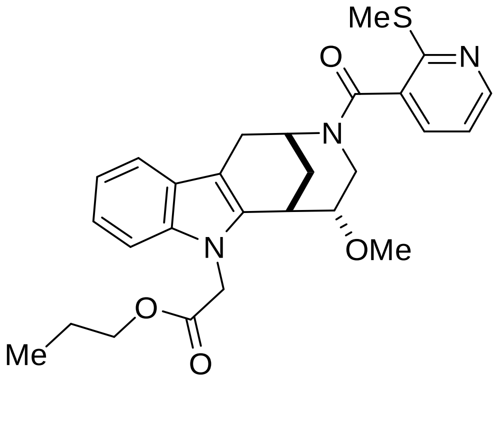

(±)-Propyl 2-(-5-methoxy-3-(2-(methylthio)nicotinoyl)-3,4,5,6-tetrahydro-1H-2,6-methanoazocino[5,4-b]indol-7(2H)-yl)acetate (5l):

Compound **5l** was synthesized from **2a** following General Procedures **C** and **F**. The compound was purified by preparative HPLC eluting with 10-100% MeCN in H_2O.

^{1}H NMR (CDCl3, 500MHz): δ 0.83-0.92 (m, 6H), 1.58-1.67 (m, 5H), 1.97 (app. d, *J*=10.9 Hz, 2H), 2.10-2.16 (m, 2H), 2.54-2.66 (m, 4H), 2.68 (s, 4H), 3.20 (app. s, 3H), 3.30-3.38 (m, 3H), 3.43 (s, 3H), 3.49 (s, 3H), 3.66-3.76 (m, 1H), 3.99-4.08 (m, 4H), 4.08-4.16 (m, 2H), 4.82 (app. dd, *J*=18.1 Hz, 2H), 4.92 (dd, *J*=4.8 and 12.9 Hz, 1H), 5.06 (d, *J*=18.0 Hz, 1H), 5.17 (d, *J*=18.0 Hz, 1H), 5.42-5.47 (m, 1H), 7.04-7.10 (m, 1H), 7.10-7.17 (m, 3H), 7.19-7.25 (m, 4H), 7.39-7.48 (m, 2H), 7.50-7.57 (m, 2H), 8.50 (d, *J*=4.7 Hz, 1H), 8.56 (d, *J*=4.7 Hz, 1H).

^{13}C NMR (CDCl3, 100MHz): δ 10.3, 13.2, 14.1, 21.8, 29.8, 31.5, 31.9, 41.1, 42.8, 44.5, 48.9, 56.8, 66.7, 99.6, 108.9, 117.9, 118.2, 119.2, 121.6, 126.3, 126.5, 133.5, 133.8, 134.0, 137.1, 149.2, 166.7, 166.9, 169.3, 169.5.

HRMS (ESI): calc. for $[M+H]^+$ $C_{27}H_{32}N_3O_4S$: 494.2108; Found: 494.2114.

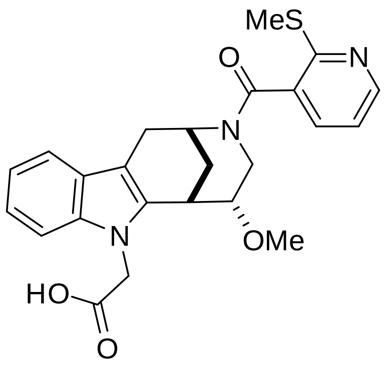

(±)-2-(5-methoxy-3-(2-(methylthio)nicotinoyl)-1,2,3,4,5,6-hexahydro-7H-2,6-methanoazocino[5,4-b]indol-7-yl)acetic acid (5n):

Compound **5a** (5.0 mg, 0.010 mmol) was dissolved in THF (0.14 mL, 0.07 M) and H_20 (60 µL, 0.16M) LiOH (1.5 mg, 0.06 mmol). The reaction was allowed to stir for 1 hour, then the reaction was taken to pH

2 with HCl 1M and extracted with CH_2Cl_2. The crude product was purified by chromatography (due to the amount of crude product the chromatographic column was performed on a Pasteur pipette, 1 to 5% MeOH/CH2Cl2) to obtain product **5n** (2.1 mg, 47%) as a white solid. Due to the lack of material the compound purity was checked by uHPLC (> 95%) and characterized only by HRMS (ESI).

HRMS (ESI)**:** calc. for $[M+H]^+$ $C_{24}H_{26}N_3O_4S$: 452.16385, found: 452.16381

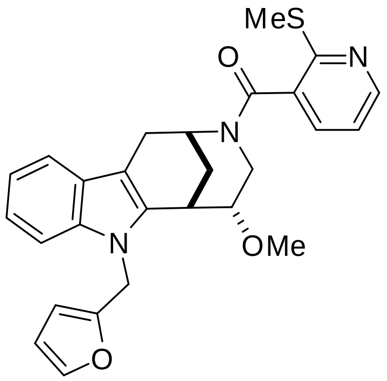

(±)-(7-(furan-2-ylmethyl)-5-methoxy-5,6-dihydro-1H-2,6-methanoazocino[5,4-b]indol-3(2H,4H,7H)-yl)(2-(methylthio)pyridin-3-yl)methanone (5o):

Methane sulfonyl chloride (2.4 mmol, 1.2 equiv.) was added to a stirred solution furfuryl alcohol (2 mmol, 1 equiv.) and Et3N (4 mmol, 2 equiv.) in CH_2Cl_2 (20 mL, 0.1 M) and the reaction mixture was stirred at rt. After 20 h, the reaction mixture was diluted with CH_2Cl_2 (20 mL) and washed with 1 M aq. HCl (20 mL). The aqueous layer was extracted with CH_2Cl_2 (3 × 10 mL) and the combined organic layers were dried over MgSO4, filtered and conc. in vacuo to give a crude. The crude was diluted in MeCN (5 mL, 0.4 M), compound **3a** was added and the resulting reaction mixture was heated to reflux. After 2.5 h the reaction mixture was allowed to cool to rt, and neutralized by the slow addition of sat. aq. NaHCO3 solution. The reaction mixture was extracted with CH_2Cl_2 (3 × 20 mL) and the combined organic layers were dried over MgSO4, filtered and conc. in vacuo to give a crude product. The crude product was submitted to General Procedure **F** affording the desired compound **5o** in 43% yield over 3 steps.

^{1}H NMR (CDCl3, 500MHz): δ 1.13-1.25 (m, 6H), 1.96-2.03 (m, 2H), 2.23-2.31 (m, 2H), 2.55-2.63 (m, 3H), 2.93-3.02 (m, 4H), 3.11-3.19 (m, 2H), 3.20-3.29 (m, 2H), 3.39 (s, 3H), 3.62-3.67 (m, 3H), 3.92 (s, br, 1H), 4.84 (dd, *J*=4.7, 12.9 Hz, 1H), 5.13 (dd, *J*=12.5 and 16.9 Hz, 1H), 5.32-5.37 (m, 2H), 5.40 (d, *J*=16.9 Hz, 1H), 5.52 (d, *J*=16.5 Hz, 1H), 5.98 (d, *J*=2.8 Hz, 1H), 6.02 (d, *J*=2.8 Hz, 1H), 6.18 (s, 2H), 6.97-7.06 (m, 3H), 7.07-7.15 (m, 3H), 7.23 (d, *J*=7.0 Hz, 2H), 7.26-7.33 (m, 3H), 7.24-7.40 (m, 3H), 8.43 (d, *J*=4.7 Hz, 1H), 8.49 (d, *J*=4.7 Hz, 1H).

^{13}C NMR (CDCl3, 100MHz): δ 13.3, 14.1, 29.8, 31.5, 31.9, 40.1, 42.7, 49.2, 57.1, 61.0, 99.6, 107.0, 109.3, 109.5, 110.3, 117.9, 119.1, 121.5, 126.1, 133.6, 133.9, 136.5, 137.6, 141.9, 149.2, 151.5, 166.9.

HRMS (ESI): calc. for $[M+H]^+$ $C_{27}H_{28}N_3O_3S$: 474.1846; Found: 474.1849.

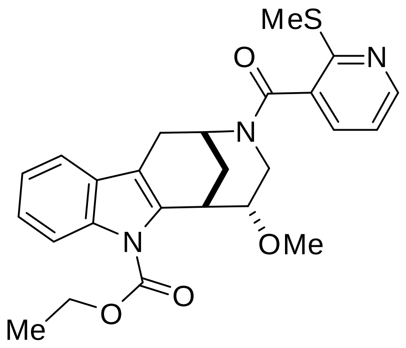

(±)-ethyl 5-methoxy-3-(2-(methylthio)nicotinoyl)-3,4,5,6-tetrahydro-1H-2,6-methanoazocino[5,4-b]indole-7(2H)-carboxylate (5p):

Sodium hydride (as a 60% suspension in mineral oil, 1.5 mmol, 1.5 equiv.) was added to a stirred solution of the indole **3a** (1 mmol) in DMF (2 mL, 0.5 M) cooled at 0 ˚C and the reaction was stirred at 0 ˚C. After 30 min, ethyl chloroformate (1.5 equiv.) was added dropwise and the reaction mixture was allowed to warm to rt. After 3 h, the reaction mixture was cooled to 0 ˚C and the quenched by slow addition of sat. brine (10 mL). The mixture was extracted with EtOAc (3 × 10 mL) and the combined organic layers were

dried over MgSO4, filtered and conc. in vacuo to give a crude which was submitted directly to General Procedure **F**. Following purification the desired compound **5p** was isolated in 36% yield over three steps.

^{1}H NMR (CDCl3, 500MHz): δ 1.43 (q, *J*=7.0 Hz, 6H), 1.90-2.09 (m, 2H), 2.13 (d, *J*=11.2 Hz, 1H), 2.20-2.31 (m, 1H), 2.32-2.60 (m, 4H), 2.62 (s, 3H), 2.65-3.04 (m, 4H), 3.09-3.18 (m, 2H), 3.22 (s, 3H), 3.36 (s, 3H), 3.54-3.65 (m, 2H), 3.86 (s, 1H), 4.34-4.47 (m, 4H), 4.48-4.55 (m, 2H), 4.72 (dd, *J*= 4.9 and 13.4 Hz, 1H), 5.30 (s, 1H), 7.02 (app. t, *J*=6.2 Hz, 1H), 7.09 (app. t, *J*=6.2 Hz, 1H), 7.14-7.25 (m, 3H), 7.26-7.34 (m, 1H), 7.35-7.48 (m, 4H), 7.97 (t, *J*=9.5 Hz, 2H), 8.43 (dd, *J*=1.7 and 5.2 Hz, 1H), 8.49 (dd, *J*=1.7 and 5.2 Hz, 1H).

^{13}C NMR (CDCl3, 100MHz): δ 14.2, 22.2, 31.1, 31.6, 41.9, 48.2, 57.9, 63.4, 78.0, 79.0 109.2, 115.6, 117.6, 117.9, 119.1, 122.6, 124.1 127.8, 128.2, 133.5, 133.8, 136.2, 149.1, 152.1, 155.9, 166.1.

HRMS (ESI): calc. for $[M+H]^+$ $C_{25}H_{28}N_3O_4S$: 466.1795; Found: 466.1800.

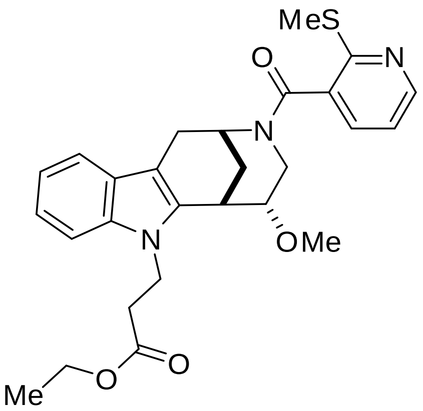

(±)-ethyl 3-(5-methoxy-3-(2-(methylthio)nicotinoyl)-3,4,5,6-tetrahydro-1H-2,6-methanoazocino[5,4-b]indol-7(2H)-yl)propanoate (5q):

By the method of Gingrich *et al.*[4] ethyl acrylate (1.5 mmol, 1.5 equiv.) was added to a stirred solution of the indole (1 mmol, 1 equiv.) and 1,8 diazabicycloundecane (2.5 mmol, 2.5 equiv.) in MeCN (10 mL, 0.1 M) and the reaction mixture was heated to reflux. After 18 h the reaction mixture was allowed to cool to rt and conc. *in vacuo* to give a crude which was submitted directly to General Procedure **F**. Following

purification by preparative HPLC eluting with 10-100% MeCN in H2O, afforded the desired compound **5q** in 52% yield over three steps.

^{1}H NMR (CDCl3, 500MHz): δ 1.10-1.21 (m, 6H), 1.76-1.84 (m, 1H), 1.93-2.00 (m, 1H), 2.12 (d, *J*=11.2 Hz, 1H), 2.16-2.20 (m, 1H), 2.21-2.33 (m, 2H), 2.54-2.57 (s, 6H), 2.61-2.81 (m, 6H), 2.86-3.00 (m, 2H), 3.09-3.20 (m, 2H), 3.21-3.27 (m, 2H), 3.36 (s, 4H), 3.55 (app. t, *J*= 7.0 Hz, 2H), 3.58-3.66 (m, 1H), 3.91 (s, 1H), 4.00-4.10 (m, 4H), 4.21-4.34 (m, 2H), 4.41-4.57 (m, 2H), 4.80 (dd, *J*= 5.2 and 12.6 Hz, 1H), 5.35 (s, 1H), 6.95 (app. t, *J*=6.2 Hz, 2H), 6.99-7.07 (m, 4H), 7.12 (t, *J*=7.6 Hz, 2H), 7.24 (t, *J*=7.2 Hz, 2H), 7.31-7.48 (m, 4H), 8.38 (dd, *J*=1.9 and 4.9 Hz, 1H), 8.44 (dd, *J*=1.7 and 4.9 Hz, 1H).

^{13}C NMR (CDCl3, 100MHz): δ 14.1, 22.6, 31.2, 31.7, 38.8, 42.6, 48.9, 56.9, 60.7, 78.1, 79.2 109.2, 117.9, 118.2,119.0, 121.3, 126.2, 126.5, 131.2, 133.3, 133.8, 136.1, 149.4, 155.8, 166.8, 166.9, 171.7.

HRMS (ESI): calc. for $[M+H]^+$ $C_{27}H_{32}N_3O_4S$: 494.2108; Found: 494.2096.

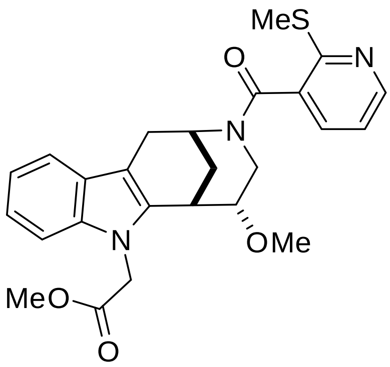

(±)-methyl 2-(5-methoxy-3-(2-(methylthio)nicotinoyl)-3,4,5,6-tetrahydro-1H-2,6-methanoazocino[5,4-b]indol-7(2H)-yl)acetate (5ad):

Compound **5ad** was synthesized from compound **3a** following General Procedure **C**, using methyl bromoacetate, directly followed by General procedure **F**.

^{1}H NMR (CDCl3, 500MHz): δ 1.91-2.00 (m, 1H), 2.10-2.14 (m, 1H), 2.26-2.59 (m, 5H), 2.65 (s, 3H), 2.71-2.79 (m, 2H), 3.00-3.12 (m, 2H), 3.18 (s, 3H), 3.30-3.39 (m, 2H), 3.42 (s, 3H), 3.48 (s, 3H), 3.71 (s,

3H), 3.73 (s, 4H), 4.03 (s, 1H), 4.84 (app. t, *J*=18.0 Hz, 3H), 4.93 (app. dd, *J*= 5.2 and 13.2 Hz, 2H), 5.07 (d, *J*=18.3 Hz, 1H), 5.19 (d, *J*=18.3 Hz, 1H), 5.45 (s, 1H), 7.01-7.07 (m, 1H), 7.09-7.18 (m, 3H), 7.20-7.26 (m, 4H), 7.35-7.58 (m, 4H), 8.47 (dd, *J*=1.7 and 4.9 Hz, 1H), 8.54 (dd, *J*=1.7 and 4.9 Hz, 1H).

^{13}C NMR (CDCl3, 100MHz): δ 13.1, 21.0, 31.4, 31.8, 42.6, 44.6, 48.8,52.2, 56.9, 77.7, 79.1, 108.8, 109.9, 114.1, 118.6, 118.9, 119.1, 119.5, 121.6, 126.2, 126.5, 131.1, 133.8, 134.1, 137.0, 149.5, 155.8, 167.0, 169.5, 169.8.

HRMS (ESI): calc. for $[M+H]^+$ $C_{25}H_{28}N_3O_4S$: 466.1795; Found: 466.1797.

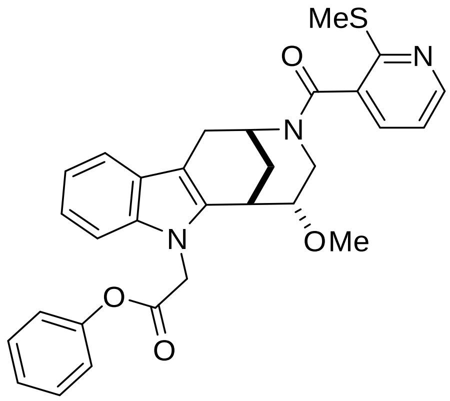

(±)-phenyl 2-(5-methoxy-3-(2-(methylthio)nicotinoyl)-3,4,5,6-tetrahydro-1H-2,6-methanoazocino[5,4-b]indol-7(2H)-yl)acetate (5ae):

Compound **5ae** was synthesised from **3a** following General Procedure **C**, using phenyl bromoacetate, directly followed by General procedure **F**.

^{1}H NMR (CDCl3, 500MHz): δ 2.00-2.29 (m, 4H), 2.47-2.64 (m, 6H), 2.65-2.71 (m, 2H), 2.81-3.11 (m, 3H), 3.17 (s, 3H), 3.23-3.29 (m, 1H), 3.32-3.38 (m, 1H), 3.40-3.45 (m, 4H), 3.70-3.83 (m, 1H), 3.99 (s, 1H), 4.18 (s, 1H), 4.29 (s, 1H), 4.91 (dd, *J*=5.2 and 12.9 Hz, 1H), 5.27 (app t, *J*=16.2 Hz, 2H), 5.39-5.45 (m, 2H), 5.48 (d, *J*=14.8 Hz, 1H), 6.95-7.04 (m, 5H), 7.06-7.10 (m, 1H), 7.12-7.24 (m, 4H), 7.25-7.38 (m, 6H), 7.40-7.58 (m, 4H), 8.02 (d, *J*=8.1 Hz, 1H), 8.14 (d, *J*=8.5 Hz, 1H), 8.50 (d, *J*=4.7 Hz, 1H), 8.57 (d, *J*=4.7 Hz, 1H).

^{13}C NMR (CDCl3, 100MHz): δ 12.3, 14.2, 22.5, 31.2, 32.7, 33.4, 41.5, 42.5, 44.8, 56.6, 57.4, 61.3, 69.4, 78.7, 108.9, 114.5, 115.7, 119.0, 119.4, 121.2, 121.9, 123.2, 124.8, 126.4, 128.5, 129.2, 129.5, 132.8, 133.1, 133.8, 136.4, 137.0, 149.6, 157.8, 166.6, 166.9, 169.2.

HRMS (ESI): calc. for $[M+H]^+$ $C_{30}H_{30}N_3O_4S$ $(M+H)^{+:}$ 528.1952; Found: 528.1950.

R4

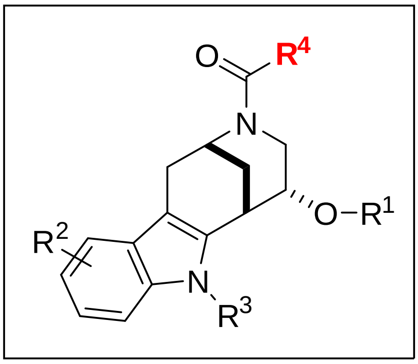

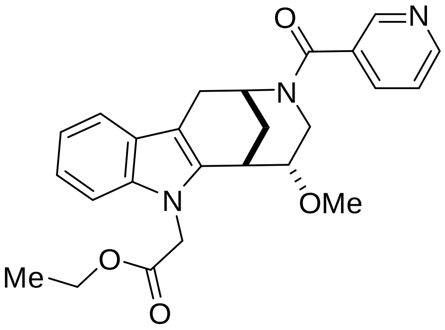

(±)-Ethyl 2-(5-methoxy-3-nicotinoyl-3,4,5,6-tetrahydro-1H-2,6-methanoazocino[5,4-b]indol-7(2H)-yl)acetate (5t):

Compound **5t** (35 mg, 88%) was synthesised from **12a** following General Procedure **H**, using nicotinic acid. The crude product was purified by chromatography (2% MeOH/CH_2Cl_2) to afford a colourless oil.

^{1}H NMR (CDCl3, 500MHz) (rotameric mixture): δ 1.23 (m, 3H), 1.95 (d, *J*=12.4 Hz, 0.6H), 2.04 (d, *J*=12.7 Hz, 0.6H), 2.13 (s, 0.8H), 2.64 (t, *J*=9.5 Hz, 0.6H), 2.86-2.96 (m, 1.4H), 3.16 (dd, *J*=5.6, 17.0 Hz, 0.6H), 3.19 (s, 1.2H), 3.33 (dd, *J*=5.9, 17.4 Hz, 0.4H), 3.41 (s, 1.8H), 3.49 (d, *J*=2.6 Hz, 1H), 3.53-3.55 (m, 0.4H), 3.59-3.72 (m, 1H), 4.11-4.24 (m, 2H), 4.27 (bs, 0.6H), 4.77-4.87 (m, 1.6H), 5.02 (d, *J*=18.0 Hz, 0.4H), 5.14 (d, *J*=18.1 Hz, 0.6H), 5.38 (bs, 0.4H), 7.10-7.13 (m, 1H), 7.19-7.23 (m, 2H), 7.39-7.52 (m, 2H), 7.79 (d, *J*=7.5 Hz, 0.4H), 7.88 (d, *J*=7.5 Hz, 0.6H), 8.67-8.76 (m, 2H).

^{13}C NMR (CDCl3, 100MHz) (rotameric mixture): δ 14.3, 26.8, 27.5, 29.4, 30.5, 30.8, 31.3, 38.3, 42.3, 43.9, 44.5, 48.2, 56.0, 60.5, 77.0, 77.9, 107.7, 108.1, 108.7, 117.1, 117.3, 118.7, 120.9, 123.0, 123.2, 125.3, 125.5, 132.1, 131.6, 132.8, 133.2, 134.6, 134.9, 136.1, 136.2, 145.7, 145.8, 148.8, 148.9, 166.6, 168.3, 168.5.

HRMS (ESI)**:** calc. for $[M+H]^+$ $C_{25}H_{28}N_3O_4$: 434.20743, found: 434.20705.

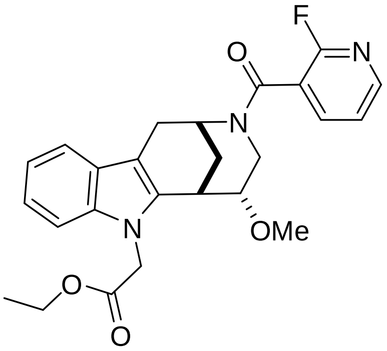

(±)-Ethyl 2-(3-(2-fluoronicotinoyl)-5-methoxy-1,2,3,4,5,6-hexahydro-7H-2,6-methanoazocino[5,4-b]indol-7-yl)acetate (5u):

Compound **5u** (5.4 mg, 20%) was synthesised following General Procedure **I**, using 2-fluoro nicotinic acid. The crude product was then purified directly by preparative HPLC (10% to 50% ACN/H_2O) to yield compound **5u**.

^{1}H NMR (CDCl3, 500MHz) (rotameric mixture 1:1.3): δ 1.23-1.26 (m, 3H), 1.97-2.00 (m, 1H), 2.10-2.21 (m, 1H), 2.62-2.80 (m, 1H), 2.90 (d, *J*=17.2 Hz, 1H), 3.10-3.22 (m, 2H), 3.33 (dd, *J*=17.3, 6.1 Hz, 0.44H), 3.41 (s, 2H), 3.48-3.49 (m, 1H), 3.66 (bs, 1H), 4.08 (bs, 0.56H), 4.12-4.24 (m, 2H), 4.80 (dd, *J*=19.2, 18.1 Hz, 1H), 4.87 (bs, 0.56H), 5.04 (d, *J*=18.0 Hz, 0.44H), 5.14 (d, *J*=18.1 Hz, 0.56H), 5.39 (bs, 0.44H), 7.10-7.13 (m, 1H), 7.19-7.23 (m, 2H), 7.27-7.29 (m, 0.44H),7.33-7.35 (m, 0.56H), 7.44 (bs, 0.56H), 7.50 (d, *J*=7.8 Hz, 0.44H), 7.87-7.92 (m, 1H), 8.28 (dd, *J*=4.8, 1.6 Hz, 0.44H), 8.33 (dd, *J*=4.8, 1.5 Hz, 0.56H)).

^{13}C NMR (CDCl3, 100MHz): δ 14.3, 27.6, 30.5, 32.0, 32.2, 43.4, 45.0, 45.0, 49.3, 57.0, 57.1, 61.5, 61.5, 78.1, 78.9, 109.1, 109.7, 118.2, 118.3, 119.3, 119.5, 119.6, 119.7, 121.9, 122.2, 122.2, 126.4, 126.5, 133.9, 134.2, 137.2, 137.3, 140.1, 148.9, 149.0, 149.1, 149.1, 157.8, 157.9, 159.1, 159.3, 163.9, 164.0, 169.3, 169.5

HRMS (ESI)**:** calc. for $[M+H]^+$ $C_{25}H_{27}N_3O_4F$: 452.19801, found: 452.19813.

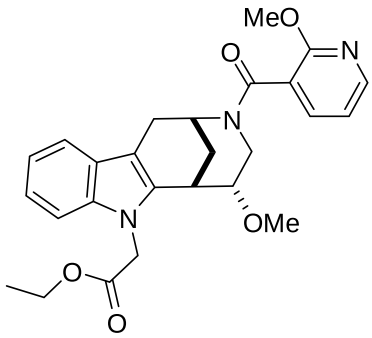

(±)-Ethyl 2-(5-methoxy-3-(2-methoxynicotinoyl)-1,2,3,4,5,6-hexahydro-7H-2,6-methanoazocino[5,4-b]indol-7-yl)acetate (5v):

Compound **5v** (39.0 mg, 92%) was synthesised from **12a** following General Procedure **H**, using 2-methoxy nicotinic acid. The crude product was purified by chromatography (3% MeOH/CH_2Cl_2) to afford a colourless oil.

^{1}H NMR (CDCl3, 500MHz): δ 1.24 (q, *J*=7.0 Hz, 3H), 1.90-1.96 (m, 0.8H), 2.09-2.17 (m, 1.2H), 2.55-3.14 (m, 3H), 3.18 (d, *J*=16.3 Hz, 1.2H), 3.27-3.36 (m, 1H), 3.41 (s, 1.8H), 3.46 (s, 1H), 3.60-3.71 (m, 1H), 3.87 (s, 0.4H), 4.01 (s, 0.6H), 4.10 (d, *J*=21.9 Hz, 2H), 4.14-4.24 (m, 2H), 4.79 (dd, *J*=14.6, 18.0 Hz, 1H), 4.88-4.93 (m, 0.6H), 5.04 (d, *J*=17.9 Hz, 0.4H), 5.15 (dd, *J*=5.4, 18.0 Hz, 0.6H), 5.42 (s, 0.4H), 6.96-7.23 (m, 4H), 7.41-7.54 (m, 1H), 7.60-7.68 (m, 1H), 8.21 (dd, *J*=1.9, 5.1 Hz, 0.4H), 8.27 (dd, *J*=1.9, 5.1 Hz, 0.6H).

^{13}C NMR (CDCl3, 100MHz): δ 14.3, 27.6, 28.4, 30.4, 30.6, 31.2, 31.7, 31.9, 32.2, 32.4, 38.8, 39.1, 42.9, 44.9, 45.0, 48.9, 49.1, 49.1, 54.3, 54.5, 57.0, 61.5, 78.1, 78.4, 79.1, 79.3, 109.0, 109.1, 117.2, 117.3, 117.4, 118.1, 118.3, 119.6, 120.7, 121.8, 126.4, 134.1, 137.2, 137.4, 147.2, 147.4, 166.2, 169.4, 169.5

HRMS (ESI)**:** calc. for $[M+H]^+$ $C_{26}H_{30}N_3O_5$: 464.21800, found: 464.21828.

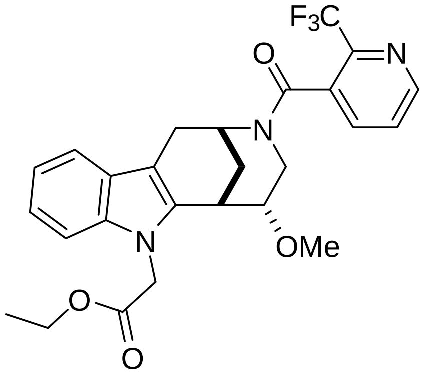

(±)-Ethyl 2-(5-methoxy-3-(2-(trifluoromethyl)nicotinoyl)-1,2,3,4,5,6-hexahydro-7H-2,6-methanoazocino[5,4-b]indol-7-yl)acetate (5w):

Compound **5w** (13.5 mg, 35%).) was synthesised following General Procedure **I**, using 2-trifluoromethoxy nicotinic acid. The crude product was purified directly by preparative HPLC (10% to 50% ACN/H_2O).

^{1}H NMR (CDCl3, 500MHz) (rotameric mixture): δ 1.21-1.26 (m, 3H), 1.90-1.93 (m, 0.5H), 1.96-1.98 (m, 0.2H), 2.08-2.15 (m, 1.3H), 2.61 (dd, *J*=12.8, 10.8 Hz, 0.4H), 2.66 (dd, *J*=12.8, 10.7 Hz, 0.2H), 2.77-2.83 (m, 0.8H), 2.87-2.92 (m, 0.8), 3.02-3.09 (m, 0.5H), 3.12-3.15 (m, 1.5H), 3.20 (dd, *J*=12.8, 4.9 Hz, 0.5H), 3.29-3.36 (m, 0.5H), 3.40-3.42 (m, 1.5H), 3.45-3.50 (m, 1H), 3.55 (dt, *J*=10.3, 4.4 Hz, 0.3H), 3.62-3.66 (m, 0.5H), 3.91-3.93 (m, 0.5H), 4.11-4.24 (m, 2H), 4.77 (dd, *J*=18.0, 5.5 Hz, 0.5H), 4.81 (dd, *J*=18.1, 4.4 Hz, 0.5H), 4.92 (dd, *J*=12.9, 5.0 Hz, 0.5H), 5.01 (dd, *J*=18.0, 12.1 Hz, 0.5H), 5.14 (dd, *J*=18.1, 8.1 Hz, 0.5H), 5.39-5.40(m, 0.2H), 5.44 (m, 0.3H), 7.10-7.15 (m, 1H), 7.19-7.23 (m, 2H), 7.43 (d, *J*=7.8 Hz, 0.4H), 7.48 (d, *J*=7.8 Hz, 0.2H), 7.50-7.54 (m, 0.8H), 7.58 (dd, *J*=7.9, 4.8 Hz, 0.2H), 7.60 (dd, *J*=7.8,

4.7 Hz, 0.2H), 7.64-7.65 (m, 0.6H), 7.75-7.76 (m, 0.3H), 7.82 (dd, *J*=7.8, 1.1 Hz, 0.3H), 8.76 (ddd, *J*=9.9, 4.6, 1.0 Hz, 0.5H), 8.81-8.82 (m, 0.5H).

^{13}C NMR (CDCl3, 100MHz): δ 14.2, 14.2, 26.6, 26.9, 27.8, 28.4, 29.9, 30.4, 30.4, 31.5, 31.7, 31.9, 32.0, 32.3, 38.6, 39.0, 42.8, 43.0, 44.7, 44.9, 44.9, 45.2, 49.0, 49.1, 56.8, 56.8, 56.9, 57.0, 61.3, 61.4, 61.4, 61.4, 77.7, 78.2, 78.2, 78.7, 108.4, 108.7, 108.8, 108.9, 109.0, 109.1, 109.4, 109.7, 117.9, 118.1, 118.2, 118.4, 119.6, 119.6, 119.6, 120.4, 120.5, 120.7, 120.7, 121.8, 122.0, 122.1, 122.1, 122.2, 122.3, 123.8, 123.9, 126.9, 126.2, 126.2, 126.4, 126.4, 126.5, 126.6, 131.3, 131.4, 131.8, 131.8, 133.4, 133.6, 134.0, 134.1, 135.4, 135.4, 135.5, 136.0, 137.1, 137.1, 137.2, 142.7, 142.9, 143.1, 143.1, 143.3, 143.3, 143.5, 143.6, 143.6, 143.7, 143.8, 149.8, 149.9, 150.0, 165.3, 165.4, 165.6, 165.8, 169.1, 169.3, 169.4

HRMS (ESI)**:** calc. for $[M+H]^+$ $C_{26}H_{27}N_3O_4F_3$: 502.19482, found: 502.19439.

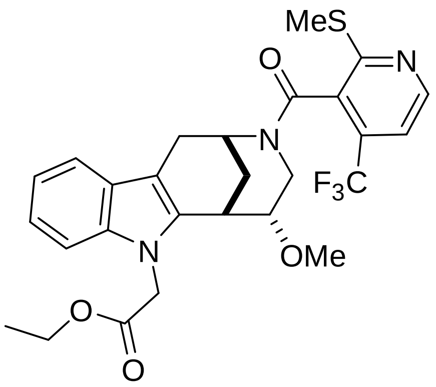

(±)-Ethyl 2-(5-methoxy-3-(2-(methylthio)-4-(trifluoromethyl)nicotinoyl)-1,2,3,4,5,6-hexahydro-7H-2,6-methanoazocino[5,4-b]indol-7-yl)acetate (5x):

Compound **5x** (80 mg, 73%) was synthesized following General Procedure **H**, using the corresponding carboxylic acid crude product synthesised following General Procedure **J** from 2-chloro-4-(trifluoromethyl)nicotinic acid (CAS: 590371-81-6). Compound **5x** was purified by preparative HPLC (10% to 50% ACN/H_2O).

^{1}H NMR (CDCl3, 400MHz) (rotameric mixture 1:2.3): δ 1.22-1.27 (m, 3H), 1.90 (ddt, *J*=26.3, 12.7, 2.5 Hz, 0.3H), 2.06-2.15 (m, 1H), 2.26 (dt, *J*=13.1, 3.6 Hz, 0.3H), 2.52 (s, 1H), 2.65-2.62 (m, 3H), 2.81-2.87 (m, 0.7H), 3.01-3.09 (m, 0.7H), 3.12-3.23 (m, 3H), 3.28-3.35 (m, 1H), 3.42 (d, *J*=4.9 Hz, 1H), 3.47-3.50 (m, 1H), 3.52-3.64 (m, 0.3H), 3.76-4.00 (m, 0.7H), 4.11-4.26 (m, 2H), 4.80 (t, *J*=16.4 Hz, 1H), 4.94-5.00 (m, 0.3H), 5.04 (d, *J*=17.9 Hz, 0.7H), 5.15 (dd, *J*=18.0, 2.7 Hz, 0.3H), 5.48 (bs, 0.7H), 7.09-7.18 (m, 1H), 7.20-7.24 (m, 2.3H), 7.27-7.31 (m, 0.7H), 7.48 (dd, *J*=12.9, 7.8 Hz, 0.3H), 7.54 (dd, *J*=11.4, 7.8 Hz, 0.7H), 8.66 (t, *J*=4.7 Hz, 0.7H), 8.64-8.71 (m, 0.3H).

^{13}C NMR (CDCl3, 100MHz): δ 13.5, 13.7, 13.9, 14.0, 14.1, 14.4, 26.7, 26.9, 27.1, 28.3, 30.1, 30.2, 30.6, 31.9, 32.0, 32.1, 32.5, 32.6, 39.0, 39.1, 43.1, 43.8, 45.1, 45.1, 49.6, 49.7, 57.1, 57.1, 57.2, 61.6, 77.9, 78.0, 78.4, 79.5, 109.0, 109.0, 109.1, 109.2, 109.3, 109.8, 109.9, 115.1, 115.1, 115.6, 118.3, 118.5, 119.6, 119.7, 119.7, 121.9, 126.5, 126.7, 126.8, 133.8, 134.0, 134.4, 137.3, 137.4, 150.0, 150.1, 150.1, 151.5, 158.4, 158.9, 159.2, 159.4, 163.9, 164.0, 169.4, 169.5, 169.6, 169.6

HRMS (ESI)**:** calc. for $[M+H]^+$ $C_{27}H_{29}N_3O_4F_3S$: 548.18254, found: 548.18209

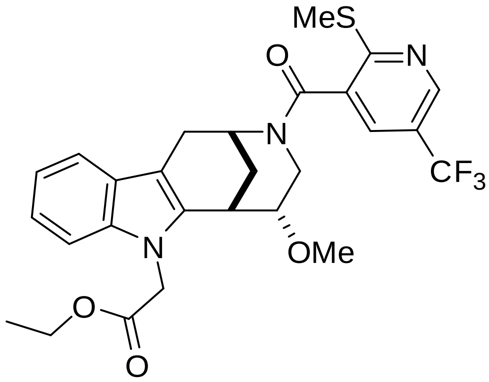

(±)-Ethyl 2-(5-methoxy-3-(2-(methylthio)-5-(trifluoromethyl)nicotinoyl)-1,2,3,4,5,6-hexahydro-7H-2,6-methanoazocino[5,4-b]indol-7-yl)acetate (5y):

Compound **5y** (76 mg, 84%) was synthesized following General Procedure **H**, using the corresponding carboxylic acid crude product synthesised following General Procedure **J** from 2-chloro-5-

(trifluoromethyl)nicotinic acid (CAS: 505084-59-3). Compound **5y** was purified by preparative HPLC (10% to 50% ACN/H_2O).

^{1}H NMR (CDCl3, 400MHz) (rotameric mixture 1:1): δ 1.23-1.28 (m, 3H), 1.98 (d, *J*=12.2 Hz, 0.5H), 2.15-2.32 (m, 1.5H), 2.58-2.67 (m, 4H), 2.95-3.13 (m, 2H), 3.20 (s, 1.5H), 3.26-3.36 (m, 1H), 3.42 (s, 1.5H), 3.49 (d, *J*=3.2 Hz, 1H), 3.67-3.73 (m, 0.5H), 3.95 (bs, 0.5H), 4.11-4.26 (m, 2H), 4.81 (dd, *J*=18.0, 13.4 Hz, 1H), 4.89 (dd, *J*=12.9, 4.9 Hz, 0.5H), 5.03 (d, *J*=17.9 Hz, 0.5H), 5.15 (d, *J*=18.0 Hz, 0.5H), 5.42 (bs, 0.5H), 7.10-7.17 (m, 1H), 7.19-7.24 (m, 2H), 7.51 (dd, *J*=19.1, 7.7 Hz, 1H), 7.62 (bs, 0.5H), 7.67 (bs, 0.5H), 8.69 (d, *J*=1.2 Hz, 0.5H), 8.76 (d, *J*=1.2 Hz, 0.5).

^{13}C NMR (CDCl3, 100MHz): δ 13.2, 13.4, 14.3, 28.5, 29.8, 30.3, 31.6, 31.9, 32.3, 39.2, 43.2, 45.0, 45.0, 49.3, 57.0, 61.4, 78.1, 79.2, 108.8, 109.0, 109.1, 109.7, 118.2, 118.3, 119.6, 121.9, 122.3, 125.0, 126.3, 126.5, 130.1, 130.8, 131.1, 133.7, 134.1, 137.2, 137.3, 146.2, 146.2, 161.1, 165.6, 165.7, 169.3, 169.4

HRMS (ESI)**:** calc. for $[M+H]^+$ $C_{27}H_{29}N_3O_4F_3S$: 548.18254, found:548.18391

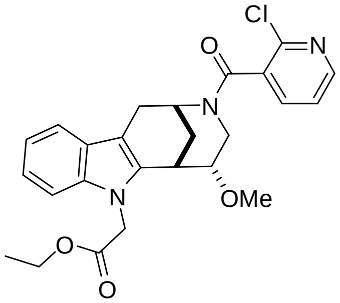

(±)-Ethyl 2-(3-(2-chloronicotinoyl)-5-methoxy-1,2,3,4,5,6-hexahydro-7H-2,6-methanoazocino[5,4-b]indol-7-yl)acetate(5af):

To a solution of 2-chloronicotinic acid (17.3 mg, 0.110 mmol) in toluene (1.6 mL, 0.067 M) oxalyl chloride (11.3 µL, 0.132 mmol) freshly distilled and a drop of DMF were added. The solution was stirred at reflux for 1h, then solvents were evaporated and the crude was redissolved in CH_2Cl_2 (0.25 mL, 0.55 M). The solution was canulated to another solution of compound **9** (30 mg, 0.091 mmol), triethylamine

(15.2 µL, 0.110 mmol) in CH2Cl2 (0.25 mL, 0.37 M). The mixture was allowed to stir at room temperature overnight. Then the organic phase was washed with water and BRINE, solvents were evaporated and the crude was purified by chromatography (3% MeOH/CH_2Cl_2). Pure compound **5af** (39.0 mg, 82%) was isolated as a colourless oil.

^{1}H NMR (CDCl3, 500MHz) (rotameric mixture): δ 1.22-1.27 (m, 3H), 1.93-2.29 (m, 2H), 2.60 (dd, *J*=10.9, 12.7 Hz, 0.4H), 2.69 (dd, *J*=13.8, 23.8 Hz, 0.6H), 2.78-2.90 (m, 0.6H), 2.96-3.01 (m, 1H), 3.10-3.22 (m, 1.4H), 3.28-3.36 (m, 1H), 3.40-3.42 (m, 1.6H), 3.48 (s, 1H), 3.62-3.78 (m, 1H), 3.98 (s, 0.6H), 4.11-4.24 (m, 2H), 4.81 (dd, *J*=10.3, 25.7 Hz, 1H), 4.90 (dd, *J*=5.3, 12.8 Hz, 0.6H), 5.03 (dd, *J*=7.6, 18.0 Hz, 0.4H), 5.14 (dd, *J*=7.2, 18.1 Hz, 0.6H), 5.43 (s, 0.4H), 7.09-7.15 (m, 1H), 7.19-7.23 (m, 2H), 7.29-7.43 (m, 1.4H), 7.48-7.53 (m, 0.6H), 7.60-7.73 (m, 1H), 8.42-8.49 (m, 1H).

^{13}C NMR (CDCl3, 100MHz): δ 14.2, 14.4, 18.8, 19.5, 20.5, 22.7, 26.9, 27.7, 27.7, 27.9, 28.4, 28.9, 29.1, 30.1, 30.3, 31.2, 31.5, 31.8, 32.1, 32.3, 33.7, 36.1, 38.7, 39.1, 41.4, 42.9, 42.9, 44.0, 44.9, 44.9, 45.1, 49.0, 49.3, 56.9, 56.9, 57.0, 57.0, 61.4, 77.8, 78.2, 78.7, 78.9, 108.5, 108.9, 108.9, 109.0, 109.5, 109.7, 117.9, 118.1, 118.2, 118.4, 119.5, 121.8, 122.8, 122.8, 123.0, 126.2, 126.4, 126.4, 132.4, 132.7, 132.8, 133.2, 133.5, 133.7, 134.0, 136.4, 136.5, 136.6, 137.1, 137.1, 137.1, 137.2, 146.6, 146.9, 147.1, 147.2, 150.0, 150.1, 150.1, 150.2, 165.2, 165.3, 165.4, 165.6, 169.2, 169.3, 169.3, 169.4.

HRMS (ESI)**:** calc. for $[M+H]^+$ $C_{25}H_{27}N_3O_4Cl$: 468.16846, found: 468.16951.

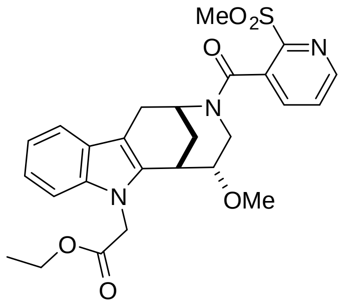

(±)-Ethyl 2-(5-methoxy-3-(2-(methylsulfonyl)nicotinoyl)-1,2,3,4,5,6-hexahydro-7H-2,6-methanoazocino[5,4-b]indol-7-yl)acetate (5ag)

mCPBA (362 mg, 2.1 mmol, 2.1 equiv.) as a solution in CH_2Cl_2 (5 mL) was added to a stirred solution of compound **5a** (479 mg, 1 mmol, 1 equiv.) in CH_2Cl_2 (5 mL) at 0 ˚C. After 30 min the reaction mixture was allowed to warm to rt. After 1 h the reaction mixture was poured into an ice-H2O mixture (20 mL) and layers separated. The organic layer was washed sequentially with sat. NaHCO3 (10 mL) and sat. brine solution (10 mL), dried over MgSO4, filtered and conc. in vacuo to give a crude which was purified by preparative HPLC eluting with 10-100% MeCN in H2O, afforded the desired compound **5ag** in 24 % yield.

^{1}H NMR (CDCl3, 500MHz): δ 1.20-1.28 (m, 6H), 1.80-1.85 (m, 1H), 1.93-2.13 (m, 2H), 2.25-2.32 (m, 1H), 2.42-2.50 (m, 1H), 2.60 (app. t, *J*=10.7 Hz, 1H), 2.75-2.92 (m, 2H), 2.96-3.12 (m, 2H), 3.20 (s, 3H), 3.25-3.33 (m, 6H), 3.37-3.52 (m, 6H), 3.71-3.78 (m, 1H), 3.84-3.92 (m, 1H), 3.98 (s, br,1H), 4.06-4.25 (m, 4H), 4.69-4.83 (m, 2H), 4.86-4.93 (m, 1H), 4.96-5.06 (m, 1H), 5.13 (app d, *J*=17 Hz, 1H), 5.44 (s, br,1H), 7.06-7.23 (m, 6H), 7.37-7.60 (m, 3H), 7.62-7.70 (m, 1H), 7.77-7.87 (m, 2H), 8.75 (app d, *J*=5.2 Hz,1H), 8.82 (app d, *J*=5.2 Hz,1H).

^{13}C NMR (CDCl3, 100MHz): δ 21.0, 31.4, 31.8, 36.4, 42.6, 44.6, 48.8,52.2, 56.9, 77.7, 79.1, 108.8, 109.9, 114.1, 118.6, 118.9, 119.1, 119.5, 121.6, 126.2, 126.5, 131.1, 133.8, 134.1, 137.0, 149.5, 158.8, 167.0, 169.5, 169.8.

HRMS (ESI): calc. for $[M+Na]^+$ $C_{26}H_{29}N_3O_6SNa$: 534.1669; Found: 534.1674.

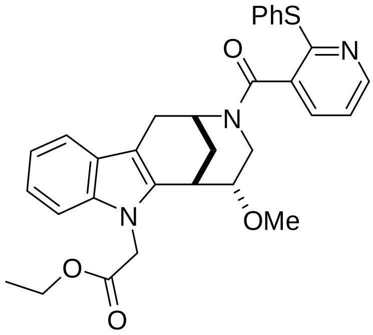

(±)-Ethyl 2-(5-methoxy-3-(2-(phenylthio)nicotinoyl)-1,2,3,4,5,6-hexahydro-7H-2,6-methanoazocino[5,4-b]indol-7-yl)acetate (5ah):

Compound **5ah** (6.5mg, 39%) was synthesised from compound **12a** following General Procedure **H**, using 2-(phenylthio)nicotinic acid. The crude product was purified by preparative HPLC, 10 to 100%, MeCN/H2O (0.1% TFA).

^{1}H NMR (CDCl3, 500MHz) (rotameric mixture): δ 1.25 (dt, *J*=11.0, 7.1 Hz, 3H), 1.99 (bs, 0.5H), 2.12-2.13 (m, 0.5H), 2.22 (bs, 0.5H), 2.41 (bs, 0.5H), 2.64-3.20 (m, 4H), 3.34 (dd, *J*=17.2, 6.0 Hz, 0.5H), 3.42 (s, 2H), 3.49 (d, *J*=2.9 Hz, 1H), 3.73-3.86 (m, 1H), 4.11 (bs, 0.5H), 4.13-4.24 (m, 2H), 4.80 (dd, *J*=22.7, 18.0 Hz, 1H), 4.93 (dd, *J*=12.9, 5.0 Hz, 0.5H), 5.03 (d, *J*=18.0 Hz, 0.5H), 5.16 (d, *J*=18.1 Hz, 0.5H), 5.46 (bs, 0.5H), 7.07-7.14 (m, 1.5H), 7.16 (dd, *J*=7.0, 5.0 Hz, 0.5H), 7.19-7.23 (m, 2H), 7.37-7.58 (m, 7H), 8.37 (dd, *J*=4.8, 1.8 Hz, 0.5H), 8.44 (dd, *J*=4.8, 1.6 Hz, 0.5).

^{13}C NMR (CDCl3, 100MHz): δ 14.2, 28.4, 30.1, 31.4, 31.8, 32.2, 39.0, 42.8, 44.9, 44.9, 49.1, 56.9, 61.3, 61.4, 77.9, 79.1, 108.8, 108.9, 109.0, 109.7, 118.0, 118.3, 119.5, 120.6, 121.7, 121.7, 126.3, 126.5, 128.7, 128.8, 129.2, 129.2, 132.2, 133.8, 134.0, 134.1, 134.4, 137.1, 137.1, 150.2, 155.0, 166.9, 166.9, 169.2, 169.4

HRMS (ESI)**:** calc. for $[M+H]^+$ $C_{31}H_{32}N_3O_4S$: 542.21080, found: 542.21115

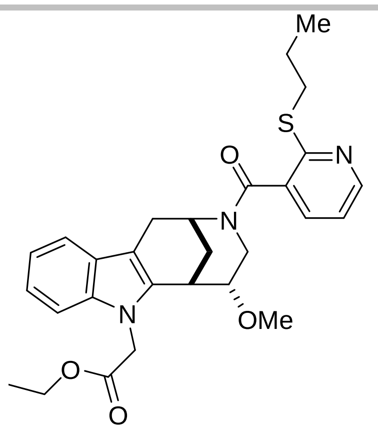

(±)-Ethyl 2-(5-methoxy-3-(2-(propylthio)nicotinoyl)-1,2,3,4,5,6-hexahydro-7H-2,6-methanoazocino[5,4-b]indol-7-yl)acetate (5ai):

Compound **5ai** (16.5 mg, 53%) was synthesised from **12a** following General Procedure **H**, using 2-(propylthio)nicotinic acid. The crude product was purified by preparative HPLC, 10 to 100%, MeCN/H2O (0.1% TFA).

^{1}H NMR (CDCl3, 500MHz) (rotameric mixture): δ 0.93-1.06 (m, 3H), 1.24 (dd, *J*=7.2, 15.5 Hz, 3H), 1.62-1.79 (m, 2H), 1.94-2.37 (m, 2H), 2.58-2.88 (m, 2H), 3.02-3.36 (m, 5H), 3.41-3-47 (m, 3H), 3.64-4.00 (m, 1H), 4.10-4.24 (m, 2H), 4.79 (t, *J*=17.8 Hz, 1H), 4.91 (dd, *J*=5.0, 12.8 Hz, 0.6H), 5.03 (d, *J*=18.0 Hz, 0.4H), 5.15 (d, *J*=18.1 Hz, 0.6H), 5.43 (s, 0.4H), 7.02 (bs, 0.4H), 7.09-7.14 (m, 1.6H), 7.18-7.23 (m, 2H), 7.36-7.52 (m, 2H), 8.44 (dd, *J*=1.66, 4.9 Hz, 0.4H), 8.50 (dd, *J*=1.7, 4.9 Hz, 0.6H).

^{13}C NMR (CDCl3, 100MHz): δ 13.8, 14.3, 22.9, 27.0, 28.4, 31.3, 31.9, 32.0, 32.1, 32.3, 39.2, 42.9, 45.0, 49.1, 57.0, 57.0, 61.5, 61.5,77.9, 109.1, 118.1, 119.3, 119.6, 121.8, 121.8, 126.4, 126.6, 133.6, 133.9, 134.2, 137.2, 137.2, 149.5, 167.3, 169.4, 169.6

HRMS (ESI)**:** calc. for $[M+H]^{+}$ $C_{28}H_{34}N_{3}O_{4}S$: 508.22645, found: 508.22657.

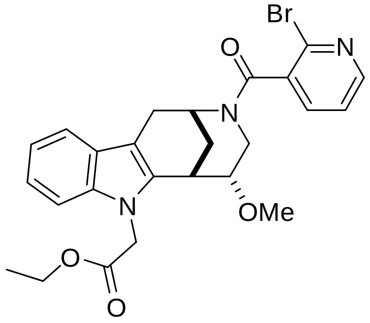

(±)-Ethyl 2-(3-(2-bromonicotinoyl)-5-methoxy-1,2,3,4,5,6-hexahydro-7H-2,6-methanoazocino[5,4-b]indol-7-yl)acetate (5aj):

Compound **5aj** (6.0 mg, 39%) was synthesised from **12a** following General Procedure **I**, using (2-bromo)nicotinic acid. The crude product was purified directly by preparative HPLC (10% to 50% ACN/H_2O).

^{1}H NMR (CDCl3, 700MHz) (rotameric mixture): δ 1.22-1.26 (m, 3H), 1.93 (s, 0.4H), 2.00 (d, *J*=11.9 Hz, 0.4H), 2.11 (m, 0.6H), 2.21 (d, *J*=13.2 Hz, 0.3H), 2.35 (d, *J*=12.8 Hz, 0.4H), 2.60 (dd, *J*=10.9, 12.7 Hz, 0.4H), 2.67-2.73 (m, 0.6H), 2.79 (dd, *J*=10.8, 12.6 Hz, 0.3H), 2.87 (d, *J*=17.2 Hz, 0.4H), 3.03-3.11 (m, 1H), 3.15-3.17 (m, 0.7H), 3.23 (s, 0.7H), 3.27-3.35 (m, 0.9H), 3.40-3.42 (m, 1.3H), 3.49 (m, 1H), 3.62-3.65 (m, 0.3H), 3.71 (dt, *J*=4.6, 10.3 Hz, 0.4H), 3.82-3.85 (m, 0.3H), 3.95-3.99 (m, 0.6H), 4.11-4.23 (m, 2H), 4.77-4.83 (m, 1H), 4.89-4.93 (m, 0.5H), 5.02 (dd, *J*=12.0, 18.0 Hz, 0.5H), 5.14 (dd, *J*=11.1, 18.1 Hz, 0.5H), 5.43 (m, 0.5H), 7.09-7.23 (m, 3H), 7.32-7.44 (m, 1H)7.49-7.53 (m, 1H), 7.59-7.66 (m, 1H), 8.40-8.47 (m, 1H).

^{13}C NMR (CDCl3, 100MHz): δ 14.2, 26.6, 27.8, 28.0, 28.4, 29.7, 29.9, 30.3, 31.2, 31.5, 31.8, 32.1, 32.3, 38.6, 39.1, 42.9, 43.0, 44.1, 44.9, 44.9, 44.9, 44.9, 44.9, 45.2, 49.1, 49.4, 56.9, 56.9, 57.0, 57.0, 61.3, 61.4, 61.4, 77.7, 78.2, 78.7, 78.9, 108.5, 108.9, 108.9, 109.0, 109.0, 109.5, 109.7, 117.9, 118.1, 118.2, 118.3, 119.5, 121.8, 121.8, 121.8, 126.2, 126.2, 126.4, 126.4, 133.5, 133.7, 134.0, 135.3, 135.6, 135.7, 135.7, 135.8, 135.9, 136.1, 136.5, 137.1, 137.1, 137.2, 138.1, 138.4, 138.6, 150.3, 150.3, 150.4, 165.9, 166.0, 166.0, 166.3, 169.2, 169.3, 169.3, 169.4

HRMS (ESI)**:** calc. for $[M+H]^+$ $C_{25}H_{27}N_3O_4Br$:512.11795, found: 512.11821. Calc. for $[M+H]^+$ $C_{25}H_{27}N_3O_4{}^{81}Br$: 514.11590, found: 514.11590

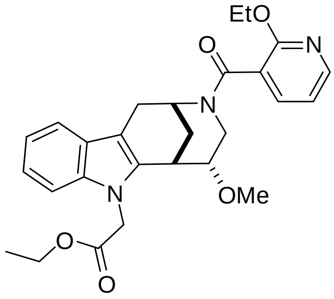

(±)-Ethyl 2-(3-(2-ethoxynicotinoyl)-5-methoxy-1,2,3,4,5,6-hexahydro-7H-2,6-methanoazocino[5,4-b]indol-7-yl)acetate (5ak):

Compound **5ak** (35mg, 80%) was synthesized from **12a** following General Proceure **H**, using (2-ethoxy)nicotinic acid. The crude product was purified directly by preparative HPLC (10% to 50% ACN/H_2O).

^{1}H NMR (CDCl3, 500MHz) (rotameric mixture): δ 1.25 (t, *J*=7.2 Hz, 3H), 1.43-1.53 (m, 3H), 1.90-1.97 (m, 0.8H), 2.11-2.22 (m, 1.2H), 2.54-3.15 (m, 3H), 3.19 (d, *J*=3.7 Hz, 1.2H), 3.24-3.34 (m, 0.8H), 3.39-3.41 (d, *J*=7.8 Hz, 1.8H), 3.47 (s, 1.2H), 3.60-3.72 (m, 1H), 4.03 (s, 0.6H), 4.10-4.31 (m, 2.4H), 4.41-4.53 (m, 1H), 4.54-4-63 (m, 0.6H), 4.76-4.85 (m, 1H), 4.88-4.94 (m, 0.6H), 5.04 (dd, *J*=7.9, 18.0 Hz, 0.4H), 5.15 (dd, *J*=8.0, 18.1 Hz), 5.42 (s, 0.4H), 6.92-7.03 (m, 1H), 7.08-7.13 (m, 1H), 7.20 (m, 2H), 7.43 (dd, *J*=7.7, 18.4 Hz, 0.6H), 7.49 (d, *J*=7.7 Hz, 0.4H), 7.58-7.68 (m, 1H), 8.17 (m, 0.4H), 8.24 (d, *J*=3.8 Hz, 0.6H).

^{13}C NMR (CDCl3, 100MHz): δ 14.3, 14.4, 14.4, 14.8, 15.0, 18.9, 19.6, 20.6, 22.7, 27.6, 27.8, 27.8, 28.4, 29.0, 29.2, 30.1, 30.6, 31.2, 31.5, 31.7, 32.1, 32.2, 32.3, 38.7, 38.1, 41.4, 42.8, 42.9, 44.2, 44.7, 45.0, 48.7, 48.9, 56.8, 56.9, 57.0, 57.1, 61.4, 62.8, 62.8, 62.9, 63.0, 78.2, 78.3, 79.0, 79.1, 108.9, 109.0, 109.0, 109.2, 109.7, 110.0, 116.8, 116.9, 117.1, 117.2, 118.0, 118.1, 118.2, 118.3, 119.5, 120.7, 120.8, 120.9, 121.3,

121.8, 126.4, 126.4, 126.5, 126.6, 133.6, 134.0, 134.3, 134.3, 137.2, 137.2, 137.3, 137.4, 138.1, 147.3, 147.4, 158.6, 158.8, 159.0, 159.1, 166.3, 166.4, 167.0, 169.4, 169.5

HRMS (ESI)**:** calc. for $[M+H]^+$ $C_{27}H_{32}N_3O_5$: 478.23365, found: 478.23391.

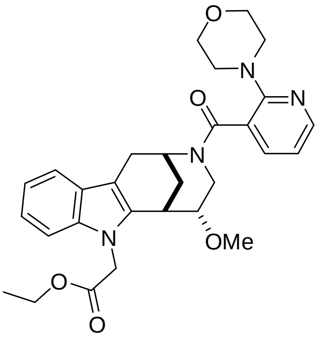

(±)-Ethyl 2-(5-methoxy-3-(2-morpholinonicotinoyl)-1,2,3,4,5,6-hexahydro-7H-2,6-methanoazocino[5,4-b]indol-7-yl)acetate (5al):

Compound **5al** (11.3 mg, 73%) was synthesized from **12a** following General Proceure **I**, using 2-morpholinonicotinic acid. The crude product was purified directly by preparative HPLC (10% to 50% ACN/H_2O).

^{1}H NMR (CDCl3, 500MHz) (rotameric mixture): δ 1.22-1.27 (m, 3H), 1.98-2.28 (m, 2H), 2.55-2.69 (m, 1H), 2.82 (dd, *J*=30.5, 17.3 Hz, 0.5H), 2.92-2.97 (m, 0.5H), 3.12-3.18 (m, 2H), 3.26-3.91 (m, 13H), 4.10-4.24 (m, 2H), 4.76-4.83 (m, 1.3H), 4.93 (dd, *J*=12.6, 4.8 Hz, 0.2H), 5.03 (dd, *J*=18.0, 6.0 Hz, 0.5H), 5.13 (dd, *J*=18.0, 6.4 Hz, 0.5H), 5.37 (d, *J*=30.2 Hz, 0.5H), 6.87-7.00 (m, 1H), 7.07-7.16 (m, 1H), 7.17-7.24 (m, 2H), 7.40 (d, *J*=7.8 Hz, 0.3H), 7.46-7.51 (m, 1H), 7.55-7.61 (m, 0.7H), 8.27 (ddd, *J*=9.8, 4.9, 1.5 Hz, 0.5H), 8.34-8.35 (m, 0.5H).

^{13}C NMR (CDCl3, 100MHz): δ 14.3, 27.3, 27.9, 27.9, 28.5, 30.6, 31.2, 31.6, 31.8, 32.0, 32.1, 32.3, 38.5, 39.4, 42.8, 42.9, 44.3, 44.8, 44.9, 45.0, 45.0, 48.6, 49.0, 49.5, 49.8, 49.9, 50.0, 56.9, 56.9, 57.0, 57.1, 61.5, 61.5, 66.9, 67.0, 67.1, 78.2, 78.5, 78.9, 79.2, 108.8, 108.9, 109.0, 109.1, 109.1, 109.3, 109.6, 116.0, 116.5, 116.6, 116.7, 118.0, 118.1, 118.2, 118.2, 119.6, 119.7, 119.9, 121.9, 121.9, 122.0, 122.9, 122.9, 123.4,

126.3, 126.3, 126.4, 126.5, 133.6, 133.8, 133.8, 134.4, 137.2, 137.2, 137.2, 169.3, 169.5 **HRMS** (ESI)**:** calc. for $[M+H]^+$ $C_{29}H_{35}N_4O_5$: 519.26020, found: 519.26056.

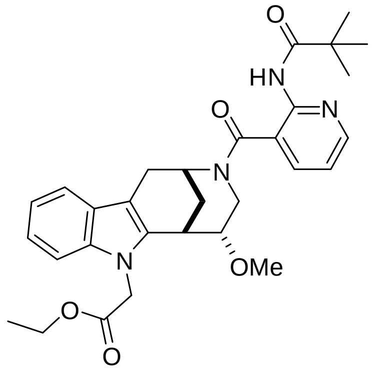

(±)-Ethyl 2-(5-methoxy-3-(2-pivalamidonicotinoyl)-1,2,3,4,5,6-hexahydro-7H-2,6-methanoazocino[5,4-b]indol-7-yl)acetate (5am):

Compound **5am** (3.2 mg, 10%) was synthesized from **12a** following General Proceure **I**, using 2-pivalamidonicotinic acid. The crude product was purified directly by preparative HPLC (10% to 50% ACN/H_2O) to yield compound **5am**. Due to lack of sufficient product the compound was characterized only by HRMS (ESI) measurement. U-HPLC chromatogram showed a purity above 95%.

HRMS (ESI)**:** calc. for $[M+H]^+$ $C_{30}H_{37}N_4O_5$: 533.27585, found: 533.27488

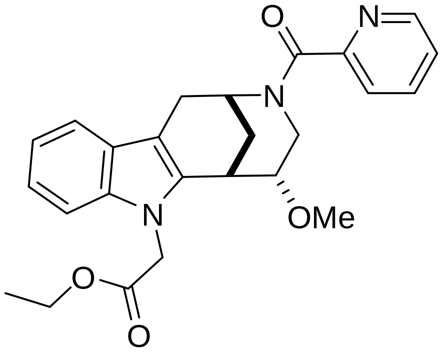

(±)-Ethyl 2-(5-methoxy-3-picolinoyl-1,2,3,4,5,6-hexahydro-7H-2,6-methanoazocino[5,4-b]indol-7-yl)acetate (5an):

Compound **5an** (25.7mg, 65%) was synthesized from **12a** following General Proceure **H**, using picolinic acid. The crude product was purified by chromatography (2% MeOH/CH_2Cl_2) to afford the product as a colourless oil.

^{1}H NMR (CDCl3, 500MHz) (rotameric mixture): δ 1.24 (q, *J*=7.2 Hz, 3H), 1.92 (dt, *J*=2.6, 12.7 Hz, 0.6H), 2.10 (dt, *J*=2.5, 13.0 Hz, 0.4H), 2.10-2.24 (m, 1H), 2.65 (dd, *J*=10.9, 12.6 Hz, 0.6H), 2.86 (dd, *J*=12.3, 13.6 Hz, 0.4H), 2.93-3.00 (m, 1H), 3.06-3.11 (m, 0.6H), 3.22 (s, 1.2H), 3.33 (dd, *J*=6.1, 17.3 Hz, 0.4H), 3.40 (s, 1.8H), 3.48 (m, 1H), 3.69-3.73 (m, 0.7H), 3.80-3.85 (m, 0.7H), 4.10-4.24 (m, 2H), 4.41 (bs, 0.6H), 4.80 (dd, *J*=15.3, 18.0 Hz, 1H), 4.87 (dd, *J*=4.9, 12.9 Hz, 0.6H), 5.06 (d, *J*=18.0Hz, 0.4H), 5.16 (d, *J*=18.1 Hz, 0.6H), 5.38 (bs, 0.4H), 7.08-7.12(m, 1H), 7.17-7.22 (m, 2H), 7.38 (dd, *J*=5.3, 6.9 Hz, 0.4H), 7.43 (dd, *J*=5.2, 7.1 Hz, 0.6H), 7.46 (d, *J*=7.8 Hz, 0.6H), 7.50 (d, *J*=7.8 Hz, 0.4H), 7.63 (d, *J*=7.9 Hz, 0.4H), 7.65 (d, *J*=7.8 Hz, 0.6H), 7.85 (t, *J*=7.9 Hz, 0.4H), 7.89 (t, *J*=7.7 Hz, 0.6H), 8.59 (d, *J*=4.6 Hz, 0.4H), 8.68 (d, *J*=4.6 Hz, 0.6H).

^{13}C NMR (CDCl3, 100MHz) (rotameric mixture): δ 14.3, 27.4, 28.5, 30.4, 31.2, 32.0, 32.3, 39.4, 43.4, 44.6, 45.0, 48.6, 56.8, 57.0, 61.4, 78.1, 78.7, 108.97, 108.98, 109.3, 109.7, 118.2, 118.3, 119.5, 121.69, 121.71, 123.5, 124.0, 124.7, 124.8, 126.4, 126.6, 134.3, 137.2, 138.1, 138.3, 147.6, 148.1, 153.8, 154.4, 167.2, 167.5, 1690.4, 169.6.

HRMS (ESI)**:** calc. for $[M+H]^+$ $C_{25}H_{28}N_3O_4$: 434.20743, found: 434.20535.

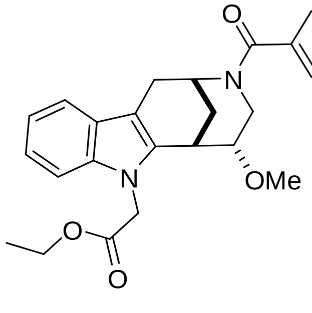

(±)-Ethyl 2-(3-isonicotinoyl-5-methoxy-1,2,3,4,5,6-hexahydro-7H-2,6-methanoazocino[5,4-b]indol-7-yl)acetate (5ao):

Compound **5ao** (4 mg, 31%) was synthesized from **12a** following General Proceure **I**, using isonicotinic acid. The crude product was purified by preparative HPLC (10% to 50% ACN/H_2O).

^{1}H NMR (CDCl3, 500MHz) (rotameric mixture): δ 1.25 (td, *J*=3.8, 7.1 Hz, 3H), 1.98-2.19 (m, 2H), 2.66 (dd, *J*=10.9, 12.7 Hz, 0.5H), 2.85-2.97 (m, 1H), 3.17 (dd, *J*=5.9, 17.1 Hz, 0.5H), 3.19 (s, 1.2H), 3.35 (td. *J*=5.8, 11.7 Hz, 1H), 3.41 (s, 1.8H), 3.51 (s, 1H), 3.64-3.68 (m, 1H), 4.07 (bs, 0.7H), 4.11-4.24 (m, 2.5H), 4.77-4.83 (m, 1.5H), 5.00 (d, *J*=18.0 Hz, 0.4H), 5.13 (d, *J*=18.0 Hz, 0.6H), 5.35 (bs, 0.3H), 7.11-7.16 (m, 1H), 7.20-7.24 (m, 2H), 7.45 (d, *J*=7.8 Hz, 0.6H), 7.50 (d, *J*=7.8 Hz, 0.4H), 7.62 (d, *J*=5.2 Hz, 1H), 7.68 (d, *J*=4.9 Hz, 1H), 8.78 (bs, 1H), 8.86 (bs, 1H).

^{13}C NMR (CDCl3, 100MHz) (rotameric mixture): δ14.3, 27.9, 28.6, 30.2, 31.4, 31.8, 32.2, 39.4, 43.6, 44.9, 45.0, 45.6, 49.3, 57.1, 57.2, 61.6, 77.9, 78.8, 108.4, 109.2, 109.5, 118.1, 118.3, 119.8, 122.1, 122.2, 122.9, 123.2, 126.2, 126.4, 133.4, 133.8, 137.2, 137.3, 145.5, 145.9, 149.4, 166.1, 169.2, 169.4.

HRMS (ESI)**:** calc. for $[M+H]^+$ $C_{25}H_{28}N_3O_4$: 434.20743, found: 434.20660.

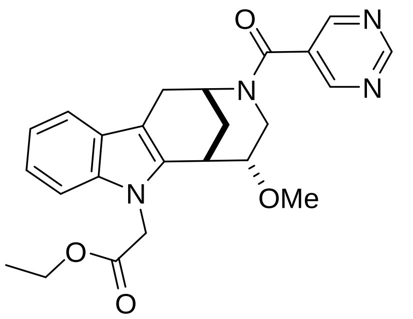

(±)-Ethyl 2-(5-methoxy-3-(pyrimidine-5-carbonyl)-1,2,3,4,5,6-hexahydro-7H-2,6-methanoazocino[5,4-b]indol-7-yl)acetate (5ap)

Compound **5ap** (9.0mg, 23%) was synthesized from **12a** following General Proceure **H**, using pyrimidine-5-carboxylic acid. The crude product was purified by preparative HPLC (10% to 50% ACN/H_2O).

^{1}H NMR (CDCl3, 400MHz) (rotameric mixture 1:1.5): δ 1.25 (t, *J*=7.0 Hz, 3H), 1.99-2.15 (m, 2H), 2.66 (t, *J*=11.7 Hz, 0.6H), 2.88-2.95 (m, 1H), 3.01 (t, *J*=10.4, 0.4H), 3.19-3.24 (m, 2H), 3.51-3.57 (m, 1.4H), 3.60-3.72 (m, 1H), 4.11-4.25 (m, 2.6H), 4.77-4.87 (m, 1.6H), 5.02 (d, *J*=18.0 Hz, 0.4H), 5.14 (d, *J*=18.1 Hz, 0.6H), 5.38 (bs, 0.4H), 7.11-7.16 (m, 1H), 7.19-7.22 (m, 2H), 7.46 (d, *J*=7.8 Hz, 0.6H), 7.54 (d, *J*=7.6 Hz, 0.4H), 8.80 (s, 0.8H), 8.88 (s, 1.2H), 9.26 (s, 0.4H), 9.32 (m, 0.6H).**HRMS** (ESI)**:** calc. for $[M+H]^+$ $C_{24}H_{27}N_4O_4$: 435.20268, found: 435.20259.

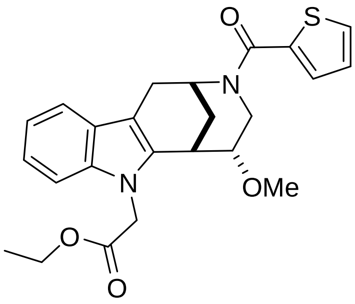

(±)-Ethyl 2-(5-methoxy-3-(thiophene-2-carbonyl)-1,2,3,4,5,6-hexahydro-7H-2,6-methanoazocino[5,4-b]indol-7-yl)acetate (5aq)

Compound **12a** (20 mg, 0.061 mmol), triethylamine (10.5 µL, 0.076 mmol) and thiophene-2-carbonyl chloride (6.52 µL, 0.061 mmol) were dissolved in CH_2Cl_2 (0.09 M, 0.667 mL), and allowed to stir overnight. Then solvents were evaporated and the crude was purified by chromatography (1% MeOH/CH2Cl2) to yield compound **5aq** (26.5mg, 98%) as a colourless oil.

^{1}H NMR (CDCl3, 500MHz) (rotameric mixture): δ 1.26 (t, *J*=7.1 Hz, 3H), 1.78 (bs, 1H), 2.00-2.07 (m, 2H), 2.62 (bs, 0.6H), 2.95 (d, *J*=15.1 Hz, 1.4H), 3.25-3.37 (m, 4H), 3.48 (d, *J*=3.0 Hz, 1H), 3.62 (bs, 1H),

4.12-4.25 (m, 2H), 4.81 (d, *J*=18.0 Hz, 2H), 5.12 (bs, 1H), 7.08 (m, 1H), 7.11-7.14 (m, 1H), 7.19-7.24 (m, 2H), 7.35 (bs, 1H), 7.47-7.50 (m, 2H).

^{13}C NMR (CDCl3, 100MHz) (rotameric mixture): δ 14.3, 22.8, 27.8, 29.2, 32.2, 41.5, 45.0, 57.0, 61.5, 78.1, 109.0, 118.2, 119.6, 121.8, 126.5, 126., 128.7, 134.3, 137.2, 137.7, 164.0, 169.5.

HRMS (ESI)**:** calc. for $[M+H]^+$ $C_{24}H_{27}N_2O_4S$: 439.16860, found: 439.16819.

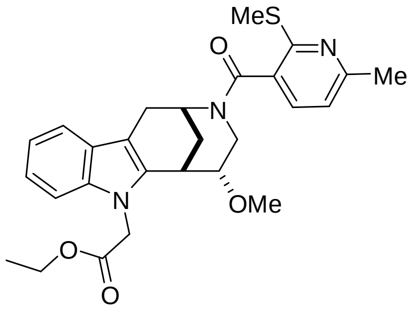

(±)-Ethyl 2-(5-methoxy-3-(6-methyl-2-(methylthio)nicotinoyl)-1,2,3,4,5,6-hexahydro-7H-2,6-methanoazocino[5,4-b]indol-7-yl)acetate (5ar):

Compound **5ar** (72 mg, 71%) was synthesized following General Procedure **H**, using the corresponding carboxylic acid crude product synthesised following General Procedure **J** from 2-chloro-6-methylnicotinic acid. The crude product was then purified by preparative HPLC (10% to 50% ACN/H_2O).

^{1}H NMR (CDCl3, 400MHz) (rotameric mixture 1:1)**:**δ 1.22-1.27 (m, 3H), 1.96 (d, *J*=11.6 Hz, 1H), 2.10-2.20 (m, 1H), 2.55 (s, 2H), 2.67 (s, 2H), 2.76-3.09 (m,2H), 3.19 (s, 1.5H), 3.29-3.39 (s, 1.5H), 3.47 (d, *J*=3.0 Hz, 1H), 3.69 (bs, 0.5H), 4.04 (bs, 0.5H), 4.10-4.26 (m, 2H), 4.80 (dd, *J*=18.0, 13.2 Hz, 1H), 4.88 (dd, *J*=12.9, 4.9 Hz, 0.5H), 5.03 (d, *J*=18.0 Hz, 0.5H), 5.14 (d, *J*=18.0 Hz, 0.5H), 5.43 (bs, 0.5H), 6.94 (d, *J*=7.7 Hz, 0.5H), 7.02 (d, *J*=7.7 Hz, 0.5H), 7.09-7.16 (m, 1H), 7.18-7.23 (m, 2H), 7.38 (d, *J*=6.5 Hz, 0.5H), 7.48 (dd, *J*=27.7, 7.6 Hz, 1.5H), 9.35 (bs, 1H).

^{13}C NMR (CDCl3, 100MHz): δ 13.5, 13.7, 24.0, 28.4, 30.2, 31.4, 31.9, 32.2, 39.5, 43.5, 45.0, 49.5, 57.1, 57.1, 61.5, 78.1, 79.1, 108.8, 109.0, 109.1, 109.6, 118.1, 118.4, 119.3, 119.7, 121.9, 126.4, 126.6, 128.6, 133.8, 134.1, 134.8, 137.2, 137.3, 154.7, 159.1, 159.1, 159.6, 167.9, 169.4, 169.6

HRMS (ESI)**:** calc. for $[M+H]^+$ $C_{27}H_{32}N_3O_4S$: 494.21080, found: 494.21134

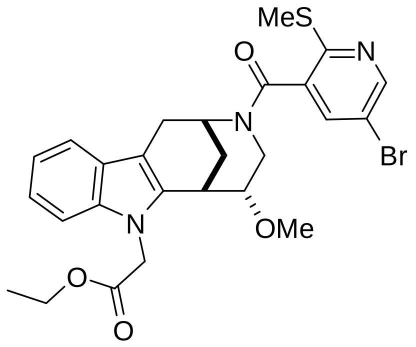

(±)-Ethyl 2-(3-(5-bromo-2-(methylthio)nicotinoyl)-5-methoxy-1,2,3,4,5,6-hexahydro-7H-2,6-methanoazocino[5,4-b]indol-7-yl)acetate (5as):

Compound **5as** (66 mg, 71%) was synthesized following General Procedure **H**, using the corresponding carboxylic acid crude product synthesised following General Procedure **J** from 2-chloro-5-bromonicotinic acid. The crude product was purified by flash chromatography (15% E.A./CH_2Cl_2) to give afford the product as a colorless oil.

^{1}H NMR (CDCl3, 400MHz) (rotameric mixture 1:1): δ 1.22-1.27 (m, 3H), 1.97 (d, *J*=11.2 Hz, 0.5H), 2.12-2.32 (m, 1.5H), 2.53-2.61 (m, 4H), 2.79-2.94 (m, 1H), 3.11 (dd, *J*=17.0, 5.7 Hz, 1H), 3.21 (s, 1.5H), 3.28-3.35 (m, 1H), 3.41 (s, 1.5H), 3.48 (d, *J*=3.2 Hz, 1H), 3.67 (bs, 0.5H), 4.00 (bs, 0.5H), 4.10-4.26 (m, 2H), 4.76-4.89 (m, 1.5H), 5.03 (d, *J*=17.9 Hz, 0.5H), 5.15 (d, *J*=18.0 Hz, 0.5H), 7.10-7.15 (m, 1H), 7.18-7.24 (m, 2H), 7.47-7.59 (m, 2H), 8.50 (d, *J*=2.2 Hz, 0.5H), 8.56 (d, *J*=2.2 Hz, 0.5H).

^{13}C NMR (CDCl3, 100MHz): δ 12.1, 12.3, 13.2, 27.4, 28.7, 29.1, 30.5, 30.8, 31.2, 38.1, 41.9, 43.9, 48.1, 55.9, 55.9, 60.3, 76.9, 78.0, 107.7, 107.9, 108.6, 114.7, 117.0, 117.2, 118.5, 118.5, 120.7, 125.2, 125.4, 131.0, 131.5, 132.7, 133.0, 134.5, 135.1, 136.1, 136.1, 149.3, 153.7, 164.3, 164.5, 168.1, 168.3

HRMS (ESI)**:** calc. for $[M+H]^+$ $C_{26}H_{29}N_3O_4{}^{81}BrS$: 560.10362, found: 560.10518

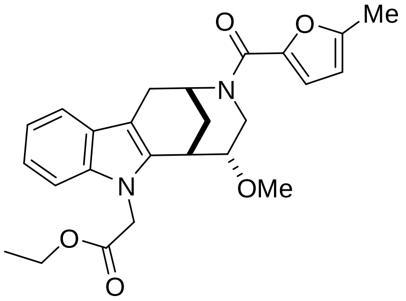

(±)-Ethyl 2-(5-methoxy-3-(5-methylfuran-2-carbonyl)-1,2,3,4,5,6-hexahydro-7H-2,6-methanoazocino[5,4-b]indol-7-yl)acetate (5at):

Compound **5at** (24mg, 90%) was synthesized following General Procedure **E**, using-5-methylfuran-2-carbonyl chloride. The crude product was purified by chromatography (1% MeOH/CH_2Cl_2) to yield compound **5at** (24mg, 90%) as a colourless oil.

^{1}H NMR (CDCl3, 500MHz): δ 1.25 (t, *J*=7.1 Hz, 3H), 2.05-2.10 (m, 2H), 2.38 (m, 4H), 2.58 (bs, 0.6H), 2.97 (m, 1.4H), 3.29 (dd, *J*=5.9, 17.1 Hz, 1H), 3.37 (s, 3H), 3.47 (s, 1H), 3.63 (m, 1H), 4.19 (m, 2H), 4.81 (d, *J*=18.0 Hz, 1H), 5.01-5.31 (m, 2H), 6.11 (bs, 1H), 6.9 (bs, 1H), 7.11 (t, *J*=7.5 Hz, 1H), 7.21 (q, *J*=8.0 Hz, 2H), 7.49 (d, *J*=7.8 Hz, 1H).

^{13}C NMR (CDCl3, 100MHz): δ 14.0, 14.3, 32.2, 39.6, 45.0, 47.8, 56.9, 61.4, 107.8, 108.0, 117.7, 118.2, 119.5, 121.7, 126.5, 134.4, 137.2, 146.2, 154.5, 160.0, 169.5.

HRMS (ESI)**:** calc. for $[M+H]^+$ $C_{25}H_{29}N_2O_5$: 437.20710, found: 437.20703.

Cheminformatic Analysis

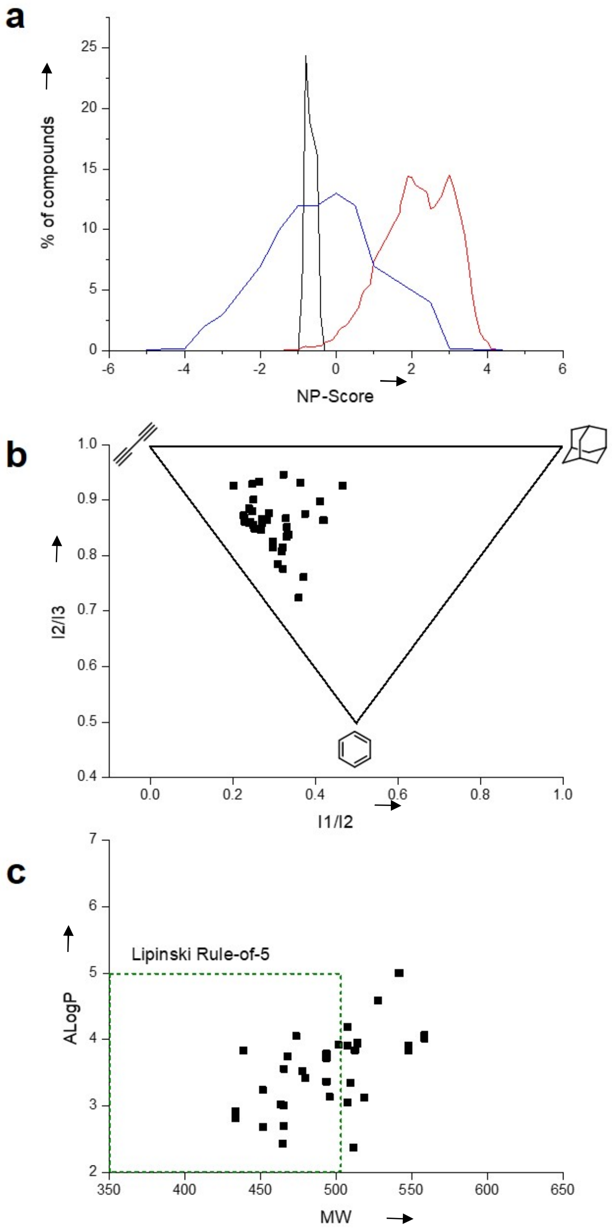

Supporting Figure S2: Chemoinformatic analysis of the indomorphan pseudo-NP collection. a) NP-score distribution. This class of pseudo-NPs displays a narrow NP-score distribution (black line) in a part of the graph that is poorly occupied by NPs (red line) yet densely covered by compounds in the DrugBank collection (blue line). b) PMI plot of the compounds in this collection. The majority of compounds reside away from the rod-like to disc-like vertex exhibiting higher three-dimensional

character. Most synthetic molecules tend to congest along the rod-disk axis (for comparison see Sauer and Schwarz)[5]. In contrast, the distribution of these pseudo-NPs in the PMI plot moves away from this axis and is comparable to the distribution displayed by diverse NPs and bioactive compounds which incorporate the indole and morphan fragments. c) The ALogP vs MW plot, demonstrates that the majority of the prepared indomorphans fall within Lipinski Ro5 space.

Supporting Table S2: Lipinski-space metrics analyses of the indomorphan pseudo-NP collection. For each metric the range and average values for the collection are given.

Property	Range	Average
MW	316-528	420
ALogP	2.37-4.67	3.88
Heavy Atom Count	23-34	30
Fsp^3	0.35-0.61	0.44
Total Polar Surface Area ($Å^2$)	84-146	104
H-Bond Donors	1.0-2.0	2
H-Bond Acceptors	6.0-10.0	7

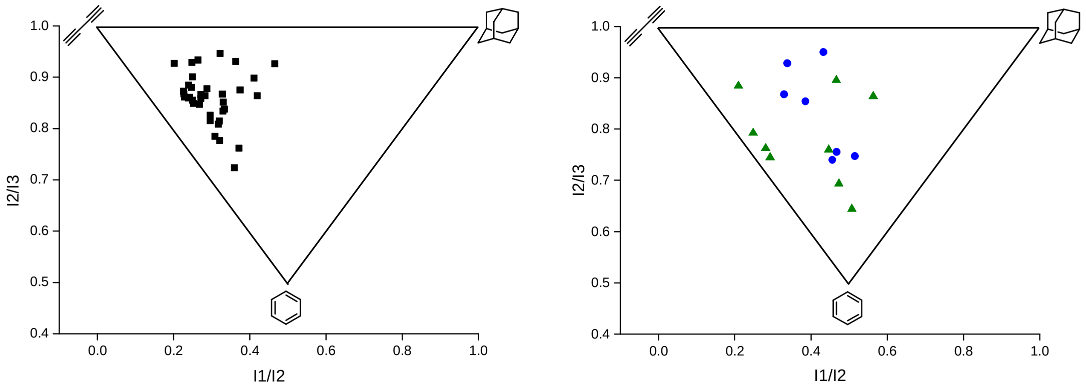

Supporting Figure S3: PMI plots for indomorphans (left, black squares) and indole- (right, green triangles) and morphan-containing (right, blue squares) NPs and non-naturally occurring, biologically active compounds. For chemical structures, see Supporting Schemes S3 and S4.

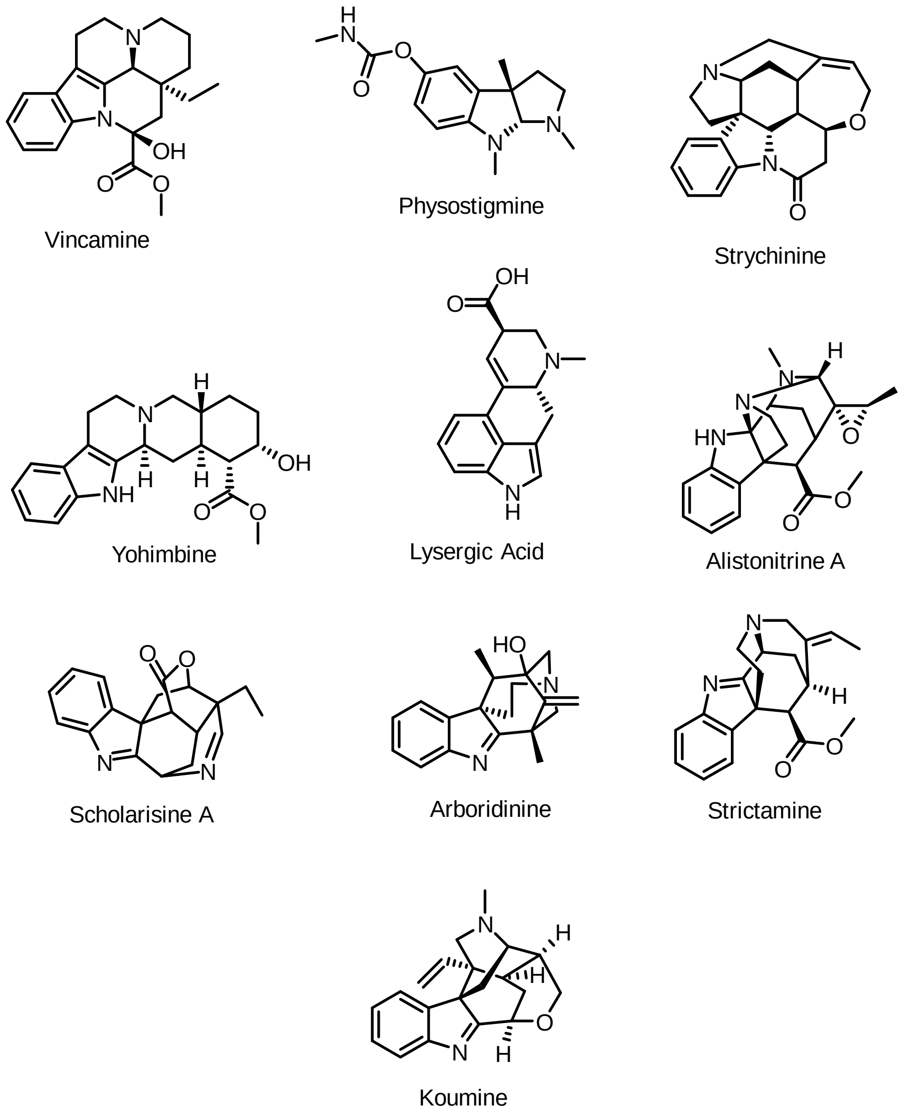

Supporting Scheme S3: Structures of indole-containing NPs used for the PMI plot in Supporting Figure S3.

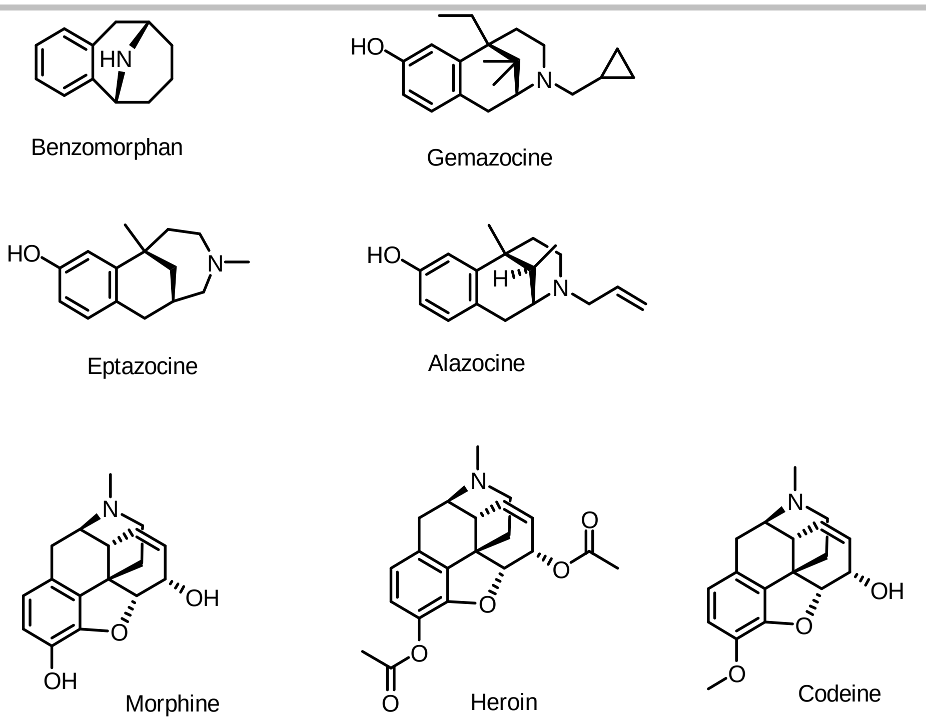

Supporting Scheme S4: Structures of morphan-containing NPs and non-naturally occurring biologically active compounds, used for the PMI plot in Supporting Figure S3.

Synthesis of the indomorphan (±)-Glupin fragments:

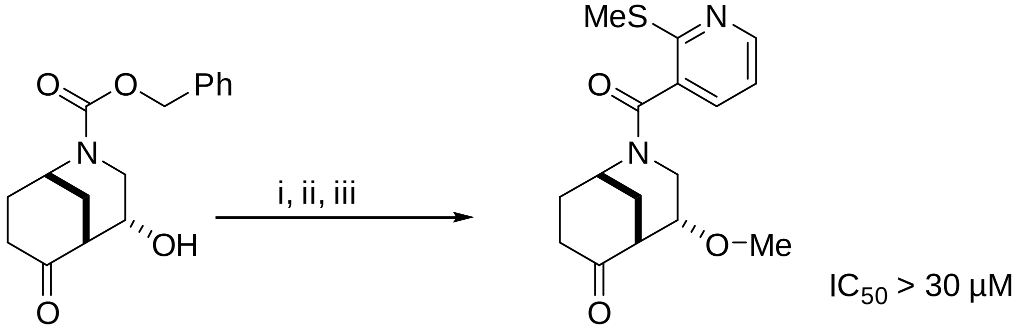

1 6

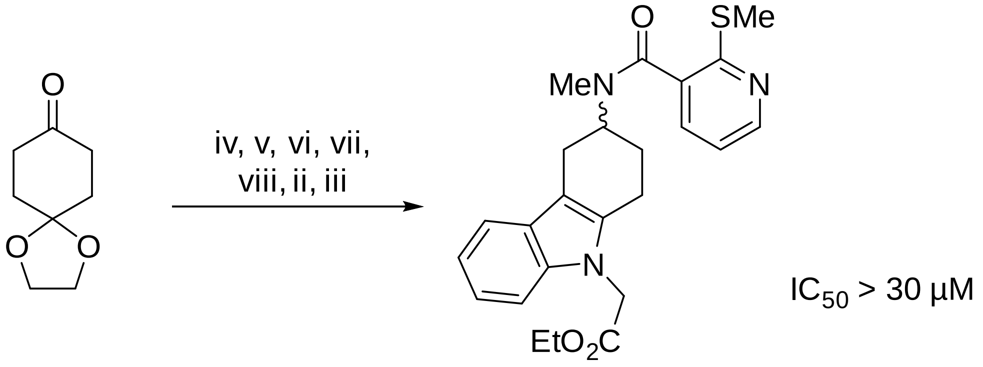

8 7

Supporting Scheme S5: Synthesis of indomorphan fragments to validate the glucose uptake activity of the combined fragments. (i) MeI, Ag_2O, CH_2Cl_2, 72 h, r.t.; (ii) H_2, Pd/C, EtOH, 5 h, r.t.; (iii) 2-(methylthio)nicotinic chloride, NEt_3, CH_2Cl_2, 12 h, r.t.; (iv) $MeNH_2$, CH_2Cl_2, M.S., 3 h, r.t. (v) $NaBH_4$, MeOH, r.t., 12 h; (vi) CbzCl, K_2CO_3, MeCN, 12 h, R.T; (vii) HCl(10%)/THF, 3 h, r.t.; ; (ix) PhN_2H_3.HCl, AcOH, reflux, 1.5 h; (viii) $BrCH_2CO_2Et$, Cs_2CO_3, DMF, 6 h, r.t.

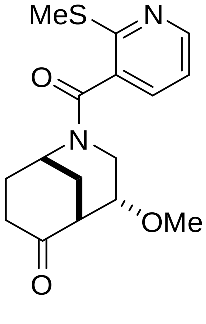

(±)-4-methoxy-2-(2-(methylthio)nicotinoyl)-2-azabicyclo[3.3.1]nonan-6-one (6):

To a solution of compound **3a** (0.046 g, 0.152 mmol) in ethanol (1.0 mL, 0.15 M), Pd/C (15mg, 100mg/mmol) was added. The atmosphere was then changed to hydrogen gas and the reaction was allowed to stir for 2 hours before filtering through celite a evaporating the solvents. The crude product was redissolved in CH_2Cl_2 (2.0 mL, 0.08M) and triethylamine (64 µL, 0.456 mmol) and 2-(methylthio)nicotinoyl chloride (57 mg, 0.304 mmol) were added. The reaction was allowed to stir overnight, solvents were evaporated and the the crude product was purified by preparative HPLC to yield compound **6** (17mg, 35%) as white solid. The product has very low solubility and was characterised only by HRMS (ESI).

HRMS (ESI)**:** calc. for $[M+H]^+$ $C_{16}H_{21}N_2O_3S$: 321.12674, found: 321.12960

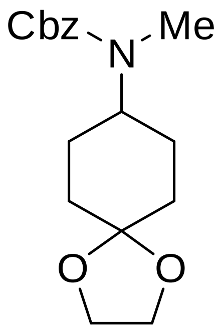

Benzyl methyl(1,4-dioxaspiro[4.5]decan-8-yl)carbamate (18):

To a solution of N-methyl-1,4-dioxaspiro[4.5]decan-8-amine(0.625 g, 3.65 mmol) in acetonitrile (24 mL, 0.155 M), potassium carbonate (1.0 g, 7.30 mmol) and benzylchloroformate (1.0 mL, 7.30 mmol) were added. The reaction was allowed to stir overnigth before evaporating the solvents. The reaction was redissolved in CH_2Cl_2 and washed with BRINE, solvents were evaporated and the crude product was purified by chromatography (0 to 7% E.A./CH_2Cl_2) to yield compound **18** (0.624 g, 56%). The product was further used without characterization at this point.

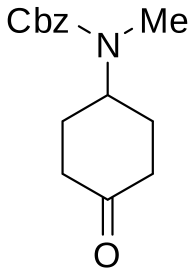

Benzyl methyl(4-oxocyclohexyl)carbamate (19):

Compound **18** (0.563 g, 1.843 mmol) was dissolved in a mixture of 10% HCl aqueous solution (25 mL, 0.075M) and THF (12 mL, 0.15M). The solution was allowed to stir for 3 hours, the product was extracted with CH_2Cl_2 and the solvents were evaporated. Crude product was purified by chromatography (30 to 50% E.A./CH_2Cl_2) to yield compound **19** (0.460 g, 95%) as a colorless oil.

^{1}H NMR (CDCl3, 500MHz): δ 1.85 (m, 2H), 1.99-2.03 (m, 2H), 2.41-2.48 (m, 4H), 2.82 (s, 3H), 4.35-4.55 (m, 1H), 5.16 (s, 2H), 7.30-7.35 (m, 1H), 7.36 (d, *J*=4.4 Hz, 3H).

^{13}C NMR (CDCl3, 100MHz): δ 28.5, 39.9, 52.9, 67.4, 128.0, 128.2, 128.6, 136.8, 156.2, 209.7

HRMS (ESI)**:** calc. for $[M+H]^+$ $C_{15}H_{20}NO_3$: 262.14377, found: 262.14392

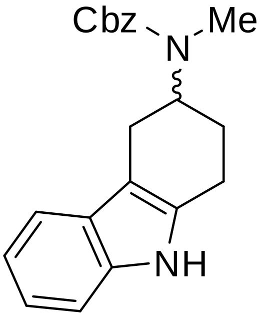

(±)-Benzyl methyl(2,3,4,9-tetrahydro-1H-carbazol-3-yl)carbamate (20):

Compound **20** (0.665 g, 86%) was synthesized from **19** following General Procedure **A**.

^{1}H NMR (CDCl3, 500MHz): δ 1.99-2.11 (m, 2H), 2.78-2.99 (m, 7H), 4.49-4.61 (m, 1H), 5.16-5.21 (m, 2H), 7.08 (t, *J*=7.3 Hz, 1H), 7.13 (t, *J*=7.2 Hz, 1H), 7.27-7.43 (m, 7H), 7.79 (bs, 1H).

^{13}C NMR (CDCl3, 100MHz): δ 23.0, 24.1, 27.3, 28.8, 52.6, 67.2, 108.8, 110.6, 117.8,119.5, 121.5, 128.0, 128.1, 128.6, 132.7, 136.4, 137.1, 156.4

HRMS (ESI)**:** calc. for $[M+H]^+$ $C_{21}H_{23}N_2O_2$: 335.17540, found: 335.17557

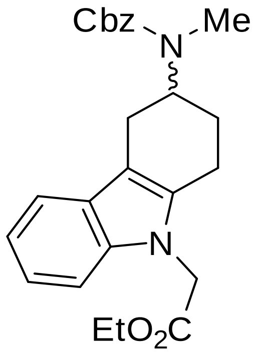

(±)-Ethyl 2-(3-(((benzyloxy)carbonyl)(methyl)amino)-1,2,3,4-tetrahydro-9H-carbazol-9-yl)acetate (21):

Compound **21** (0.370 g, 71%) was synthesized from **20** following General Procedure **B**.

^{1}H NMR (CDCl3, 400MHz): δ 1.25 (t, *J*=7.1 Hz, 3H), 2.00-2.17 (m, 2H), 2.78-3.00 (m, 7H), 4.19 (q, *J*=7.1 Hz, 2H), 4.45-4.60 (m, 1H), 4.72 (s, 2H), 5.18 (2, 2H), 7.06-7.13 (m, 1H), 7.16-7.18 (m, 2H), 7.29-7.41 (m, 5H), 7.43 (d, *J*=7.7 Hz, 1H).

ESI: calc. for $[M+H]^+$ $C_{25}H_{29}N_2O_4$: 420.2, found: 421.2; calc. for $[M+Na]^+$ $C_{25}H_{28}N_2O_4$: 443.2, found: 443.2

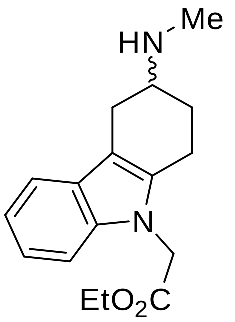

(±)-Ethyl 2-(3-(methylamino)-1,2,3,4-tetrahydro-9H-carbazol-9-yl)acetate (22):

To a solution of compound **21** (0.304 g, 0.748 mmol) in ethanol (7.5 mL, 0.1 M), Pd/C (38 mg, 50mg/mmol) and ammonium formate (0.236 g, 3.739 mmol) were added. The reaction was stir at reflux for 2 h. The reaction was filtered through celite and solvents were evaporated, the crude product was then purified by chromatography (5 to 20% MeOH/CH_2Cl_2) to yield compound **22** (0.165 g, 66%) as a formate salt.

^{1}H NMR (CDCl3, 600MHz): δ 1.25 (t, *J*=7.1 Hz, 3H), 2.07-2.14 (m, 1H), 2.39-2.41 (m, 1H), 2.67 (s, 3H), 2.75-2.89 (m, 3H), 3.22 (dd, *J*=14.6, 4.9 Hz, 1H), 3.25-3.30 (m, 1H), 3.48 (s, 1H), 4.19 (1, *J*=4.2 Hz, 2H), 4.70 (q, *J*=17.7 Hz, 2H), 7.08-7.11 (m, 1H), 7.17 (d, *J*=4.0 Hz, 2H), 7.45 (d, *J*=7.8 Hz, 1H).

^{13}C NMR (CDCl3, 100MHz): δ 14.3, 20.3, 25.2, 26.7, 31.2, 44.8, 55.5, 61.8, 106.9, 108.6 ,118.1, 119.9, 121.9, 127.2, 134.0, 137.3, 168.8

HRMS (ESI)**:** calc. for $[M+H]^+$ $C_{16}H_{21}N_2O_2$:273.15975, found: 273.15998

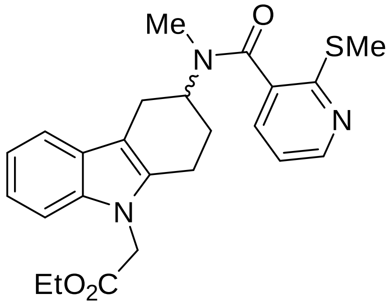

(±)-Ethyl 2-(3-(N-methyl-2-(methylthio)nicotinamido)-1,2,3,4-tetrahydro-9H-carbazol-9-yl)acetate (7):

Compound **7** (0.032 g, 67%) was synthesized from **22** following General Procedure **E**.

^{1}H NMR (CDCl3, 500MHz) (rotameric mixture 1:2.3):δ 1.22-1.28 (m, 3H), 2.05-2.27 (m, 2H), 2.53-2.71 (m, 3H), 2.77-3.10 (m, 5H), 3.15 (s, 1.3H), 2.77-3.10 (m, 5H), 3.15 (s, 1.3H), 3.47 (s, 0.7H), 3.78-3.94 (m, 0.7H), 4.15-4.23 (m, 2H), 4.65 (s, 1H), 4.74-4.79 (m, 1H), 5.13-5.19 (m, 0.3H), 6.98-7.12 (m, 4H), 7.41-7.49 (m, 2H), 8.14-8.26 (m, 0.3H), 8.41-8.49 (m, 0.7H).

HRMS (ESI)**:** calc. for $[M+H]^+$ $C_{23}H_{26}N_3O_3S$: 424.16894, found: 424.16896

Separation of (±)-Glupin racemic mixture:

(+/-)-Glupin:

Analytical HPLC Separation:(+/-)-Glupin was submitted to Chiral Analytical HPLC separation. Column: ChiralPak® IC. Eluent: 40% EtOH/60% iso-Hexane. Flowrate: 0.5mL/min. The first enantiomer to exit the column is referred as e1. E_1: T_R=54.4 min. E_2: T_R=69.9 min (see Chiral Chromatograms section).

Preparative HPLC Separation: (+/-)-Glupin (not as the TFA salt) was submitted to Chiral preparative HPLC separation. Column: ChiralPak® IC. Eluent: 40% EtOH/60% *iso*-Hexane. Flowrate: 4mL/min. The first enantiomer to exit the column is referred as e1. E_1: T_R=45.7 min. E_2: T_R=61.1 min (see Chiral Chromatogram section). Optical rotations were measured in a Schmidt + Haensch Polartronic HH8 polarimeter as chloroform solutions.

Determination of the absolute configuration of the enantiomers of the indomorphan class.

In order to determine the stereochemistry of the three chiral carbons of the morphan ring of (+)-Glupin, acetylated (S)-mandelate acid was introduced on R1 position at compound **1** (see Supporting Scheme S7). The resulting diastereoisomers were separated by chromatography and their stereochemistry determined based on the NMR shifts of the neighbouring protons, as described by Bonjoch *et al*.[Fehler! Textmarke nicht definiert.] Afterwards, the two diastereoisomers were taken separately through the Glupin synthesis pathway to generate indomorphan enantiomer analogues **26** and **26'** (the chiral information of the mandelate group is erased under the reducting conditions to remove the Cbz protecting group, see below). The glucose uptake inhibiting activity of both analogues was assayed and based on the difference of activity the stereochemistry of (+) and (-)-Glupin was assigned.

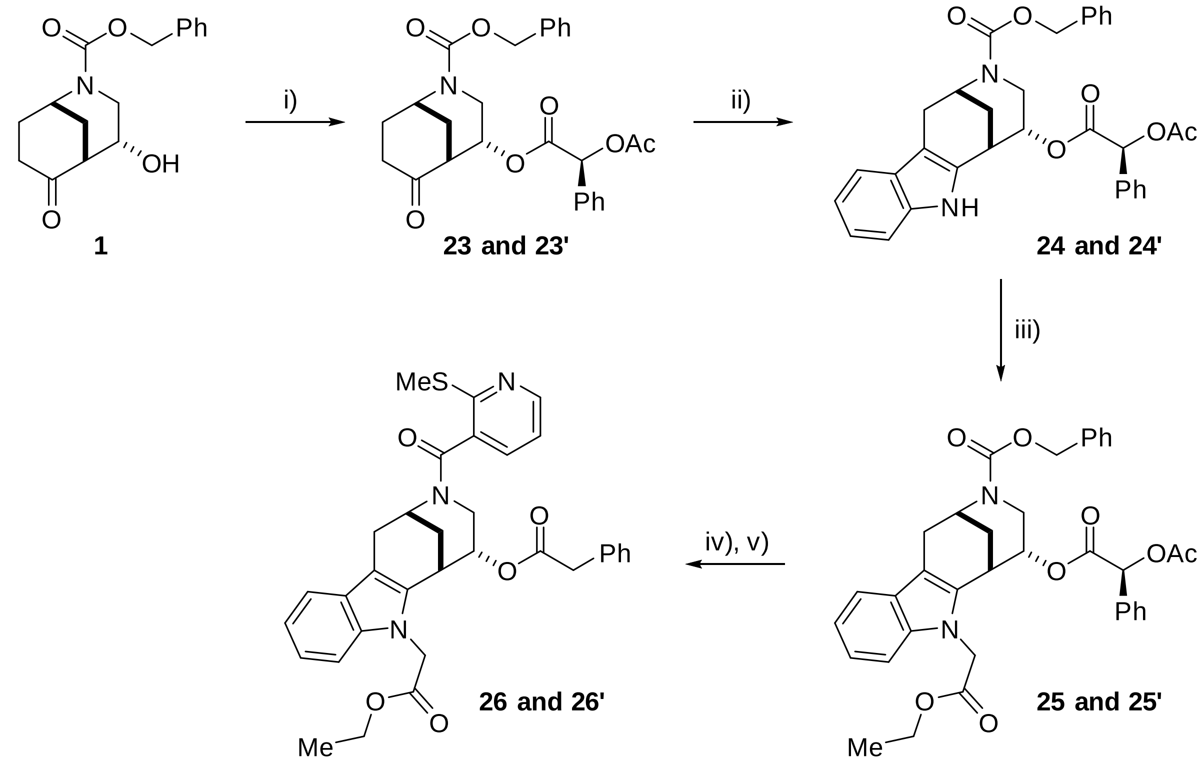

Supporting Scheme S6: Synthesis of indomorphan analogues **22** and **22'**. (i) (S)-*O*-Mandelic acid, thionyl chloride, pyridine/CH_2Cl_2, -10 ºC; (ii) PhN_2H_3.HCl, AcOH, reflux; (iii) $BrCH_2CO_2Et$, Cs_2CO_3,

DMF, 6 h, r.t.; (iv) H_2, Pd/C, EtOH, 5 h, r.t.; (vii) 2-(methylthio)nicotinic chloride, NEt_3, CH_2Cl_2, 12 h, r.t.

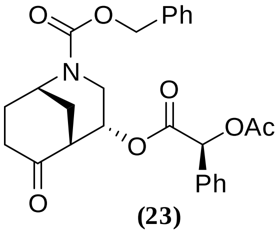

(23)

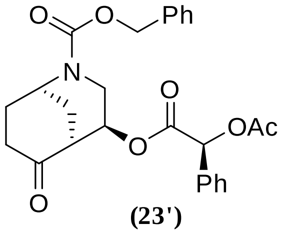

(23')

To a solution of (S)-*O*-acetyl mandelic acid (CAS number: 7322-88-5) (1.47 g, 7.57 mmol) in CH_2Cl_2 (22.3 mL, 0.34M), thionyl chloride (0.60 mL, 8.33 mmol) freshly distilled and two drops of DMF were added. The reaction was stirred at reflux conditions for 2 hours before evaporating the solvents. The crude product was redissolved in CH_2Cl_2 (20.0 mL, 0.175M) and added dropwise to a solution of racemic compound **1** (1.0 g, 3.456 mmol) in pyridine (20.0 mL, 0.175 M) kept at -10°. The reaction was allowed to stir at this temperature for 6 hours. The reaction was quenched with a saturated aqueous solution of Cu (II) and washed with this solution (6/8X) until no pyridine remained in the organic phase (determined by the color of the solution). The organic phase was finally washed with ammonium chloride saturated solution and with BRINE before evaporating solvents. Crude product was purified by chromatography (5 to 10% E.A./CH_2Cl_2) to yield compound **23** (less polar) (0.530g, 34%) and compound **23'** (more polar) (0.500g, 31%).

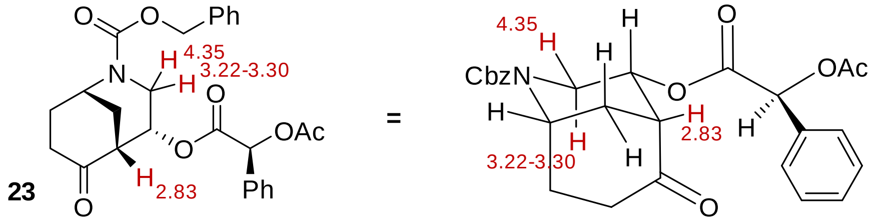

less polar (1*S*,4*R*,5*R*)

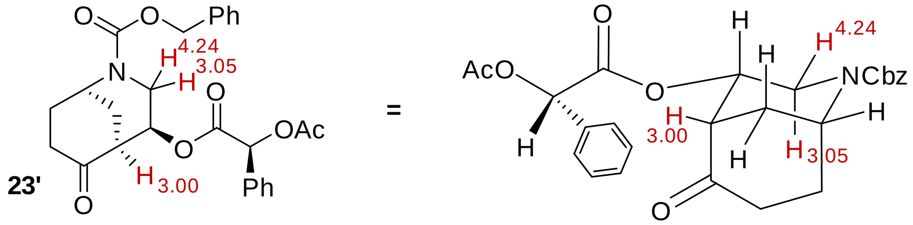

more polar (1*R*,4*S*,5*S*)

Supporting Figure S4: 1H shifts of the neighboring protons of the chiral auxiliary.

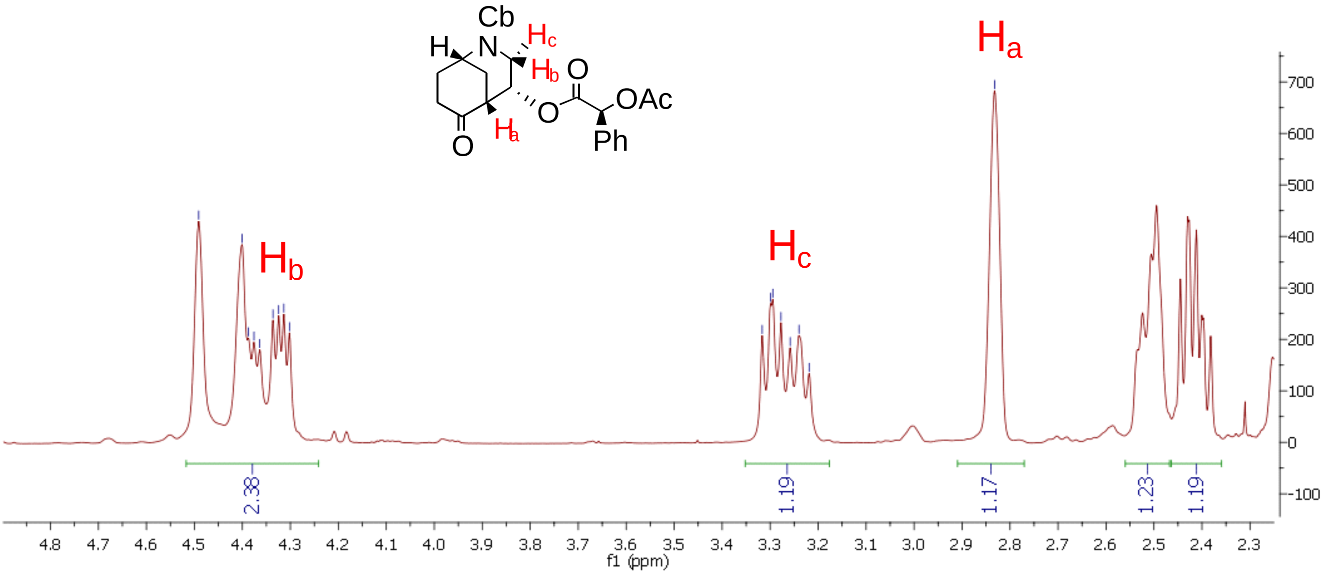
Cb
H
N
Hc
Hb
O
OAc
O
Ha
Ph
O
Ha
Hb
Hc
2.38
1.19
1.17
1.23
1.19
f1 (ppm)

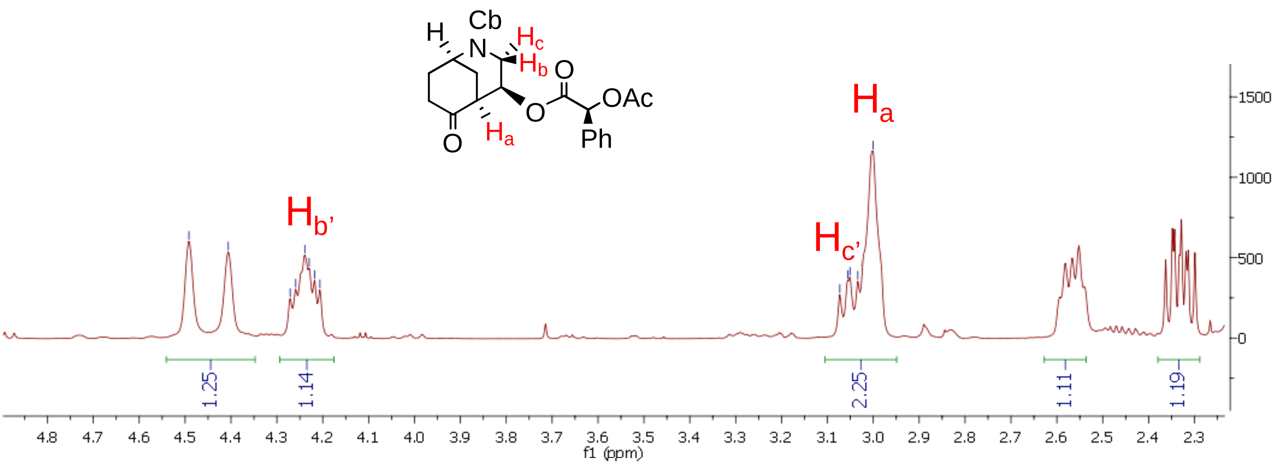
Cb
H
N
Hc
Hb
O
OAc
O
Ha
Ph
O
Ha
Hb'
Hc'
1.25
1.14
2.25
1.11
1.19
f1 (ppm)

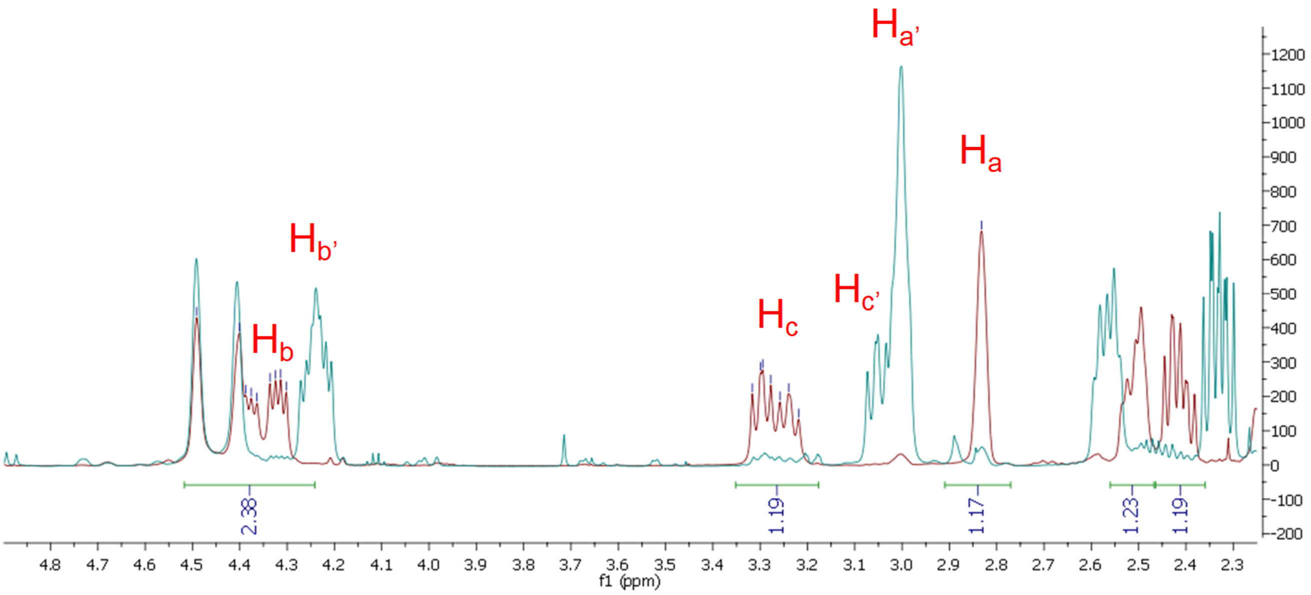
Ha'
Ha
Hb'
Hb
Hc
Hc'
2.38
1.19
1.17
1.23
1.19
f1 (ppm)

Supporting Figure S5: Comparison of the 1H spectra of compounds 23, 23’ and 23-23’ diastereomeric mixture.

Benzyl (1S,4R,5R)-4-(2-acetoxy-2-phenylacetoxy)-6-oxo-2-azabicyclo[3.3.1]nonane-2-carboxylate (less polar) (23):

^{1}H NMR (CDCl3, 600MHz) (rotameric mixture 1:1): δ 1.94-2.03 (m, 3H), 2.16-2.25 (m, 4H), 2.38-2.45 (m, 1H), 2.49-2.53 (m, 1H), 2.83 (s, 1H), 3.22-3.26 (m, 0.5H), 3.30 (dd, *J*=13.2, 10.3 Hz, 0.5H), 4.35 (ddd, *J*=37.2, 13.6, 7.1 Hz, 0.5H), 4.40 (m, 0.5H), 4.49 (s, 0.5H), 5.09-5.18 (m, 3H), 5.84 (d, *J*=11.5 Hz, 1H), 7.31-7.44 (m, 10H).

^{13}C NMR (CDCl3, 100MHz): δ 20.7, 29.7, 30.8, 30.5, 38.7, 43.6, 43.7, 44.2, 44.4, 47.1, 67.6, 67.7, 68.9, 69.2, 74.5, 127.5, 128.1, 128.3, 128.6, 128.8, 129.4, 133.0, 133.1, 136.3, 155.6, 167.7, 167.9, 170.4, 170.5, 207.1, 207.2

HRMS (ESI)**:** calc. for $[M+H]^+$ $C_{26}H_{28}NO_7$: 466.18603, found: 466.18568

Benzyl (1R,4S,5S)-4-((S)-2-acetoxy-2-phenylacetoxy)-6-oxo-2-azabicyclo[3.3.1]nonane-2-carboxylate (more polar) (23’):

^{1}H NMR (CDCl3, 600MHz) (rotameric mixture 1:1): δ 1.96-2.04 (m, 3H), 2.16-2.22 (m, 4H), 2.33 (ddd, *J*=17.8, 11.7, 8.9 Hz, 1H), 2.54-2.59 (m, 1H), 3.00 (bs, 1.5H), 3.05 (dd, *J*=13.3, 10.3 Hz, 0.5H), 4.24 (m, 1H), 4.41 (s, 0.5H), 4.49 (s, 0.5H), 5.08-5.17 (m, 3H), 5.90 (s, 1H), 7.31-7.36 (m, 8H), 7.40-7.41 (m, 2H).

^{13}C (CDCl3, 100MHz): δ 20.7, 29.6, 30.4, 30.5, 30.7, 38.7, 38.8, 43.3, 43.4, 44.2, 44.3, 47.1, 47.1, 67.6,

67.7, 69.0, 69.2, 74.3, 74.3, 127.7, 128.1, 128.3, 128.3, 128.6, 128.9, 129.4, 133.5, 136.3, 155.3, 155.4, 167.7, 167.8, 170.1, 170.2, 207.4, 207.6

HRMS (ESI)**:** calc. for $[M+H]^+$ $C_{26}H_{28}NO_7$: 466.18603, found: 466.18581

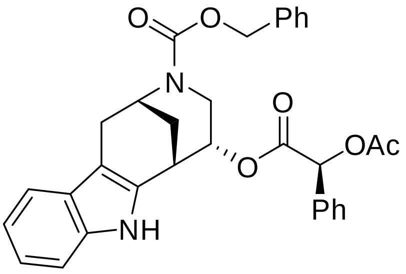

Benzyl (2R,5R,6S)-5-((S)-2-acetoxy-2-phenylacetoxy)-1,2,4,5,6,7-hexahydro-3H-2,6-methanoazocino[5,4-b]indole-3-carboxylate (24):

Compound **24** (0.424 mg, 67%) was synthesized from **23** following General Procedure **A**.

^{1}H NMR (CDCl3, 500MHz) (rotameric mixture 1:1): δ 2.02-2.06 (m, 1H), 2.10-2.12 (m, 1H), 2.35 (d, *J*=5.2 Hz, 3H), 2.83-2.93 (m, 2H), 3.15-3.21 (m, 1H), 3.69-3.72 (m, 1H), 3.90 (dd, *J*=12.7, 5.7 Hz, 0.5H), 4.03 (dd, *J*=12.8, 5.7 Hz, 0.5H), 4.78 (bs, 0.5H), 4.86 (bs, 0.5H), 4.95-4.99 (m, 1H), 5.13 (s, 1H), 5.16-5.22 (m, 1H), 5.61 (d, *J*=8.6 Hz, 1H), 7.13 (t, *J*=7.5 Hz, 1H), 7.21 (t, *J*=7.6 Hz, 1H), 7.29-7.45 (m, 11H), 7.52 (t, *J*=8.9 Hz, 1H), 9.34 (s, 0.5H), 9.38 (s, 0.5H).

^{13}C (CDCl3, 100MHz): δ 21.0, 27.6, 27.9, 29.6, 30.0, 30.9, 31.0, 40.7, 40.8, 45.0, 45.1, 67.4, 67.6, 72.5, 72.7, 75.7, 75.8, 109.0, 109.2, 111.3, 111.3, 118.0, 118.1, 119.2 121.5, 121.5, 126.6, 126.6, 127.4, 127.8, 128.1, 128.1, 128.2, 128.3, 128.6, 128.7, 129.1, 129.8, 129.9, 131.3, 131.4, 132.0, 132.1, 136.2, 136.2

HRMS (ESI)**:** calc. for $[M+H]^+$ $C_{32}H_{31}N_2O_6$: 539.21766, found: 539.21778

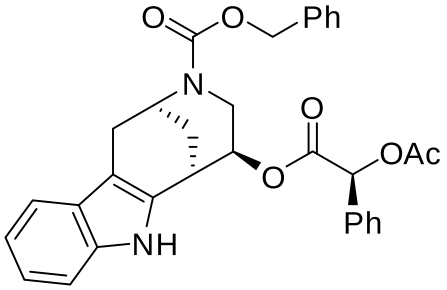

Benzyl (2S,5S,6R)-5-((S)-2-acetoxy-2-phenylacetoxy)-1,2,4,5,6,7-hexahydro-3H-2,6-methanoazocino[5,4-b]indole-3-carboxylate (24‘):

Compound **24’** (0.473 mg, 93%) was synthesized from **23’** following General Procedure **A**.

^{1}**H NMR** (CDCl3, 500MHz) (rotameric mixture 1:1): δ 1.98 (t, *J*=14.2 Hz, 1H), 2.08-2.10 (m, 1H), 2.18 (d, *J*=8.5 Hz, 3H), 2.76-2.84 (m, 2H), 3.07 (dd, *J*=17.1, 5.4 Hz, 1H), 3.24 (bs, 0.5H), 3.32 (bs, 0.5H), 4.02 (dd, *J*=12.5, 5.5 Hz, 0.5H), 4.11-4.16 (m, 0.5H), 4.74 (bs, 0.5H), 4.81 (bs, 0.5H), 5.12-5.23 (m, 3H), 5.86 (s, 1H), 6.55 (s, 0.5H), 7.00 (s, 0.5H), 7.07-7.17 (m, 3H), 7.32-7.44 (m, 6H), 7.52-7.68 (m, 5H).

^{13}**C NMR** (CDCl3, 100MHz): δ 20.9, 21.0, 27.6, 28.0, 30.0, 30.4, 31.7, 31.7, 41.1, 41.2, 44.9, 45.0, 67.5, 67.6, 72.1, 72.1, 74.6, 75.0, 109.5, 109.8, 110.9, 118.0, 118.1, 119.4, 121.7, 121.7, 126.5, 126.6, 128.1, 128.1, 128.2, 128.2, 128.3, 128.4, 128.7, 128.7, 129.5, 129.6, 130.1, 130.1, 130.4, 130.6, 133.9, 135.7, 135.8, 136.5, 136.7, 155.4, 155.5, 167.9, 167.9, 171.2, 171.7

HRMS (ESI)**:** calc. for $[M+H]^+$ $C_{32}H_{31}N_2O_6$: 539.21766, found: 539.21784

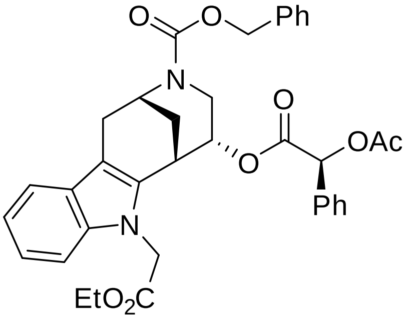

Benzyl (2R,5R,6S)-5-(2-acetoxy-2-phenylacetoxy)-7-(2-ethoxy-2-oxoethyl)-1,2,4,5,6,7-hexahydro-3H-2,6-methanoazocino[5,4-b]indole-3-carboxylate (25):

Compound **25** (0.022 mg, 19%) was synthesized from **24** following General Procedure **B**, and purified by preparative HPLC from a complicated mixture of products and characterized only by HRMS (ESI) before proceeding with the synthesis.

HRMS (ESI)**:** calc. for $[M+H]^+$ $C_{36}H_{37}N_2O_8$:625.25444, found: 625.25516;calc. for $[M+Na]^+$ $C_{36}H_3N_2O_8Na$: 647.23639, found: 647. 23724

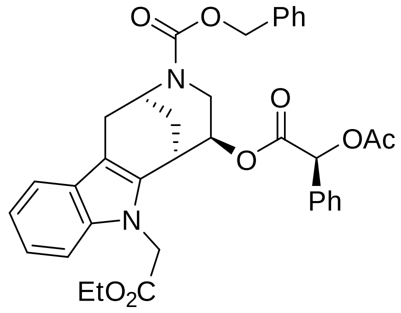

Benzyl (2S,5S,6R)-5-((S)-2-acetoxy-2-phenylacetoxy)-7-(2-ethoxy-2-oxoethyl)-1,2,4,5,6,7-hexahydro-3H-2,6-methanoazocino[5,4-b]indole-3-carboxylate (25’):

Compound **25’** (0.022 mg, 19%) was synthesized from **24’** following General Procedure **B**, and purified by preparative HPLC from a complicated mixture of products and characterized only by HRMS (ESI) before proceeding with the synthesis.

HRMS (ESI)**:** calc. for $[M+H]^+$ $C_{36}H_{37}N_2O_8$: 625.25444, found: 625.25516;calc. for $[M+Na]^+$ $C_{36}H_3N_2O_8Na$: 647.23639, found: 647. 23724

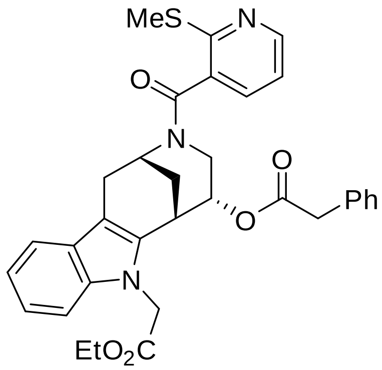

(2R,5R,6S)-7-(2-ethoxy-2-oxoethyl)-3-(2-(methylthio)nicotinoyl)-2,3,4,5,6,7-hexahydro-1H-2,6-methanoazocino[5,4-b]indol-5-yl 2-phenylacetate (26):

Compound **26** (0.005 mg, 24%) was synthesised from **25** following General Procedures **E** and **D**, without the need for purification of the intermediate product. Compound **26** was purified by preparative HPLC (0 to 100% ACN/H_2O with 0.1% of TFA).

^{1}H NMR (CDCl3, 700MHz) (rotameric mixture 1:1): δ 1.21-1.23 (m, 3H), 2.09-2.29 (m, 2H), 2.55-2.63 (m, 3H), 2.76-3.18 (m, 2.5H), 3.36 (ddd, *J*=23.7, 15.2 5.7 Hz, 1H),3.41 (d, *J*=2.44 Hz, 0.5H), 3.45-3.54 (m, 2H), 3.60 (d, *J*=15.0 Hz, 0.5H), 3.66 (d, *J*=15.0 Hz, 0.5H), 4.03 (bs, 0.5H), 4.06-4.17 (m, 2H), 4.34 (d, *J*=23.1 Hz, 2H), 4.73 (dd, *J*=13.0, 5.3 Hz, 0.5H), 5.25 (bs, 0.5H), 5.46 (s, 0.5H), 7.05-7.24 (m, 6H), 7.28-7.55 (m, 5H), 8.47 (dd, *J*=4.9, 1.6 Hz, 0.5H), 8.53 (dd, *J*=4.9, 1.7 Hz, 0.5H).

HRMS (ESI)**:** calc. for $[M+H]^+$ $C_{33}H_{34}N_3O_5$: 584.22137, found: 584.22218

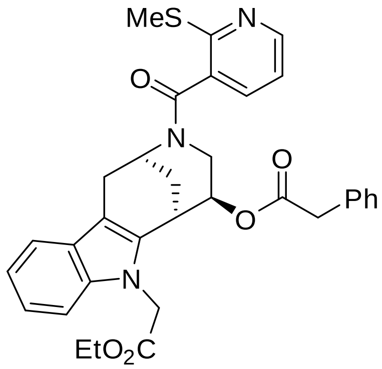

(2S,5S,6R)-7-(2-ethoxy-2-oxoethyl)-3-(2-(methylthio)nicotinoyl)-2,3,4,5,6,7-hexahydro-1H-2,6-methanoazocino[5,4-b]indol-5-yl 2-phenylacetate (26'):

Compound **26'** (0.005 mg, 24%) was synthesised from **25'** following General Procedures **E** and **D**, without the need for purification of the intermediate product. Compound **26'** was purified by preparative HPLC (0 to 100% ACN/H_2O with 0.1% of TFA).

^{1}H NMR (CDCl3, 700MHz) (rotameric mixture 1:1): δ 1.21-1.23 (m, 3H), 2.04-2.29 (m, 2H), 2.55-2.63 (m, 3H), 2.76-3.18 (m, 2.5H), 3.36 (ddd, *J*=23.6, 15.1, 5.7 Hz, 1H),3.41 (d, *J*=2.4 Hz, 0.5H), 3.45-3.54 (m, 2H), 3.60 (d,*J*=15.0 Hz, 1H), 4.03 (bs, 0.5H), 4.06-4.16 (m, 2H), 4.34 (d, *J*=23.5 Hz, 1H), 4.73 (dd, *J*=12.9, 5.3 Hz, 0.5H), 5.25 (bs, 0.5H), 5.46 (s, 0.5H), 7.06-7.24 (m, 6H), 7.28-7.55 (m, 5H), 8.48 (dd, *J*=4.9, 1.6 Hz, 0.5H), 8.53 (dd, *J*=4.9, 1.7 Hz, 0.5H).

HRMS (ESI)**:** calc. for $[M+H]^+$ $C_{33}H_{34}N_3O_5$: 584.22137, found: 584.22197

Determination of the activity of the separated enantiomers 26 and 26'

MDA-MB-231 cells were grown in high glucose Dulbecco's modified Eagle's medium (DMEM, PAN Biotech) supplemented with 10% fetal calf serum (FCS, Invitrogen-Gibco) with 1% non-essential amino acids (PAN Biotech) and 1% Penicillin/Streptomycin (PAN Biotech). Cells were seeded in black CellBIND 96-well plates with clear bottom (Corning) and allowed to attach overnight. Cells were washed three times with KRB buffer (20 mM Hepes, 5 mM KH_2PO_4, 1 mM $MgCl_2$, 1 mM $CaCl_2$, 136 mM NaCl, 4.7 mM KCl, pH 7.4) containing 0.1% BSA and then incubated with the compounds or vehicle control and 1 mM 2DG for 30 min as described earlier.[6] After washing three times with KRB buffer, the plates were sealed with adhesive aluminum foil and cells were lysed with 60 µL 1% CHAPS in 0.06 M HCl. Subsequently, plates were heated using microplate heater for 15 min at 65°C. After cooling down to room temperature, 20 µL 0.5 M Tris were added to neutralize the acid, before 80 µL of detection enzyme mixture were added. The detection enzyme solution contained 25 µM resazurin sodium salt (Acros), 6

U/mL glucose-6-phosphate dehydrogenase (G8404; Sigma Aldrich), 0.2 U/mL diaphorase from *Chlostridium kluyveri* (D5540; Sigma Aldrich) and 0.1mM $NADP^+$ (Applichem) in 0.125 mM Tris, pH 8.4 with 0.1% BSA. After incubation for 2 h at room temperature, fluorescence intensity was measured at ex/em 530/590 nm using a Tecan M200 plate reader. Data were normalized to the respective DMSO control (which was set to 100 %) and cells without 2DG (set to 0 %) using Graph Pad Prism 5 or 6 software and IC_{50} values were determined using a four-parameter fit.

Activities: 10 ± 1.0 µM (**26**) and 0.30 ± 0.12 µM (**26’**)

Based on the activities and known structures of compounds **26** and **26’**, we assigned the absolute configuration of the chiral centers in the Glupin enantiomers (+) and (-).

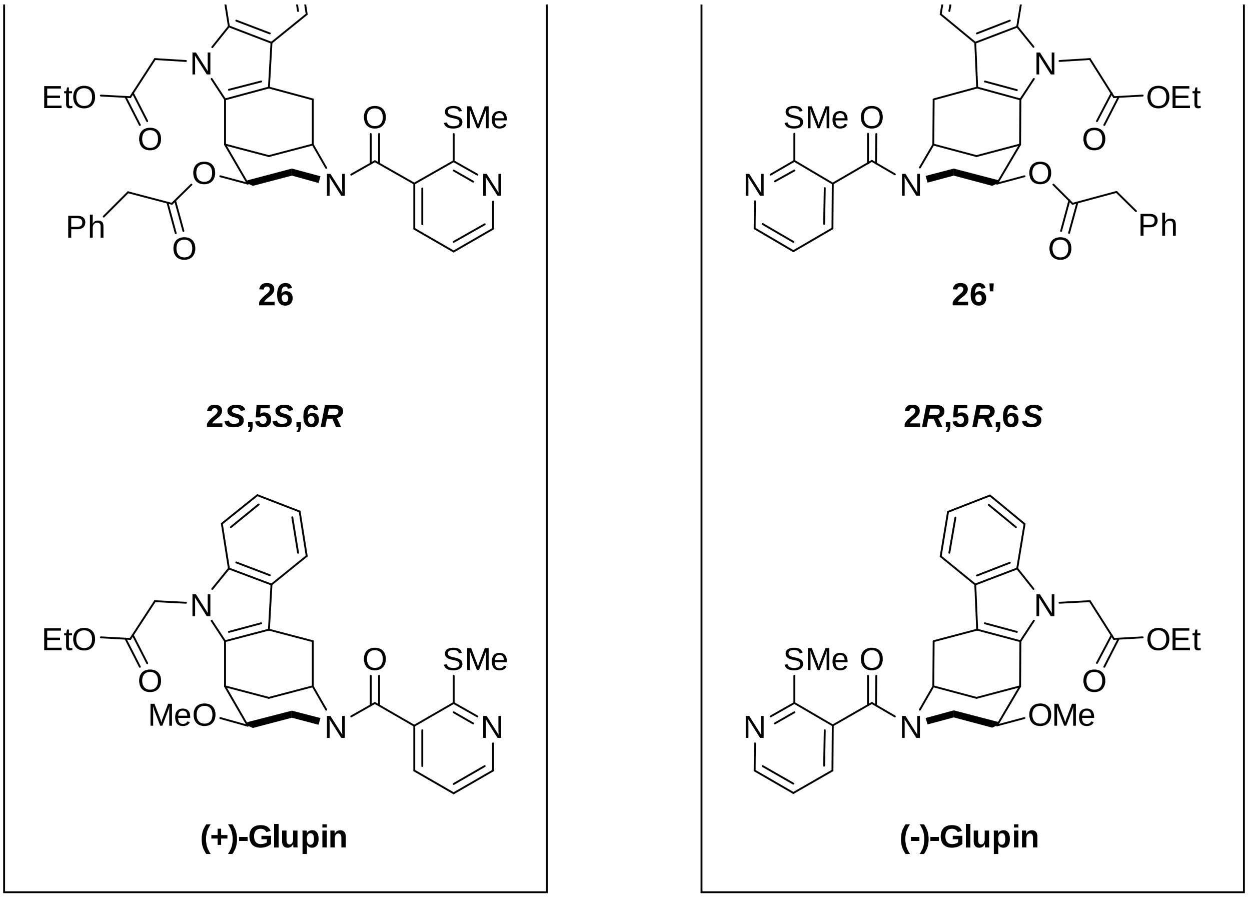

Supporting Figure S6: Absolute configuration of the enantiomers 26 and 26’ and (+)-Glupin and (-)-Glupin.

NMR Spectra:

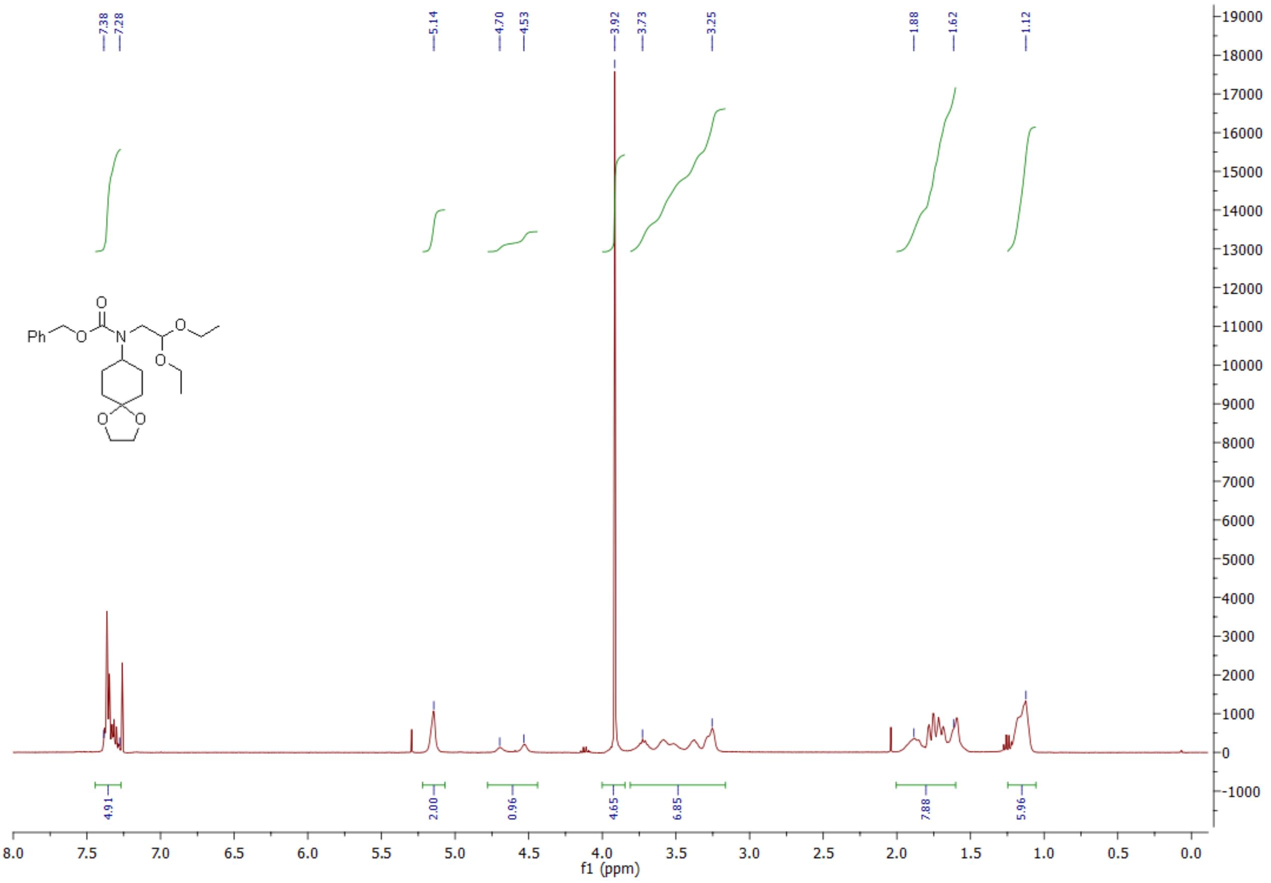
7.38
7.28
5.14
4.70
4.53
3.92
3.73
3.25
1.88
1.62
1.12
Ph
O
N
4.91
2.00
0.96
4.65
6.85
7.88
5.96
f1 (ppm)

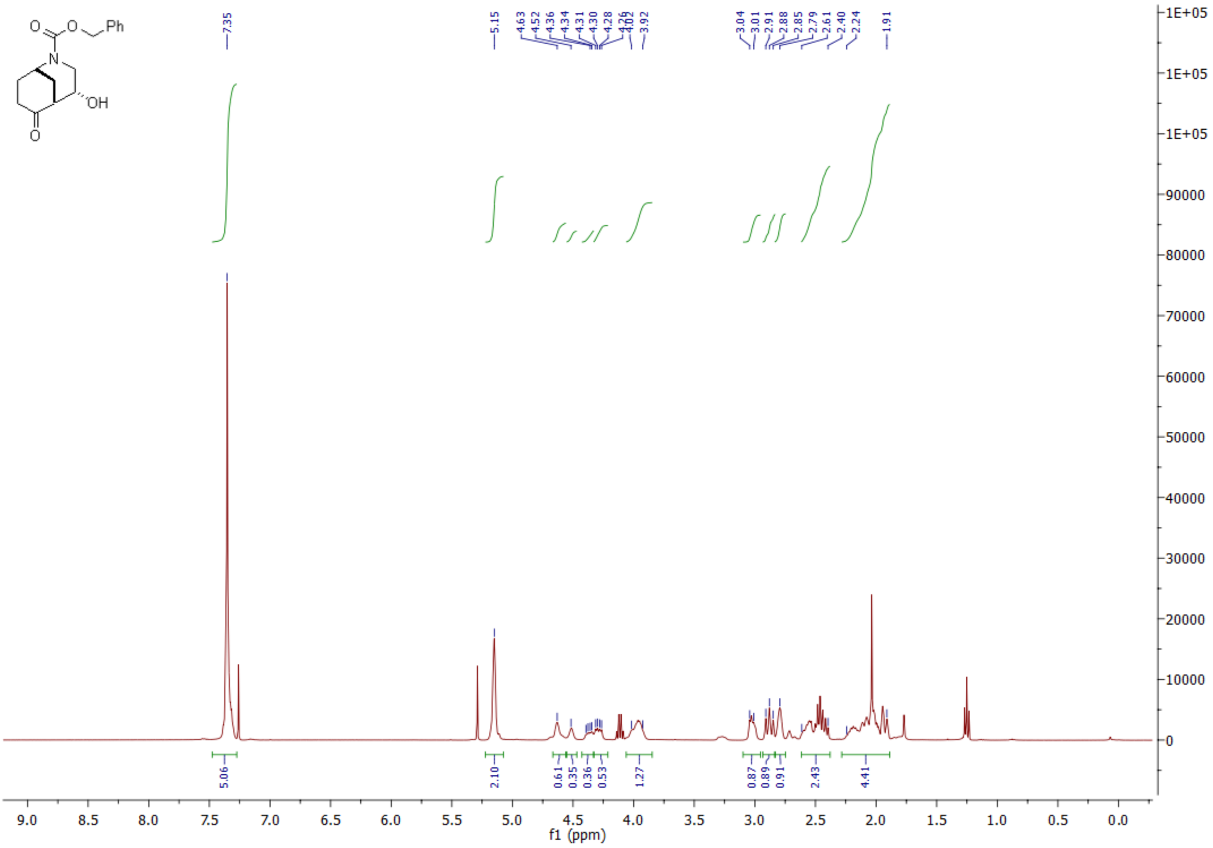
Ph
OH
7.35
5.15
4.63
4.52
4.36
4.34
4.31
4.30
4.28
3.92
3.04
3.01
2.91
2.88
2.85
2.79
2.61
2.40
2.24
1.91
5.06
2.10
0.61
0.35
0.36
0.53
1.27
0.87
0.89
0.91
2.43
4.41
f1 (ppm)

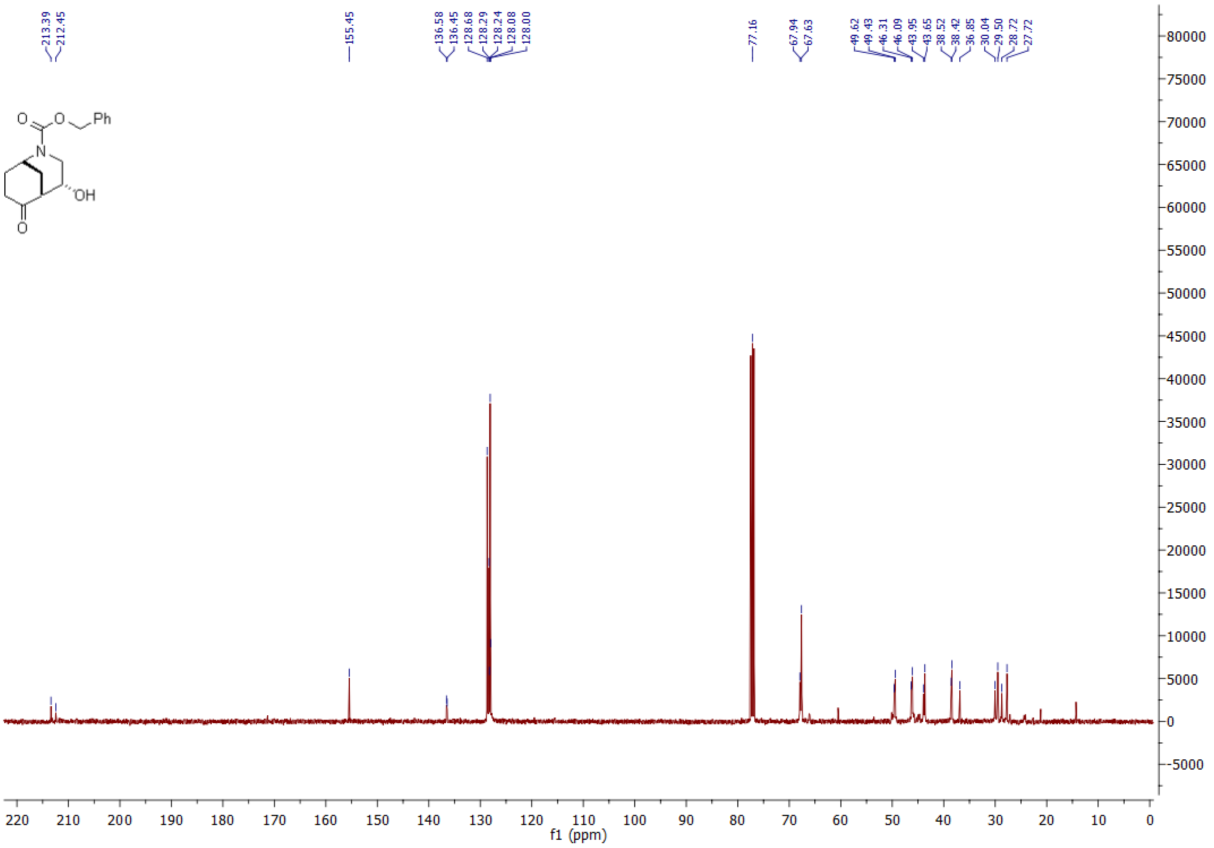
213.39
212.45
155.45
136.58
136.45
128.68
128.29
128.24
128.08
128.00
77.16
67.94
67.63
49.62
49.43
46.31
46.09
43.95
43.65
38.52
38.42
36.85
30.04
29.50
28.72
27.72
Ph
OH
f1 (ppm)

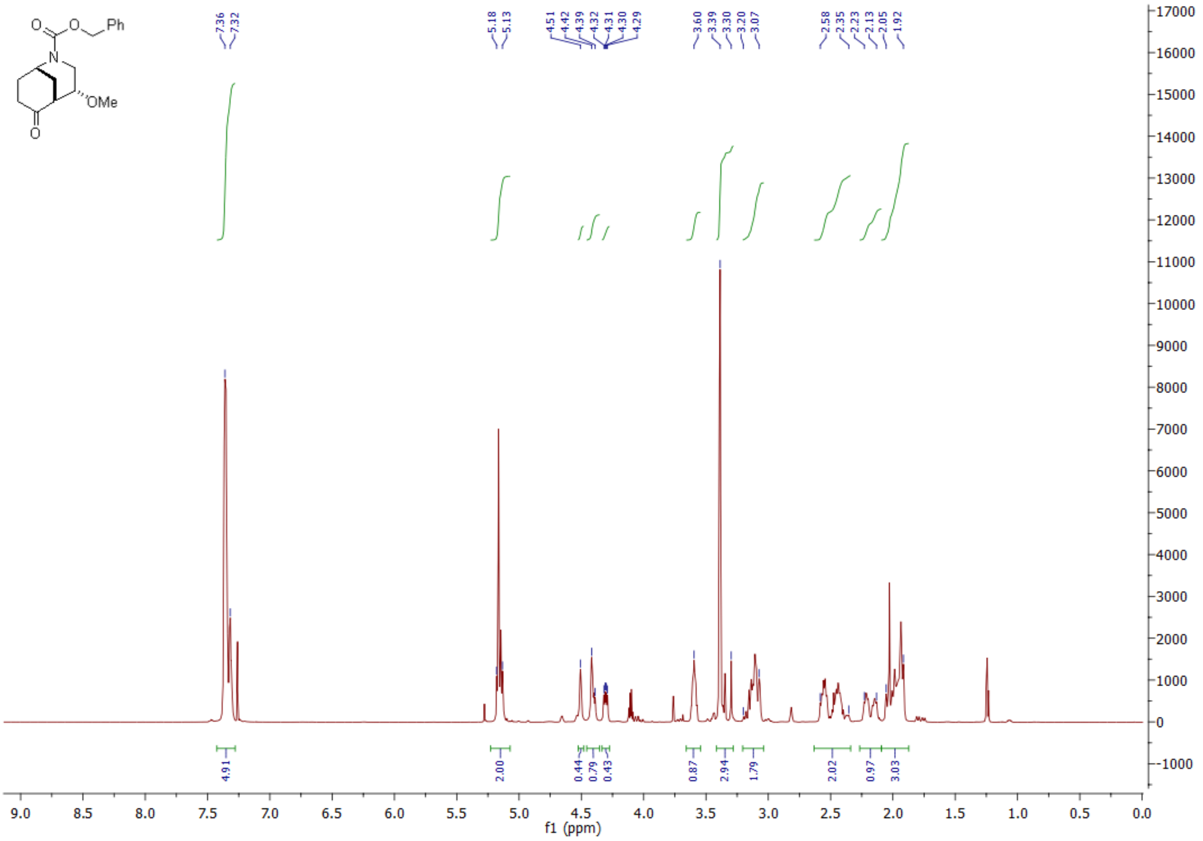
OMe
Ph
f1 (ppm)

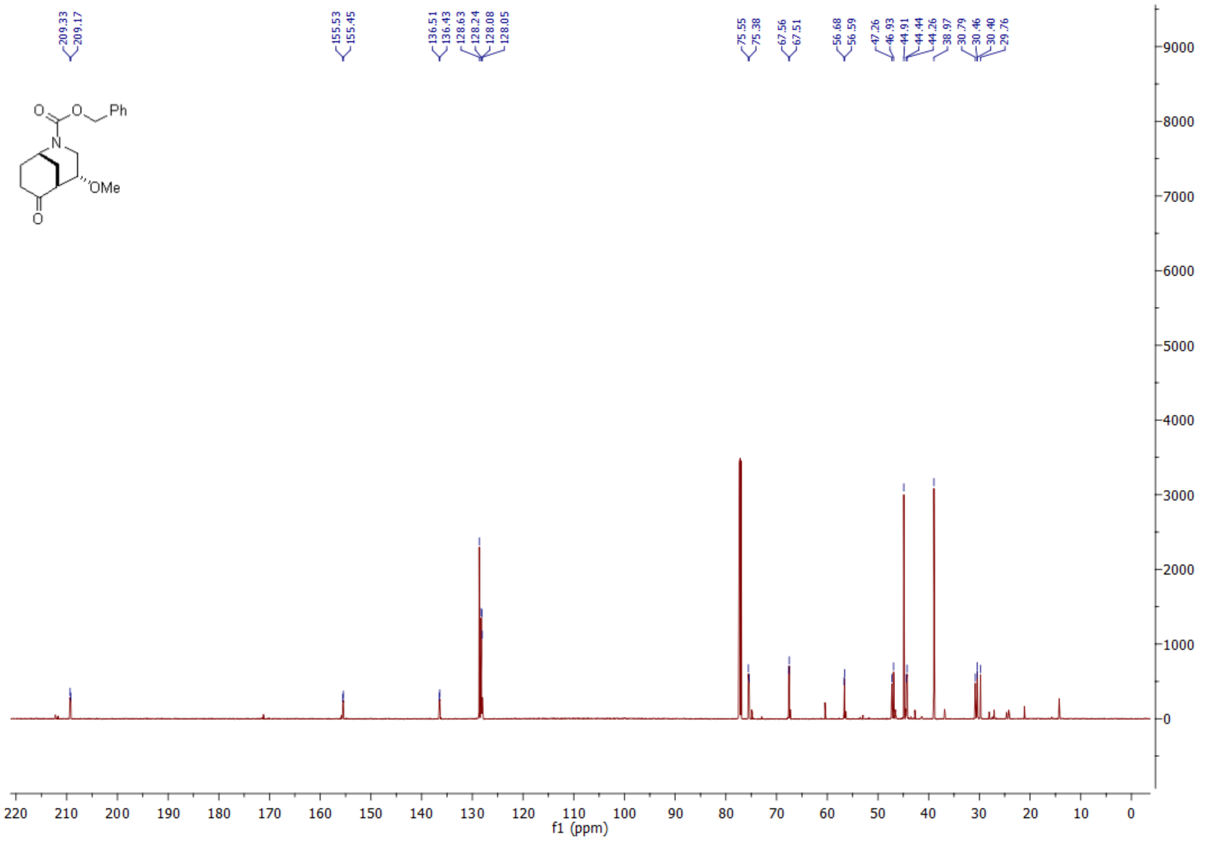
OMe
Ph
f1 (ppm)

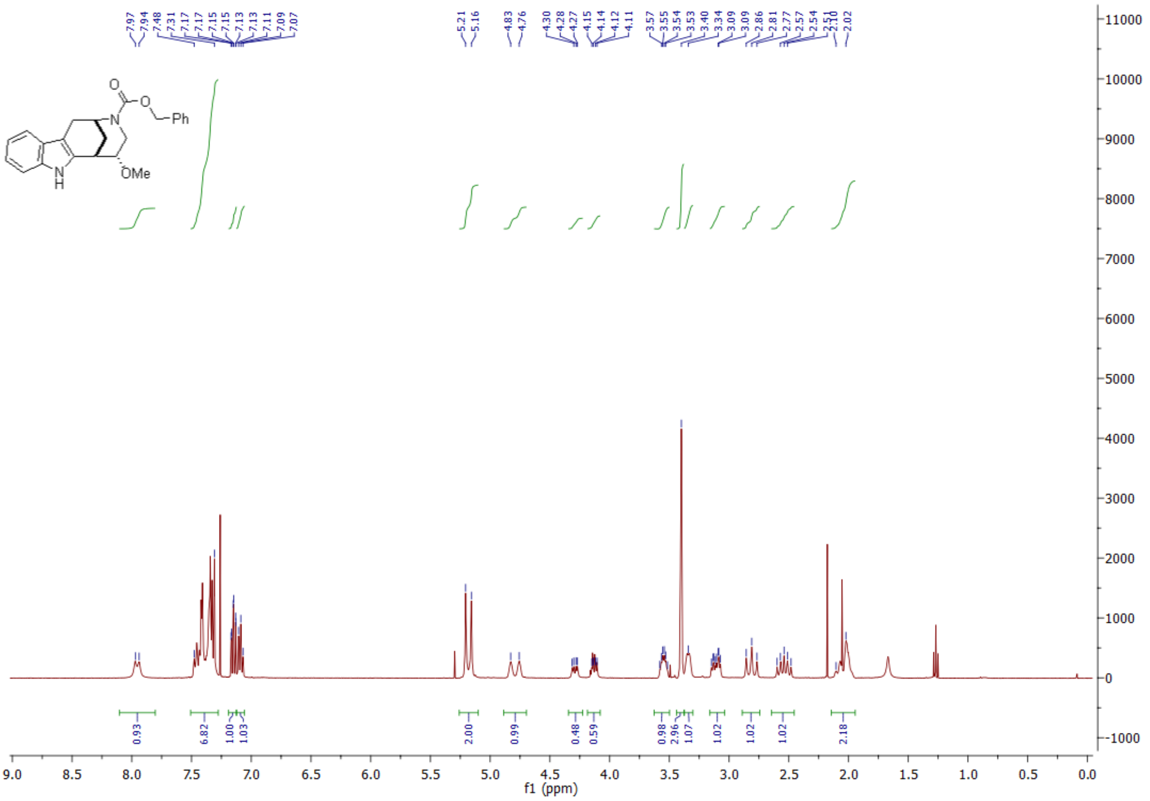
OMe
Ph
f1 (ppm)

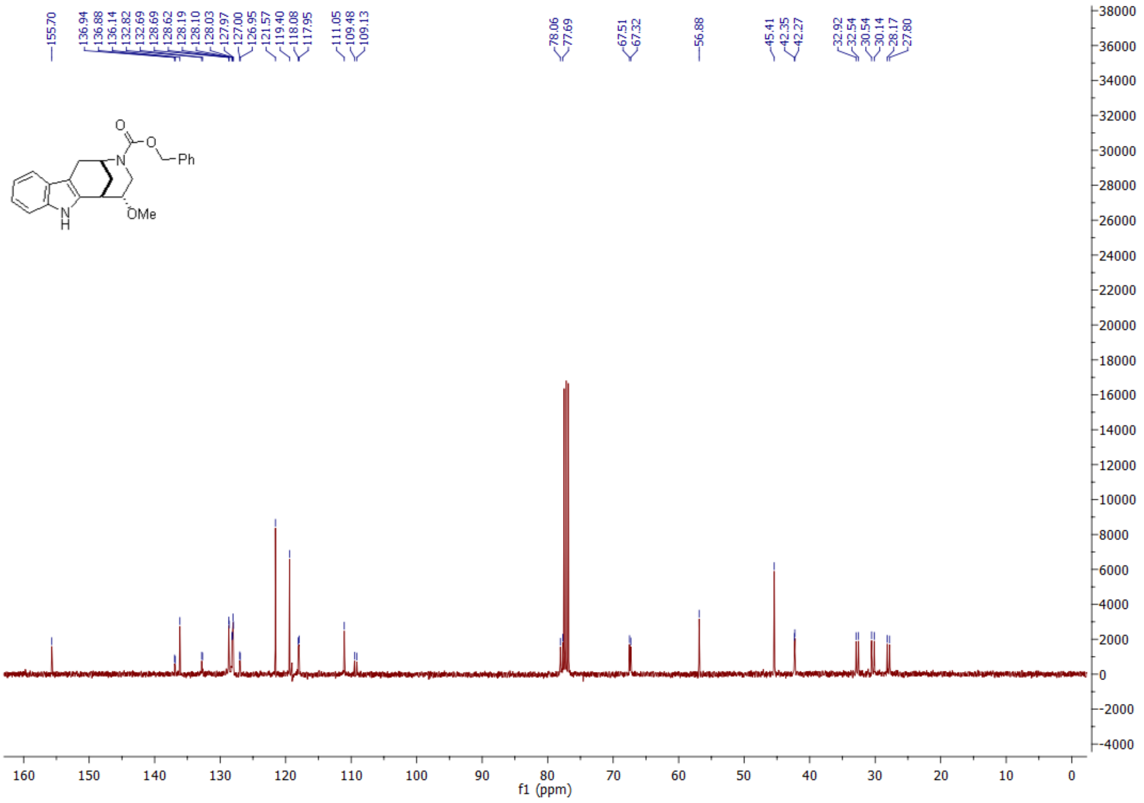
OMe
Ph
f1 (ppm)

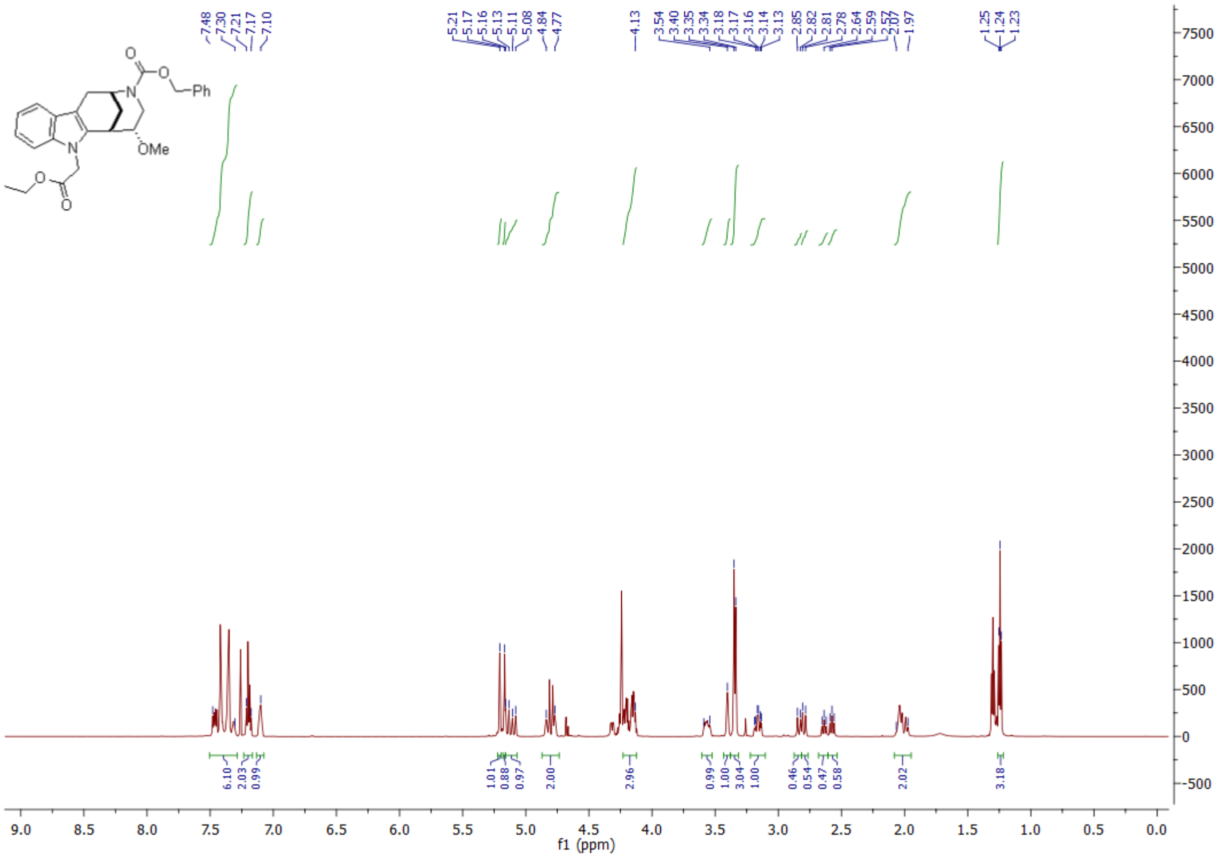

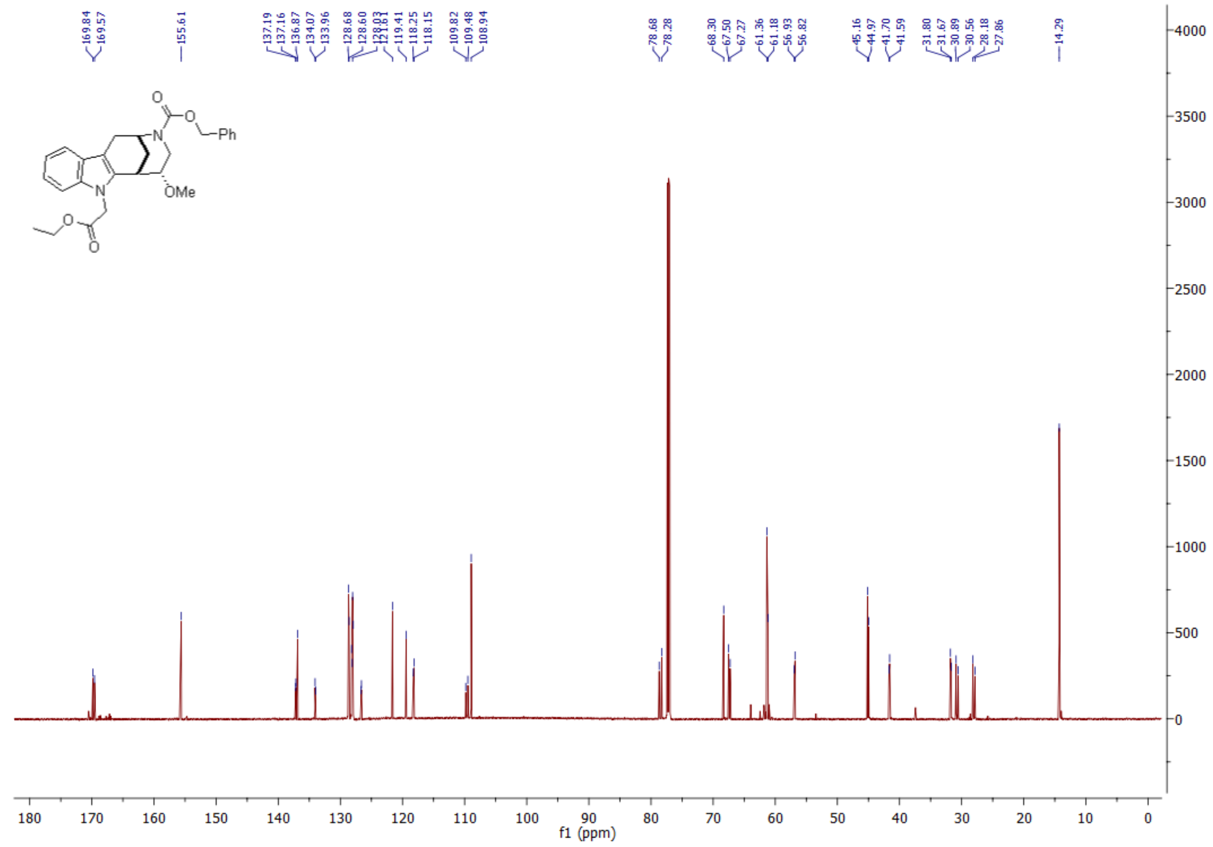

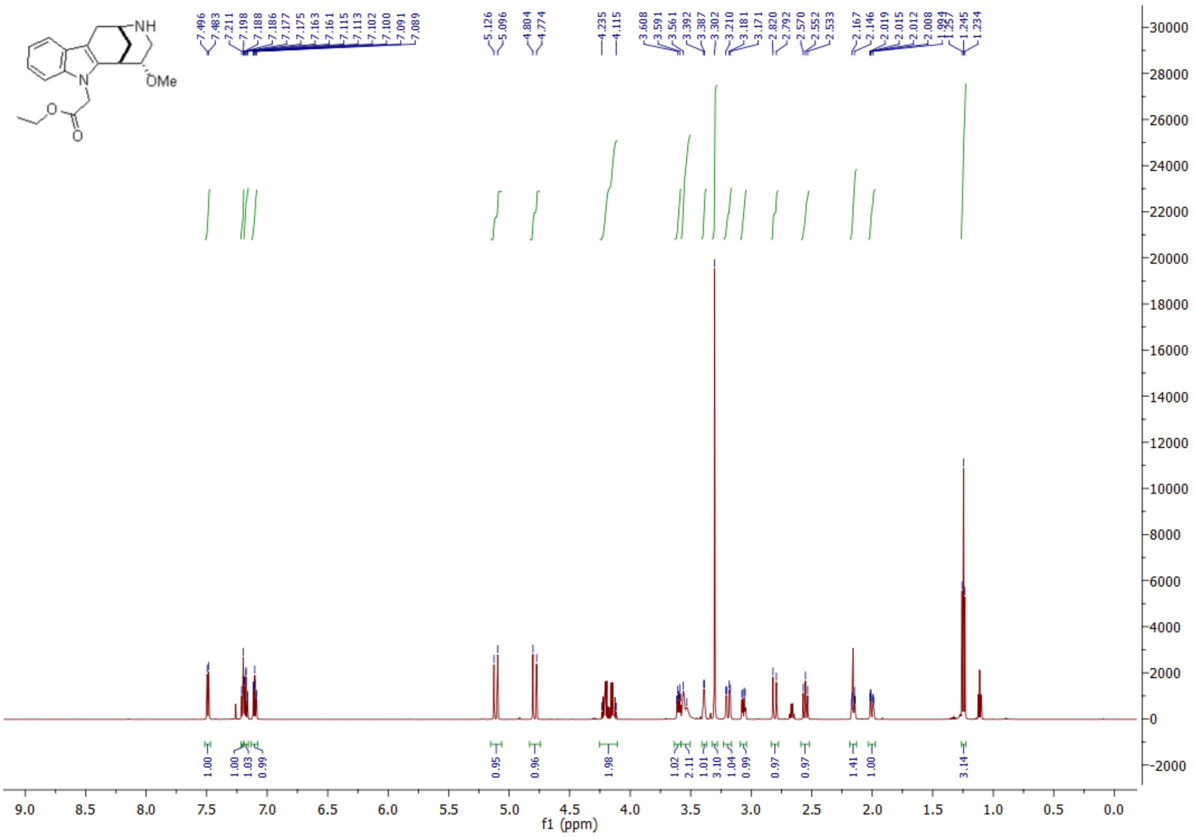

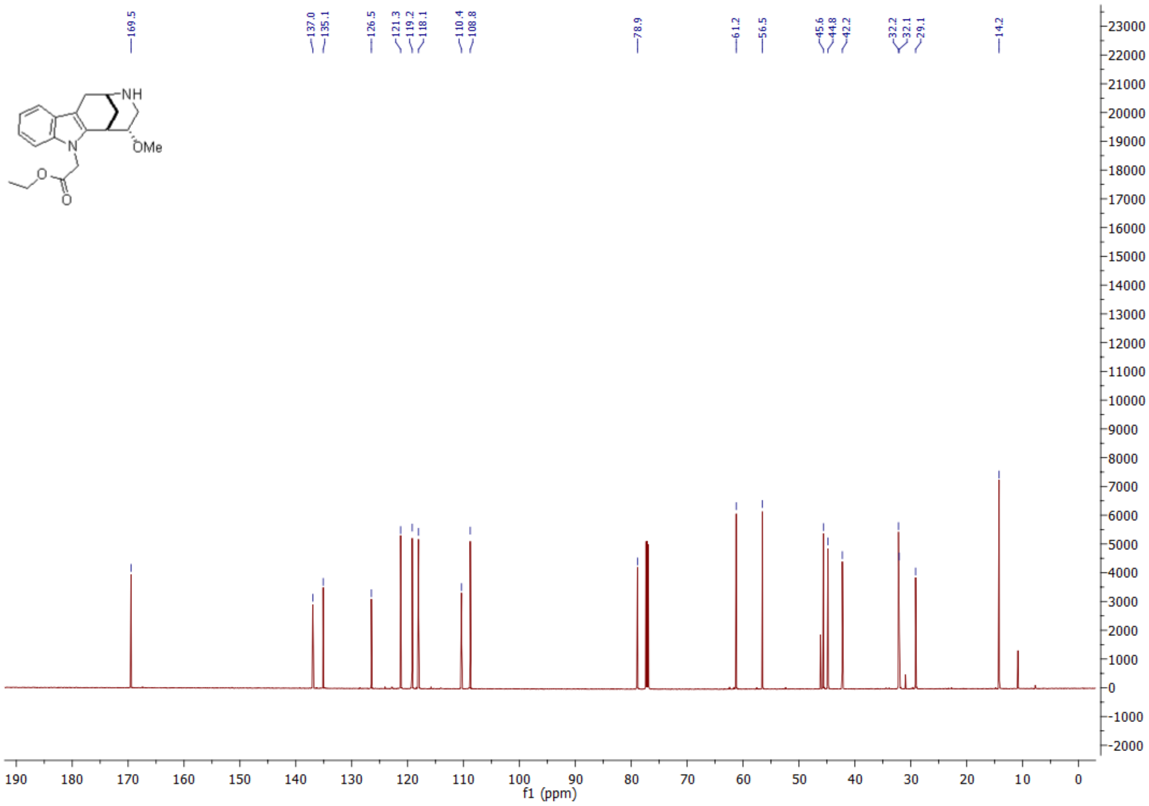

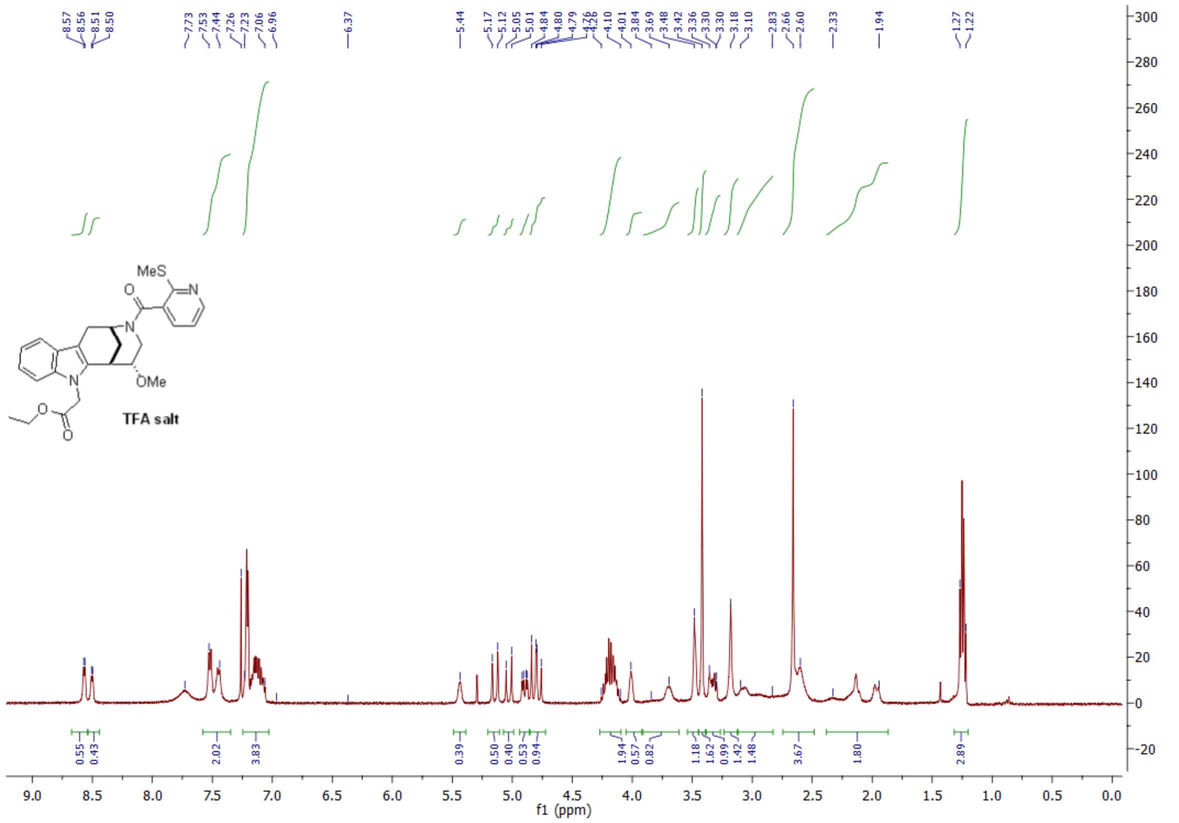
MeS
N
O
OMe
TFA salt
f1 (ppm)

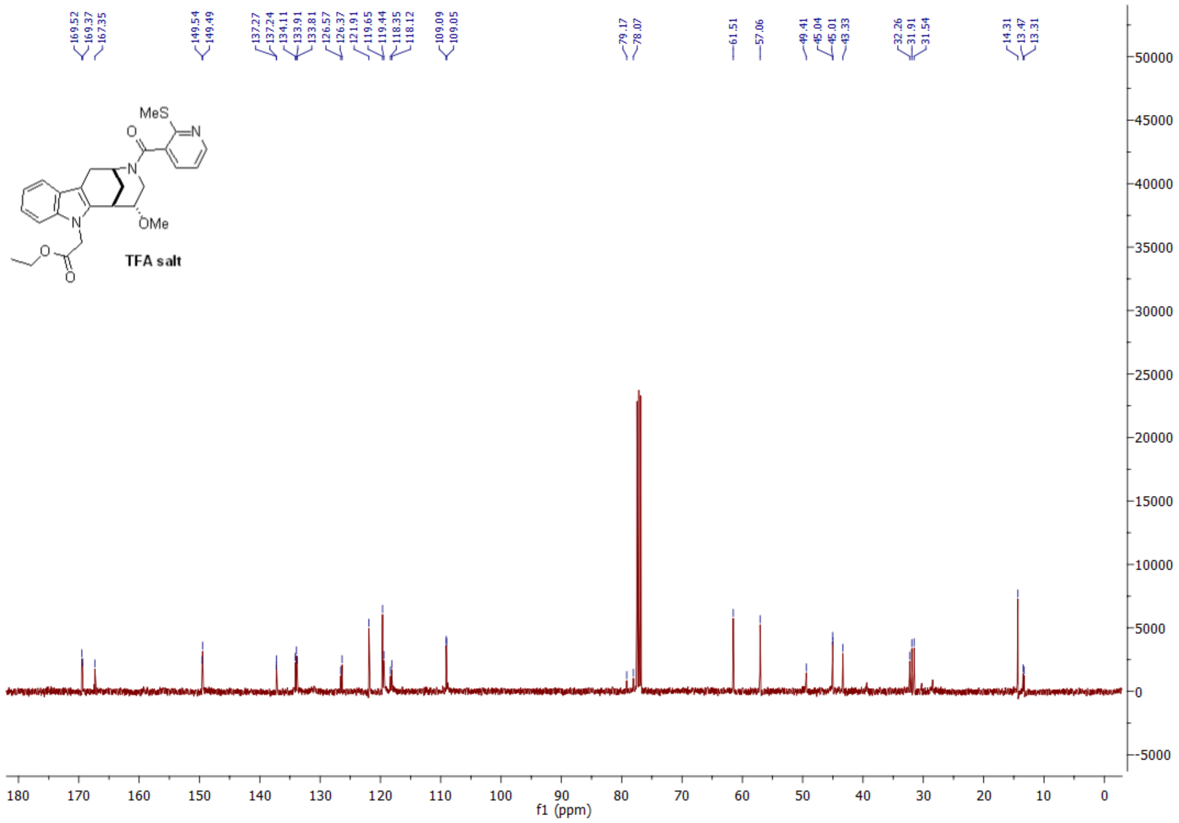
MeS
N
O
OMe
TFA salt
f1 (ppm)

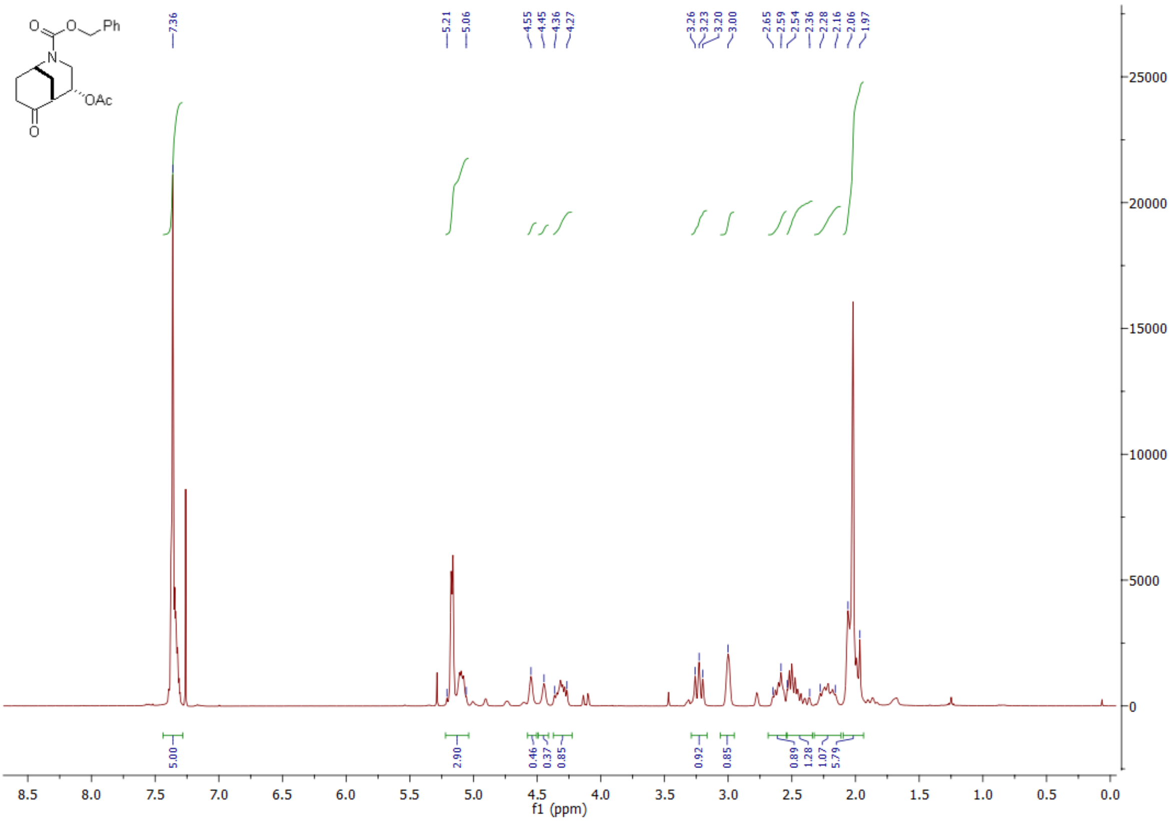
7.36
5.21
5.06
4.55
4.45
4.36
4.27
3.26
3.23
3.20
3.00
2.65
2.59
2.54
2.36
2.28
2.16
2.06
1.97
5.00
2.90
0.46
0.37
0.85
0.92
0.85
0.89
1.28
1.07
5.79
f1 (ppm)

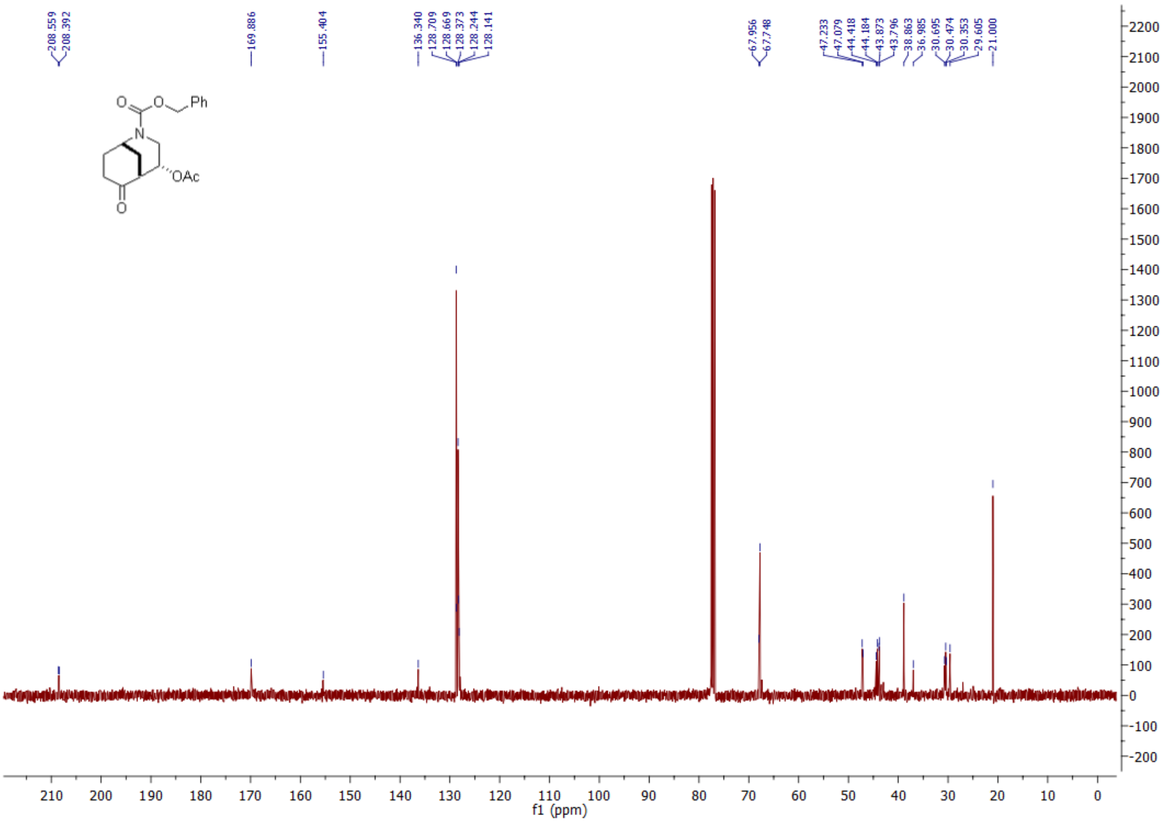
208.559
208.392
169.886
155.404
136.340
128.709
128.669
128.373
128.244
128.141
67.956
67.748
47.233
47.079
44.418
44.184
43.873
43.796
38.863
36.985
30.695
30.474
30.353
29.605
21.000
f1 (ppm)

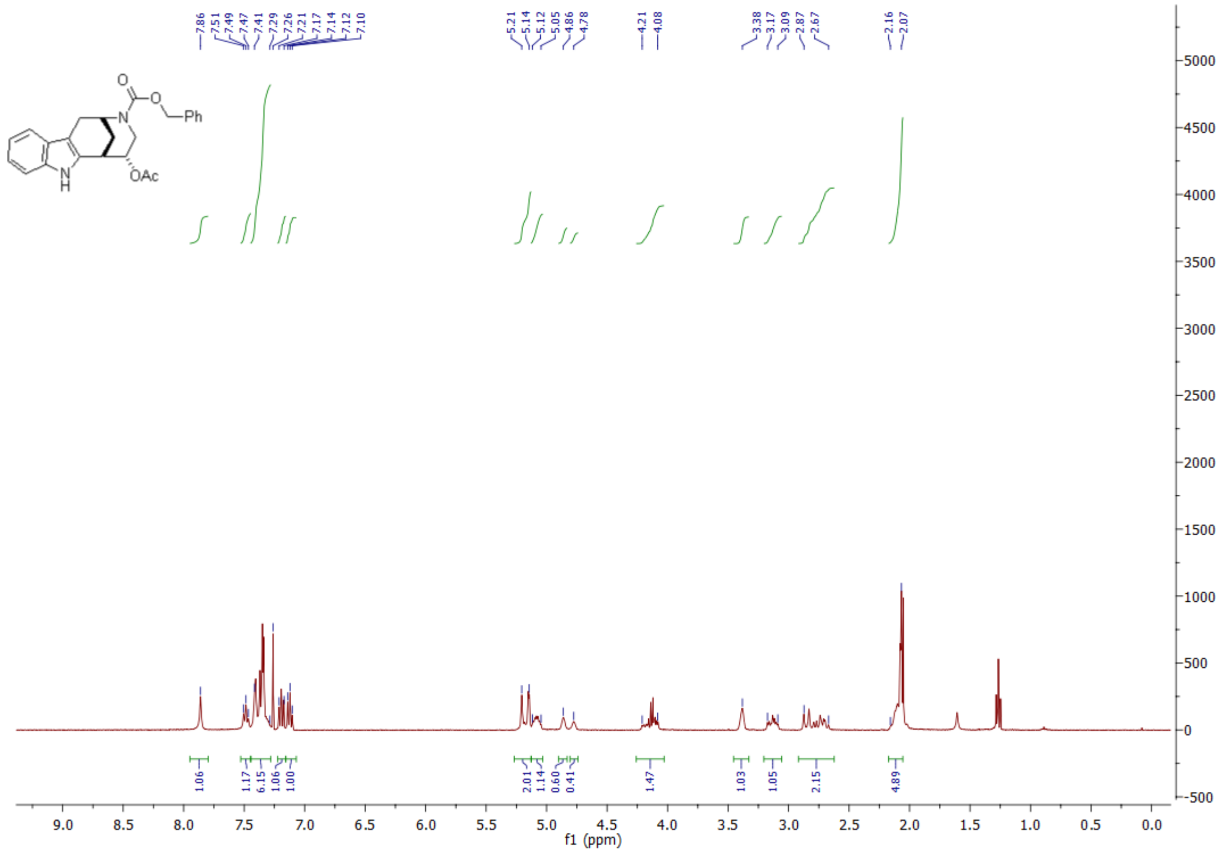

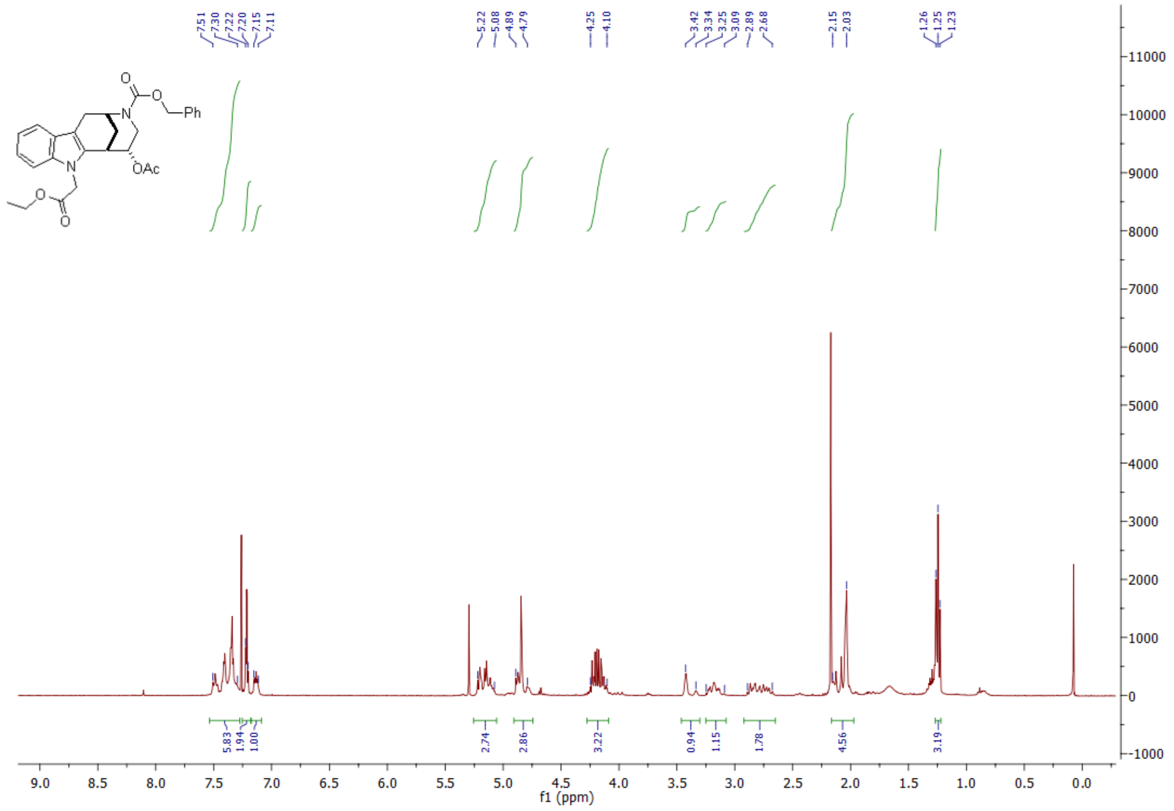
OAc
Ph
f1 (ppm)

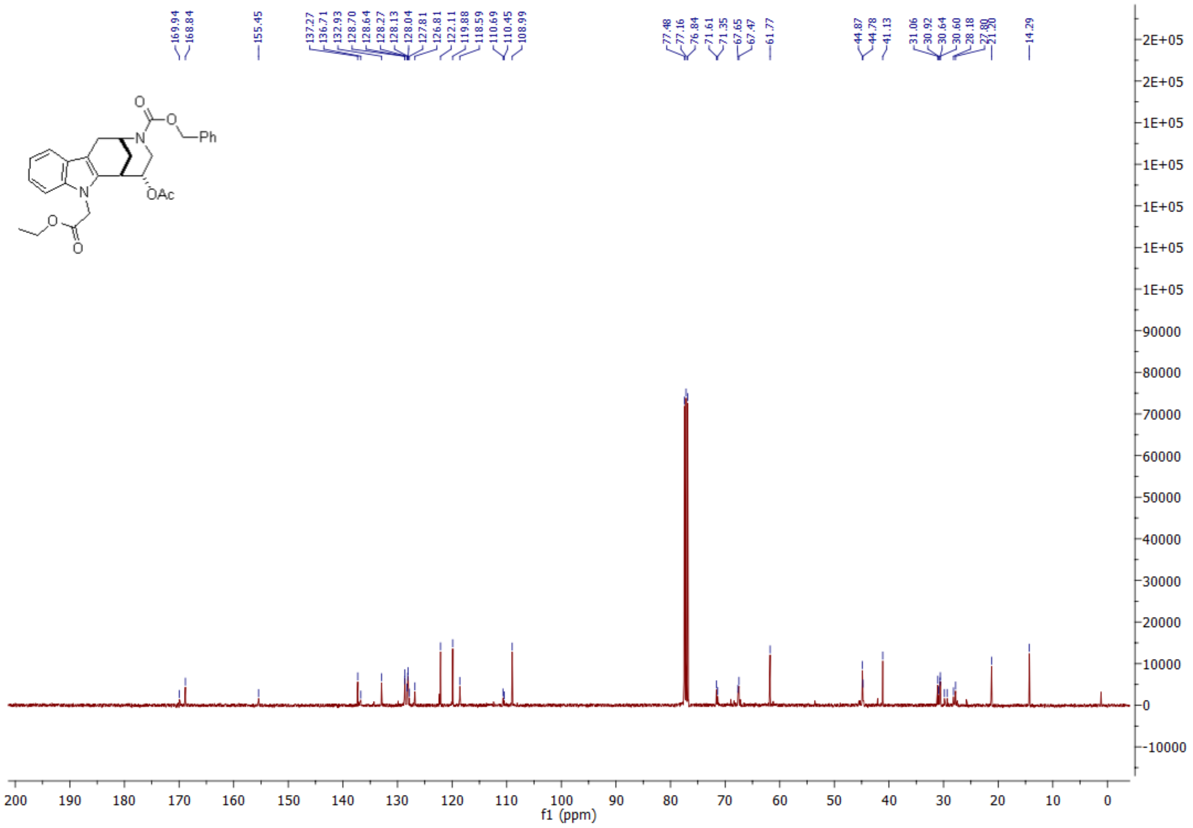
OAc
Ph
f1 (ppm)

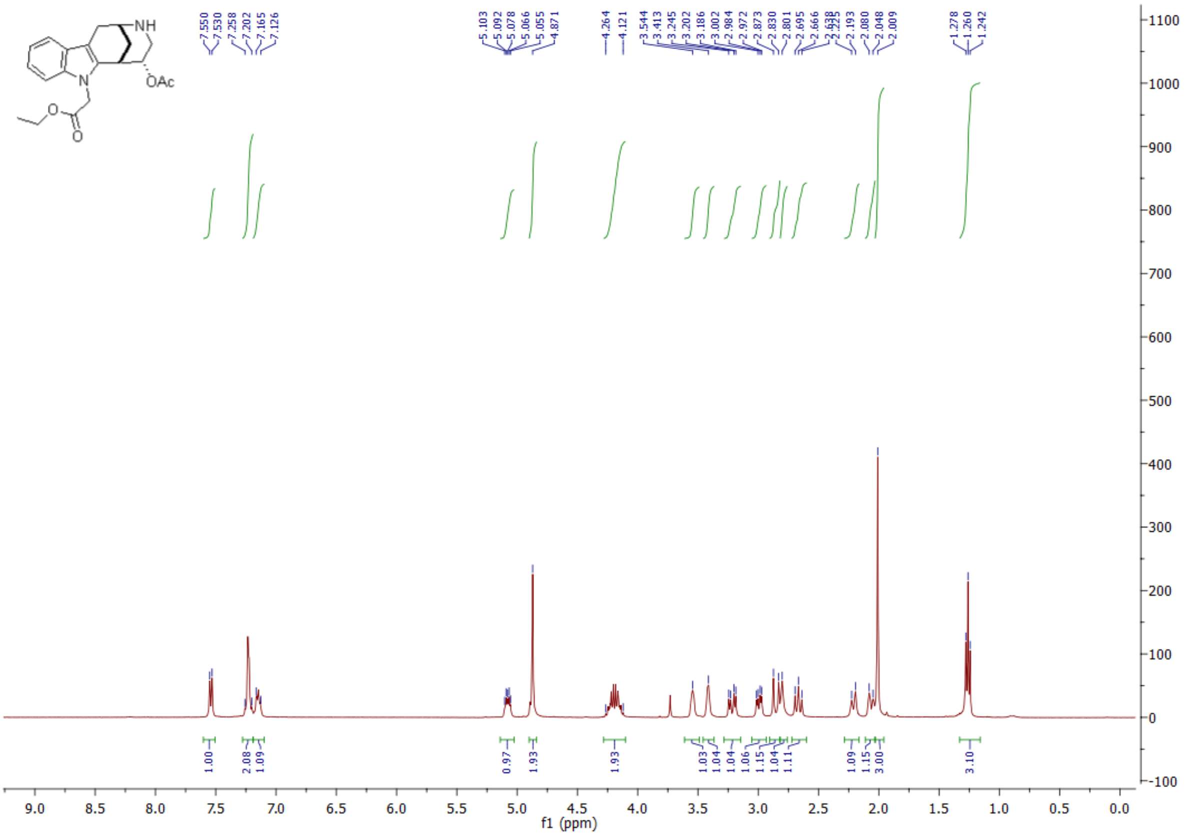
NH
OAc
N
O
O
7.550
7.530
7.258
7.202
7.165
7.126
5.103
5.092
5.078
5.066
5.055
4.871
4.264
4.121
1.278
1.260
1.242
1.00
2.08
1.09
0.97
1.93
1.93
3.10
f1 (ppm)

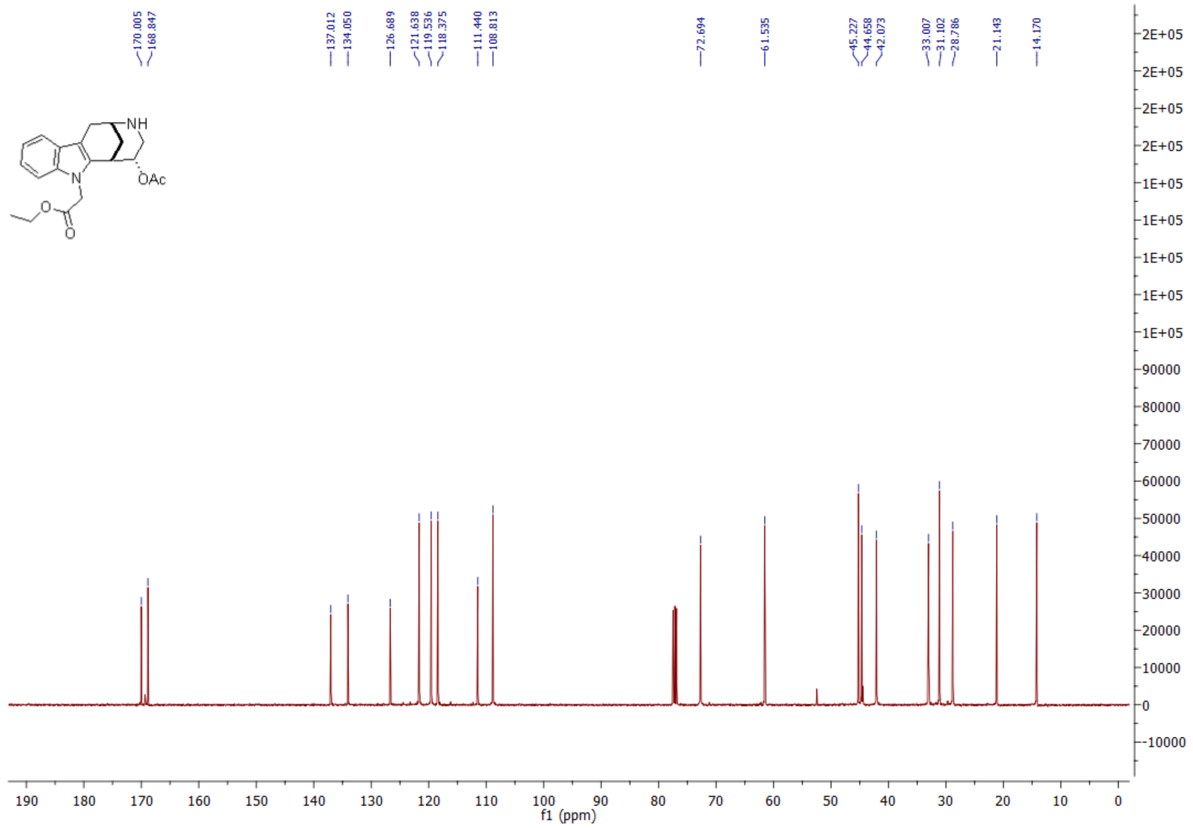
170.005
168.847
137.012
134.050
126.689
121.638
119.536
118.375
111.440
108.813
72.694
61.535
45.227
44.658
42.073
33.007
31.102
28.786
21.143
14.170
NH
OAc
N
O
O
f1 (ppm)

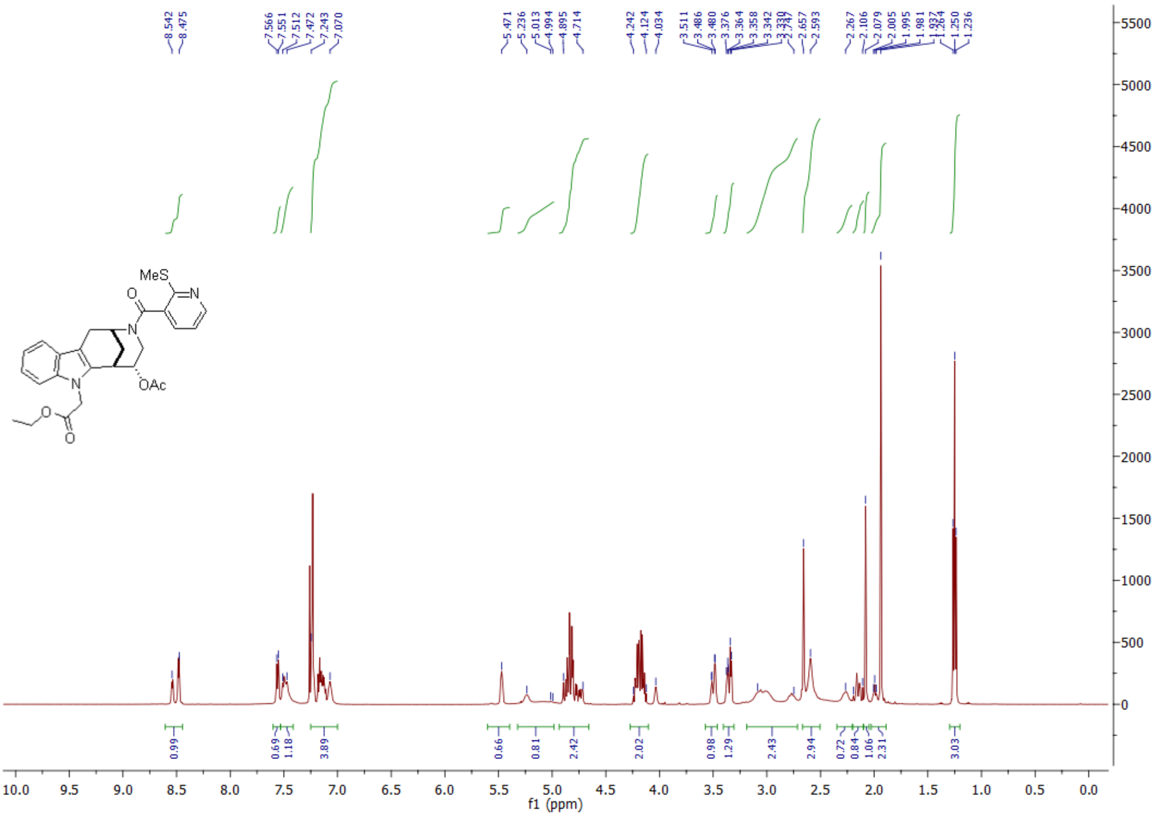
MeS
N
O
N
OAc
N
O
O
f1 (ppm)

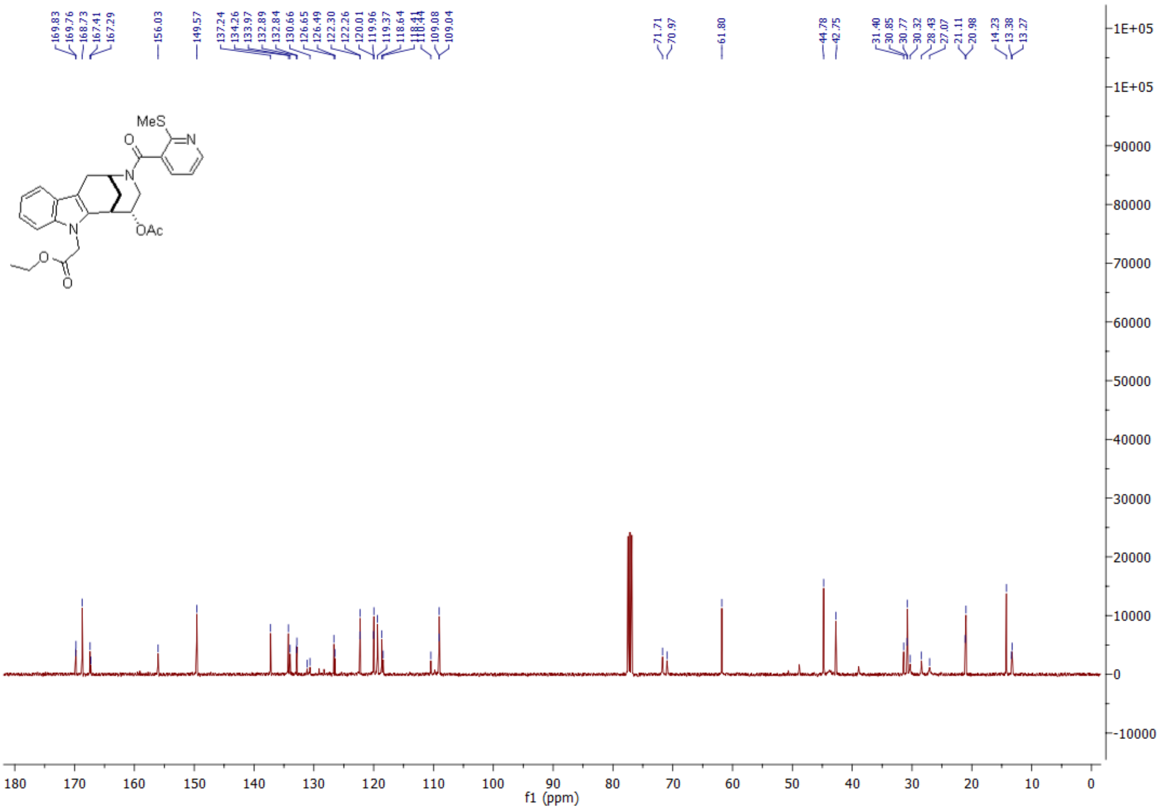
MeS
N
O
N
OAc
N
O
O
f1 (ppm)

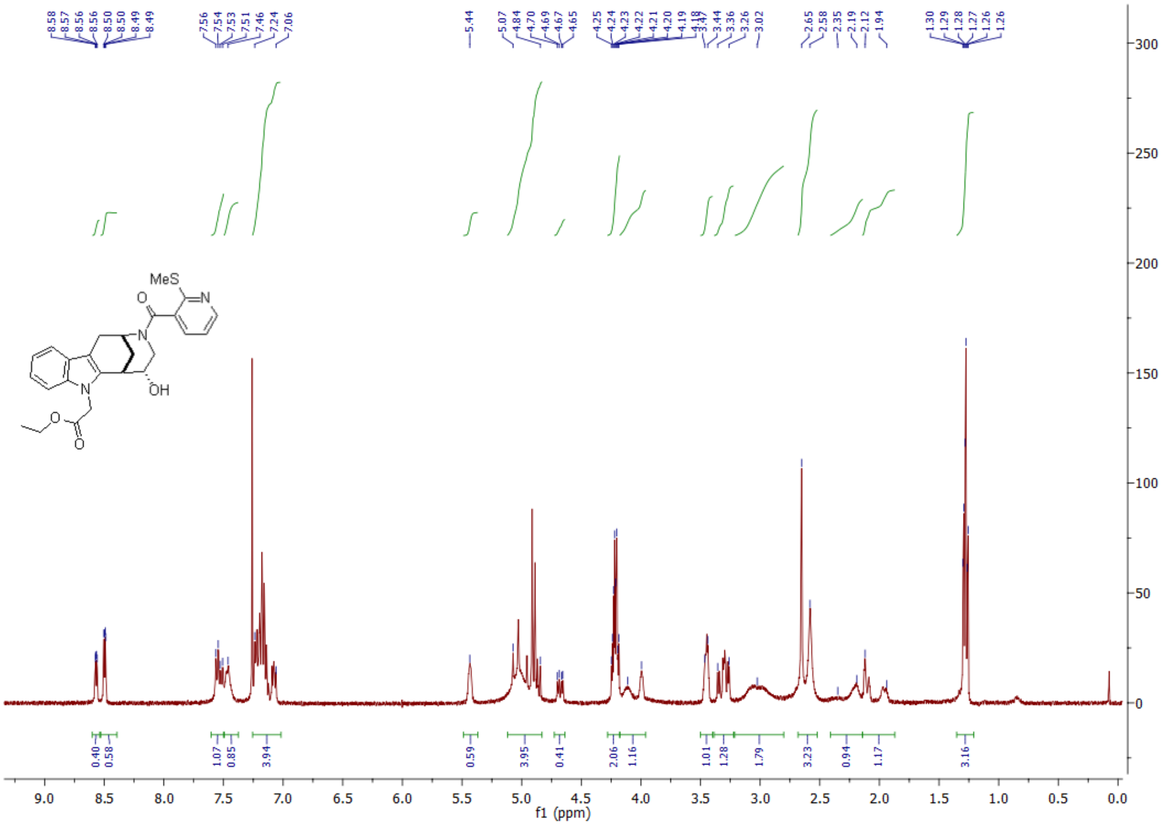
MeS
N
O
N
N
OH
O
O
f1 (ppm)

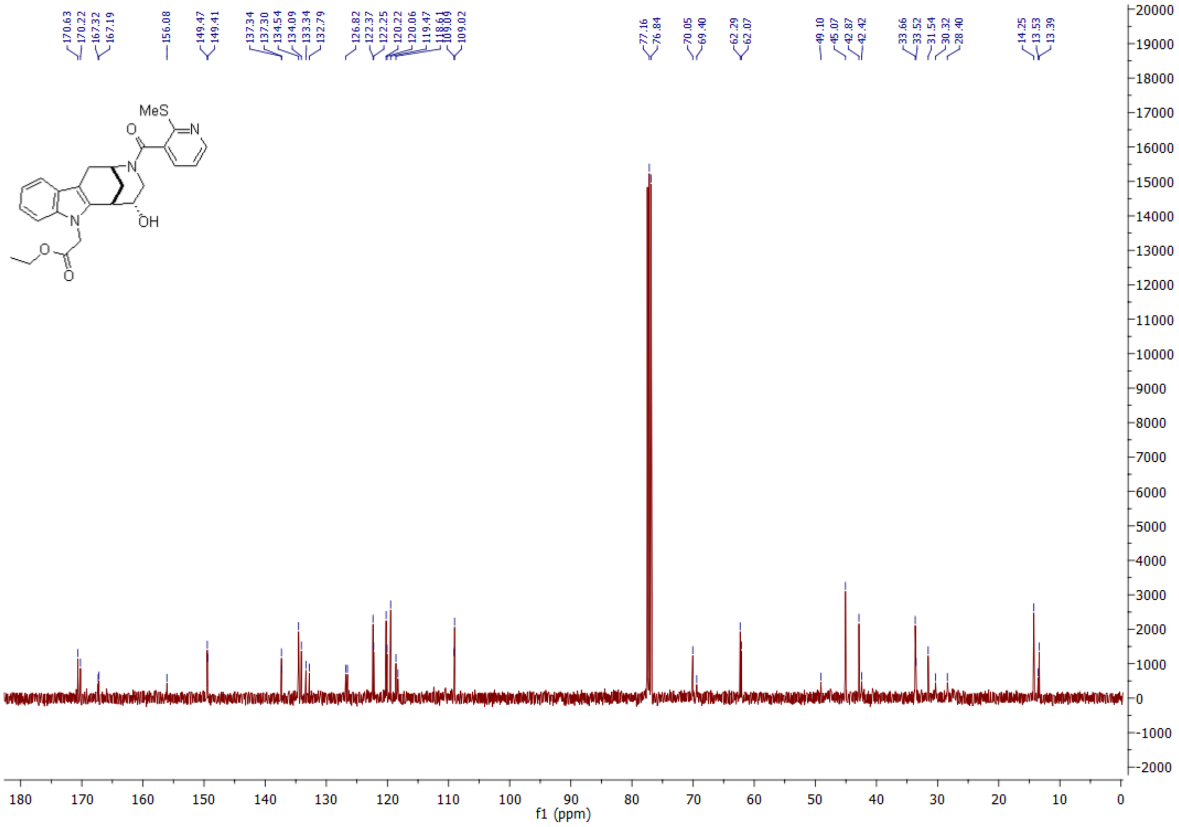
MeS
N
O
N
N
OH
O
O
f1 (ppm)

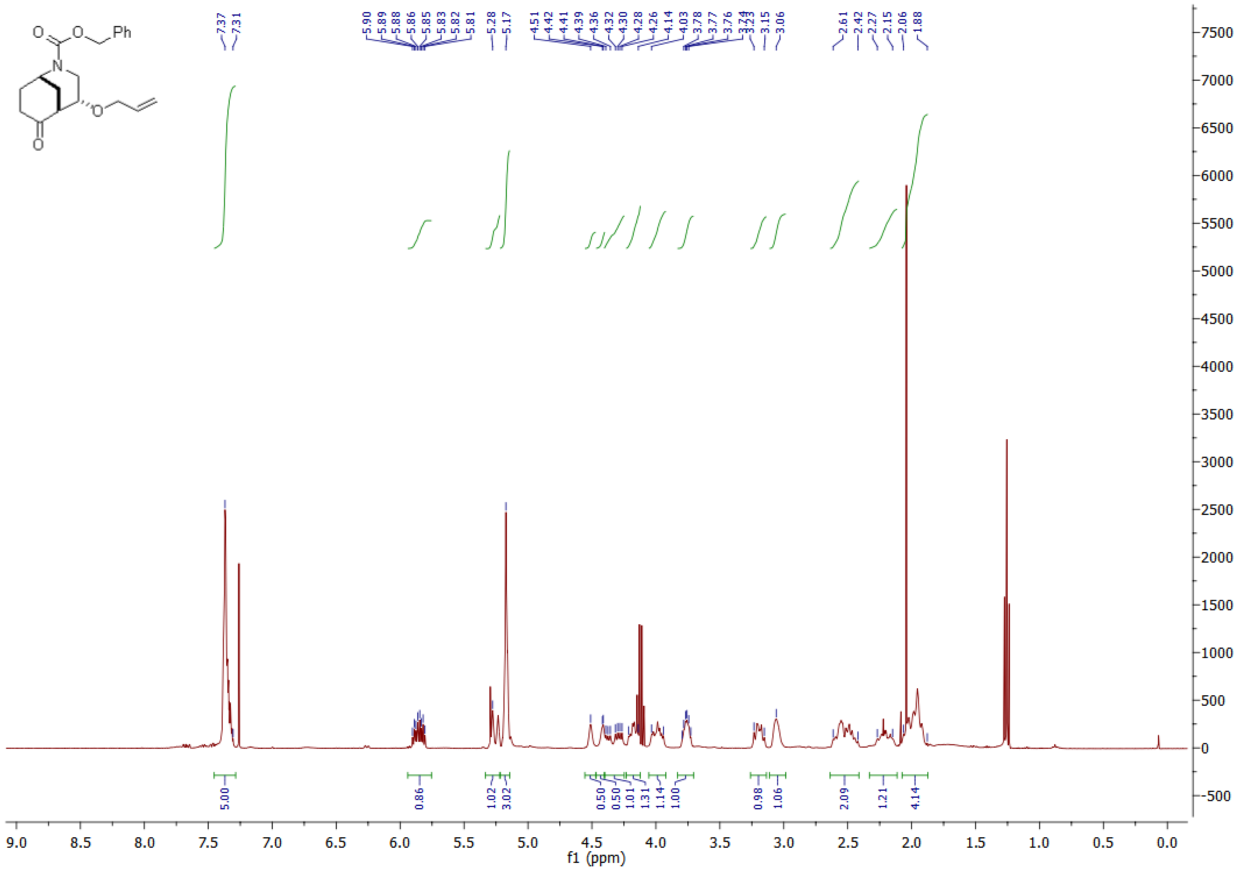
7.37
7.31
5.90
5.89
5.88
5.86
5.85
5.83
5.82
5.81
5.28
5.17
4.51
4.42
4.41
4.39
4.36
4.32
4.30
4.28
4.26
4.14
4.03
3.78
3.77
3.76
3.24
3.23
3.15
3.06
2.61
2.42
2.27
2.15
2.06
1.88
5.00
0.86
1.02
3.02
0.50
0.50
1.01
1.31
1.14
1.00
0.98
1.06
2.09
1.21
4.14
f1 (ppm)
Ph
O
N

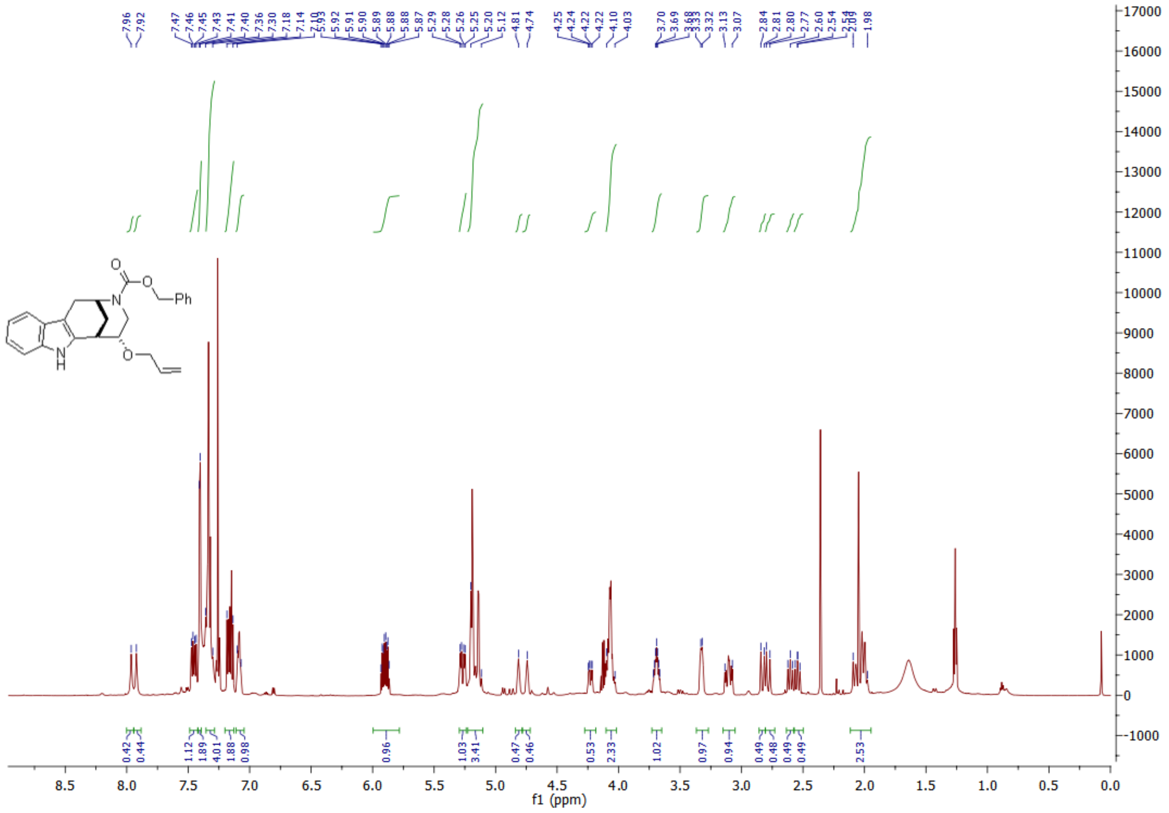

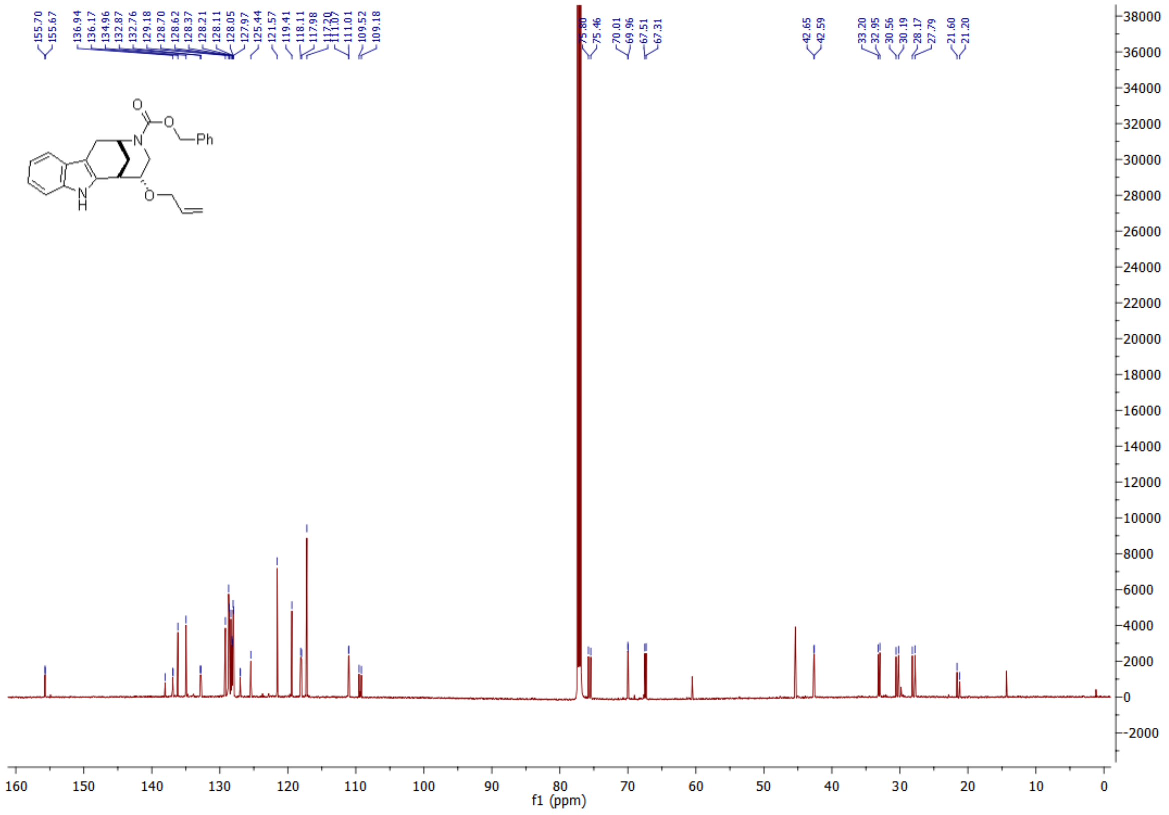

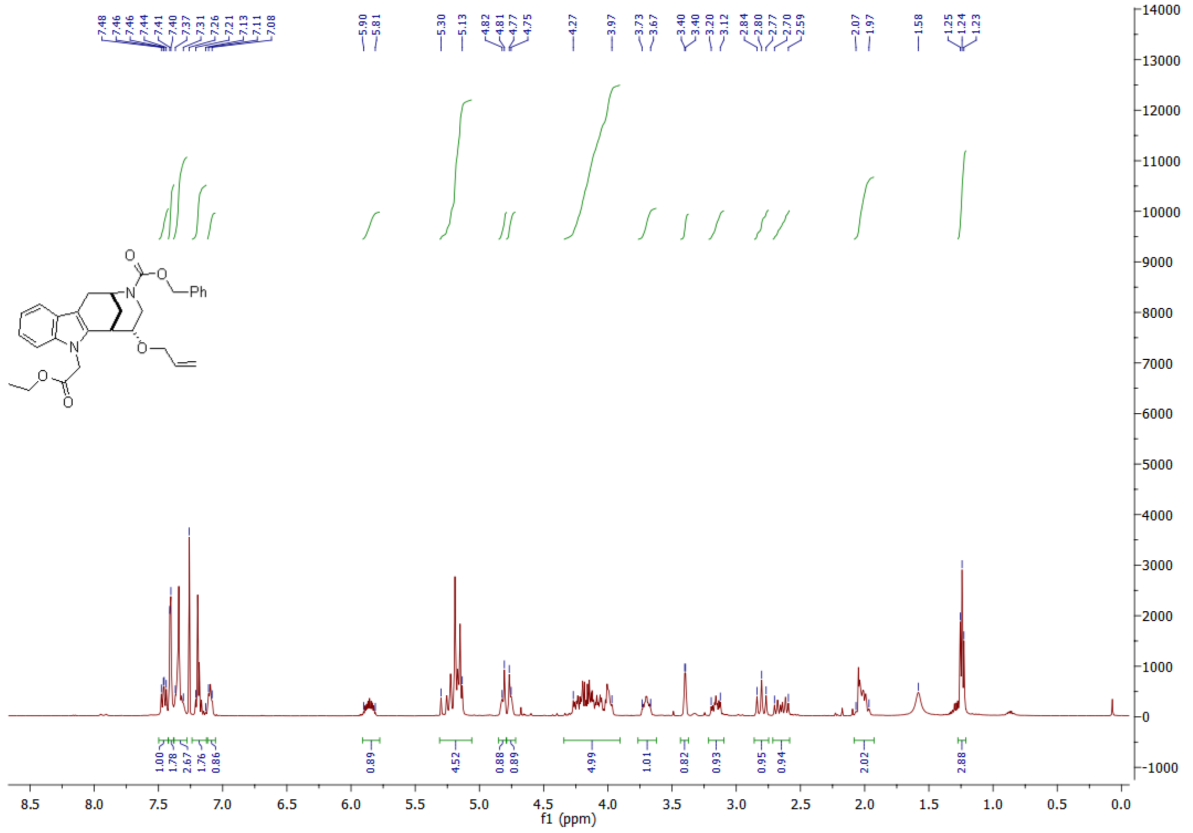
7.48
7.46
7.46
7.44
7.41
7.40
7.37
7.31
7.26
7.21
7.13
7.11
7.08
5.90
5.81
5.30
5.13
4.82
4.81
4.77
4.75
4.27
3.97
3.73
3.67
3.40
3.40
3.20
3.12
2.84
2.80
2.77
2.70
2.59
2.07
1.97
1.58
1.25
1.24
1.23
1.00
1.78
2.67
1.76
0.86
0.89
4.52
0.88
0.89
4.99
1.01
0.82
0.93
0.95
0.94
2.02
2.88
f1 (ppm)
Ph
O
N

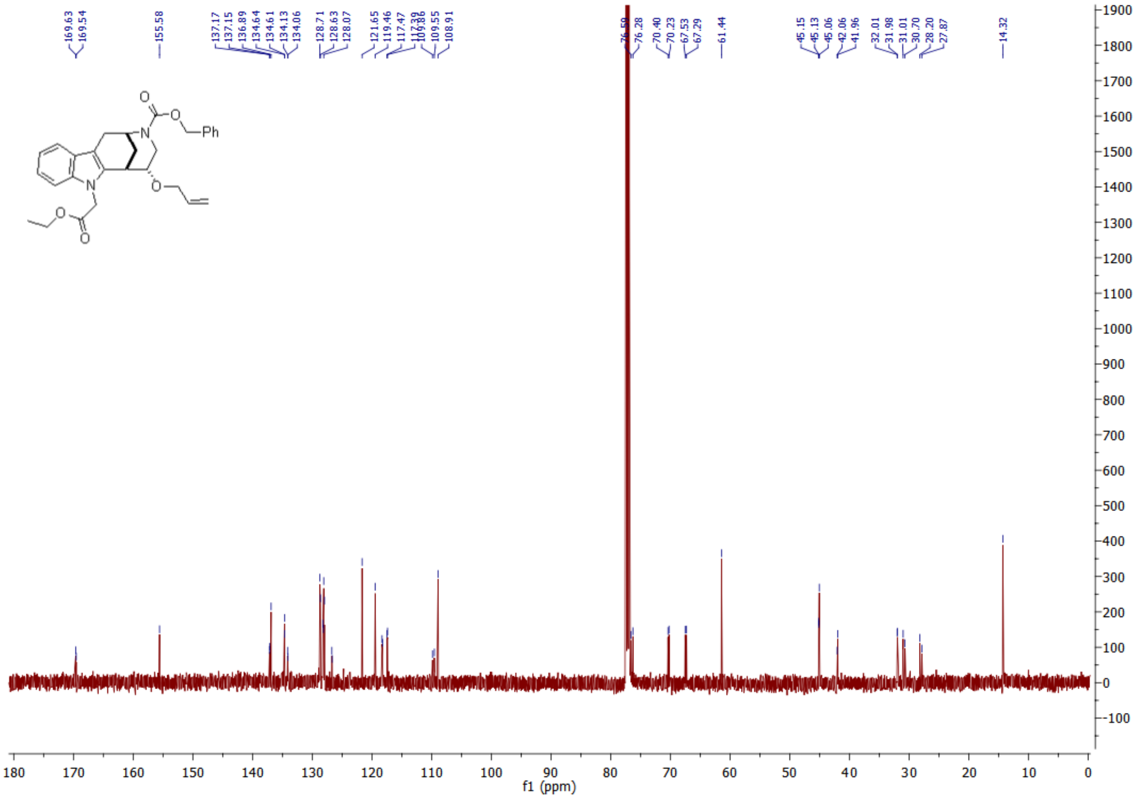
169.63
169.54
155.58
137.17
137.15
136.89
134.64
134.61
134.13
134.06
128.71
128.63
128.07
121.65
119.46
117.47
109.55
108.91
76.28
70.40
70.23
67.53
67.29
61.44
45.15
45.13
45.06
42.06
41.96
32.01
31.98
31.01
30.70
28.20
27.87
14.32
f1 (ppm)
Ph
O
N

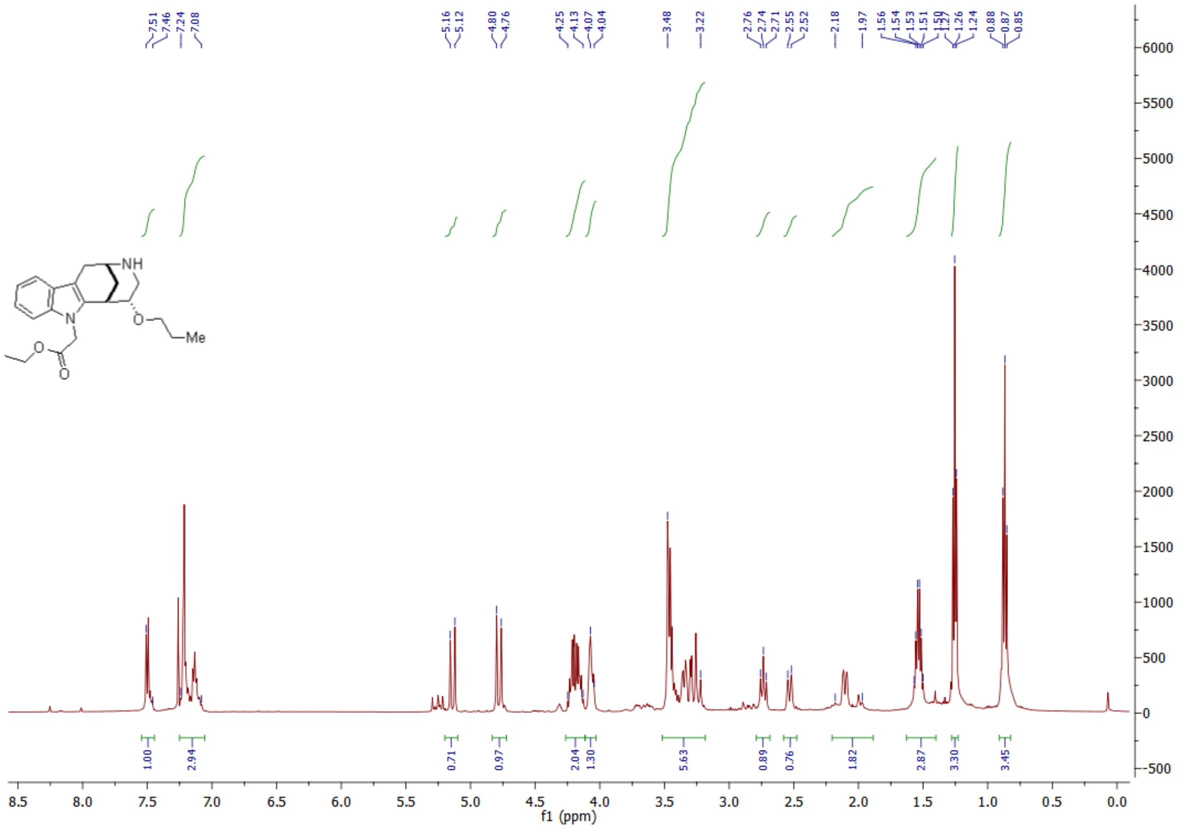
NH
N
O
Me
O
O
f1 (ppm)

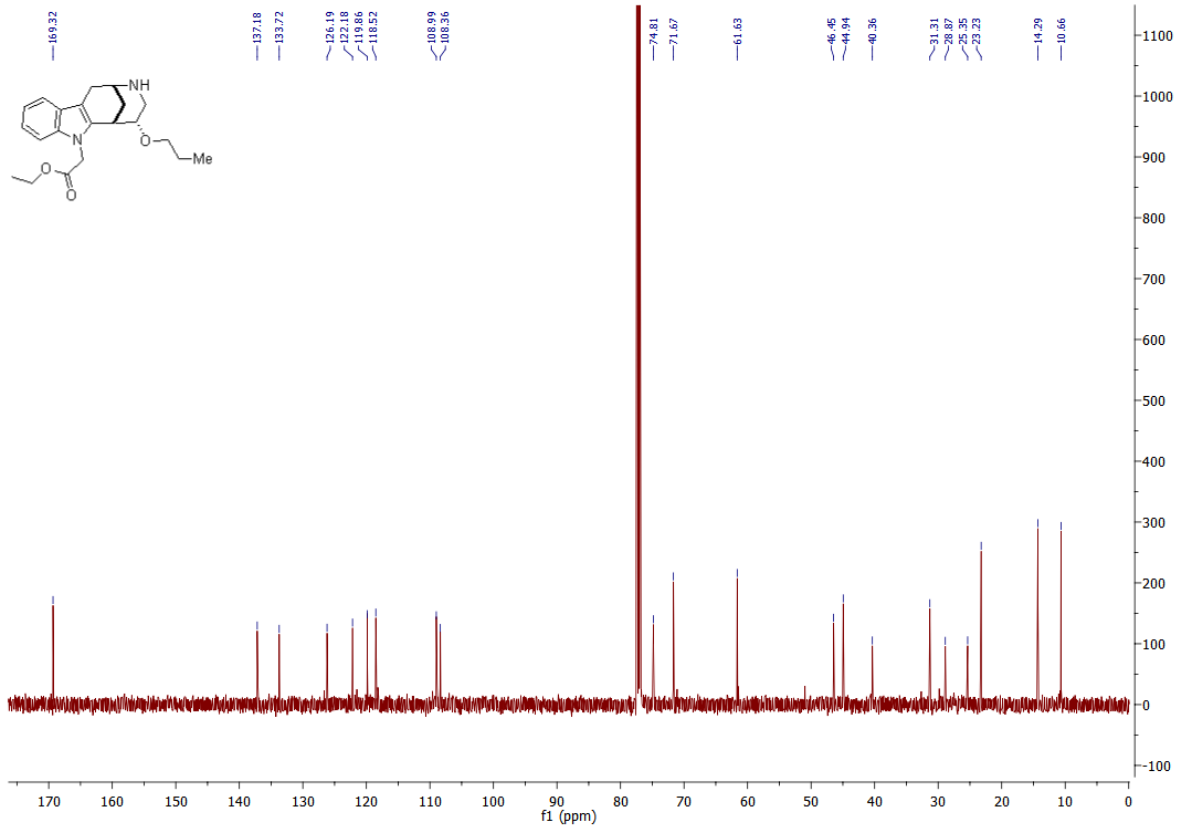
NH
N
O
Me
O
O
f1 (ppm)

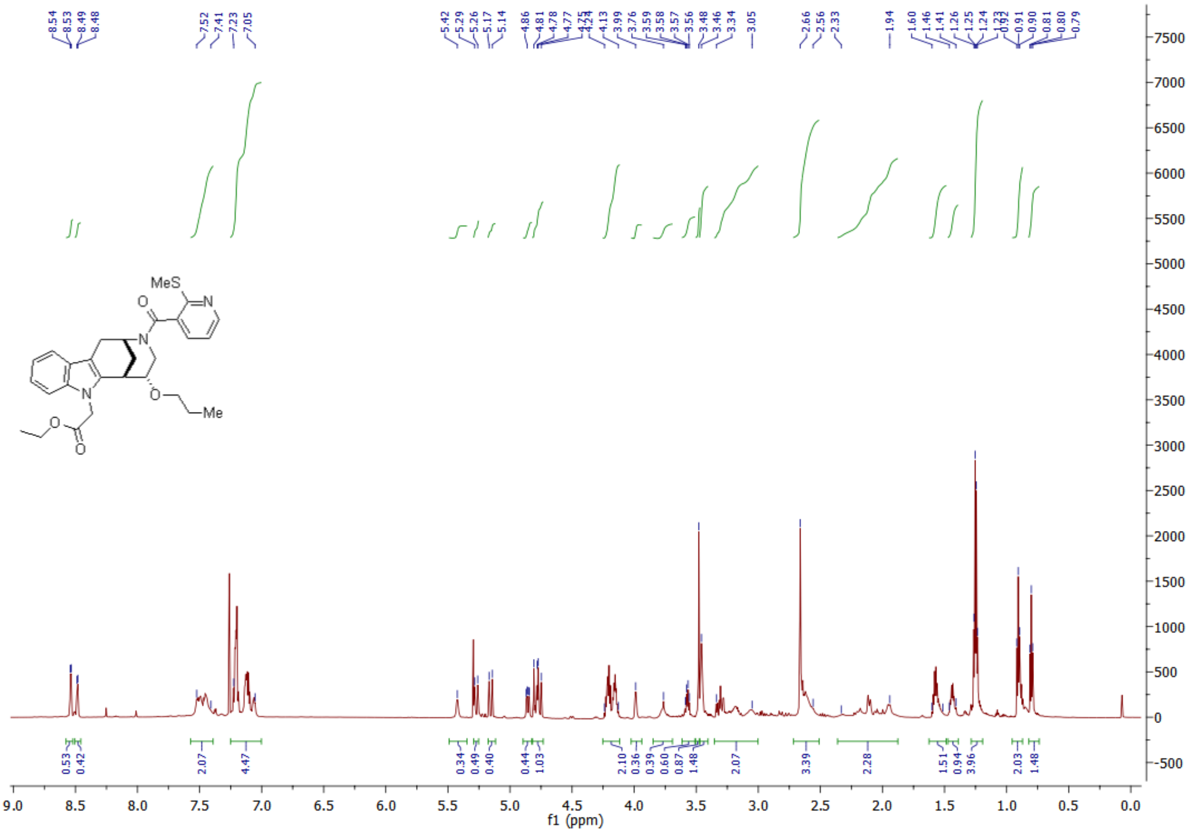
8.54 8.53 8.49 8.48
7.52 7.41 7.23 7.05
5.42 5.29 5.26 5.17 5.14
4.86 4.81 4.78 4.77 4.25 4.24 4.13 3.99 3.76 3.59 3.58 3.57 3.56 3.48 3.46 3.34 3.05
2.66 2.56 2.33
1.94 1.60 1.46 1.41 1.26 1.25 1.24 1.23 0.92 0.91 0.90 0.81 0.80 0.79
MeS
O
N
N
N
O
Me
O
O
0.53 0.42 2.07 4.47 0.34 0.49 0.40 0.44 1.03 2.10 0.36 0.39 0.60 0.87 1.48 2.07 3.39 2.28 1.51 0.94 3.96 2.03 1.48
f1 (ppm)

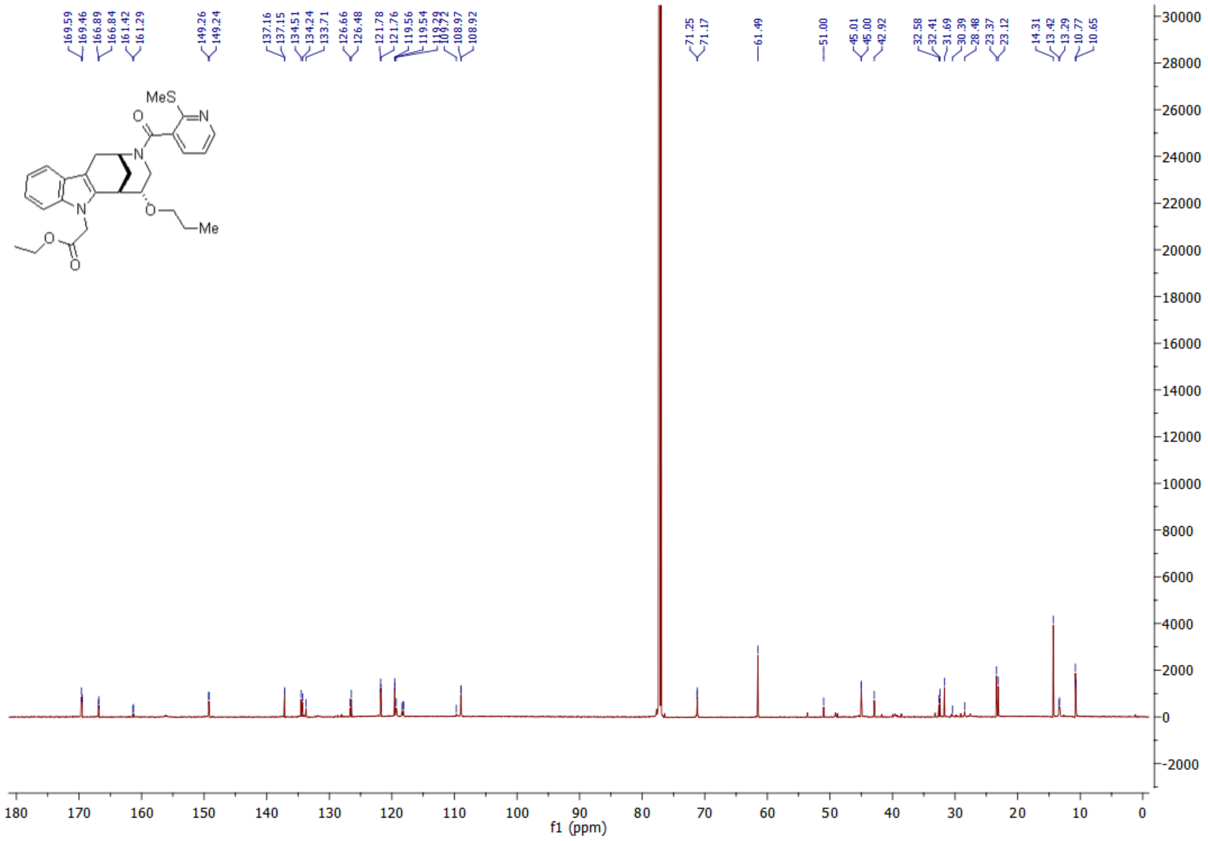
169.59 169.46 166.89 166.84 161.42 161.29
149.26 149.24
137.16 137.15 134.51 134.24 133.71 126.66 126.48 121.78 121.76 119.56 119.54 108.97 108.92
71.25 71.17
61.49
51.00
45.01 45.00 42.92
32.58 32.41 31.69 30.39 28.48 23.37 23.12
14.31 13.42 13.29 10.77 10.65
MeS
O
N
N
N
O
Me
O
O
f1 (ppm)

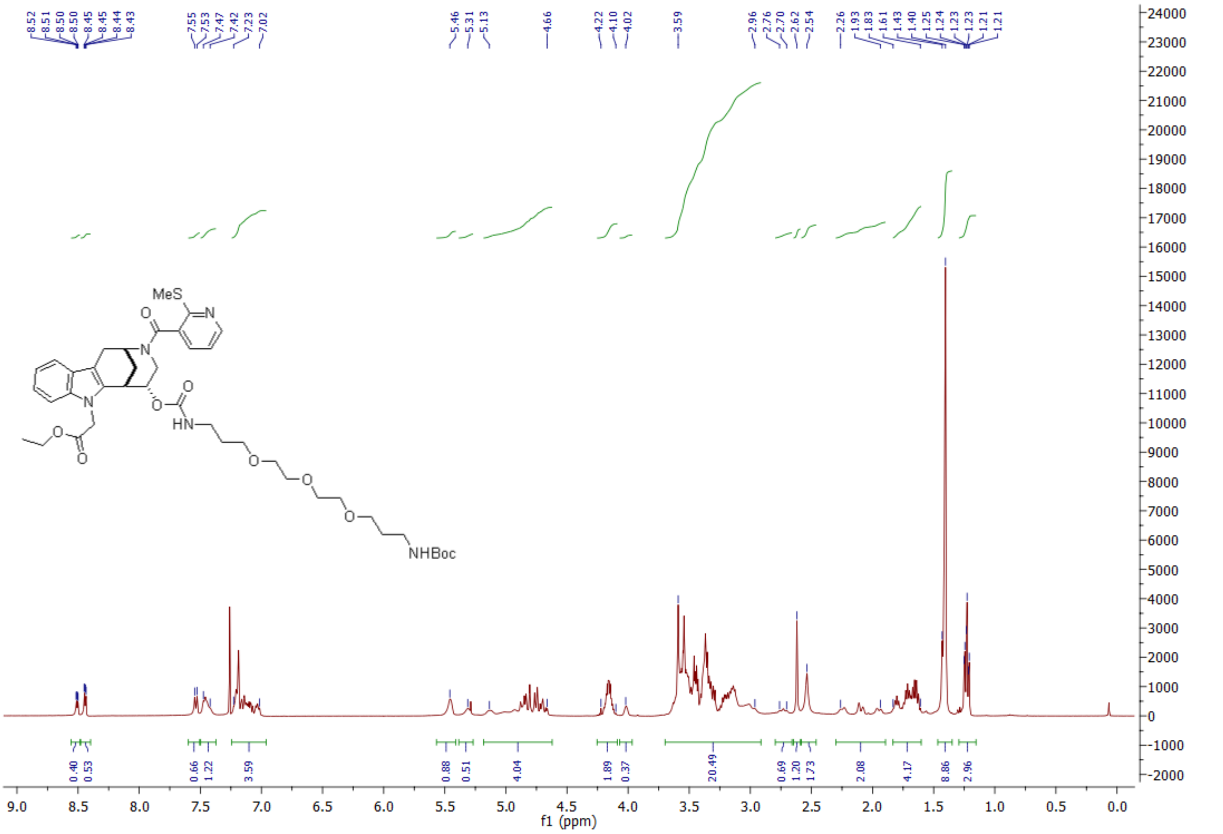

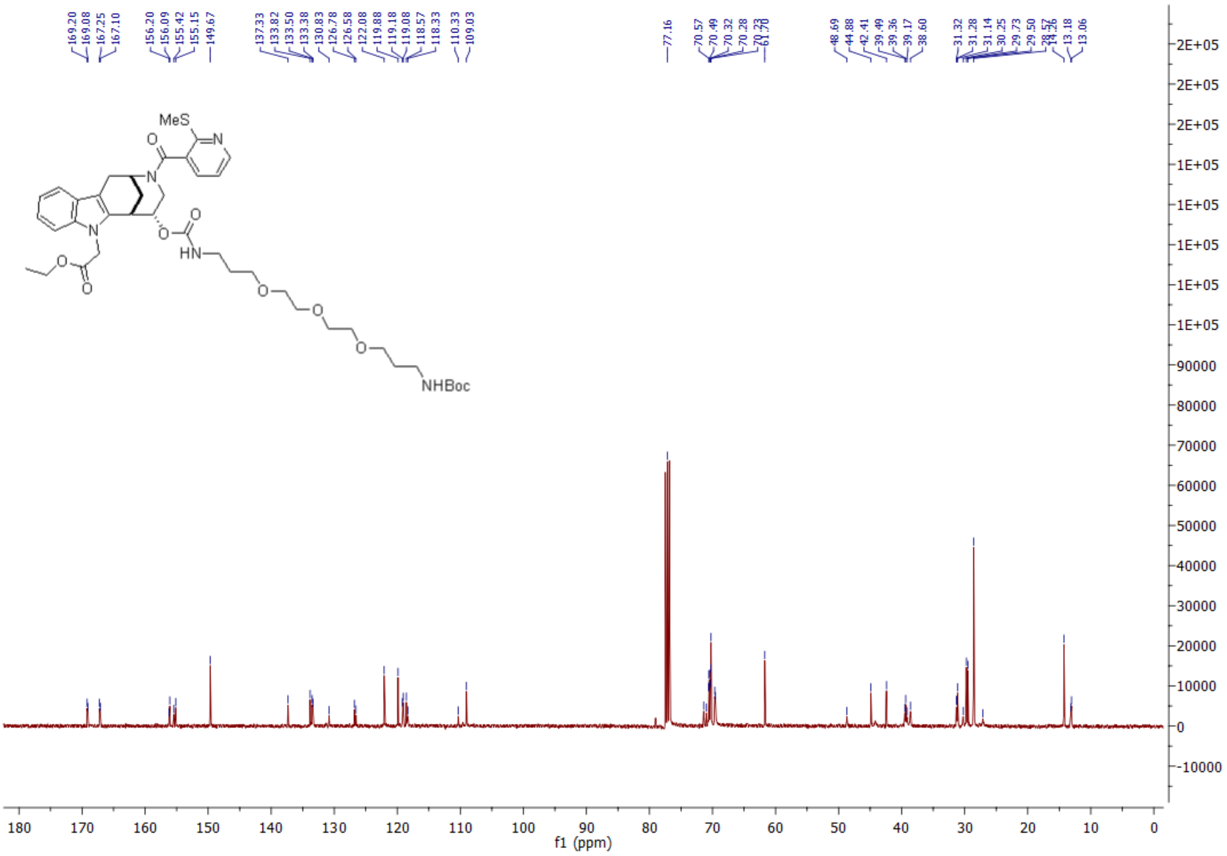

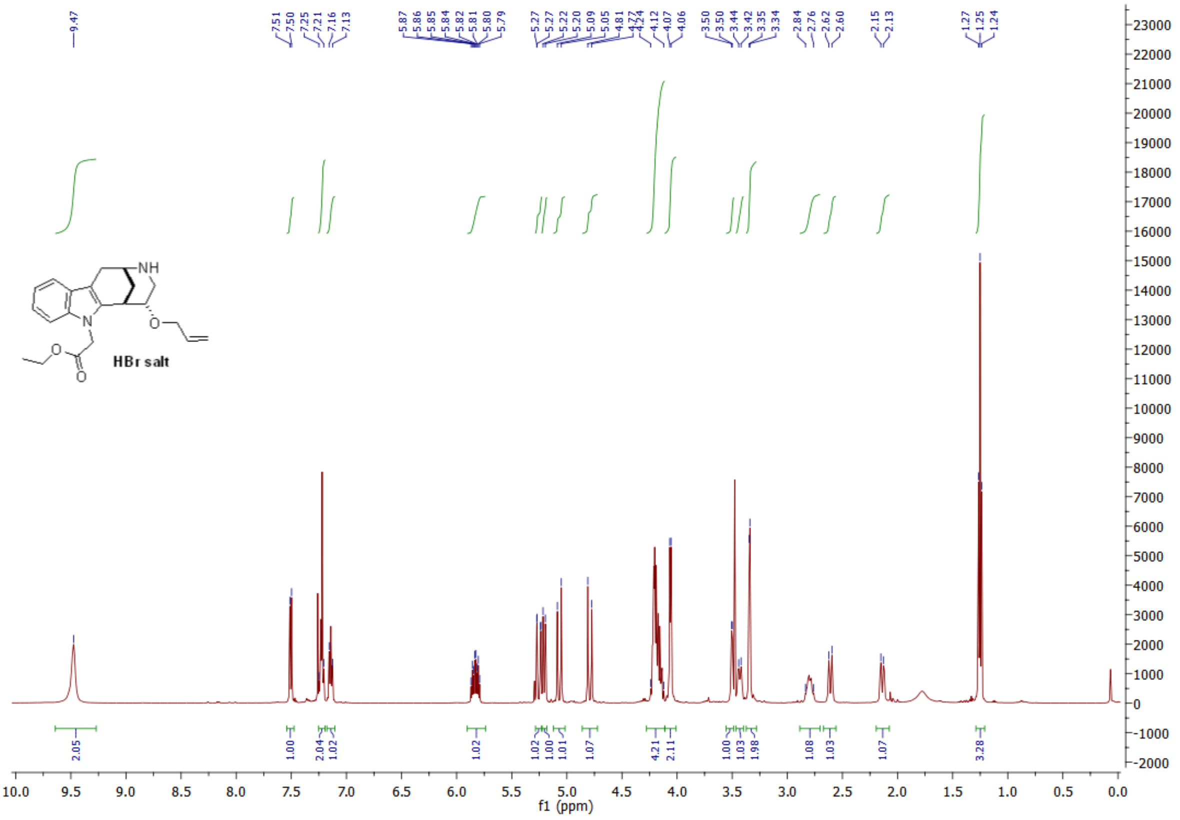

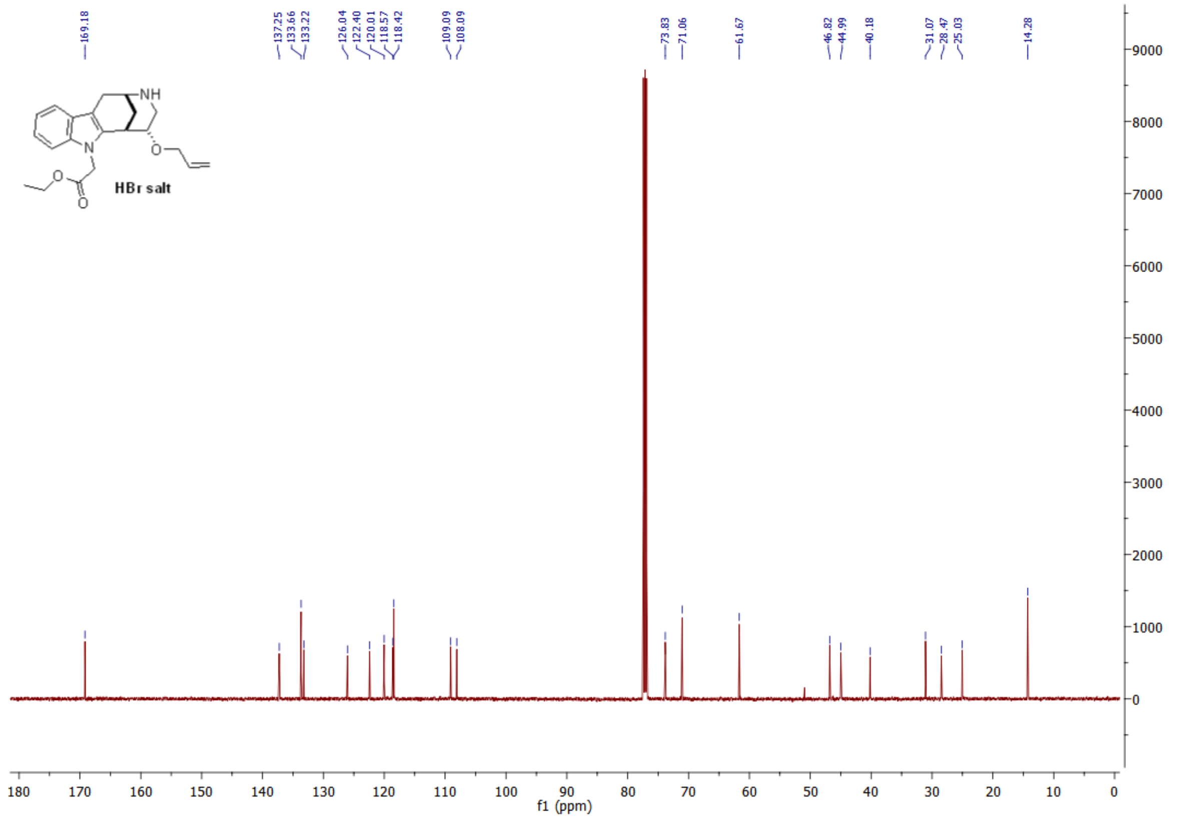

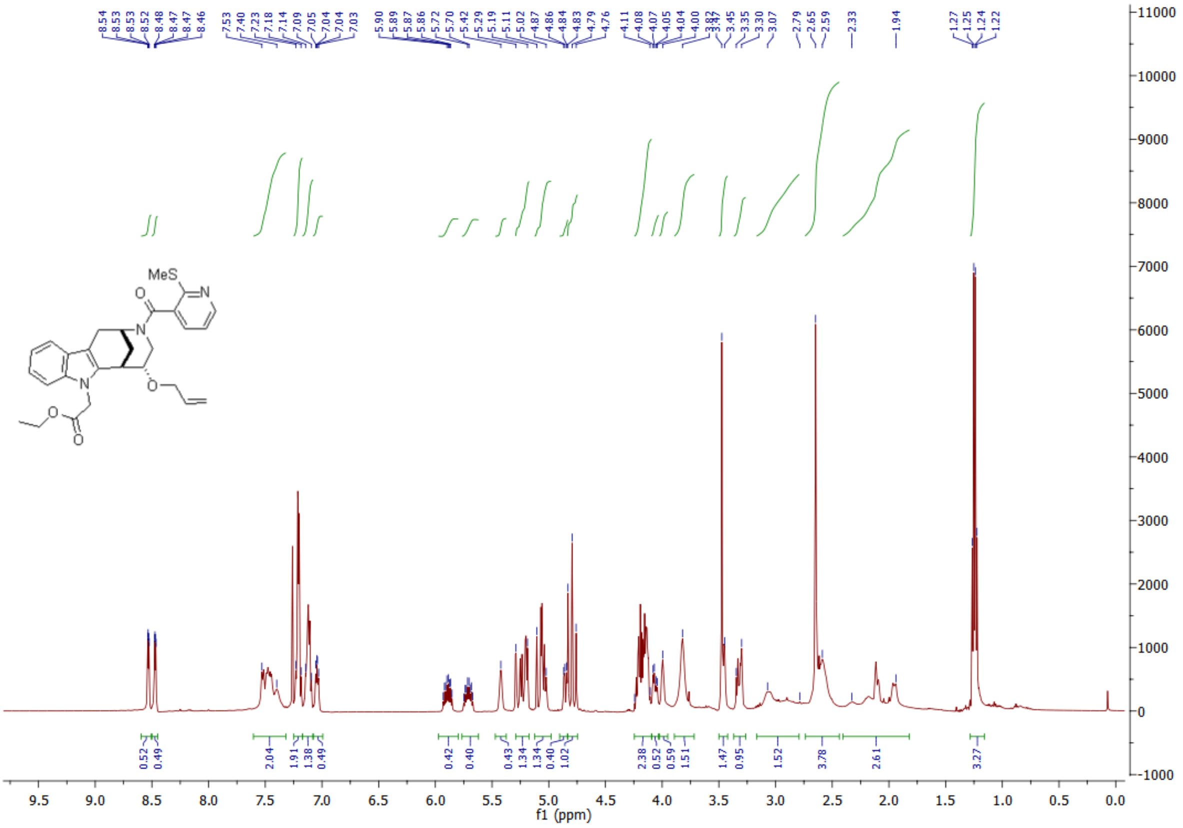
MeS
N
O
N
N
O
O
O
f1 (ppm)

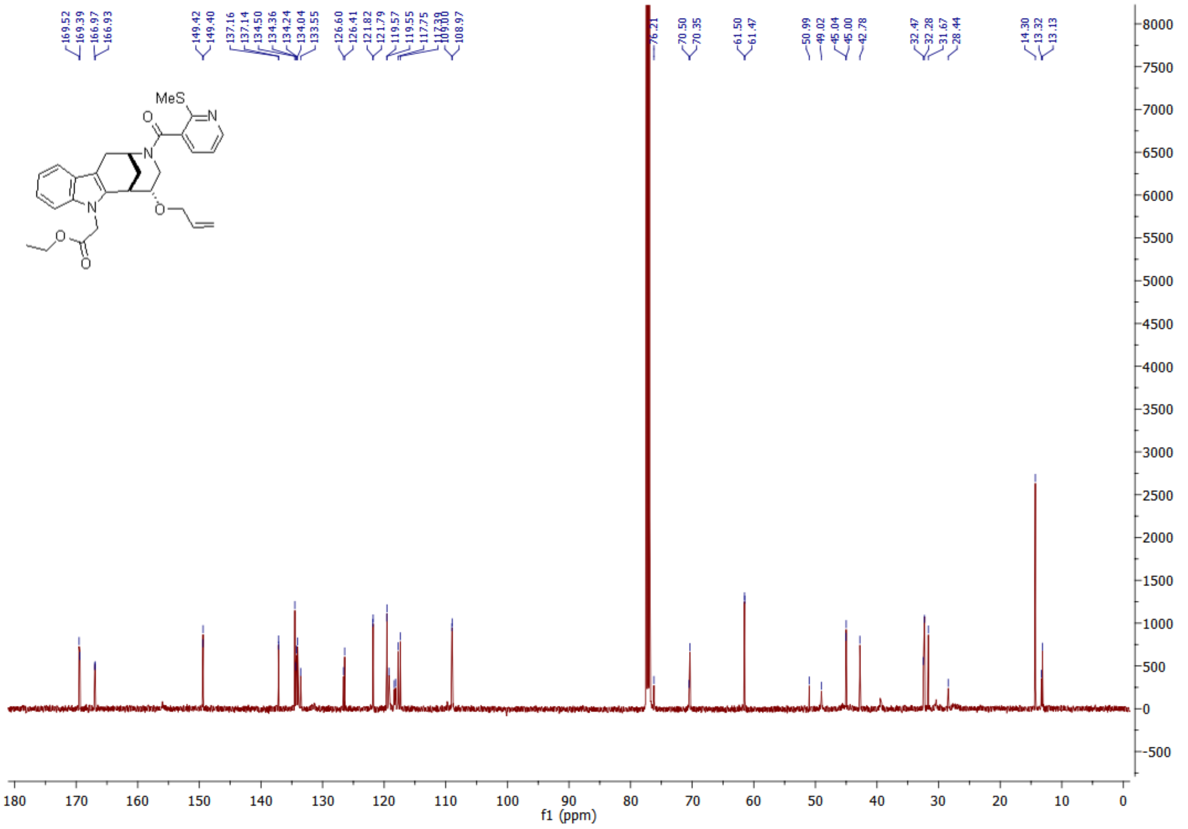
MeS
N
O
N
N
O
O
O
f1 (ppm)

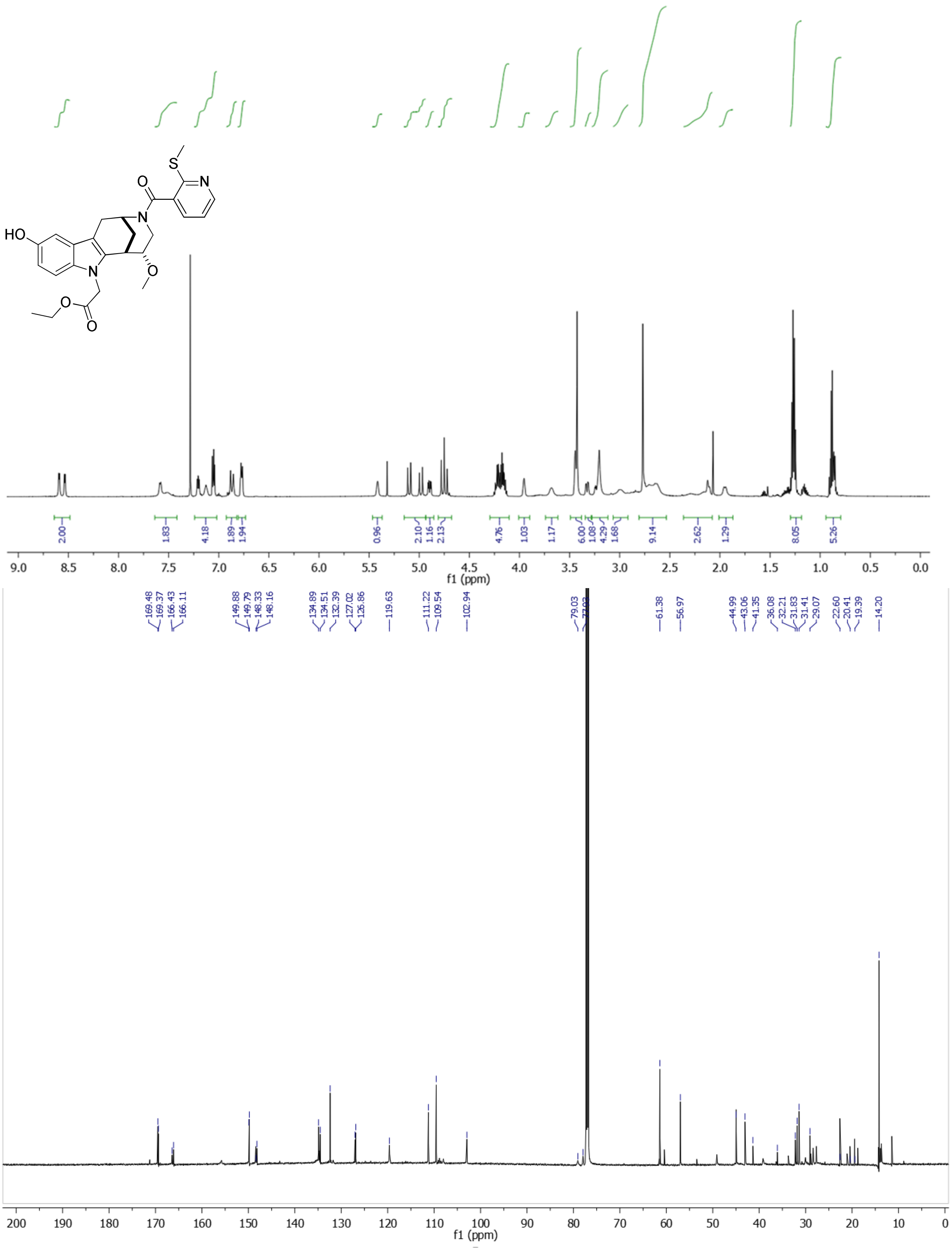

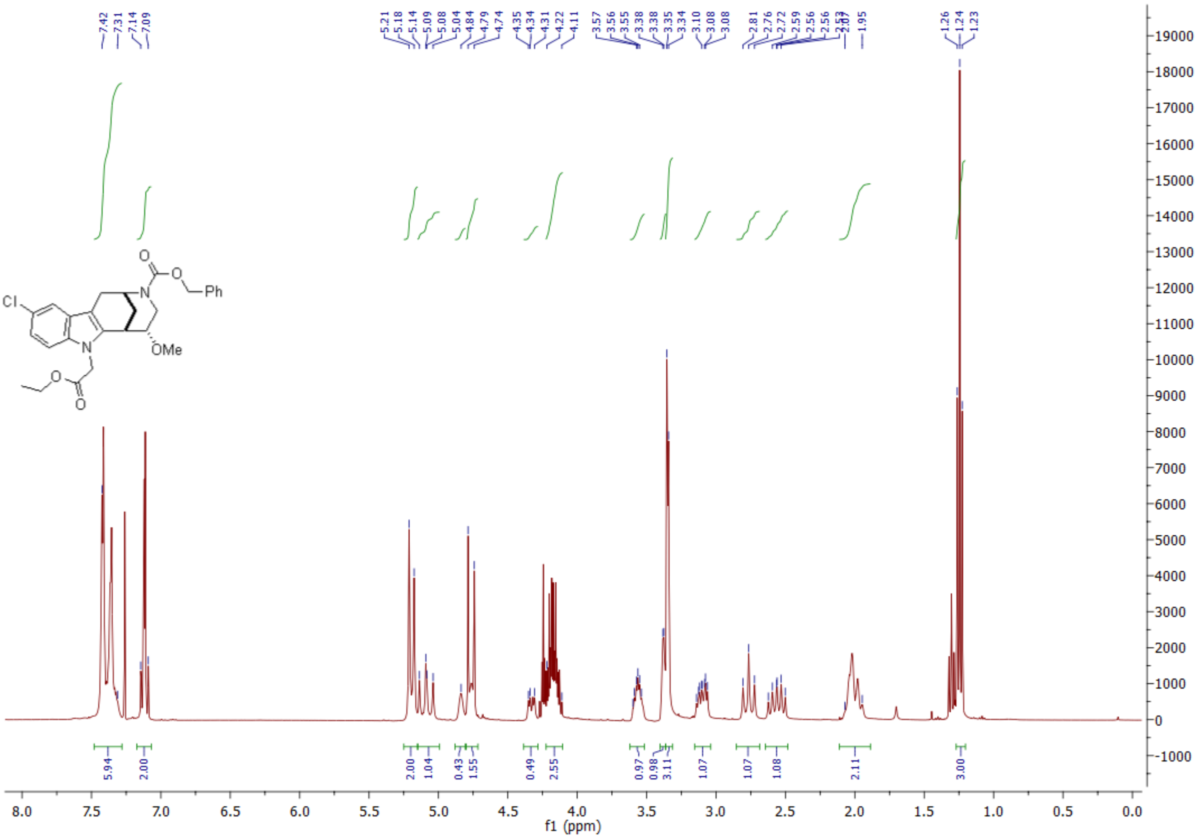
Cl
N
O
O
Ph
OMe
f1 (ppm)

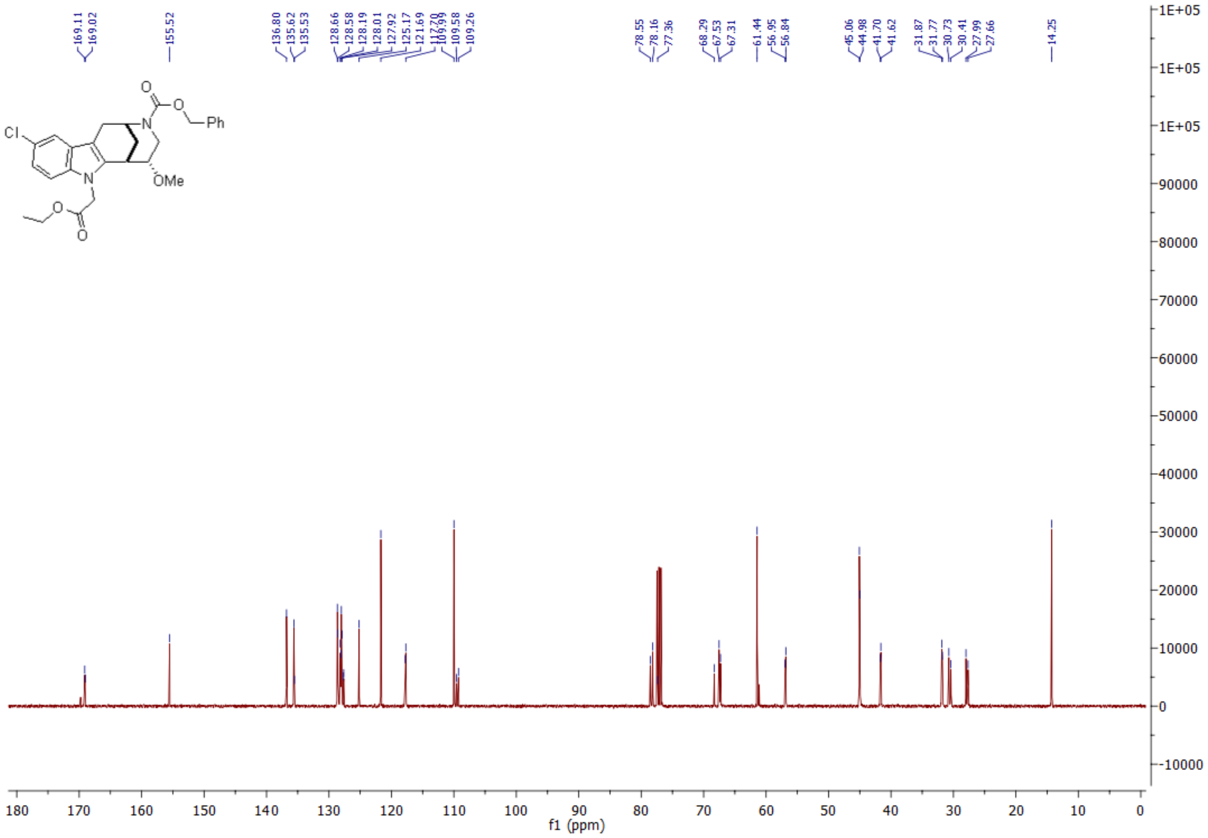
Cl
N
O
O
Ph
OMe
f1 (ppm)

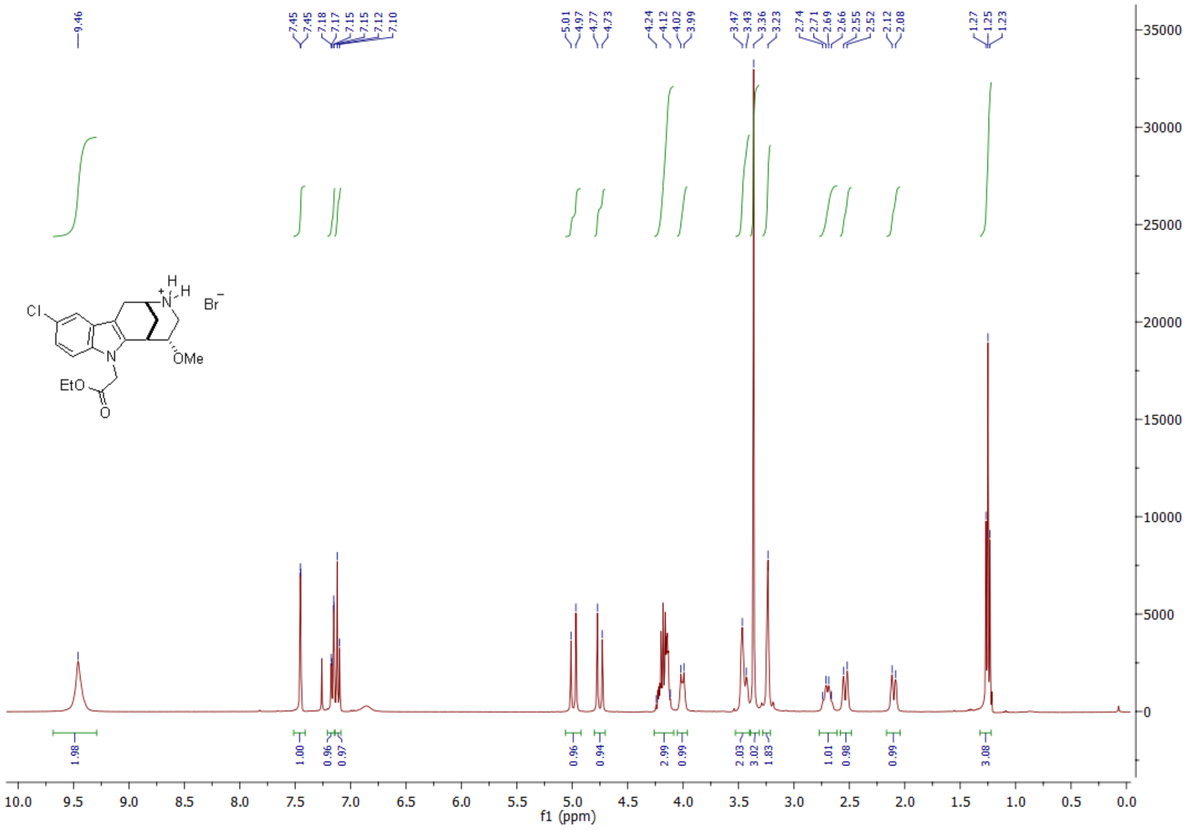
9.46
7.45
7.45
7.18
7.17
7.15
7.15
7.12
7.10
5.01
4.97
4.77
4.73
4.24
4.12
4.02
3.99
3.47
3.43
3.36
3.23
2.74
2.71
2.69
2.66
2.55
2.52
2.12
2.08
1.27
1.25
1.23
Cl
H
H
N
Br
N
OMe
EtO
O
f1 (ppm)

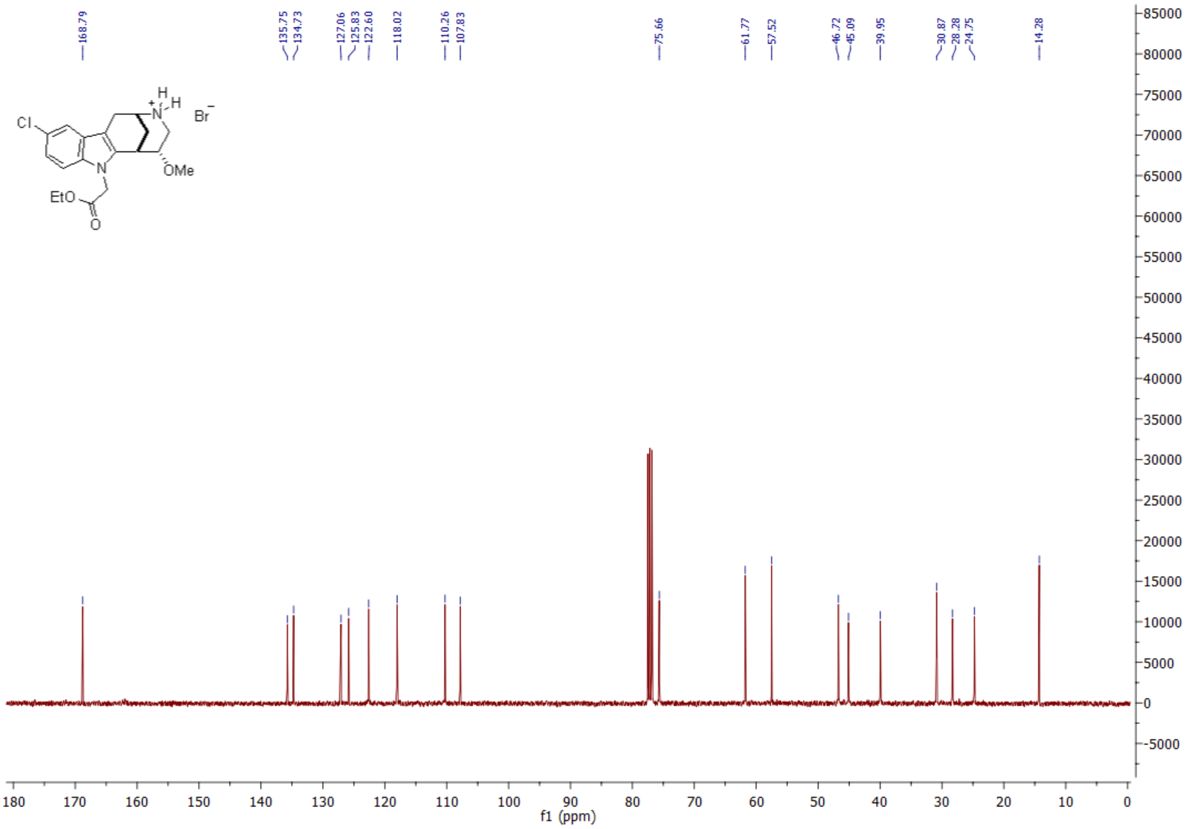
168.79
135.75
134.73
127.06
125.83
122.60
118.02
110.26
107.83
75.66
61.77
57.52
46.72
45.09
39.95
30.87
28.28
24.75
14.28
Cl
H
H
N
Br
N
OMe
EtO
O
f1 (ppm)

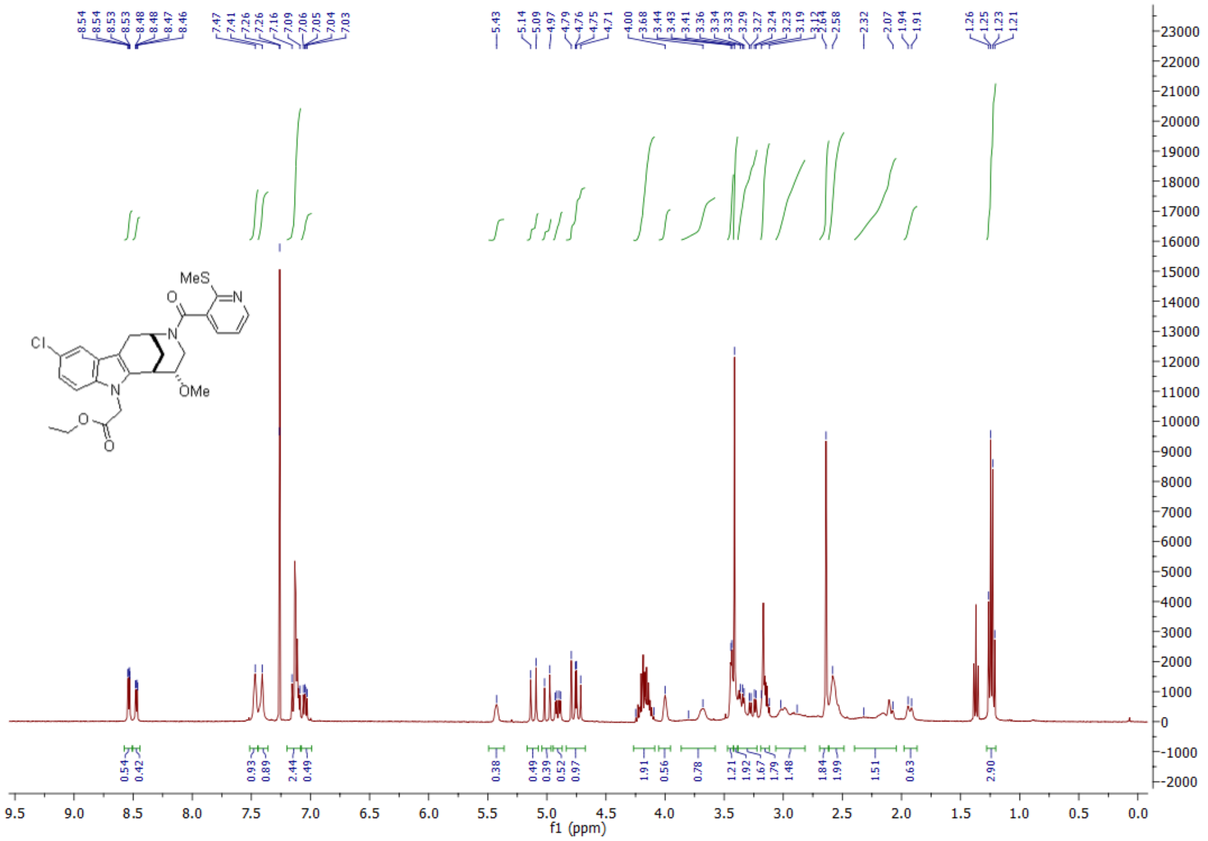
MeS
N
O
Cl
N
OMe
O
O
f1 (ppm)

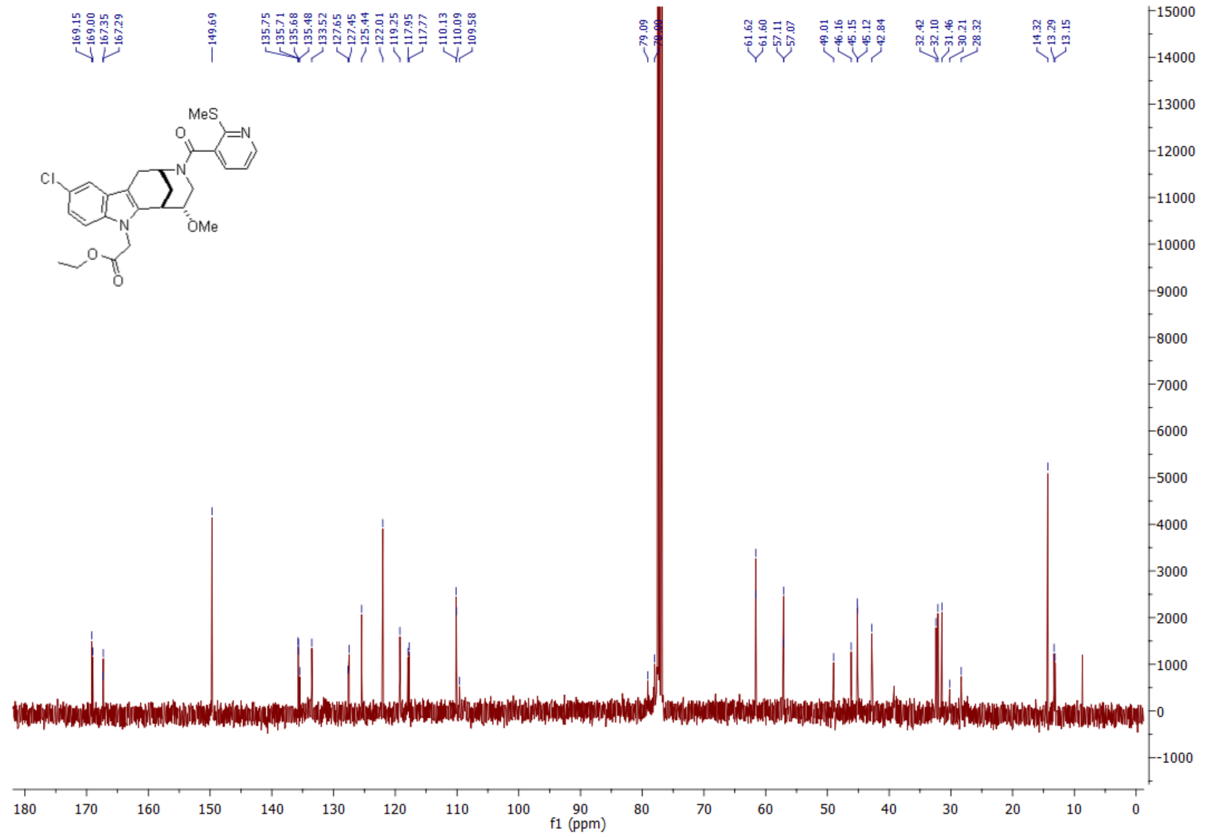
MeS
N
O
Cl
N
OMe
O
O
f1 (ppm)

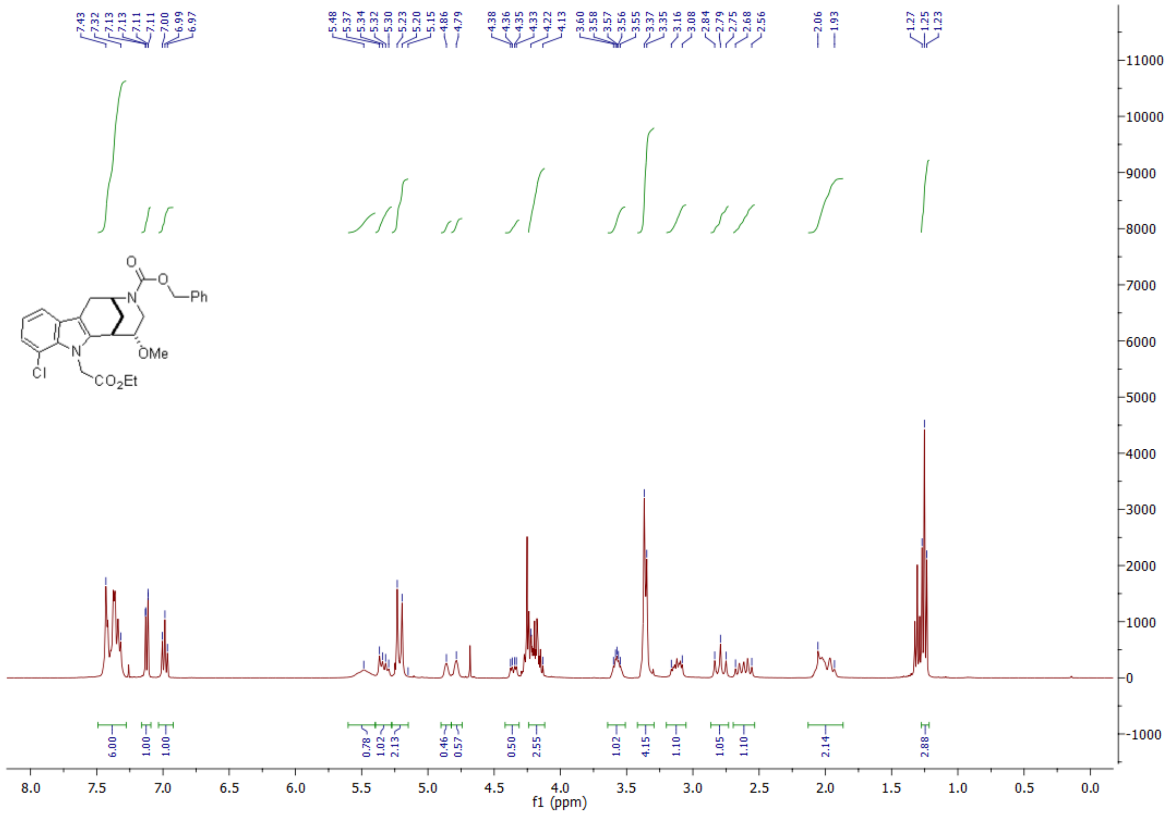

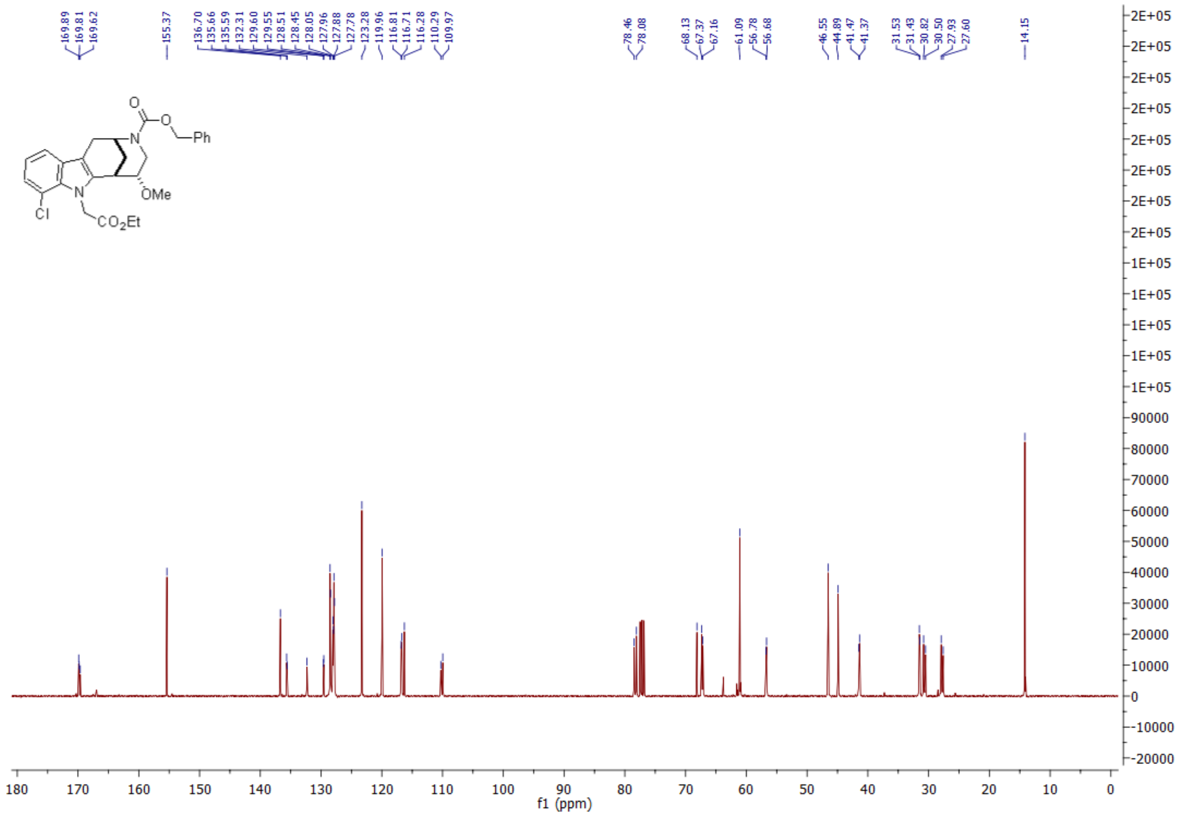

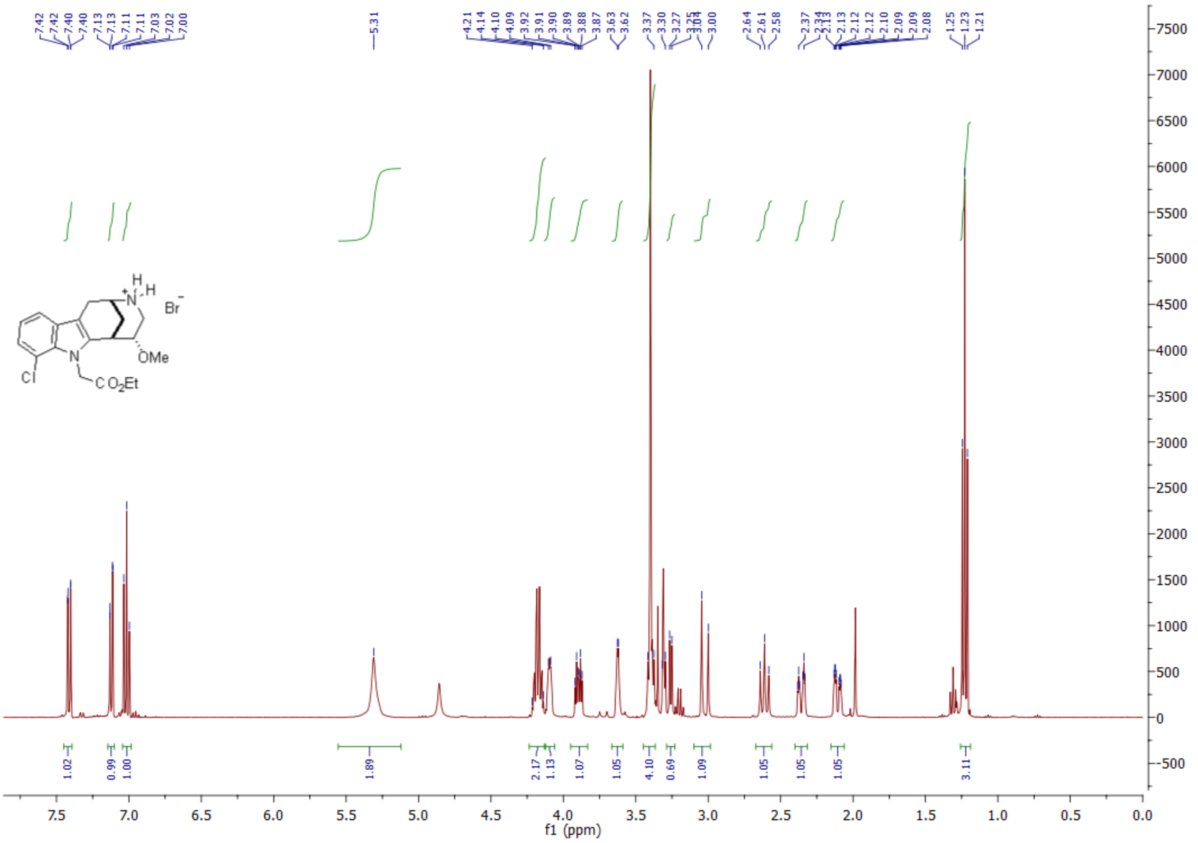

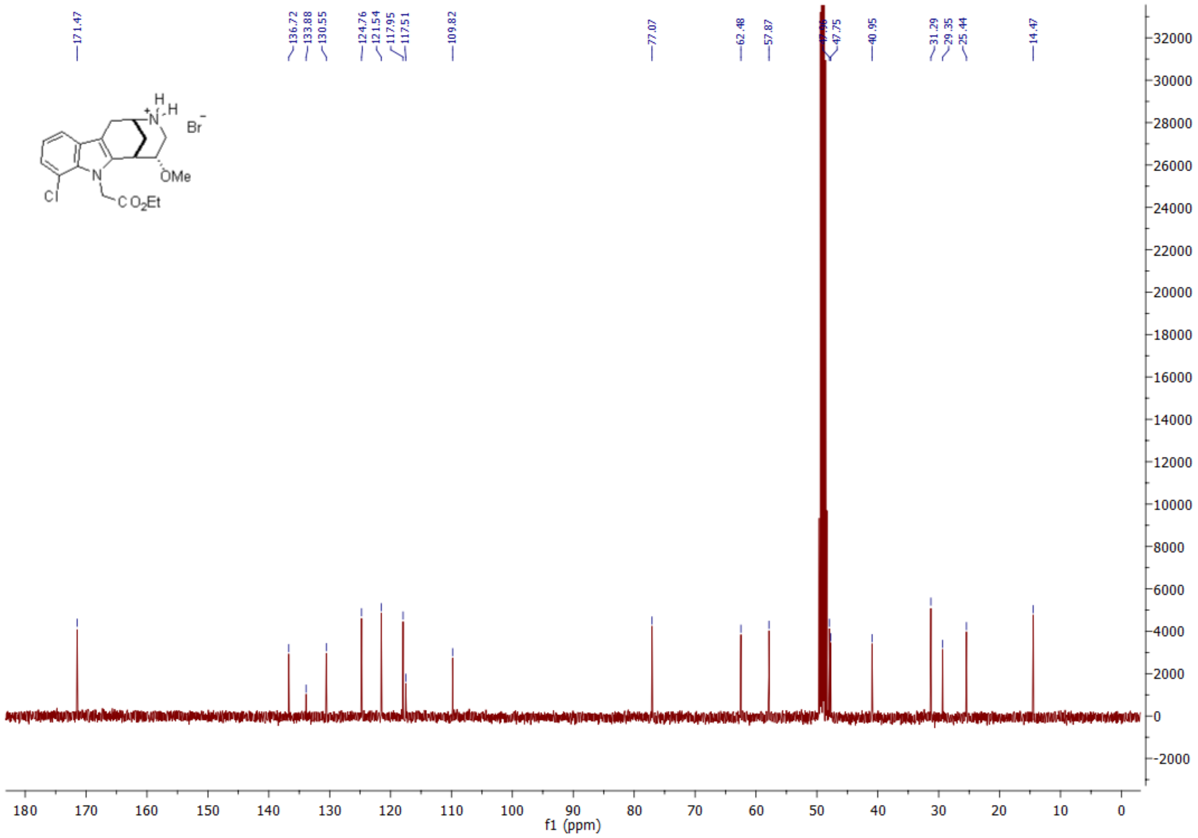

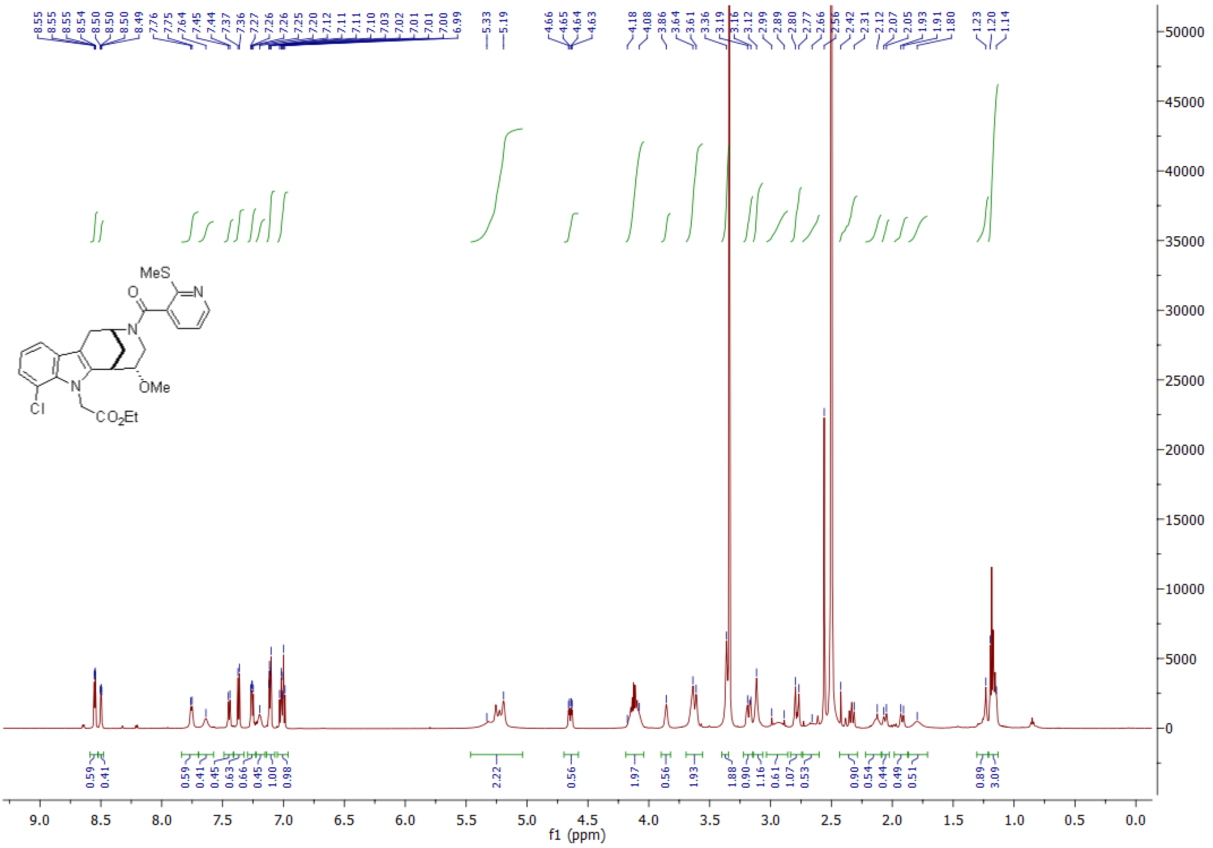

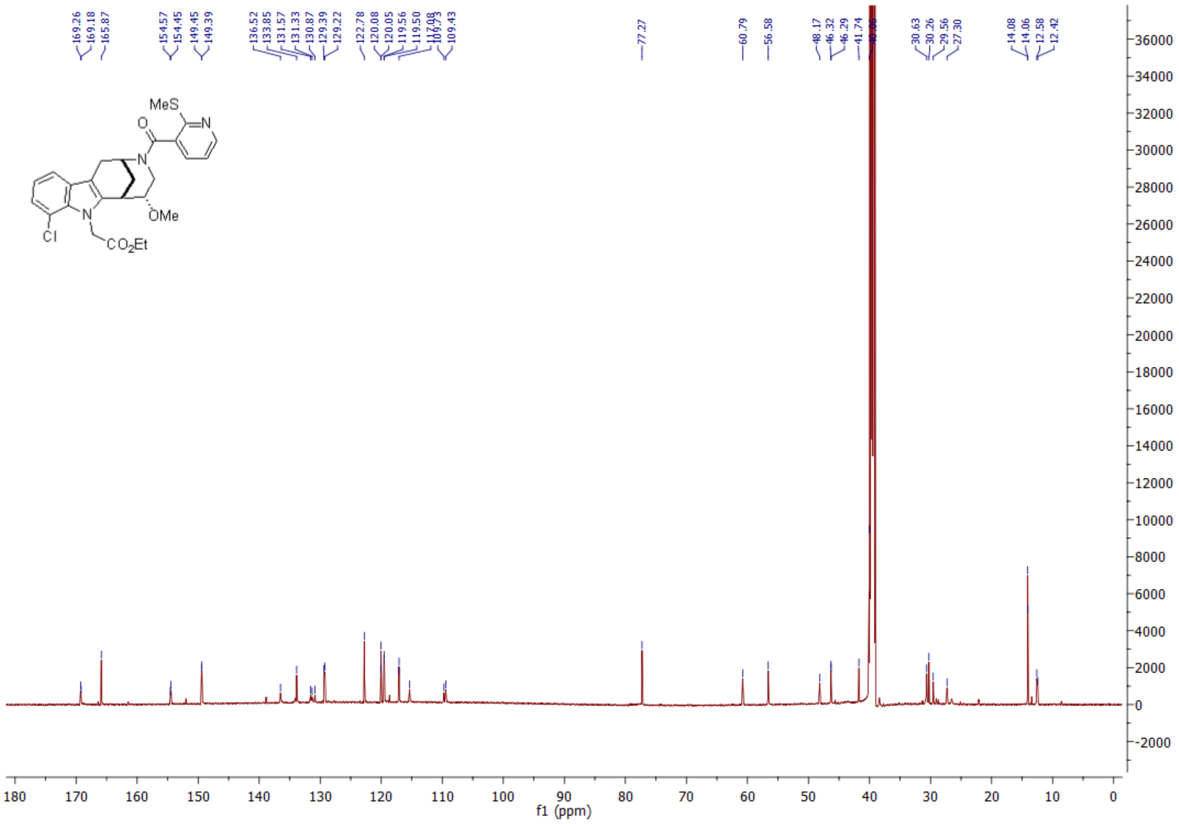

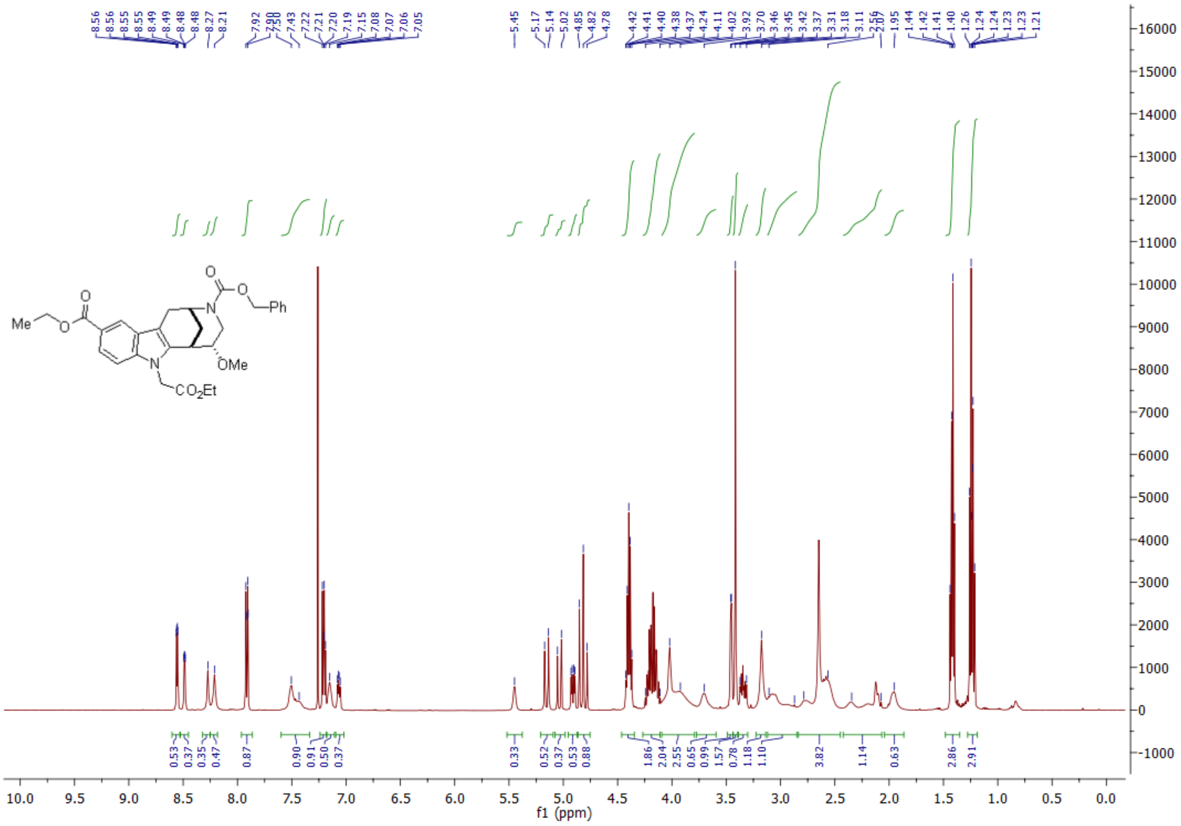

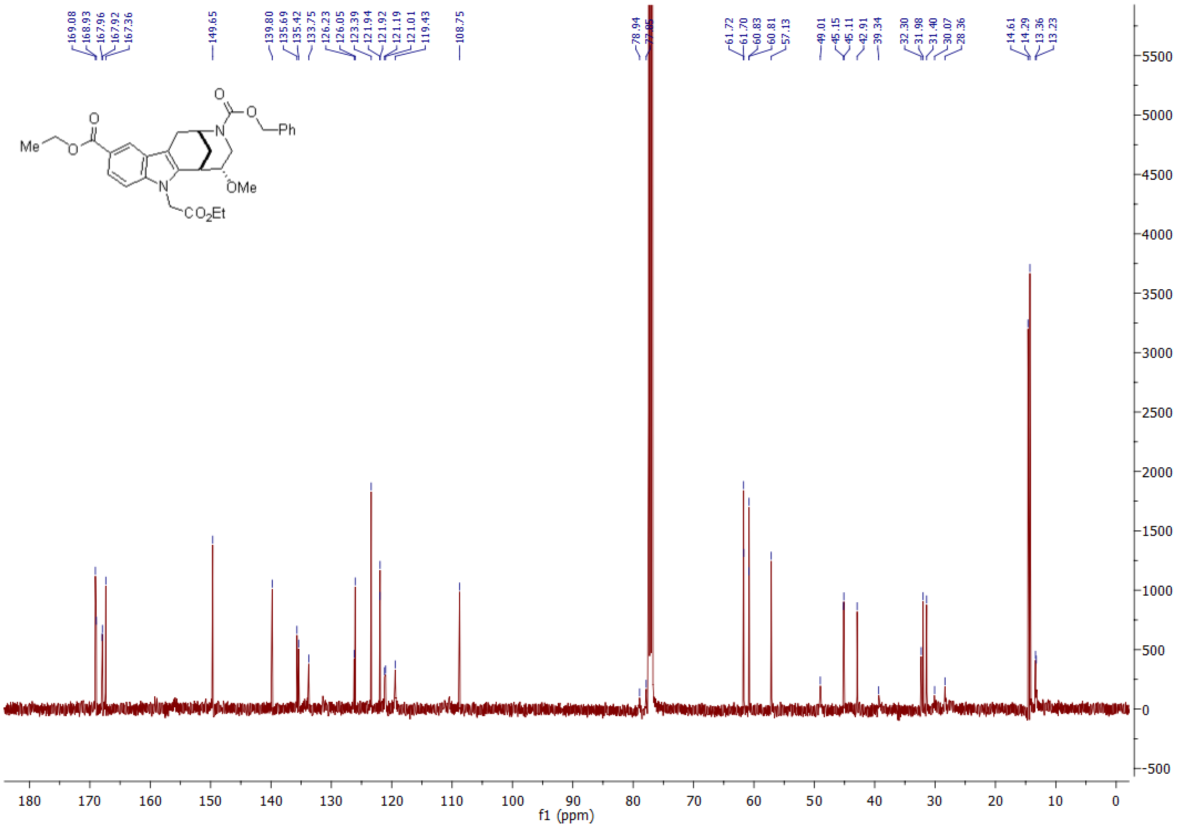

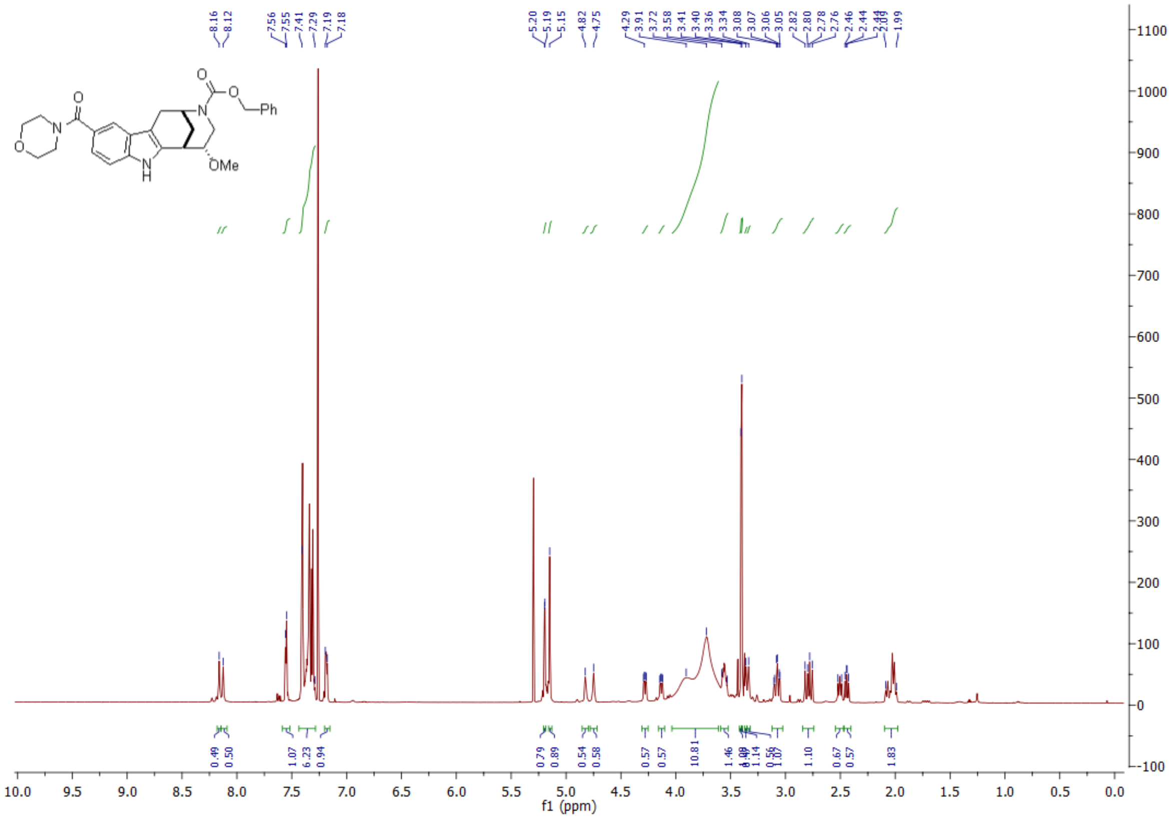

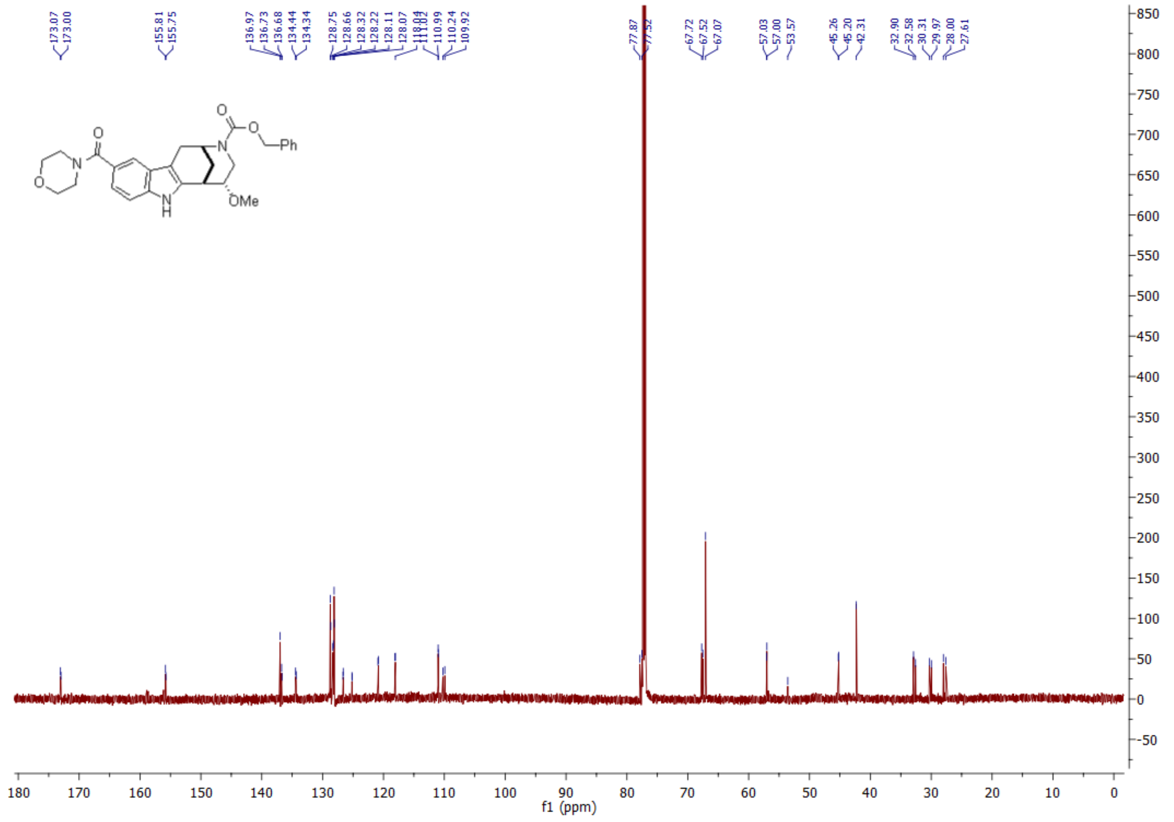

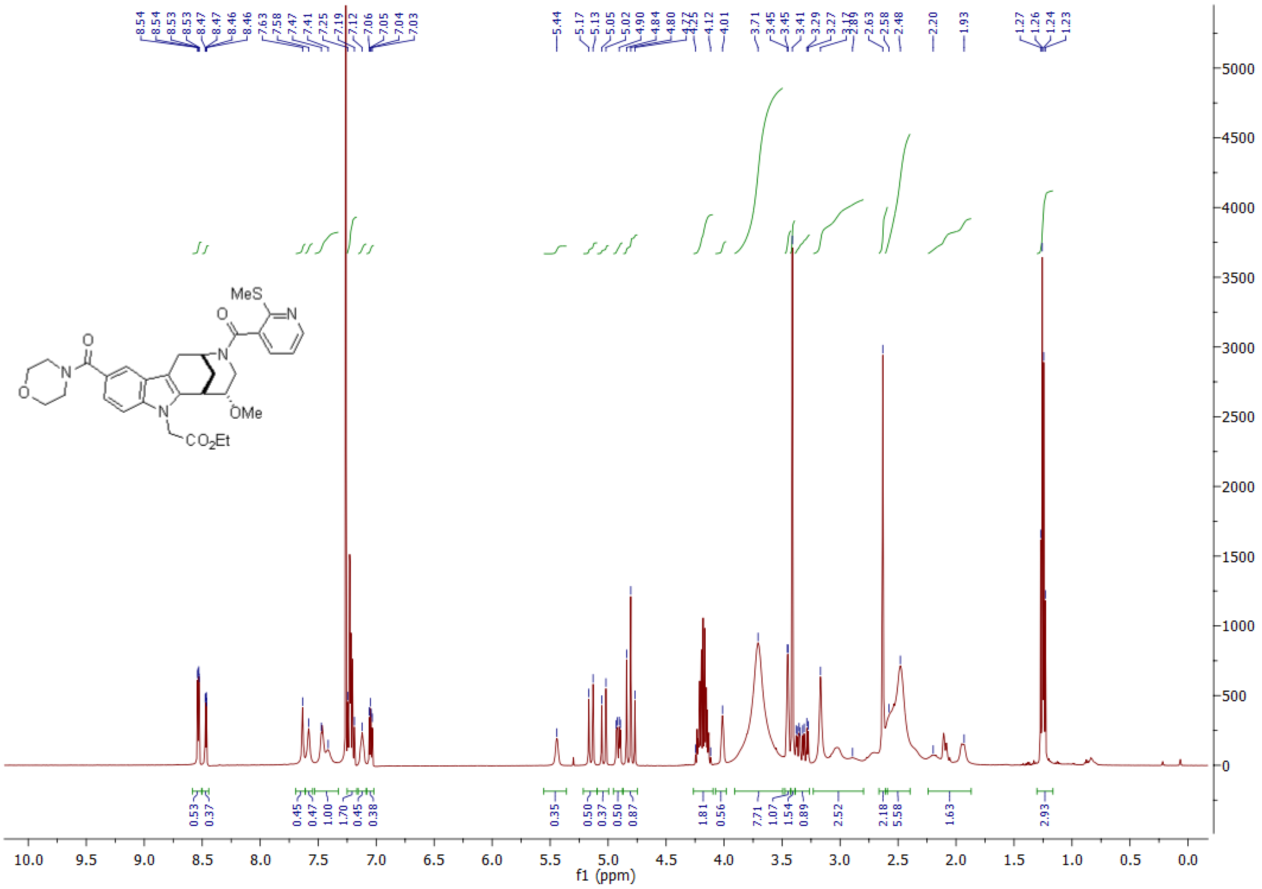

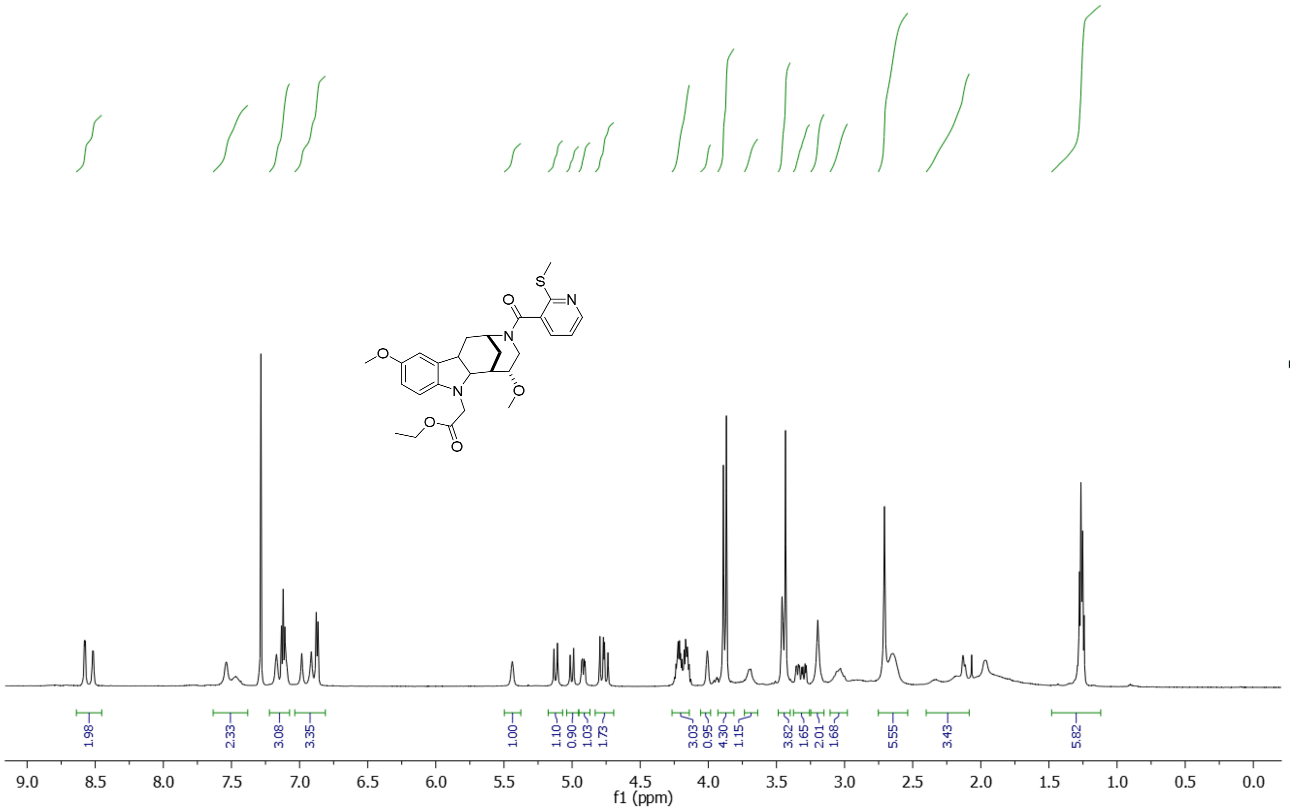
1.98
2.33
3.08
3.35
1.00
1.10
0.90
1.03
1.73
3.03
0.95
4.30
1.15
3.82
1.65
2.01
1.68
5.55
3.43
5.82
f1 (ppm)

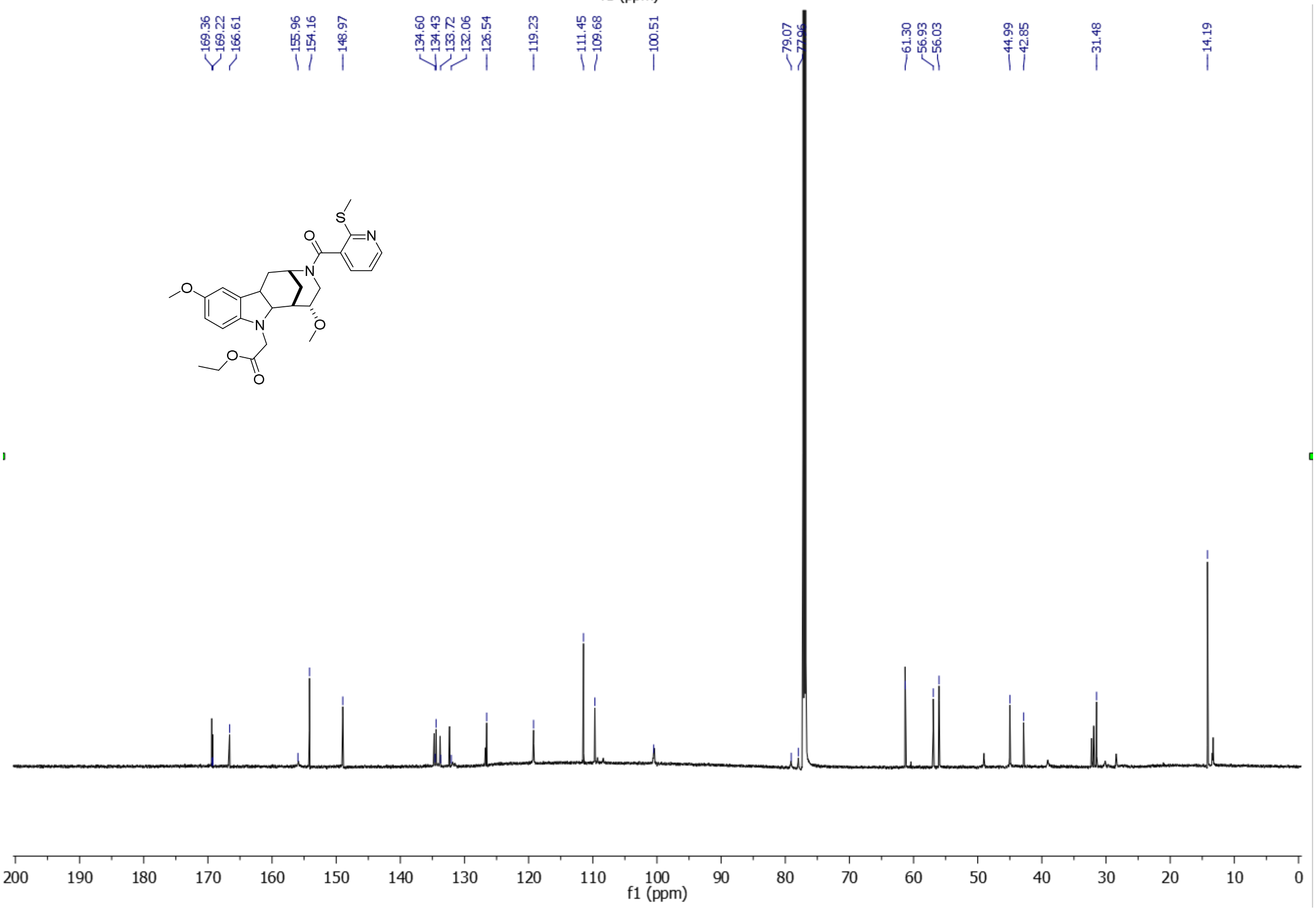
169.36
169.22
166.61
155.96
154.16
148.97
134.60
134.43
133.72
132.06
126.54
119.23
111.45
109.68
100.51
79.07
77.06
61.30
56.93
56.03
44.99
42.85
31.48
14.19
f1 (ppm)

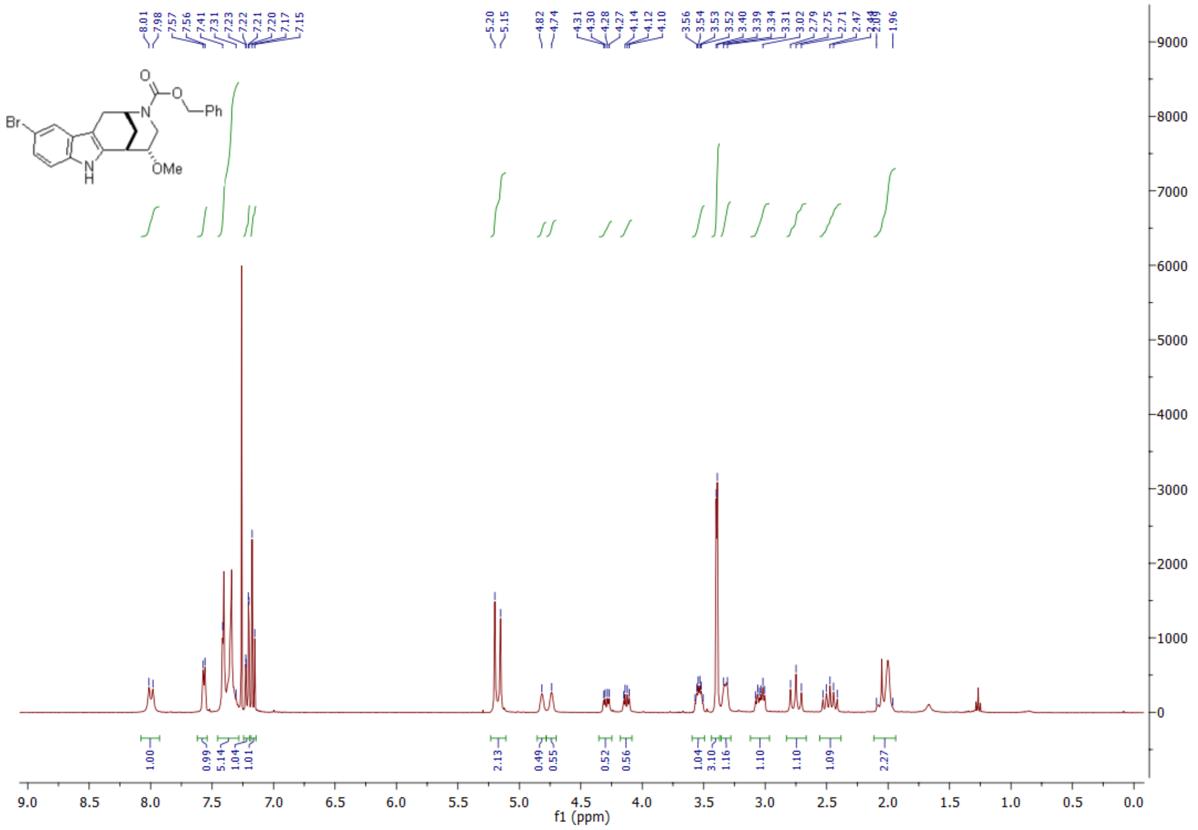
8.01
7.98
7.57
7.56
7.41
7.31
7.23
7.22
7.21
7.20
7.17
7.15
5.20
5.15
4.82
4.74
4.31
4.30
4.28
4.27
4.14
4.12
4.10
3.56
3.54
3.53
3.52
3.40
3.39
3.34
3.31
3.02
2.79
2.75
2.71
2.47
1.96
Br
O
O
N
Ph
N
H
OMe
1.00
0.99
5.14
1.04
1.01
2.13
0.49
0.55
0.52
0.56
1.04
3.10
1.16
1.10
1.10
1.09
2.27
f1 (ppm)

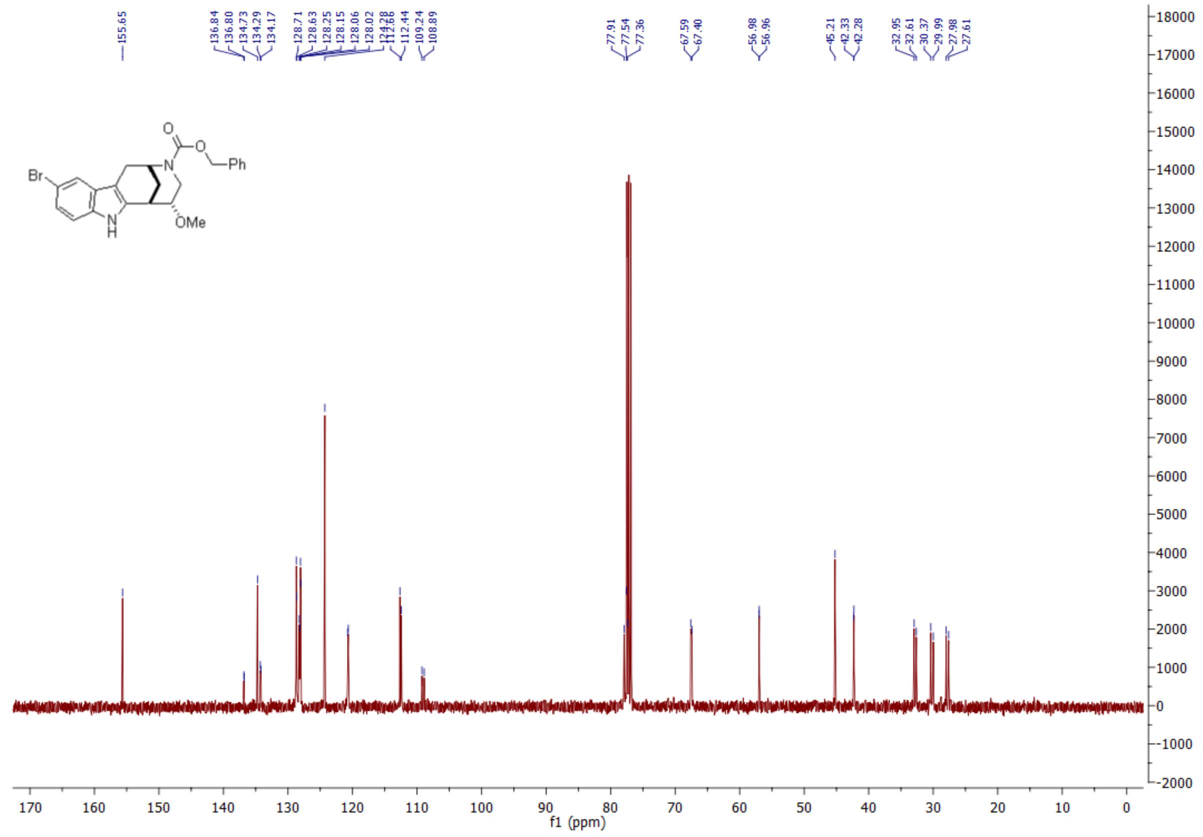
155.65
136.84
136.80
134.73
134.29
134.17
128.71
128.63
128.25
128.15
128.06
128.02
112.44
109.24
108.89
77.91
77.54
77.36
67.59
67.40
56.98
56.96
45.21
42.33
42.28
32.95
32.61
30.37
29.99
27.98
27.61
Br
O
O
N
Ph
N
H
OMe
f1 (ppm)

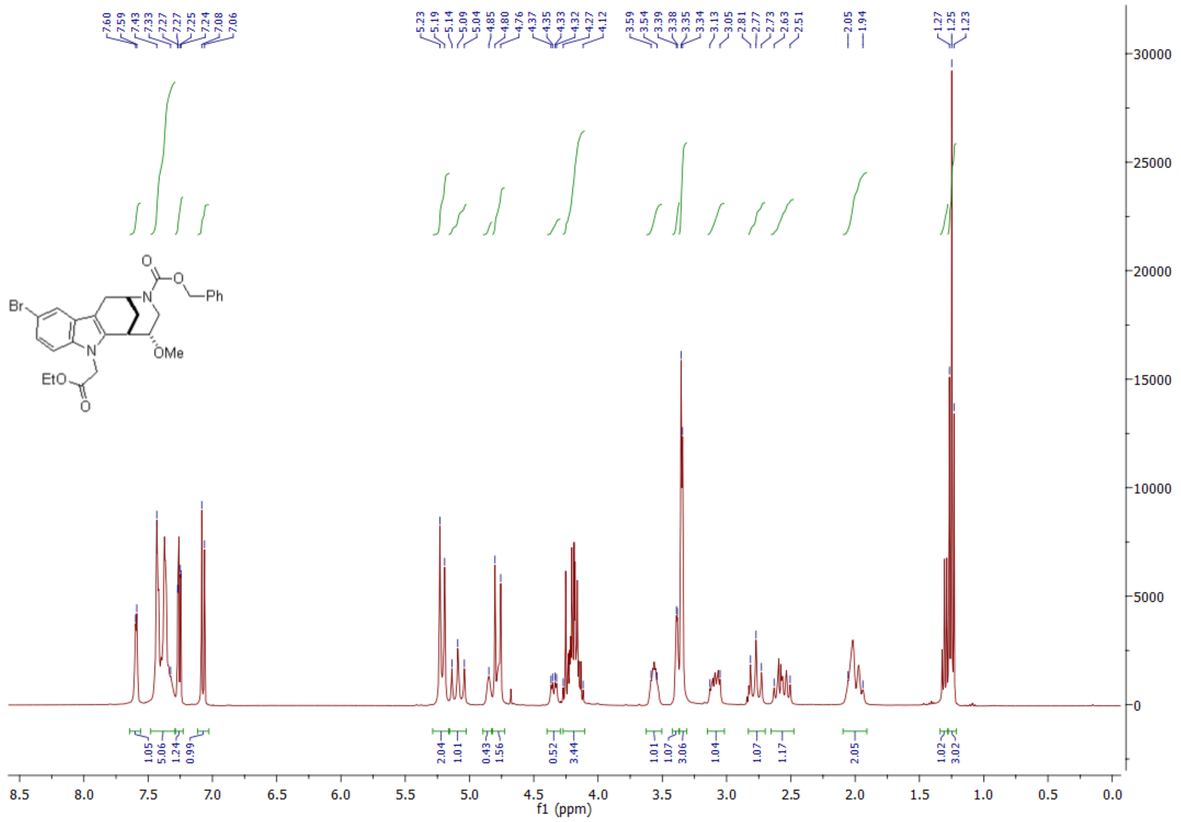
7.60 7.59 7.43 7.33 7.27 7.27 7.25 7.24 7.08 7.06
5.23 5.19 5.14 5.09 5.04 4.85 4.80 4.76 4.37 4.35 4.33 4.32 4.27 4.12
3.59 3.54 3.39 3.38 3.35 3.34 3.13 3.05 2.81 2.77 2.73 2.63 2.51
2.05 1.94
1.27 1.25 1.23
Br
N
O
O
Ph
OMe
EtO
O
1.05 5.06 1.24 0.99 2.04 1.01 0.43 1.56 0.52 3.44 1.01 1.07 3.06 1.04 1.07 1.17 2.05 1.02 3.02
f1 (ppm)

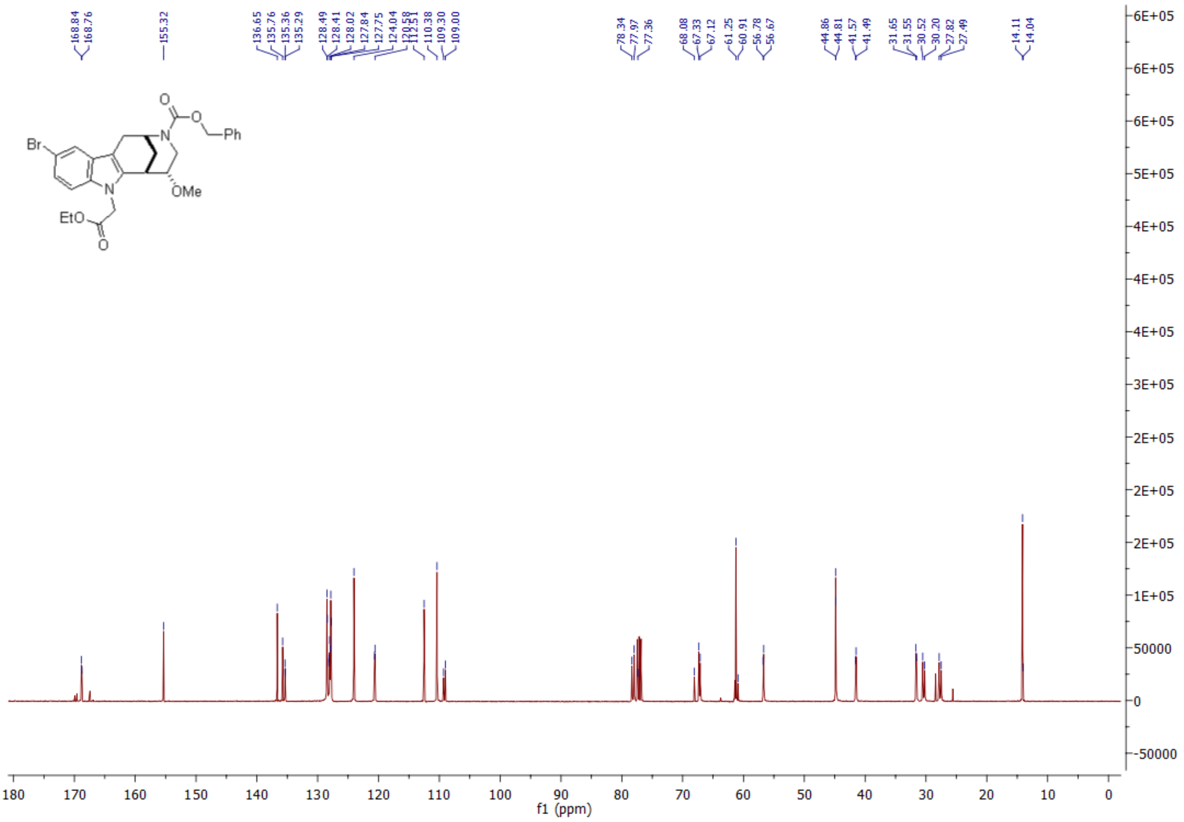
168.84 168.76
155.32
136.65 135.76 135.36 135.29 128.49 128.41 128.02 127.84 127.75 124.04 110.38 109.30 109.00
78.34 77.97 77.36
68.08 67.33 67.12 61.25 60.91 56.78 56.67
44.86 44.81 41.57 41.49
31.65 31.55 30.52 30.20 27.82 27.49
14.11 14.04
Br
N
O
O
Ph
OMe
EtO
O
f1 (ppm)

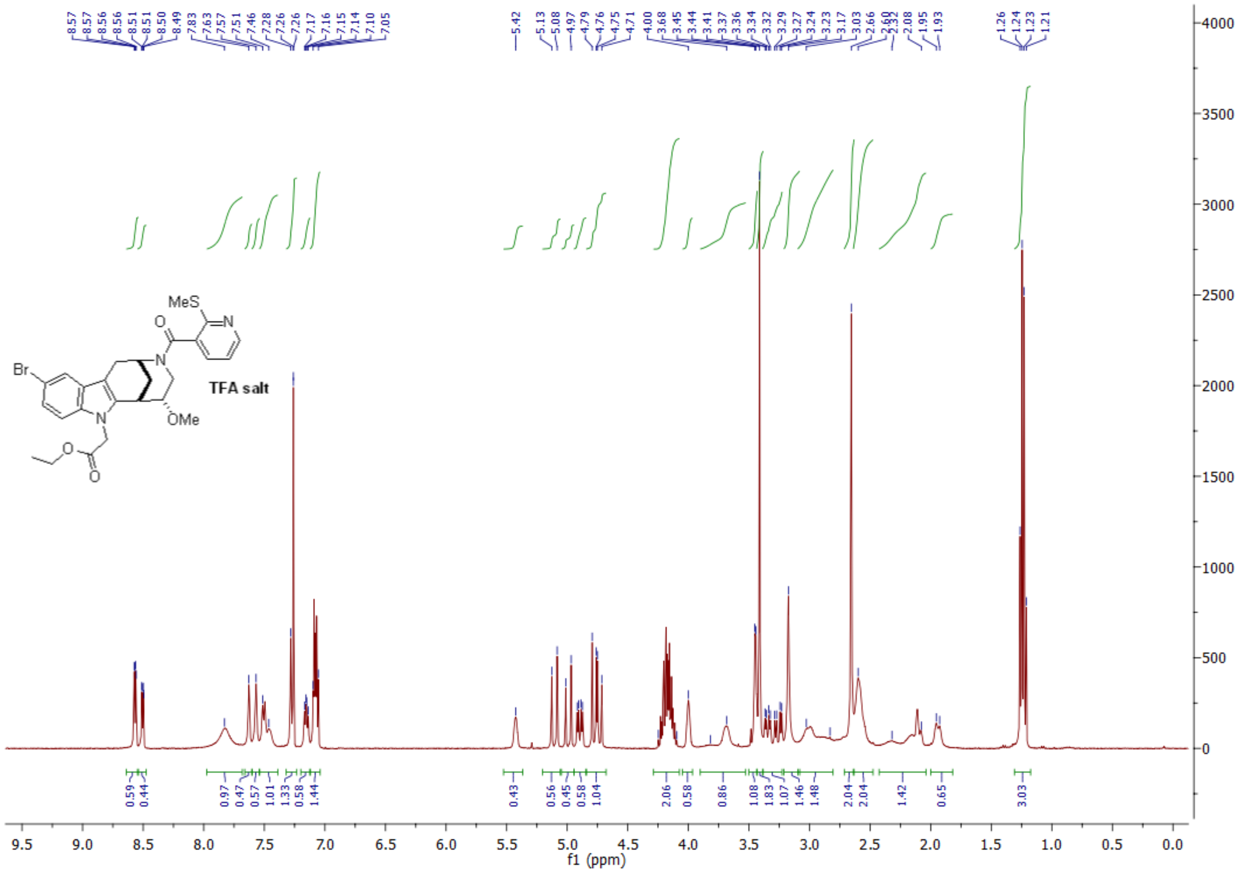
MeS
O
N
Br
N
OMe
O
O
TFA salt
f1 (ppm)

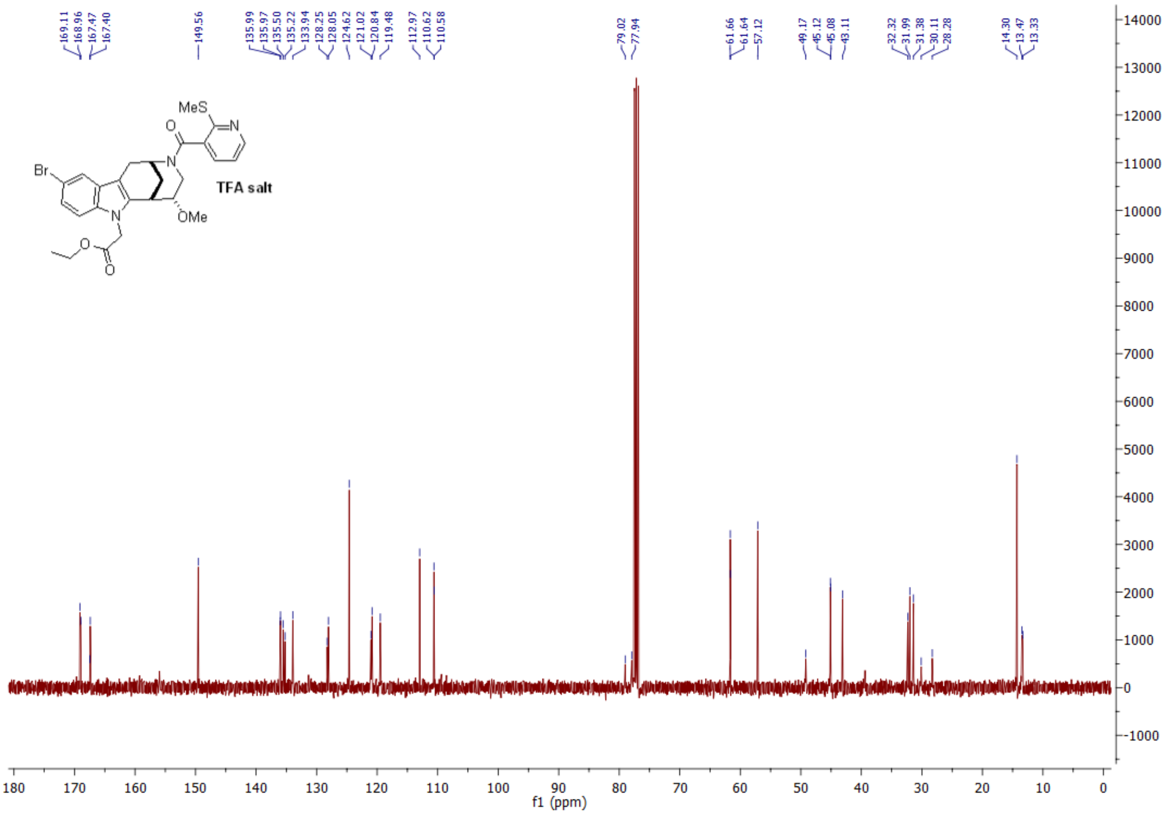
MeS
O
N
Br
N
OMe
O
O
TFA salt
f1 (ppm)

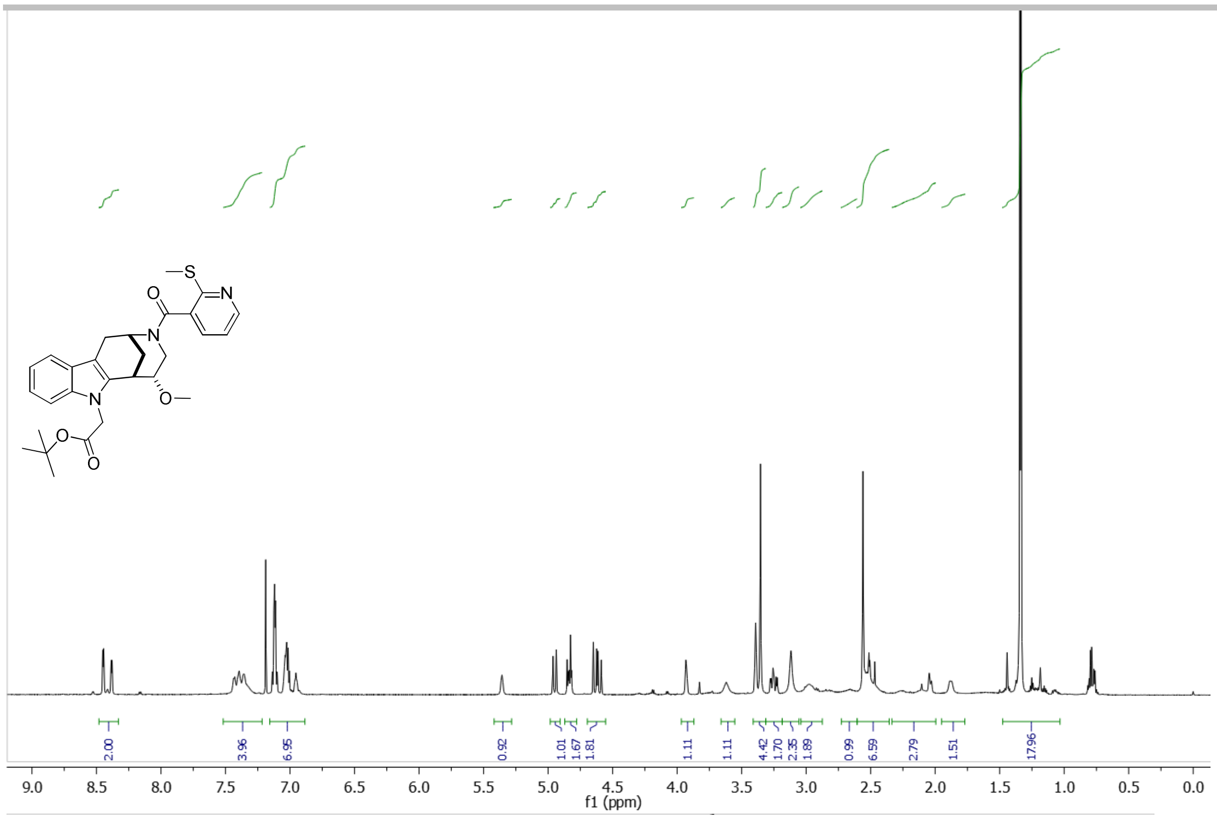
2.00
3.96
6.95
0.92
1.01
1.67
1.81
1.11
1.11
4.42
1.70
2.35
1.89
0.99
6.59
2.79
1.51
17.96
9.0 8.5 8.0 7.5 7.0 6.5 6.0 5.5 5.0 4.5 4.0 3.5 3.0 2.5 2.0 1.5 1.0 0.5 0.0
f1 (ppm)

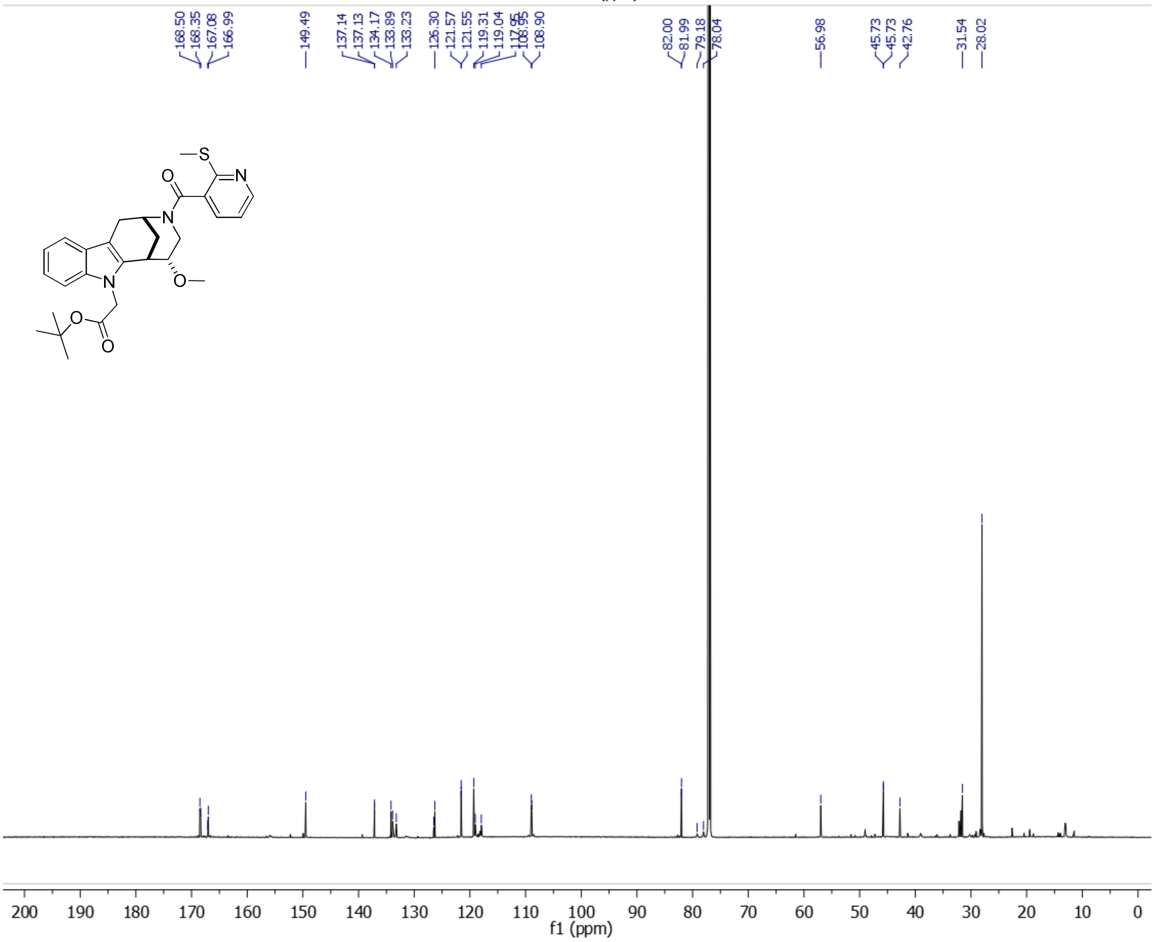
168.50
168.35
167.08
166.99
149.49
137.14
137.13
134.17
133.89
133.23
126.30
121.57
121.55
119.31
119.04
117.95
108.95
108.90
82.00
81.99
79.18
78.04
56.98
45.73
45.73
42.76
31.54
28.02
200 190 180 170 160 150 140 130 120 110 100 90 80 70 60 50 40 30 20 10 0
f1 (ppm)

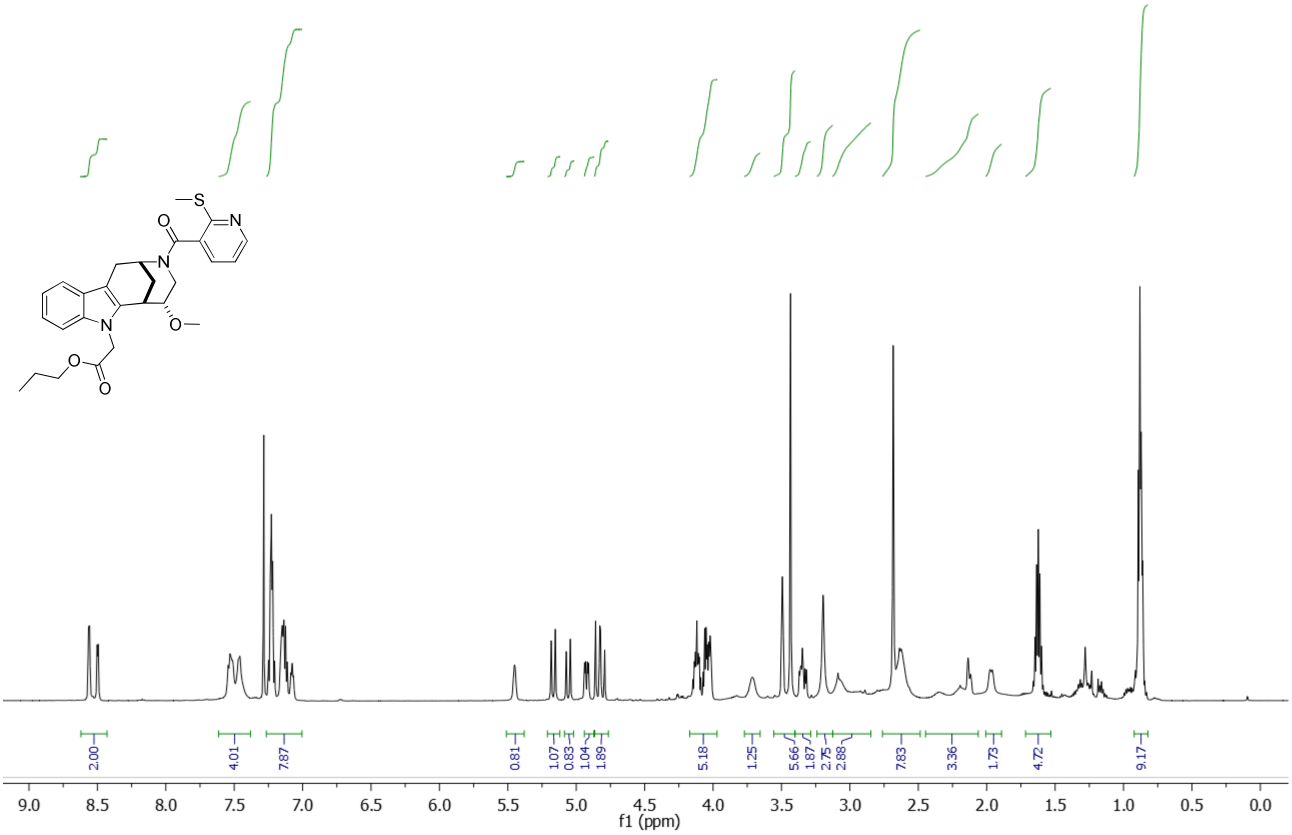

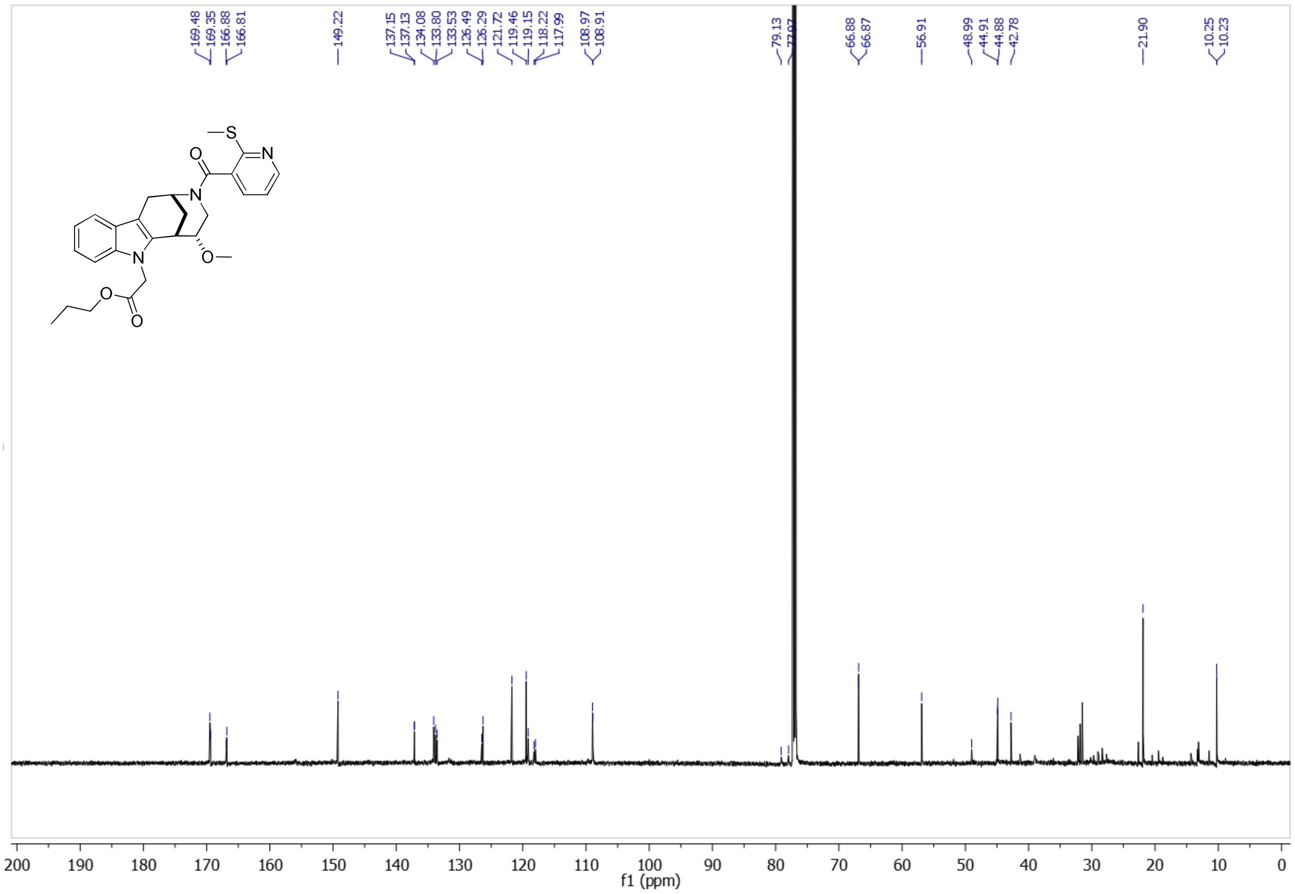

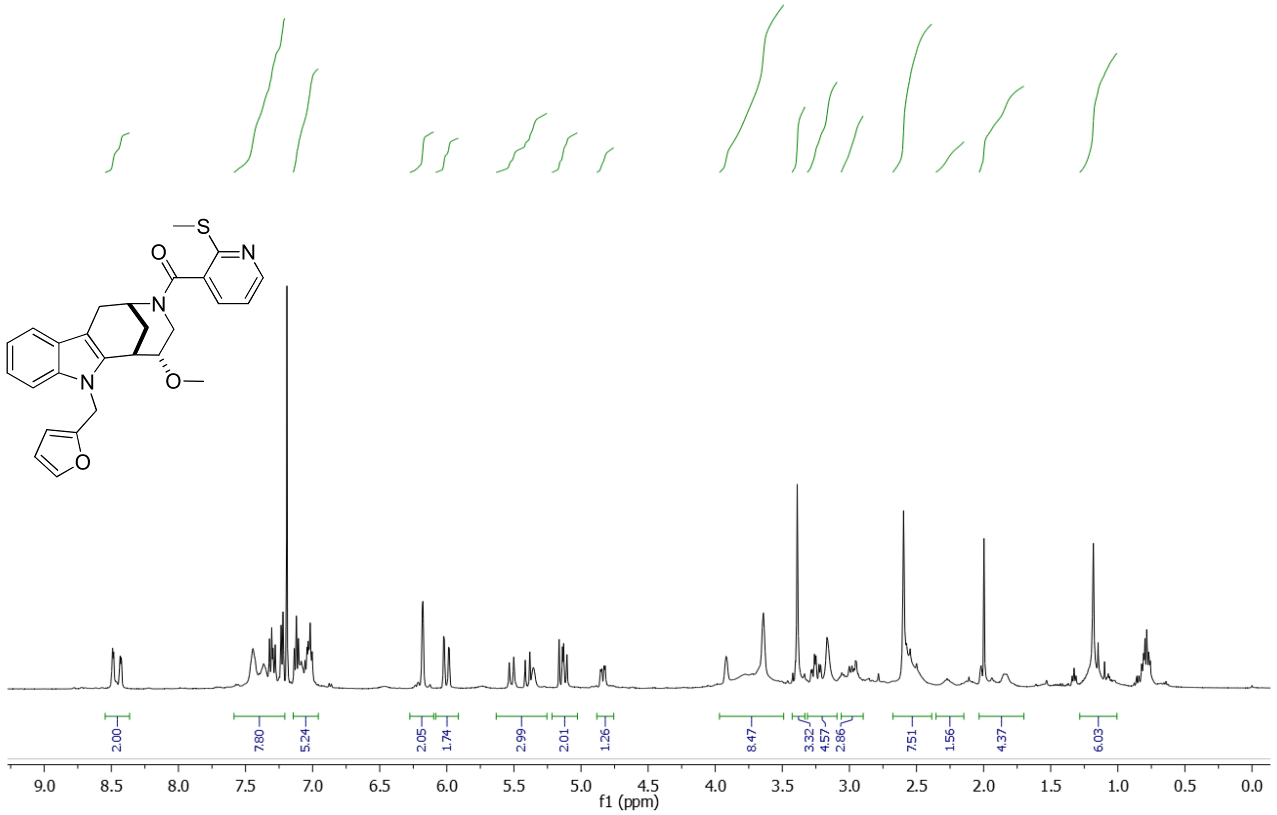
2.00
7.80
5.24
2.05
1.74
2.99
2.01
1.26
8.47
3.32
4.57
2.86
7.51
1.56
4.37
6.03
f1 (ppm)

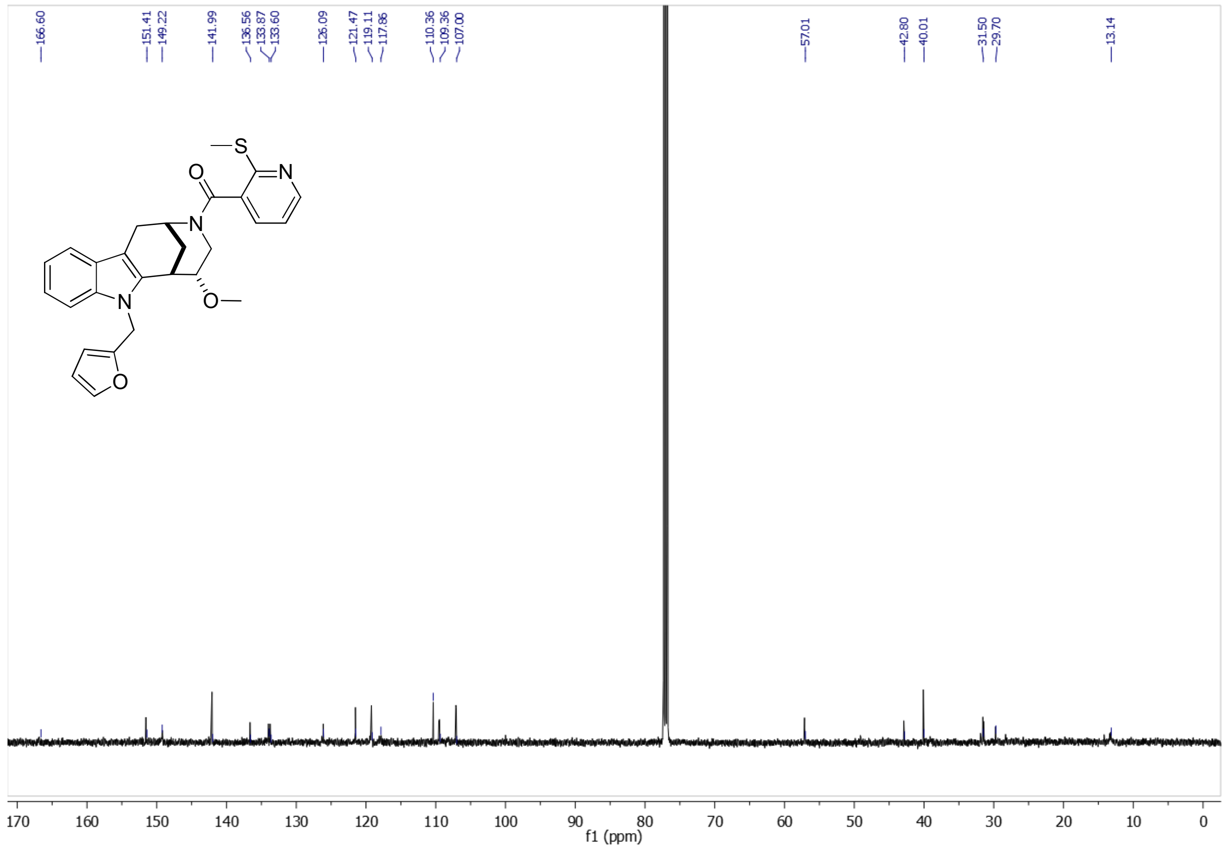
166.60
151.41
149.22
141.99
136.56
133.87
133.60
126.09
121.47
119.11
117.86
110.36
109.36
107.00
57.01
42.80
40.01
31.50
29.70
13.14
f1 (ppm)

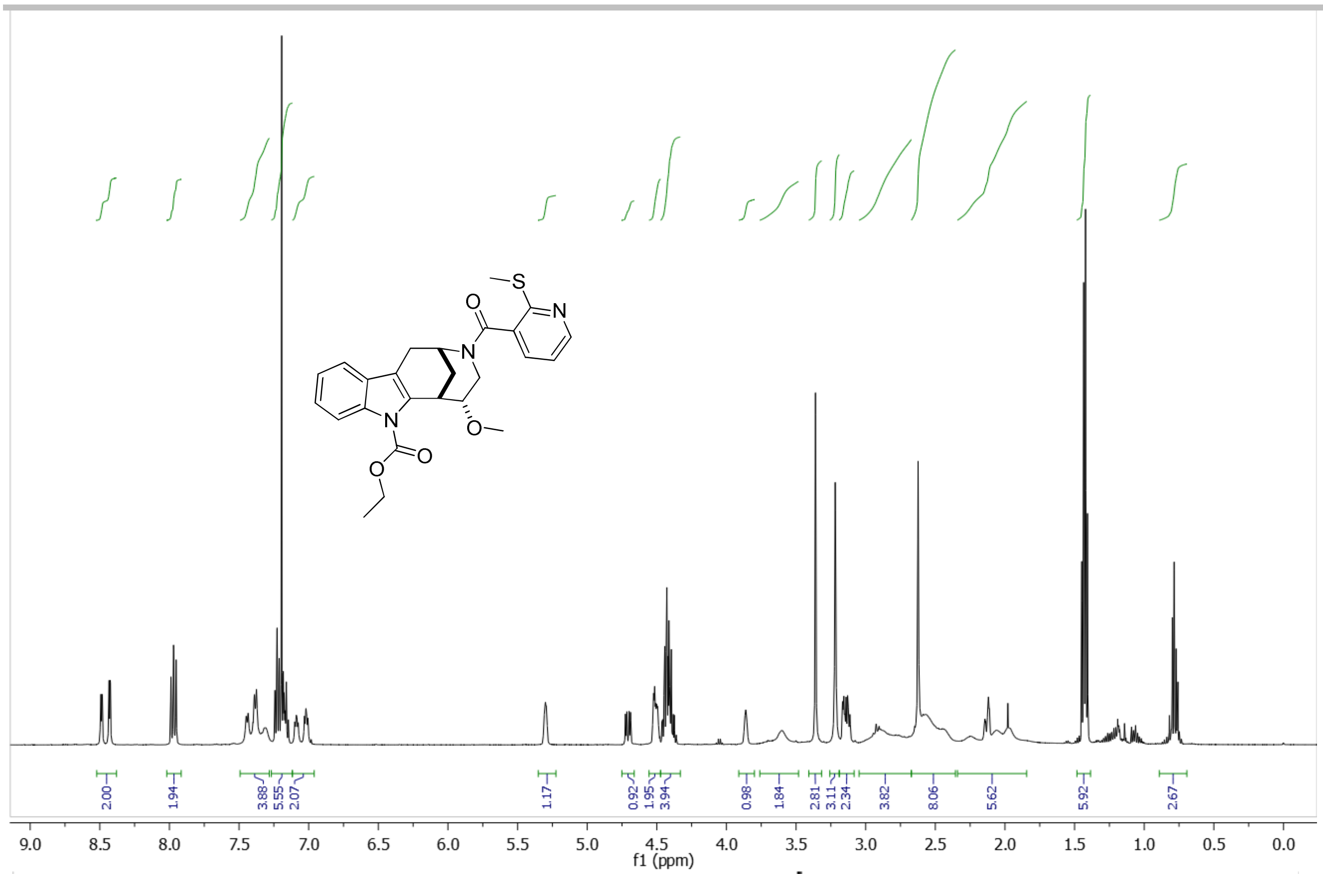

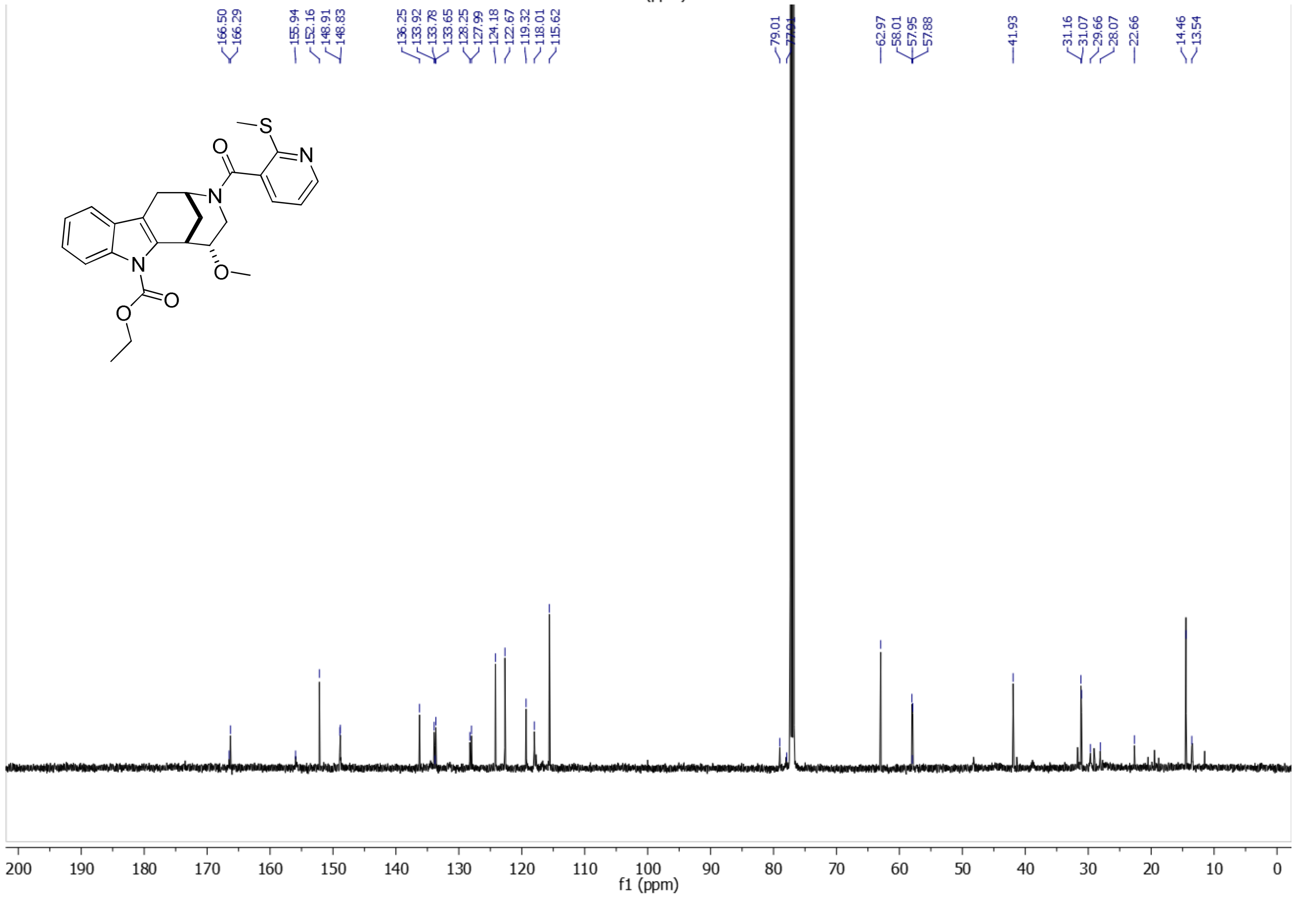

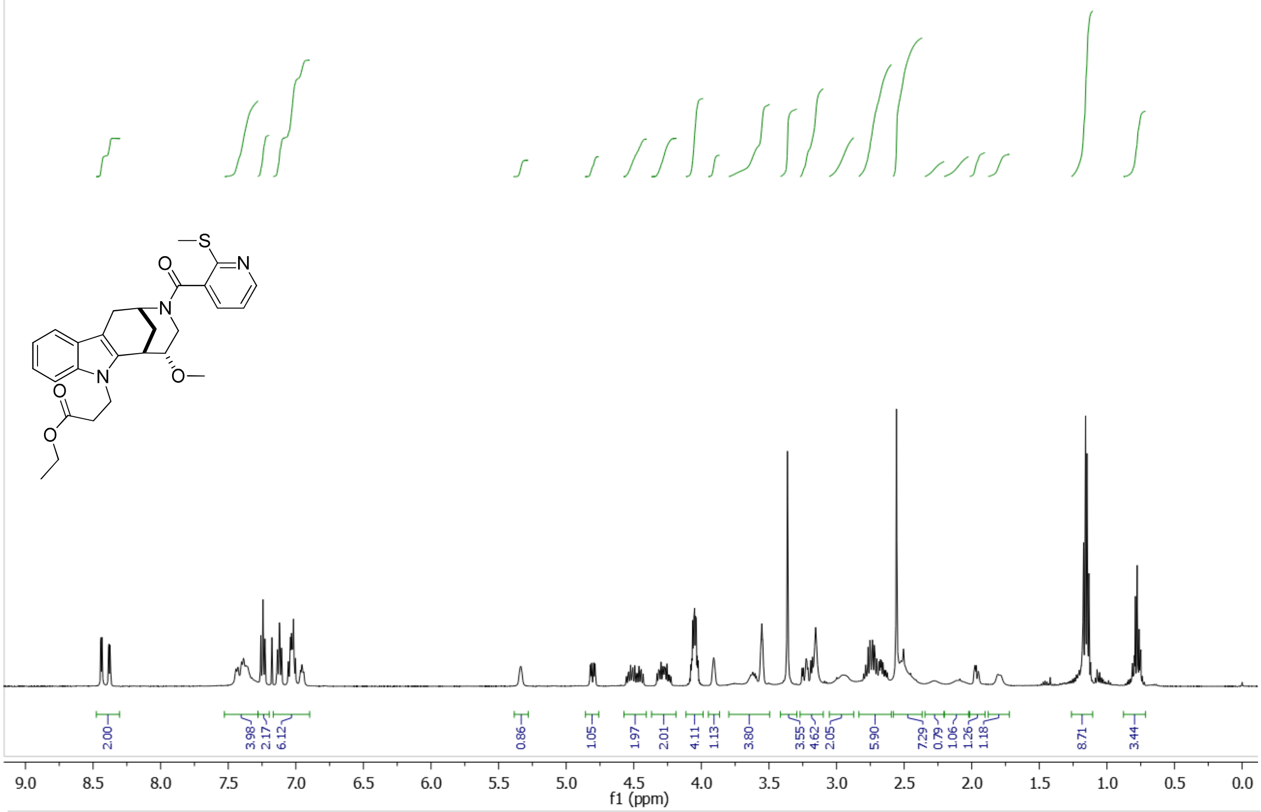
S
N
O
N
N
O
O
O
2.00
3.98
2.17
6.12
0.86
1.05
1.97
2.01
4.11
1.13
3.80
3.55
4.62
2.05
5.90
7.29
0.79
1.06
1.26
1.18
8.71
3.44
9.0
8.5
8.0
7.5
7.0
6.5
6.0
5.5
5.0
4.5
4.0
3.5
3.0
2.5
2.0
1.5
1.0
0.5
0.0
f1 (ppm)

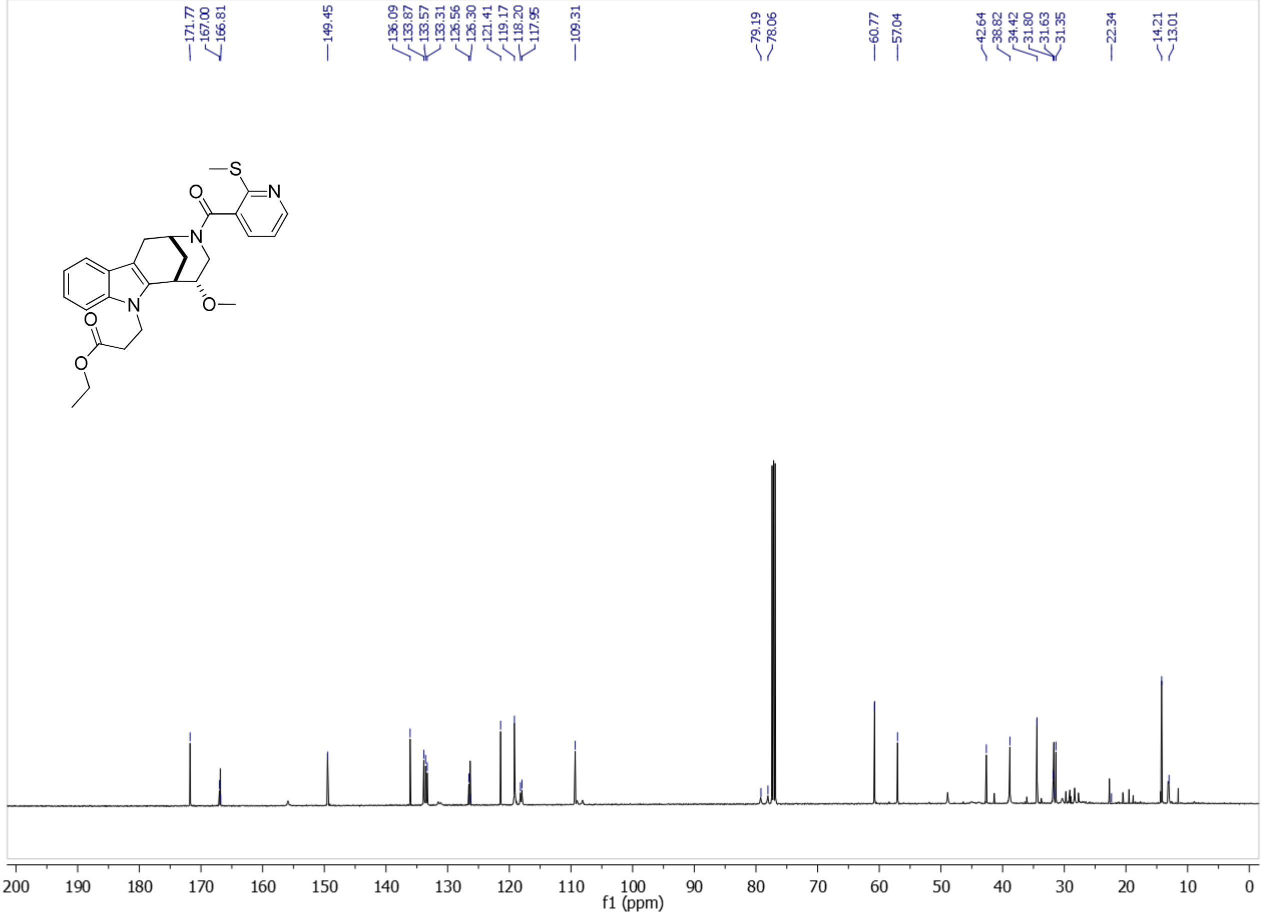
171.77
167.00
166.81
149.45
136.09
133.87
133.57
133.31
126.56
126.30
121.41
119.17
118.20
117.95
109.31
79.19
78.06
60.77
57.04
42.64
38.82
34.42
31.80
31.63
31.35
22.34
14.21
13.01
S
N
O
N
N
O
O
O
200
190
180
170
160
150
140
130
120
110
100
90
80
70
60
50
40
30
20
10
0
f1 (ppm)

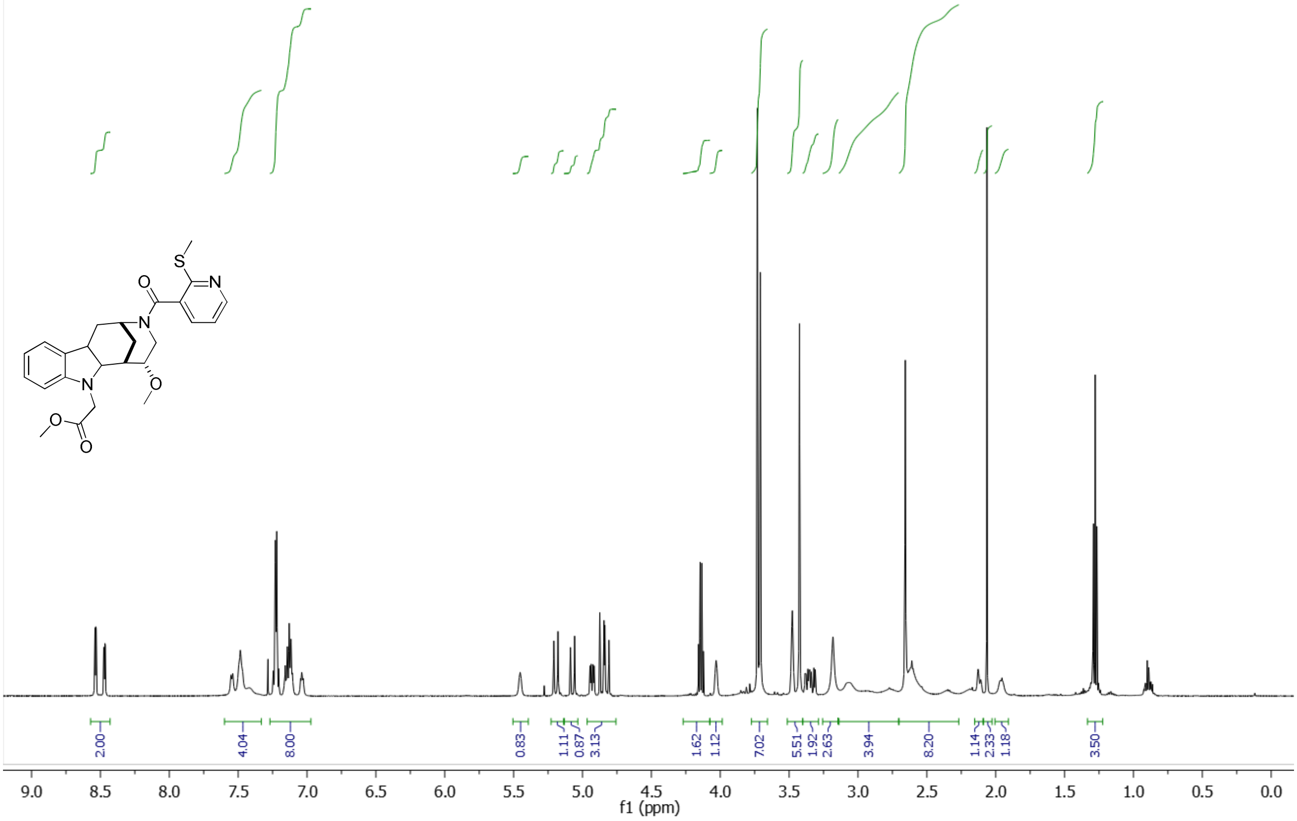
2.00
4.04
8.00
0.83
1.11
0.87
3.13
1.62
1.12
7.02
5.51
1.92
2.63
3.94
8.20
1.14
2.33
1.18
3.50
f1 (ppm)

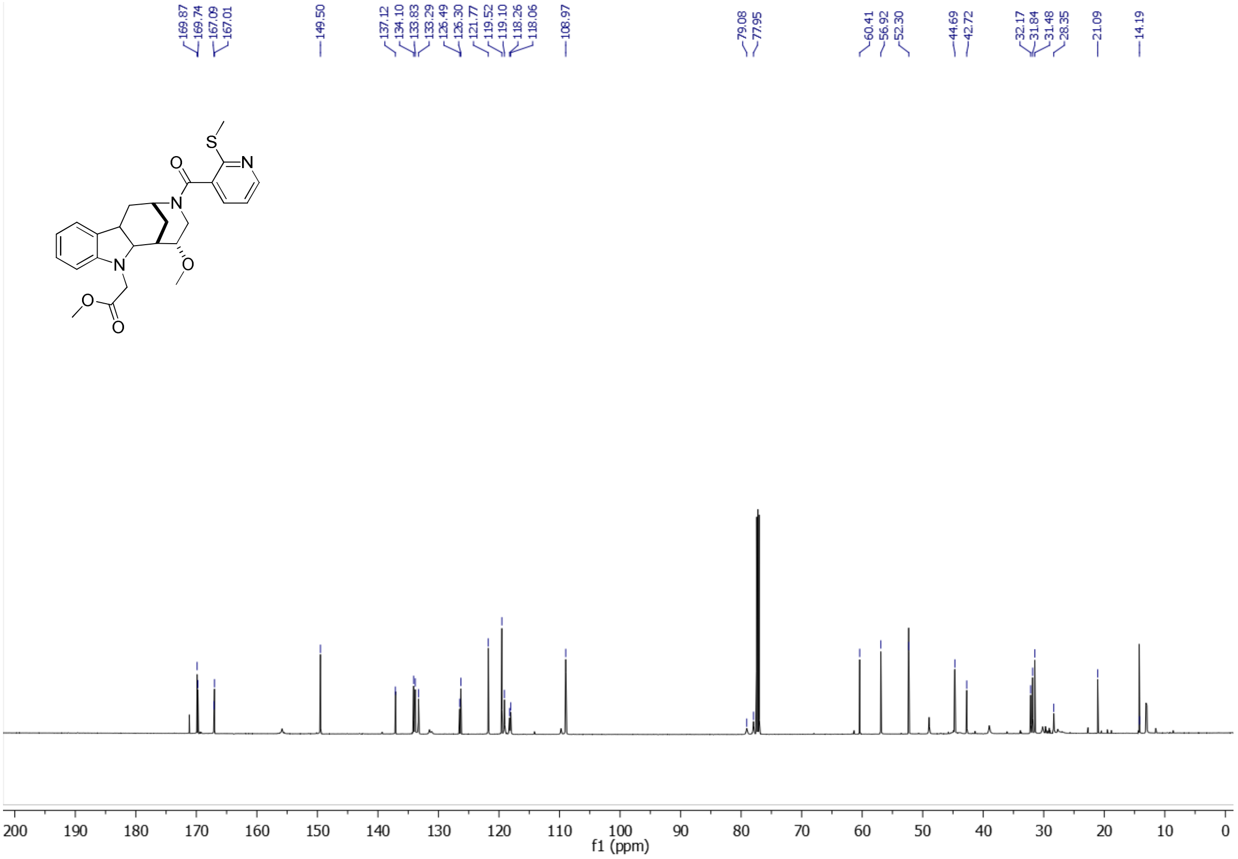
169.87
169.74
167.09
167.01
149.50
137.12
134.10
133.83
133.29
126.49
126.30
121.77
119.52
119.10
118.26
118.06
108.97
79.08
77.95
60.41
56.92
52.30
44.69
42.72
32.17
31.84
31.48
28.35
21.09
14.19
f1 (ppm)

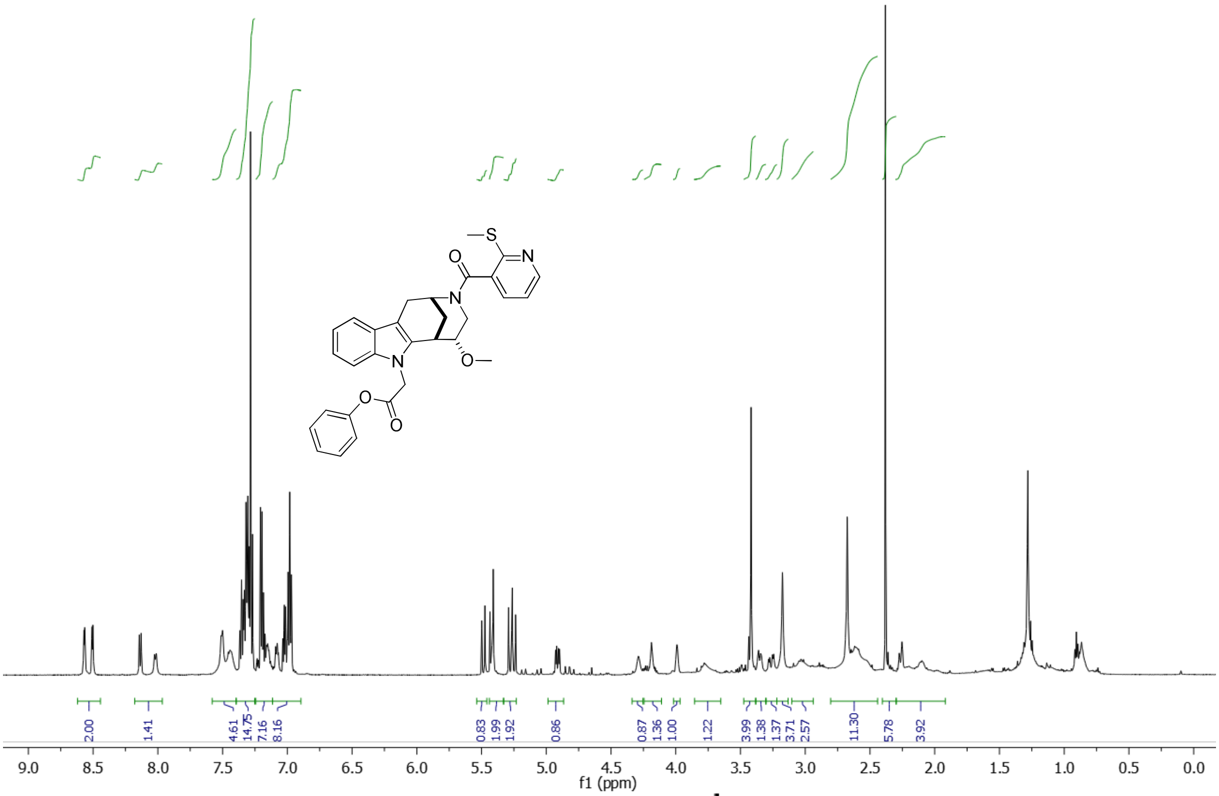

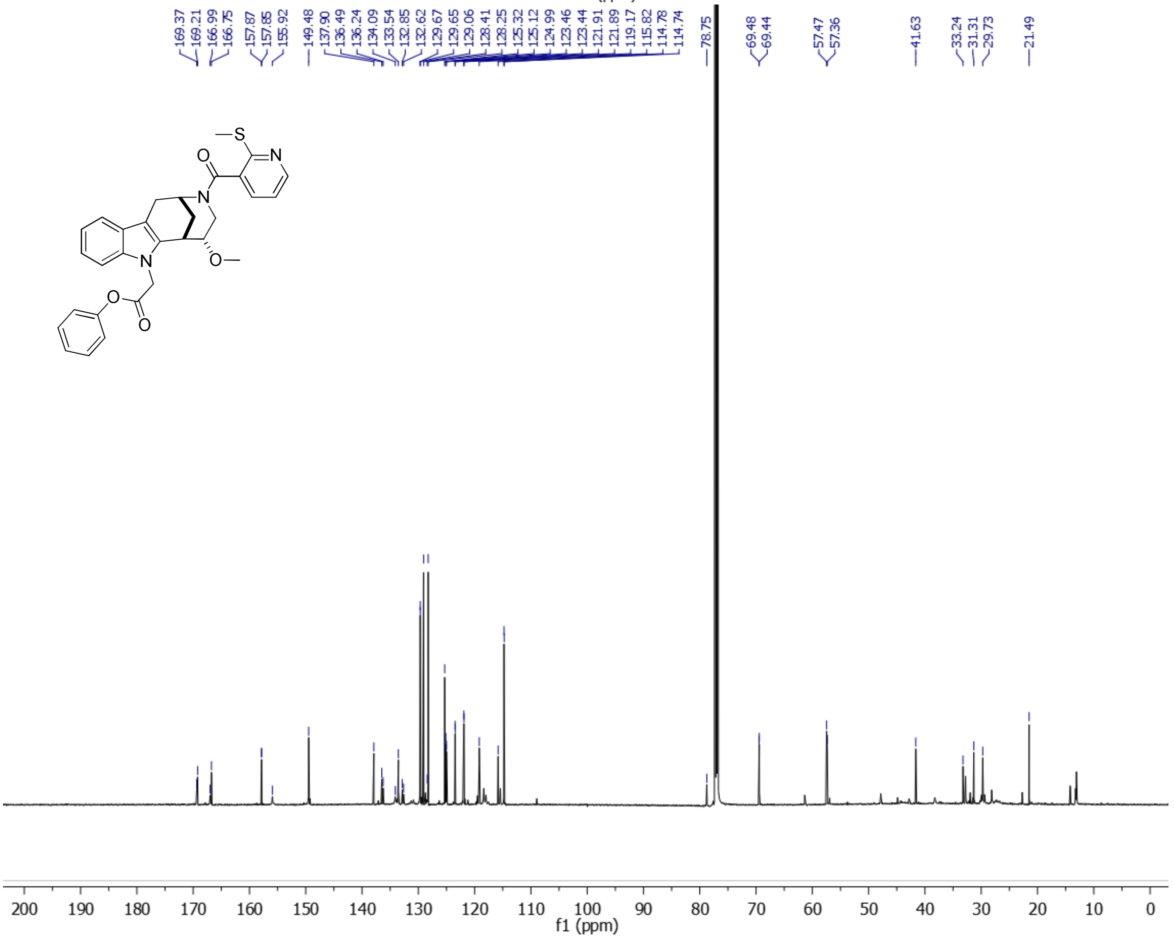

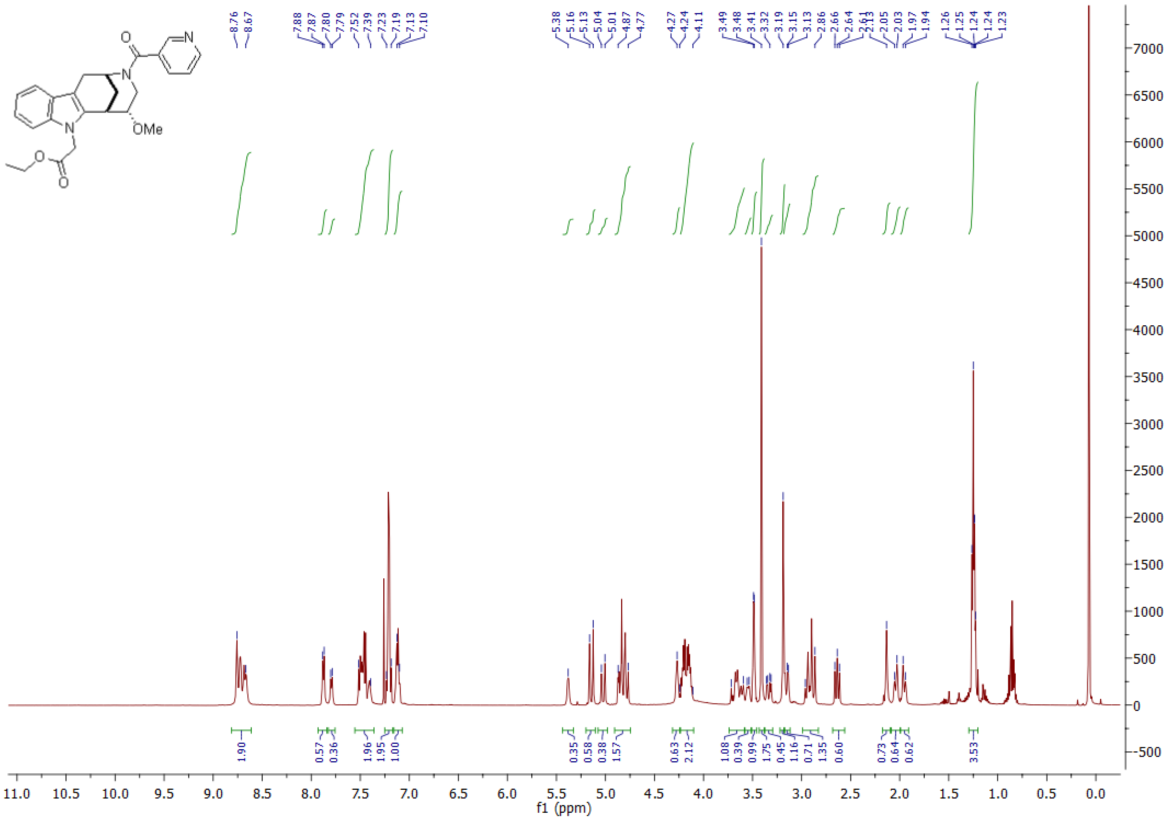

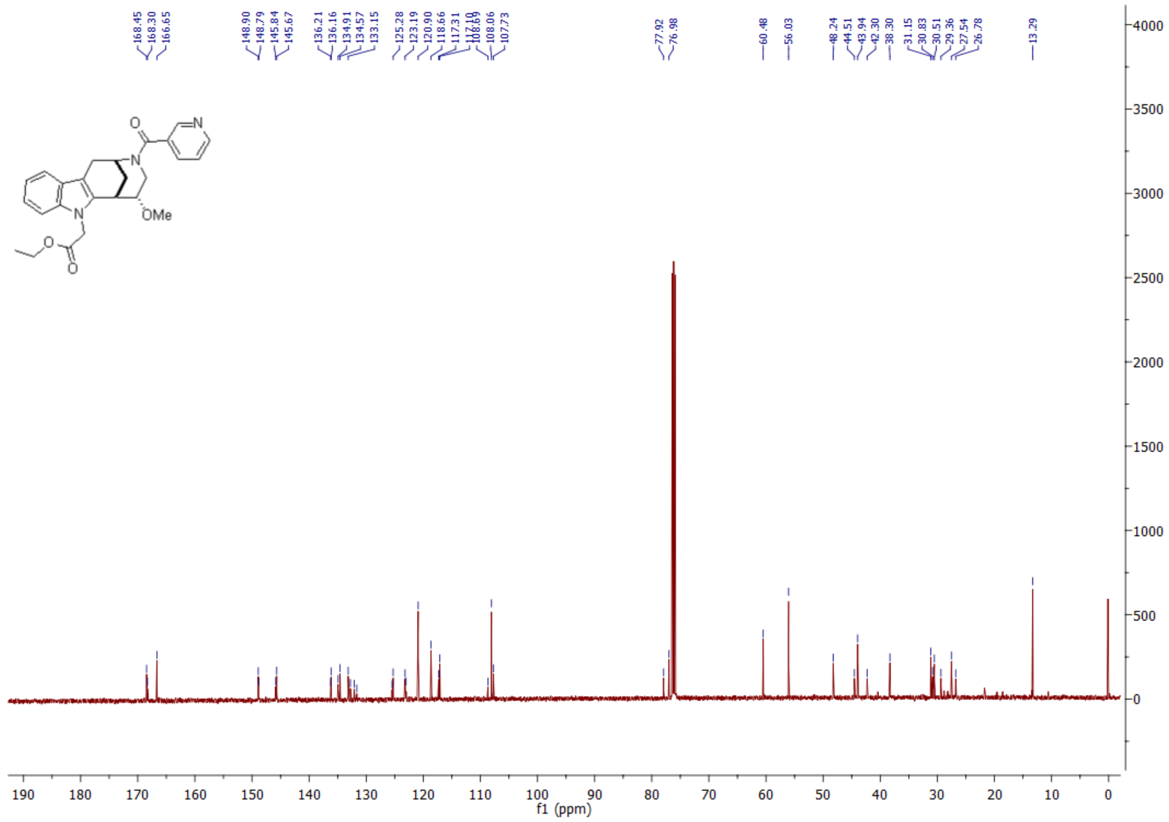

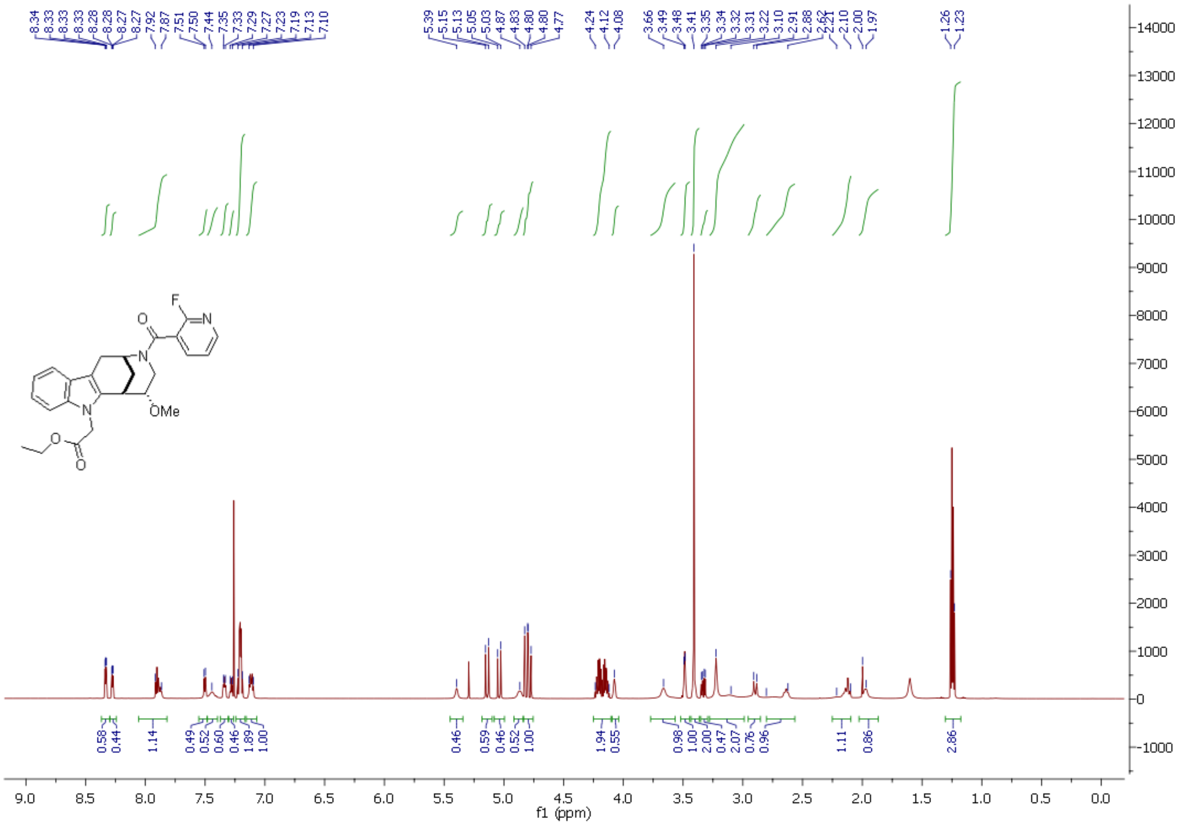

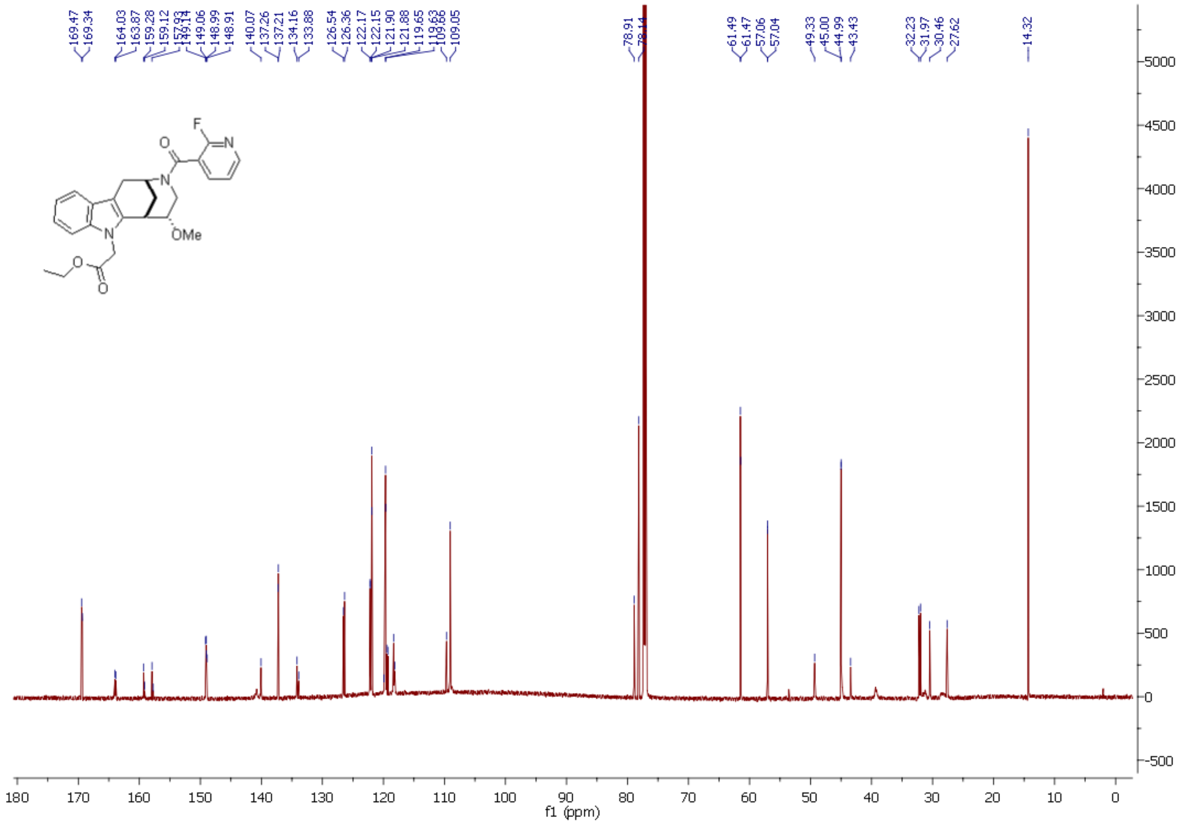

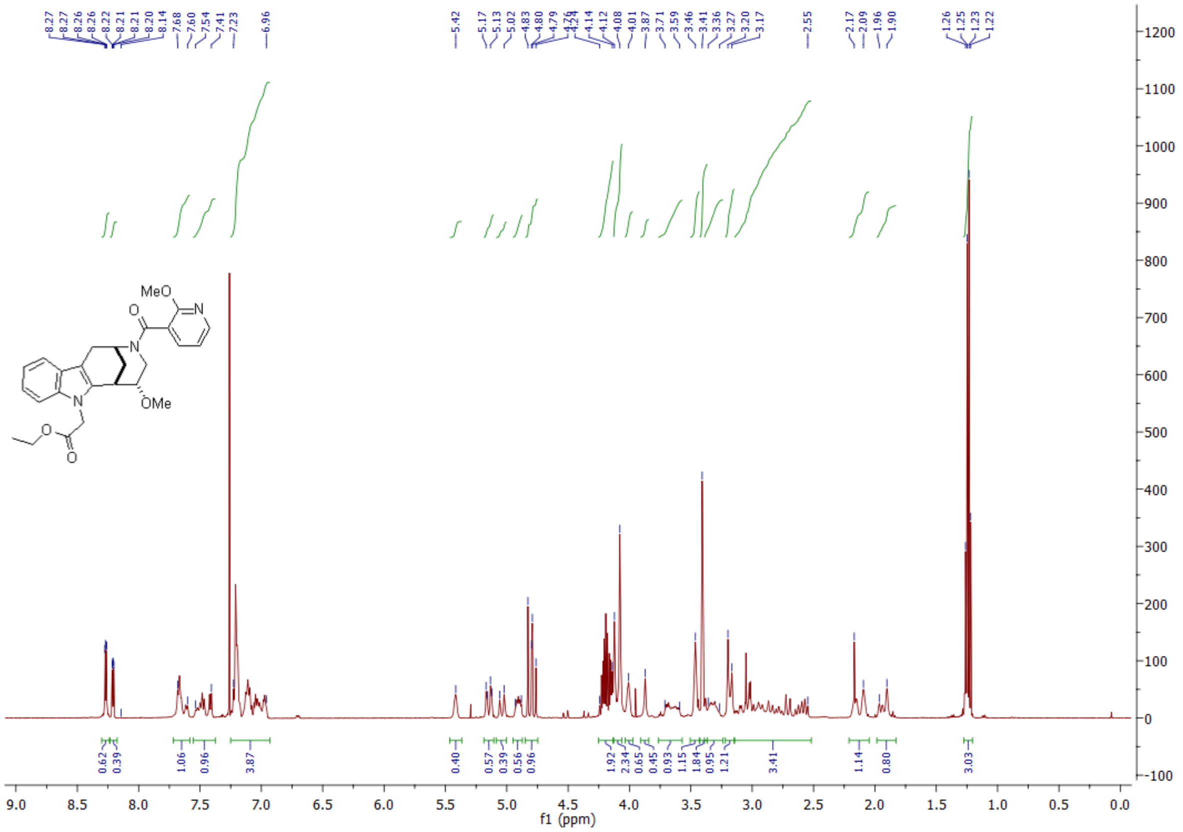

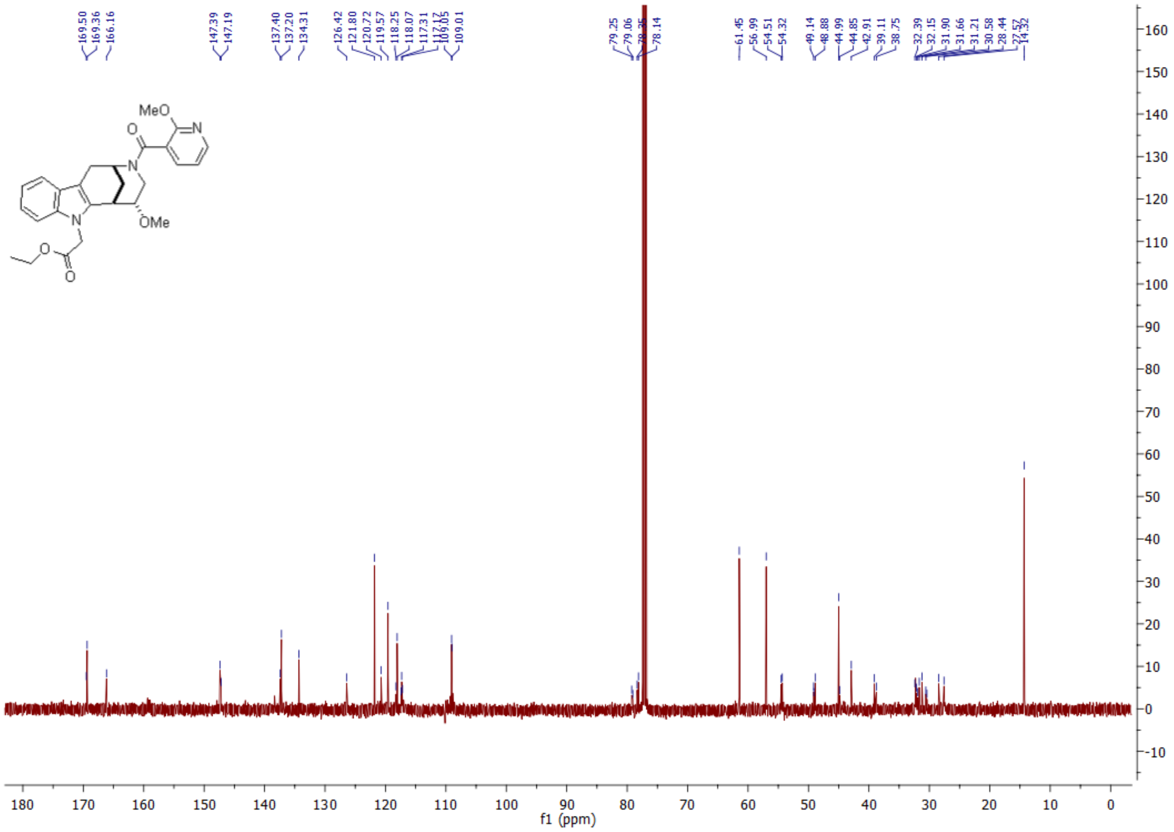

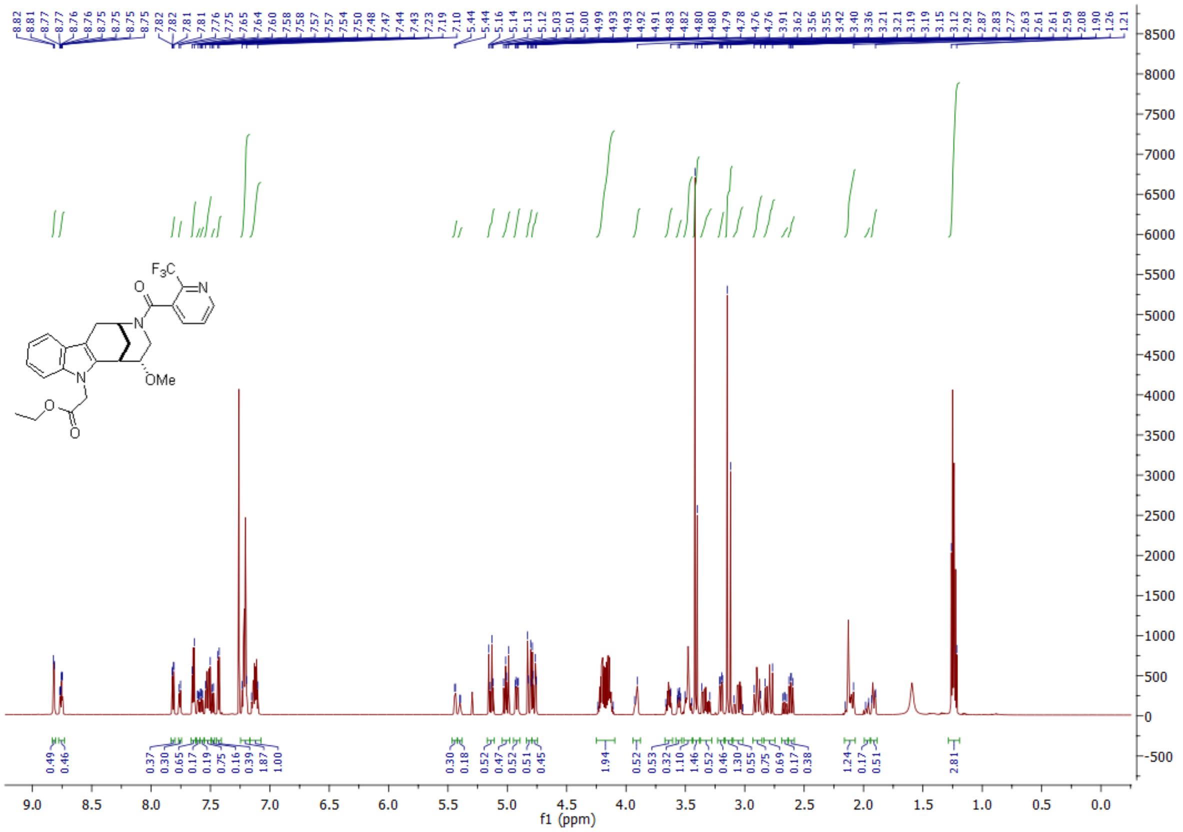

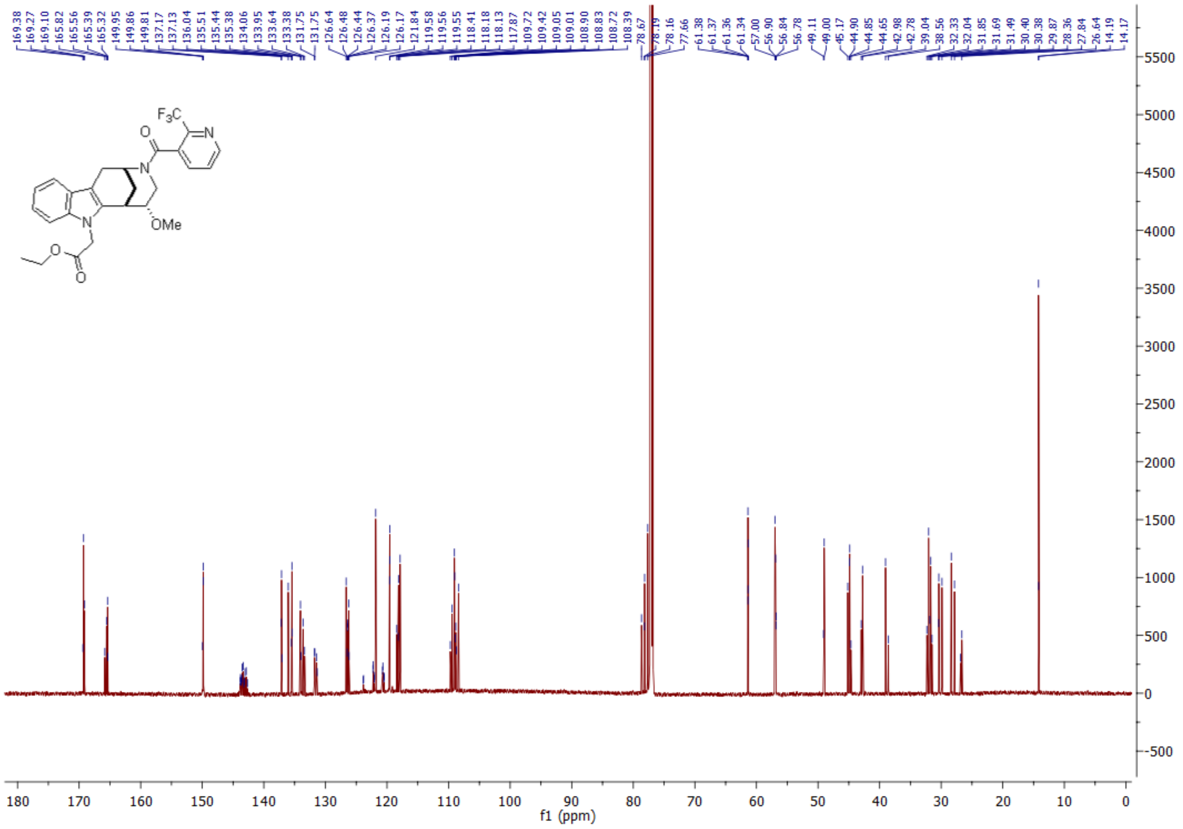

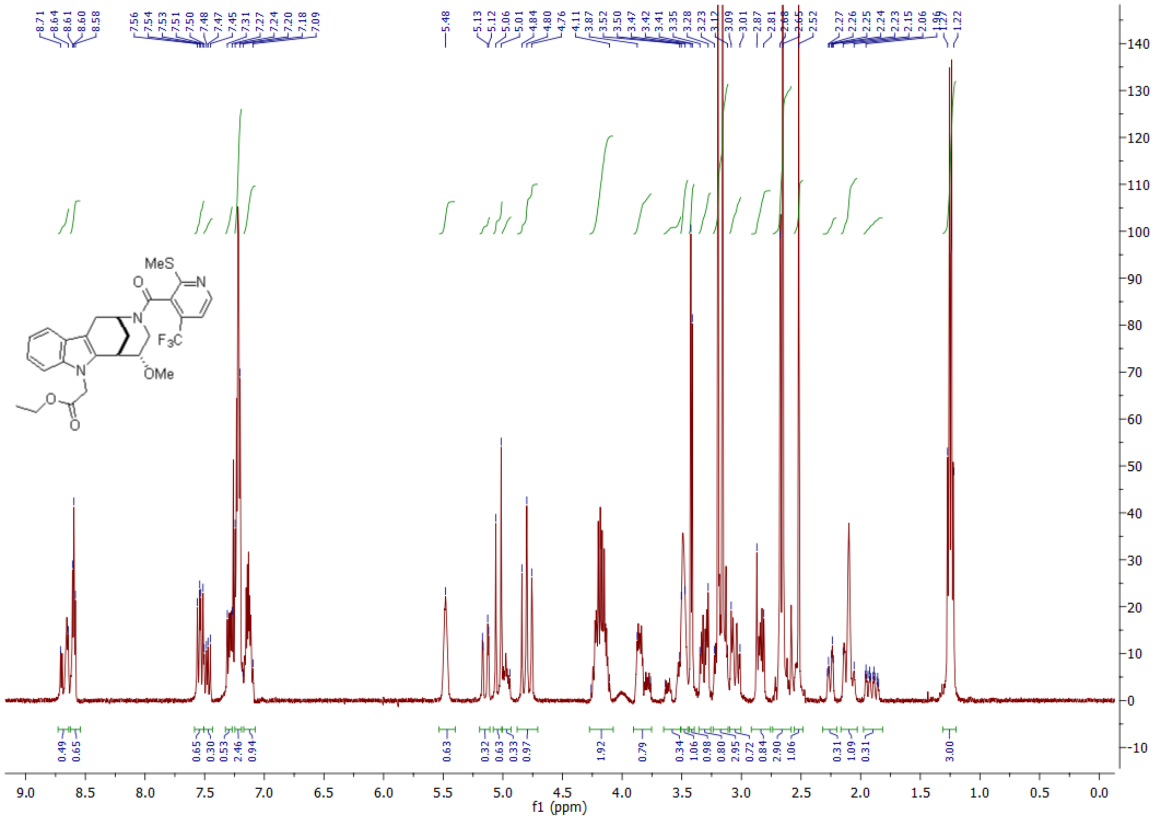

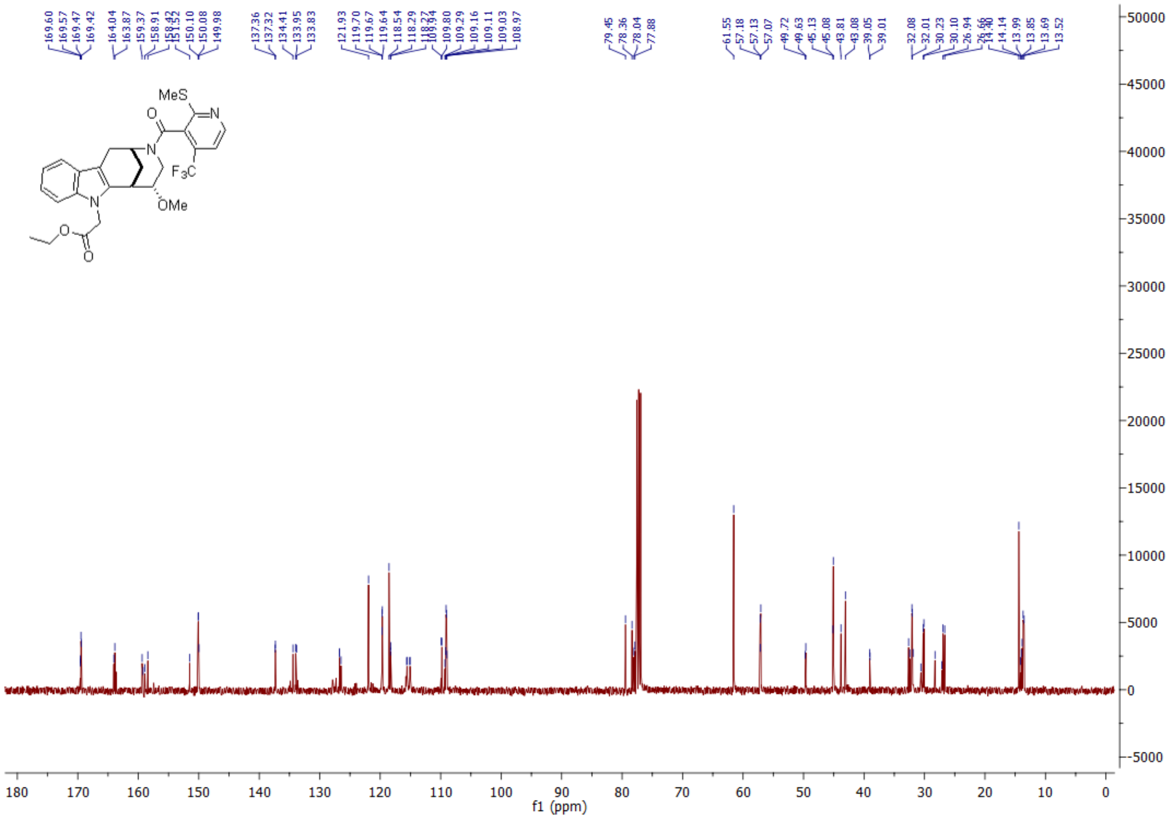

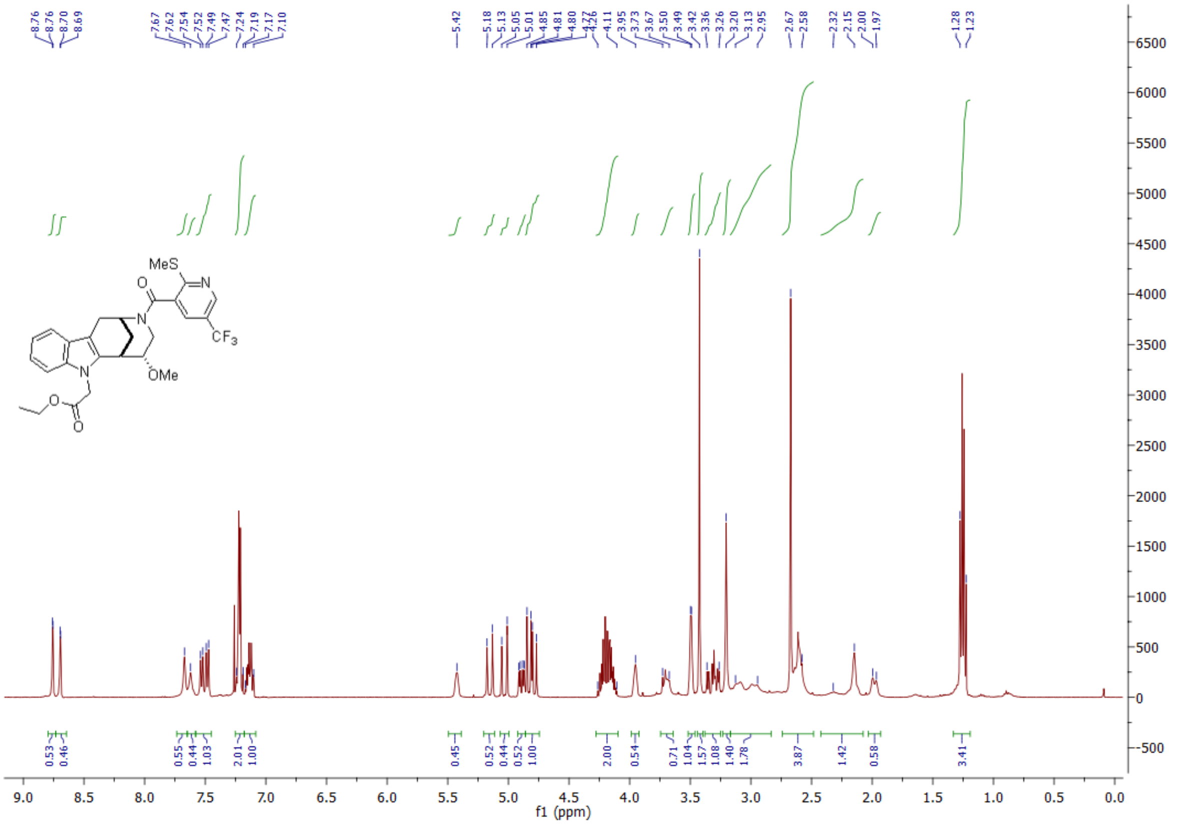

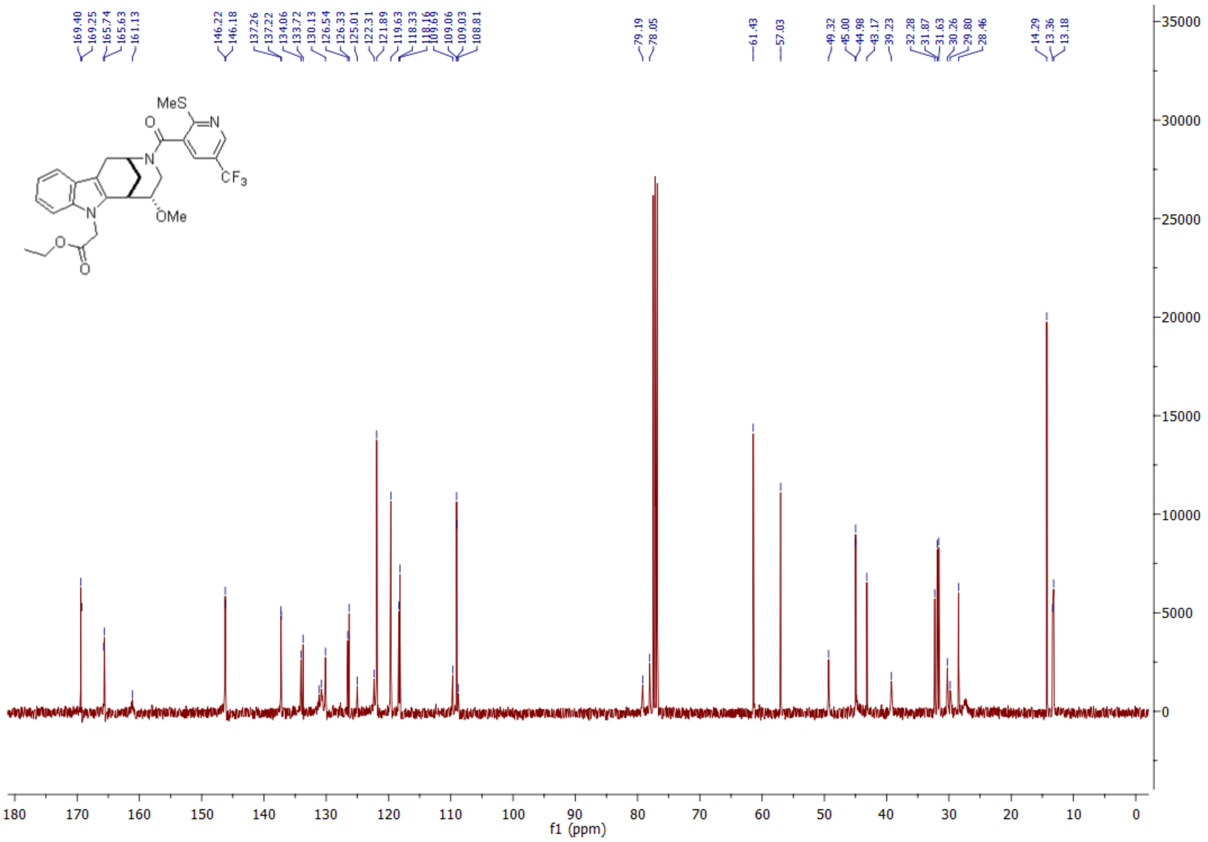

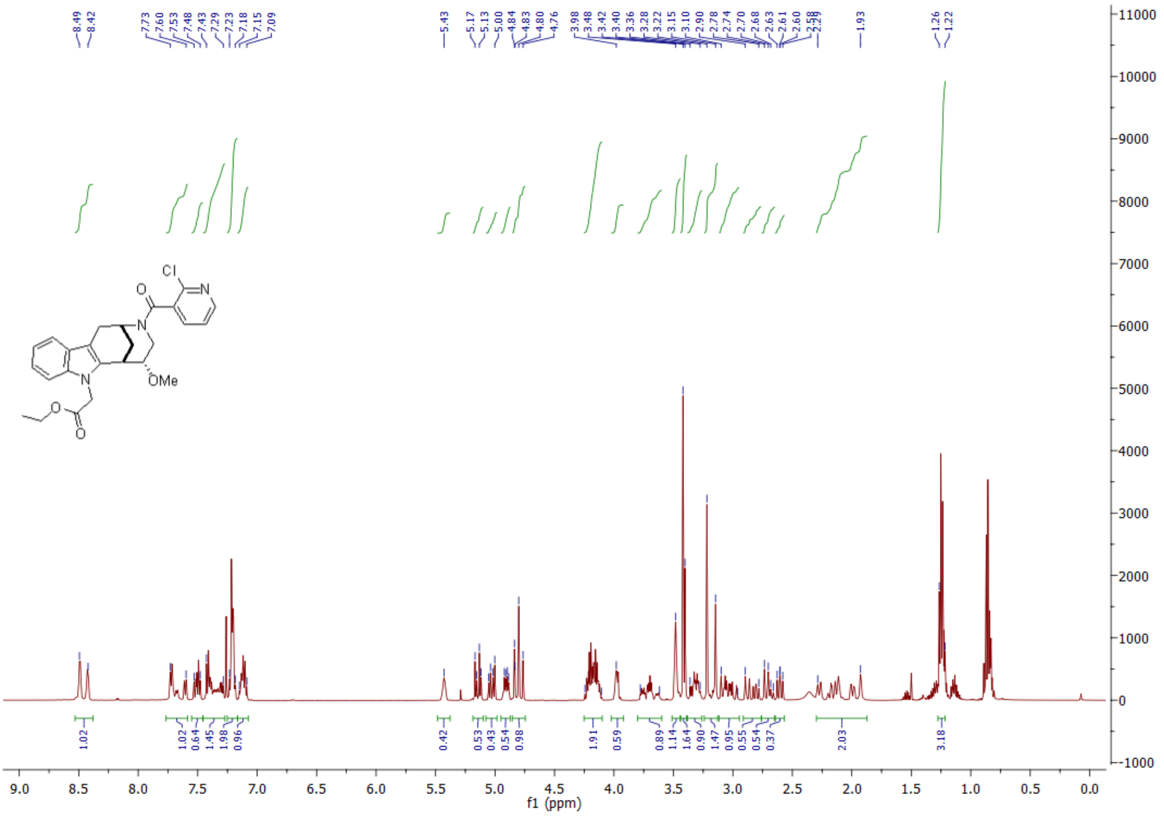

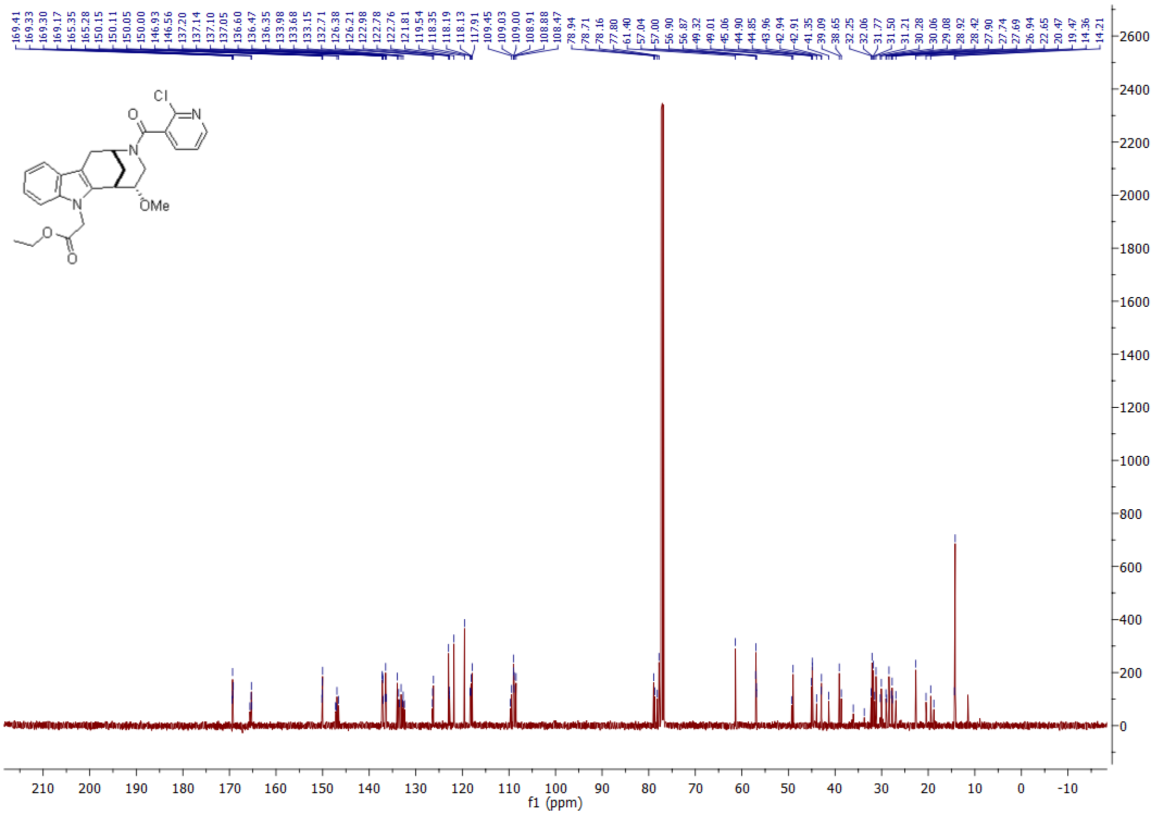

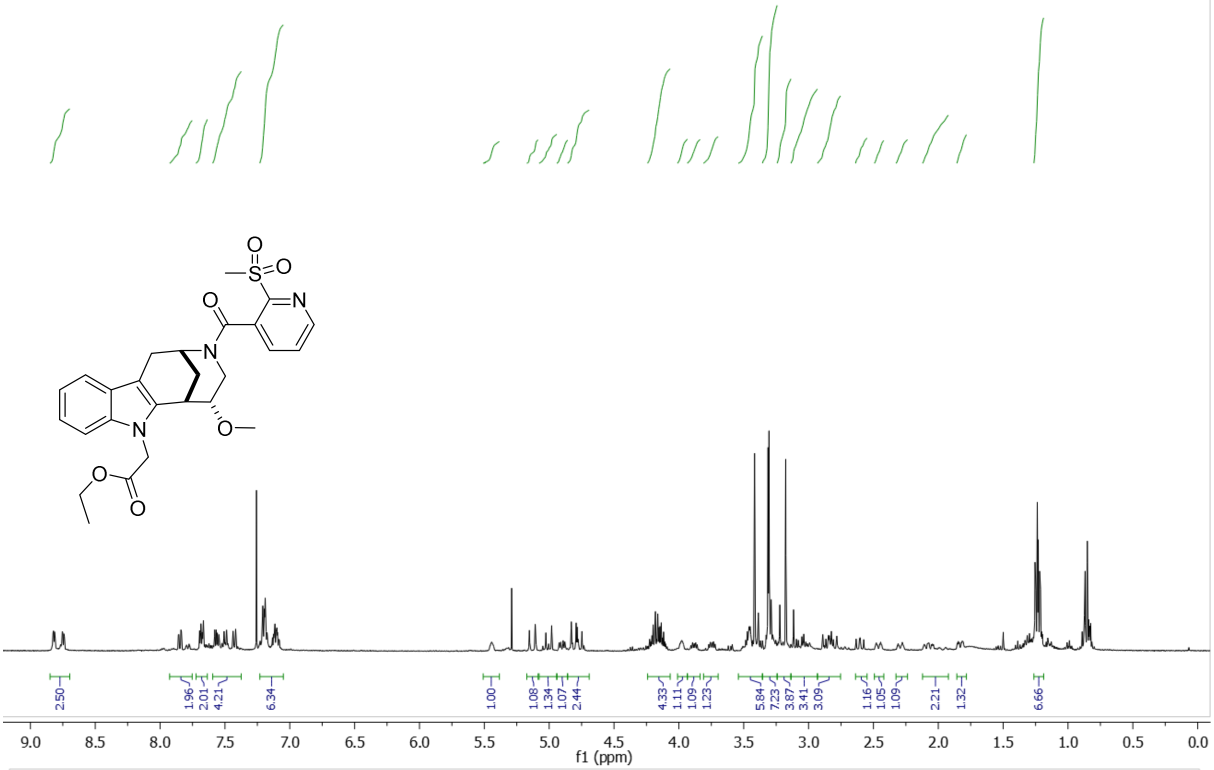

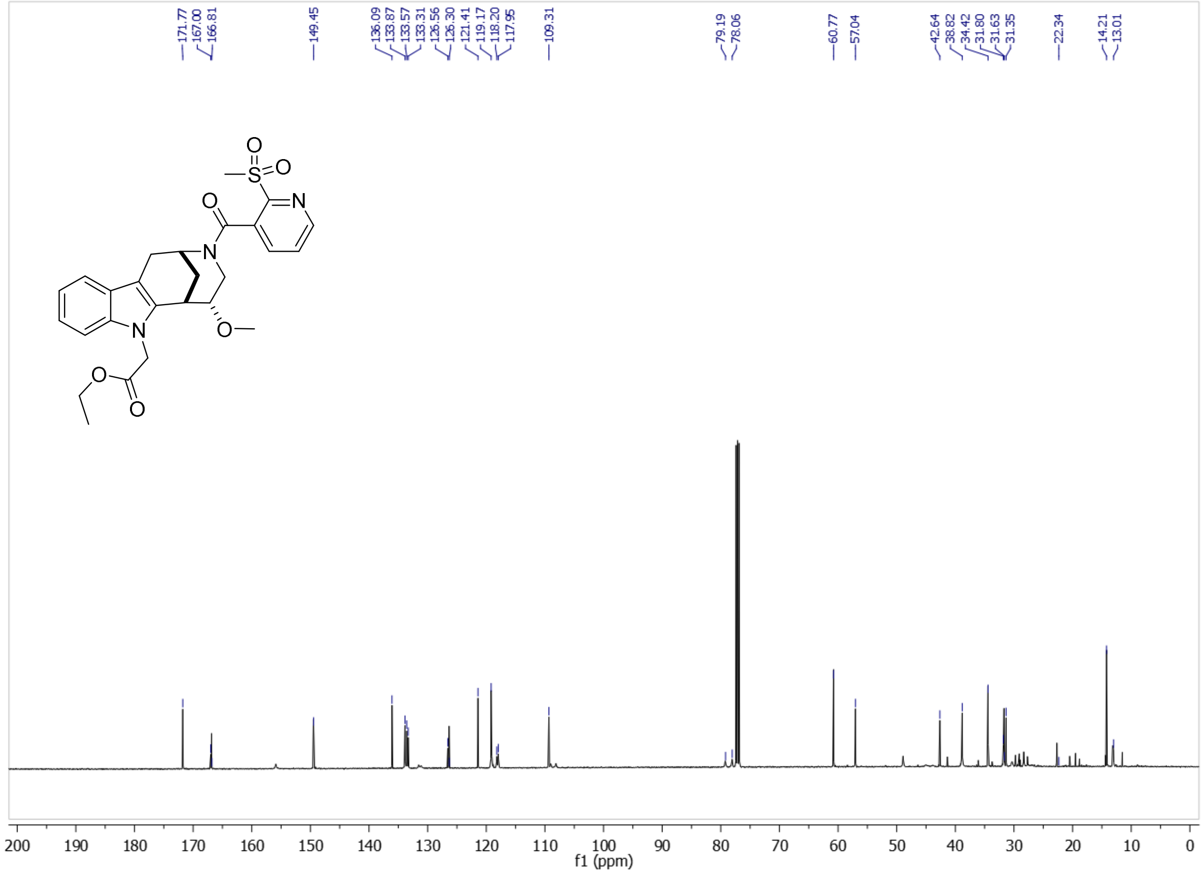

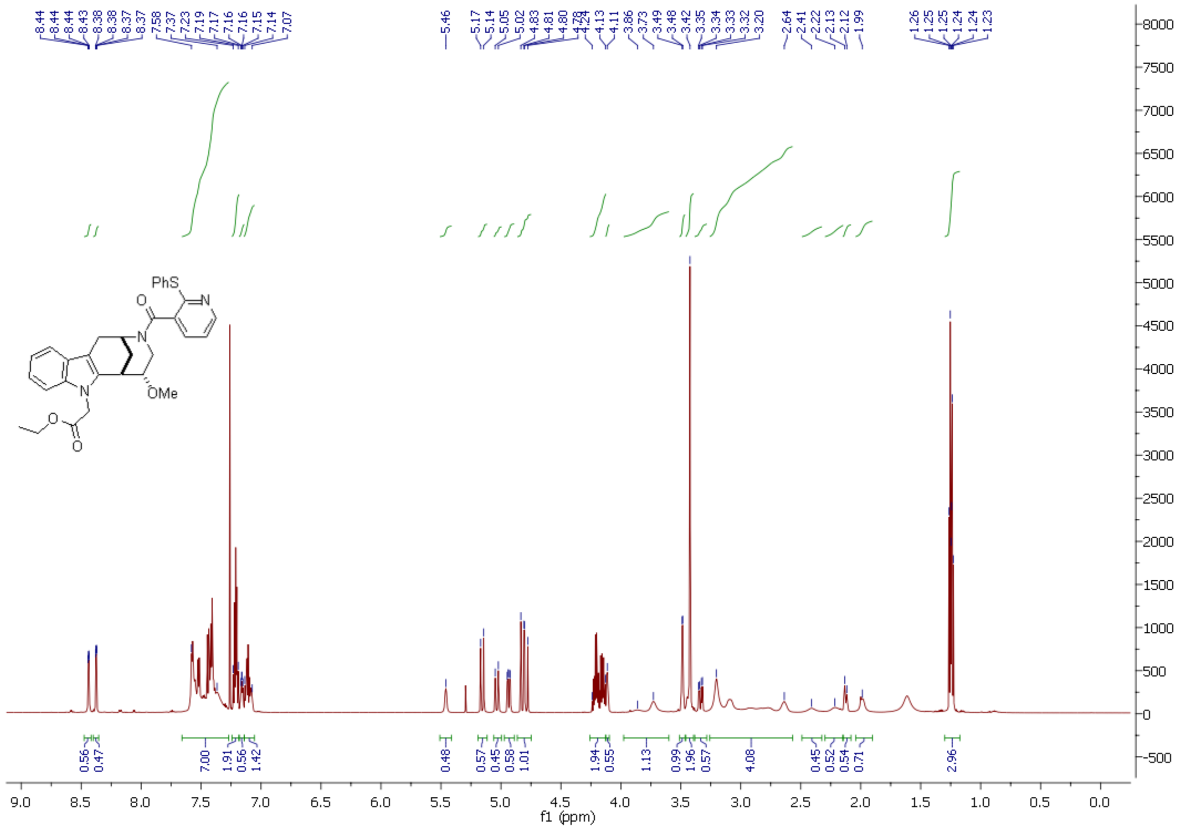

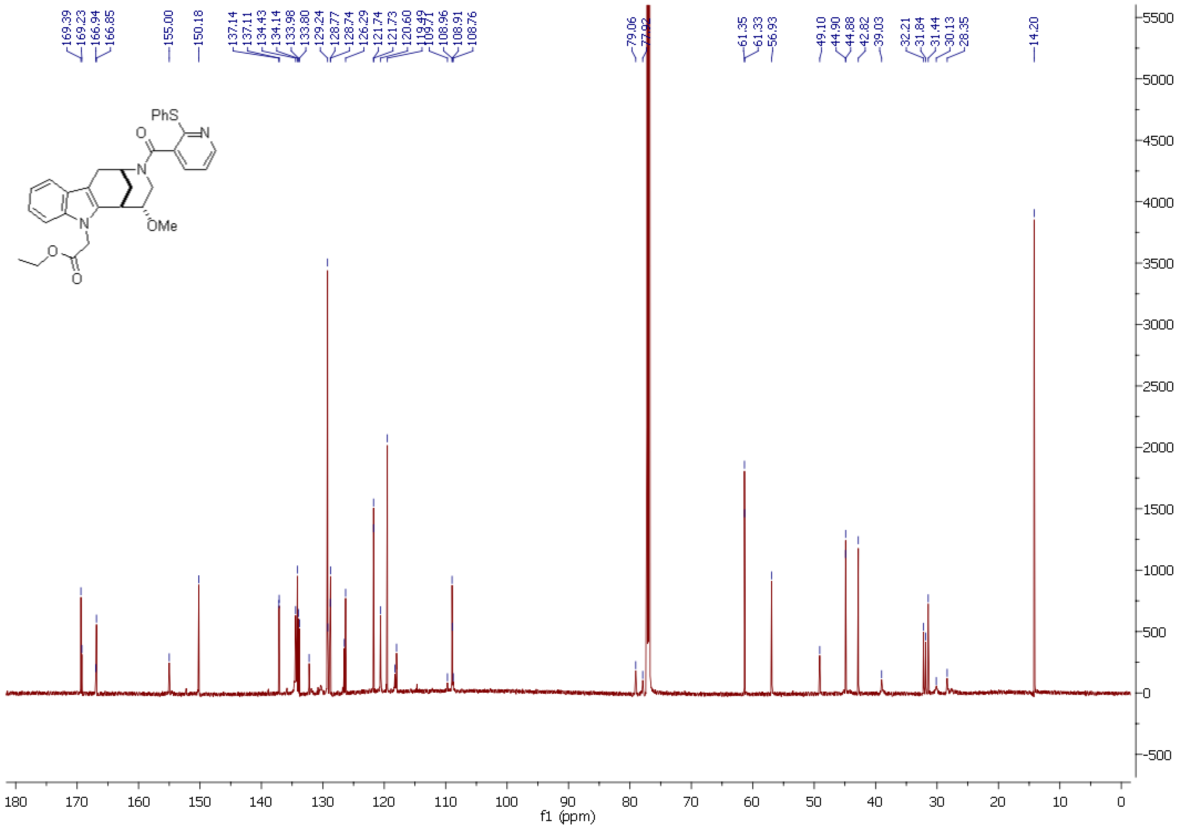

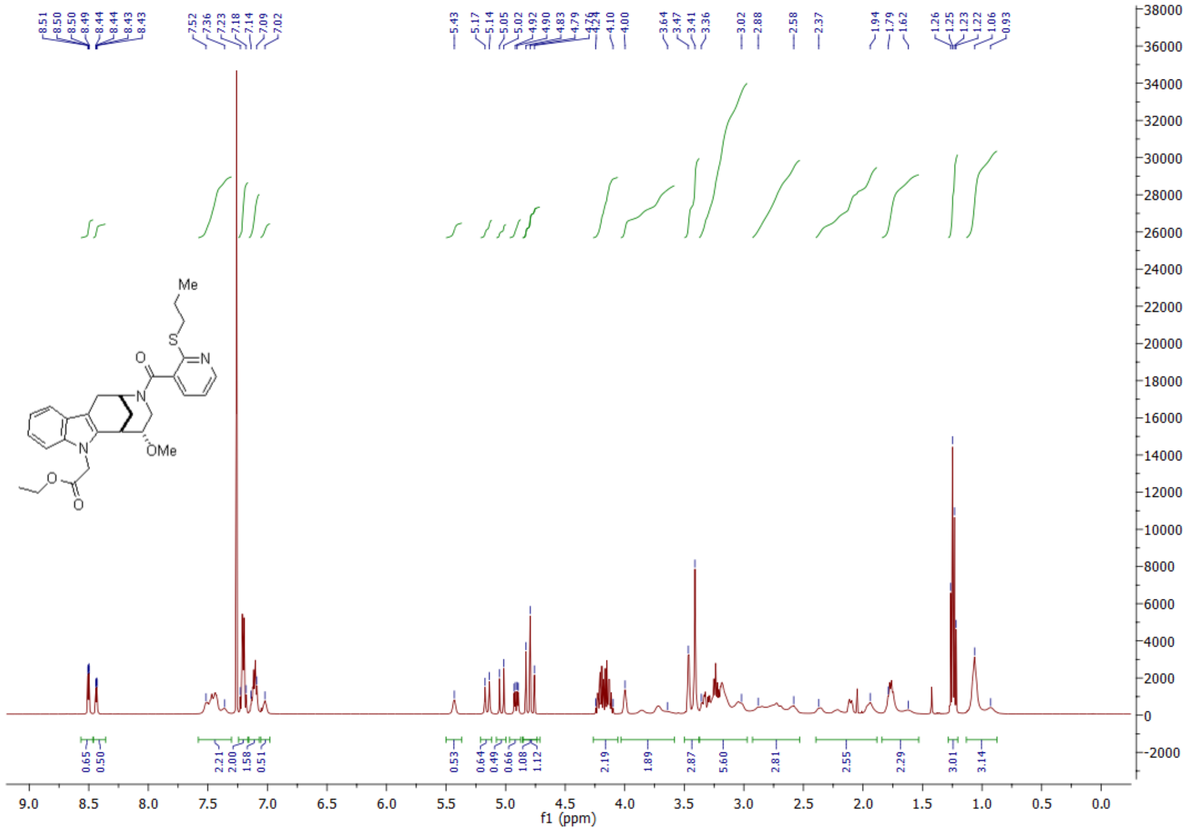

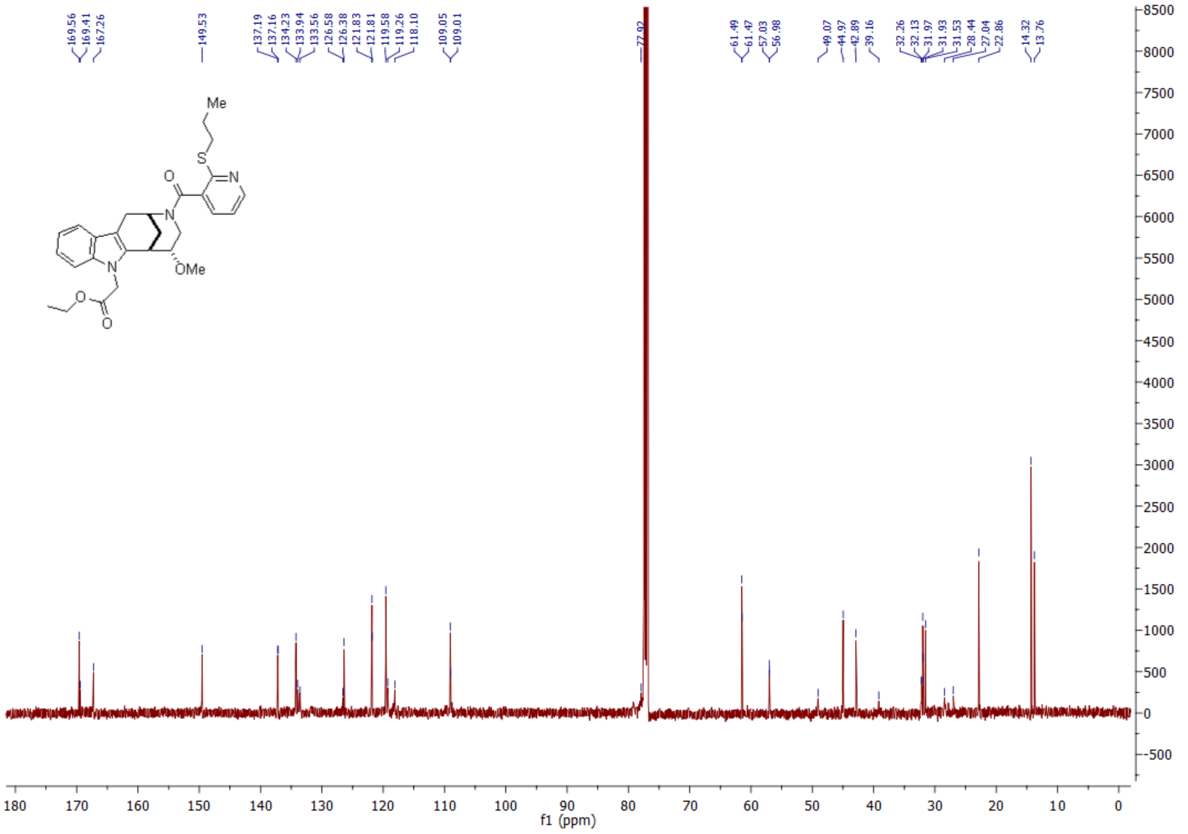

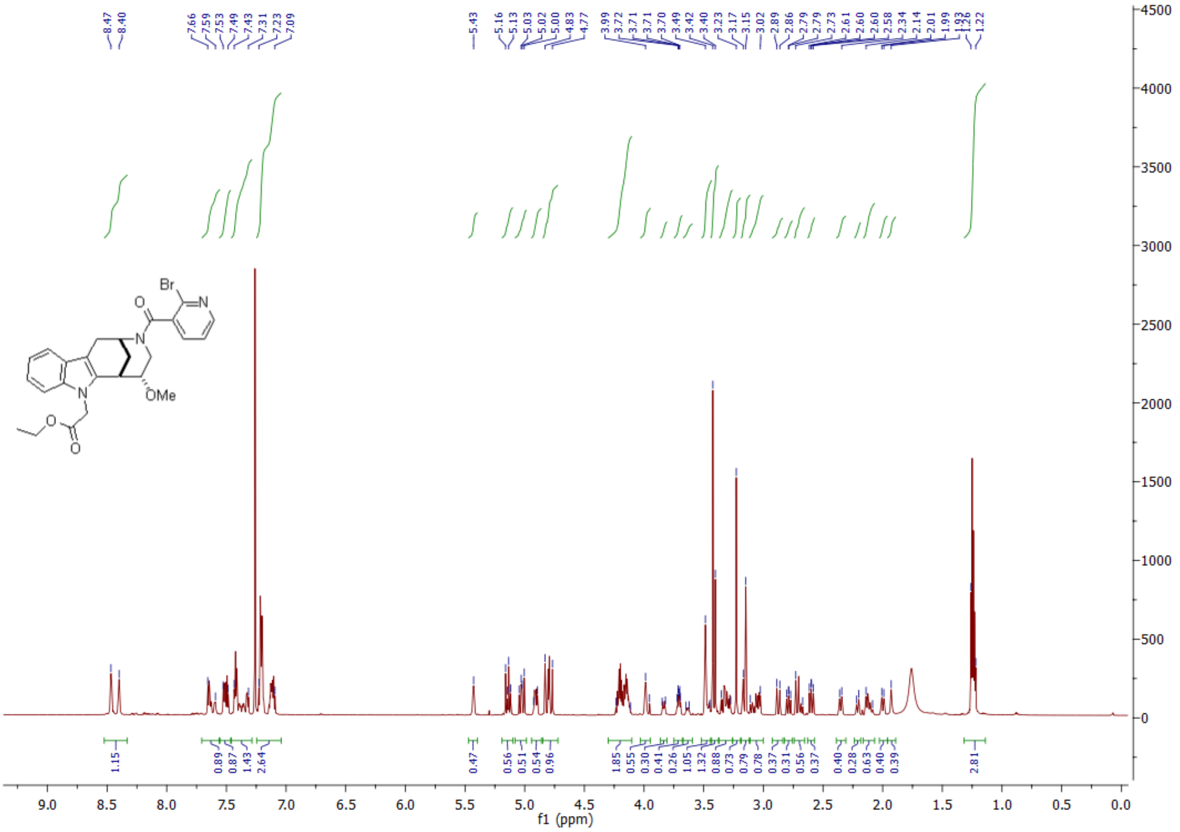
Br
N
O
N
OMe
O
O
f1 (ppm)

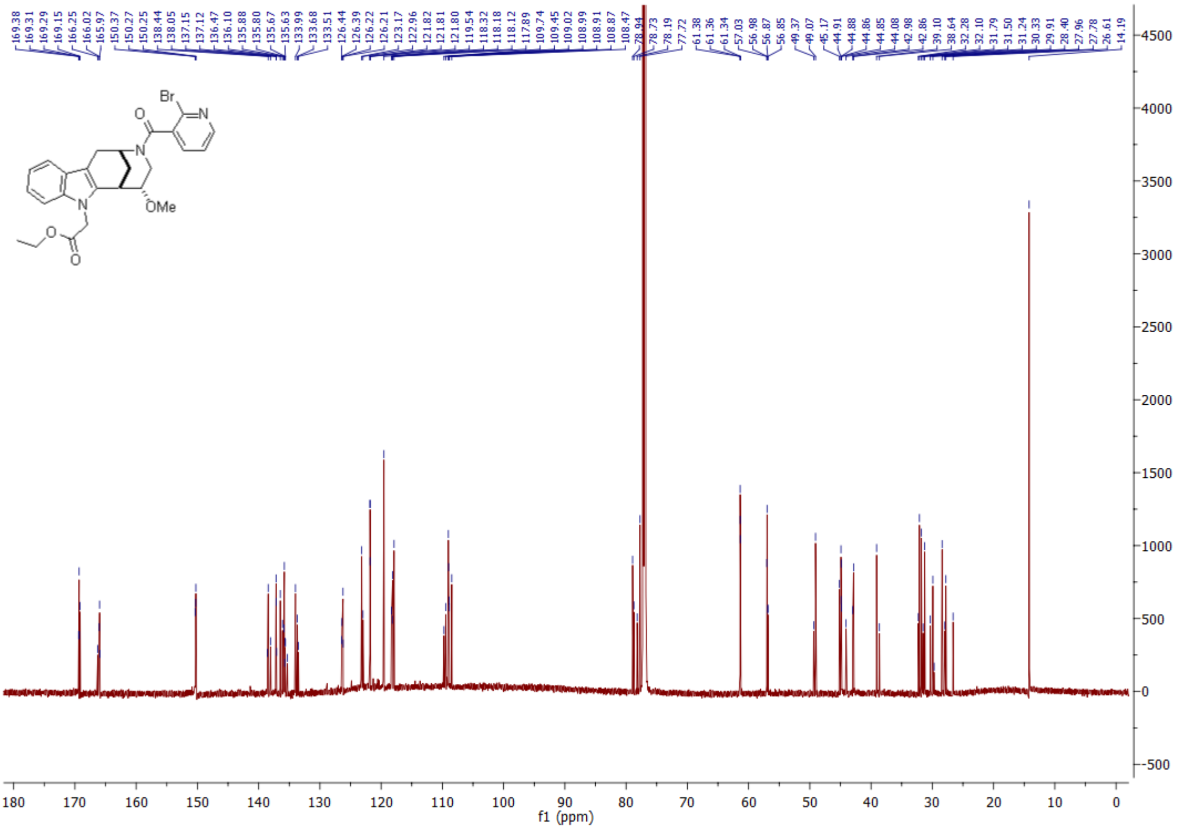
Br
N
O
N
OMe
O
O
f1 (ppm)

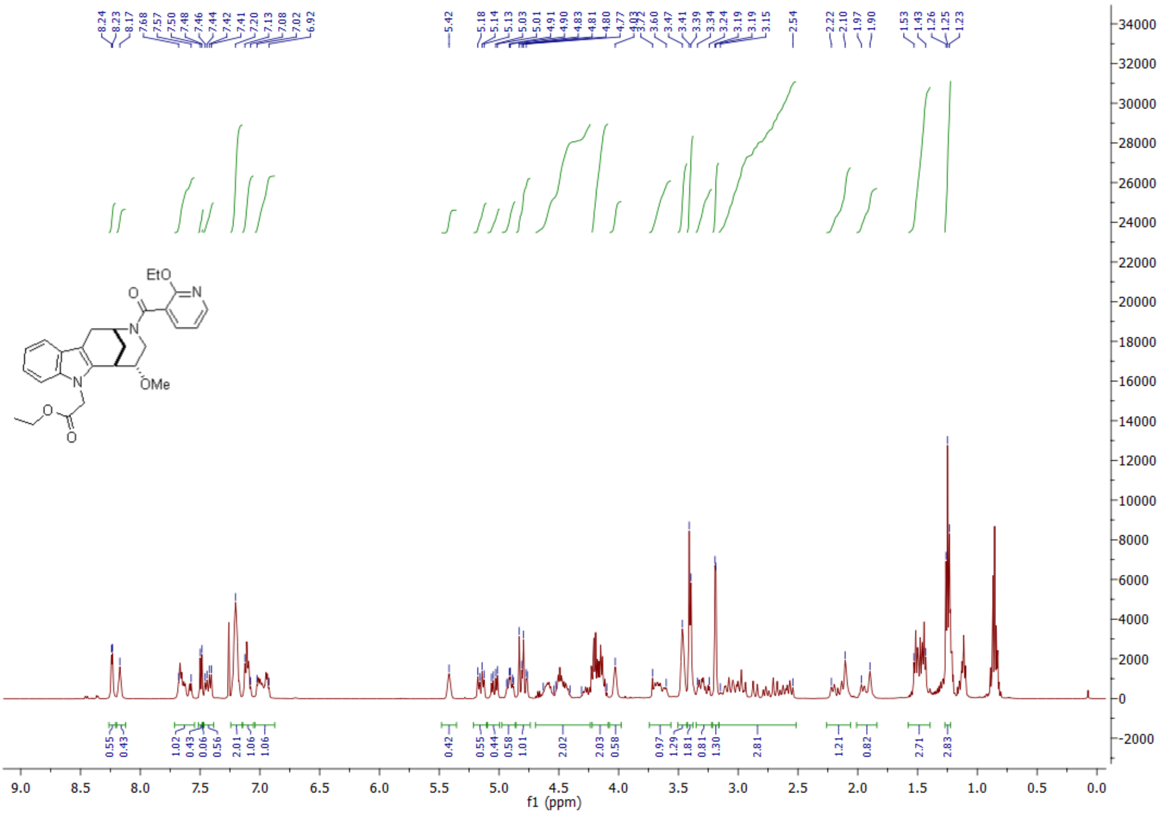

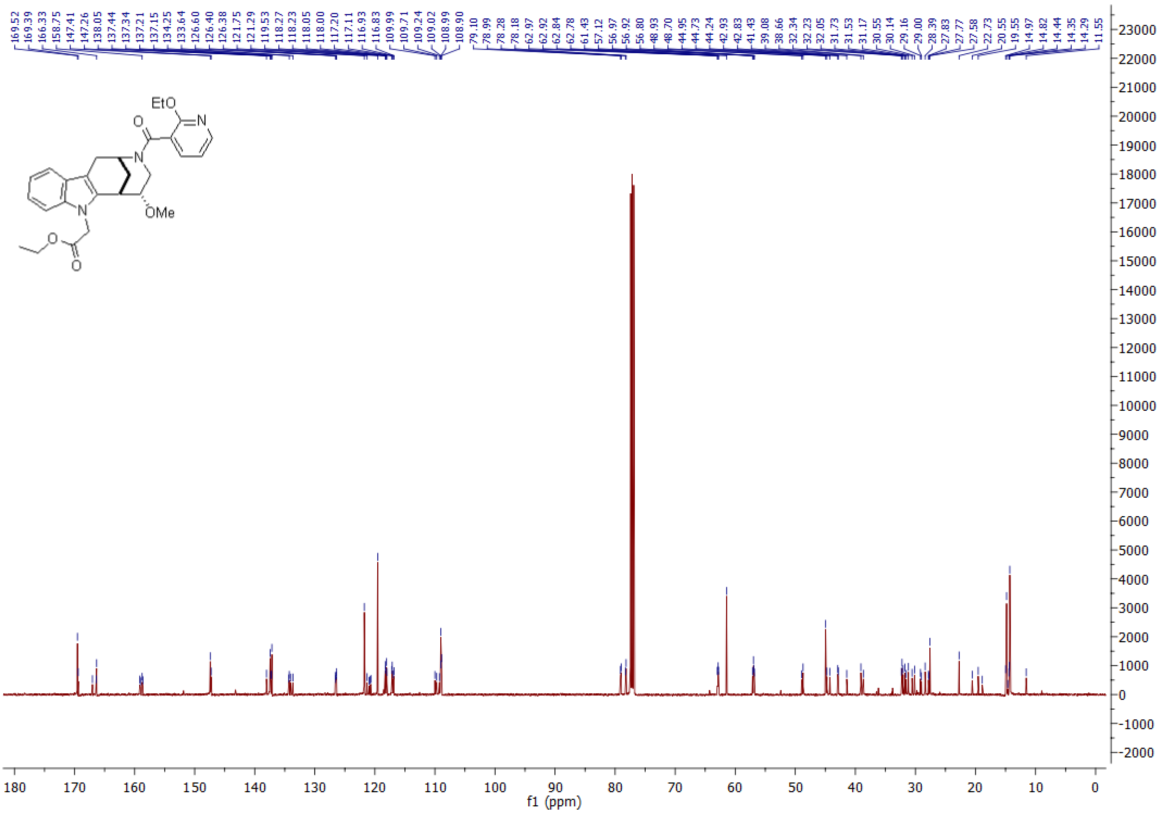

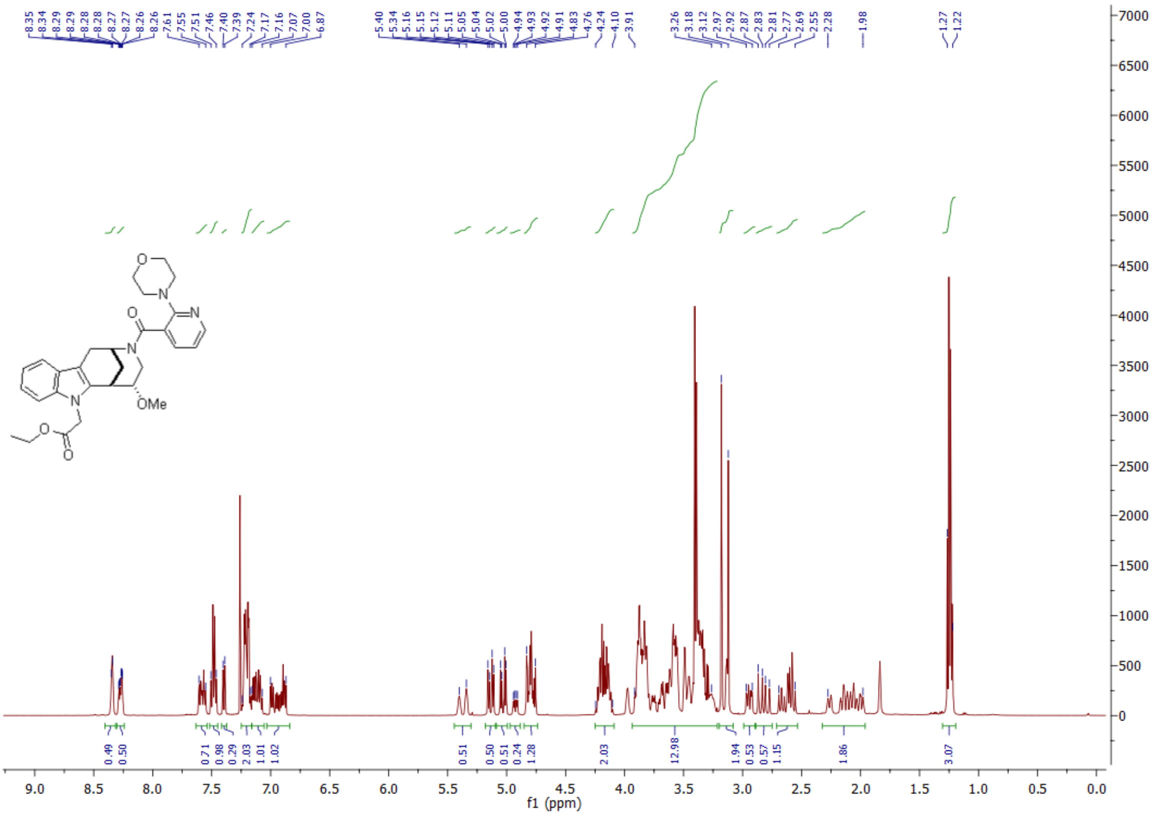

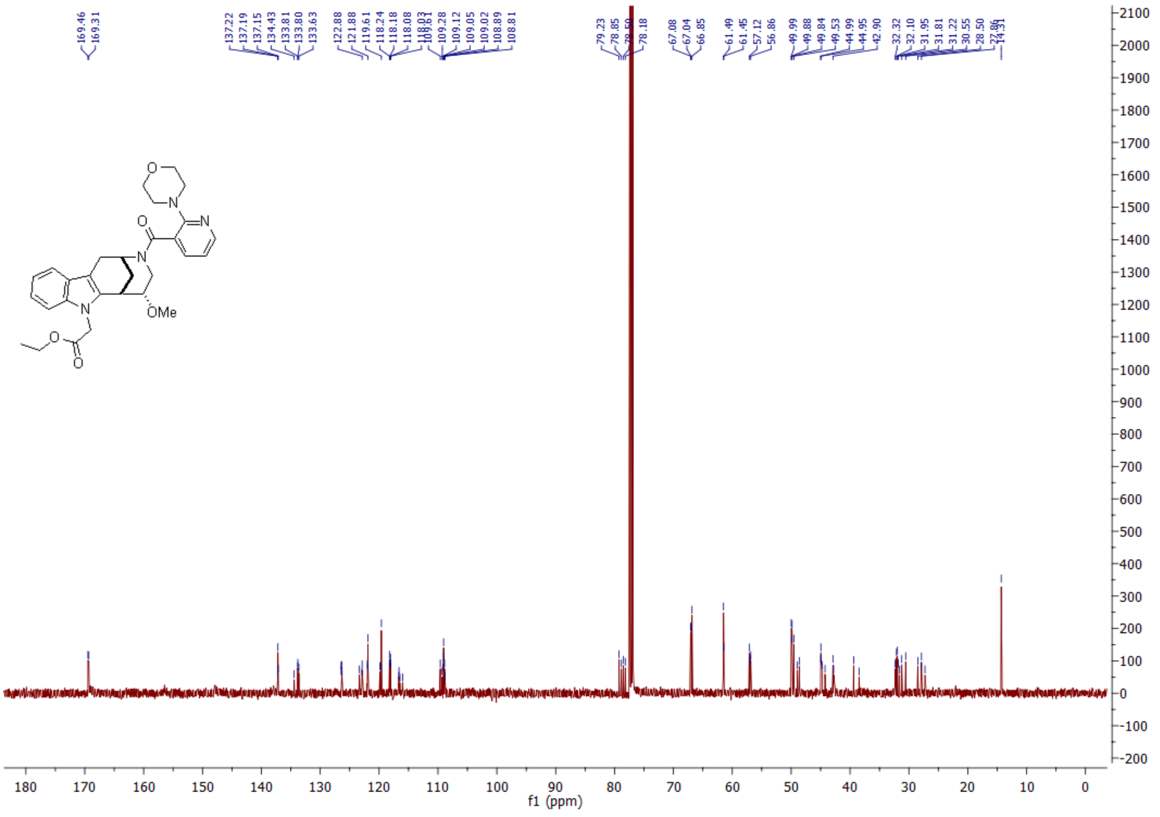

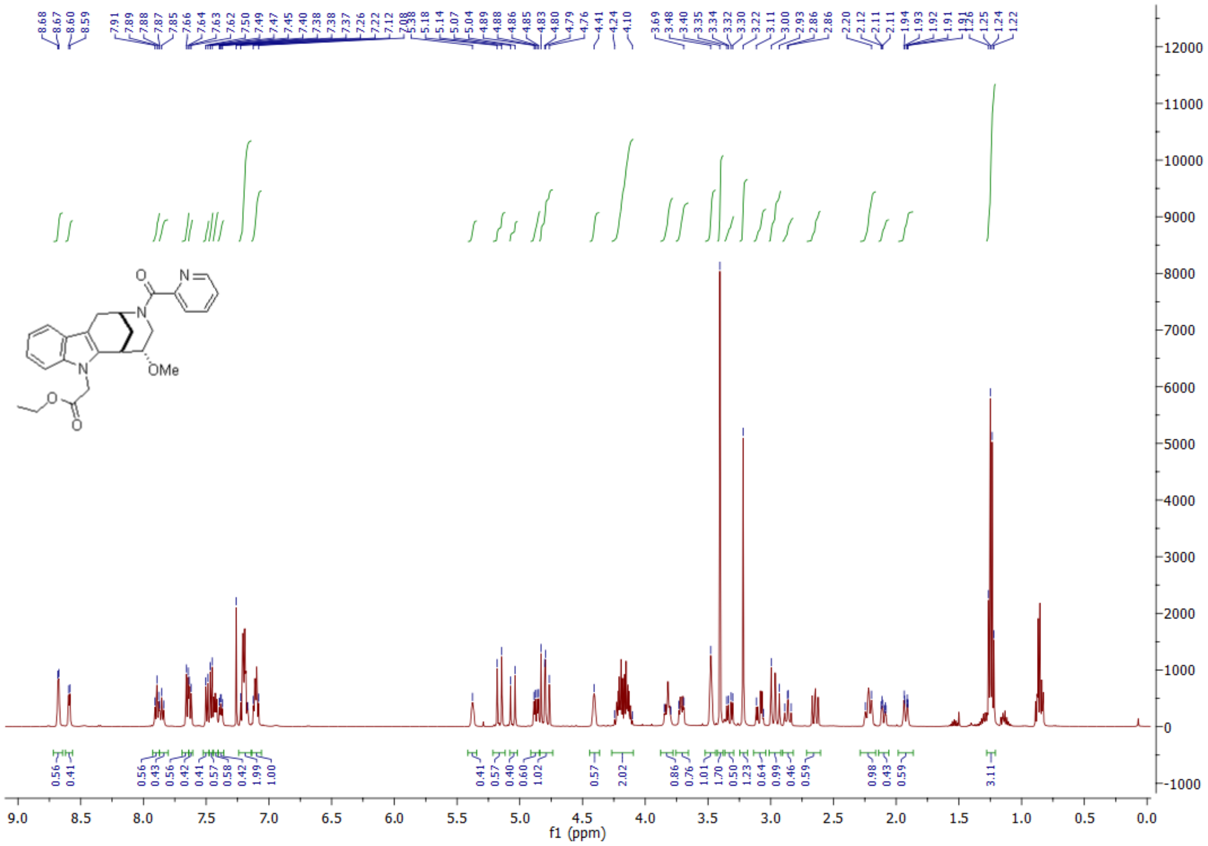
OMe
f1 (ppm)

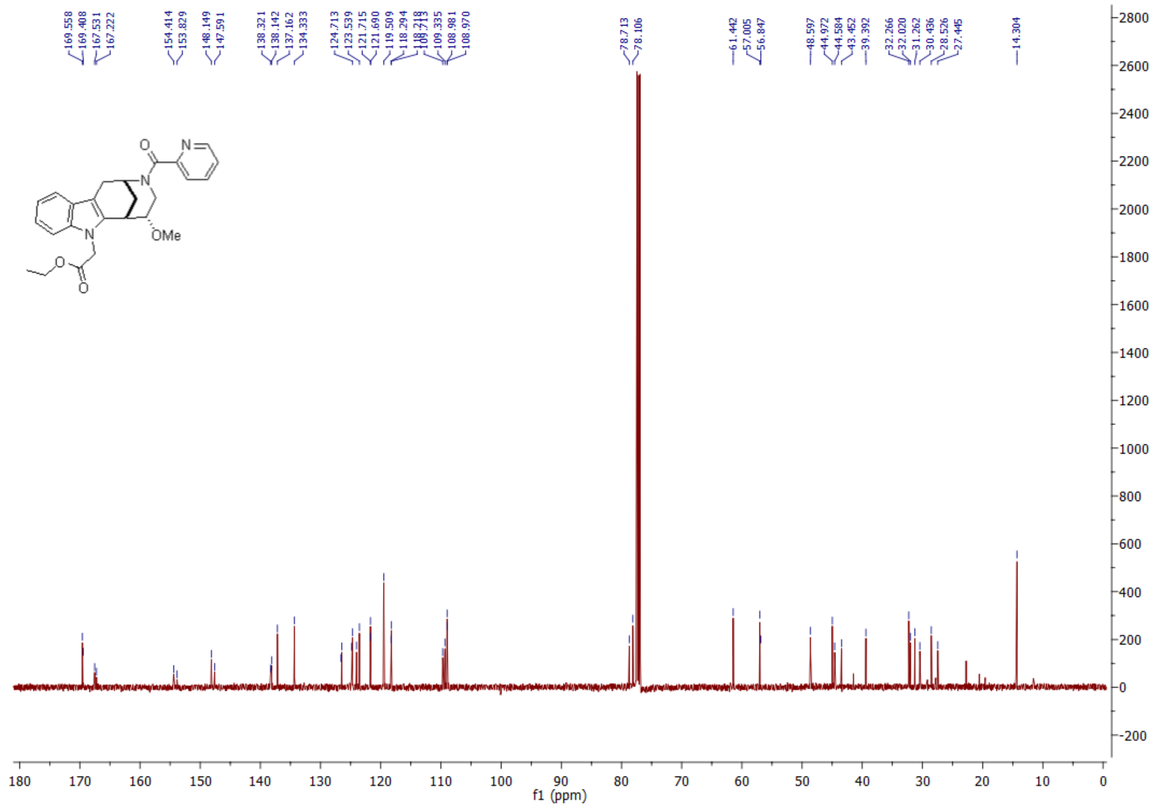
OMe
f1 (ppm)

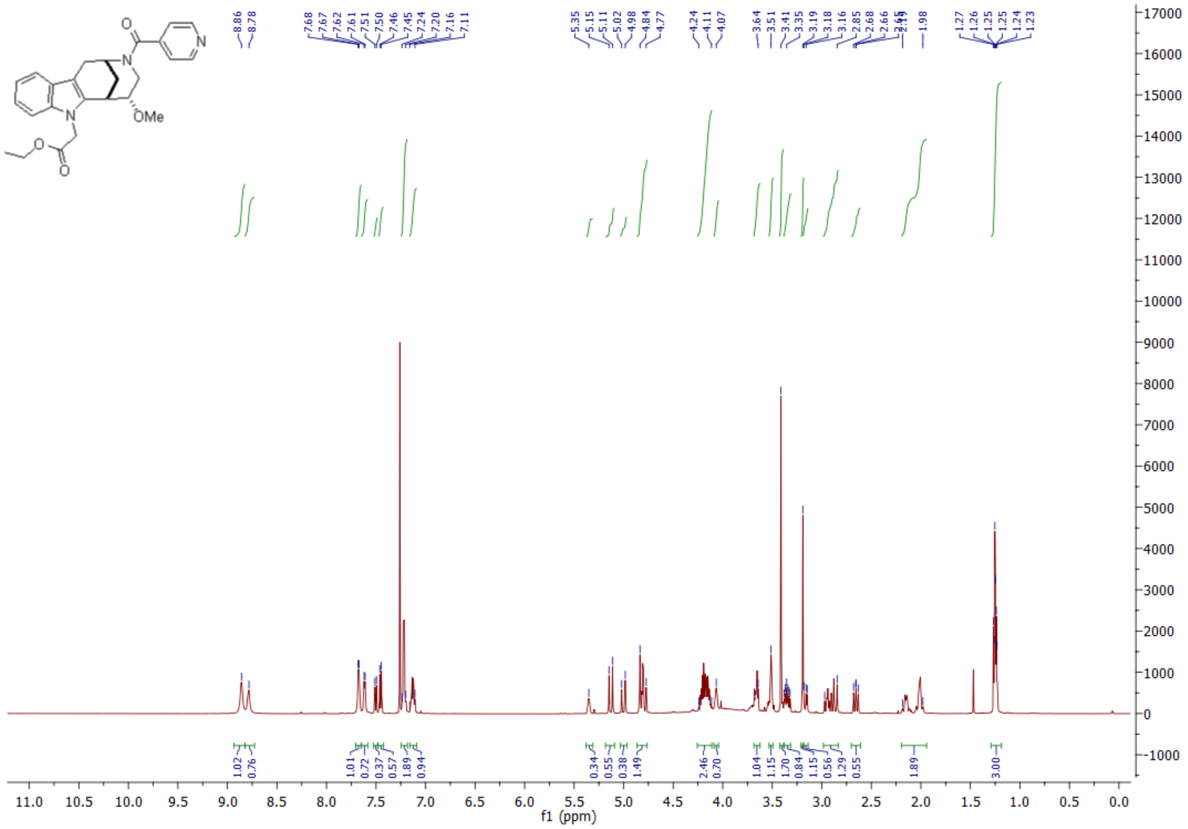

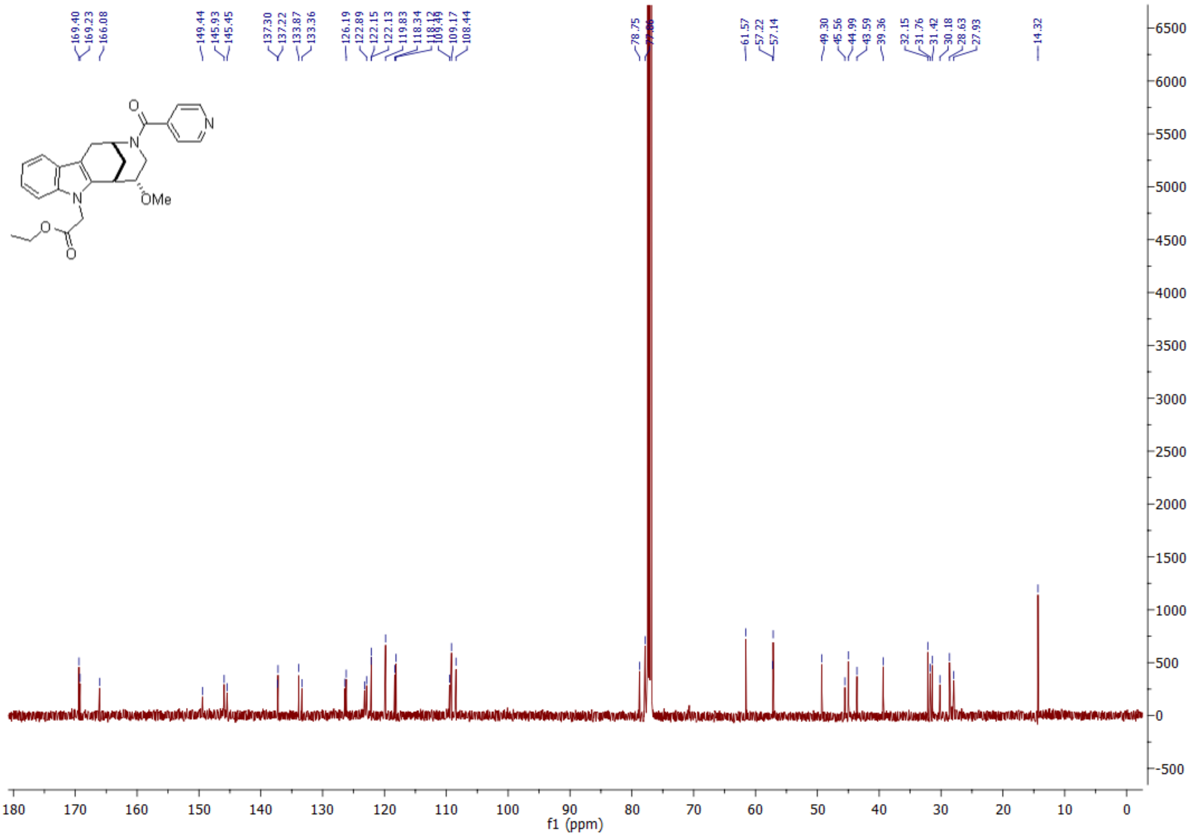

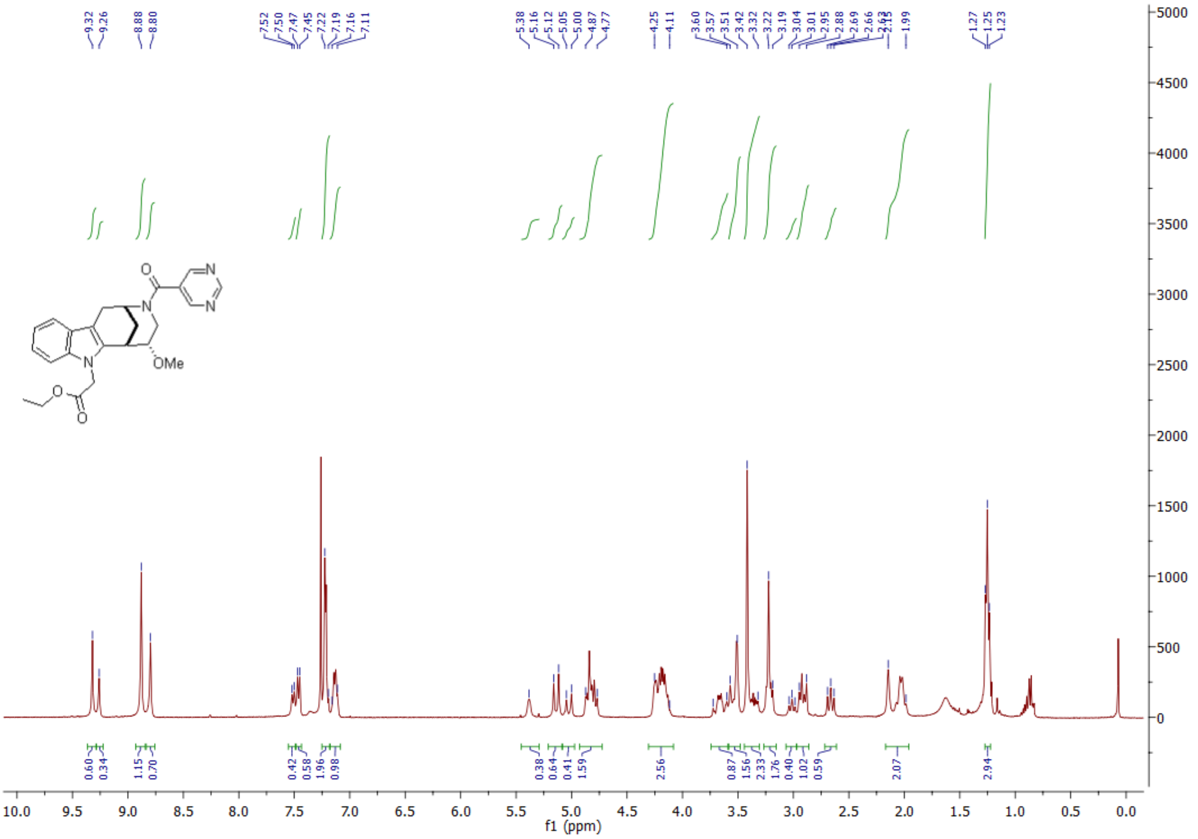

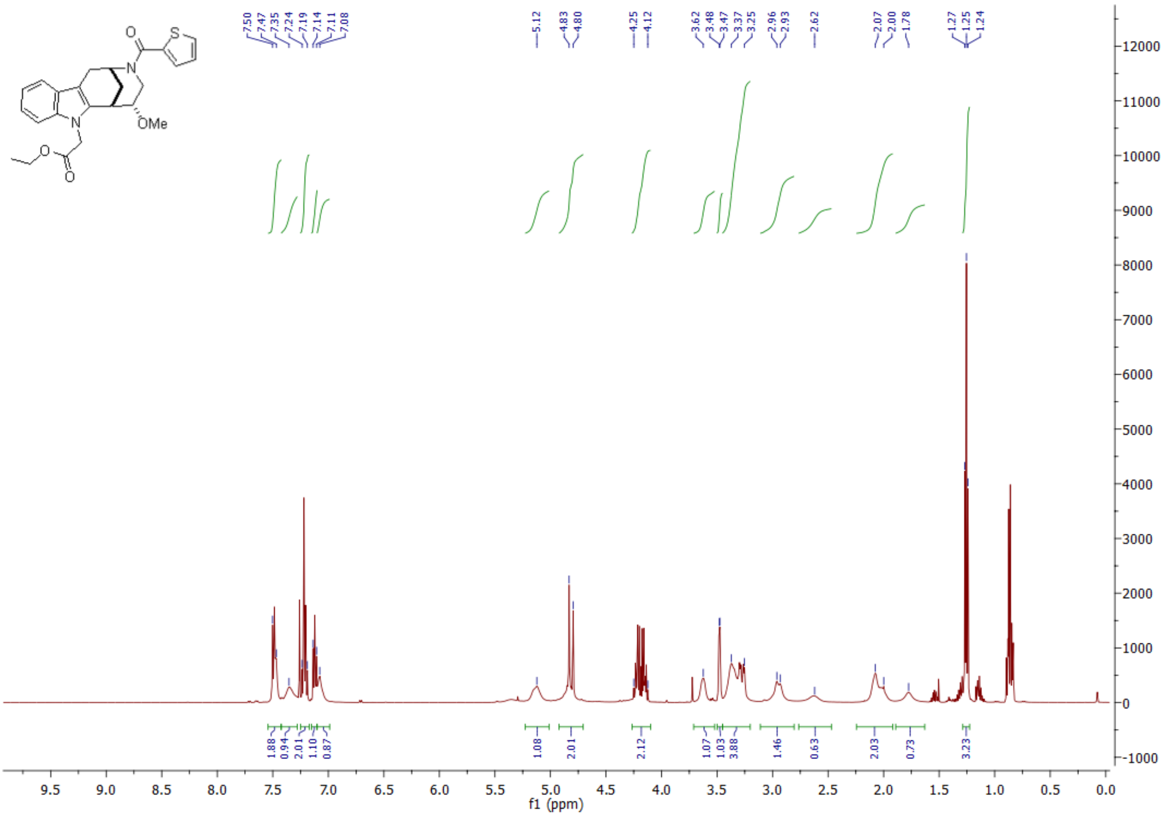
OMe
7.50
7.47
7.35
7.24
7.19
7.14
7.11
7.08
5.12
4.83
4.80
4.25
4.12
3.62
3.48
3.47
3.37
3.25
2.96
2.93
2.62
2.07
2.00
1.78
1.27
1.25
1.24
1.88
0.94
2.01
1.10
0.87
1.08
2.01
2.12
1.07
1.03
3.88
1.46
0.63
2.03
0.73
3.23
f1 (ppm)

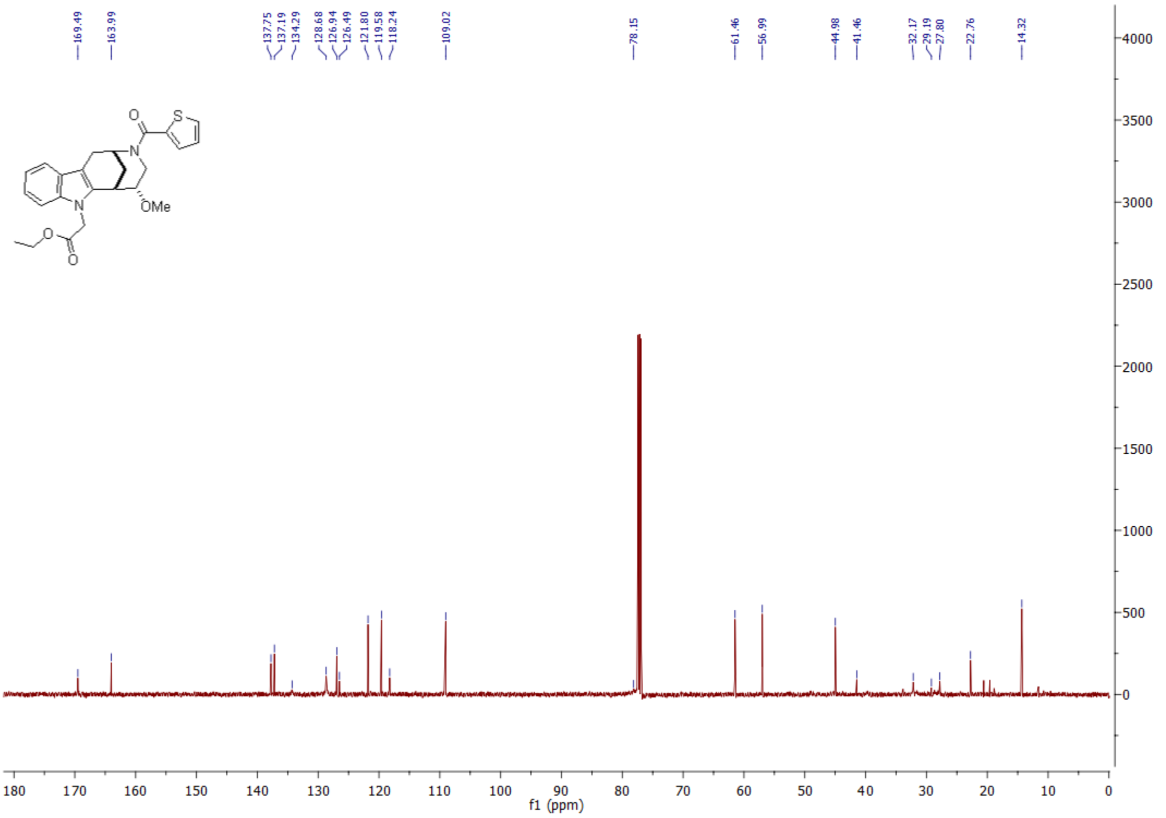
OMe
169.49
163.99
137.75
137.19
134.29
128.68
126.94
126.49
121.80
119.58
118.24
109.02
78.15
61.46
56.99
44.98
41.46
32.17
29.19
27.80
22.76
14.32
f1 (ppm)

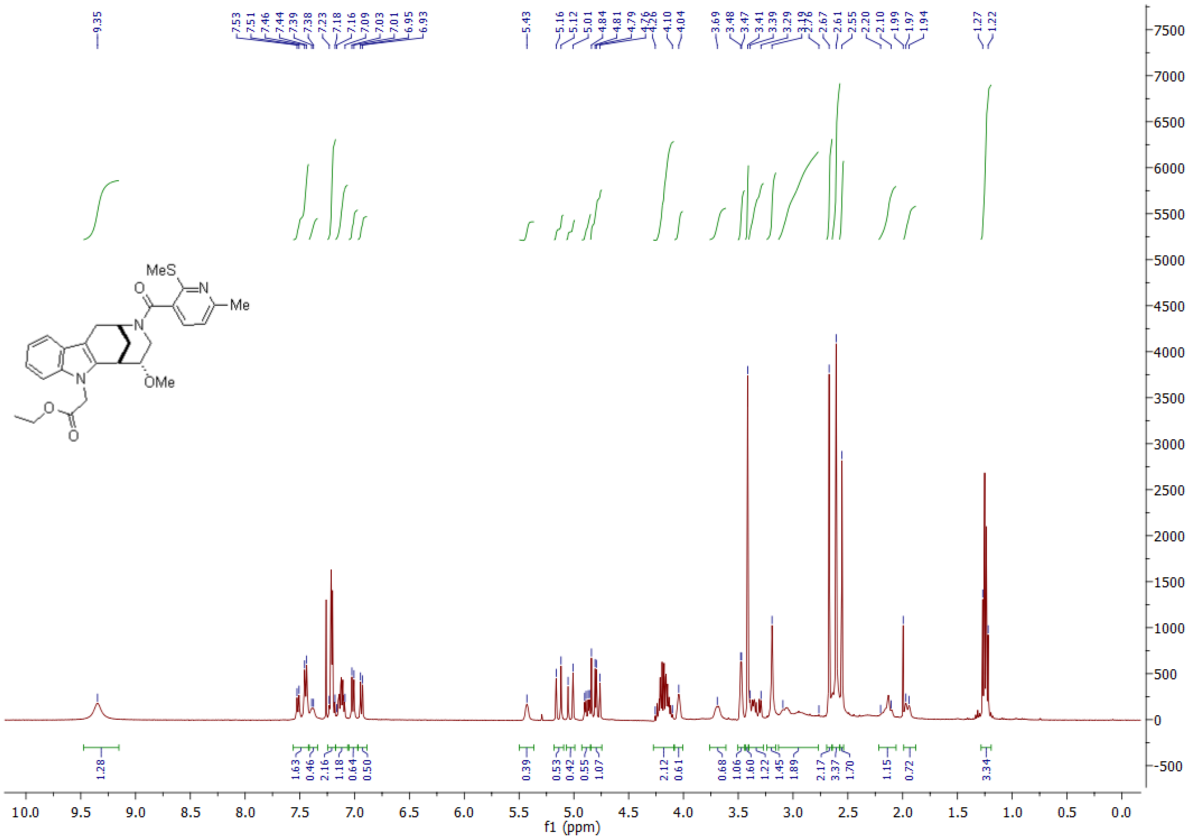

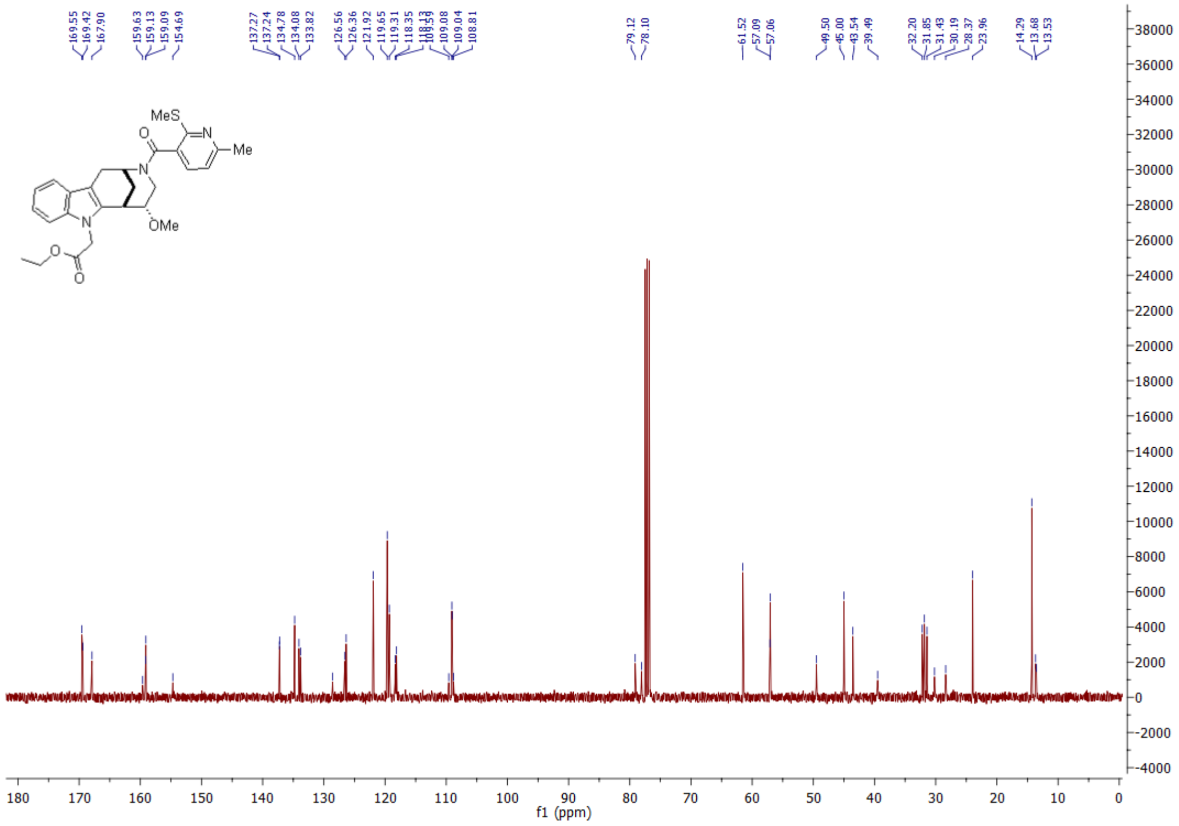

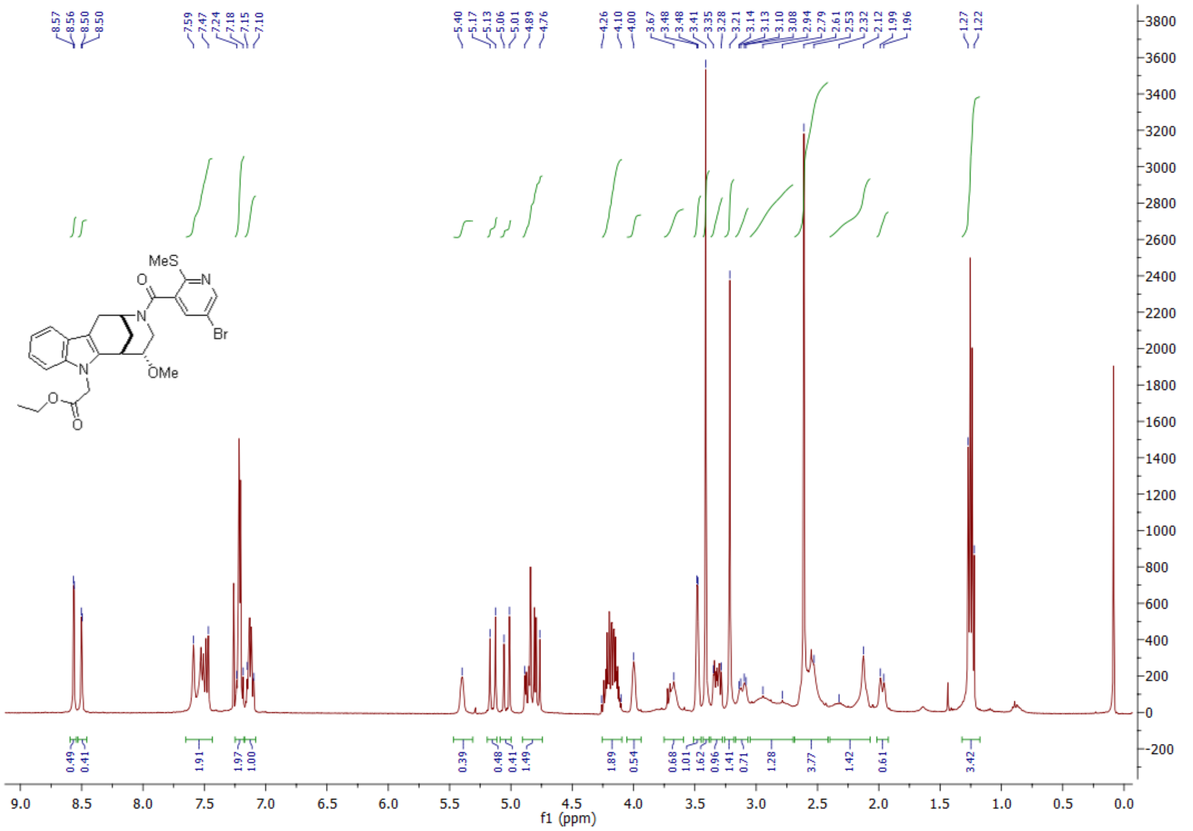

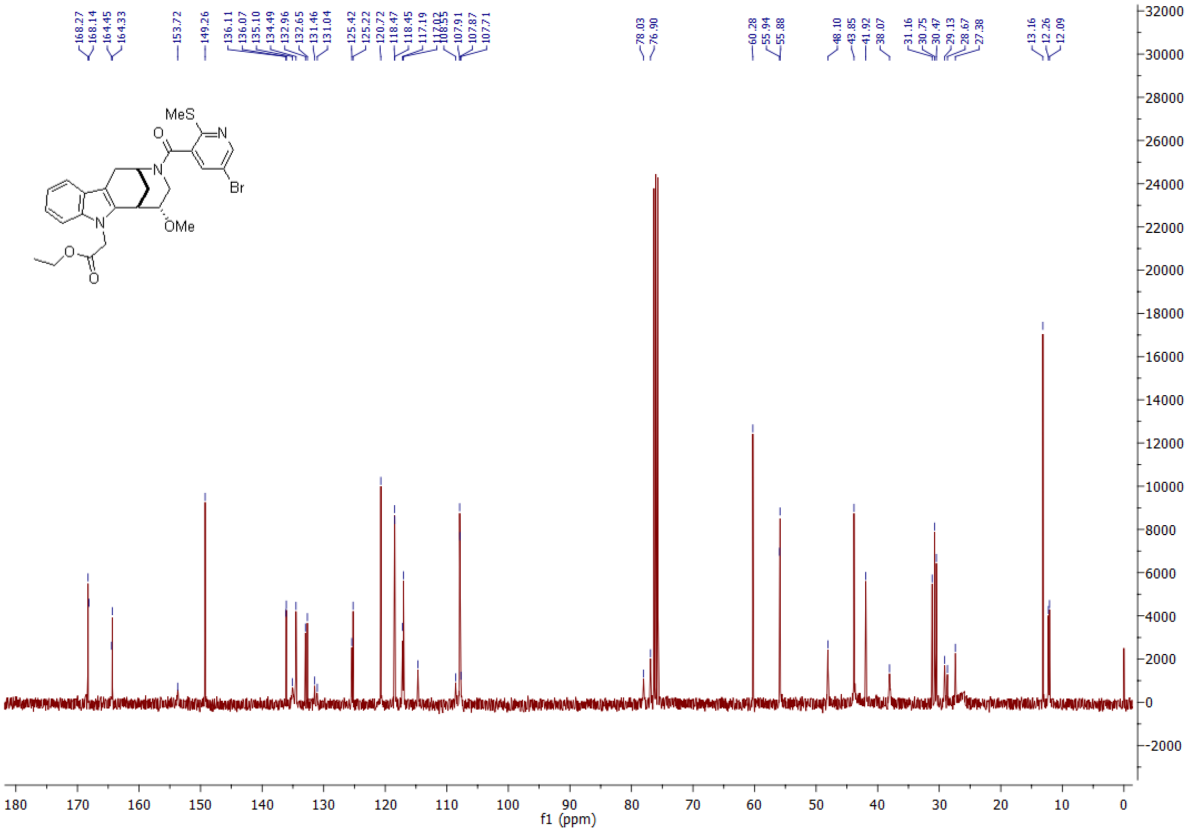

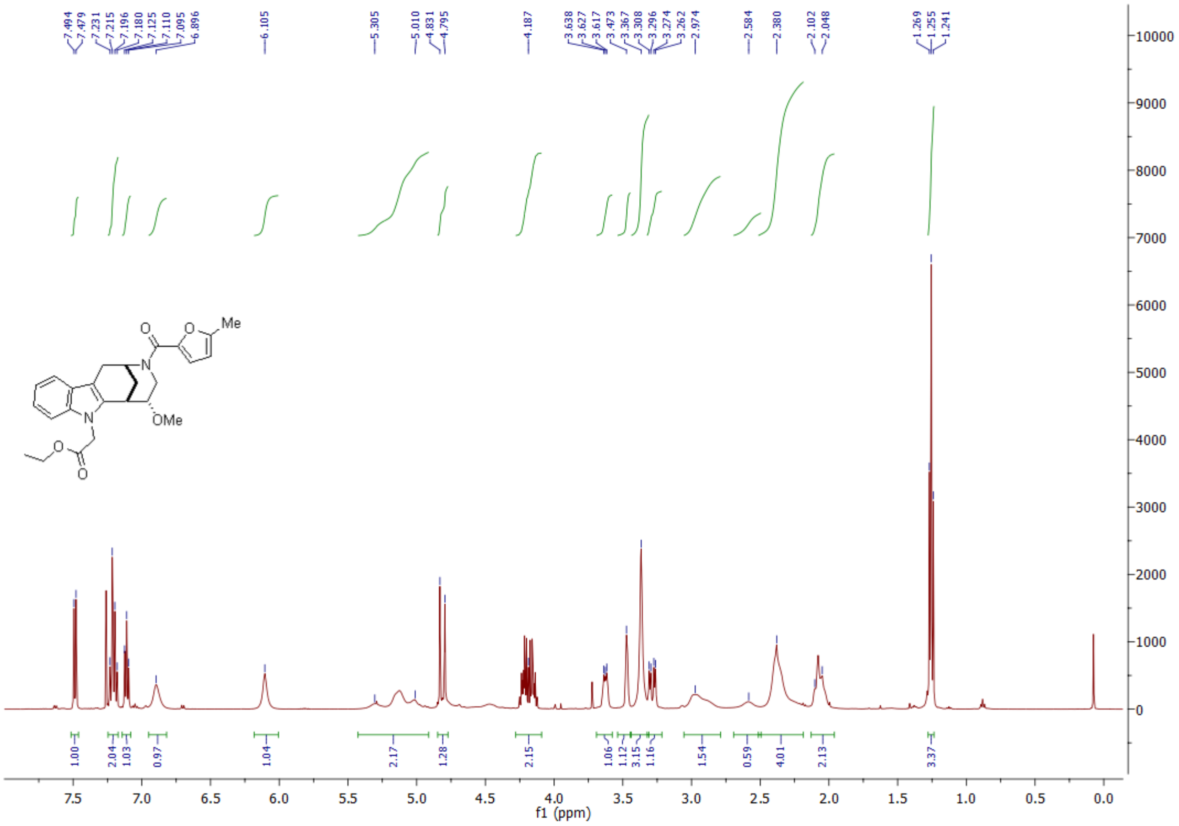
7.494
7.479
7.231
7.215
7.196
7.180
7.125
7.110
7.095
6.896
6.105
5.305
5.010
4.831
4.795
4.187
3.638
3.627
3.617
3.473
3.367
3.308
3.296
3.274
3.262
2.974
2.584
2.380
2.102
2.048
1.269
1.255
1.241
Me
O
O
N
N
OMe
O
O
1.00
2.04
1.03
0.97
1.04
2.17
1.28
2.15
1.06
1.12
3.15
1.16
1.54
0.59
4.01
2.13
3.37
f1 (ppm)

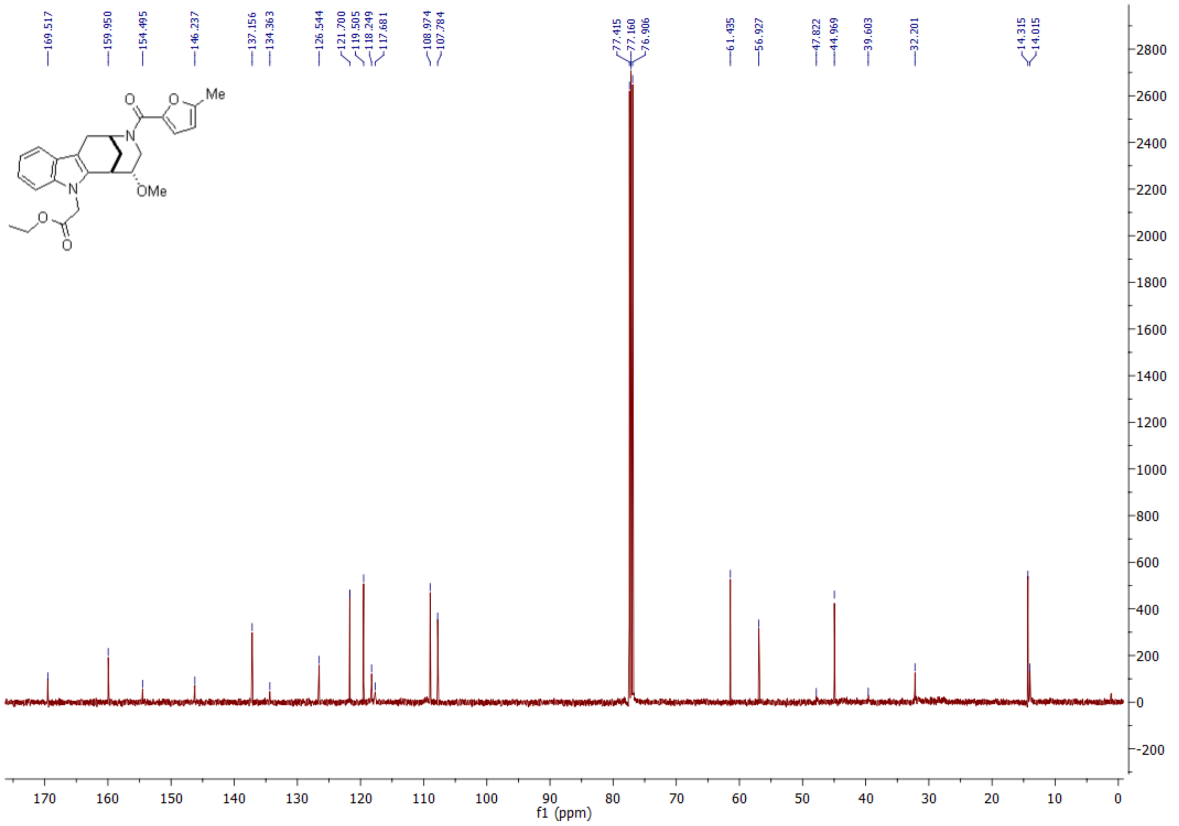
169.517
159.950
154.495
146.237
137.156
134.363
126.544
121.700
119.505
118.249
117.681
108.974
107.784
77.415
77.160
76.906
61.435
56.927
47.822
44.969
39.603
32.201
14.315
14.015
Me
O
O
N
N
OMe
O
O
f1 (ppm)

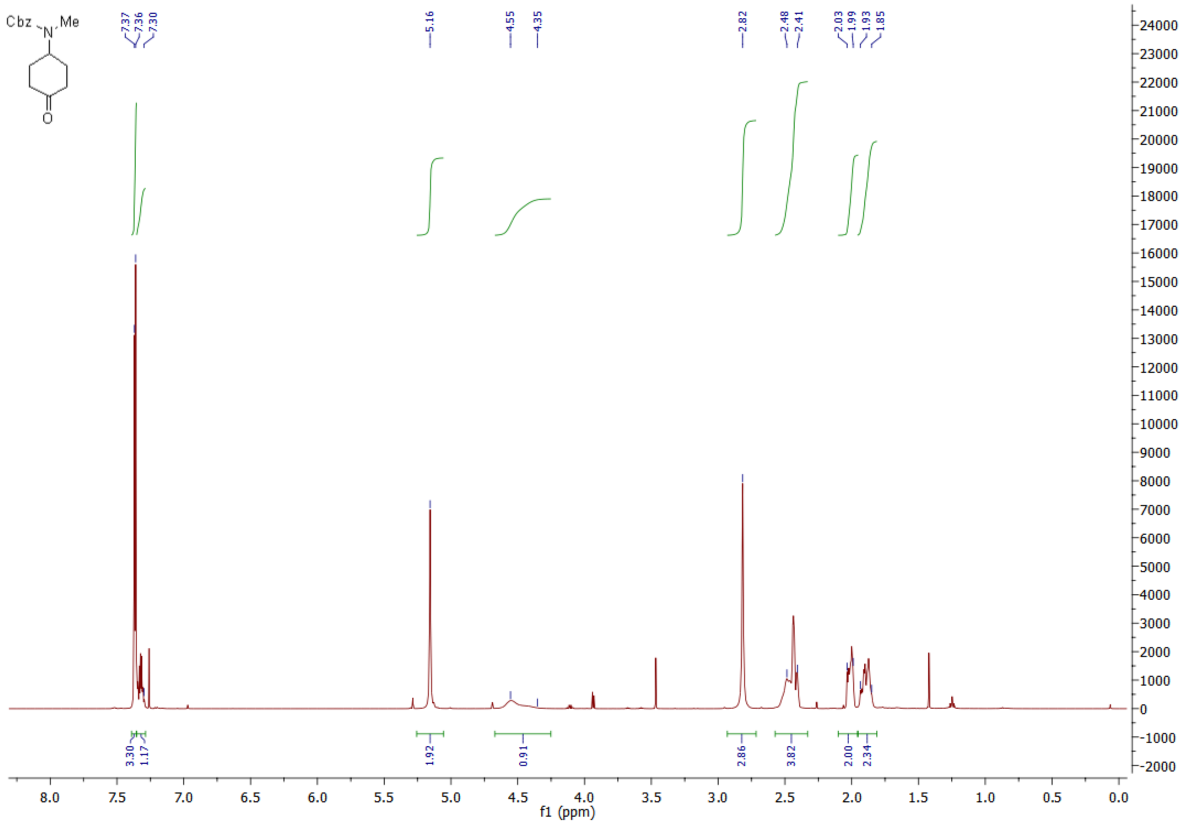
Cbz
N
Me
O
7.37
7.36
7.30
5.16
4.55
4.35
2.82
2.48
2.41
2.03
1.99
1.93
1.85
3.30
1.17
1.92
0.91
2.86
3.82
2.00
2.34
f1 (ppm)

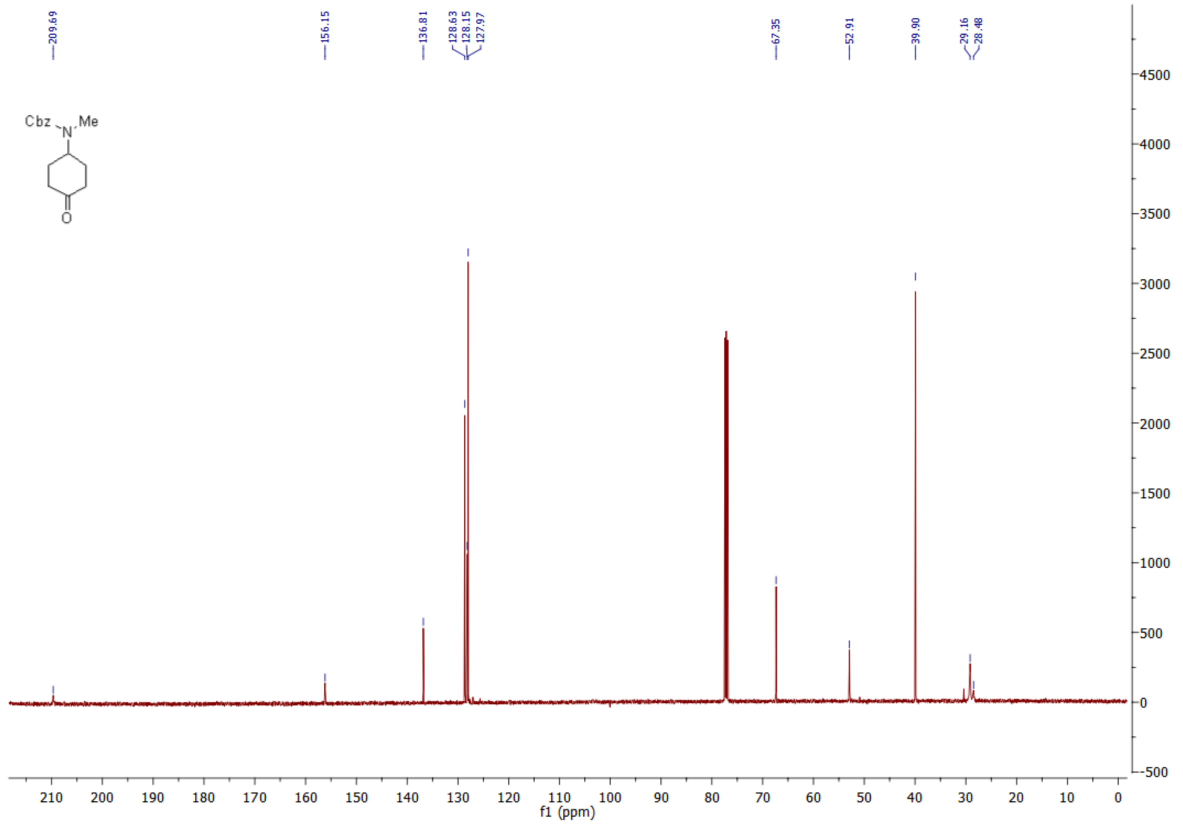
Cbz
N
Me
O
209.69
156.15
136.81
128.63
128.15
127.97
67.35
52.91
39.90
29.16
28.48
f1 (ppm)

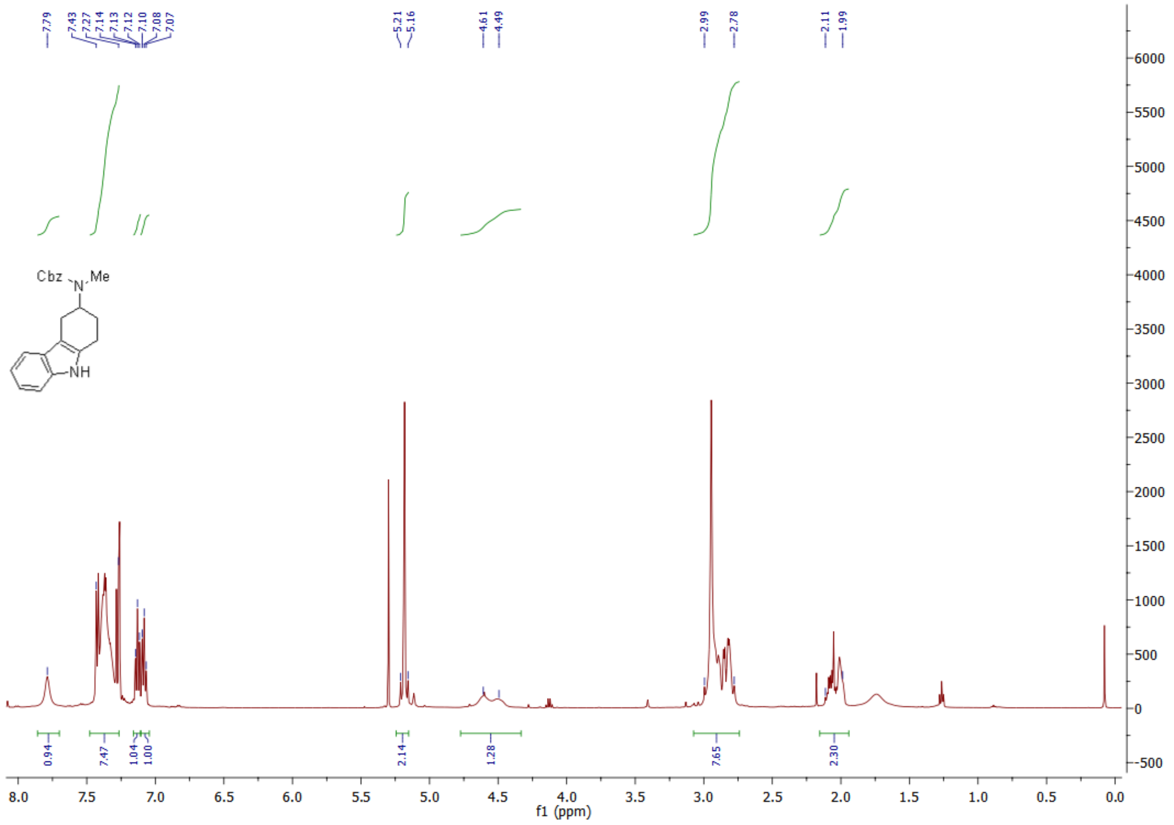

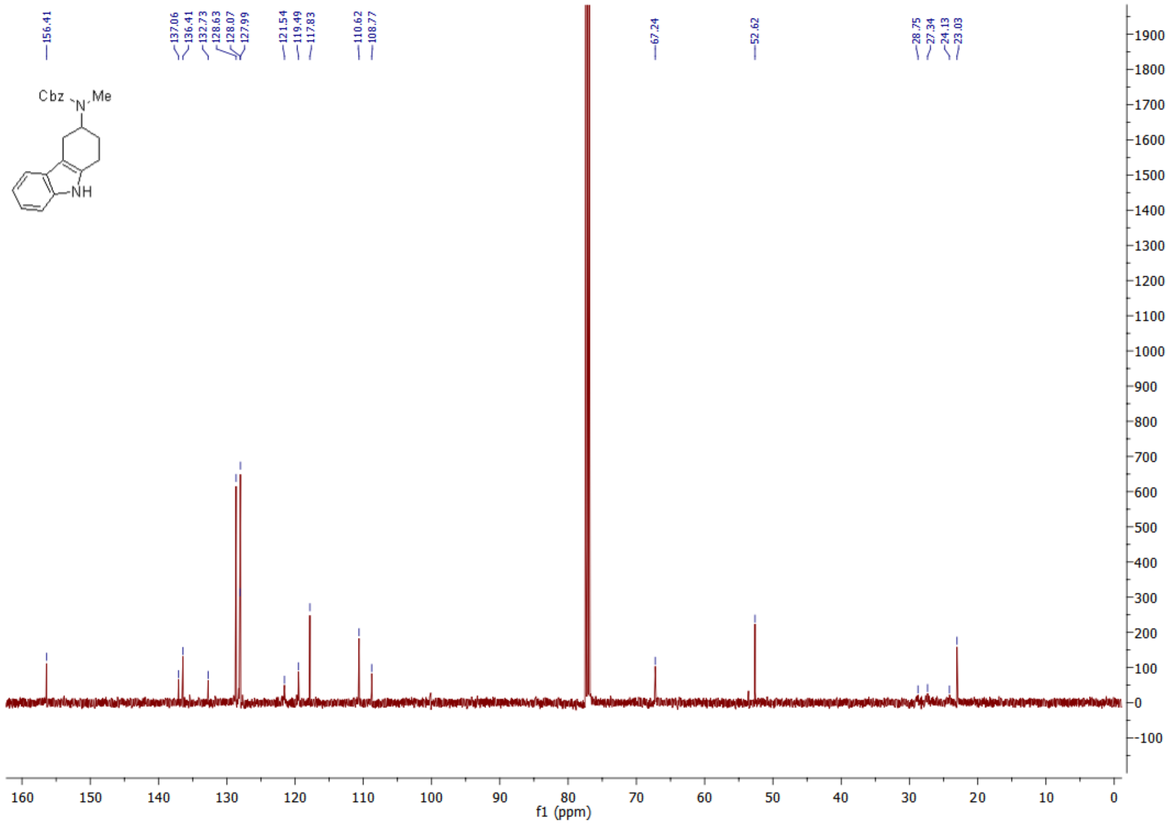

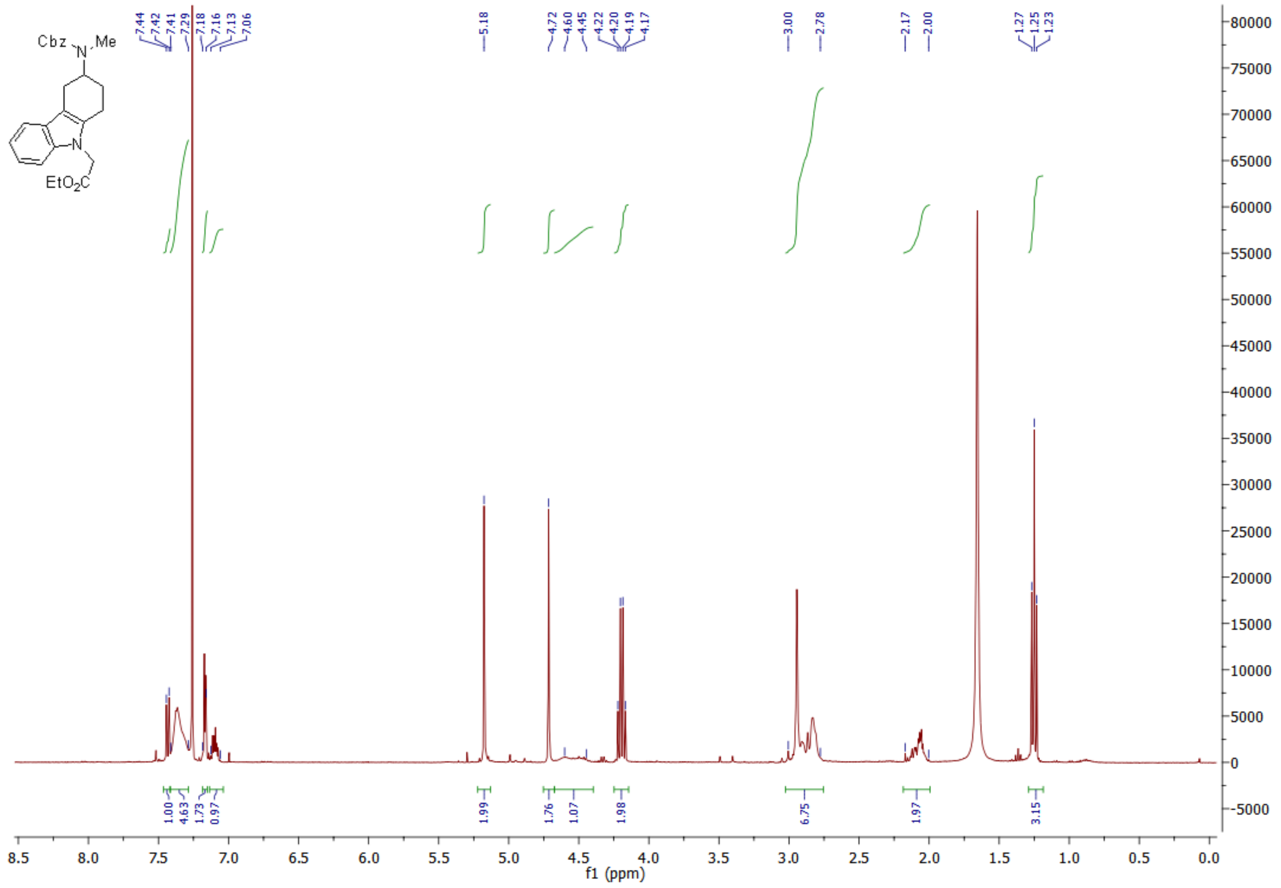
Cbz
N
Me
N
EtO2C
7.44
7.42
7.41
7.29
7.18
7.16
7.13
7.06
5.18
4.72
4.60
4.45
4.22
4.20
4.19
4.17
3.00
2.78
2.17
2.00
1.27
1.25
1.23
1.00
4.63
1.73
0.97
1.99
1.76
1.07
1.98
6.75
1.97
3.15
f1 (ppm)

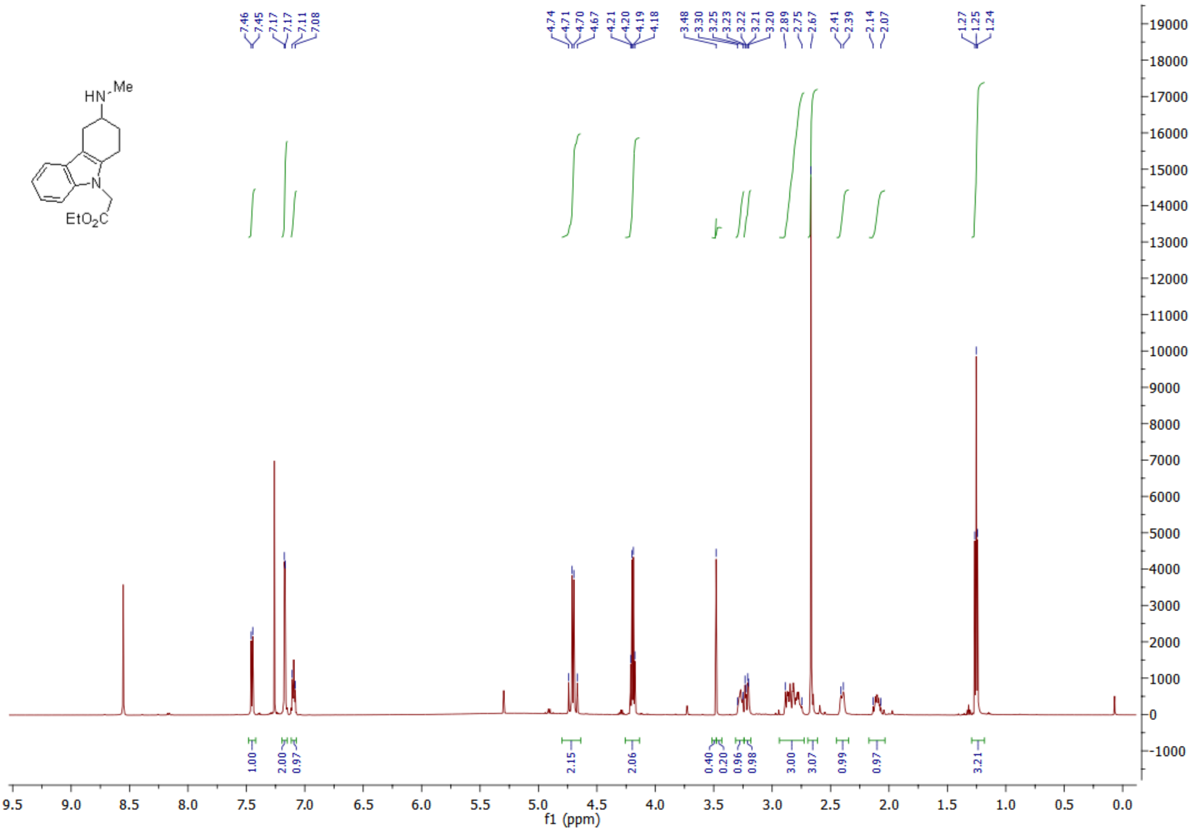
7.46
7.45
7.17
7.17
7.11
7.08
4.74
4.71
4.70
4.67
4.21
4.20
4.19
4.18
3.48
3.30
3.25
3.23
3.22
3.21
3.20
2.89
2.75
2.67
2.41
2.39
2.14
2.07
1.27
1.25
1.24
HN
Me
N
EtO2C
1.00
2.00
0.97
2.15
2.06
0.40
0.20
0.96
0.98
3.00
3.07
0.99
0.97
3.21
f1 (ppm)

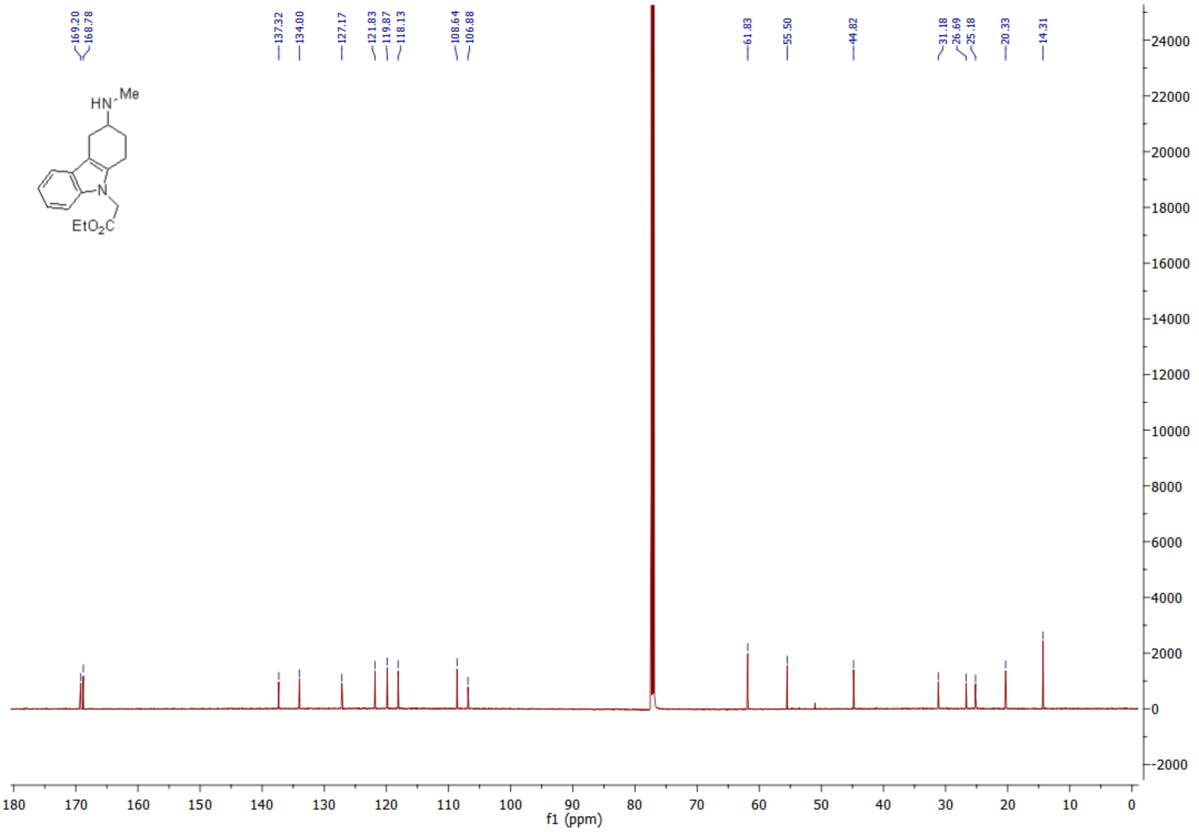
169.20
168.78
137.32
134.00
127.17
121.83
119.87
118.13
108.64
106.88
61.83
55.50
44.82
31.18
26.69
25.18
20.33
14.31
HN
Me
N
EtO2C
f1 (ppm)

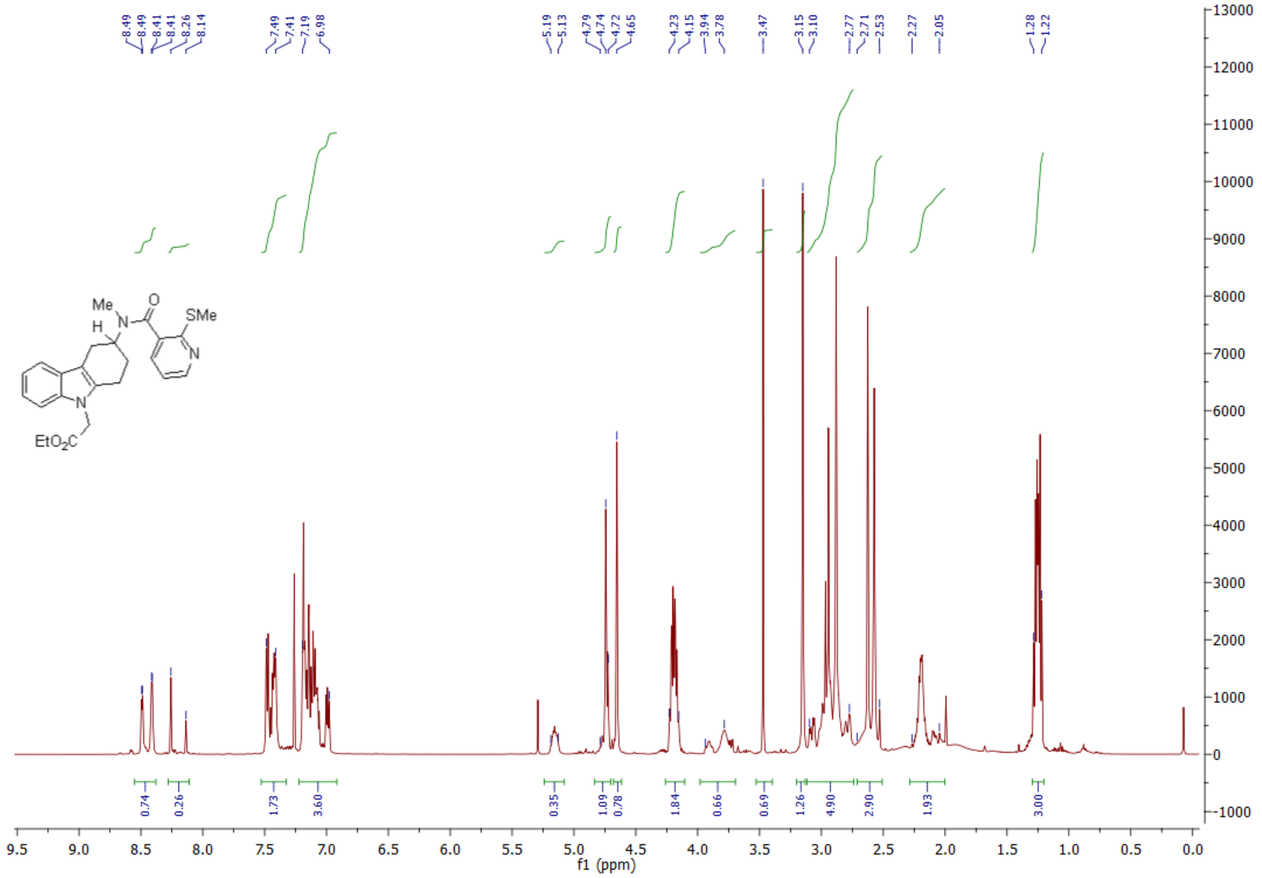
8.49
8.49
8.41
8.41
8.26
8.14
7.49
7.41
7.19
6.98
5.19
5.13
4.79
4.74
4.72
4.65
4.23
4.15
3.94
3.78
3.47
3.15
3.10
2.77
2.71
2.53
2.27
2.05
1.28
1.22
Me
H
N
O
SMe
N
N
EtO2C
0.74
0.26
1.73
3.60
0.35
1.09
0.78
1.84
0.66
0.69
1.26
4.90
2.90
1.93
3.00
f1 (ppm)

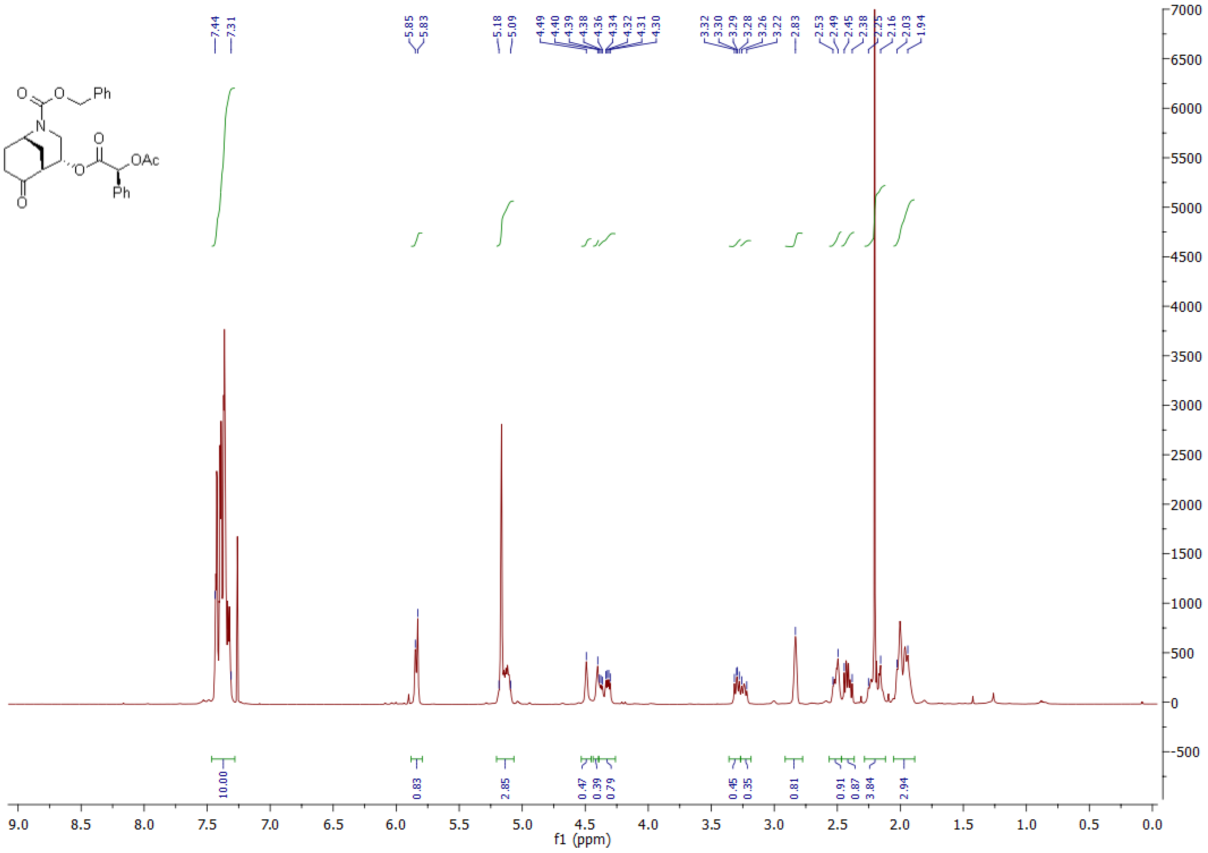
7.44
7.31
5.85
5.83
5.18
5.09
4.49
4.40
4.39
4.38
4.36
4.34
4.32
4.31
4.30
3.32
3.30
3.29
3.28
3.26
3.22
2.83
2.53
2.49
2.45
2.38
2.25
2.16
2.03
1.94
OAc
Ph
10.00
0.83
2.85
0.47
0.39
0.79
0.45
0.35
0.81
0.91
0.87
3.84
2.94
f1 (ppm)

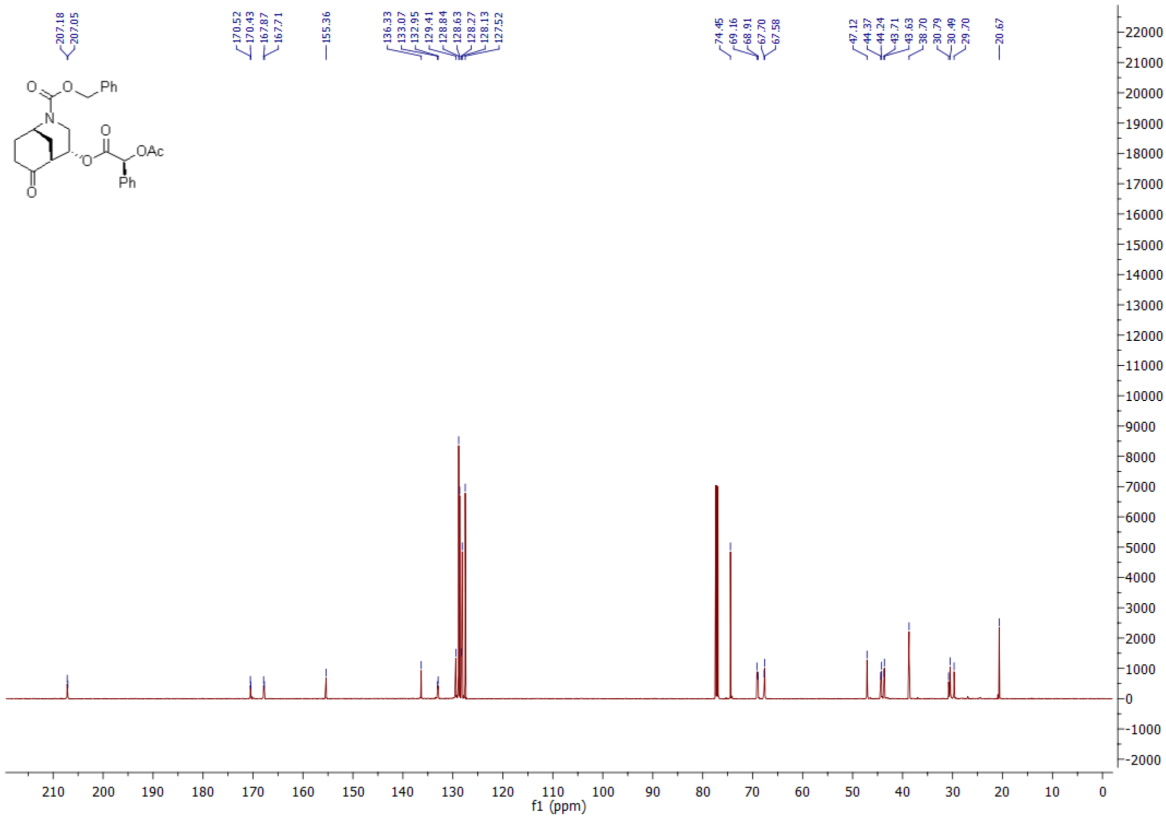
207.18
207.05
170.52
170.43
167.87
167.71
155.36
136.33
133.07
132.95
129.41
128.84
128.63
128.27
128.13
127.52
74.45
69.16
68.91
67.70
67.58
47.12
44.37
44.24
43.71
43.63
38.70
30.79
30.49
29.70
20.67
OAc
Ph
f1 (ppm)

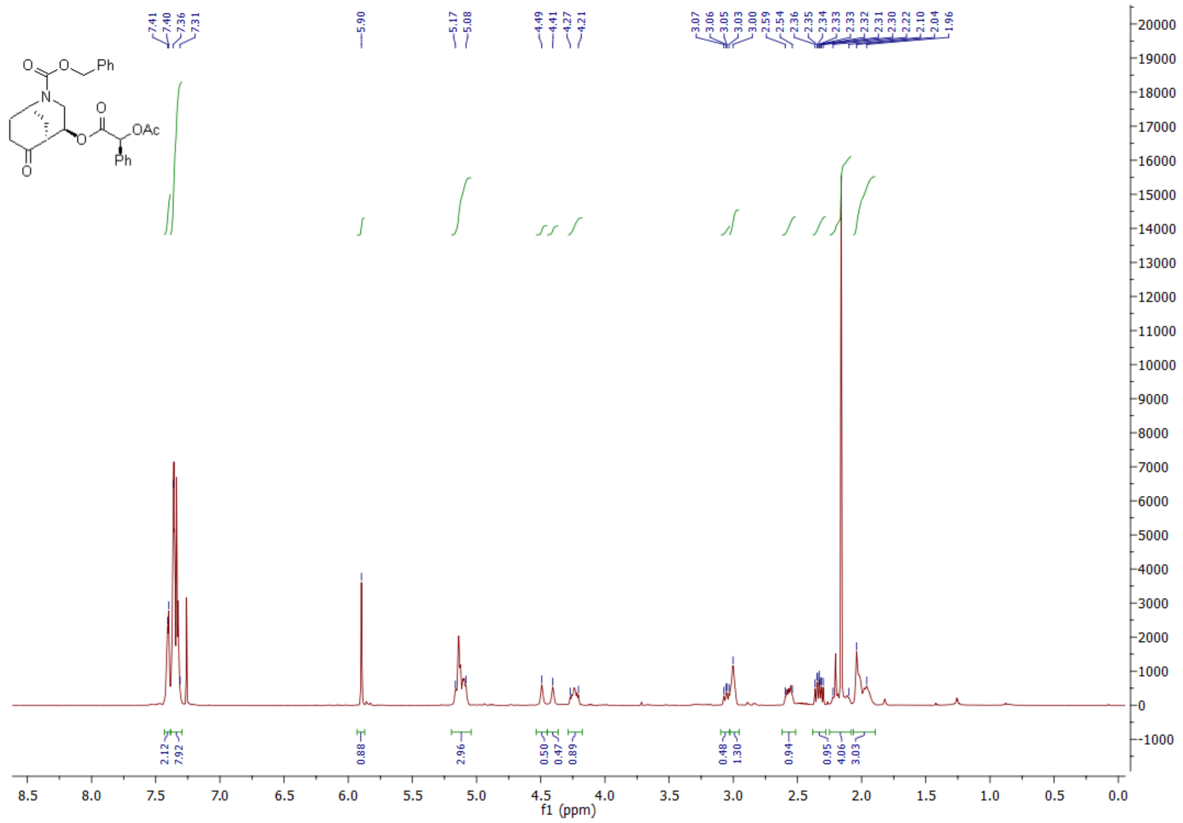
7.41
7.40
7.36
7.31
5.90
5.17
5.08
4.49
4.41
4.27
4.21
3.07
3.06
3.05
3.03
3.00
2.59
2.54
2.36
2.35
2.34
2.33
2.33
2.32
2.31
2.30
2.22
2.10
2.04
1.96
2.12
7.92
0.88
2.96
0.50
0.47
0.89
0.48
1.30
0.94
0.95
4.06
3.03
f1 (ppm)

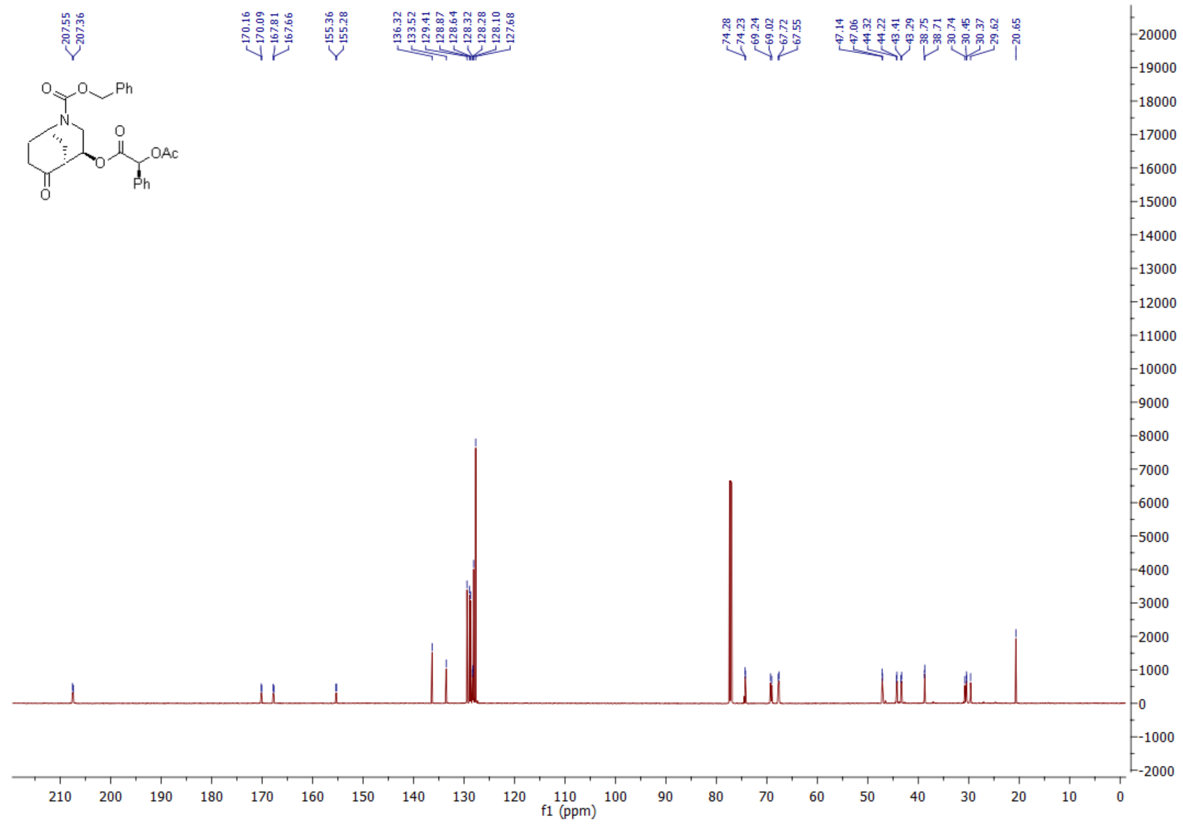
207.55
207.36
170.16
170.09
167.81
167.66
155.36
155.28
136.32
133.52
129.41
128.87
128.64
128.32
128.28
128.10
127.68
74.28
74.23
69.24
69.02
67.72
67.55
47.14
47.06
44.32
44.22
43.41
43.29
38.75
38.71
30.74
30.45
30.37
29.62
20.65
f1 (ppm)

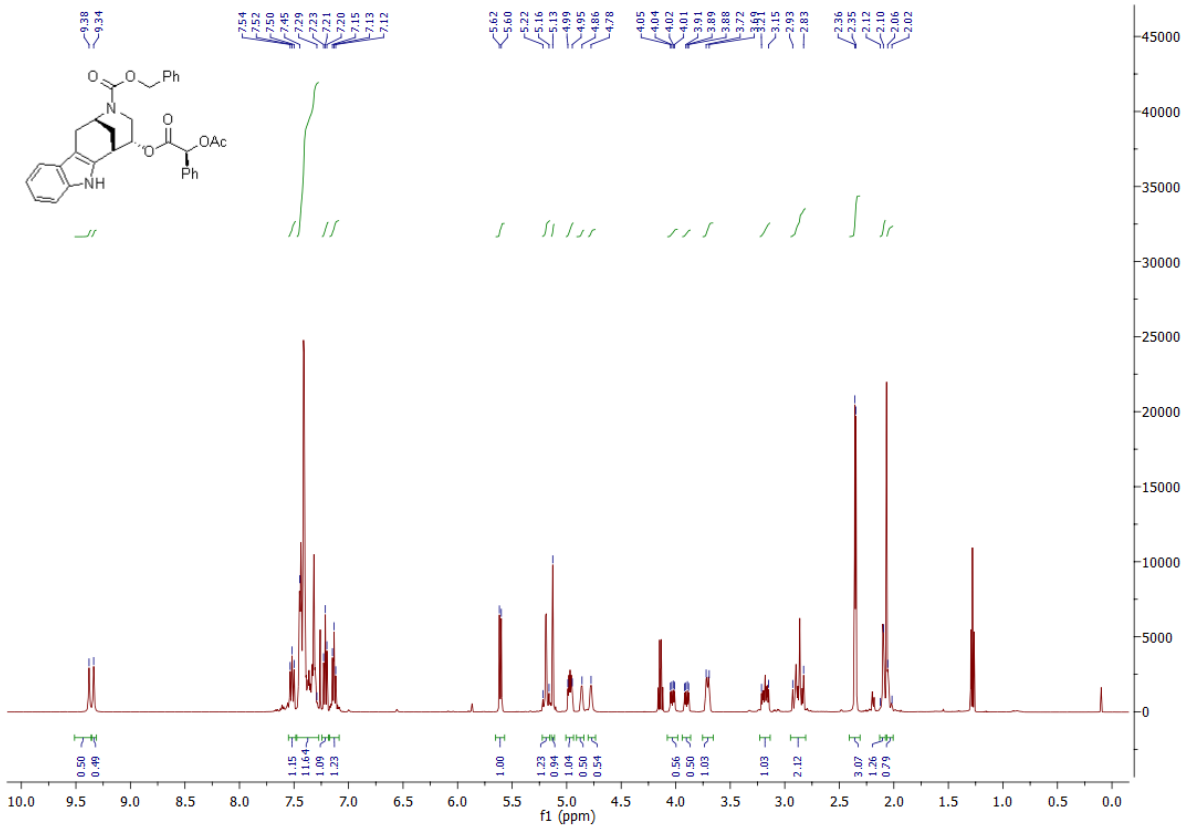

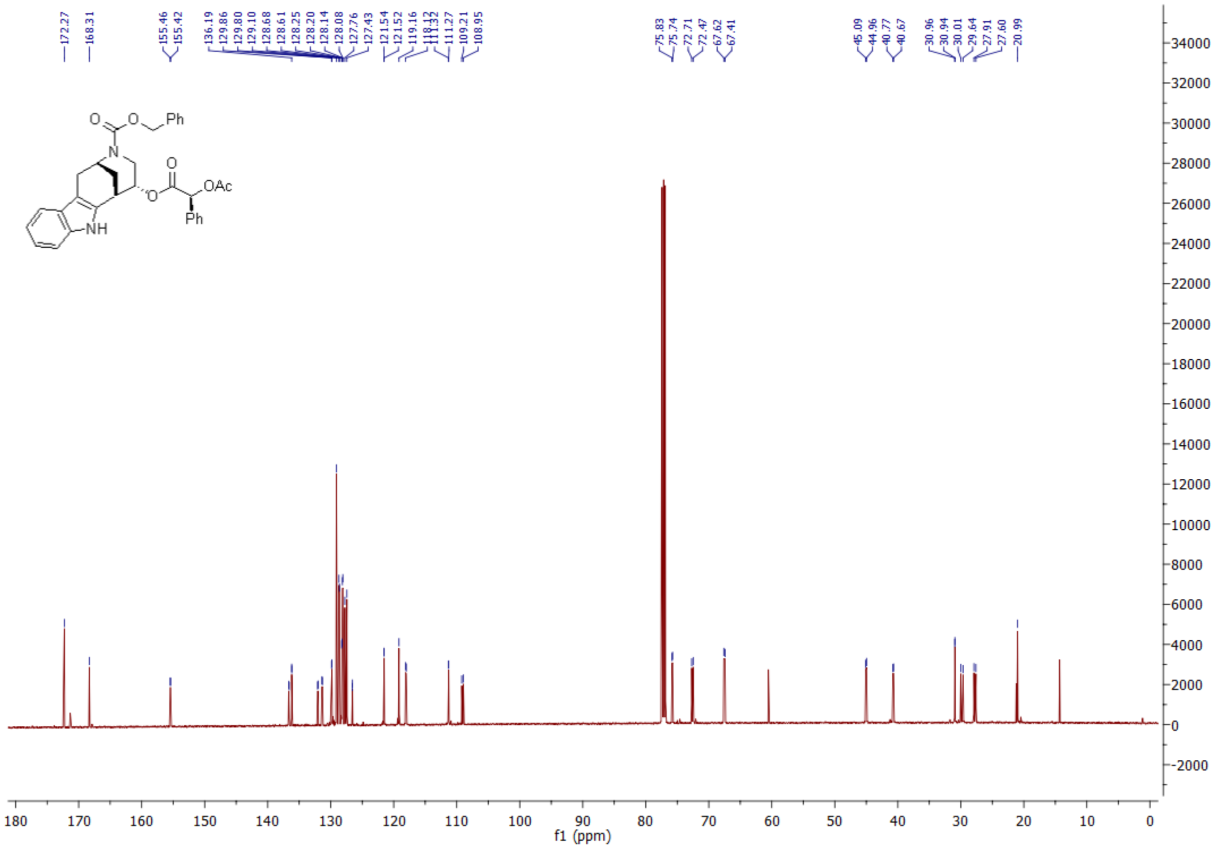

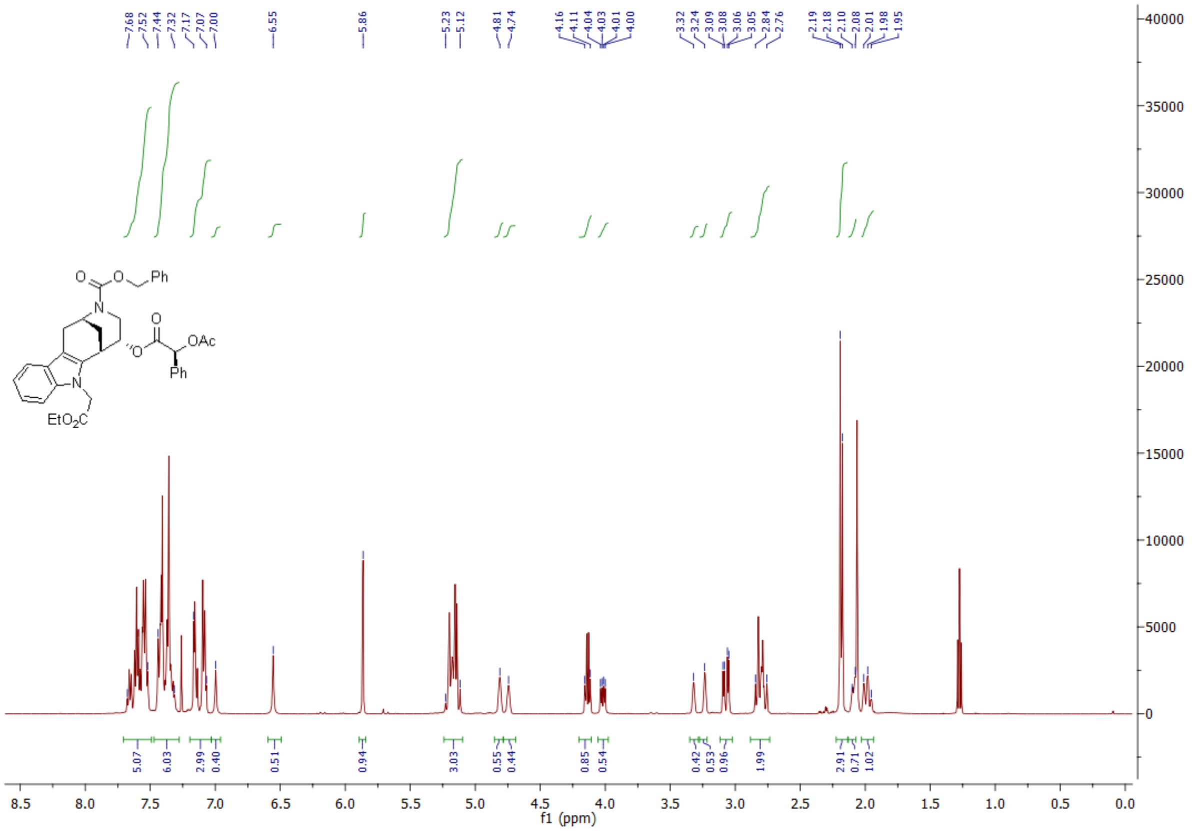

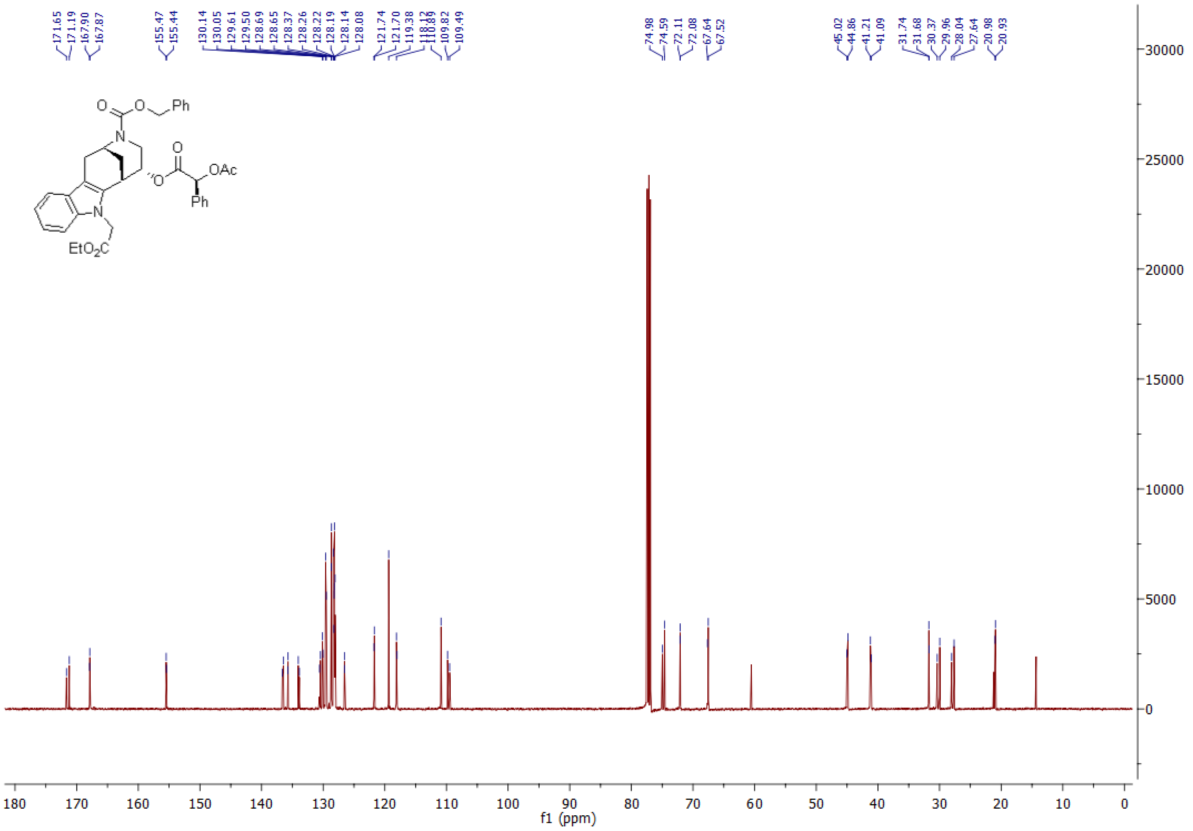

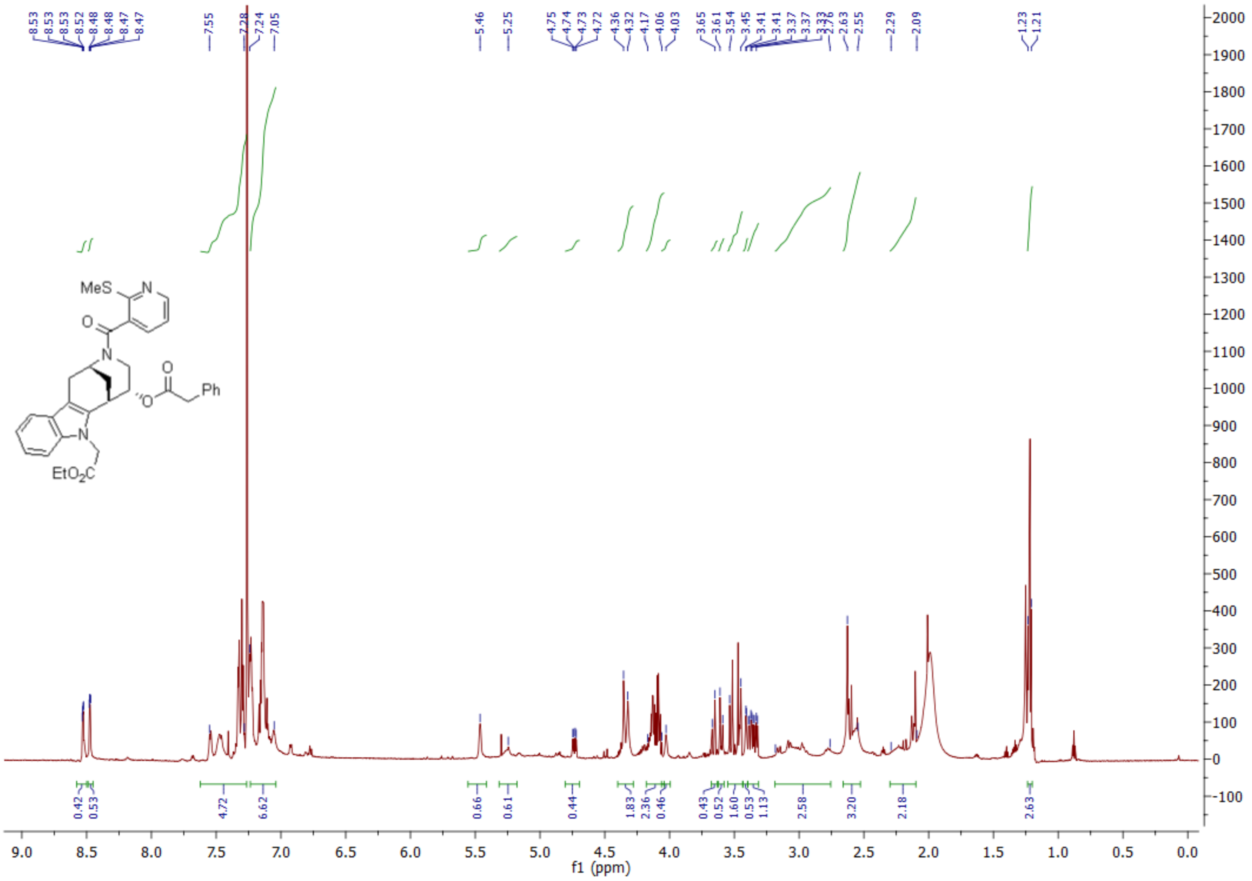

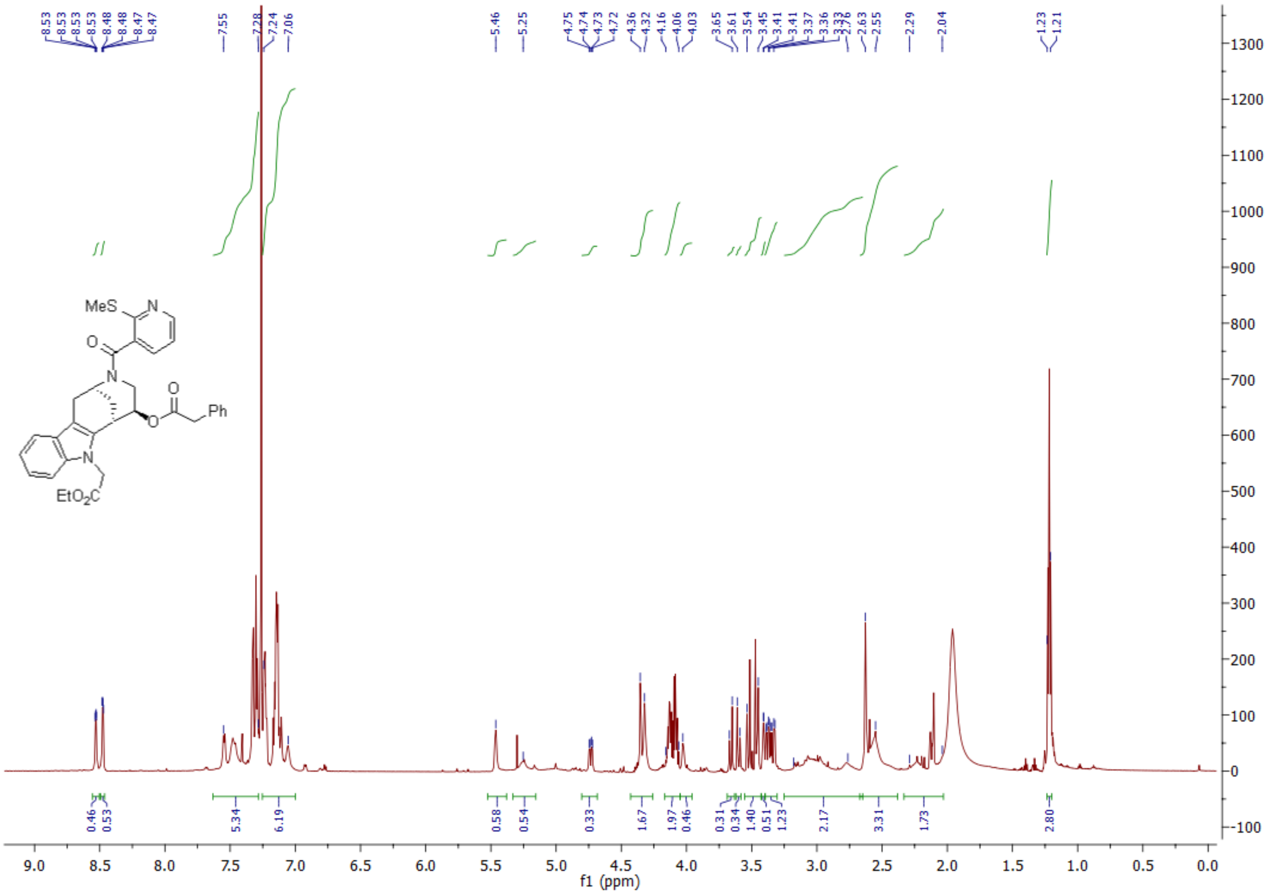

Chiral Chromatograms:

(+/-)-Glupin-1:

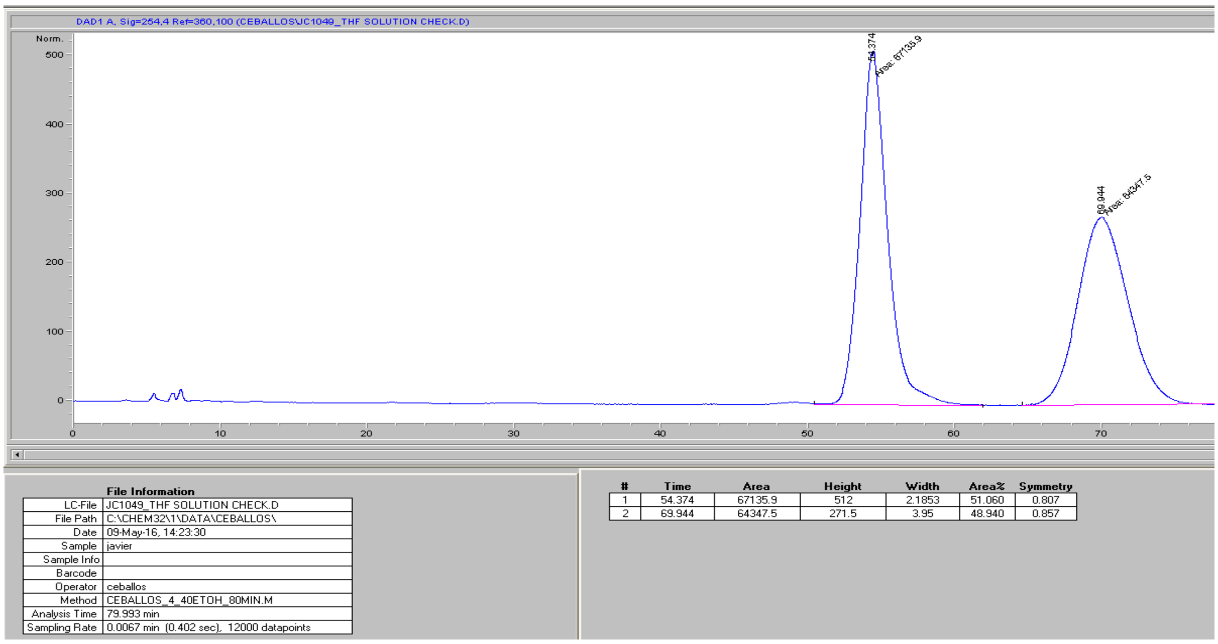

File Information

LC-File	JC1049_THF SOLUTION CHECK.D
File Path	C:\CHEM32\1\DATA\CEBALLOS\
Date	09-May-16, 14:23:30
Sample	javier
Sample Info	
Barcode	
Operator	ceballos
Method	CEBALLOS_4_40ETOH_80MIN.M
Analysis Time	79.993 min
Sampling Rate	0.0067 min (0.402 sec), 12000 datapoints

#	Time	Area	Height	Width	Area%	Symmetry
1	54.374	67135.9	512	2.1853	51.060	0.807
2	69.944	64347.5	271.5	3.95	48.940	0.857

(+)-Glupin-1:

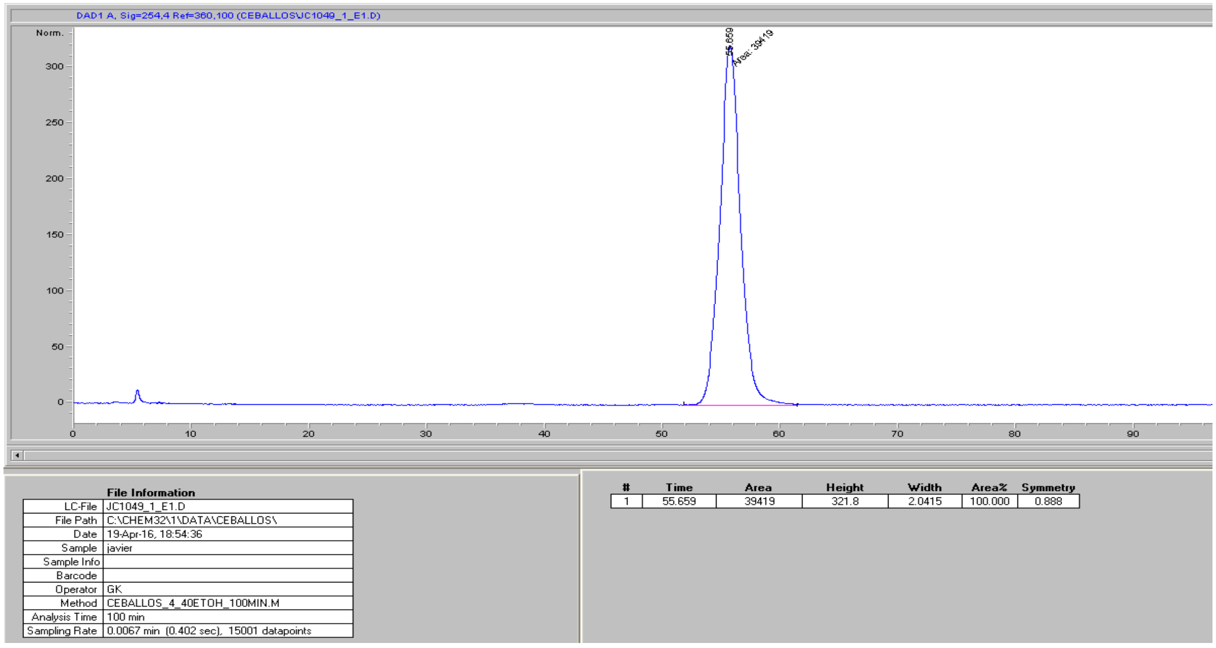

File Information

LC-File	JC1049_1_E1.D
File Path	C:\CHEM32\1\DATA\CEBALLOS\
Date	19-Apr-16, 18:54:36
Sample	javier
Sample Info	
Barcode	
Operator	GK
Method	CEBALLOS_4_40ETOH_100MIN.M
Analysis Time	100 min
Sampling Rate	0.0067 min (0.402 sec), 15001 datapoints

#	Time	Area	Height	Width	Area%	Symmetry
1	55.659	39419	321.8	2.0415	100.000	0.888

(-)-Glupin-1:

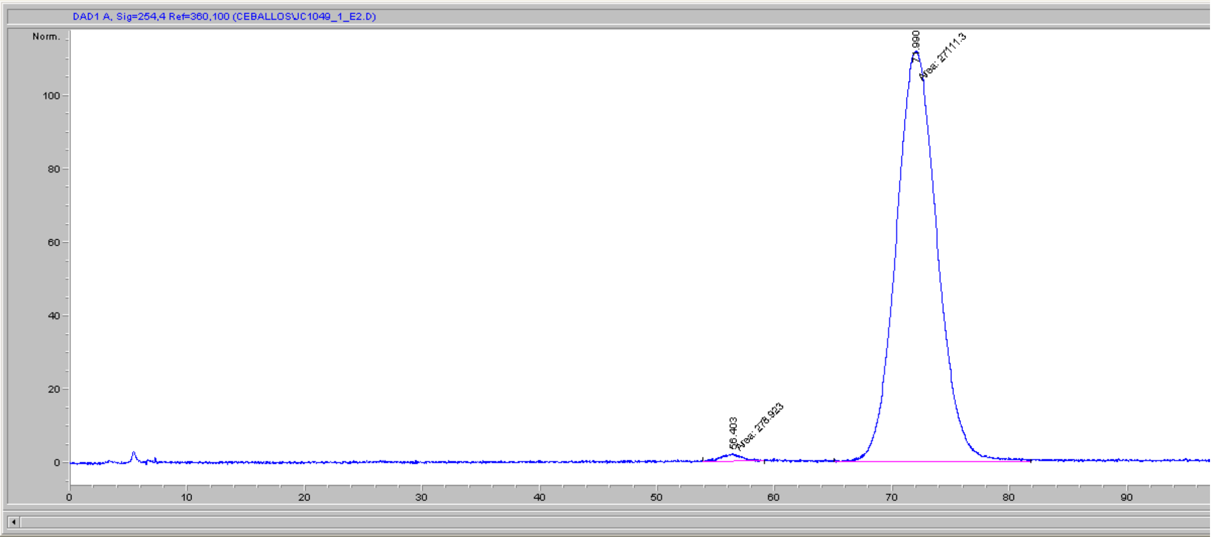

File Information

LC-File	JC1049_1_E2.D
File Path	C:\CHEM32\1\DATA\CEBALLOS\
Date	19-Apr-16, 20:36:01
Sample	javier
Sample Info	
Barcode	
Operator	GK
Method	CEBALLOS_4_40ETOH_100MIN.M
Analysis Time	100 min
Sampling Rate	0.0067 min (0.402 sec), 15001 datapoints

#	Time	Area	Height	Width	Area%	Symmetry
1	56.403	278.9	2.2	2.1128	1.018	1.469
2	71.99	27111.3	111.7	4.0439	98.982	0.928

Preparative Separation of (+) and (-)-Glupin-1:

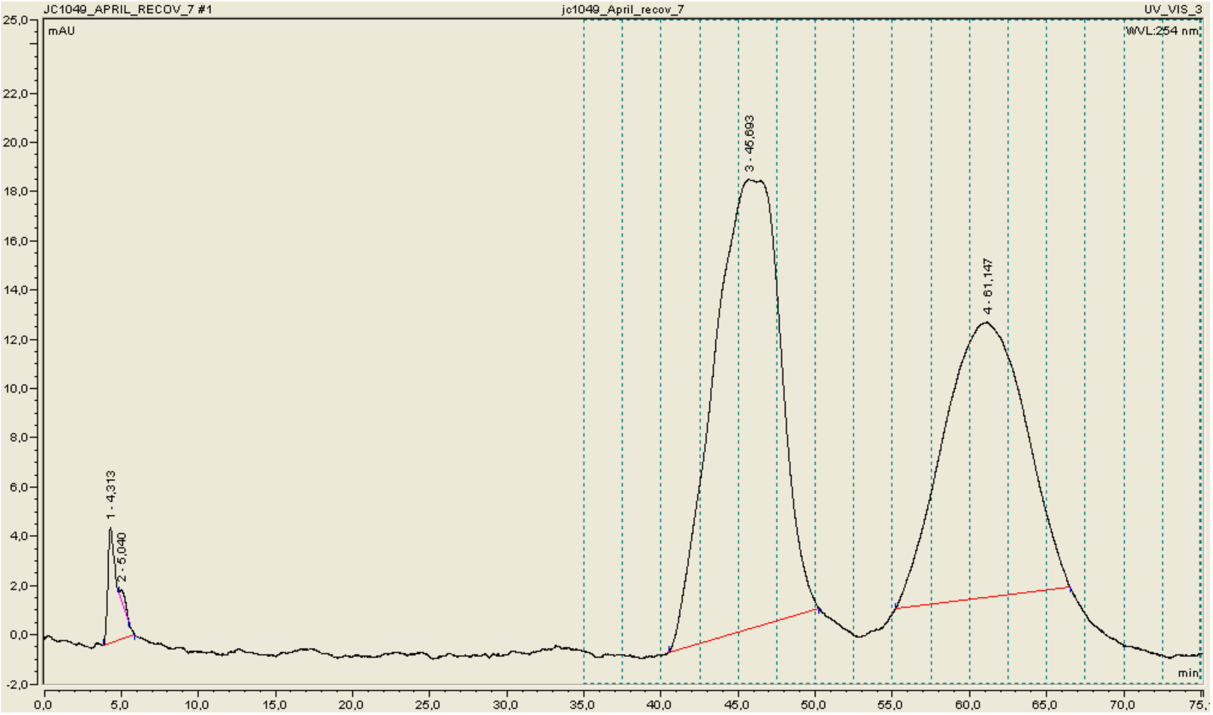

Indomorphan MS and UV absoprtion chromatograms

General Procedure:

Sample dilution:

A volume of 3µL of 10 mM DMSO solution is diluted with 27µL of a acetonitrile/water mixture (50/50 vol./vol. %)

LC Method parameters

Run time	5 min
Column	Shim-Pack XR-ODS, 2,0mm i.d. x 50mm, S/N 80822262, 2,2µm
Flow rate	0,5 mL/min
Eluent A	water 0,1% formic acid
Eluent B	acetonitrile 0,1% formic acid
Wash solvent	methanol
Rinsing volume	500µL
Initial eluent B concentration	10%

Gradient

Time [min]	A [%]	B [%]
0	90	10
2,5	0	100
3,75	0	100
3,76	90	10
5	90	10

Column temperature	30 °C
Injection volume	4 µL

PDA	190 – 800 nm
Wavelength for purity calc.	210 nm Band Width 4 nm
Sampling rate	3,125 Hz

MS Method parameters

Acquisition mode:	scan
Ionization Mode:	DUIS (ESI, APCI), negative and positive mode
Start m/z:	100
End m/z:	2000
Scan speed:	15000u/sec
Event Time:	0,15s
DL Temperature:	230°C
Nebulizing Gas Flow:	1,5L/min
Heat block:	230°C
Drying Gas Flow:	15L/min

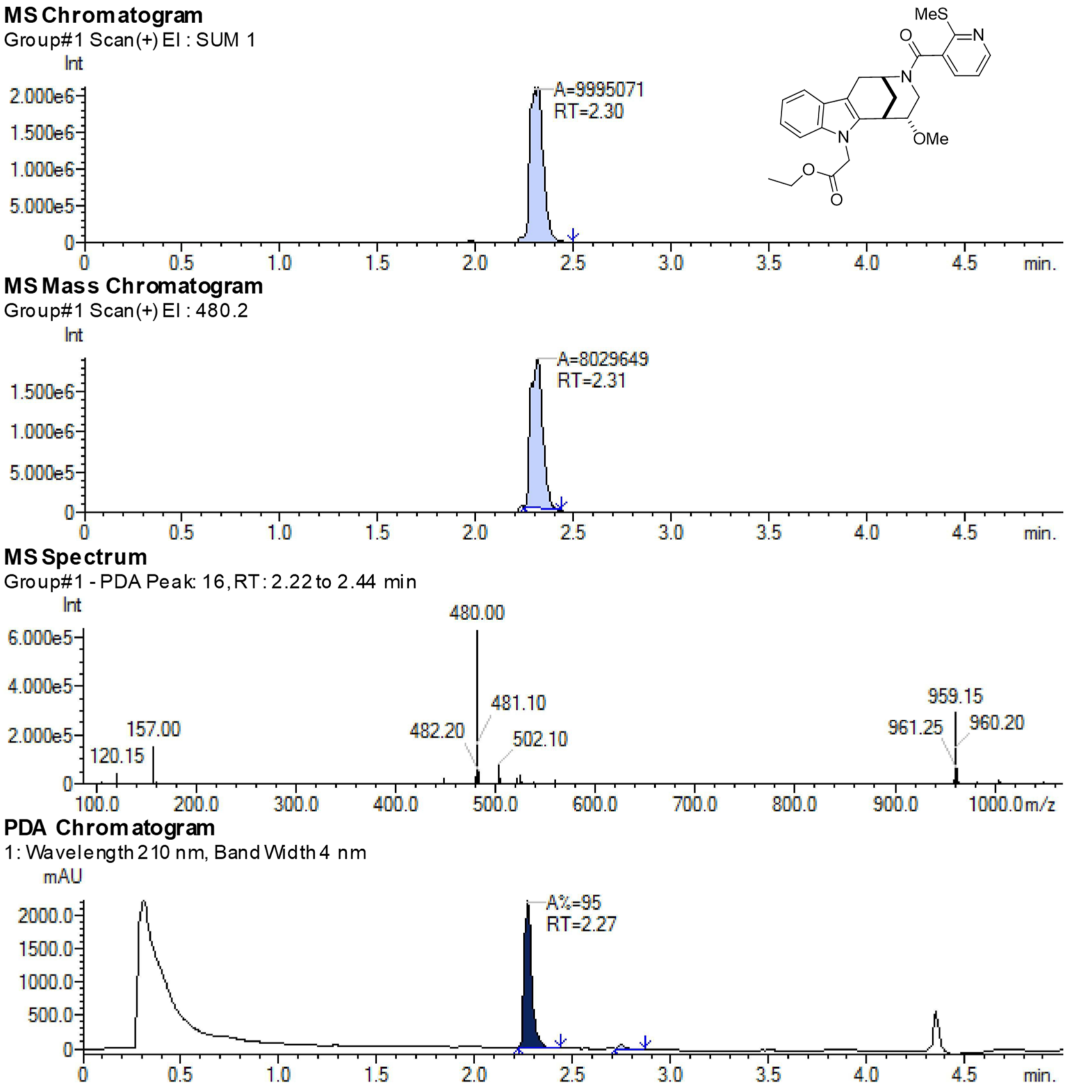
MS Chromatogram
Group#1 Scan(+) EI : SUM 1
Int
A=9995071
RT=2.30
min.
MS Mass Chromatogram
Group#1 Scan(+) EI : 480.2
Int
A=8029649
RT=2.31
min.
MS Spectrum
Group#1 - PDA Peak: 16, RT: 2.22 to 2.44 min
Int
480.00
481.10
482.20
502.10
157.00
120.15
959.15
961.25
960.20
m/z
PDA Chromatogram
1: Wavelength 210 nm, Band Width 4 nm
mAU
A%=95
RT=2.27
min.

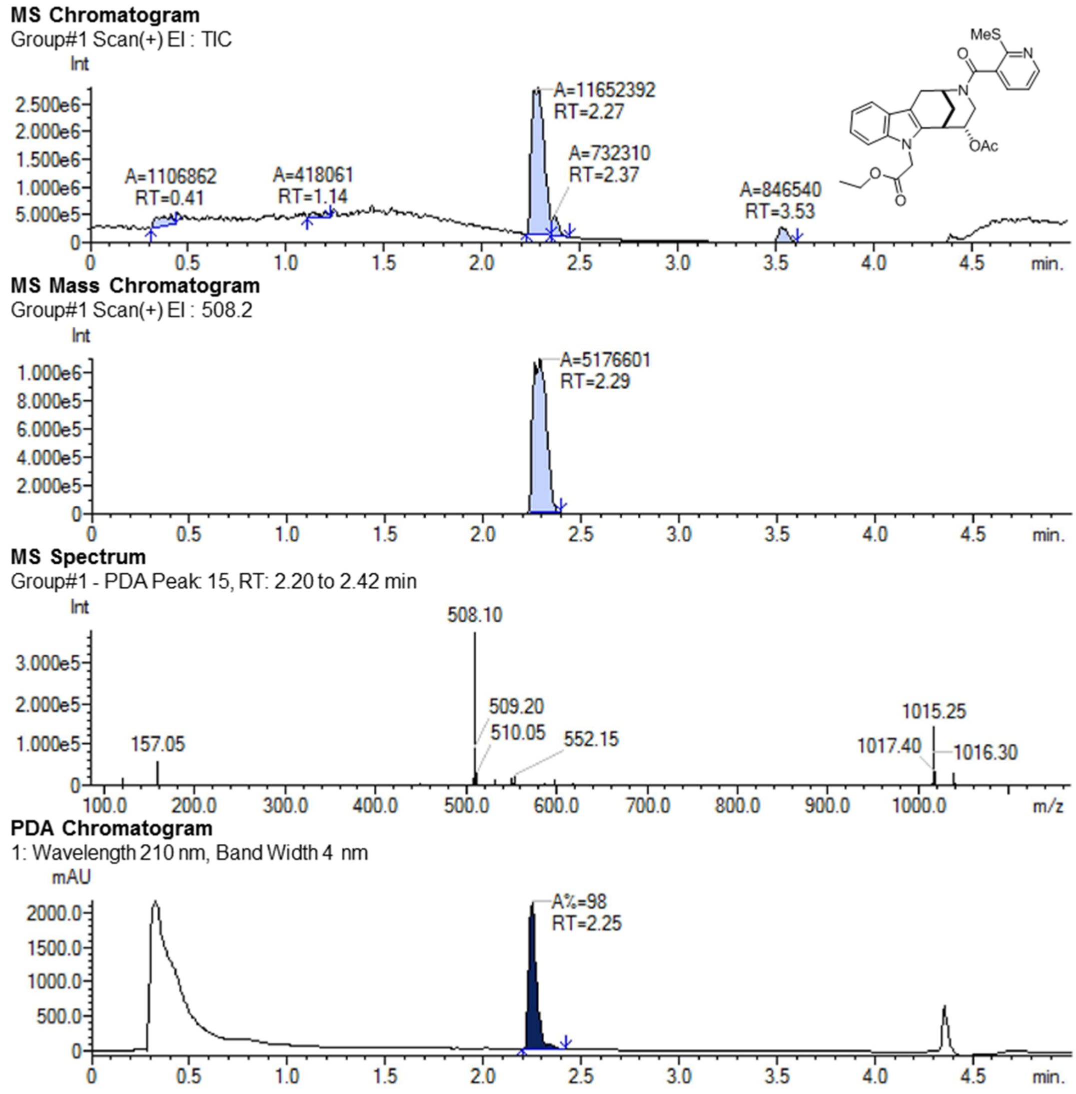
MS Chromatogram
Group#1 Scan(+) EI : TIC
Int
A=1106862
RT=0.41
A=418061
RT=1.14
A=11652392
RT=2.27
A=732310
RT=2.37
A=846540
RT=3.53
MeS
N
O
OAc
min.
MS Mass Chromatogram
Group#1 Scan(+) EI : 508.2
A=5176601
RT=2.29
MS Spectrum
Group#1 - PDA Peak 15, RT: 2.20 to 2.42 min
508.10
509.20
510.05
552.15
157.05
1015.25
1017.40
1016.30
m/z
PDA Chromatogram
1: Wavelength 210 nm, Band Width 4 nm
mAU
A%=98
RT=2.25

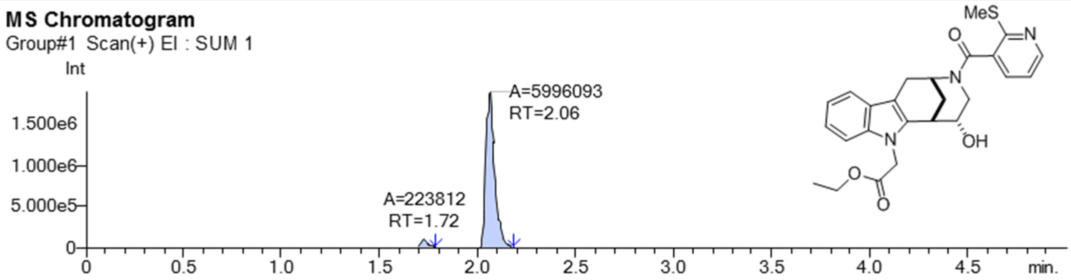
MS Chromatogram
Group#1 Scan(+) EI : SUM 1
Int
1.500e6
1.000e6
5.000e5
0
A=5996093
RT=2.06
A=223812
RT=1.72
0
0.5
1.0
1.5
2.0
2.5
3.0
3.5
4.0
4.5
min.
MeS
N
O
N
OH
N
O
O

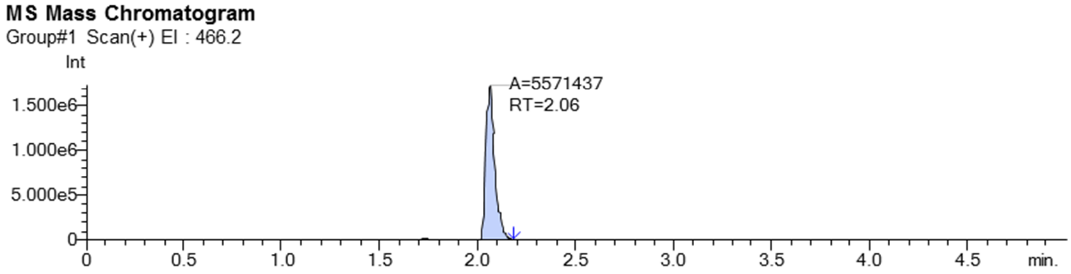
MS Mass Chromatogram
Group#1 Scan(+) EI : 466.2
Int
1.500e6
1.000e6
5.000e5
0
A=5571437
RT=2.06
0
0.5
1.0
1.5
2.0
2.5
3.0
3.5
4.0
4.5
min.

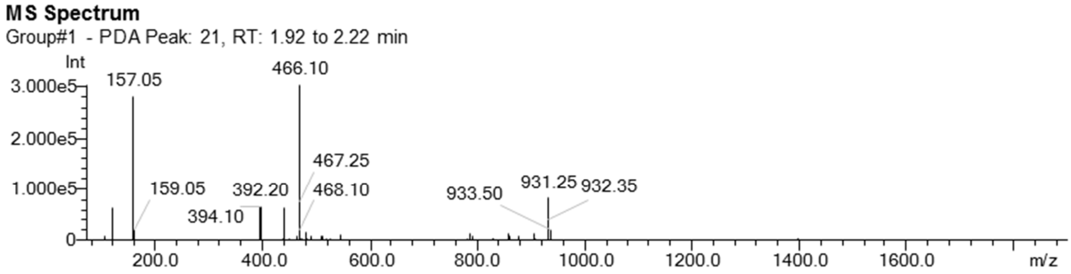
MS Spectrum
Group#1 - PDA Peak: 21, RT: 1.92 to 2.22 min
Int
3.000e5
2.000e5
1.000e5
0
157.05
466.10
467.25
159.05
392.20
468.10
394.10
933.50
931.25
932.35
200.0
400.0
600.0
800.0
1000.0
1200.0
1400.0
1600.0
m/z

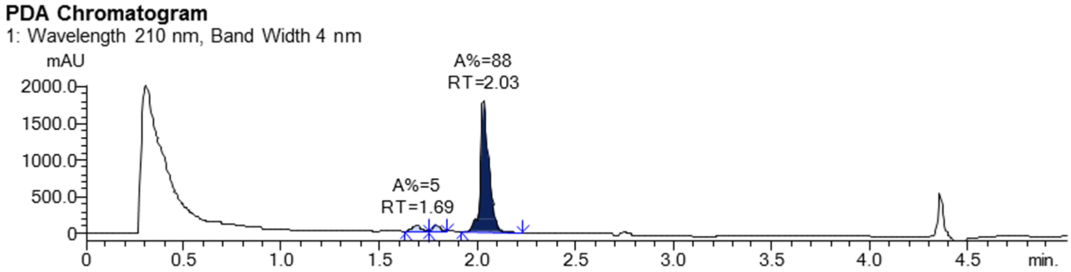
PDA Chromatogram
1: Wavelength 210 nm, Band Width 4 nm
mAU
2000.0
1500.0
1000.0
500.0
0
A%=88
RT=2.03
A%=5
RT=1.69
0
0.5
1.0
1.5
2.0
2.5
3.0
3.5
4.0
4.5
min.

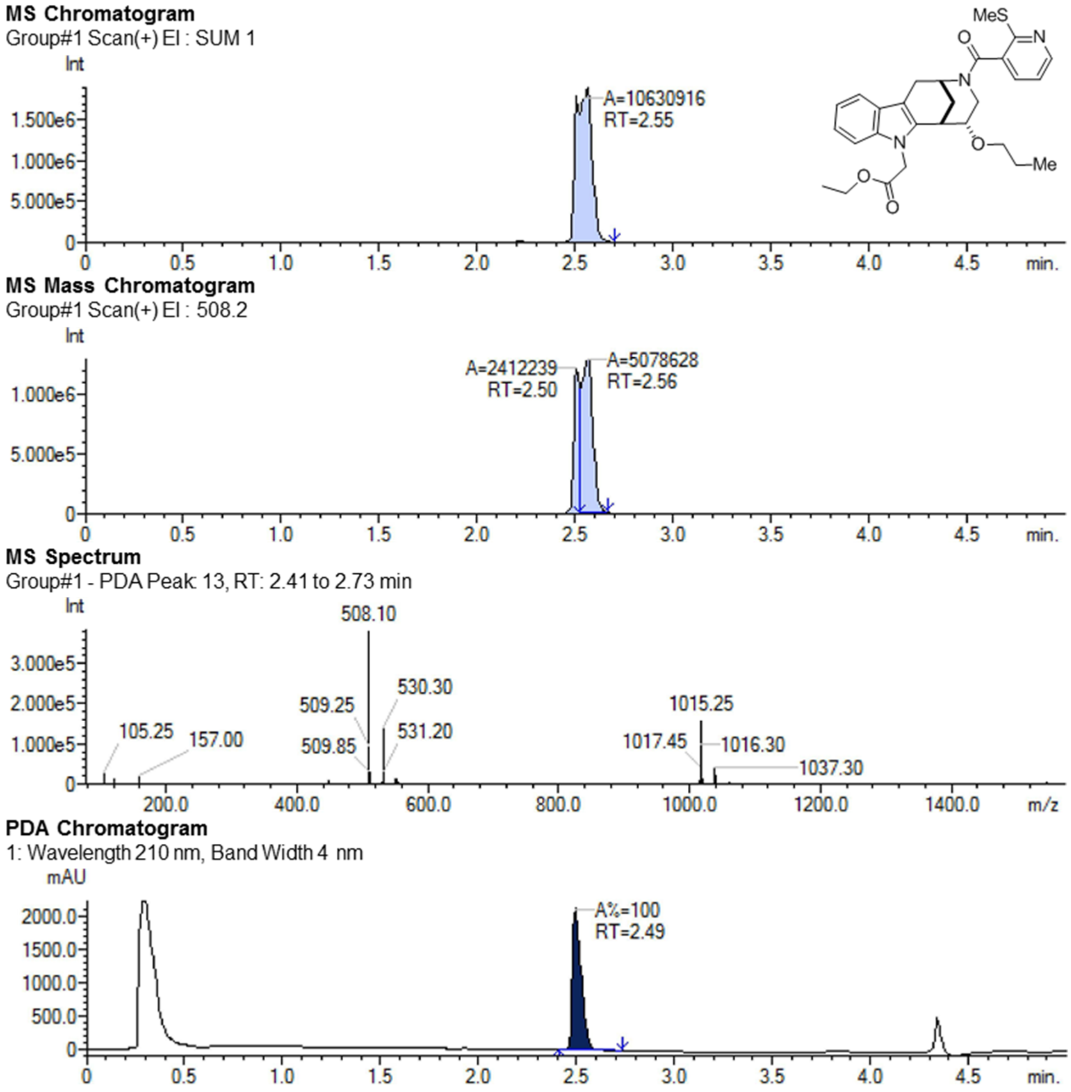
MS Chromatogram
Group#1 Scan(+) EI : SUM 1
Int
A=10630916
RT=2.55
min.
MeS
N
O
N
N
O
Me
O
O
MS Mass Chromatogram
Group#1 Scan(+) EI : 508.2
Int
A=2412239
RT=2.50
A=5078628
RT=2.56
min.
MS Spectrum
Group#1 - PDA Peak 13, RT: 2.41 to 2.73 min
Int
508.10
509.25
530.30
105.25
157.00
509.85
531.20
1015.25
1017.45
1016.30
1037.30
m/z
PDA Chromatogram
1: Wavelength 210 nm, Band Width 4 nm
mAU
A%=100
RT=2.49
min.

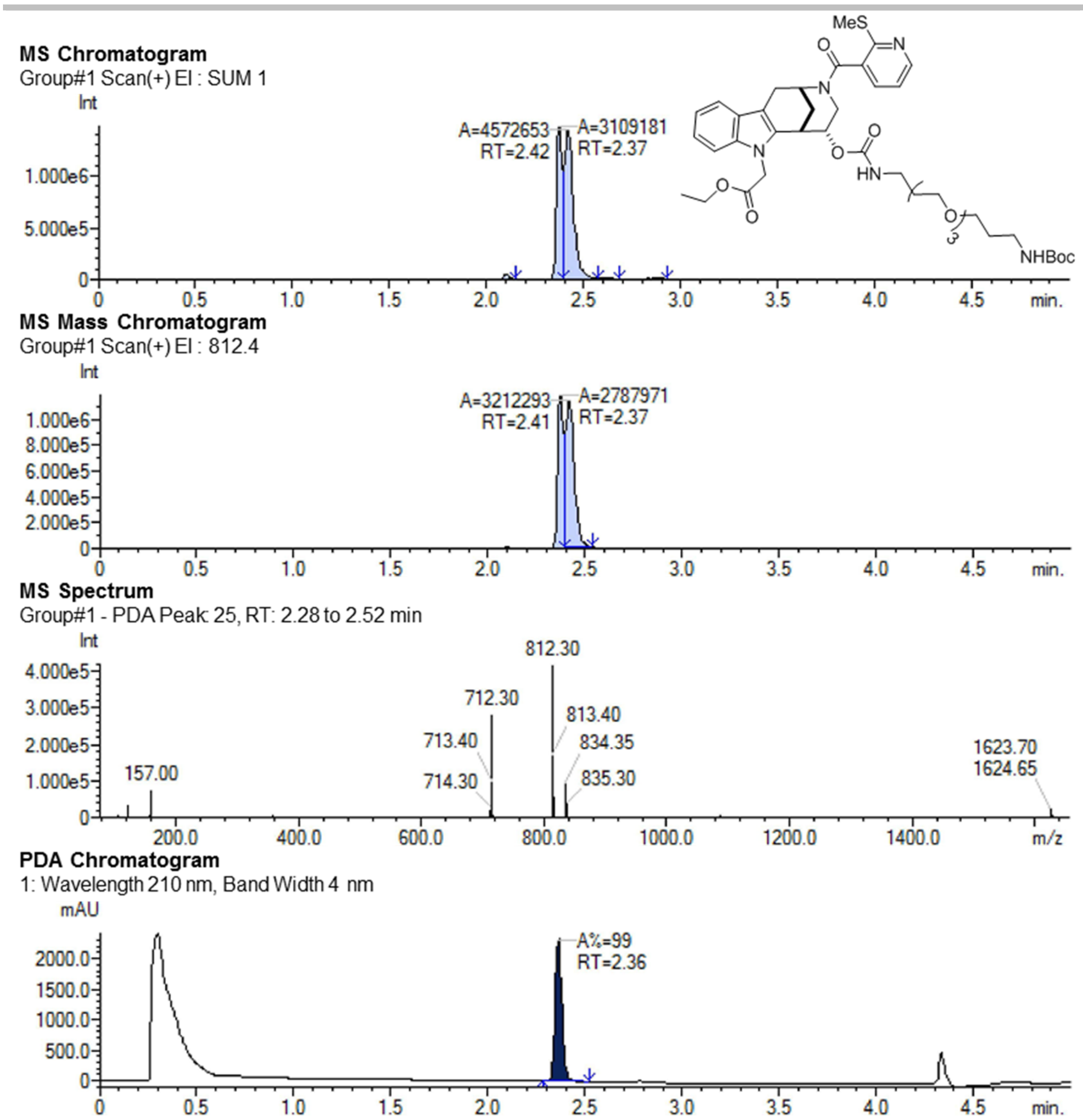
MS Chromatogram
Group#1 Scan(+) EI : SUM 1
Int
A=4572653
RT=2.42
A=3109181
RT=2.37
MeS
N
O
HN
NHBoc
MS Mass Chromatogram
Group#1 Scan(+) EI : 812.4
A=3212293
RT=2.41
A=2787971
RT=2.37
MS Spectrum
Group#1 - PDA Peak 25, RT: 2.28 to 2.52 min
812.30
712.30
813.40
713.40
834.35
714.30
835.30
157.00
1623.70
1624.65
m/z
PDA Chromatogram
1: Wavelength 210 nm, Band Width 4 nm
mAU
A%=99
RT=2.36
min.

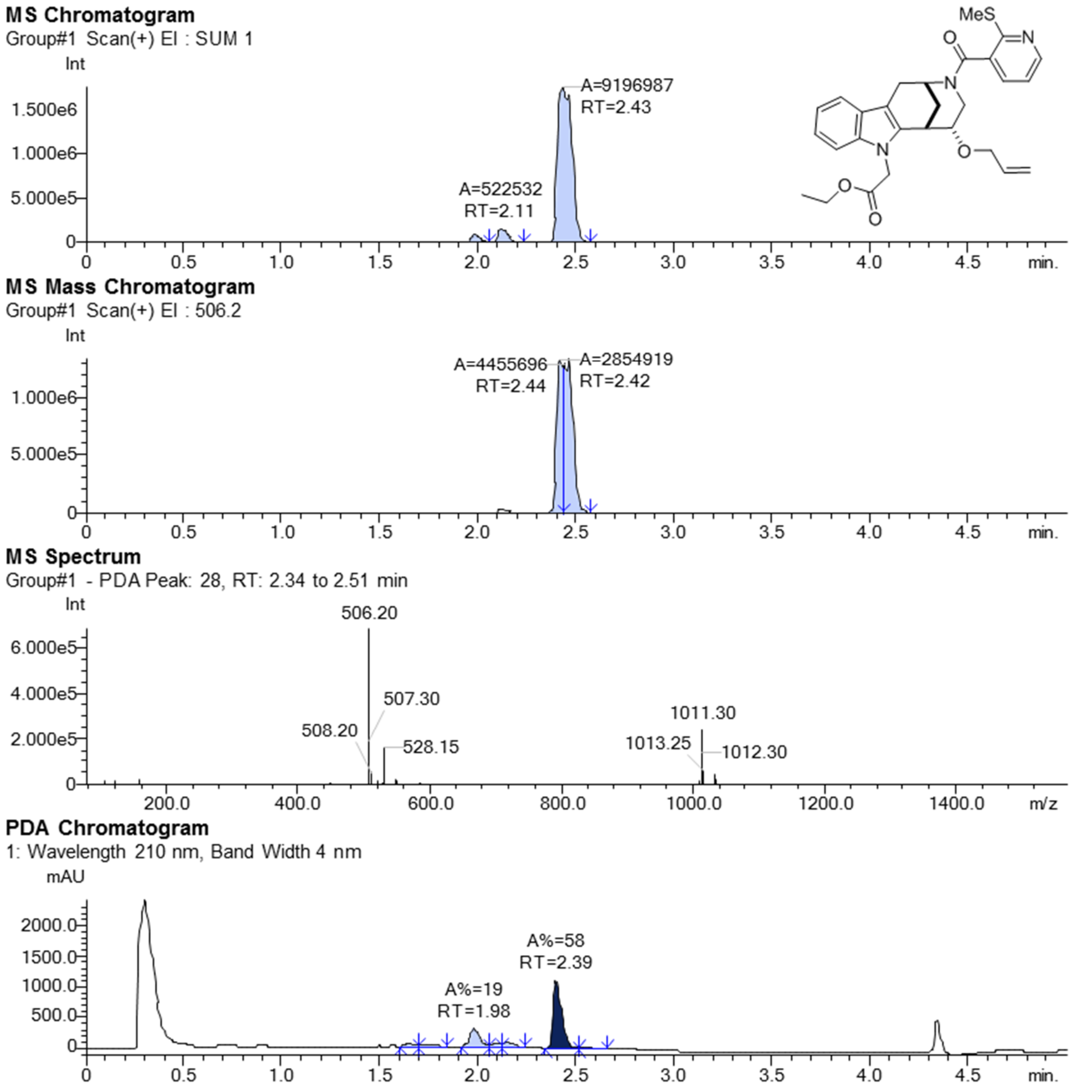
MS Chromatogram
Group#1 Scan(+) EI : SUM 1
Int
1.500e6
1.000e6
5.000e5
0
A=9196987
RT=2.43
A=522532
RT=2.11
0 0.5 1.0 1.5 2.0 2.5 3.0 3.5 4.0 4.5 min.
MeS
O
N
N
N
O
O
O
MS Mass Chromatogram
Group#1 Scan(+) EI : 506.2
Int
1.000e6
5.000e5
0
A=4455696
RT=2.44
A=2854919
RT=2.42
MS Spectrum
Group#1 - PDA Peak: 28, RT: 2.34 to 2.51 min
Int
6.000e5
4.000e5
2.000e5
506.20
507.30
508.20
528.15
1011.30
1013.25
1012.30
200.0 400.0 600.0 800.0 1000.0 1200.0 1400.0 m/z
PDA Chromatogram
1: Wavelength 210 nm, Band Width 4 nm
mAU
2000.0
1500.0
1000.0
500.0
0
A%=58
RT=2.39
A%=19
RT=1.98

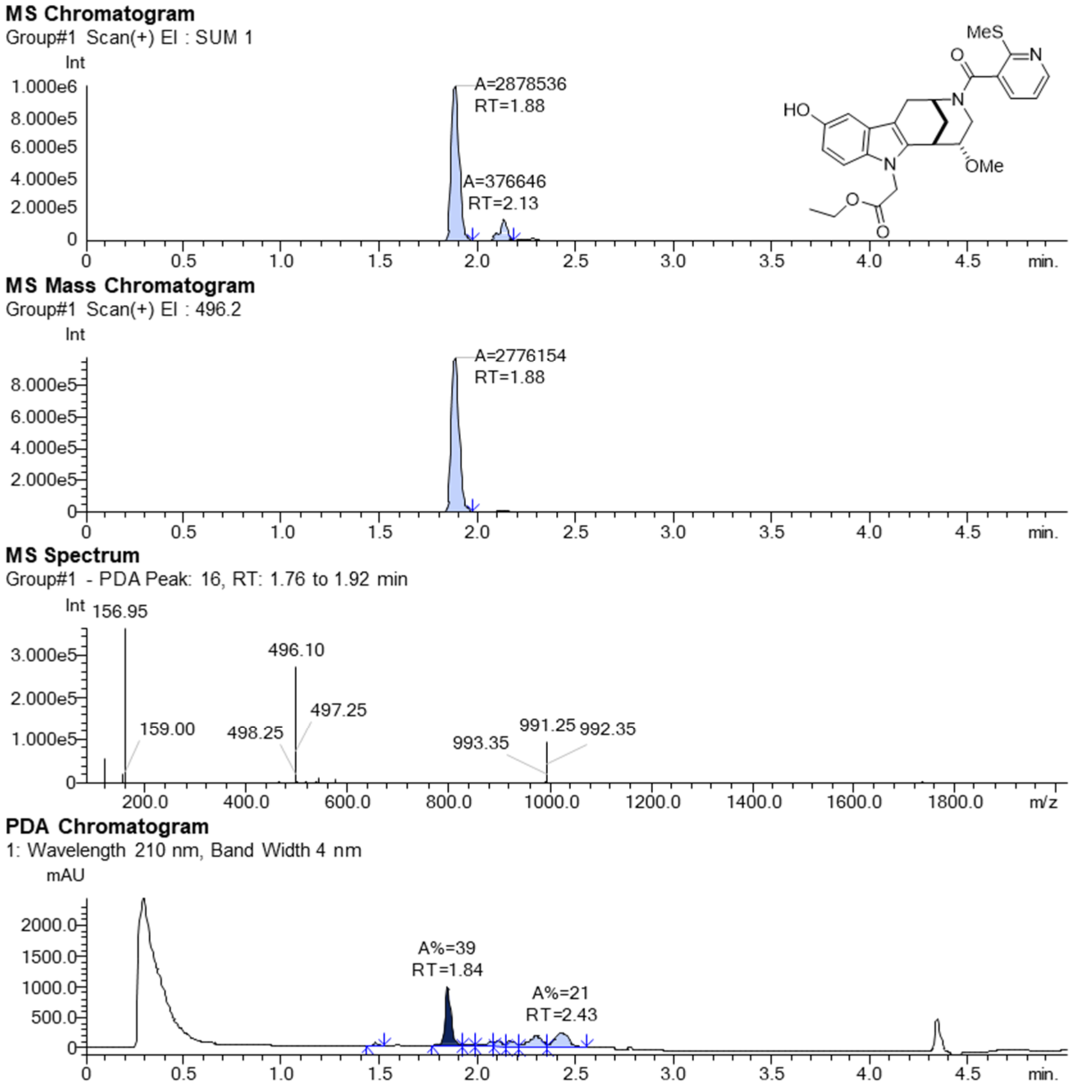
MS Chromatogram
Group#1 Scan(+) EI : SUM 1
Int
A=2878536
RT=1.88
A=376646
RT=2.13
MeS
N
O
HO
N
N
OMe
O
O
min.
MS Mass Chromatogram
Group#1 Scan(+) EI : 496.2
Int
A=2776154
RT=1.88
min.
MS Spectrum
Group#1 - PDA Peak: 16, RT: 1.76 to 1.92 min
Int
156.95
496.10
497.25
159.00
498.25
991.25
992.35
993.35
m/z
PDA Chromatogram
1: Wavelength 210 nm, Band Width 4 nm
mAU
A%=39
RT=1.84
A%=21
RT=2.43
min.

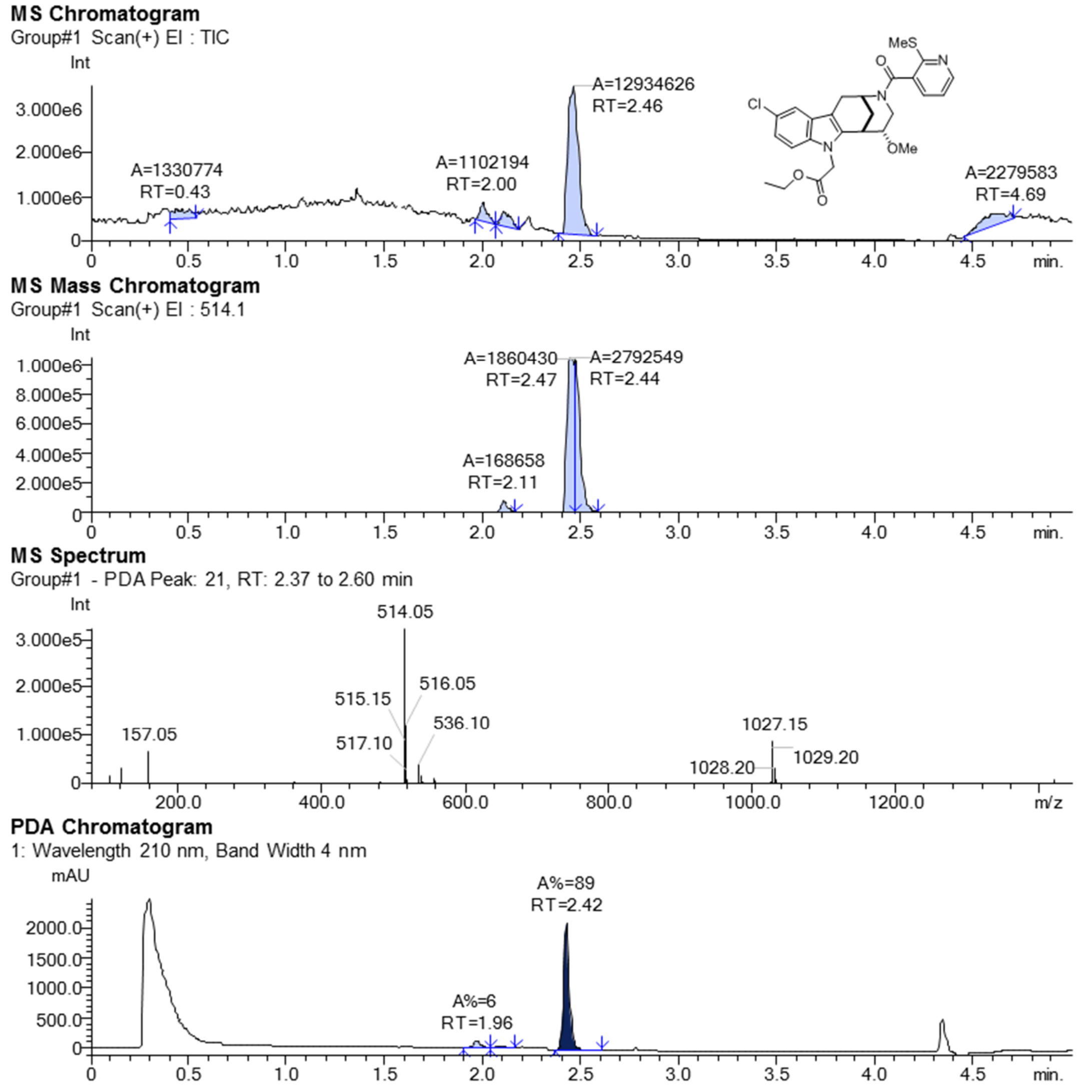
MS Chromatogram
Group#1 Scan(+) EI : TIC
Int
A=1330774
RT=0.43
A=1102194
RT=2.00
A=12934626
RT=2.46
A=2279583
RT=4.69
MeS
N
O
Cl
N
OMe
O
O
min.
MS Mass Chromatogram
Group#1 Scan(+) EI : 514.1
Int
A=1860430
RT=2.47
A=2792549
RT=2.44
A=168658
RT=2.11
min.
MS Spectrum
Group#1 - PDA Peak: 21, RT: 2.37 to 2.60 min
Int
514.05
515.15
516.05
536.10
517.10
157.05
1027.15
1029.20
1028.20
m/z
PDA Chromatogram
1: Wavelength 210 nm, Band Width 4 nm
mAU
A%=89
RT=2.42
A%=6
RT=1.96
min.

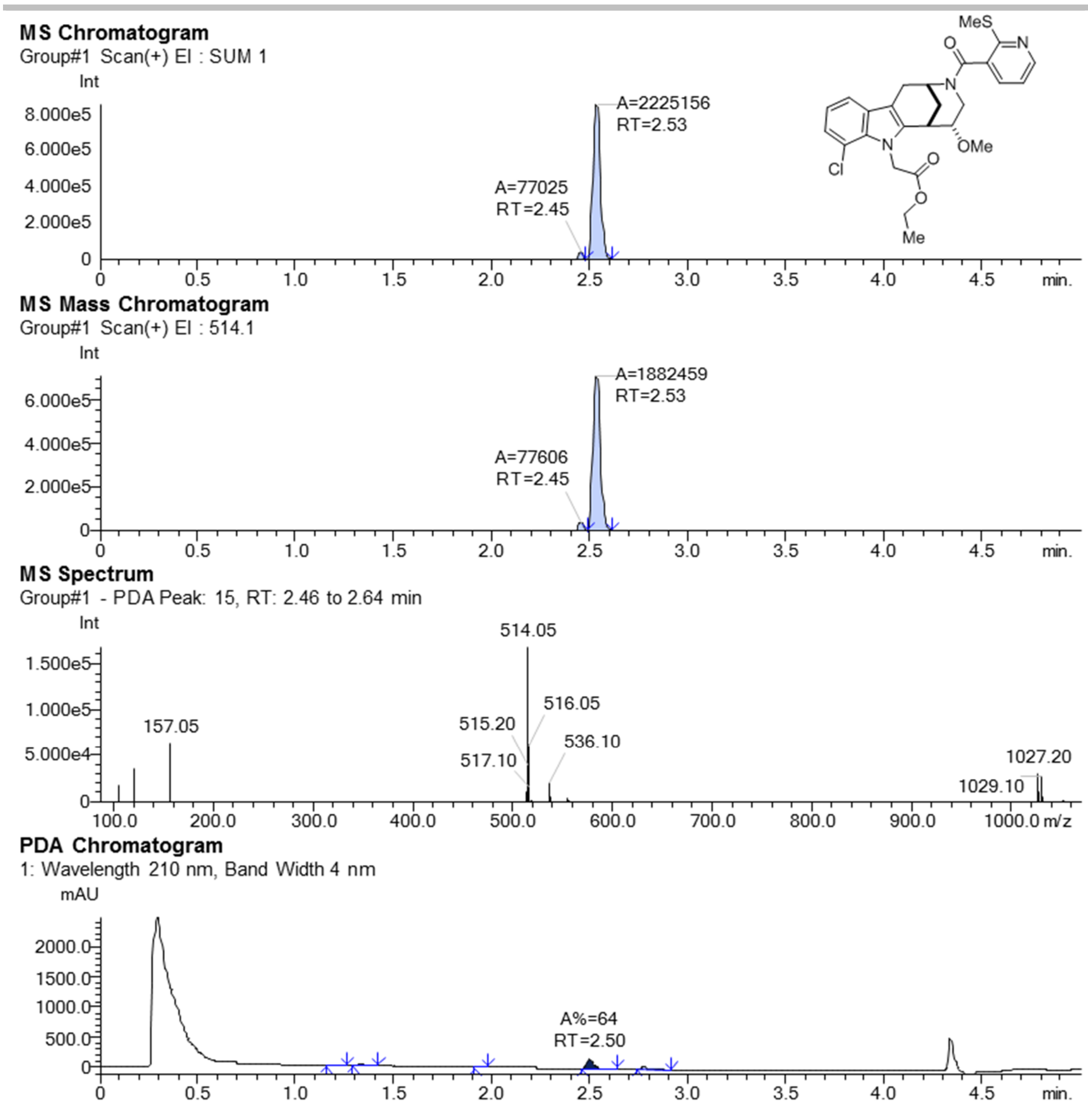
MS Chromatogram
Group#1 Scan(+) EI : SUM 1
Int
8.000e5
6.000e5
4.000e5
2.000e5
0
A=2225156
RT=2.53
A=77025
RT=2.45
0 0.5 1.0 1.5 2.0 2.5 3.0 3.5 4.0 4.5 min.
MeS
N
O
N
OMe
Cl
N
O
O
Me
MS Mass Chromatogram
Group#1 Scan(+) EI : 514.1
Int
6.000e5
4.000e5
2.000e5
0
A=1882459
RT=2.53
A=77606
RT=2.45
0 0.5 1.0 1.5 2.0 2.5 3.0 3.5 4.0 4.5 min.
MS Spectrum
Group#1 - PDA Peak: 15, RT: 2.46 to 2.64 min
Int
1.500e5
1.000e5
5.000e4
0
514.05
516.05
157.05
515.20
536.10
517.10
1027.20
1029.10
100.0 200.0 300.0 400.0 500.0 600.0 700.0 800.0 900.0 1000.0 m/z
PDA Chromatogram
1: Wavelength 210 nm, Band Width 4 nm
mAU
2000.0
1500.0
1000.0
500.0
0
A%=64
RT=2.50
0 0.5 1.0 1.5 2.0 2.5 3.0 3.5 4.0 4.5 min.

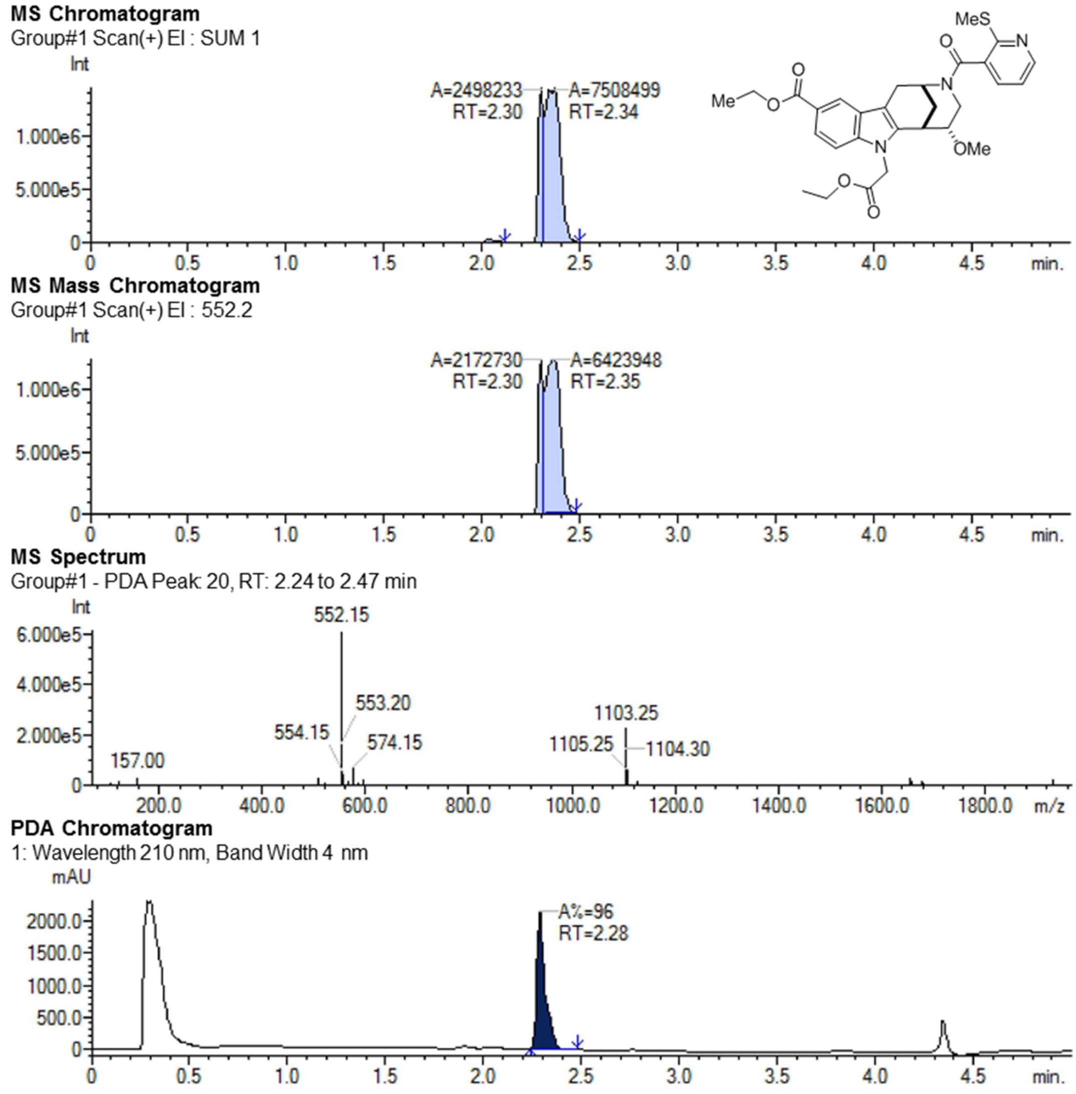
MS Chromatogram
Group#1 Scan(+) EI : SUM 1
Int
A=2498233
RT=2.30
A=7508499
RT=2.34
1.000e6
5.000e5
0
0.5
1.0
1.5
2.0
2.5
3.0
3.5
4.0
4.5
min.
MeS
N
O
O
Me
O
N
N
OMe
O
O
MS Mass Chromatogram
Group#1 Scan(+) EI : 552.2
Int
A=2172730
RT=2.30
A=6423948
RT=2.35
1.000e6
5.000e5
MS Spectrum
Group#1 - PDA Peak 20, RT: 2.24 to 2.47 min
Int
552.15
6.000e5
4.000e5
2.000e5
553.20
554.15
574.15
157.00
1103.25
1105.25
1104.30
200.0
400.0
600.0
800.0
1000.0
1200.0
1400.0
1600.0
1800.0
m/z
PDA Chromatogram
1: Wavelength 210 nm, Band Width 4 nm
mAU
A%=96
RT=2.28
2000.0
1500.0
1000.0
500.0

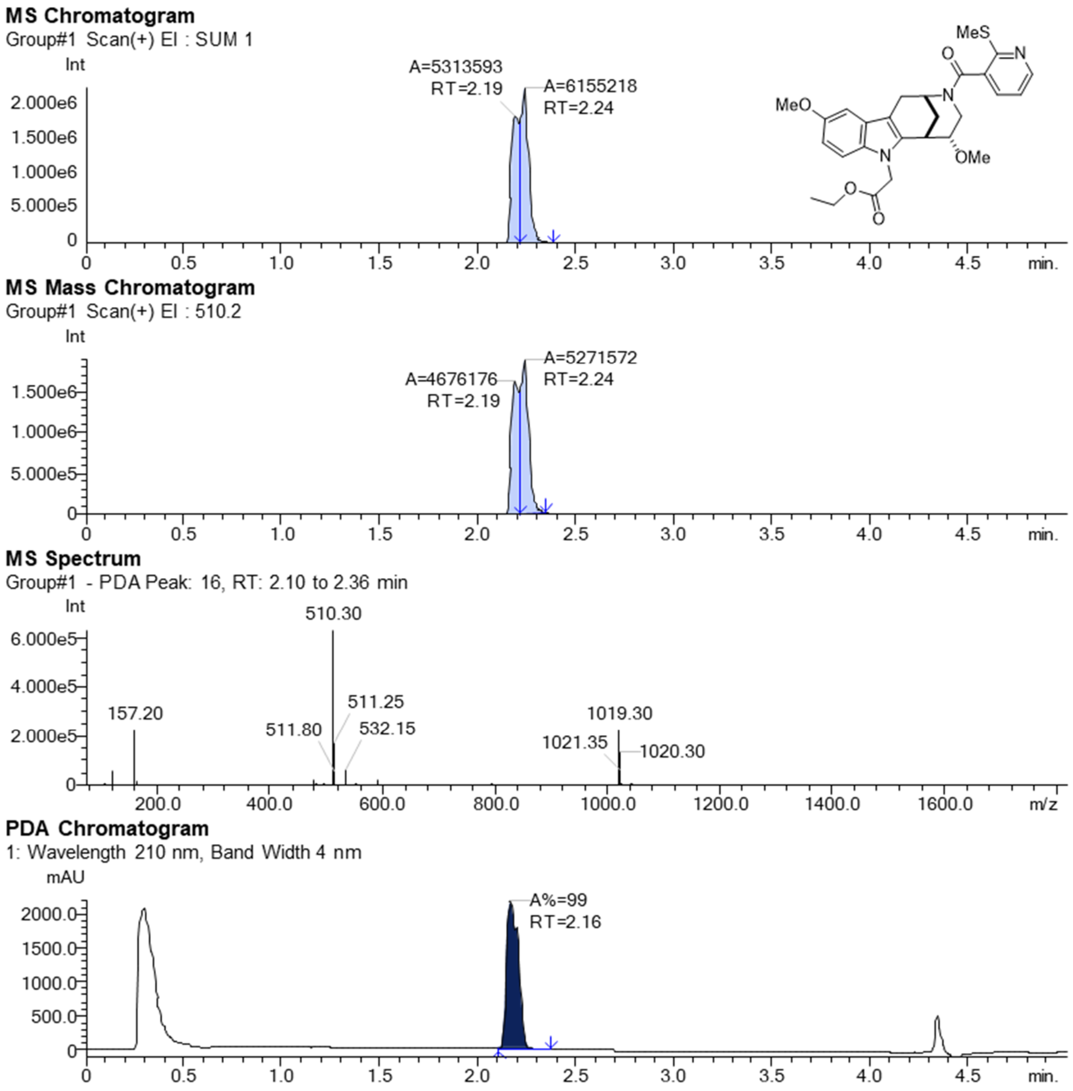
MS Chromatogram
Group#1 Scan(+) EI : SUM 1
Int
A=5313593
RT=2.19
A=6155218
RT=2.24
2.000e6
1.500e6
1.000e6
5.000e5
0
0 0.5 1.0 1.5 2.0 2.5 3.0 3.5 4.0 4.5 min.
MeS
N
O
MeO
N
N
OMe
O
O
MS Mass Chromatogram
Group#1 Scan(+) EI : 510.2
Int
A=4676176
RT=2.19
A=5271572
RT=2.24
1.500e6
1.000e6
5.000e5
0
0 0.5 1.0 1.5 2.0 2.5 3.0 3.5 4.0 4.5 min.
MS Spectrum
Group#1 - PDA Peak: 16, RT: 2.10 to 2.36 min
Int
510.30
6.000e5
4.000e5
2.000e5
0
157.20
511.80
511.25
532.15
1019.30
1021.35
1020.30
200.0 400.0 600.0 800.0 1000.0 1200.0 1400.0 1600.0 m/z
PDA Chromatogram
1: Wavelength 210 nm, Band Width 4 nm
mAU
2000.0
1500.0
1000.0
500.0
0
A%=99
RT=2.16
0 0.5 1.0 1.5 2.0 2.5 3.0 3.5 4.0 4.5 min.

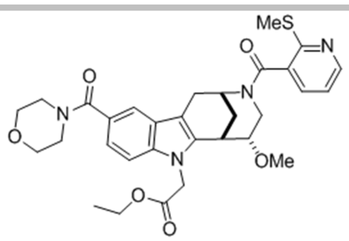

MS Chromatogram
Group#1 Scan(+) EI : SUM 1

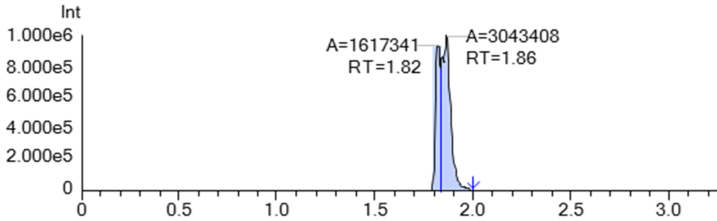

MS Mass Chromatogram
Group#1 Scan(+) EI : 593.2

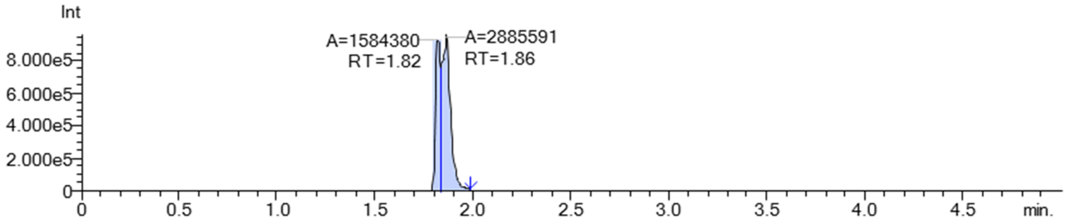

MS Spectrum
Group#1 - PDA Peak: 16, RT: 1.73 to 1.91 min

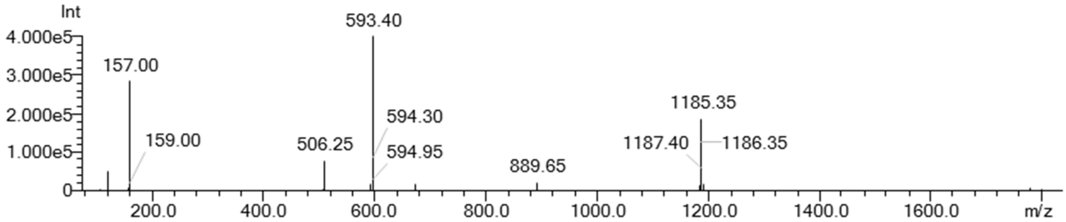

PDA Chromatogram
1: Wavelength 210 nm, Band Width 4 nm

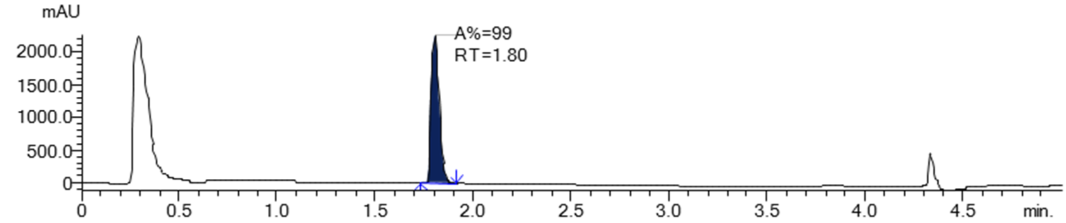

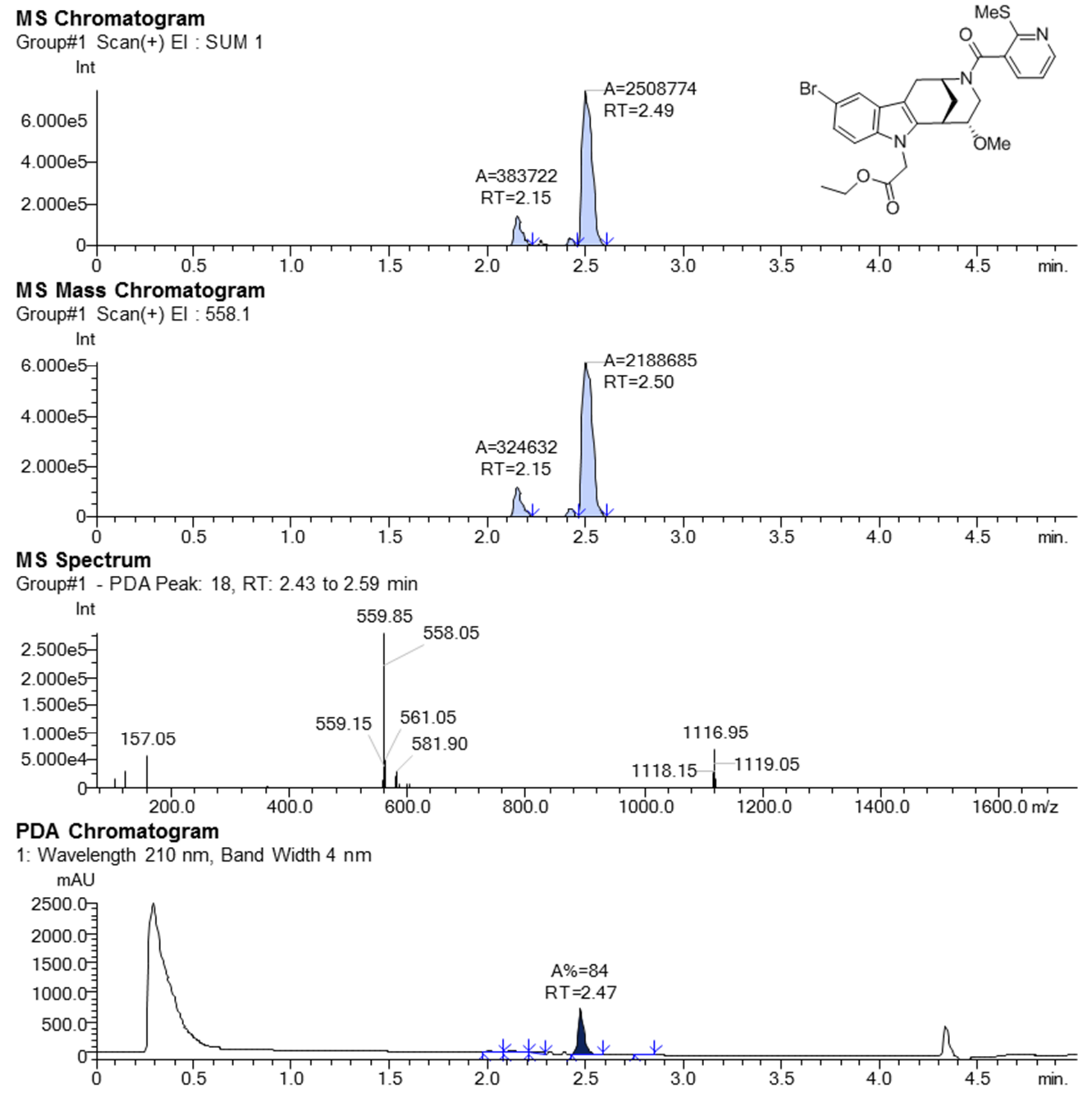
MS Chromatogram
Group#1 Scan(+) EI : SUM 1
Int
6.000e5
4.000e5
2.000e5
0
A=2508774
RT=2.49
A=383722
RT=2.15
0
0.5
1.0
1.5
2.0
2.5
3.0
3.5
4.0
4.5
min.
MeS
O
N
Br
N
N
OMe
O
O
MS Mass Chromatogram
Group#1 Scan(+) EI : 558.1
Int
6.000e5
4.000e5
2.000e5
0
A=2188685
RT=2.50
A=324632
RT=2.15
MS Spectrum
Group#1 - PDA Peak: 18, RT: 2.43 to 2.59 min
Int
2.500e5
2.000e5
1.500e5
1.000e5
5.000e4
0
559.85
558.05
157.05
559.15
561.05
581.90
1116.95
1118.15
1119.05
200.0
400.0
600.0
800.0
1000.0
1200.0
1400.0
1600.0 m/z
PDA Chromatogram
1: Wavelength 210 nm, Band Width 4 nm
mAU
2500.0
2000.0
1500.0
1000.0
500.0
0
A%=84
RT=2.47

MS Chromatogram
Group#1 Scan(+) EI : SUM 1

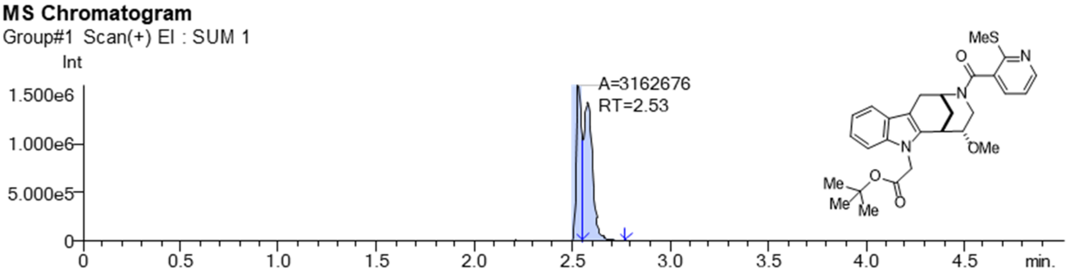

MS Mass Chromatogram
Group#1 Scan(+) EI : 508.2

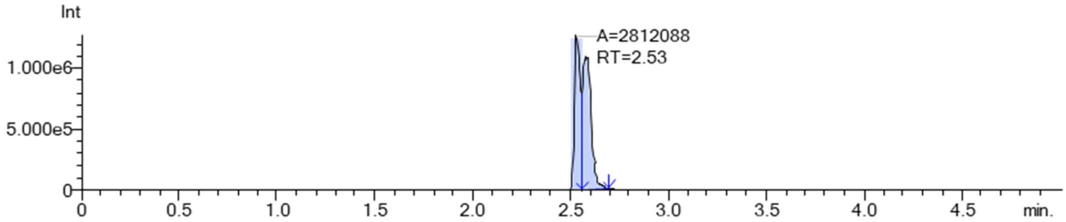

MS Spectrum
Group#1 - PDA Peak: 16, RT: 2.47 to 2.64 min

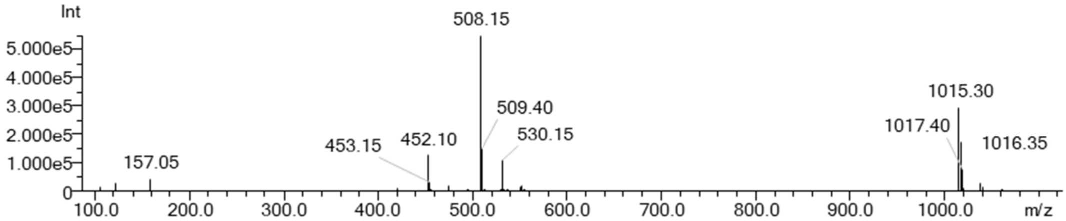

PDA Chromatogram
1: Wavelength 210 nm, Band Width 4 nm

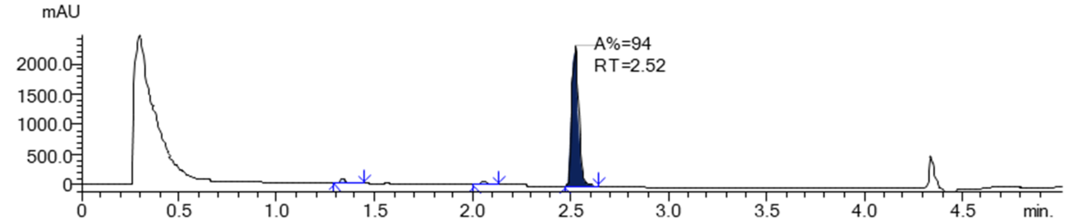

MS Chromatogram
Group#1 Scan(+) EI : SUM 1

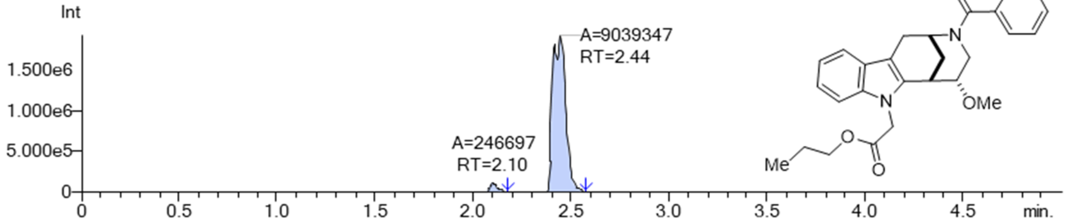

MS Mass Chromatogram
Group#1 Scan(+) EI : 494.2

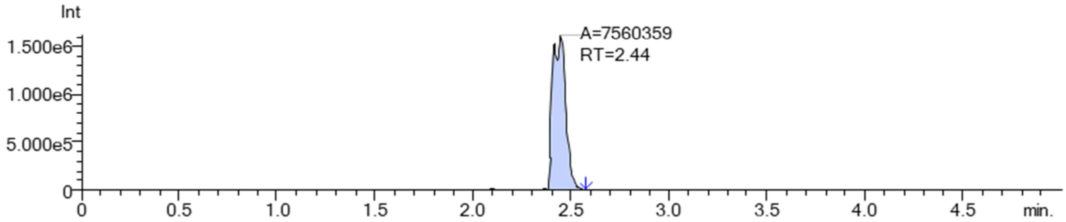

MS Spectrum
Group#1 - PDA Peak: 19, RT: 2.31 to 2.52 min

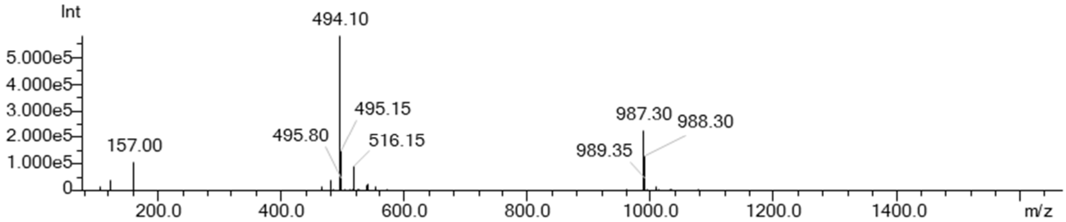

PDA Chromatogram
1: Wavelength 210 nm, Band Width 4 nm

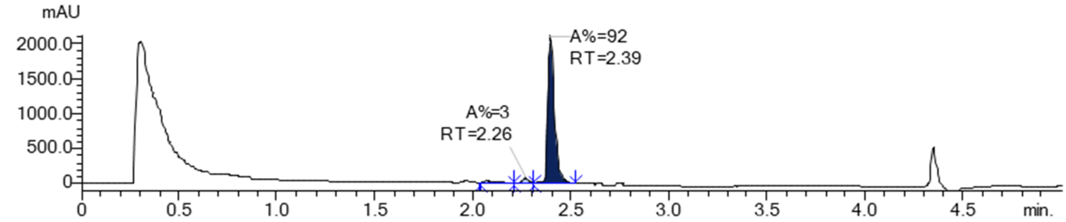

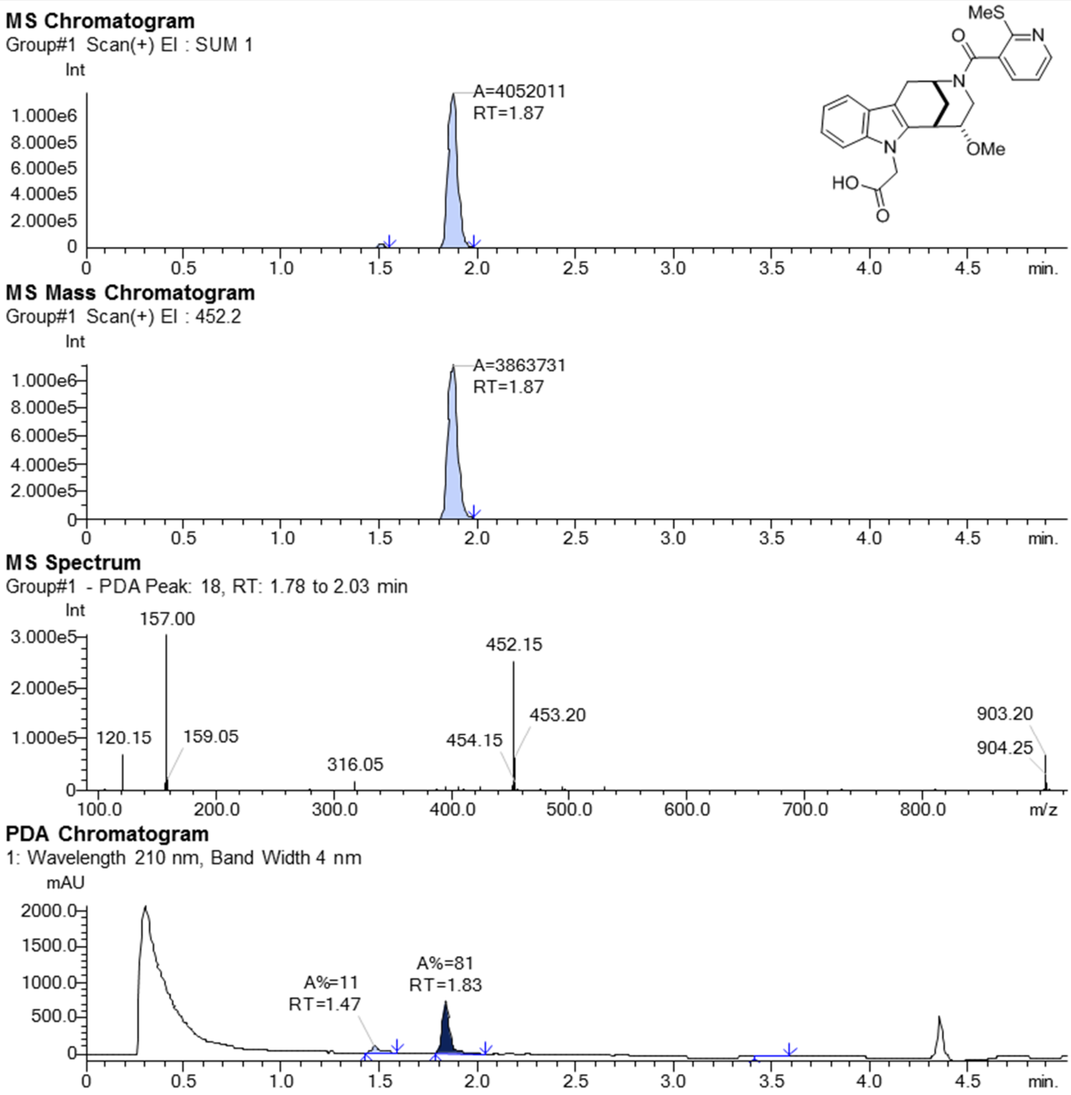
MS Chromatogram
Group#1 Scan(+) EI : SUM 1
Int
A=4052011
RT=1.87
MeS
N
O
N
OMe
HO
O
min.
MS Mass Chromatogram
Group#1 Scan(+) EI : 452.2
A=3863731
RT=1.87
MS Spectrum
Group#1 - PDA Peak: 18, RT: 1.78 to 2.03 min
157.00
452.15
453.20
903.20
904.25
120.15
159.05
454.15
316.05
m/z
PDA Chromatogram
1: Wavelength 210 nm, Band Width 4 nm
mAU
A%=11
RT=1.47
A%=81
RT=1.83

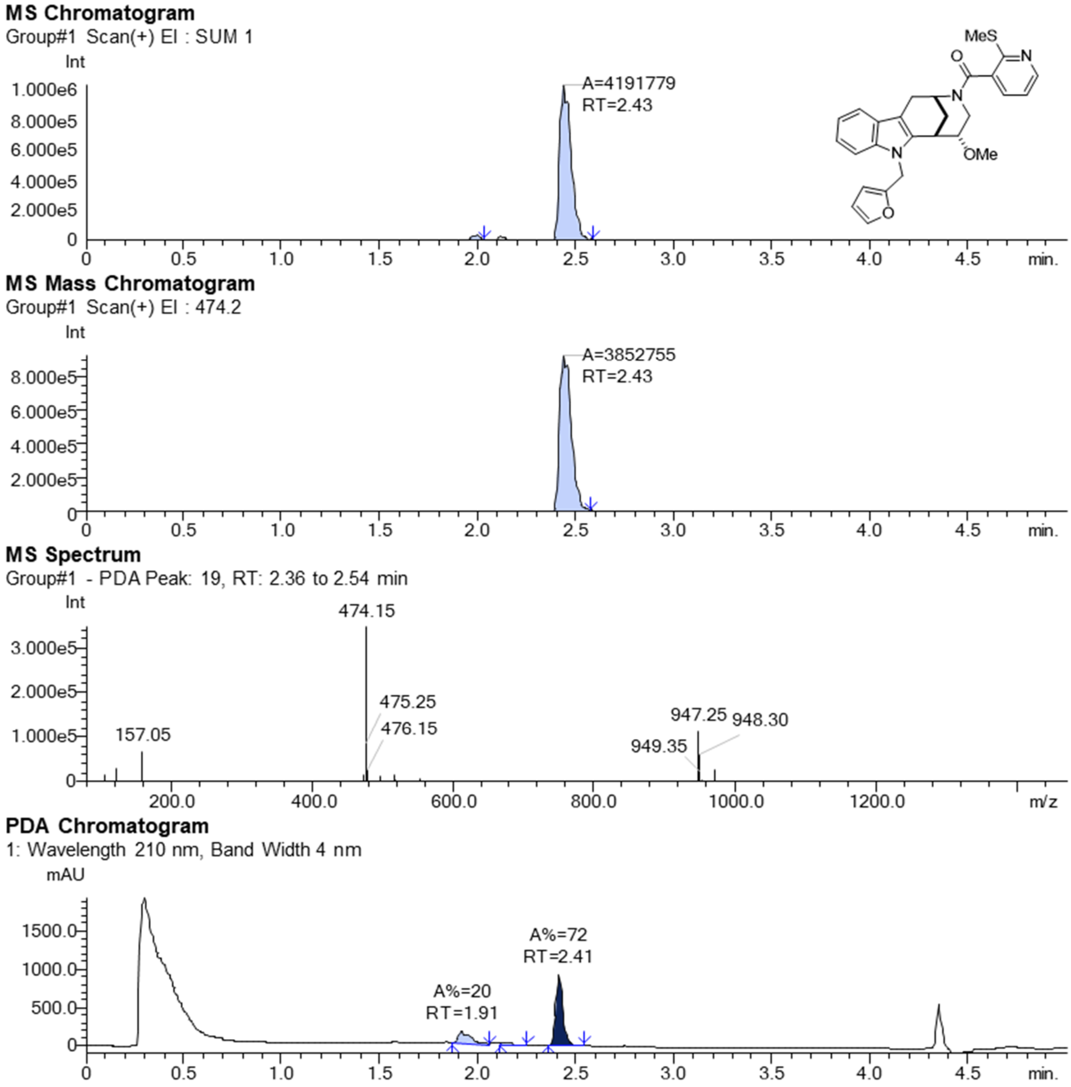
MS Chromatogram
Group#1 Scan(+) EI : SUM 1
Int
A=4191779
RT=2.43
min.
MeS
O
N
N
N
OMe
O
MS Mass Chromatogram
Group#1 Scan(+) EI : 474.2
A=3852755
RT=2.43
MS Spectrum
Group#1 - PDA Peak: 19, RT: 2.36 to 2.54 min
474.15
475.25
476.15
157.05
947.25
948.30
949.35
m/z
PDA Chromatogram
1: Wavelength 210 nm, Band Width 4 nm
mAU
A%=72
RT=2.41
A%=20
RT=1.91

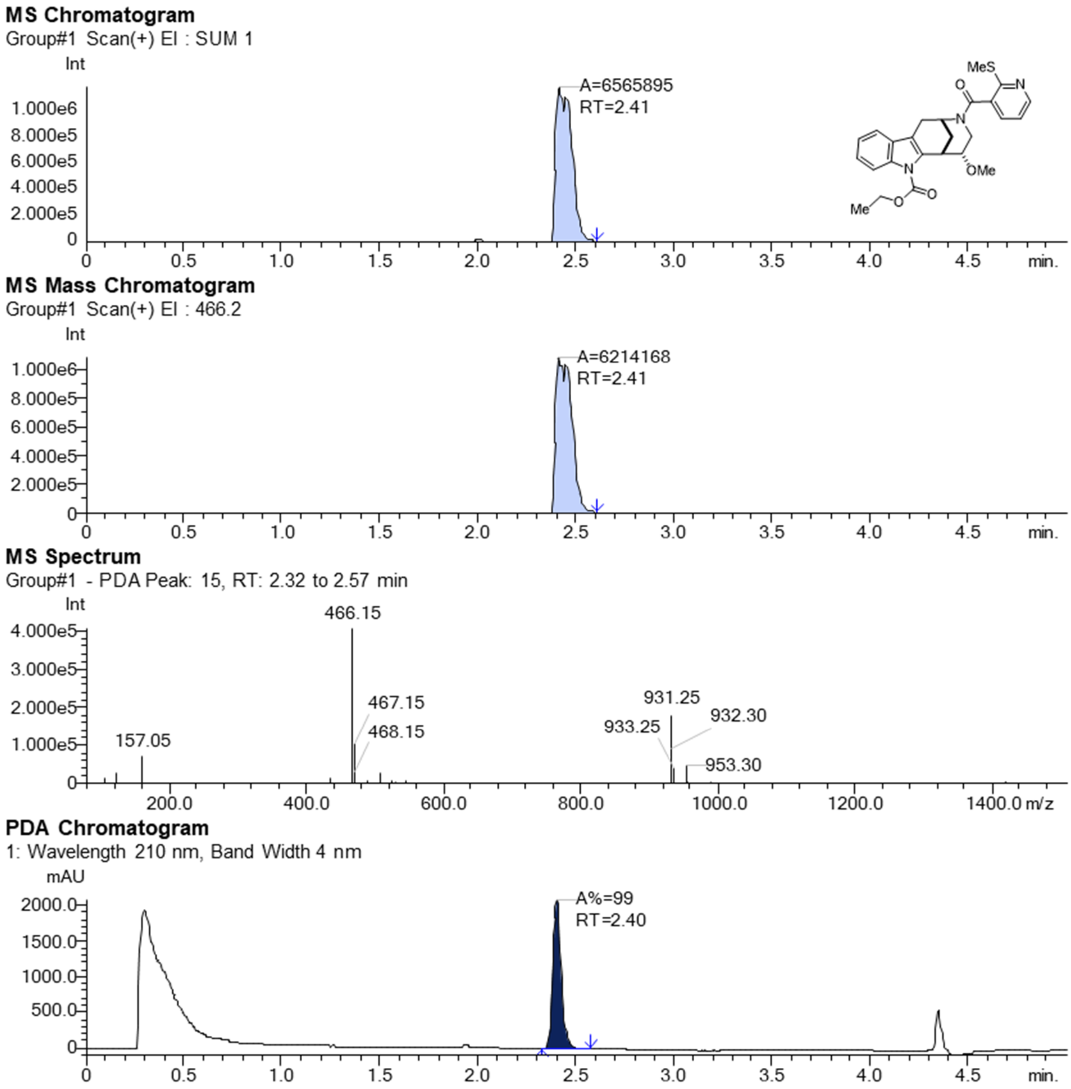
MS Chromatogram
Group#1 Scan(+) EI : SUM 1
A=6565895
RT=2.41
MeS
O
N
OMe
Me
O
MS Mass Chromatogram
Group#1 Scan(+) EI : 466.2
A=6214168
RT=2.41
MS Spectrum
Group#1 - PDA Peak: 15, RT: 2.32 to 2.57 min
466.15
467.15
468.15
157.05
931.25
932.30
933.25
953.30
PDA Chromatogram
1: Wavelength 210 nm, Band Width 4 nm
A%=99
RT=2.40

MS Chromatogram
Group#1 Scan(+) EI : SUM 1

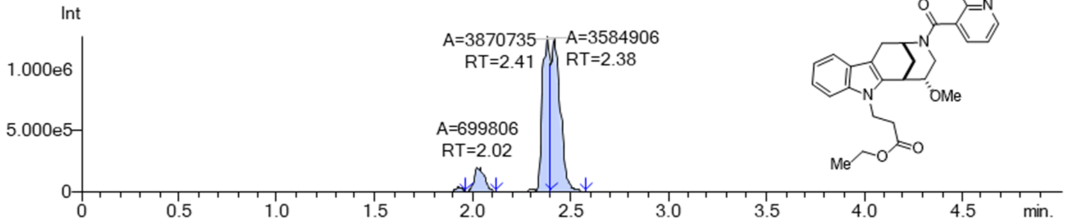

MS Mass Chromatogram
Group#1 Scan(+) EI : 494.2

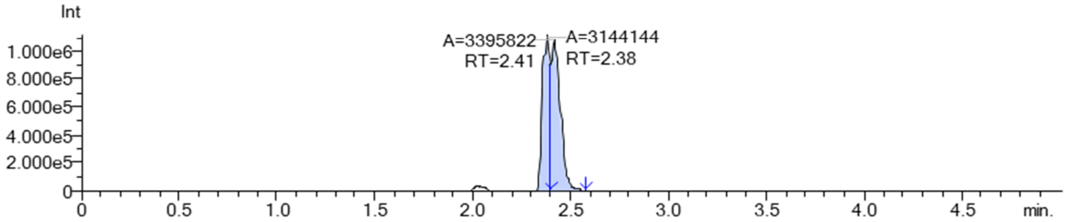

MS Spectrum
Group#1 - PDA Peak: 25, RT: 2.30 to 2.52 min

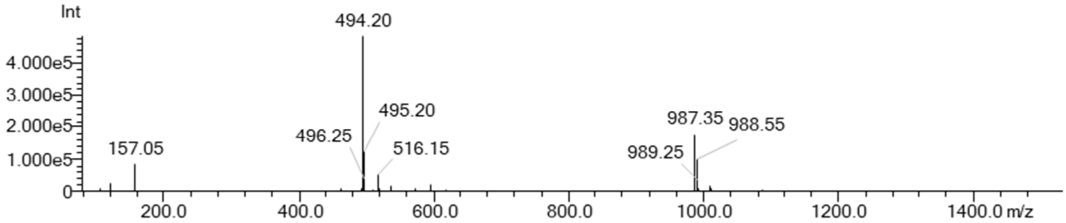

PDA Chromatogram
1: Wavelength 210 nm, Band Width 4 nm

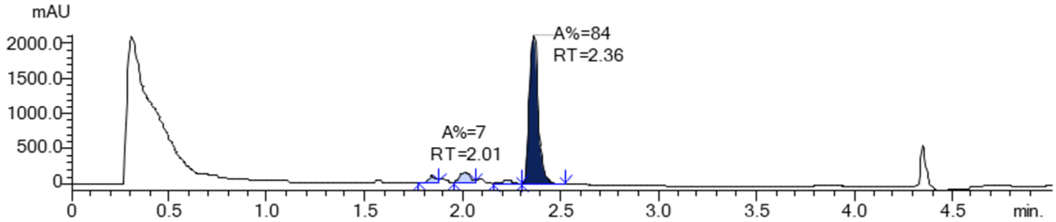

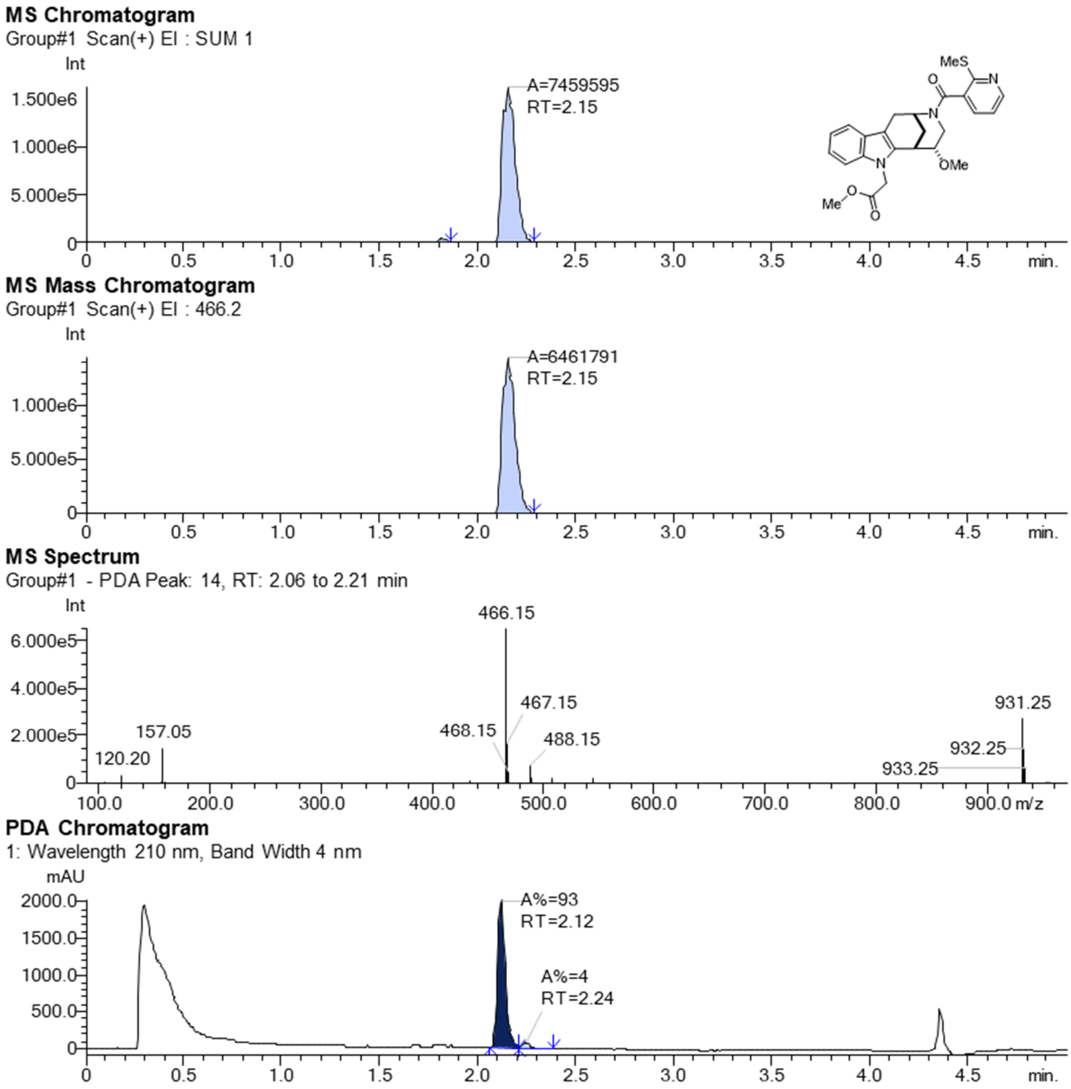
MS Chromatogram
Group#1 Scan(+) EI : SUM 1
A=7459595
RT=2.15
MeS
MS Mass Chromatogram
Group#1 Scan(+) EI : 466.2
A=6461791
RT=2.15
MS Spectrum
Group#1 - PDA Peak: 14, RT: 2.06 to 2.21 min
466.15
467.15
468.15
488.15
157.05
120.20
931.25
932.25
933.25
PDA Chromatogram
1: Wavelength 210 nm, Band Width 4 nm
A%=93
RT=2.12
A%=4
RT=2.24

MS Chromatogram

Group#1 Scan(+) EI : SUM 1

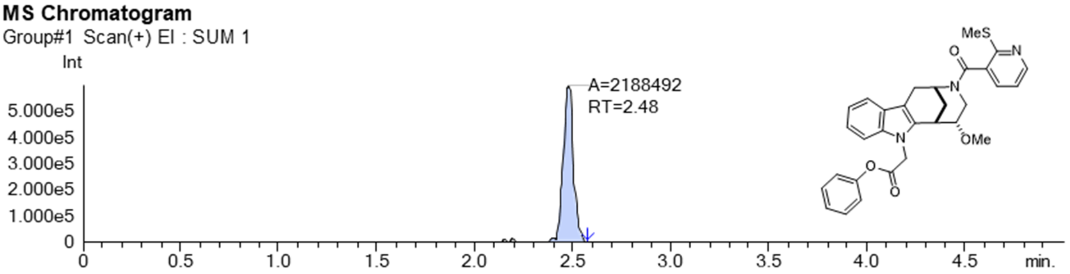

MS Mass Chromatogram

Group#1 Scan(+) EI : 528.2

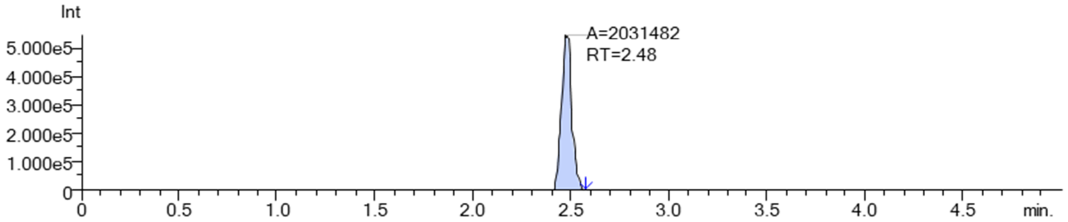

MS Spectrum

Group#1 - PDA Peak: 20, RT: 2.35 to 2.52 min

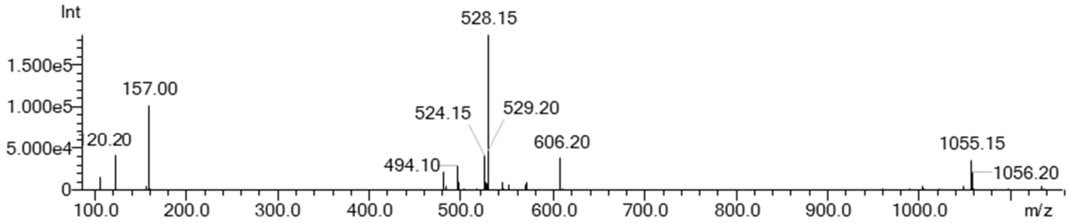

PDA Chromatogram

1: Wavelength 210 nm, Band Width 4 nm

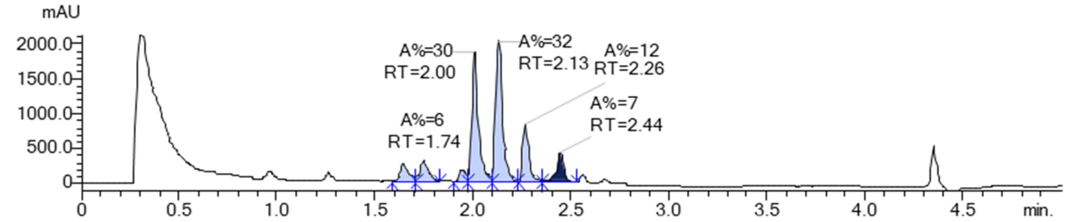

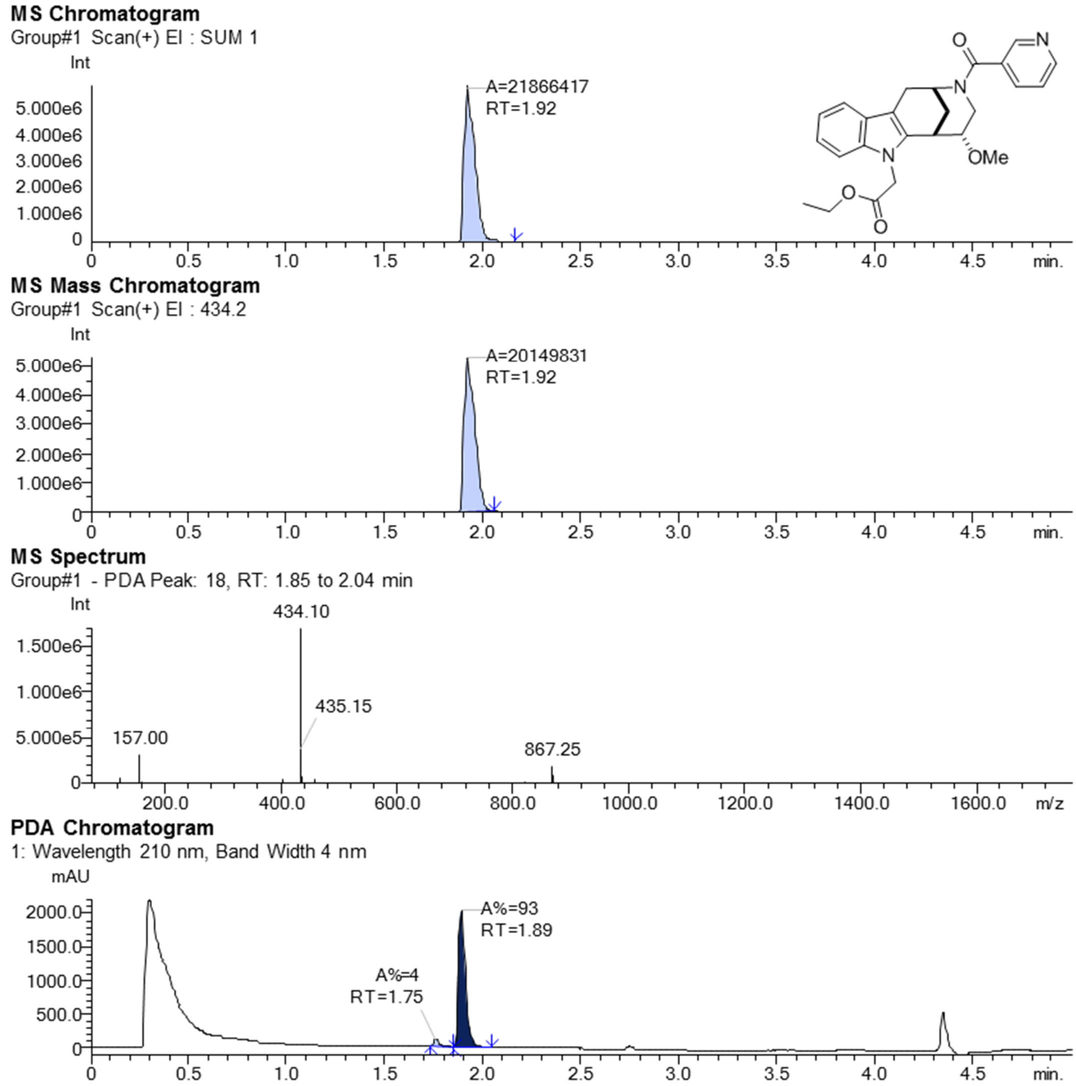
MS Chromatogram
Group#1 Scan(+) EI : SUM 1
A=21866417
RT=1.92
OMe
MS Mass Chromatogram
Group#1 Scan(+) EI : 434.2
A=20149831
RT=1.92
MS Spectrum
Group#1 - PDA Peak: 18, RT: 1.85 to 2.04 min
434.10
435.15
157.00
867.25
PDA Chromatogram
1: Wavelength 210 nm, Band Width 4 nm
A%=93
RT=1.89
A%=4
RT=1.75

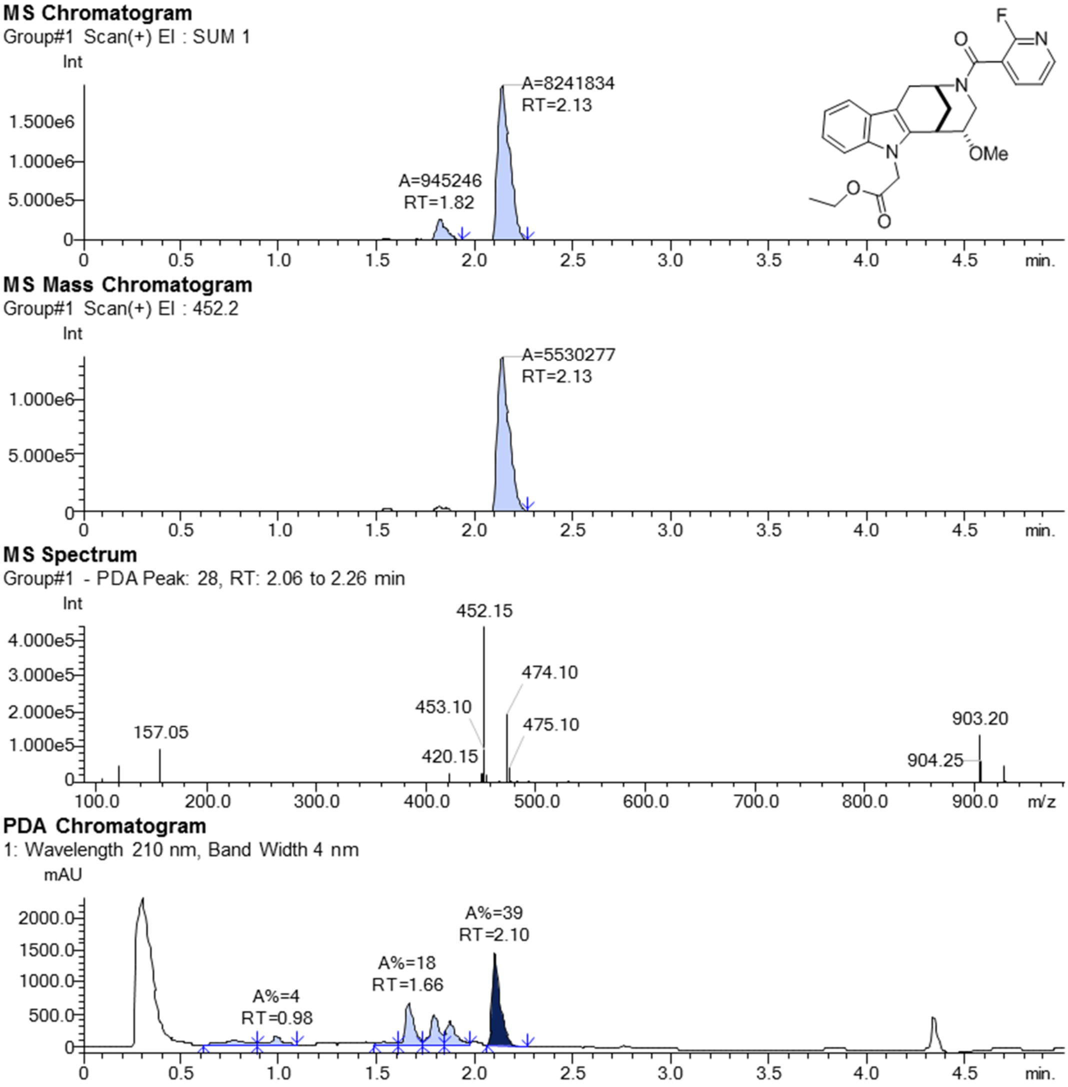
MS Chromatogram
Group#1 Scan(+) EI : SUM 1
A=8241834
RT=2.13
A=945246
RT=1.82
MS Mass Chromatogram
Group#1 Scan(+) EI : 452.2
A=5530277
RT=2.13
MS Spectrum
Group#1 - PDA Peak: 28, RT: 2.06 to 2.26 min
452.15
474.10
453.10
475.10
420.15
157.05
903.20
904.25
PDA Chromatogram
1: Wavelength 210 nm, Band Width 4 nm
A%=39
RT=2.10
A%=18
RT=1.66
A%=4
RT=0.98
F
N
O
OMe

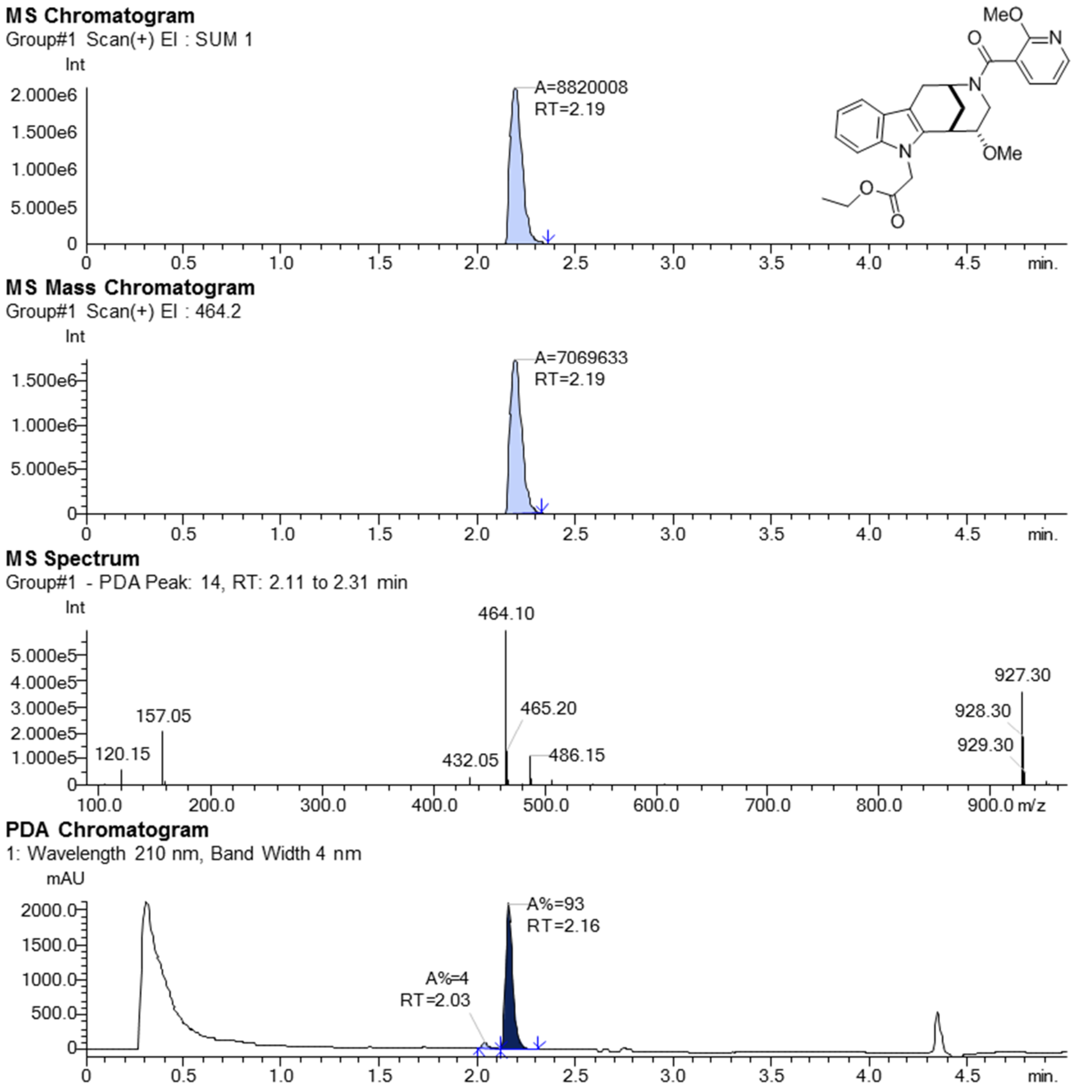
MS Chromatogram
Group#1 Scan(+) EI : SUM 1
Int
2.000e6
1.500e6
1.000e6
5.000e5
0
A=8820008
RT=2.19
0
0.5
1.0
1.5
2.0
2.5
3.0
3.5
4.0
4.5
min.
MeO
O
N
N
OMe
N
O
O
MS Mass Chromatogram
Group#1 Scan(+) EI : 464.2
Int
1.500e6
1.000e6
5.000e5
A=7069633
RT=2.19
MS Spectrum
Group#1 - PDA Peak: 14, RT: 2.11 to 2.31 min
Int
5.000e5
4.000e5
3.000e5
2.000e5
1.000e5
464.10
157.05
120.15
465.20
432.05
486.15
927.30
928.30
929.30
100.0
200.0
300.0
400.0
500.0
600.0
700.0
800.0
900.0 m/z
PDA Chromatogram
1: Wavelength 210 nm, Band Width 4 nm
mAU
2000.0
1500.0
1000.0
500.0
A%=93
RT=2.16
A%=4
RT=2.03

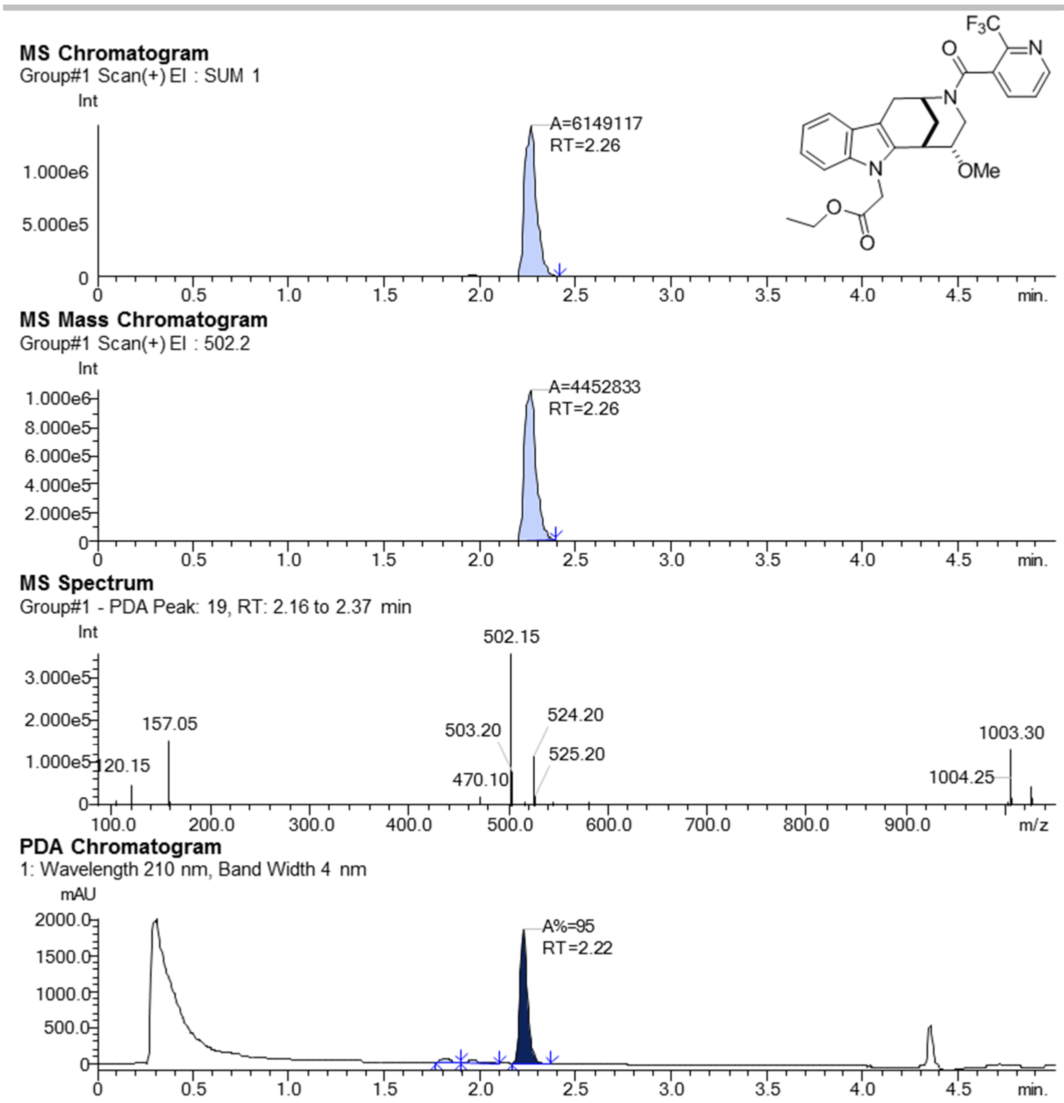
MS Chromatogram
Group#1 Scan(+) EI : SUM 1
Int
1.000e6
5.000e5
0
A=6149117
RT=2.26
0 0.5 1.0 1.5 2.0 2.5 3.0 3.5 4.0 4.5 min.
F3C
O
N
N
N
OMe
O
O
MS Mass Chromatogram
Group#1 Scan(+) EI : 502.2
Int
1.000e6
8.000e5
6.000e5
4.000e5
2.000e5
0
A=4452833
RT=2.26
0 0.5 1.0 1.5 2.0 2.5 3.0 3.5 4.0 4.5 min.
MS Spectrum
Group#1 - PDA Peak: 19, RT: 2.16 to 2.37 min
Int
3.000e5
2.000e5
1.000e5
0
502.15
157.05
120.15
503.20
470.10
524.20
525.20
1003.30
1004.25
100.0 200.0 300.0 400.0 500.0 600.0 700.0 800.0 900.0 m/z
PDA Chromatogram
1: Wavelength 210 nm, Band Width 4 nm
mAU
2000.0
1500.0
1000.0
500.0
0
A%=95
RT=2.22
0 0.5 1.0 1.5 2.0 2.5 3.0 3.5 4.0 4.5 min.

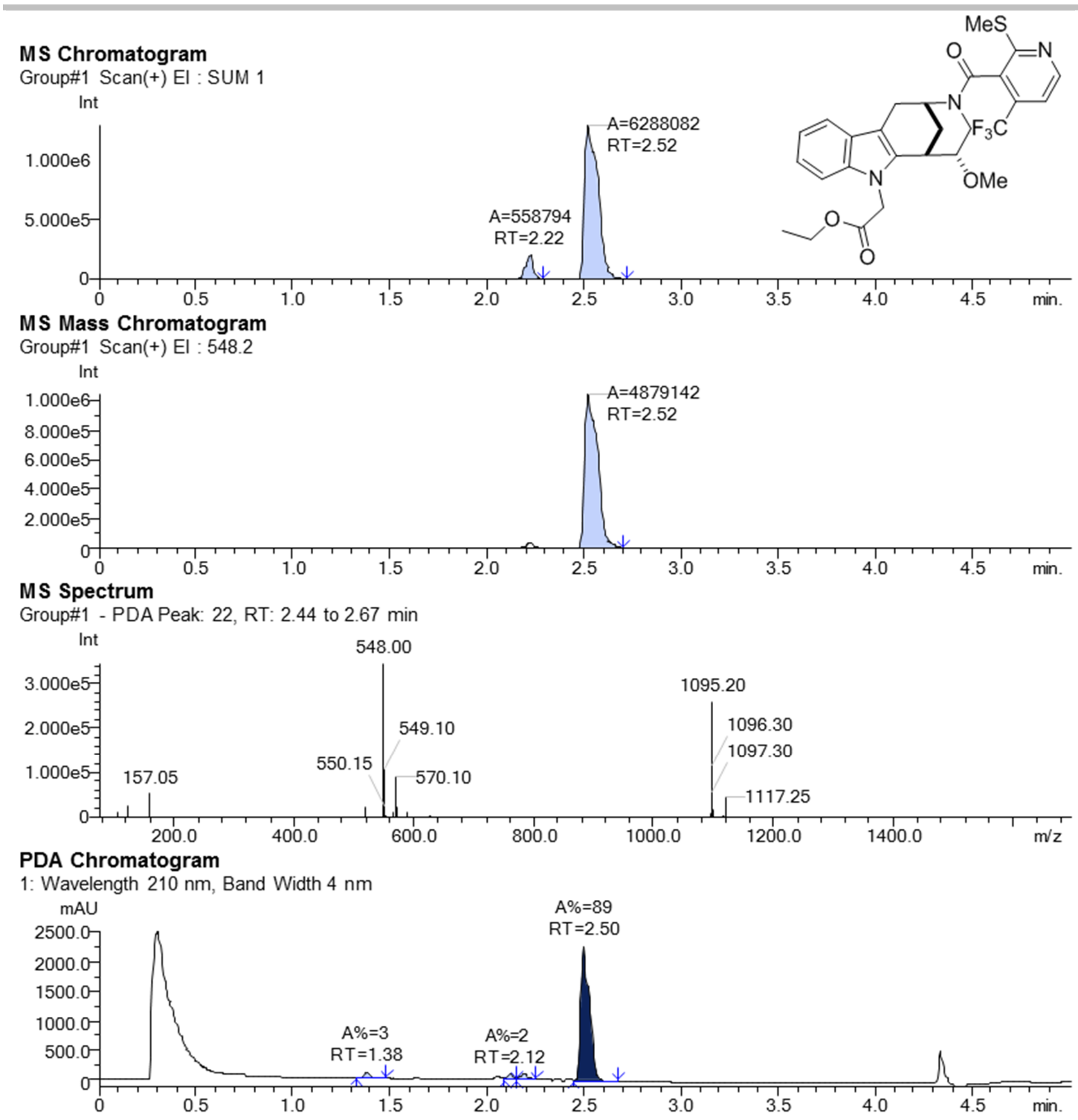
MS Chromatogram
Group#1 Scan(+) EI : SUM 1
A=6288082
RT=2.52
A=558794
RT=2.22
MeS
N
O
N
F3C
OMe
N
O
O
MS Mass Chromatogram
Group#1 Scan(+) EI : 548.2
A=4879142
RT=2.52
MS Spectrum
Group#1 - PDA Peak: 22, RT: 2.44 to 2.67 min
548.00
549.10
550.15
570.10
157.05
1095.20
1096.30
1097.30
1117.25
PDA Chromatogram
1: Wavelength 210 nm, Band Width 4 nm
A%=89
RT=2.50
A%=3
RT=1.38
A%=2
RT=2.12

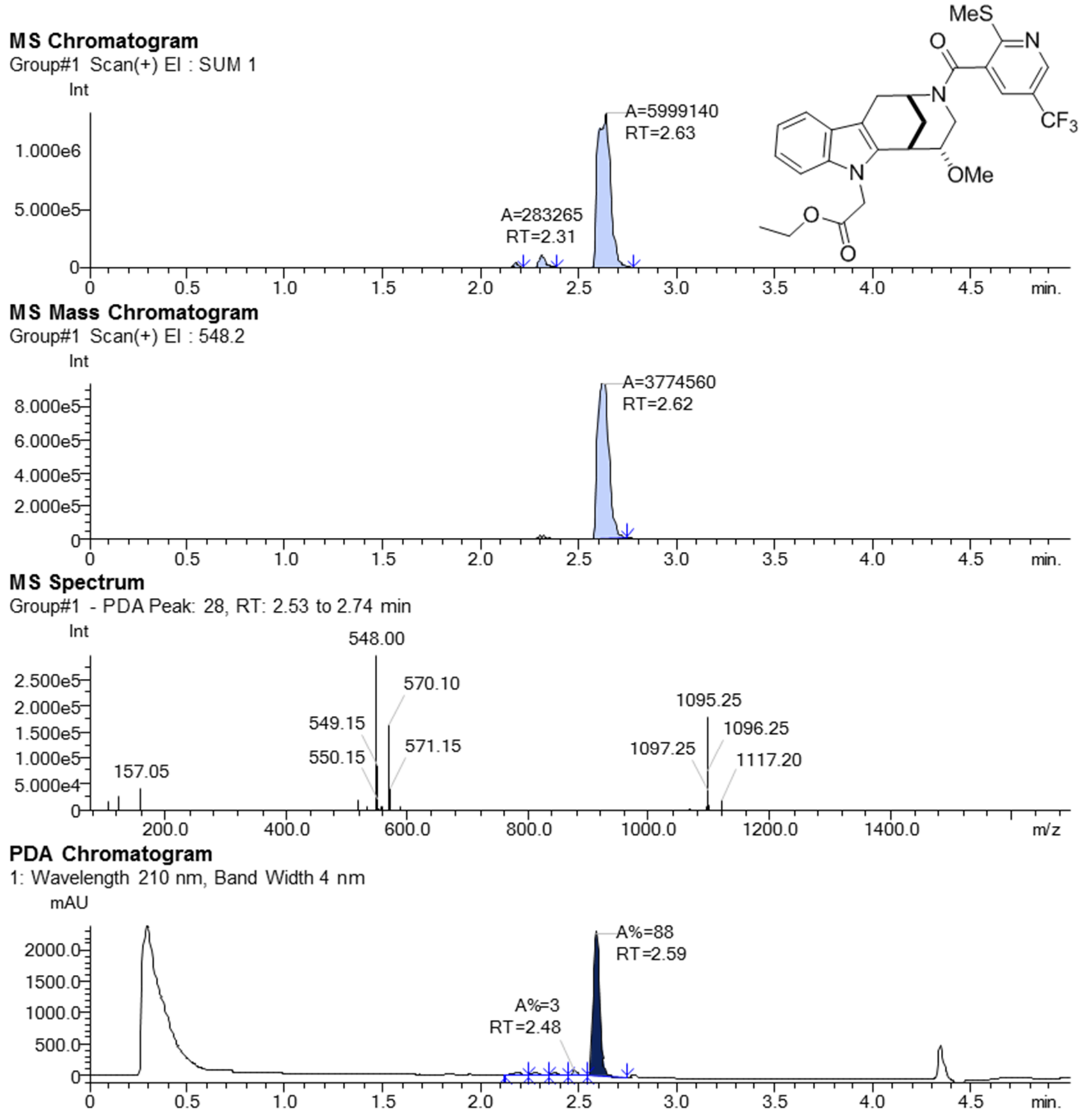
MS Chromatogram
Group#1 Scan(+) EI : SUM 1
Int
1.000e6
5.000e5
0
A=5999140
RT=2.63
A=283265
RT=2.31
0 0.5 1.0 1.5 2.0 2.5 3.0 3.5 4.0 4.5 min.
MeS
N
O
N
CF3
N
OMe
O
O
MS Mass Chromatogram
Group#1 Scan(+) EI : 548.2
Int
8.000e5
6.000e5
4.000e5
2.000e5
0
A=3774560
RT=2.62
0 0.5 1.0 1.5 2.0 2.5 3.0 3.5 4.0 4.5 min.
MS Spectrum
Group#1 - PDA Peak: 28, RT: 2.53 to 2.74 min
Int
2.500e5
2.000e5
1.500e5
1.000e5
5.000e4
0
548.00
570.10
549.15
550.15
571.15
157.05
1095.25
1096.25
1097.25
1117.20
200.0 400.0 600.0 800.0 1000.0 1200.0 1400.0 m/z
PDA Chromatogram
1: Wavelength 210 nm, Band Width 4 nm
mAU
2000.0
1500.0
1000.0
500.0
0
A%=88
RT=2.59
A%=3
RT=2.48
0 0.5 1.0 1.5 2.0 2.5 3.0 3.5 4.0 4.5 min.

MS Chromatogram
Group#1 Scan(+) EI : SUM 1

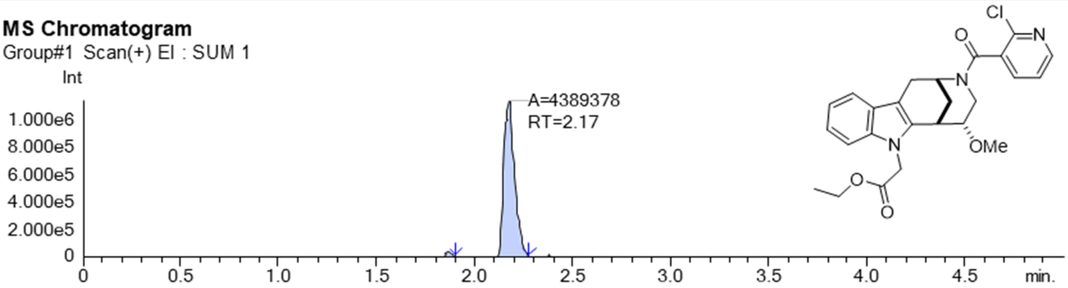

MS Mass Chromatogram
Group#1 Scan(+) EI : 468.2

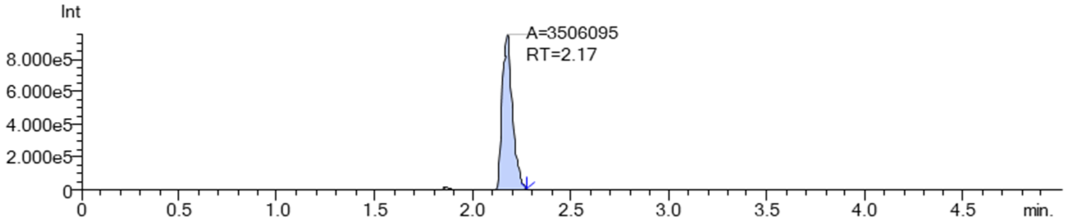

MS Spectrum
Group#1 - PDA Peak: 19, RT: 2.09 to 2.23 min

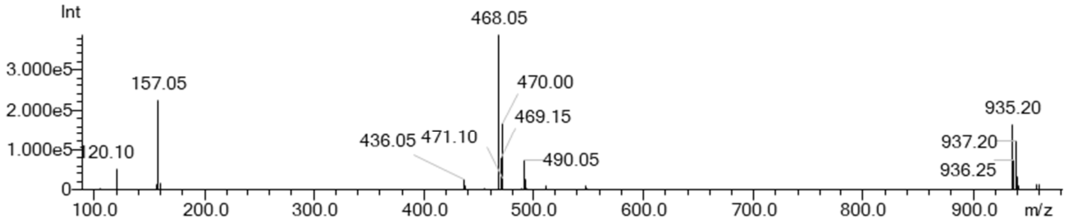

PDA Chromatogram
1: Wavelength 210 nm, Band Width 4 nm

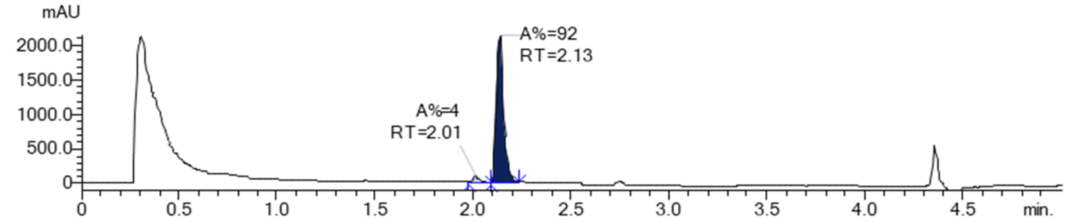

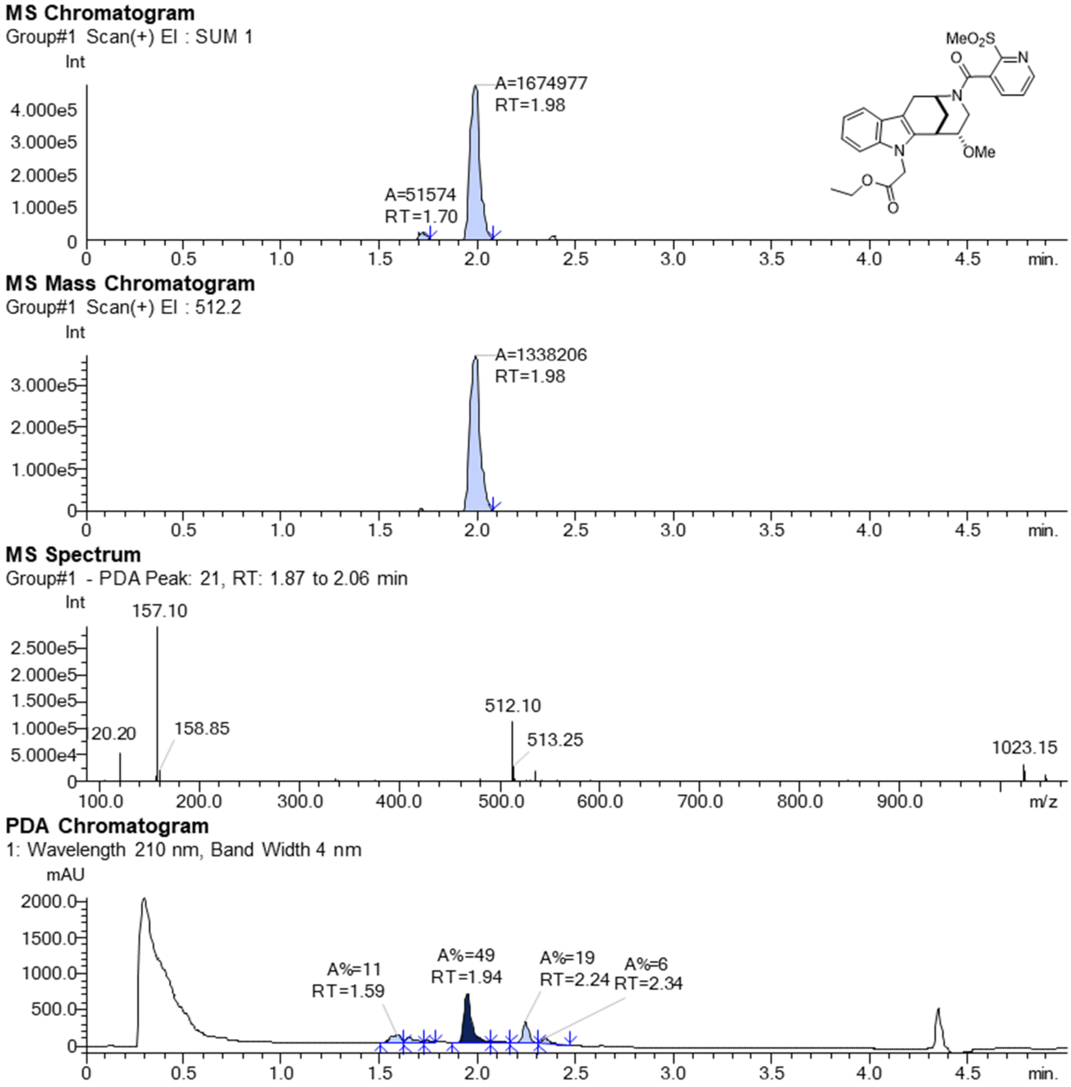
MS Chromatogram
Group#1 Scan(+) EI : SUM 1
Int
A=1674977
RT=1.98
A=51574
RT=1.70
min.
MeO2S
N
O
N
OMe
O
O
MS Mass Chromatogram
Group#1 Scan(+) EI : 512.2
Int
A=1338206
RT=1.98
min.
MS Spectrum
Group#1 - PDA Peak: 21, RT: 1.87 to 2.06 min
Int
157.10
120.20
158.85
512.10
513.25
1023.15
m/z
PDA Chromatogram
1: Wavelength 210 nm, Band Width 4 nm
mAU
A%=11
RT=1.59
A%=49
RT=1.94
A%=19
RT=2.24
A%=6
RT=2.34
min.

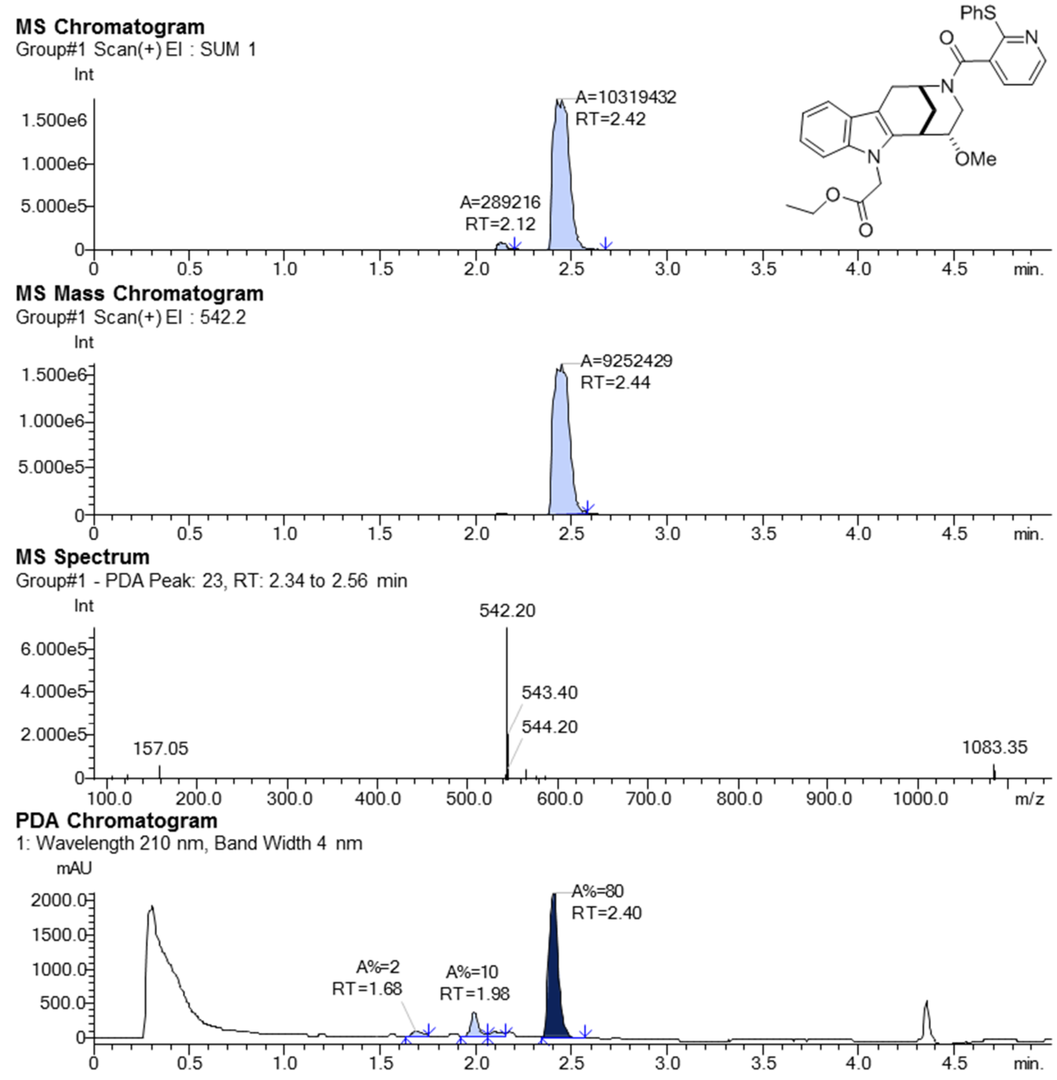
MS Chromatogram
Group#1 Scan(+) EI : SUM 1
A=10319432
RT=2.42
A=289216
RT=2.12
MS Mass Chromatogram
Group#1 Scan(+) EI : 542.2
A=9252429
RT=2.44
MS Spectrum
Group#1 - PDA Peak: 23, RT: 2.34 to 2.56 min
542.20
543.40
544.20
157.05
1083.35
PDA Chromatogram
1: Wavelength 210 nm, Band Width 4 nm
A%=80
RT=2.40
A%=2
RT=1.68
A%=10
RT=1.98
PhS
N
O
OMe

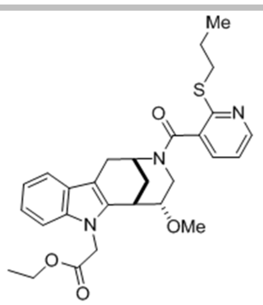

MS Chromatogram
Group#1 Scan(+) EI : SUM 1

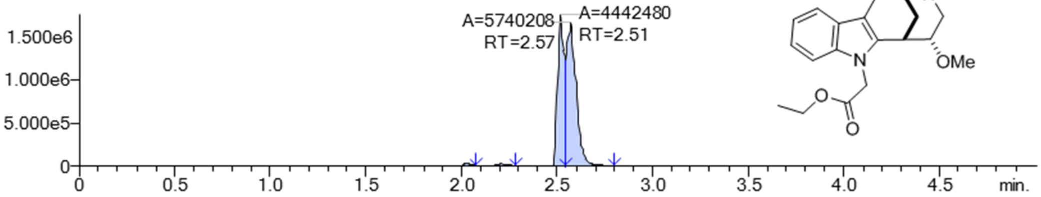

MS Mass Chromatogram
Group#1 Scan(+) EI : 508.2

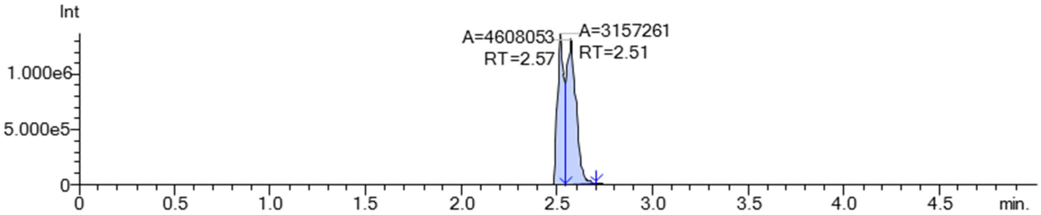

MS Spectrum
Group#1 - PDA Peak: 23, RT: 2.45 to 2.69 min

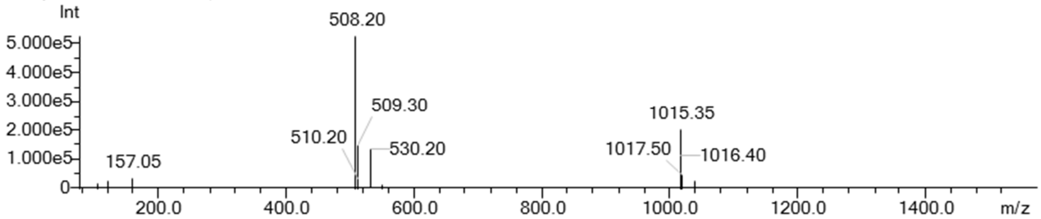

PDA Chromatogram
1: Wavelength 210 nm, Band Width 4 nm

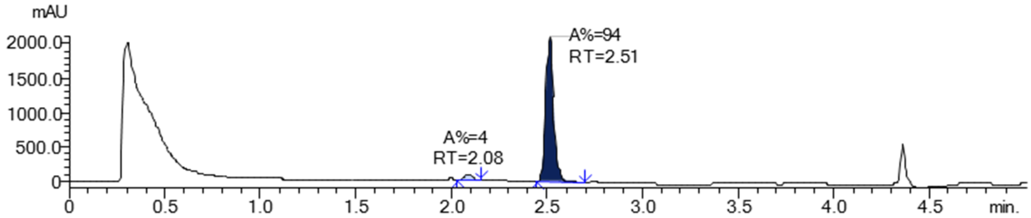

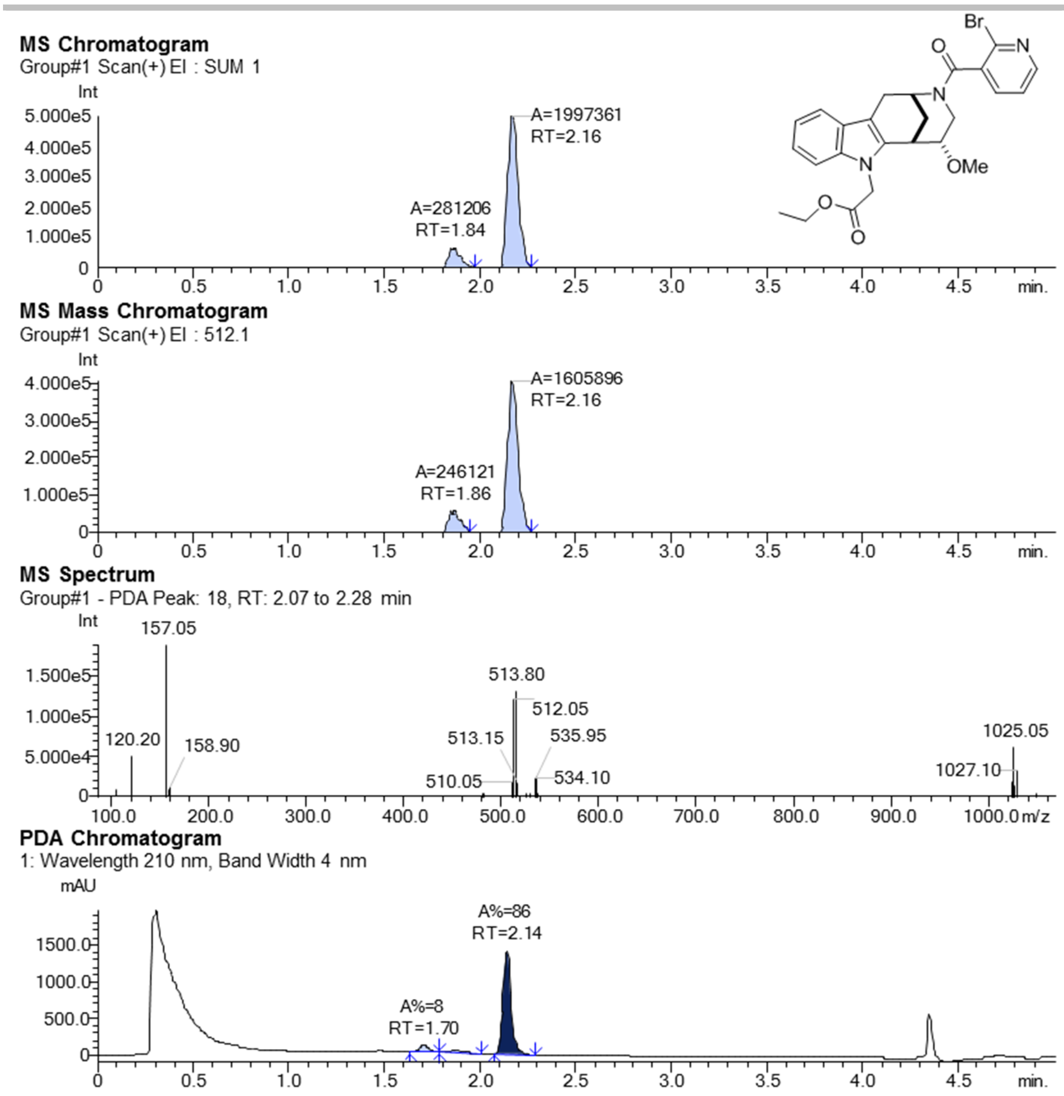
MS Chromatogram
Group#1 Scan(+) EI : SUM 1
Int
A=1997361
RT=2.16
A=281206
RT=1.84
min.
MS Mass Chromatogram
Group#1 Scan(+) EI : 512.1
Int
A=1605896
RT=2.16
A=246121
RT=1.86
min.
MS Spectrum
Group#1 - PDA Peak: 18, RT: 2.07 to 2.28 min
Int
157.05
513.80
512.05
120.20
158.90
513.15
535.95
1025.05
510.05
534.10
1027.10
m/z
PDA Chromatogram
1: Wavelength 210 nm, Band Width 4 nm
mAU
A%=86
RT=2.14
A%=8
RT=1.70
min.
Br
N
O
OMe

MS Chromatogram

Group#1 Scan(+) EI : SUM 1

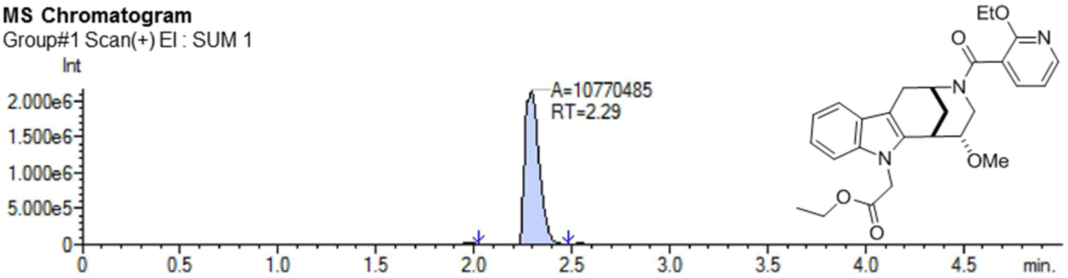

MS Mass Chromatogram

Group#1 Scan(+) EI : 478.2

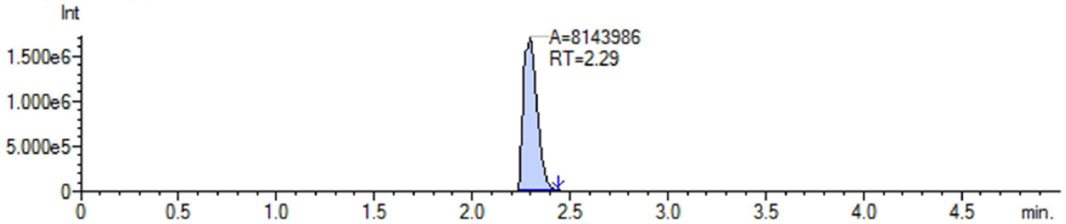

MS Spectrum

Group#1 - PDA Peak 18, RT: 2.20 to 2.43 min

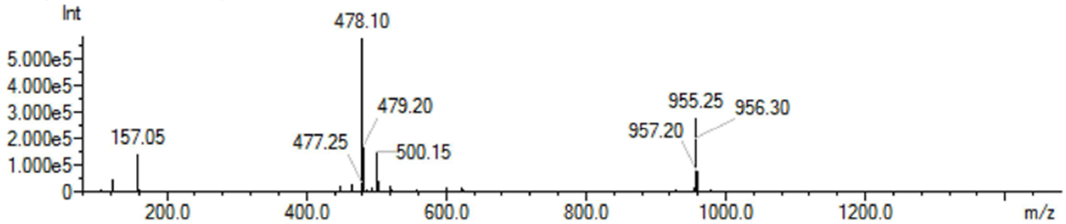

PDA Chromatogram

1: Wavelength 210 nm, Band Width 4 nm

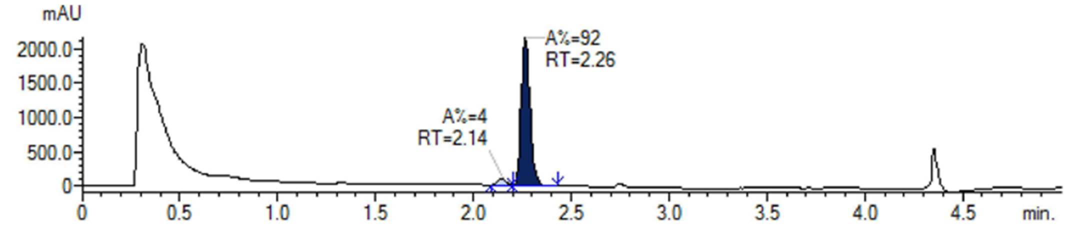

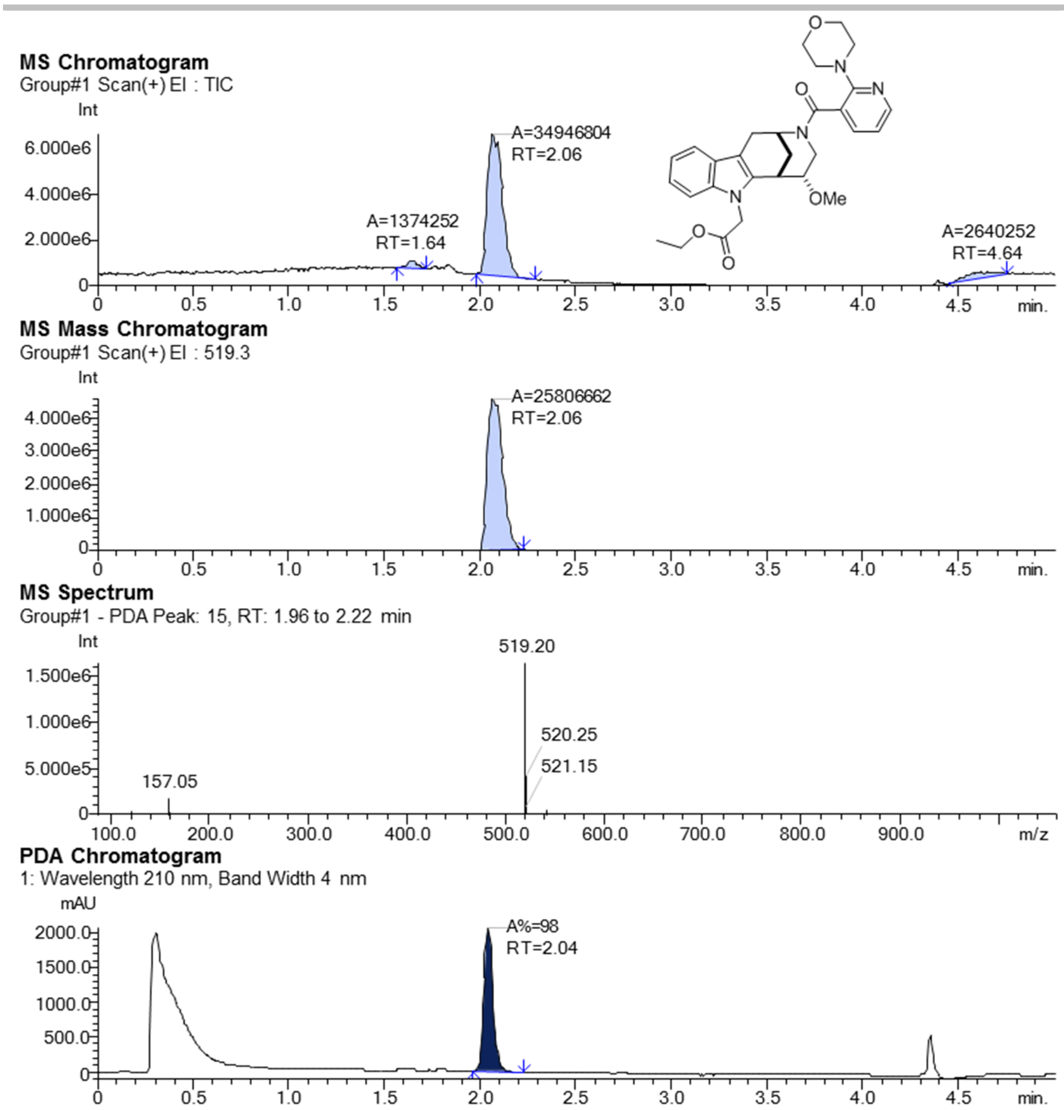
MS Chromatogram
Group#1 Scan(+) EI : TIC
Int
6.000e6
4.000e6
2.000e6
0
A=34946804
RT=2.06
A=1374252
RT=1.64
A=2640252
RT=4.64
OMe
0
0.5
1.0
1.5
2.0
2.5
3.0
3.5
4.0
4.5
min.
MS Mass Chromatogram
Group#1 Scan(+) EI : 519.3
Int
4.000e6
3.000e6
2.000e6
1.000e6
0
A=25806662
RT=2.06
min.
MS Spectrum
Group#1 - PDA Peak: 15, RT: 1.96 to 2.22 min
Int
1.500e6
1.000e6
5.000e5
0
519.20
520.25
521.15
157.05
100.0
200.0
300.0
400.0
500.0
600.0
700.0
800.0
900.0
m/z
PDA Chromatogram
1: Wavelength 210 nm, Band Width 4 nm
mAU
2000.0
1500.0
1000.0
500.0
0
A%=98
RT=2.04
min.

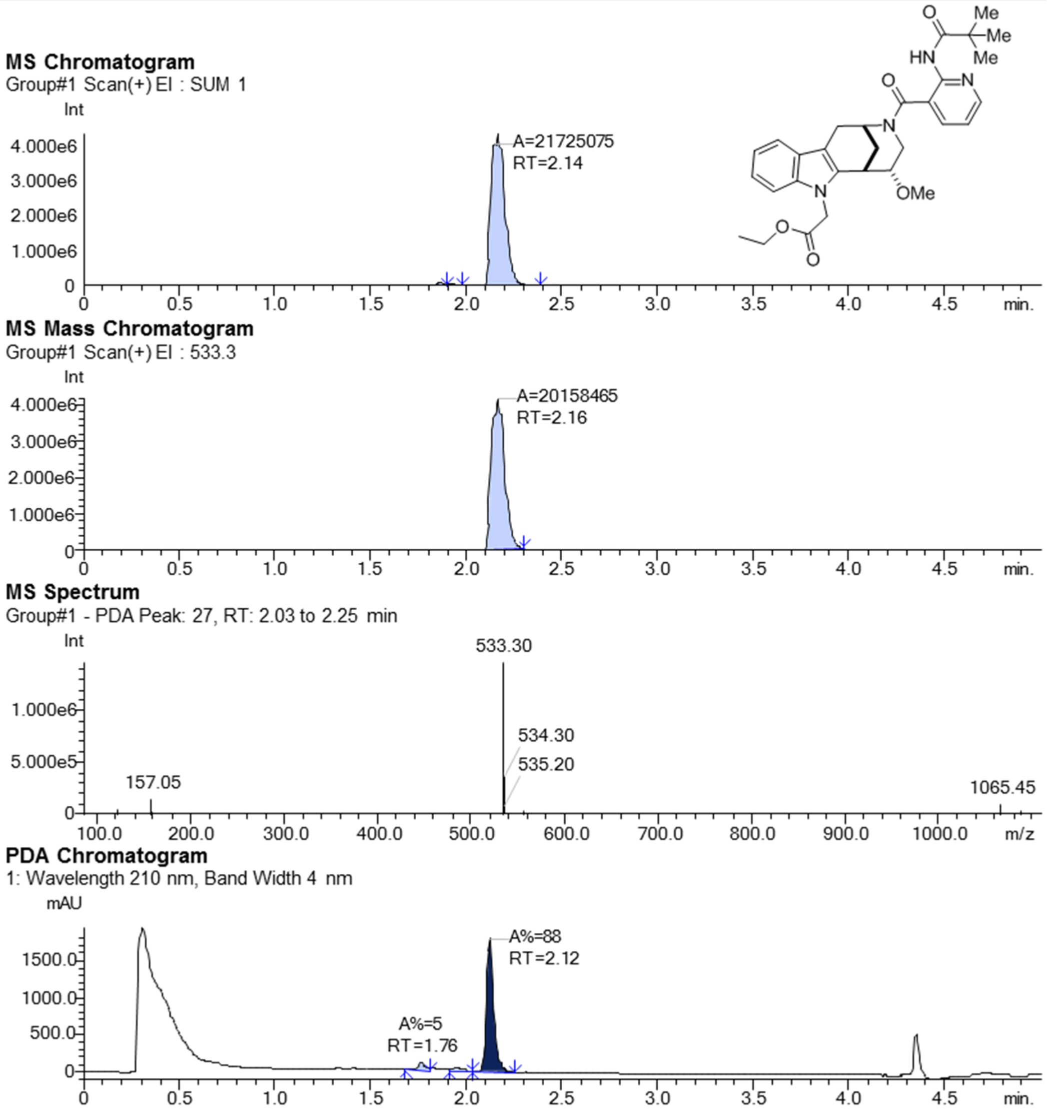
MS Chromatogram
Group#1 Scan(+) EI : SUM 1
A=21725075
RT=2.14
MS Mass Chromatogram
Group#1 Scan(+) EI : 533.3
A=20158465
RT=2.16
MS Spectrum
Group#1 - PDA Peak: 27, RT: 2.03 to 2.25 min
533.30
534.30
535.20
157.05
1065.45
PDA Chromatogram
1: Wavelength 210 nm, Band Width 4 nm
A%=88
RT=2.12
A%=5
RT=1.76

MS Chromatogram

Group#1 Scan(+) EI : SUM 1

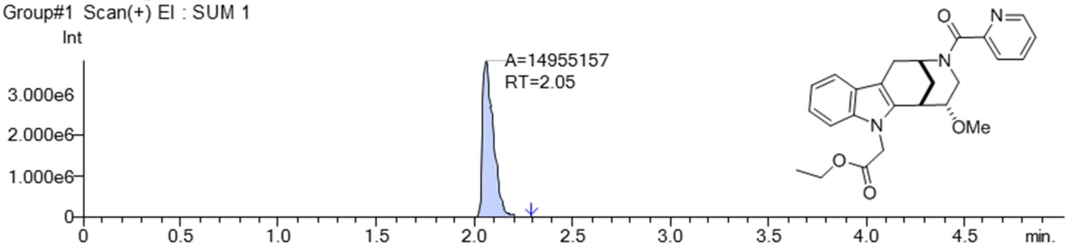

MS Mass Chromatogram

Group#1 Scan(+) EI : 434.2

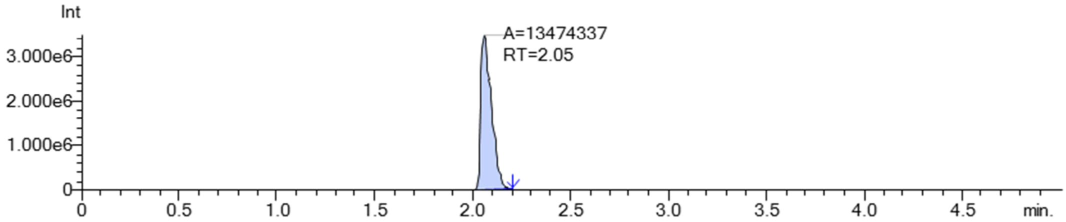

MS Spectrum

Group#1 - PDA Peak: 16, RT: 1.99 to 2.23 min

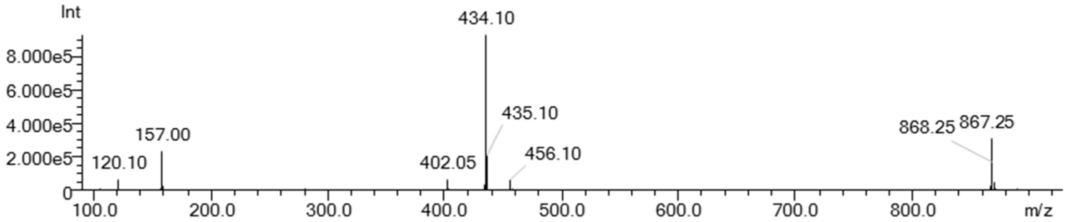

PDA Chromatogram

1: Wavelength 210 nm, Band Width 4 nm

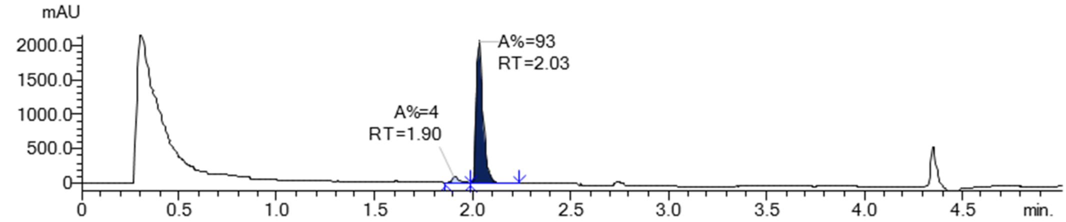

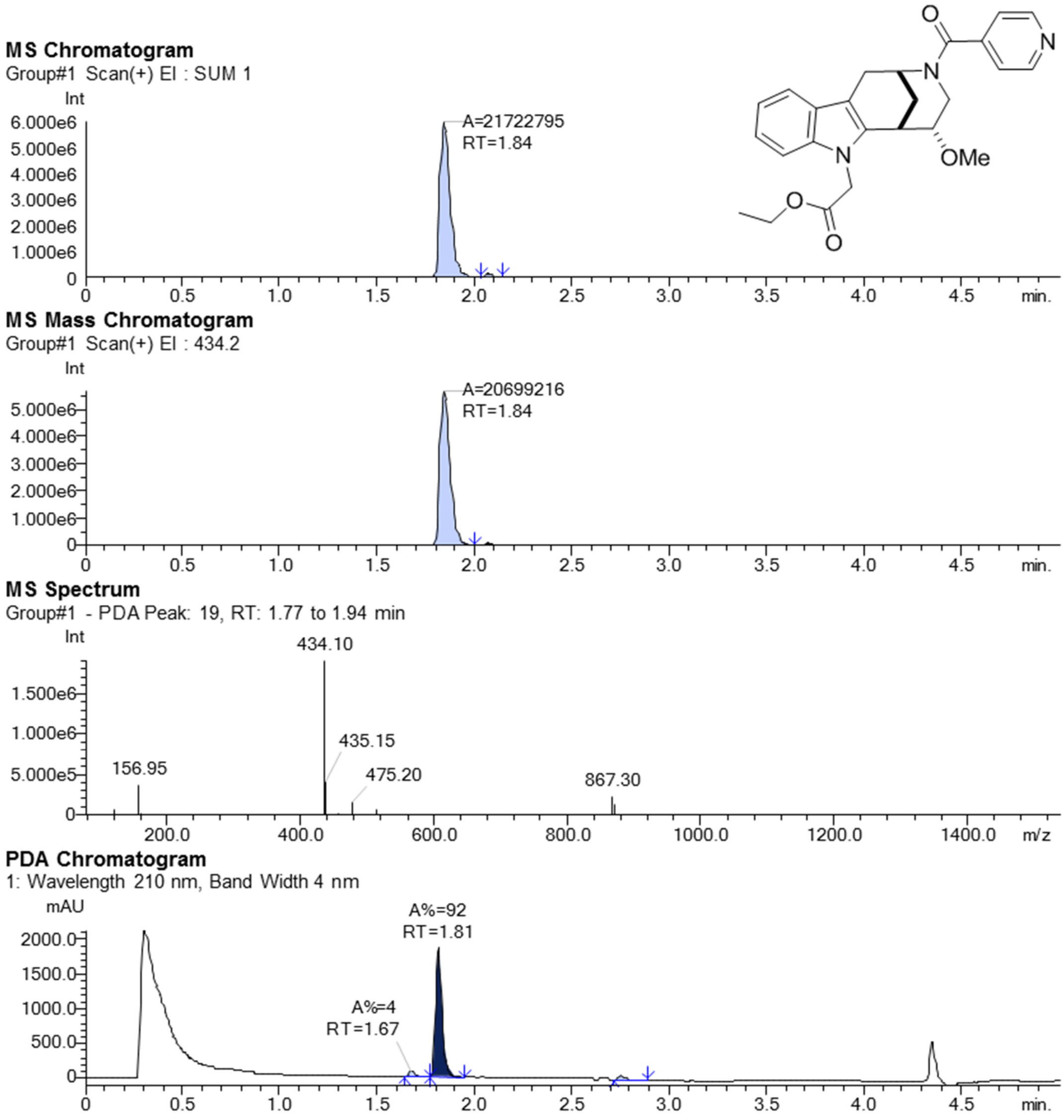
MS Chromatogram
Group#1 Scan(+) EI : SUM 1
Int
A=21722795
RT=1.84
min.
MS Mass Chromatogram
Group#1 Scan(+) EI : 434.2
Int
A=20699216
RT=1.84
min.
MS Spectrum
Group#1 - PDA Peak: 19, RT: 1.77 to 1.94 min
Int
434.10
435.15
156.95
475.20
867.30
m/z
PDA Chromatogram
1: Wavelength 210 nm, Band Width 4 nm
mAU
A%=92
RT=1.81
A%=4
RT=1.67
min.
O
N
N
N
OMe
O
O

MS Chromatogram
Group#1 Scan(+) EI : SUM 1

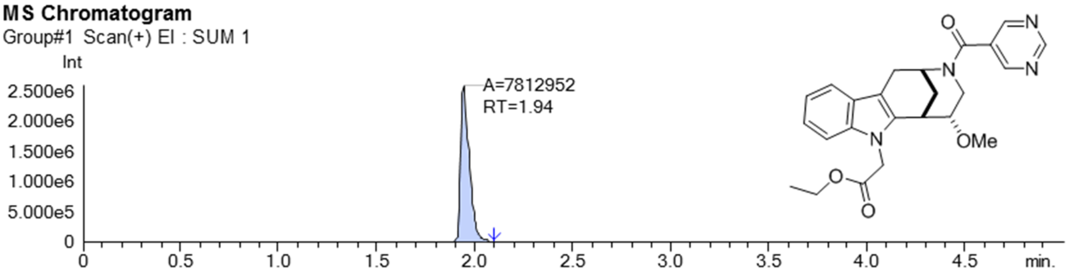

MS Mass Chromatogram
Group#1 Scan(+) EI : 435.2

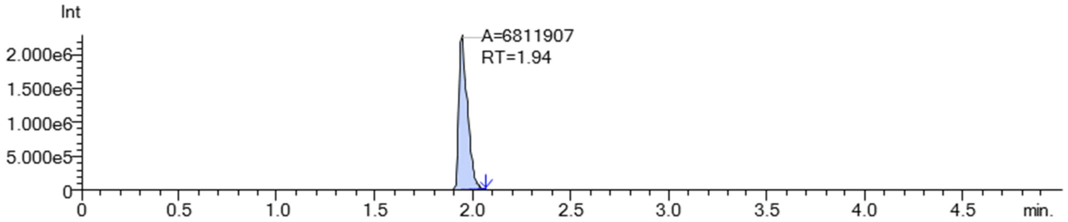

MS Spectrum
Group#1 - PDA Peak: 18, RT: 1.87 to 2.05 min

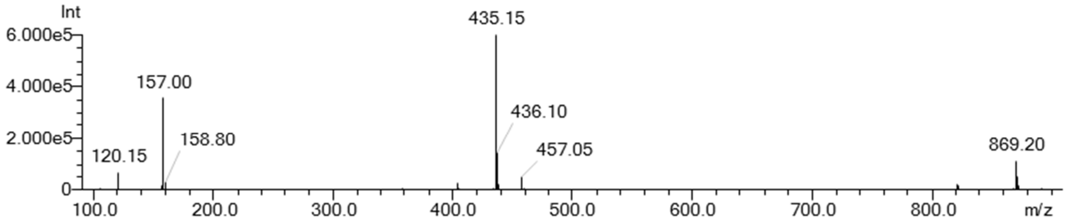

PDA Chromatogram
1: Wavelength 210 nm, Band Width 4 nm

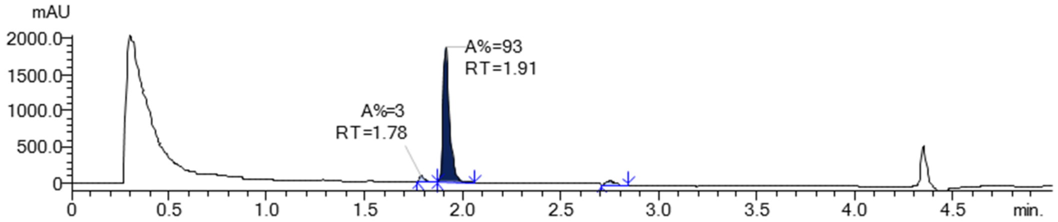

MS Chromatogram
Group#1 Scan(+) EI : SUM 1

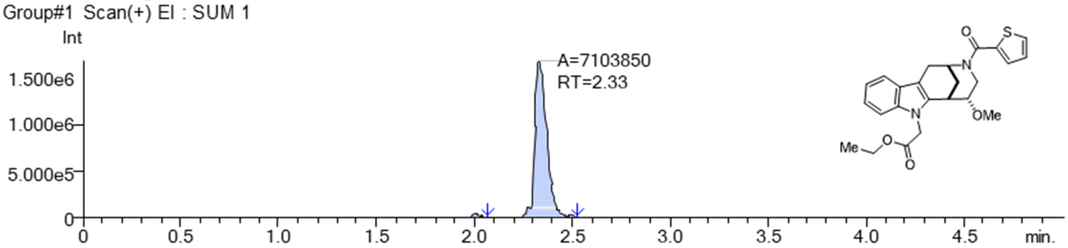

MS Mass Chromatogram
Group#1 Scan(+) EI : 439.2

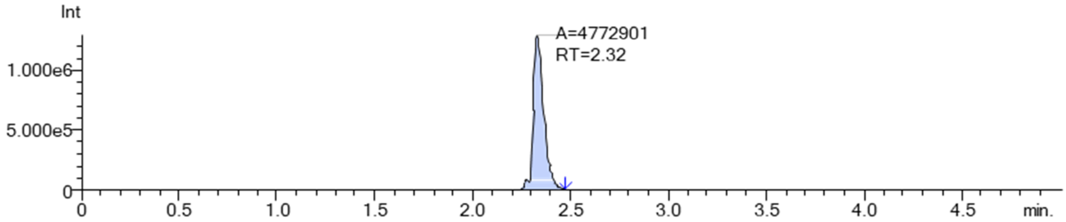

MS Spectrum
Group#1 - PDA Peak: 18, RT: 2.22 to 2.45 min

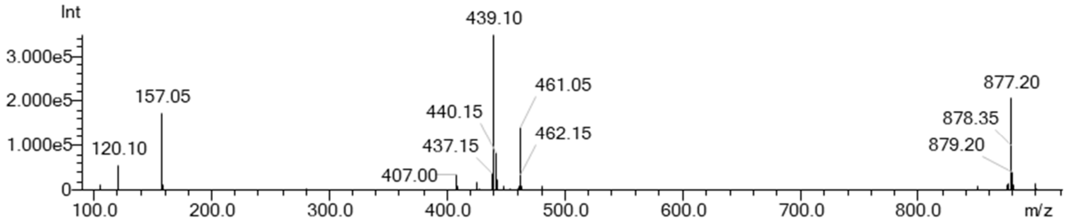

PDA Chromatogram
1: Wavelength 210 nm, Band Width 4 nm

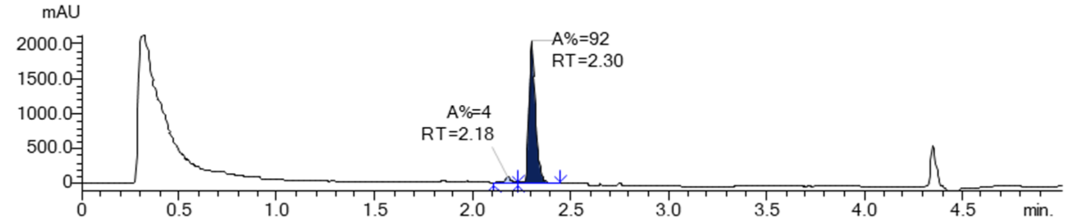

MS Chromatogram
Group#1 Scan(+) EI : SUM 1

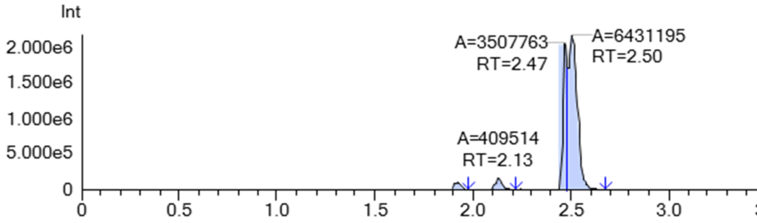

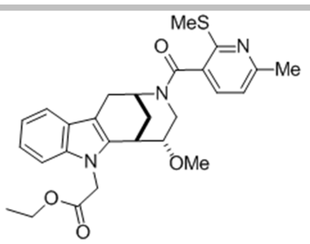

MS Mass Chromatogram
Group#1 Scan(+) EI : 494.2

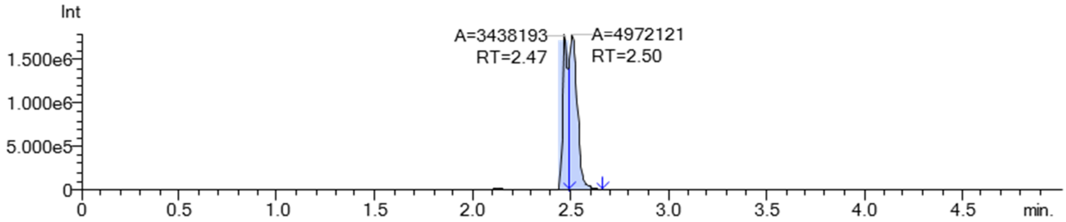

MS Spectrum
Group#1 - PDA Peak: 25, RT: 2.41 to 2.61 min

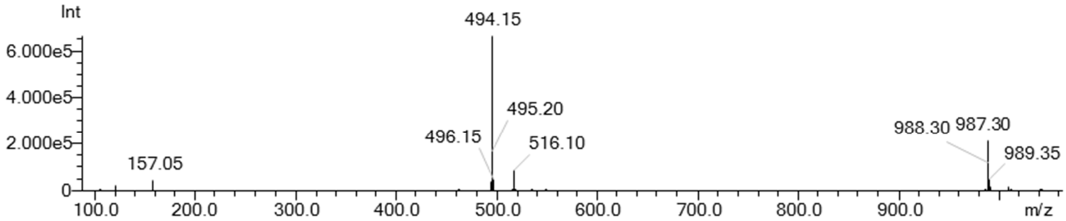

PDA Chromatogram
1: Wavelength 210 nm, Band Width 4 nm

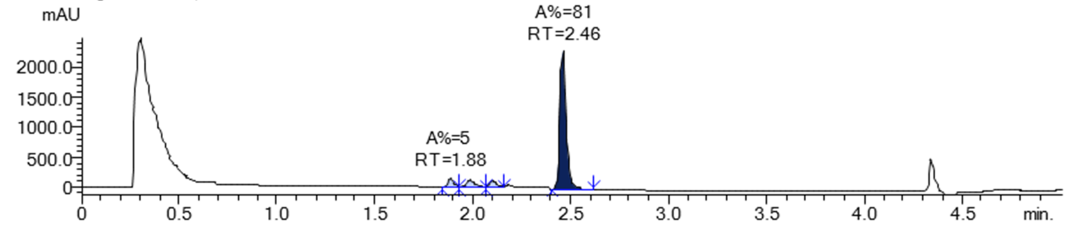

MS Chromatogram
Group#1 Scan(+) EI : SUM 1

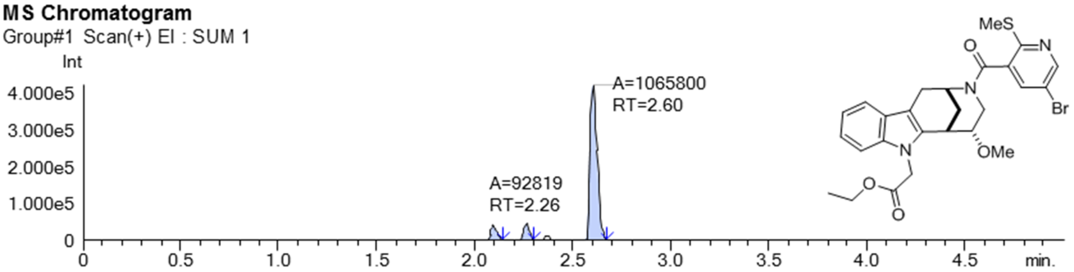

MS Mass Chromatogram
Group#1 Scan(+) EI : 558.1

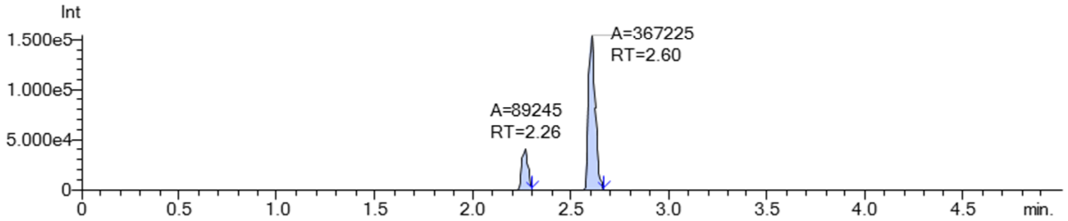

MS Spectrum
Group#1 - PDA Peak: 16, RT: 2.52 to 2.66 min

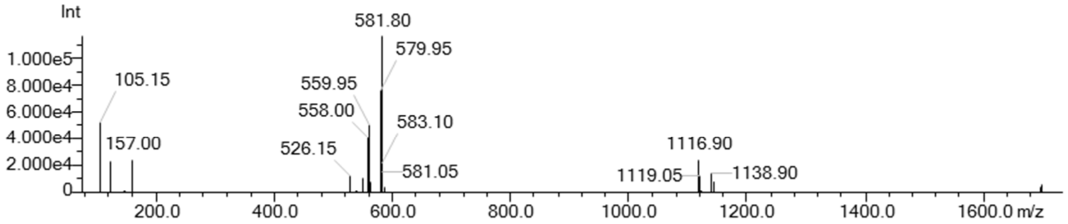

PDA Chromatogram
1: Wavelength 210 nm, Band Width 4 nm

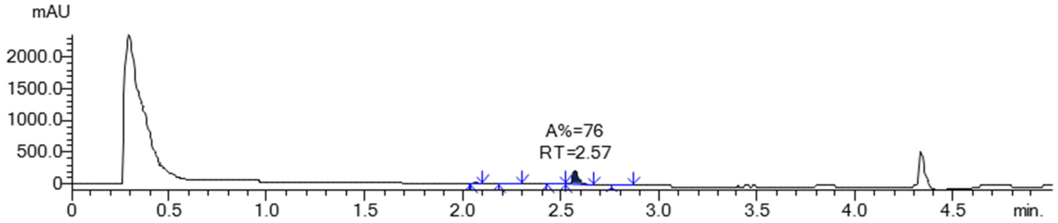

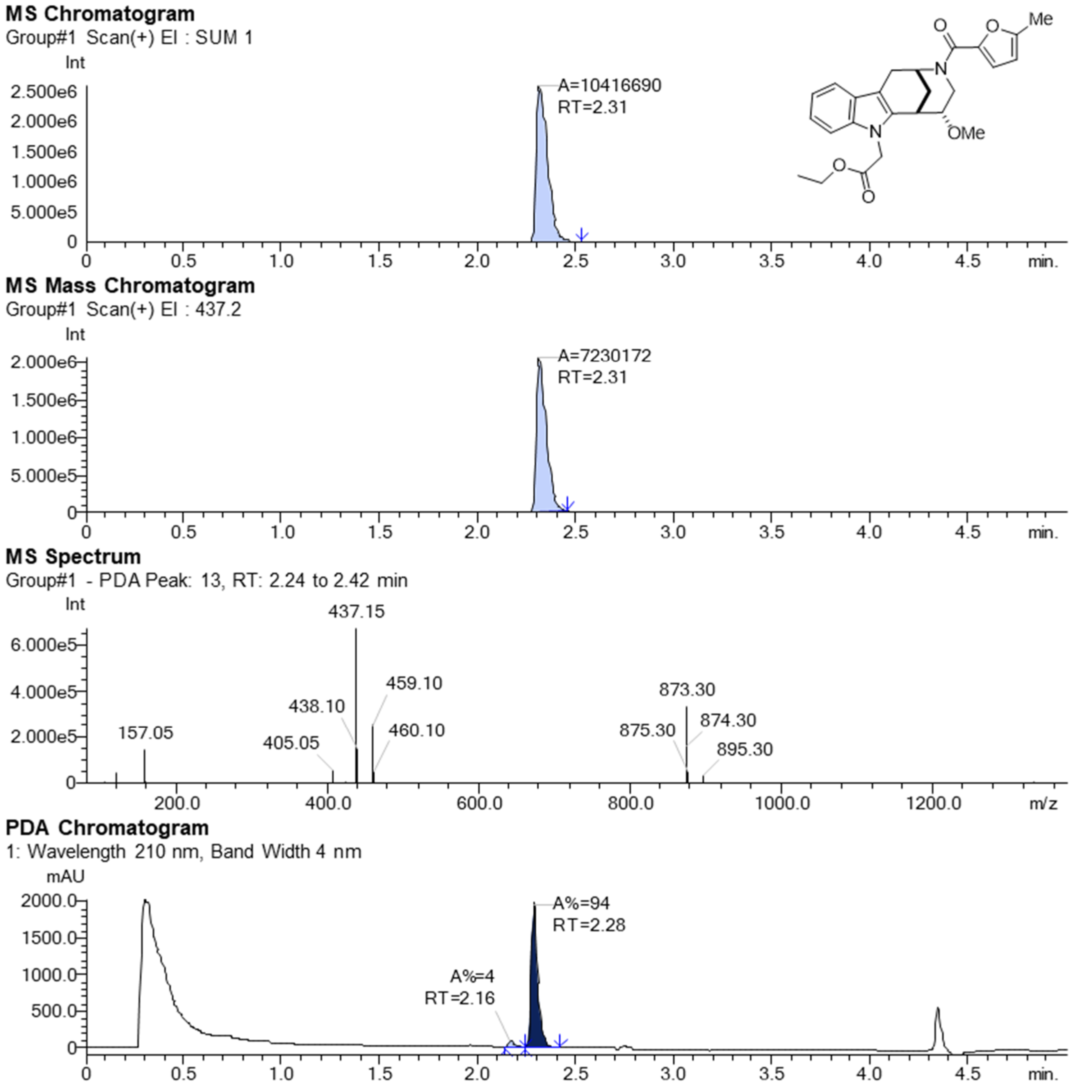
MS Chromatogram
Group#1 Scan(+) EI : SUM 1
Int
A=10416690
RT=2.31
min.
MS Mass Chromatogram
Group#1 Scan(+) EI : 437.2
Int
A=7230172
RT=2.31
min.
MS Spectrum
Group#1 - PDA Peak: 13, RT: 2.24 to 2.42 min
Int
437.15
438.10
459.10
460.10
405.05
157.05
873.30
874.30
875.30
895.30
m/z
PDA Chromatogram
1: Wavelength 210 nm, Band Width 4 nm
mAU
A%=94
RT=2.28
A%=4
RT=2.16
min.
Me
OMe

MS Chromatogram

Group#1 Scan(+) EI : SUM 1

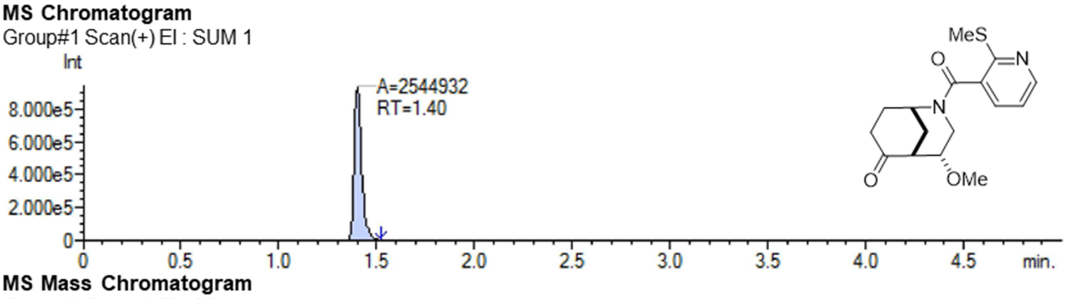

MS Mass Chromatogram

Group#1 Scan(+) EI : 321.1

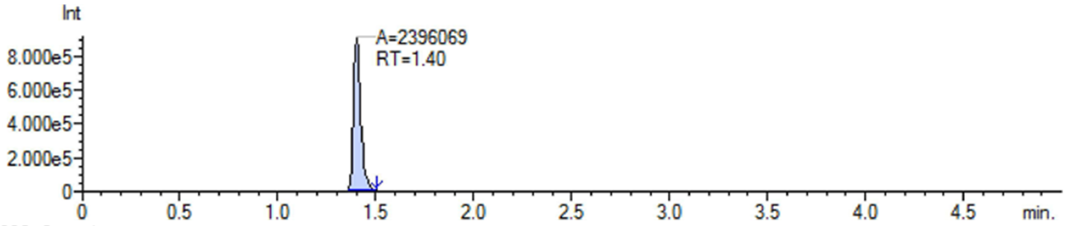

MS Spectrum

Group#1 - PDA Peak 9, RT: 1.32 to 1.47 min

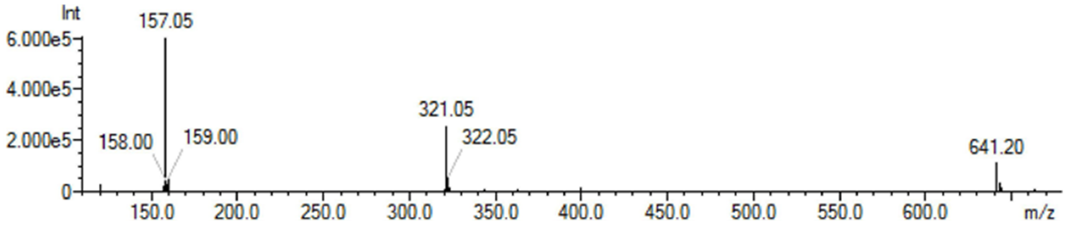

PDA Chromatogram

1: Wavelength 210 nm, Band Width 4 nm

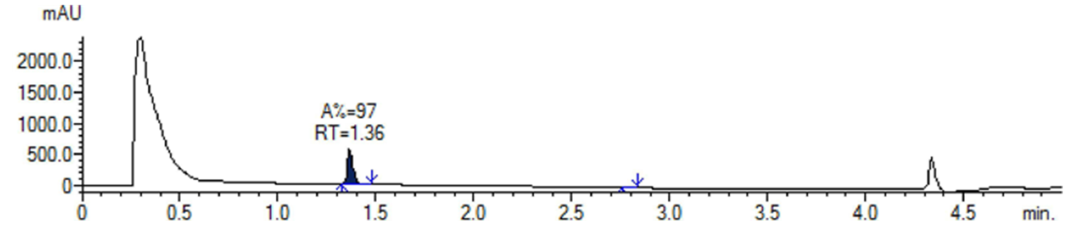

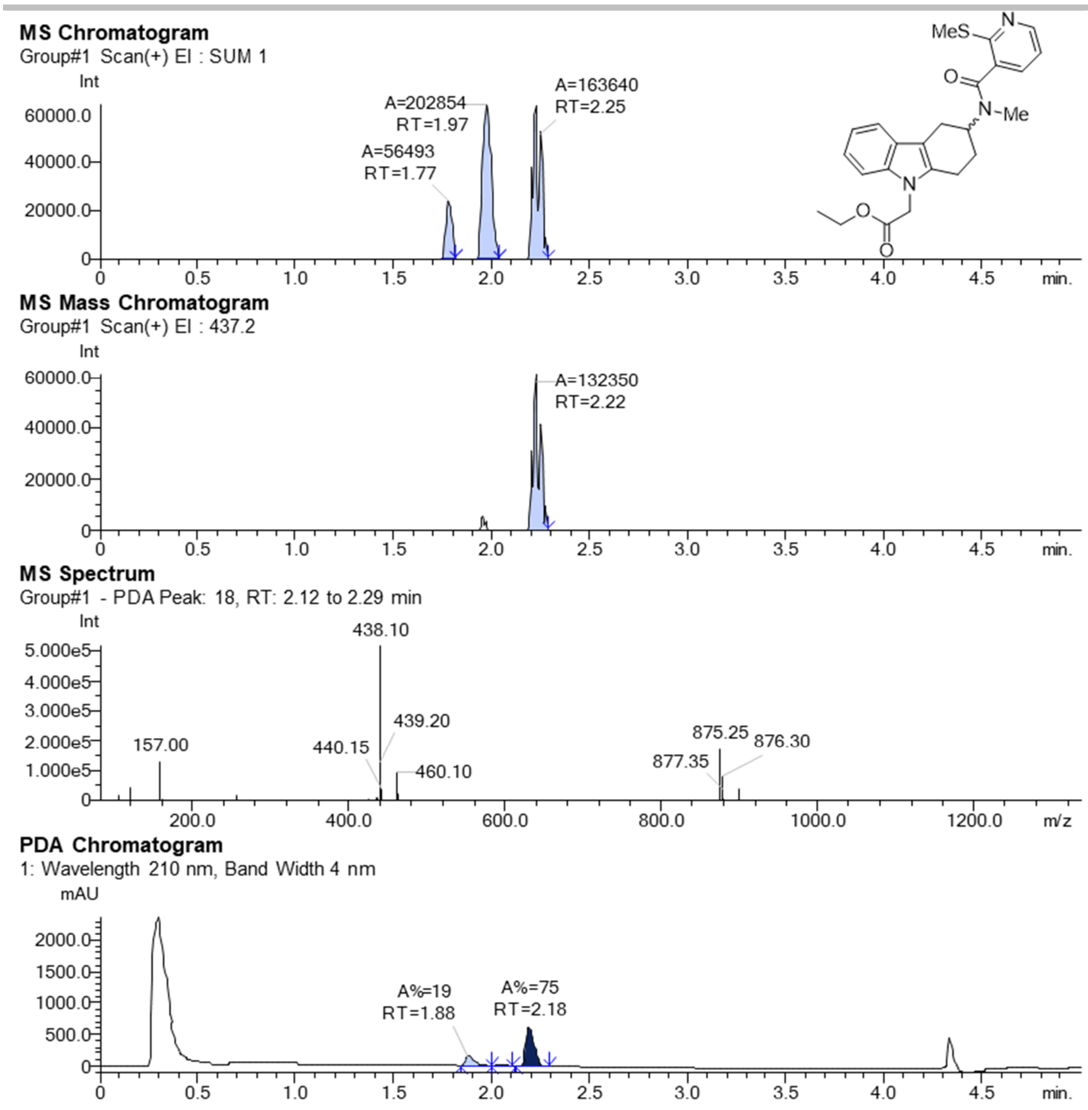
MS Chromatogram
Group#1 Scan(+) EI : SUM 1
Int
A=202854
RT=1.97
A=163640
RT=2.25
A=56493
RT=1.77
min.
MeS
N
O
N-Me
N
O
O
MS Mass Chromatogram
Group#1 Scan(+) EI : 437.2
A=132350
RT=2.22
MS Spectrum
Group#1 - PDA Peak: 18, RT: 2.12 to 2.29 min
438.10
439.20
157.00
440.15
460.10
875.25
876.30
877.35
m/z
PDA Chromatogram
1: Wavelength 210 nm, Band Width 4 nm
mAU
A%=19
RT=1.88
A%=75
RT=2.18

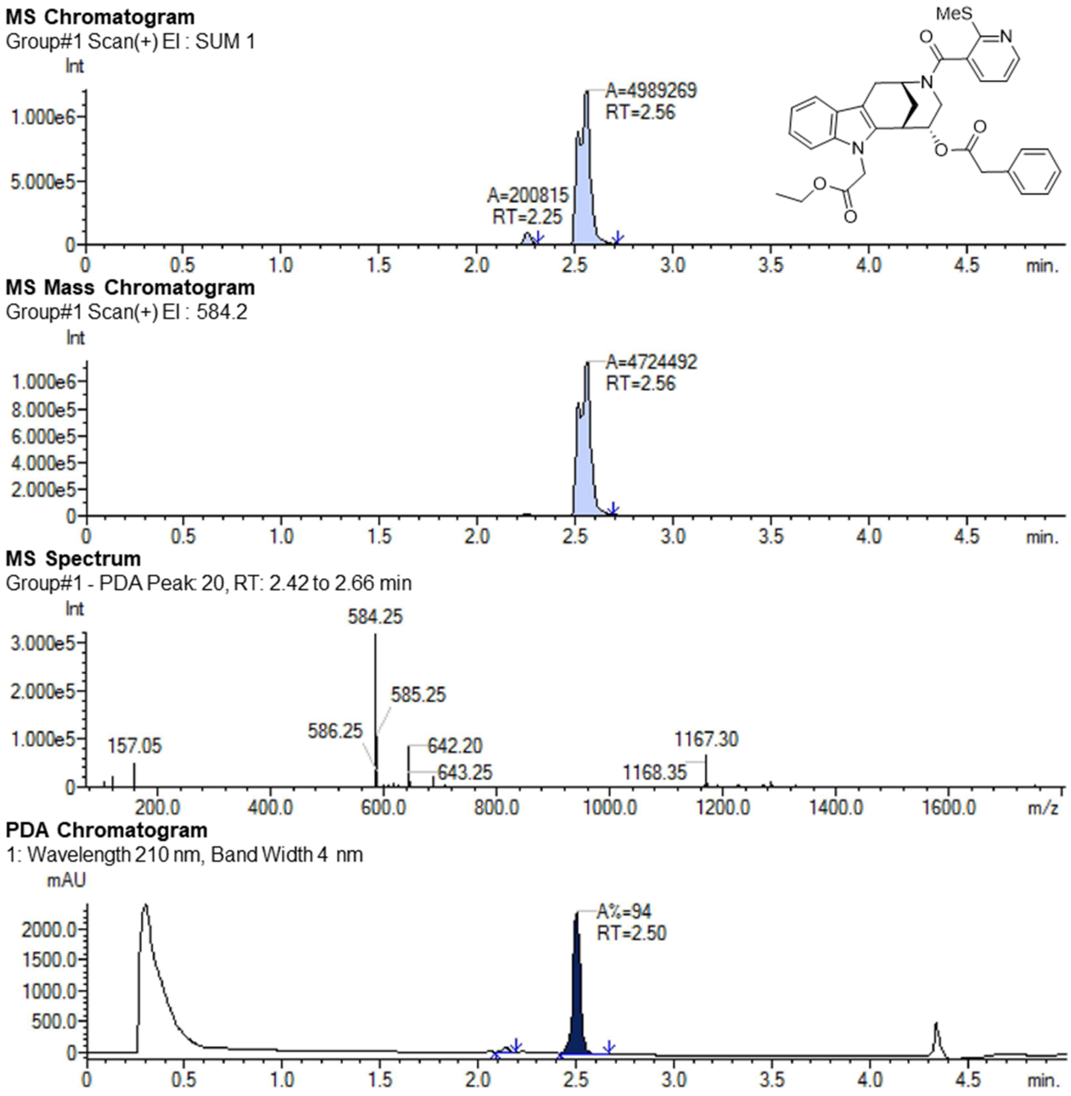
MS Chromatogram
Group#1 Scan(+) EI : SUM 1
A=4989269
RT=2.56
A=200815
RT=2.25
MeS
N
O
MS Mass Chromatogram
Group#1 Scan(+) EI : 584.2
A=4724492
RT=2.56
MS Spectrum
Group#1 - PDA Peak 20, RT: 2.42 to 2.66 min
584.25
585.25
586.25
157.05
642.20
643.25
1167.30
1168.35
PDA Chromatogram
1: Wavelength 210 nm, Band Width 4 nm
A%=94
RT=2.50

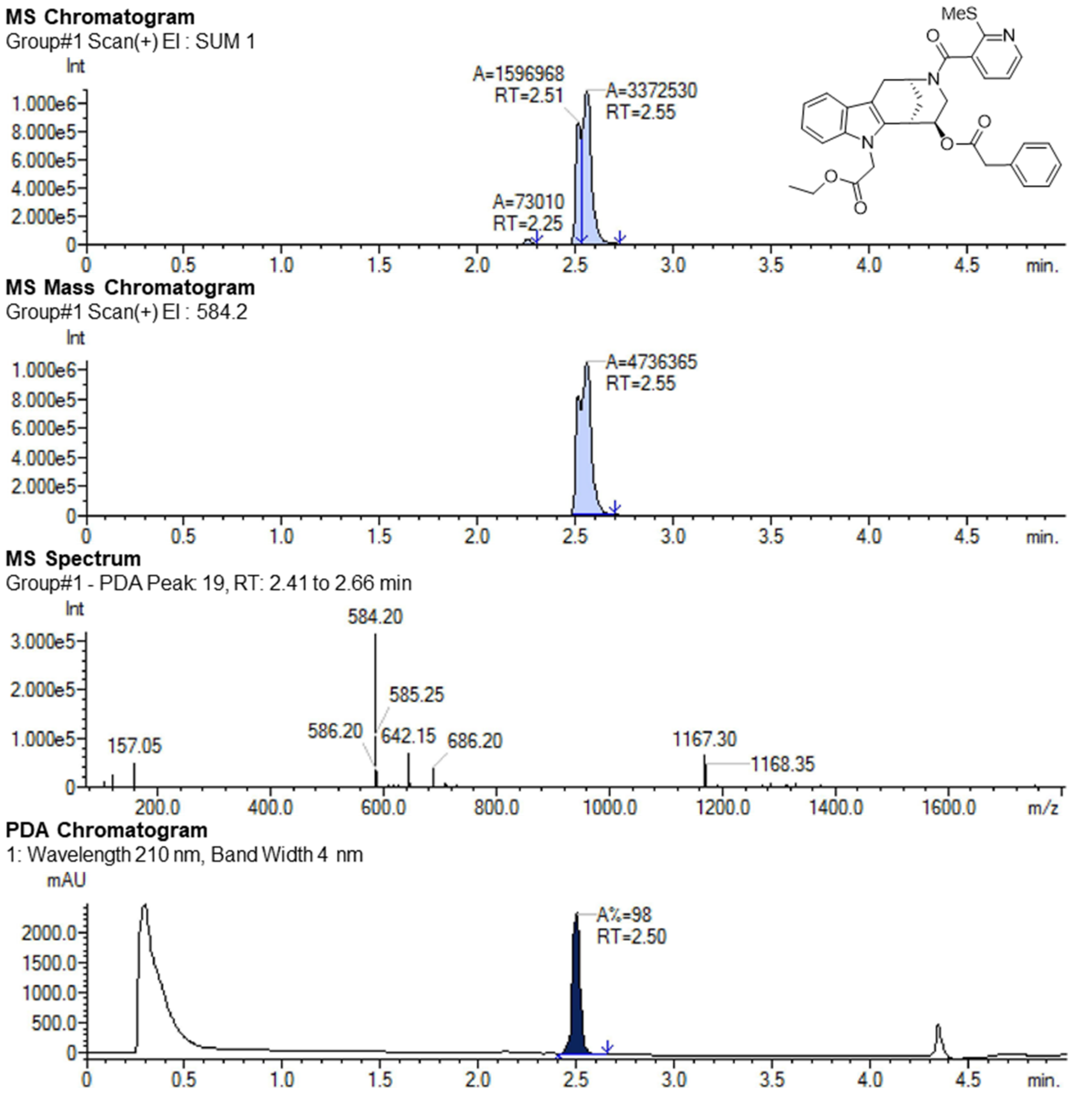
MS Chromatogram
Group#1 Scan(+) EI : SUM 1
Int
A=1596968
RT=2.51
A=3372530
RT=2.55
A=73010
RT=2.25
min.
MeS
N
O
MS Mass Chromatogram
Group#1 Scan(+) EI : 584.2
A=4736365
RT=2.55
MS Spectrum
Group#1 - PDA Peak 19, RT: 2.41 to 2.66 min
584.20
585.25
586.20
642.15
686.20
157.05
1167.30
1168.35
m/z
PDA Chromatogram
1: Wavelength 210 nm, Band Width 4 nm
mAU
A%=98
RT=2.50

Biology Part

Supporting Figures S7-S13

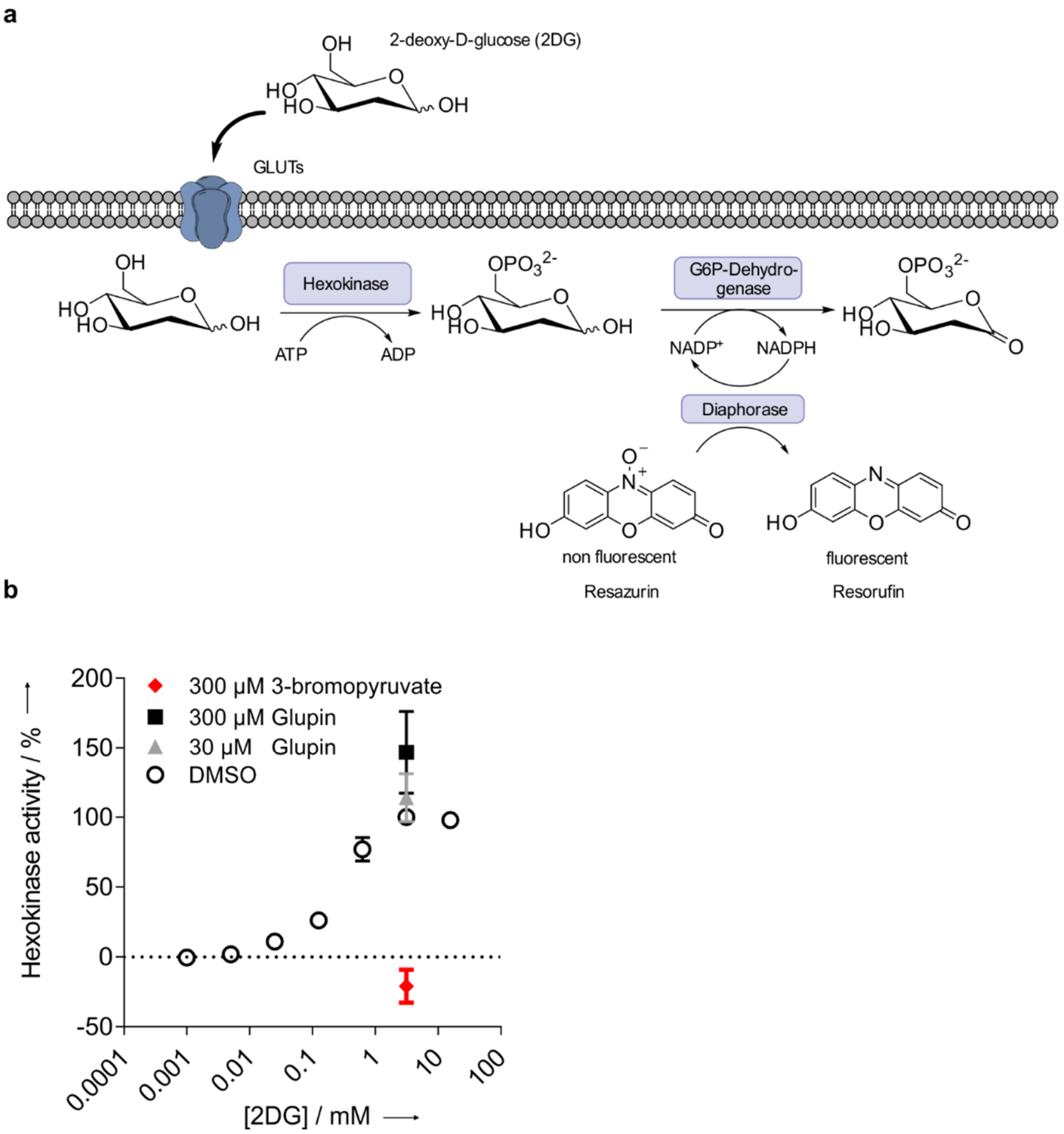

Supporting Figure S7: Screening for modulators of glucose uptake. (a) Schematic representation of the cellular glucose uptake assay. 2-deoxy-*D*-glucose (2DG) is taken up by glucose transporters. 2DG is subsequently phosphorylated by intracellular hexokinase to 2-deoxyglucose-6-phosphate (2DG6P), which is not further metabolized and is trapped inside the cells. 2DG6P is converted by glucose-6-phosphate dehydrogenase (G6PDH) to NADPH and 6-phospho-2-deoxyglucoronic acid. NADPH is subsequently used as reducing agent to generate fluorescent resorufin from non-fluorescent resazurin by diaphorase. The fluorescence intensity (ex/em 530/590) is directly proportional to the amount of 2DG6P

in the cell lysate. (c) Influence of Glupin on the activity of hexokinase. Lysates of HCT116 cells were supplemented with ATP, $NADP^+$, 2DG and G6PDH and were incubated with the compounds for 40 min. The generation of $NADPH+H^+$ was determined at ex/em 340 nm/445 as a measure of hexokinase kinase activity. Data are mean values ± s.d. of three biological replicates.

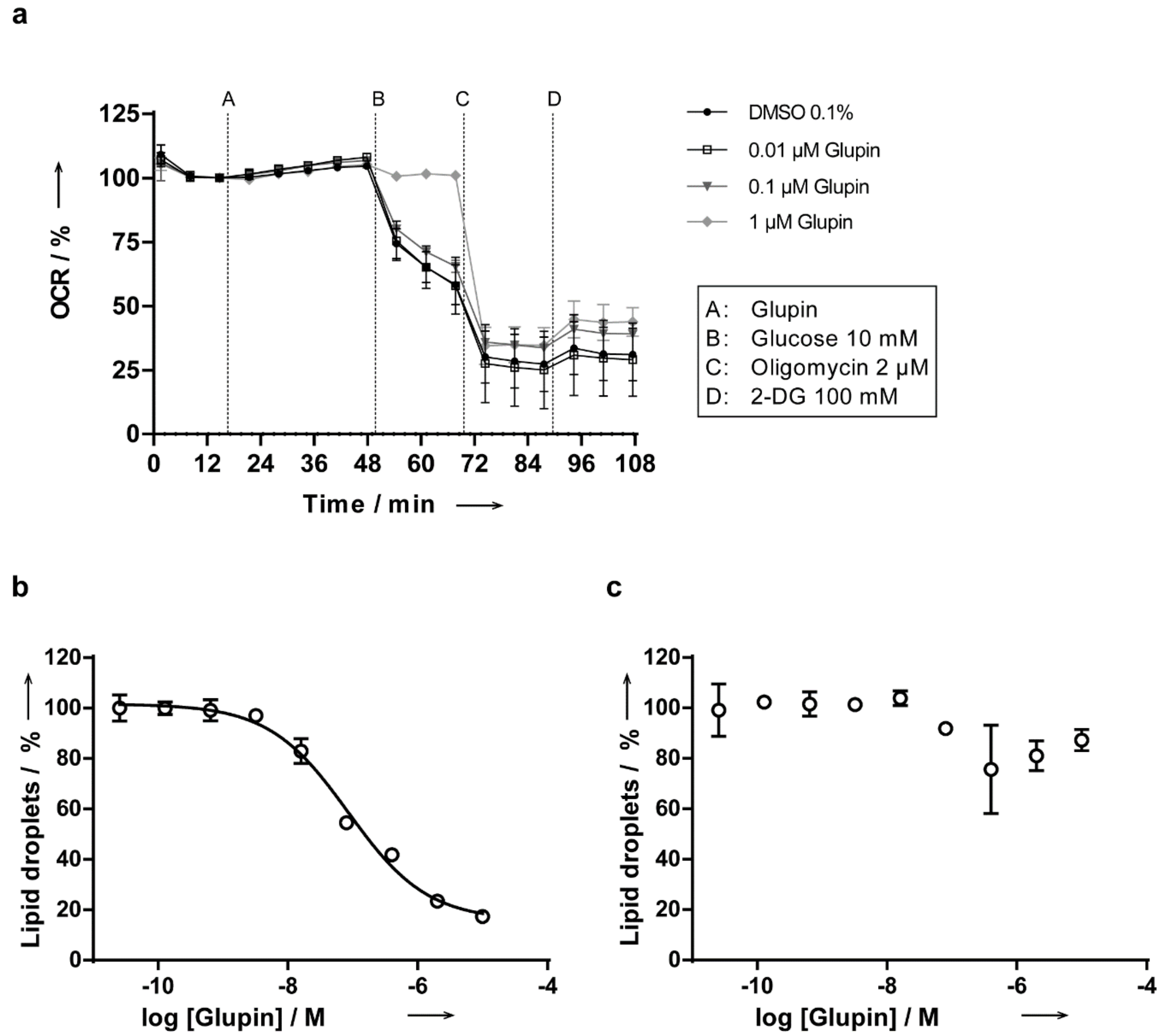

Supporting Figure S8: Influence of Glupin on mitochondrial respiration and lipid droplet formation. (a) Glucose-starved MDA-MB-231 cells were treated with Glupin and oxygen consumption rate (OCR) was measured over time in a Seahorse XFp Extracellular flux analyzer. Data are mean values ± SD (N=3, n=3). (b and c) MDA-MB-231 cells were treated with 400 µM oleic acids together with Glupin for 18 h (b) or were pretreated with 400 µM oleic acids overnight followed by addition of the compound and incubation for 18 h (c). Data are mean values SD (n=2).

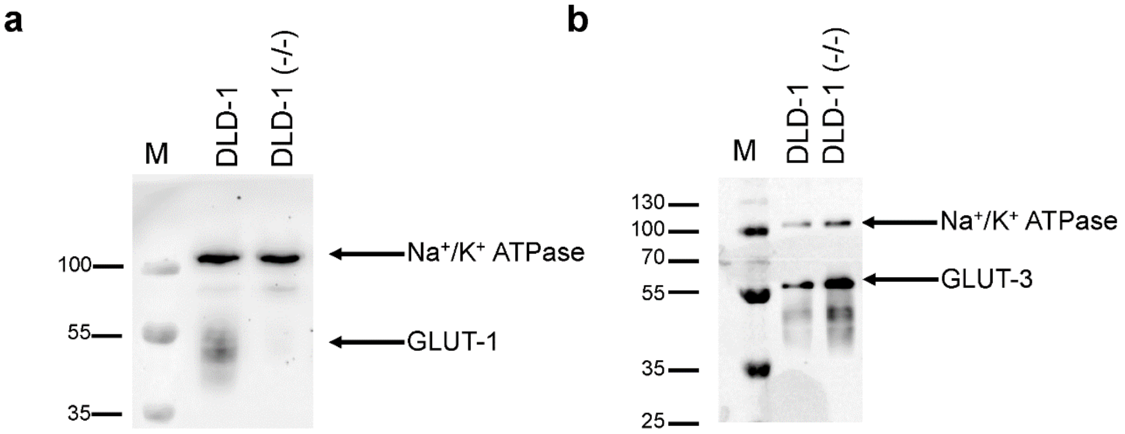

Supporting Figure S9: Detection of GLUT-1 and GLUT-3 in DLD-1 and DLD-1 GLUT1(-/-) cells. Lysates of DLD-1 and DLD-1 *GLUT1*(-/-) (DLD-1 (-/-)) cells were subjected to SDS-PAGE and immunoblotting using antibodies against GLUT-1 (a) or GLUT-3 (b) and anti-Na^{+}/K^{+} ATPase as a reference.[7]

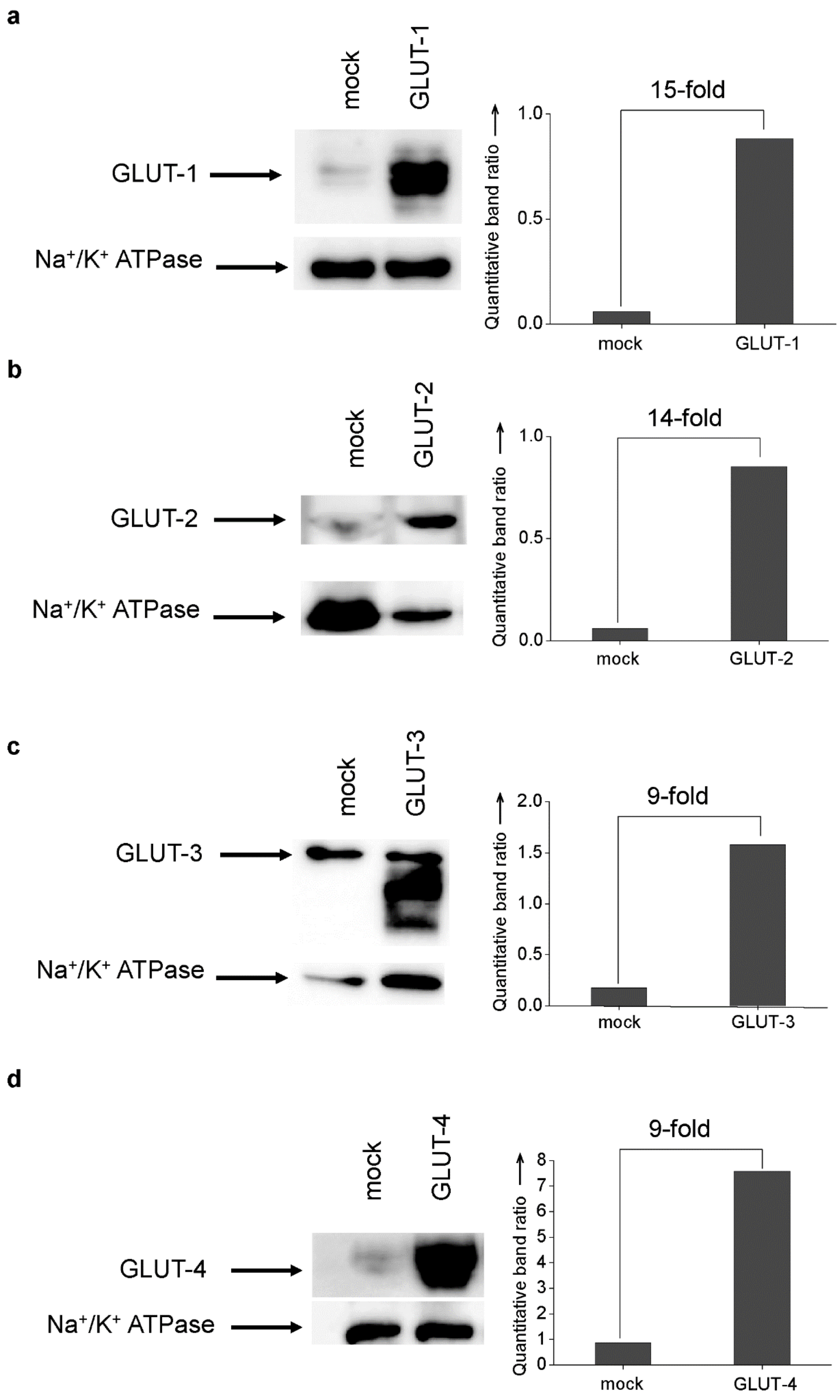
a
mock
GLUT-1
GLUT-1
Na+/K+ ATPase
15-fold
Quantitative band ratio
1.0
0.5
0.0
mock
GLUT-1
b
mock
GLUT-2
GLUT-2
Na+/K+ ATPase
14-fold
Quantitative band ratio
1.0
0.5
0.0
mock
GLUT-2
c
mock
GLUT-3
GLUT-3
Na+/K+ ATPase
9-fold
Quantitative band ratio
2.0
1.5
1.0
0.5
0.0
mock
GLUT-3
d
mock
GLUT-4
GLUT-4
Na+/K+ ATPase
9-fold
Quantitative band ratio
8
7
6
5
4
3
2
1
0
mock
GLUT-4

Supporting Figure S10: Detection of GLUT-1-4 upon overexpression in CHO cells. Cells were transfected with expression plasmids for GLUT-1 (a), GLUT-2 (b), GLUT-3 (c) or GLUT-4 (d). 48 h post transfection cells were lysed and proteins were subjected to SDS-PAGE and immunoblotting using antibodies against the respective GLUT and anti-Na^+/K^+ ATPase as a reference. The graphs show the densitometric quantification of the band intensities in relation to the intensity of the respective band for Na^+/K^+ ATPase.[7]

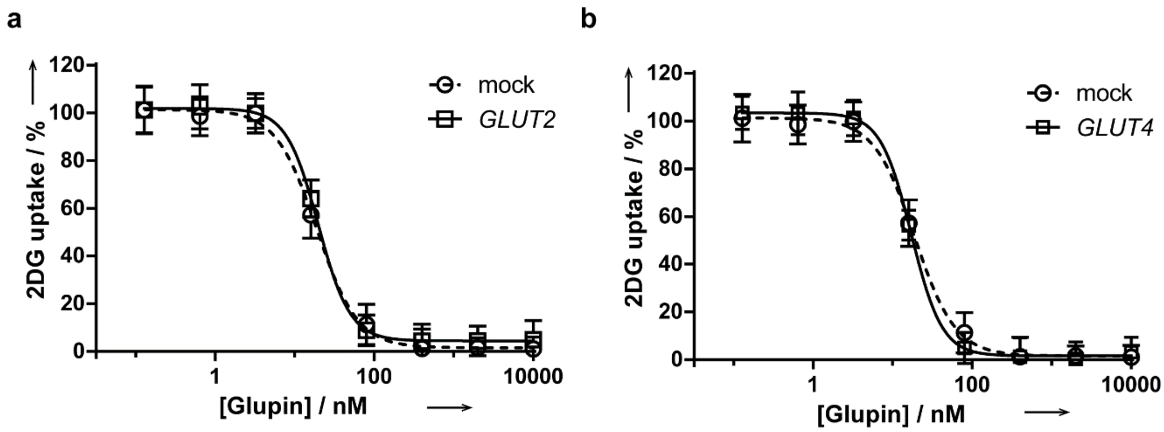

Supporting Figure S11. Influence of Glupin in GLUT-2 or GLUT-4 overexpression CHO cells. (a) Uptake of 2DG in CHO cells that ectopically express *GLUT2* as compared to cells transfected with an empty vector (mock). (b) Uptake of 2DG in CHO cells that ectopically express GLUT-3 as compared to cells transfected with an empty vector (mock). 2DG uptake was determined using the resazurin/diaphorase detection system. Data are mean values ± SD (N=3, n=3).

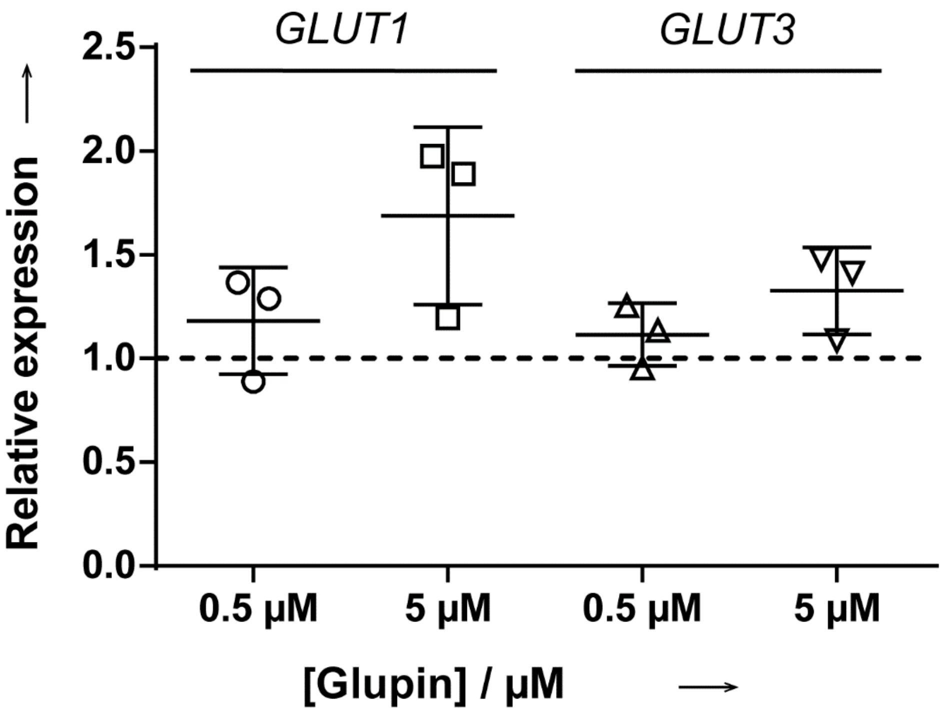

Supporting Figure S12: Influence of Glupin on the expression of GLUT-1 and GLUT-3. DLD-1 cells were treated with Glupin at 25 mM glucose for 48 h prior to isolation of total RNA and cDNA synthesis. Quantitative PCR was performed to assess the expression levels of *GLUT1* and *GLUT3* or *ATP1A1*, *TUBB* and *ACTB* as reference genes. Data are mean values of n=3 ± SD.

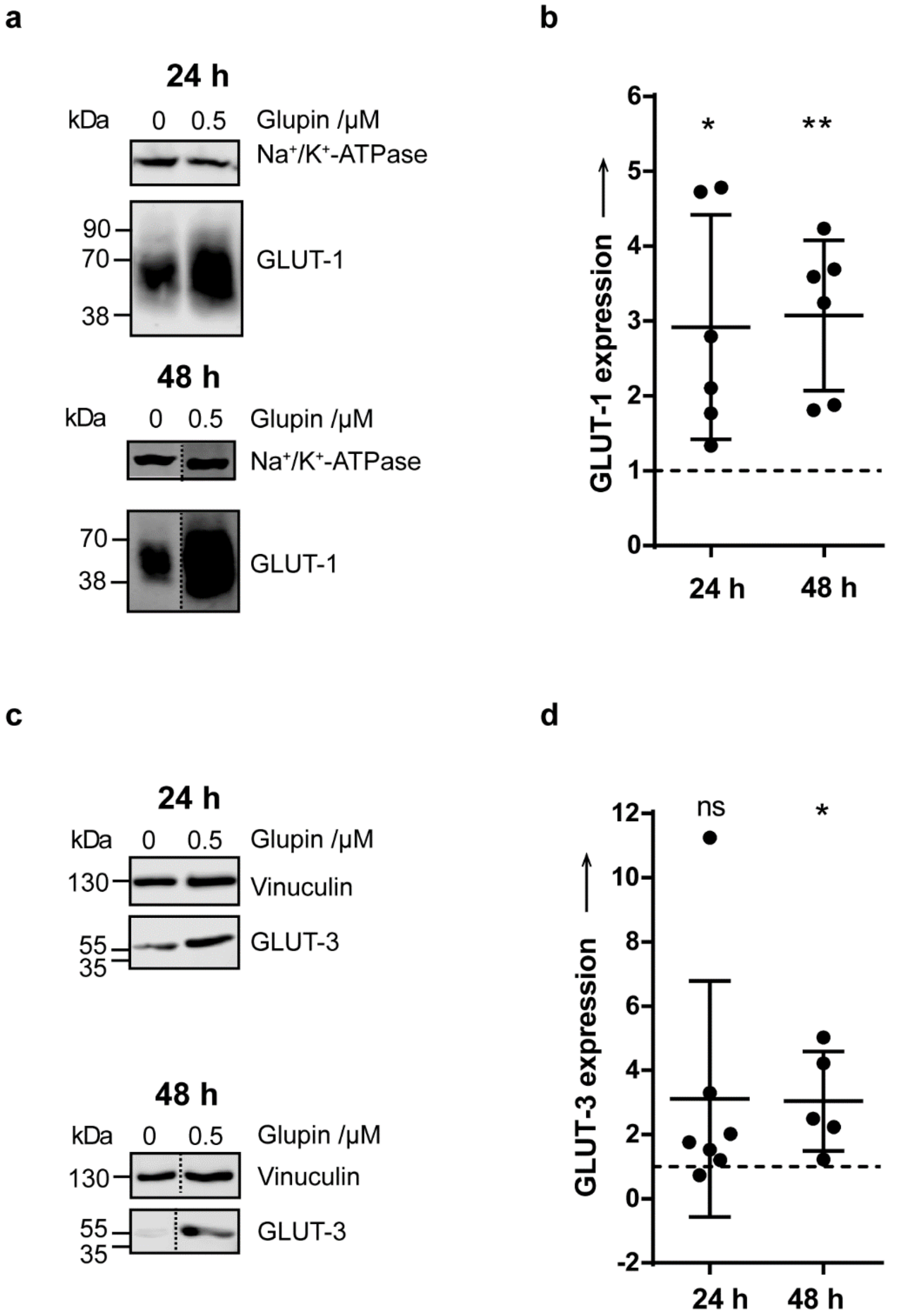

Supporting Figure 13: Influence of Glupin on the expression of GLUT-1 and GLUT-3. DLD-1 cells were treated with 0.5 µM Glupin at 25 mM glucose for 24 or 48 h prior to cell lysis, SDS-PAGE and immunoblotting for GLUT-1 (a and b) or GLUT-3 (c and d) and Na^+/K^+-ATPase or Vinculin as a control. Immunoblots are representative of at least six independent experiments. A dashed line indicates cropped immunoblot images from the same blot. (b and d) Quantification of the data from a and c. Data are mean

values (n=6 for b and n=6-8 for d) ± SD. Statistical analysis was performed using unpaired two-tailed t-test with Welch's correction. ns: not significant; *: $p<0.05$; **: $p<0.01$.

Supporting Tables

Supporting Table S3: Influence of (±)-Glupin on metabolites in Molt16 cells. Data is displayed as fold changes of significantly altered metabolites (p = 0.05, n=2) in MOLT16 cells treated with 50 nM (±)-Glupin for 24 h compared to DMSO controls.

**Metabolites**	**Fold change**	**p-value**
citric acid / isocitric acid	0.256	0.0011
lactic acid	0.472	0.0010
glycerol-3-phosphate	0.556	0.0113
ribitol/arabitol	0.570	0.0064
UDP-N-acetylglucosamine	0.630	0.0030
lanosterol	0.641	0.0116
beta-alanine	0.724	0.0414
succinic acid	0.858	0.0169
proline	1.141	0.0210
5,6-dihydrouracil	1.150	0.0471
glycine	1.333	0.0318
aspartic acid	2.297	0.0212

Supporting Table S4: IC50 values for inhibition of glucose uptake in CHO and GLUT-1-4 overexpressing cell lines. Influence of GLUT-1-4 overexpression on the activity of Glupin in CHO cells. CHO cells were transfected with the respective expression plasmids prior to treatment with the compound and determination of 2DG uptake using the diaphorase/resazurin system. Data are mean values ± S.D. (N=3, n=3).

**Plasmid**	**IC_{50} / nM**
pTCN	19.2 ± 2.1
pTCN-GLUT1	162 ± 14
pTCN-GLUT2	19.7 ± 2.4
pTCN-GLUT4	18.9 ± 3.2
pCMV-SPORT6	19.1 ± 2.9
pCMV-SPORT6-GLUT3	68 ± 7.2

Supporting Table S5: IC50 values for inhibition of cell growth by Glupin in a panel of 94 cell lines.

Cell growth was determined after 72 h of compound treatment using a sulforhodamine B assay.

**Cell line**	**Cell growth inhibition IC_{50}/μM**	**Tissue**
UMUC3	0.032	bladder
MIAPACA2	0.061	pancreas
WSUNHL	0.062	hematological
SKNSH	0.084	brain
SUDHL6	0.092	hematological
UO31	0.109	kidney
HL60	0.112	hematological
JIMT1	0.113	breast
5637	0.114	bladder
HT1080	0.118	connective tissue
HS578T	0.120	breast
K562	0.137	hematological
SKNAS	0.175	brain
RAMOS	0.212	hematological
SUDHL10	0.219	hematological

**Cell line**	**Cell growth inhibition IC_{50}/µM**	**Tissue**
A431	0.230	skin
CLS439	0.240	bladder
T24	0.243	bladder
A2780	0.248	ovary
PANC1005	0.269	pancreas
786O	0.274	kidney
TE671	0.299	muscle
DU145	0.317	prostate
A549	0.320	lung
SF268	0.370	brain
HCT15	0.407	colon
SF295	0.427	brain
CASKI	0.433	cervix
HEK293	0.463	kidney
U2OS	0.502	bone
MDAMB435	0.513	skin
CALU6	0.538	lung
A204	0.562	muscle

Cell line	Cell growth inhibition IC_{50}/μM	Tissue
SKHEP1	0.569	liver
NCIH460	0.572	lung
BXPC3	0.591	pancreas
A375	0.659	skin
ASPC1	0.695	pancreas
SKLMS1	0.699	uterus
PANC1	0.706	pancreas
CAKI1	0.805	kidney
DLD1	0.825	colon
PC3	0.827	prostate
RD	0.842	muscle
JAR	0.886	placenta
JEG3	0.933	placenta
NCIH82	1.168	lung
HELA	1.249	cervix
EJ28	1.386	bladder
OVCAR4	1.408	ovary
HCT116	1.470	colon

Cell line	Cell growth inhibition IC$_{50}$/μM	Tissue
MHHES1	1.494	bone
C33A	1.497	cervix
IGROV1	1.759	ovary
MINO	1.766	hematological
MT3	2.545	breast
L363	2.868	hematological
HT29	3.353	colon
CACO2	3.510	colon
A673	3.640	muscle
RDES	3.712	bone
COLO205	3.728	colon
MDAMB468	4.323	breast
LOVO	5.410	colon
22RV1	5.569	prostate
SKOV3	6.071	ovary
J82	7.250	bladder
MDAMB231	8.378	breast
SNB75	9.131	brain

Cell line	Cell growth inhibition IC$_{50}$/μM	Tissue
MDAMB436	11.106	breast
MG63	30.000	bone
MCF7	30.000	breast
BT20	30.000	breast
U87MG	30.000	brain
ACHN	30.000	kidney
IMR90	30.000	lung
SAOS2	30.000	bone
PLCPRF5	30.000	liver
KASUMI1	30.000	hematological
SKMEL5	30.000	skin
COLO678	30.000	colon
MV411	30.000	hematological
SKMEL28	30.000	skin
HEPG2	30.000	liver
HS729	30.000	muscle
GRANTA519	30.000	hematological
SW620	30.000	colon

**Cell line**	**Cell growth inhibition IC_{50}/μM**	**Tissue**
THP1	30.000	hematological
NCIH292	30.000	lung
EFO21	30.000	ovary
SKBR3	30.000	breast
PBMC	30.000	hematological
NCIH358M	30.000	lung
OVCAR3	30.000	ovary

Supporting Table S6: IC50 values for inhibition of cell growth by Glupin in a panel of 94 cell lines.

Cell growth was determined after 72 h of compound treatment using a sulforhodamine B assay.

**Cell line**	**Cell growth inhibition IC50 /µM**	**Tissue**
UMUC3	0.032	bladder
5637	0.114	
CLS439	0.240	
T24	0.243	
EJ28	1.386	
J82	7.250	
U2OS	0.502	bone
MHHES1	1.494	
RDES	3.712	
MG63	30.000	
SAOS2	30.000	
SKNSH	0.084	brain
SKNAS	0.175	
SF268	0.370	
SF295	0.427	
SNB75	9.131	
U87MG	30.000	
JIMT1	0.113	breast
HS578T	0.120	

Cell line	Cell growth inhibition IC50 /µM	Tissue
MT3	2.545	
MDAMB468	4.323	
MDAMB231	8.378	
MDAMB436	11.106	
MCF7	30.000	
BT20	30.000	
SKBR3	30.000	
CASKI	0.433	Cervix
HELA	1.249	
C33A	1.497	
HCT15	0.407	Colon
DLD1	0.825	
HCT116	1.470	
HT29	3.353	
CACO2	3.510	
COLO205	3.728	
LOVO	5.410	
COLO678	30.000	
SW620	30.000	
HT1080	0.118	connective tissue
WSUNHL	0.062	hematological

Cell line	Cell growth inhibition IC50 /µM	Tissue
SUDHL6	0.092	
HL60	0.112	
K562	0.137	
RAMOS	0.212	
SUDHL10	0.219	
MINO	1.766	
L363	2.868	
KASUMI1	30.000	
MV411	30.000	
GRANTA519	30.000	
THP1	30.000	
PBMC	30.000	
UO31	0.109	kidney
786O	0.274	
HEK293	0.463	
CAKI1	0.805	
ACHN	30.000	
SKHEP1	0.569	liver
PLCPRF5	30.000	
HEPG2	30.000	
A549	0.320	lung

Cell line	Cell growth inhibition IC50 /µM	Tissue
CALU6	0.538	
NCIH460	0.572	
NCIH82	1.168	
IMR90	30.000	
NCIH292	30.000	
NCIH358M	30.000	
TE671	0.299	muscle
A204	0.562	
RD	0.842	
A673	3.640	
HS729	30.000	
A2780	0.248	ovary
OVCAR4	1.408	
IGROV1	1.759	
SKOV3	6.071	
EFO21	30.000	
OVCAR3	30.000	
MIAPACA2	0.061	pancreas
PANC1005	0.269	
BXPC3	0.591	
ASPC1	0.695	

Cell line	Cell growth inhibition IC50 /µM	Tissue
PANC1	0.706	
JAR	0.886	placenta
JEG3	0.933	
DU145	0.317	prostate
PC3	0.827	
22RV1	5.569	
A431	0.230	skin
MDAMB435	0.513	
A375	0.659	
SKMEL5	30.000	
SKMEL28	30.000	
SKLMS1	0.699	uterus

Experimental

Cell culture

All cell lines except from SW480, MOLT-16, DLD-1 wt, DLD-1 GLUT-1 (-/-) and MCF7-LC3-GFP were grown in high glucose Dulbecco's modified Eagle's medium (DMEM, PAN Biotech) supplemented with 10% fetal calf serum (FCS, Invitrogen-Gibco) with 1% non-essential amino acids (PAN Biotech) and 1% Penicillin/Streptomycin (PAN Biotech). SW480 cells were cultured in L-15 medium (DMEM, PAN Biotech) supplemented with 10% fetal calf serum (FCS, Invitrogen-Gibco) and 1% Penicillin/Streptomycin (PAN Biotech). MOLT-16 cells were cultured in RPMI-1640 : FBS 9:1 + 50 U / mL Penicillin-Streptomycin. DLD-1 wt and GLUT-1 (-/-) cells (Horizon Discoveries, Cambridge, UK) were cultured in RPMI1640 medium supplemented with 10% fetal calf serum (FCS, Invitrogen-Gibco) and 1% Penicillin/Streptomycin (PAN Biotech). MCF7 cells stably transfected with EGFP-LC3 (MCF7-GFP-LC3) were cultured in Eagle's MEM (PAN Biotech) containing 10% FBS (Invitrogen-Gibco), sodium pyruvate (PAN Biotech), non-essential amino acids (PAN Biotech), 0.01 mg/mL bovine insulin (Sigma Aldrich) and 200 µg/mL G418. All cell lines were grown at 37°C in a humidified atmosphere and 5% CO_2.

Resazurin-based 2DG uptake assay (semi-automated high throughput)

All buffers, reagents and steps were identical with those described for the manual screening Washing and liquid addition steps were done using an automated cell washer or a multidrop device, respectively. Screening was performed in a 384-well plates. 15,000 HCT116 cells/well seeded into 384- well black-walled, clear bottomed plates (CellBIND, Corning) and incubated overnight. Compounds were added at 30 µM (final concentration for screening or serial dilutions staring from 30 µM for IC_{50} determinations) from DMSO stocks using the acoustic nanoliter dispenser ECHO 520. Fluorescence intensity was read from the bottom with a SpectraMax Paradigm Multi-Mode Microplate Reader (Molecular devices) using

the Rhodamine filter settings. Hit compounds were subjected to cell viability assay as determined using the CellTiter Glo reagent (Promega) using HCT116 cells and a treatment for 30 min.

For luminescence as an orthogonal 2DG uptake readout, cells were treated as described above. However, instead the detection enzyme mix, a modified version of NADP/NADPH-Glo™ Assay-solution (Promega GmbH, Germany) was added. The kit's components referred to as "NADH cycling enzyme" and "NADH cycling substrate" in the manual were left out and replaced by 6U/mL glucose-6-phosphate dehydrogenase. Volumes and buffers were used as described in the manufacturer's protocol for the individual detection of $NADP^+$. Luminescence was read after 15 min.

Resazurin-based 2DG uptake assay (low throughput)

The 2DG uptake assay was performed by the Yamamoto *et al.* procedure.[6] 40000 cells/well were seeded in black CellBIND 96-well plates with clear bottom (Corning) and allowed to attach overnight. Cells were washed three times with KRB buffer (20 mM Hepes, 5 mM KH_2PO_4, 1 mM $MgCl_2$, 1 mM $CaCl_2$, 136 mM NaCl, 4.7 mM KCl, pH 7.4) containing 0.1% BSA and then incubated with the compounds or vehicle control and 1 mM 2DG for 30 min as described earlier. After washing three times with KRB buffer, the plates were sealed with adhesive aluminum foil and cells were lysed with 60 µL 1% CHAPS in 0.06 M HCl. Subsequently, plates were heated using microplate heater for 15 min at 65°C. After cooling down to room temperature, 20 µL 0.5 M Tris were added to neutralize the acid, before 80 µL of detection enzyme mixture were added. The detection enzyme solution contained 25 µM resazurin sodium salt (Acros), 6 U/mL glucose-6-phosphate dehydrogenase (G8404; Sigma Aldrich), 0.2 U/mL diaphorase from *Chlostridium kluyveri* (D5540; Sigma Aldrich) and 0.1mM $NADP^+$ (Applichem) in 0.125 mM Tris, pH 8.4 with 0.1% BSA. After incubation for 2 h at room temperature, fluorescence intensity was measured at ex/em 530/590 nm using a Tecan M200 plate reader. Data were normalized to the respective DMSO

control (which was set to 100 %) and cells without 2DG (set to 0 %) using Graph Pad Prism 5 or 6 software and IC_{50} values were determined using a four-parameter fit.
The observed difference in the obtained IC_{50} values for Glupin in the automated screening vs. manually performed assay is most likely attributed to the minituarization that is required for high-troughput screening and usually requires different plate format (384 vs. 96-well plates) and different cell number. In this case, the automated assay was performed in 384-well plates (vs. 96-well plates for the manual assay) with 15,000 cells per well (vs. 40,000 cells per well in the 96-well plates).

For kinetic measurements, 15,000 cells were seeded into black CellBIND 384-well plates (Corning) and allowed to attach overnight. Then, the plates were washed 3X with KRB buffer containing 0.1%BSA and treated with different concentrations of 2DG (0-60mM) and Glupin (0-1µM) for 30s to 30min. The uptake was determined using the resazurin/diaphorase system as described above. 2DG uptake was stopped by aspirating the 2DG solution followed by quick wash with ice-cold KRB buffer (0.1 % BSA). Km and Vmax were determined by fitting the data according to Michaelis-Menten using non-linear fitting.

Hexokinase assay

HCT116 cells lysed with RIPA buffer (10 mM Tris-Cl (pH 8.0, 1 mM EDTA, 1% Triton X-100, 0.1% sodium deoxycholate, 0.1% SDS, 140 mM NaCl, 1 mM phenylmethylsulfonyl fluoride (PMSF) and protease inhibitor cocktail (Roche)) on ice for 5 min. The lysate was centrifuged (500 x g, 4 °C, 10 min), the supernatant was transferred to a fresh tube and snap frozen and stored at -80 °C. The assay was performed in a black 96 well plate in 100 µL total volume. 10% (v/v) cell lysate (conc. 0.04 mg/mL) were supplemented with 0.3 mM ATP, 10% (v/v) of 2-DG (concentration range 0-15.6 mM), 43% (v/v) enzyme mix (0.1 mM $NADP^+$, 16 U/mL G6PDH in assay buffer) and 10% (v/v) compound (Glupin or 3-

bromopyruvate as a control)(Matsushita et al., 2012) and filled up with assay buffer (100 mM Tris, 5 mM $MgCl_2$, pH 8.5). Samples were incubated for 40 min at 37 °C. Fluorescence intensity of NADPH+H^+ (ex/em 340 nm/445 nm) was measured with the Tecan Infinite M200 plate reader (Tecan). The background was subtracted and all values were normalized to the maximal fluorescence intensity at 15.6 mM 2-DG.

^{3}H-2DG uptake assay

Cells were seeded into clear cell culture-treated plates and grown to approx. 80% confluence. Cells were then washed three times with KRB buffer and incubated for 30 min with compounds and 1 µCi/mL 3H-2DG (PerkinElmer LAS, Germany). Extracellular ^{3}H-2DG was removed by washing three times with ice-cold KRB buffer. Cells were then lysed in 0.1 M NaOH for 30 min. 200 µL of the lysate was added to 2 mL of Emulsifier-Safe scintillation liquid (PerkinElmer LAS, Rodgau, Germany) and uptake was quantified using a Wallac 1409 scintillation counter (PerkinElmer LAS, Rodgau, Germany). Normalization to the amount of total protein levels was pereformed using a Bradford Assay Reagent (Bio-Rad).

Seahorse measurements

To determine the glycolysis rate upon compound treatment, the extracellular acidification rate (ECAR) and the oxygen consumption rate (OCR) was measured using the Seahorse XFp Extracellular flux analyzer (Agilent) in combination with a Glycolysis Stress Test Kit (Agilent). 250,000 MDA-MB-231 cells/well were seeded in DMEM with 10% FBS into Seahorse XFp Cell Culture Miniplates (Agilent) and incubated overnight. XFp sensor cartridges were hydrated according to the manufacturer's protocol. Next day, medium was exchanged for DMEM XF Base Medium (pH 7.4) containing 2 mM GlutaMAX

(Gibco) and cells were starved for 45 min at 37°C in a CO_2-free incubator. OCR and ECAR were monitored in six minute intervals. After three initial intervals (in total 15 min), test compounds were injected, followed by five measuring intervals. Afterwards 1 mM glucose, 2 µM oligomycin and 100 mM 2-deoxyglucose were injected sequentially, each injection followed by three measuring intervals. OCR and ECAR data were normalized to the last measurement before test compound injection.

Metabolite profiling

For each data point approximately 4 * 10^6 MOLT-16 cells in log phase were cultured in 6 mL medium in a 6-well culture plate. Cells were either treated with 0.1% DMSO (controls) or 50 nM (±)-Glupin and 0.1% DMSO. After 24 h the cell densities were determined using counting chambers. Working on ice, cell suspensions were transferred to centrifuge tubes and centrifuged at 200 g for 10 min at 4°C. Cell pellets were washed twice with 2 mL cold PBS followed by centrifugation and removal of the supernatant. Cell pellets were frozen and stored at -80 °C before extraction of the metabolites. Cell pellets were then thawed on ice and 400 µL extraction mix (methanol:water 9:1 + internal standards) were added and the samples were transferred into Eppendorf tubes. Two tungsten carbide beads were added to each tube and the samples were extracted with a Retsch MM301 vibration mill at 30 Hz for two minutes. After storage on ice for 45 min the tungsten beads were removed and the samples were centrifuged at 14000 rpm (18620 g) for 15 min at 4 °C. 150 µL of the supernatants were transferred to vials and concentrated under reduced pressure to complete dryness and then stored at -80 °C. Prior to GC-MS analysis the samples were derivatized. The cell pellets were thawed on ice and then dried under reduced pressure at room temperature for 20 min. 20 µL of a 15 µg/µL methoxamine solution in pyridine was added and the samples were dissolved by shaking them for 10 min and they were then allowed to stand for 20 h at room

temperature. 20 µL MSTFA with 1% TMSCl was then added and after 1 h at room temperature, 20 µL heptane containing 15 ng/µL methyl stearate was added.

The samples were analyzed at the Swedish Metabolomics Center in Umeå, Sweden, using a Leco Pegasus HT time-of-flight mass spectrometer equipped with an Agilent 7890A gas chromatograph (GC) and a 30 m DB-5ms Ultra Inert GC-column with an inner diameter of 0.25 mm. Automated splitless injection of 1 µl sample was performed at an injection temperature of 270 °C. The purge time was 75 seconds with a rate of 20 mL/min. Helium was used as carrier gas (1 mL/min). The primary GC oven temperature was 70 °C for 2 minutes and then increased 20 °C/minute to 320 °C, where it was held constant for 8 minutes. The transfer line temperature between the gas chromatograph and mass spectrometer was 250 °C. The ion source temperature was 200 °C and the electron impact ionization energy was 70 eV. Mass spectra were collected at 20 Hz in the mass range 50 to 800 m/z and the detector voltage vas 1670 V. A series of n-alkanes (C8-C40) were used as external retention index standards.

The raw GC-chromatograms were aligned using the internal standards and the GC-MS data was compared against an in-house spectral library using the in-house RDA software (Swedish Metabolomics Centre, Umeå, Sweden). The data was then curated using NIST MS search v2.0. The annotated integrated data was normalized against the cell density and the injection standard, methyl stearate, in each sample. For statistical evaluation, two-sided student's t-tests were performed in Microsoft Excel, assuming equal variance.

Cellular thermal shift assay (CETSA)

SW480 cell lysates were prepared according to the following procedure: nearly confluent cells were washed with PBS and detached using trypsin (0.05% Trypsin, 0.02% EDTA; PAN Biotech). The trypsin was inactivated with media and the cell suspension was centrifuged at 1,200 rpm. The cell pellet was

resuspended in lysis buffer (PBS, 0.4% NP40 alternative, EDTA-free Protease Inhibitor Cocktail (Roche)) and stored on ice. The cell suspension was subjected to four freeze-and-thaw cycles and short sonication (10 s). After ultracentrifugation at 4 °C (20 min, 100,000 x g) the supernatant was collected, the protein concentration was measured by means of Bradford and stored snap frozen at -80 °C. For CETSA and Thermal Proteomic Profiling (TPP) cell lysates were diluted to 2 mg/mL and divided into two reaction tubes. One fraction was treated with 10 µM Glupin, the other with 1% (v/v) DMSO and incubated for 10 min at room temperature respectively. The treated lysates were split in 10 fractions each and subjected a temperature gradient (36.9-67°C) for 3 min. The lysates were centrifuged at 100,000 x g for 20 min and 4°C and the soluble fractions were analyzed using immunoblotting (CETSA) or 10plex TMT labeling for mass spectrometry based readout.[8] For TMT-labeling the samples were reduced with TCEP, alkylated with iodoacetamide, precipitated with acetone and tryptic digested in TEAB buffer overnight. Afterwards samples were spinned down and labeled with TMT label according to the description of the manufacturer, but using just half the amount of labeling reagent. 120 µl of each labeled aliquot incubated with Glupin were combined into one sample and 120 µl of each DMSO aliquot into a second one. Both samples were evaporated to dryness. Prefractionation of samples using high pH conditions, nanoHPLC-MS/MS analysis, and data evaluation using MaxQuant software[9] (v.1.5.3.30) and an in-house programmed excel macro to calculate melting curves were performed as described by Martin-Gago et al.[10] The mean values of the melting temperatures of GLUT1-1 and GLUT-3 of two biological replicates were calculated and a Boltzmann fit was performed.

Transient overexpression of GLUT-1-4 in CHO cells and 2DG assay[7]

1x10^6 CHO cells were seeded 10 cm cell culture plates. After overnight incubation, cells were transfected with the different using Lipofectamine 3000 according to the supplier's instructions. Briefly, DNA-lipid

complexes (100 µg plasmid DNA, DNA:lipid ratio 1:2) were prepared in OptiMEM and then added to the cells. In the case of GLUT-4-transfected cells, Insulin (100 µg/mL) was added to the transfection medium. After incubation for 48 h cells were either reseeded (40,000 cell/well) in 96-well plates for monitoring glucose uptake or cells were directly lysed and protein expression was subsequently analysed by means of immunoblotting. In the case of GLUT-4 transfected cells, Insulin (100 µg/mL) was added to the culture medium medium.

The 2DG assay was performed as described above (low throughput resazurin-based assay). In the case of GLUT-4 transfected cells, Insulin (100 µg/mL) was added to the buffer.

Real-time live-cell analysis

MDA-MB-231 cells were seeded at a density of 2,500 cells/well into clear 96-well plates and allowed to adhere overnight. Increasing concentrations of compounds or vehicle control were added to the cells in fresh medium containing 0 mM, 5 mM or 25 mM glucose, respectively and analysed in real-time using an IncuCyte ZOOM Live-Cell Imaging System (Essen Bioscience, UK) at 20x or 10x magnification for 48 h. Images were acquired in phase contrast and green fluorescence mode every hour. Images were analyzed using the IncuCyte Zoom 2016/A software by automated image segmentation. For confluence measurements, data were normalized to confluence at time point t=0.

Detection of lipid droplets

MDA-MB-231 cells were seeded in black 96-well plates with clear bottom (Corning) and incubated overnight. The growth medium was then replaced with serum-reduced medium (5% FBS) containing the desired concentration of the test substance or vehicle control and oleic acid (400 µM final concentration). After treatment for 18 h cells were fixed with 3.7% paraformaldehyde in PBS, permeabilized with 0.1%

Triton-X 100 and stained for 1-2 h at room temperature using 0.5 µg/mL BODIPY® 493/503 neutral lipid stain (Invitrogen) for visualization of lipid droplets and with 1µg/mL Hoechst33342 (Invitrogen) to stain the DNA.

For assaying mobilization of existent lipid droplets, MDA-MB-231 cells were pretreated with 400 µM oleic acid overnight prior to addition of the compound.

RT-qPCR

DLD-1 cells were seeded with a density of $6.3*10^4$ cells/well in a 12 well plate and allowed to adhere for 24 hours. After treatment with 5 or 0.5 µM Glupin (RPMI medum) or different glucose concentrations (DMEM medum) for the indicated times, total RNA was isolated using RNeasy Mini Kit following the instructions provided by the manufacturer (Qiagen). The reverse transcription was performed using Quantitect Reverse transcription kit (Qiagen). Qunatitative PCR was done using Sso Advanced SYBR Green Mix (Bio-Rad) in a iCycler iQ5 thermal cycler (Bio-Rad). Primer sequences and amplicon sizes are depicted below.

Gene	Forward primer (5′-3′)	Reverse primer (5′-3′)	Product size, bp
GLUT1	TTGCAGGCTTCTCCAACTGGAC	CAGAACCAGGAGCACAGTGAAG	112
GLUT3	TGCCTTTGGCACTCTCAACCAG	GCCATAGCTCTTCAGACCCAAG	97
Actin	CACCATTGGCAATGAGCGGTTC	AGGTCTTTGCGGATGTCCACGT	134
TUBB	CTGGACCGCATCTCTGTGTACT	GCCAAAAGGACCTGAGCGAACA	116
ATP1A1	GGCAGTGTTTCAGGCTAACCAG	TCTCCTTCACGGAACCACAGCA	118

Data were normalized to the levels of Actin, TUBB and ATP1A1 using the $\Delta\Delta C_t$-method. [11]

Immunoblotting

200,000 CHO cells were seeded in 6-well plates, and transiently transfected as described above and lysed in 1.5xSDS sample buffer without bromophenol blue. Protein concentrations were determined using the DC Assay (Bio-rad) according to the manuacturer's instructions. Proteins were resolved by means of SDS-PAGE and then transferred to PVDF membrane using semi-dry transfer. Membranes were blocked in 5% skimmed milk in 0.1% Tween20 in Tris-buffered saline (TBS-T, blocking buffer) for 1 h at room temperature prior to incubation with the primary antibody in blocking buffer overnight at 4 °C or for one hour at room temperature. After washing with TBS-T, membranes were incubated with the secondary antibody, which was coupled to horseradish peroxidase (HRP) in blocking buffer for one hour at room temperature. Signals were visualized using the SuperSignal West Pico Chemiluminescent Substrate or the SuperSignal West Femto Maximum Sensitivity Substrate (Thermo Fischer) on the Odyssey Fc imaging system (Li-COR). Densitometric algorithms were used for protein band quantification.

For analysis GLUT-1 and GLUT-3 expression in DLD1 cells, cells were seeded with a density of $3.06*10^4$/well (24 h) or $1.53*10^4$/well (48 h, 72 h) 24 h prior the experiment in a 6 Well plate. The cells were treated for the indicated time points. Prior to lysis the cells were washed once with PBS, detached using cell-dissociation solution (Sigma Aldrich) and washed once more with PBS followed by a centrifugation step (4 °C, 5 min, 1,200 rpm). The pellet was lysed using 2% dodecyl-maltosid in phosphate-buffered saline (PBS) for 15 min on ice. After centrifugation at 14,000 rpm (4 °C, 15 min) the lysates were collected and the protein concentration was measured using DC protein assay (Bio-Rad) or Bradford reagent (Bio-Rad). Afterwards the samples were loaded on a 6% SDS gel. The proteins were transferred on a PVDF membrane by means of semi-dry electrophoretic transfer (Bio-Rad). The membranes were blocked using Odyssey® blocking buffer (Li-Cor) for 1 h at room temperature. All antibody solutions were prepared in Odyssey® blocking buffer. The proteins GLUT-1 and GLUT-3 were detected using the primary antibodies anti-GLUT-1 (mouse; dilution factor 1:5,000; #ab40084; Abcam) and anti-GLUT-3 (rabbit; dilution factor 1:5,000; #ab191071; Abcam). Na^+/K^+-ATPase and Vinculin

served as loading controls for GLUT-1 and GLUT-3 respectively (anti-Na^+/K^+-ATPase; rabbit; dilution factor 1:10,000; #ab76020; Abcam / anti-Vinculin; mouse; dilution factor 1:5,000; #sc-59803; Santa Cruz). After overnight incubation of the primary antibody the membranes were washed three times with PBS. A solution containing infrared dye-labeled 800CW anti-mouse (donkey; dilution factor 1:5,000; #926-32212, Li-Cor) and 680RD anti-rabbit secondary antibody (donkey; dilution factor 1:5,000; #926-68071, Li-Cor) was used to detect GLUT-1 and Na^+/K^+-ATPase and a solution of 800CW anti-rabbit (donkey; dilution factor 1:5,000; #926-32213, Li-Cor) and 680RD anti-mouse secondary antibody (donkey; dilution factor 1:5,000; #926-68072, Li-Cor) to detect GLUT-3 and Vinculin. The membranes were incubated with the secondary antibody solutions for 1 h at room temperature. The membranes were washed two times using PBS-Tween20 (0.1% v/v) and once using PBS before visualization by means of Li-Cor Odyssey® CLx. Densitometric algorithms were used for protein band quantification (Image Studio Ver. 5.2).

Cell panel profiling

A panel of 94 cell lines was exposed to Glupin for 72 h to assess the influence on cell growth (Oncolead, Germany). Cell growth was determined using sulforhodamine B assay. Measurements were performed using a total protein staining protocol [12]. Briefly, after treatement cells were fixed addition of 10% TCA (for adherent growing cells) or 50% TCA (for semi-adherent growing cells or cells growing in suspension). After an incubation at 4°C for 1 h, plates were washed twice with 400 µl of deionized water and dried. Cells were then stained with 100 µl of 0.04% wt/v sulforhodamine B. Plates were incubated at room temperature for at least 30 min and washed six times with 1% acetic acid to remove unbound stain. Plates were left to dry at room temperature and bound SRB was solubilized with 100 µl of 10 mM Tris.

Optical density was measured at 492, 520 and 560 nm using a Deelux-LED96 plate reader (Deelux Labortechnik GmbH, Germany).

Statistics

Data from independent experiments (n) are presented as mean values ± standard deviation (SD). *N* is the number of technical replicates and *n* is the number of biological replicates. Data fitting was performed using GraphPad Prism 6.0. Statistical analysis was performed using unpaired t test with Welch's correction (GraphPad Prism 6.0).

References

[1] F. Diaba, J. Bonjoch, *Org Biomol Chem* **2009**, *7*, 2517.

[2] P. D. Johnson, J. H. Sohn, V. H. Rawal, *J Org Chem* **2006**, *71*, 7899.

[3] M. G. Johnson, J. J. Liu, A. R. Li, B. van Lengerich, S. Wang, J. C. Medina, T. L. Collins, J. Danao, L. Seitz, A. Willee, W. D'Souza, A. L. Budelsky, P. W. Fan, S. G. Wong, *Bioorganic & medicinal chemistry letters* **2014**, *24*, 2877.

[4] D. E. Gingrich, D. R. Reddy, M. A. Iqbal, J. Singh, L. D. Aimone, T. S. Angeles, M. Albom, S. Yang, M. A. Ator, S. L. Meyer, C. Robinson, B. A. Ruggeri, C. A. Dionne, J. L. Vaught, J. P. Mallamo, R. L. Hudkins, *Journal of medicinal chemistry* **2003**, *46*, 5375.

[5] W. H. Sauer, M. K. Schwarz, *J Chem Inf Comput Sci* **2003**, *43*, 987.

[6] a) N. Yamamoto, K. Kawasaki, K. Kawabata, H. Ashida, *Analytical biochemistry* **2010**, *404*, 238; b) N. Yamamoto, T. Sato, K. Kawasaki, S. Murosaki, Y. Yamamoto, *Analytical biochemistry* **2006**, *351*, 139.

[7] G. Karageorgis, Reckzeh, E.S.,Ceballos, J., Schwalfenberg, M., Sievers, S., Ostermann, C., Pahl, A., Ziegler, S., Waldmann, H., *Nat Chem* **2018**, *10*, 1103.

[8] a) M. M. Savitski, F. B. Reinhard, H. Franken, T. Werner, M. F. Savitski, D. Eberhard, D. Martinez Molina, R. Jafari, R. B. Dovega, S. Klaeger, B. Kuster, P. Nordlund, M. Bantscheff, G. Drewes, *Science* **2014**, *346*, 1255784; b) F. B. Reinhard, D. Eberhard, T. Werner, H. Franken, D. Childs, C. Doce, M. F. Savitski, W. Huber, M. Bantscheff, M. M. Savitski, G. Drewes, *Nature methods* **2015**, *12*, 1129; c) H. Franken, T. Mathieson, D. Childs, G. M. Sweetman, T. Werner, I. Togel, C. Doce, S. Gade, M. Bantscheff, G. Drewes, F. B. Reinhard, W. Huber, M. M. Savitski, *Nature protocols* **2015**, *10*, 1567.

[9] J. Cox, M. Mann, *Nat Biotechnol* **2008**, *26*, 1367.

[10] P. Martin-Gago, E. K. Fansa, C. H. Klein, S. Murarka, P. Janning, M. Schurmann, M. Metz, S. Ismail, C. Schultz-Fademrecht, M. Baumann, P. I. H. Bastiaens, A. Wittinghofer, H. Waldmann, *Angew Chem Int Edit* **2017**, *56*, 2423.

[11] K. J. Livak, T. D. Schmittgen, *Methods* **2001**, *25*, 402.

[12] V. Vichai, K. Kirtikara, *Nature protocols* **2006**, *1*, 1112.
